# A bioinformatic analysis to systematically unveil shared pathways and molecular mechanisms underlying monkeypox and its predominant neurological manifestations

**DOI:** 10.3389/fcimb.2025.1506687

**Published:** 2025-07-02

**Authors:** Amir Hossein Barjasteh, Hanieh Latifi, Ali Sepehrinezhad

**Affiliations:** ^1^ Student Research Committee, Mashhad University of Medical Sciences, Mashhad, Iran; ^2^ Neuroscience Research Center, Mashhad University of Medical Sciences, Mashhad, Iran; ^3^ Department of Neuroscience, Faculty of Medicine, Mashhad University of Medical Sciences, Mashhad, Iran

**Keywords:** monkeypox, bioinformatics, natural killer cells, headache, T-helper cells, myalgia, photophobia, drug repurposing

## Abstract

**Background:**

Monkeypox (MPOX) is a zoonotic disease caused by the MPOX virus (MPXV). MPOX resurfaced globally in May 2022, spreading throughout six WHO regions, resulting in nearly 87,000 cases and 112 deaths. Clinical symptoms include swollen lymph nodes, fever, joint pain and several neurological complications such as headache, encephalitis, myalgia, fatigue, photophobia and seizures. Despite these manifestations, the precise mechanisms of MPXV’s neurotropism remain elusive. This study aimed to explore the genetic underpinnings of MPOX-related neurological manifestations, including headache, myalgia, fatigue, and photophobia, using advanced bioinformatics tools.

**Methods:**

Data were sourced from the GeneCards database, which is an integrated database of human genes. Genes linked to MPOX and its neurological manifestations were identified and cross-referenced to uncover shared genes between these conditions. Network visualization was created using STRING, followed by topological analysis in Cytoscape to identify key genes based on degree and betweenness centrality. Functional enrichment analysis through ToppGene provided insights into molecular functions, biological processes, and cellular components associated with these target genes. Pathway analysis was performed using WikiPathways, and cell-type-specific enrichment was conducted using Enrichr. Additionally, we predicted functional microRNAs using mirTarbase and identified potential drug candidates via the Stitch database.

**Results:**

We identified 32 MPOX-associated genes and a large set of neurological manifestation-related genes. Ten hub genes, including CD55, CXCL1, NFKB1, CXCL8, CD4, IL6, MX1, CFH, KLRK1, and CD46 were shared between MPOX and its neurological manifestations. Five novel genes, including CFHR3, C5AR1, C3AR1, IFNA2, and CXCL3 were predicted to be associated with MPOX and its neurological complications. Gene ontology analysis highlighted biological processes such as immune regulation, viral life cycle, and lymphocyte activation, while pathway enrichment identified critical signaling mechanisms like prostaglandin signaling, toll-like receptor 4 (TLR4) signaling, complement activation, and neuroinflammation. Moreover, cell types such as T-helper cells, natural killer cells, and microglia were found to be significantly impacted by MPOX and its frequent neurological complications. We identified 11 key microRNAs associated with MPOX-neurological manifestations and repurposed eight potential drugs, offering promising therapeutic strategies.

**Conclusion:**

This study emphasizes the central role of the complement system, immunological responses, and inflammatory pathways in the neurological manifestations of MPOX. The identification of novel genes and predicted therapeutic targets paves the way for future research and therapeutic interventions. Experimental validation is required to confirm these findings and determine the effectiveness of the proposed treatments.

## 1 Introduction

Monkeypox (MPOX) is an emerging zoonotic disease caused by the monkeypox virus (MPXV), a member of the *Orthopoxvirus* genus within the *Poxviridae* family, closely related to the variola virus responsible for smallpox (El Eid et al., 2022; Karagoz et al., 2023).The virus was first identified in 1958 during research involving monkeys, hence its name. The first human case was reported in 1970 in a 9-month-old boy from the Democratic Republic of the Congo (DRC) (Ladnyj et al., 1972; Mileto et al., 2022). Since then, sporadic outbreaks have occurred primarily in endemic regions of Africa, with occasional cases reported outside the continent, including the United States in 2003 and cases in the United Kingdom, Singapore, and Israel between 2018 and 2019 (Ligon, 2004; Reynolds et al., 2007; Oladoye, 2021; Adler et al., 2022).

MPOX re-emerged in May 2022 and quickly spread over Europe, the Americas, and all six World Health Organization (WHO) regions, causing around 87,000 cases and 112 deaths in 110 countries (Al-Tammemi et al., 2022; Lim et al., 2023; Mitjà et al., 2023). While the primary reservoir of MPXV has yet to be found, rodents are the most likely candidates. Transmission occurs through direct or indirect contact with respiratory droplets, infected skin lesions, or body fluids from infected animals or humans, as well as via placenta transfer and sexual interaction (Guarner et al., 2004; Heskin et al., 2022; Riopelle et al., 2022).

Clinically, MPOX presents with symptoms such as swollen lymph nodes, fever, back pain, muscle pain, and headache, resembling a milder form of smallpox (Ahmed et al., 2023). Additionally, a wide range of neurological complications such as headache, encephalitis, myalgia, seizure, decreased hearing, visual changes, fatigue, photophobia, malaise, loss of appetite, and changes in consciousness have been observed sporadically in MPOX patients, which may be linked to the virus’s ability to penetrate brain tissue, as evidenced in certain infected animals (Badenoch et al., 2022; Billioux et al., 2022; Slomski, 2022; Khan et al., 2023). Neuroinvasion mechanisms remain poorly understood but may involve direct invasion via the olfactory epithelium and hematogenous spread via infected monocytes/macrophages have been suggested as two possible routes for viral neuroinvasion. Despite these hypotheses, the precise mechanisms of neurotropism remain unknown (Sepehrinezhad et al., 2023). Studies have demonstrated that the MPXV is capable of infecting human astrocytes, microglia, and neurons. Animal studies have also reported that replication of the virus is possible in brain parenchyma (Earl et al., 2012; Hutson et al., 2015; Sergeev et al., 2016; Chailangkarn et al., 2022; Miranzadeh Mahabadi et al., 2024; Bauer et al., 2023; Schultz-Pernice et al., 2023). Moreover, elevated levels of inflammatory markers such as interleukin-6 (IL-6), interleukin-1B (IL-1B), tumor necrosis factor alpha (TNFα) and C-X-C motif chemokine ligand 8 (CXCL8) in MPOX patients further support the hypothesis of an immune-mediated neurological pathogenesis (Johnston et al., 2015; Wong et al., 2018; Agrati et al., 2023).

Although more cases are now reporting neurological complications linked to MPOX, we still know very little about how the virus actually causes these manifestations at the molecular level. There are currently no treatments specifically designed to address the neurological complications of MPOX. While antivirals like tecovirimat and cidofovir have been approved for smallpox and are sometimes used in MPOX cases, it’s unclear how effective they are against viruses that can affect the brain (Das et al., 2023; Prévost et al., 2024). This gap in knowledge highlights the urgent need to better understand how MPXV interacts with the nervous system and to explore new therapeutic strategies that can help protect patients from these potentially serious complications.

In this context, our study utilizes advanced bioinformatics approaches to identify shared genes and molecular mechanisms between MPOX and its most prevalent neurological manifestations (headache, myalgia, fatigue, and photophobia). Through integrative analysis including gene network construction, pathway enrichment, cell-types and drug repurposing, we aim to uncover key immune and inflammatory pathways that may underlie MPOX-associated neurological manifestations and suggest viable intervention options for therapy development.

## 2 Methods

### 2.1 Dataset selection and collection of relevant genes

All genes utilized in this study were sourced from the GeneCards database (https://www.genecards.org/) (Stelzer et al., 2016; Grissa et al., 2022). GeneCards is an integrated database that contains a variety of proteomic, genomic, and transcriptomic information about human genes. We discovered after doing a thorough literature review that headache, myalgia, fatigue, and photophobia were the most common neurological manifestations of MPOX (Badenoch et al., 2022; Khan et al., 2023). Genes associated with these manifestations and MPOX were cross-referenced from GeneCards and literature on MPOX. Subsequently, we exported all gene sets to an Excel file to uncover shared genes between MPOX and neurological manifestation’s linked genes. All subsequent analyses focused on the shared genes between MPOX-related and neurological manifestations-associated genes (target genes).

### 2.2 Network visualization of target genes and predicting novel associated genes

To visualize and predict protein-protein interactions in the *Homo sapiens* organism, we submitted our target genes to STRING (https://string-db.org/). The resulting network was then imported into Cytoscape version 3.10.1 for analysis of the key topological features, such as degree, and betweenness centrality. These characteristics aid in identifying the most influential genes in the network: nodes with the most connections (degree) and genes in the most central positions (betweenness). According to gene-gene interaction data in STRING we also predicted some novel genes for MPOX-neurological manifestations shared genes.

### 2.3 Functional analysis, microRNA prediction and drug repurposing

To identify and predict potentially relevant functional mechanisms for our target genes, we performed an integrative enrichment analysis with ToppGene (https://toppgene.cchmc.org/). Different functional enrichment was carried out using the ToppFun panel in the ToppGene online tool (Ghanbarzehi et al., 2023). Each gene set was individually enriched for gene ontology (GO), which includes molecular function, biological activity, and cellular component. Pathway analysis was performed through WikiPathways in ToppGene. Cell-type specific enrichment analysis was also conducted through Cell-marker 2024 section in Enrichr (https://maayanlab.cloud/Enrichr/). Importantly, we also predicted several functional miRNAs for the MPOX-neurological manifestations associated genes using mirTarbase in ToppGene. Subsequently, we repurposed some potentially effective drugs for our target genes using stitch database in ToppGene. We used a p-value cut-off of ≤0.05 in all statistical analyses. All results were presented as -log (p-value) to enable visualization of both high and low significance values on a linear scale and displayed using GraphPad Prism version 9 or Biorender.com.

## 3 Results

### 3.1 Identified gene sets and reconstructed genetic network

**Figure 1:**
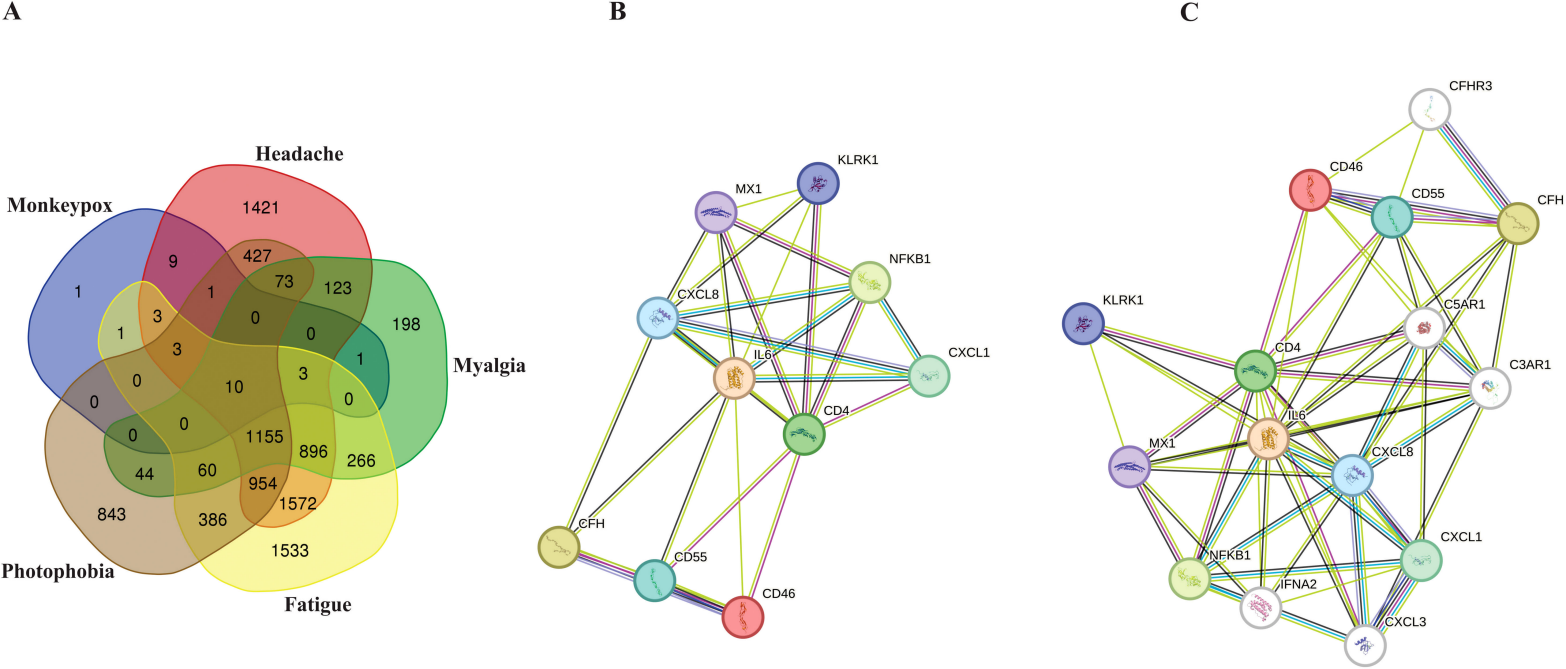
Hub genes associated with MPOX and its neurological manifestations and its reconstructed genetic networks. **(A)** Venn diagram represents the distribution of genes associated with MPOX and its neurological manifestations in five distinct categories. **(B)** Genetic network of hub genes associated with MPOX and its neurological manifestations. **(C)** Genetic network of hub genes associated with MPOX and its neurological manifestations and predicted five novel associated genes (white nodes). Edge colors indicating interaction types: light blue, known interactions from curated databases; pink, known interactions from experimentally determined; green, predicted interactions from gene neighborhood analysis; dark blue, predicted interactions from gene co-occurrence; yellow, interactions inferred from text mining; black, co-expression-based interactions.

According to extracted data from GeneCards, 32 genes were detected to be linked to the MPOX (**Supplementary Table S1**). Furthermore, for neurological manifestations gene sets, 6650, 2829, 6842, and 3956 genes were associated with headache (**Supplementary Table S2**), myalgia (**Supplementary Table S3**), fatigue (**Supplementary Table S4**), and photophobia (**Supplementary Table S5**), respectively. To determine shared genes between MPOX-associated and neurological manifestations-related genes, we inserted all genes in an excel file that concluded 10 genes (Hub genes) including CD55 molecule (*CD55*), C-X-C motif chemokine ligand 1 (*CXCL1*), nuclear factor kappa B (*NFKB1*), subunit 1, CXCL8, CD4 molecule (*CD4*), IL-6, MX dynamin-like GTPase 1 (*MX1*), complement factor H (*CFH*), killer cell lectin-like receptor K1 (*KLRK1*), CD46 molecule (*CD46*) were shared among totally 20309 genes in five gene sets (**Figure 1A, B** and **Table 1**). Network analyzer using Cytoscape revealed main network features of the reconstructed genetic network as 27 edges, average number of neighbors: 5.400, network diameter: 2, clustering coefficient: 0.779, network heterogeneity: 0.333, and network centralization: 0.500 (**Figure 1B**). In this network, genes such as *IL6*, *CD4*, *CXCL8*, *MX1* and *NFKB1* had the highest degree besides *IL6*, *CD4*, *CXCL8*, *CFH*, *MX1* and *NFKB1* had greatest betweenness centrality (**Table 1**). Based on gene-gene interaction data in STRING, we also predicted five novel genes such as complement factor H related 3 (*CFHR3*), complement C5a receptor 1 (*C5AR1*), complement C3a receptor 1 (*C3AR1*), interferon alpha 2 (*IFNA2*), and C-X-C motif chemokine ligand 3 (*CXCL3*) for MPOX-neurological manifestations associated genes (**Figure 1C**). According to the literature and primary experimental studies, some of these human hub genes (i.e., *CFH*, *NFKB1*, *CXCL8*) interact with MPXV-specific genes (**Supplementary Table S6**). The *MOPICE* (monkeypox virus inhibitor of complement enzymes) gene, which is mainly present in the Congo Basin clade (clade I) of MPXV, plays an important role in modulating the host complement system. The *MOPICE* protein binds directly to complement components C3b and C4b, thereby inhibiting activation through both classical and alternative complement pathways (Liszewski et al., 2006; Estep et al., 2011; Hudson et al., 2012). *A46R*, expressed by both clade I and clade II (West African) strains, inhibits TLR/NF-kB signaling by targeting the NFKB1 pathway (Chen et al., 2005). *C23L*, which primarily present in clade I strains, is a homolog of host chemokines such as CXCL8, and may modulate host immune responses via chemokine mimicry (Reading et al., 2003).

**Table 1:** Network parameters for MPOX and neurological manifestations shared genes.

Index	Gene name	Gene full name	Degree	Betweenness centrality
1	IL6	Interleukin 6	9	0.21527777777
2	CD4	CD4 molecule	8	0.15277777777
3	CXCL8	C-X-C motif chemokine ligand 8	7	0.08564814814
4	MX1	MX dynamin like GTPase 1	5	0.00694444444
5	NFKB1	Nuclear factor kappa B subunit 1	5	0.00694444444
6	CFH	Complement factor H	4	0.01851851851
7	CD55	CD55 molecule	4	0.00694444444
8	CD46	CD46 molecule	4	0.00694444444
9	CXCL1	C-X-C motif chemokine ligand 1	4	0.0
10	KLRK1	Killer cell lectin like receptor K1	4	0.0

### 3.2 Result of gene ontology and pathway analysis for MPOX and its common neurological manifestations

**Figure 2:**
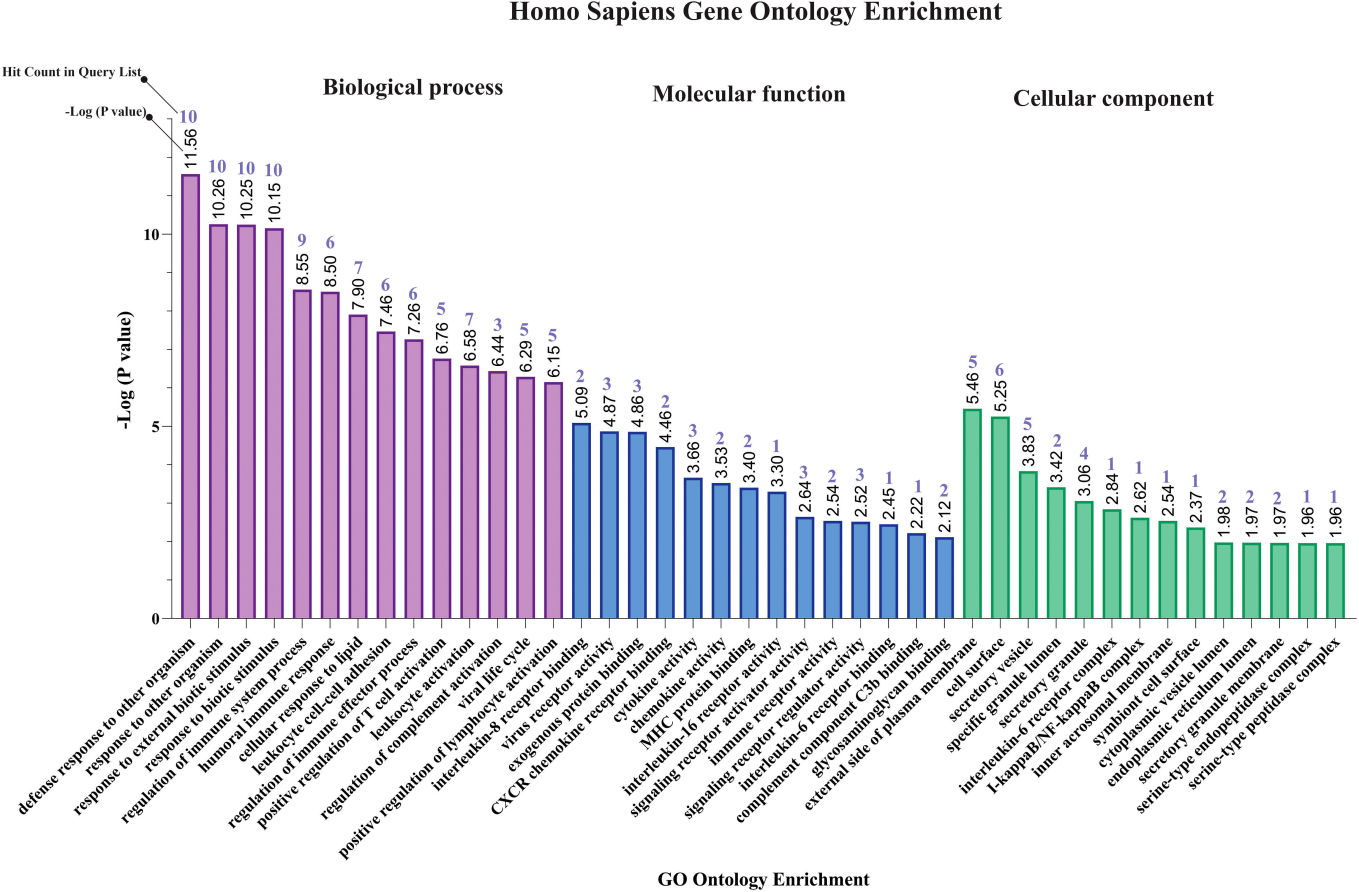
Gene ontology enrichment of shared genes between MPOX and its neurological manifestations. From left to right purple, blue, and green bars represent biological process, molecular function, and cellular component respectively.

The results of gene ontology analysis indicated that response to other organism (GO:0051707), response to external biotic stimulus (GO:0043207), regulation of immune system process (GO:0002682), humoral immune response (GO:0006959), cellular response to lipid (GO:0071396), leukocyte cell-cell adhesion (GO:0007159), positive regulation of T cell activation (GO:0050870), regulation of complement activation (GO:0030449), viral life cycle (GO:0019058), and positive regulation of lymphocyte activation (GO:0051251) were primary affected biological processes for MPOX-neurological associated genes (**Figure 2**). The most enriched molecular functions for our target shared genes included interleukin-8 receptor binding (GO:0005153), virus receptor activity (GO:0001618), exogenous protein binding (GO:0140272), CXCR chemokine receptor binding (GO:0045236), cytokine activity (GO:0005125), chemokine activity (GO:0008009), MHC protein binding (GO:0042287), interleukin 16 receptor activity (GO:0042012), signaling receptor activator activity (GO:0030546), immune receptor activity (GO:0140375), signaling receptor regulator activity (GO:0030545), interleukin 6 receptor binding (GO:0005138), complement component C3b binding (GO:0001851), and glycosaminoglycan binding (GO:0005539). Furthermore, the significantly enriched cellular component in MPXV and its neurological manifestations were primarily associated with external side of plasma membrane (GO:0009897), secretary vesicles (GO:0099503), interleukin 6 receptor complex (GO:0005896), I-kappaB/NF-kappaB complex (GO:0033256), inner acrosomal membrane (GO:0002079) and serine-endopeptidase complex (GO:1905286) (**Figure 2**).

**Figure 3:**
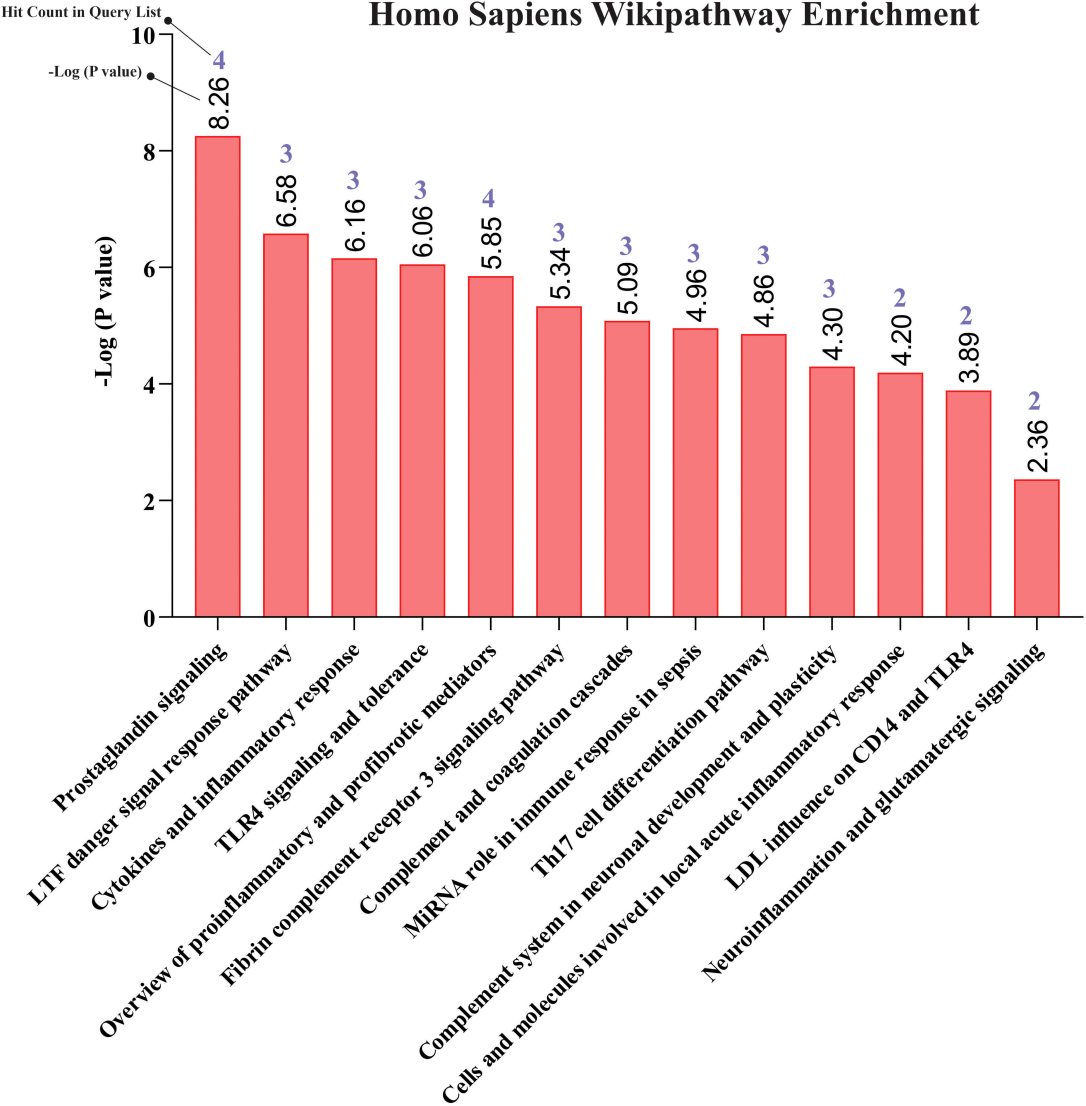
Biological pathway enrichment results for MPOX and its common neurological manifestations.

**Figure 4:**
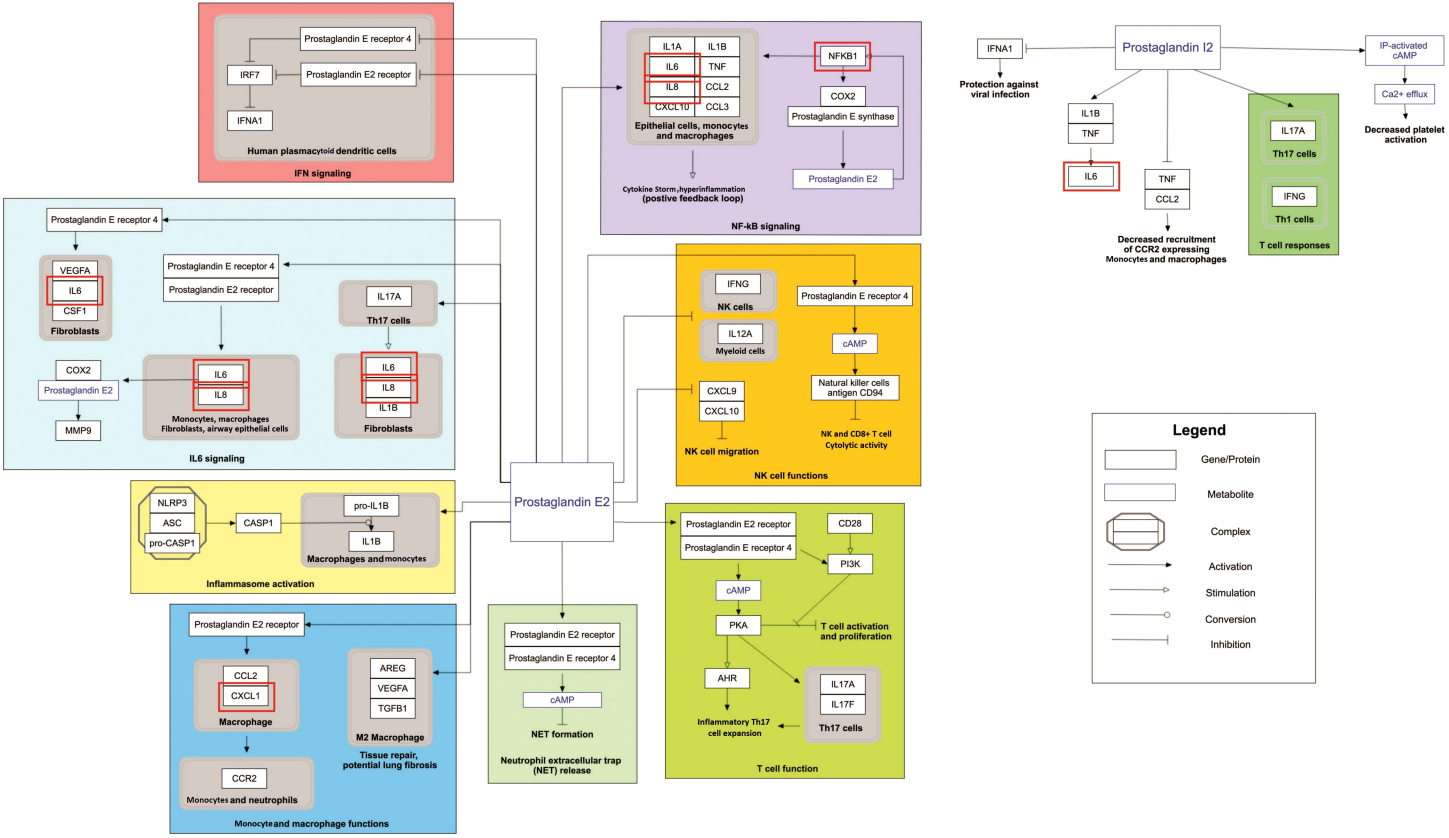
Prostaglandin signaling as a significantly enriched pathway for MPOX and its neurological manifestations. Genes associated with both MPOX and its common neurological manifestations are shown in red boxes. WikiPathways database.

**Figure 5:**
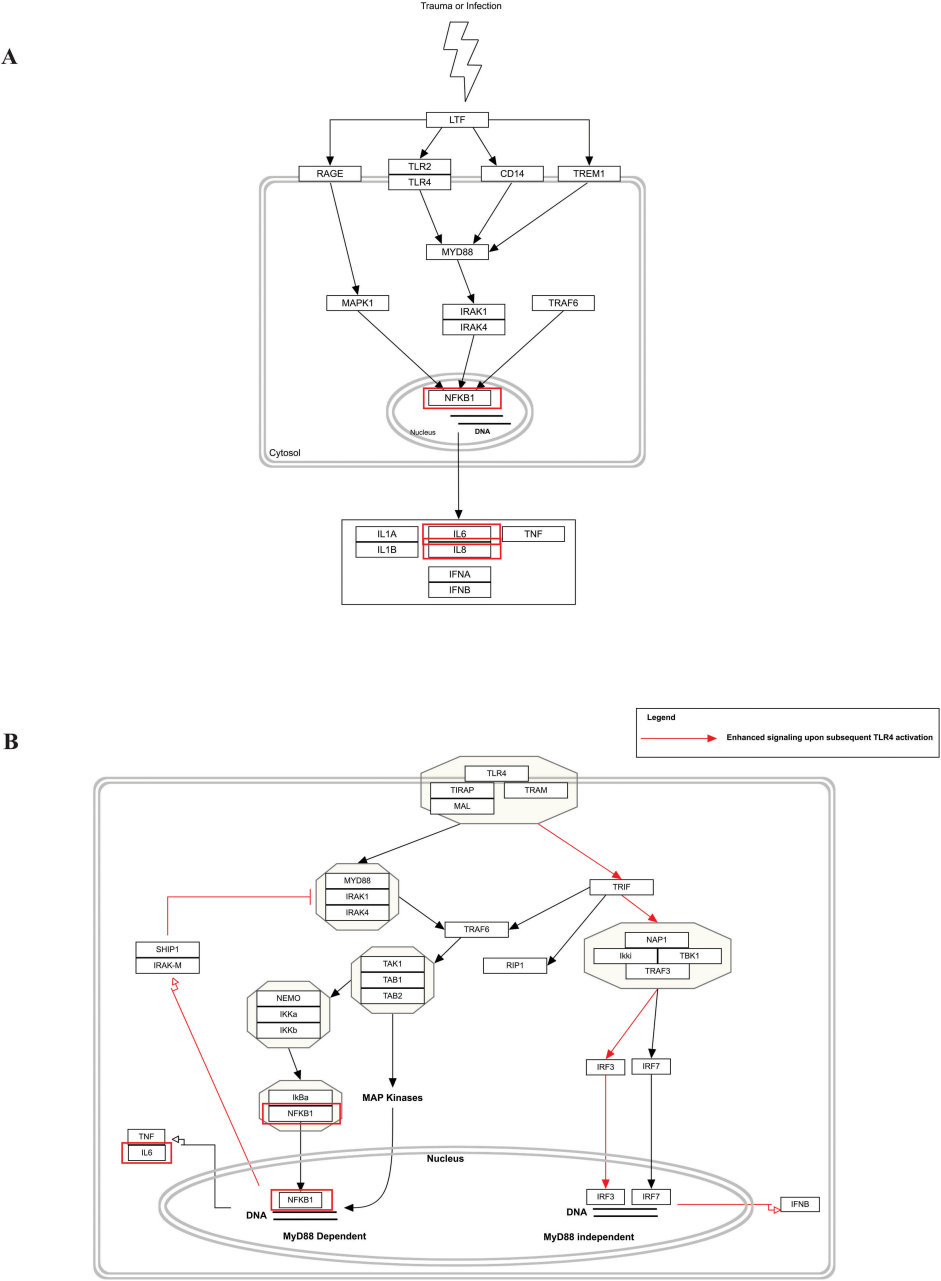
Biological pathway enrichment results for MPOX and its common neurological manifestations. **(A)** LTF danger signal response pathway and **(B)** TLR4 signaling and tolerance. Genes associated to both MPOX and its common neurological manifestations are shown in red boxes. WikiPathways database.

**Figure 6:**
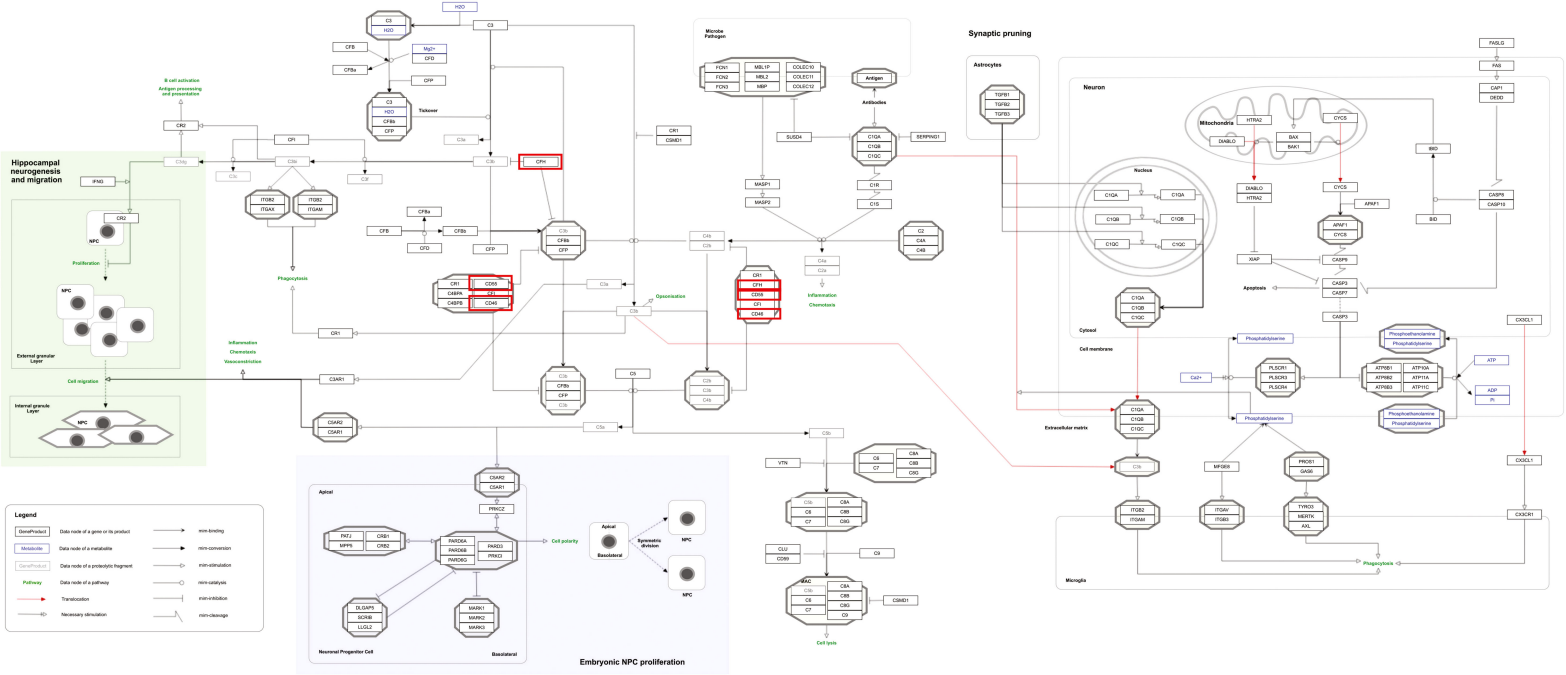
Complement system in neuronal development and plasticity pathway as a significantly enriched pathway for MPOX and its neurological manifestations. Genes associated with both MPOX and its common neurological manifestations are shown in red boxes. WikiPathways database.

**Figure 7:**
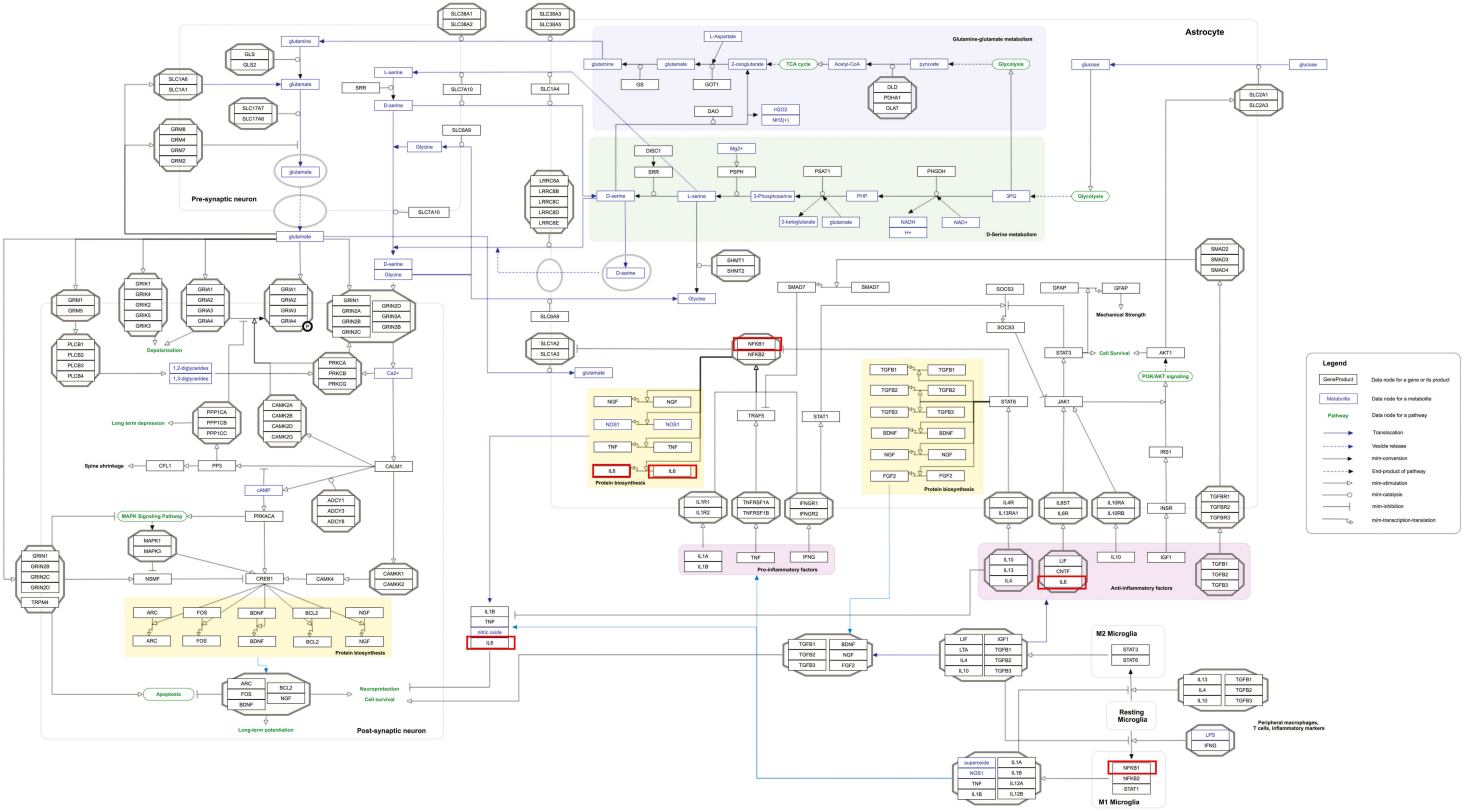
Neuroinflammation and glutamatergic signaling are outstanding pathways for MPOX and its neurological manifestations. Genes associated with both MPOX and its common neurological manifestations are shown in red boxes. WikiPathways database.

Our pathway enrichment analysis also predicted various inflammatory signaling pathways such as prostaglandin signaling (M42532), Lactoferrin (LTF) danger signal response pathway (M39701), cytokines and inflammatory response (M39711), TLR4 signaling and tolerance (M39561), overview of proinflammatory and profibrotic mediators (M42533), fibrin complement receptor 3 signaling pathway (MM15897), complement and coagulation cascades (M39649), TH17 cell differentiation pathway (M45541), complement system in neuronal development and plasticity (M42535), cells and molecules involved in local acute inflammatory response (M39733), LDL influence on CD14 and TLR4 (M45556), and neuroinflammation and glutamatergic signaling (M42572) for MPOX-neurological manifestations associated genes (**Figure 3**). It was revealed that prostaglandin signaling pathways were matched with MPOX-neurological manifestations associated genes, which were enriched with four proteins IL-6, NFKB1, CXCL1, and CXCL8 (IL-8; **Figure 4**). Interestingly, two more major enriched pathways were LTF danger signal response pathway and TLR4 signaling and tolerance, which were predicted based on three MPOX-neurological manifestations associated proteins IL6, NFKB1, and CXCL8 (IL-8; **Figure 5A, B**). It also resulted that the complement system in neuronal development and plasticity pathway was enriched according to three important hub genes including CFH, CD46, and CD55 (**Figure 6**). Two common genes between MPOX and its neurological complications, such as IL-6 and NFKB1, also predict another crucial signaling pathway, namely neuroinflammation and glutamatergic signaling (**Figure 7**).

### 3.3 Cell-type specific enrichment analysis

**Figure 8:**
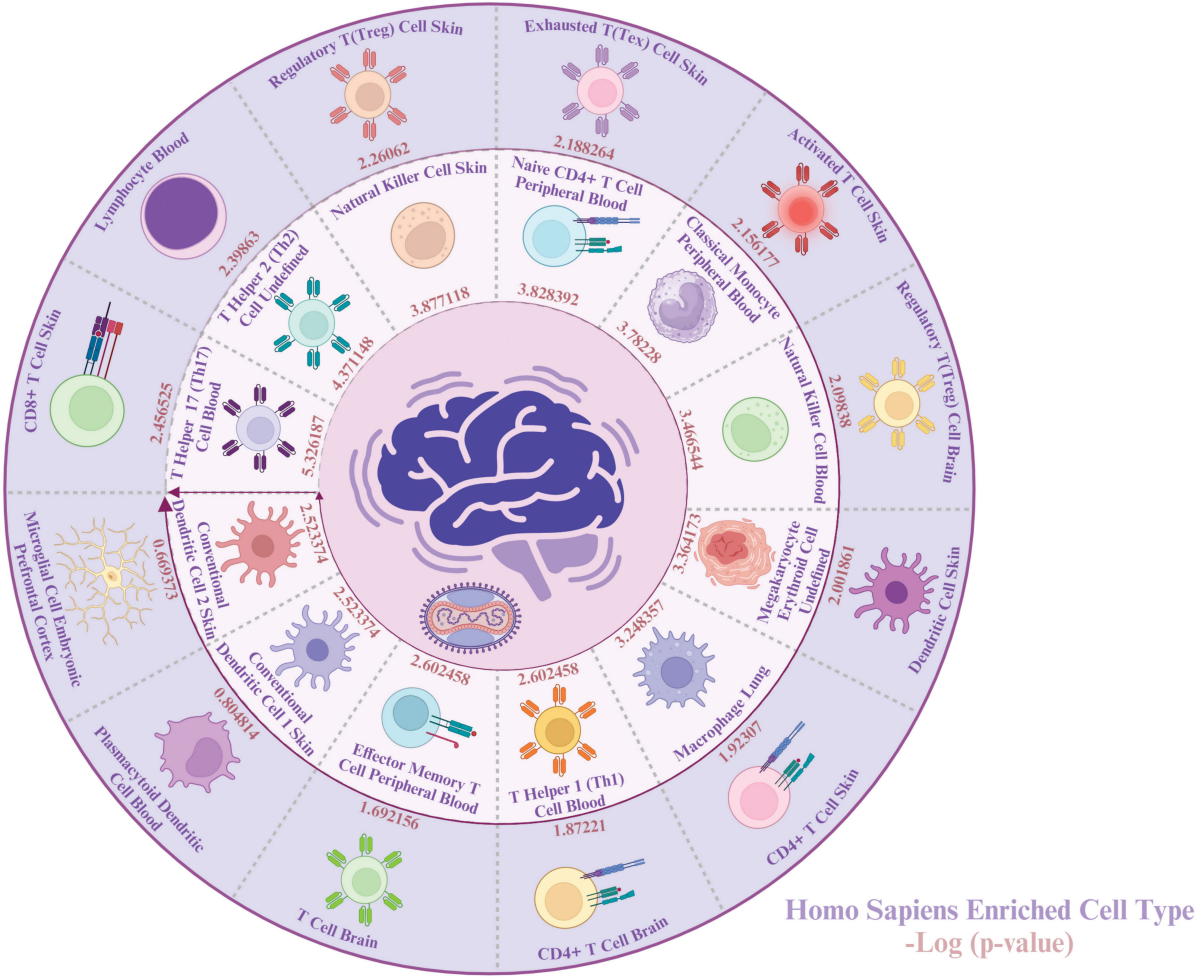
Cell-specific enrichment results for MPOX and its common neurological manifestations. Created with BioRender.com.

To gain a better understanding of the types of cells impacted by both MPOX and neurological manifestations, we conducted a cell-type-specific analysis of their common genes using Enrichr. The enrichment results revealed that MPOX and its neurological manifestations predominantly impact immune cells [T helper 17 (Th17) cell, T helper 2 (Th2), natural killer cell, naive CD4^+^ T cell, classical monocyte, natural killer cell, macrophage, T helper 1 (Th1) cell, effector memory T cell, conventional dendritic cell 1 (cDC1), conventional dendritic cell 2 (cDC2), CD8^+^ T cell lymphocyte], megakaryocyte erythroid cell, and central nervous system (CNS)-resident cells, microglia, (**Figure 8**).

### 3.4 Predicted microRNAs and repurposed drugs

**Figure 9:**
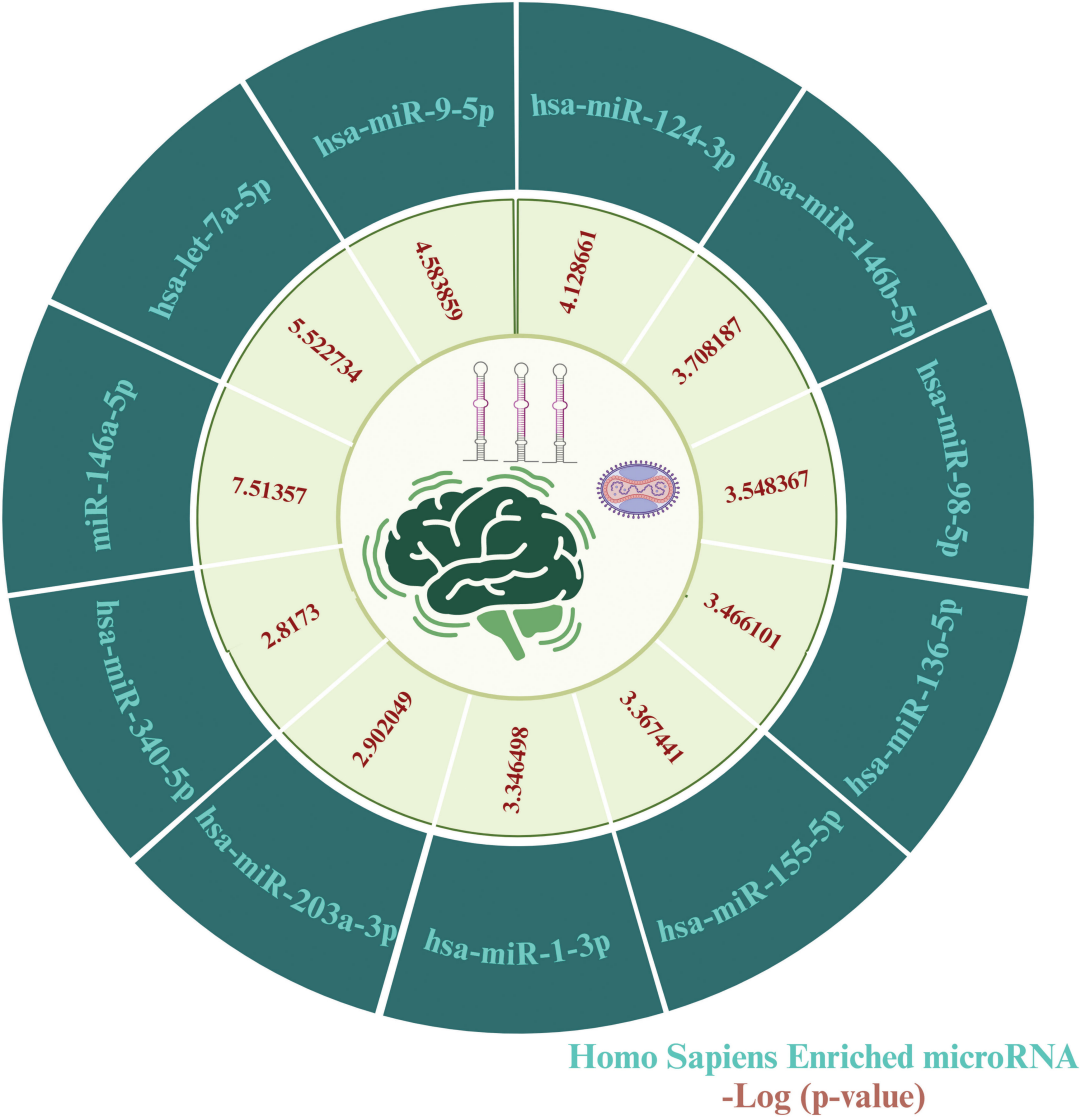
MicroRNA predicting enrichment results for MPOX and its common neurological manifestations. The values for the inner ring are the -log (P value) of enrichment for each miRNA which represents the statistical significance of association with MPOX-neurological manifestation genes. Created with BioRender.com.

To identify key microRNAs linked with MPOX and its prevalent neurological manifestations, mirTarbase was used. We identified 11 important microRNAs including hsa-miR-146a-5p (*IL6, CFH, NFKB1, CXCL8*), hsa-let-7a-5p (*IL6*, *CD55*, *NFKB1*, *CXCL8*), hsa-miR-9-5p (*IL6*, *KLRK1*, *NFKB1*), hsa-miR-124-3p (*IL6*, *CD55*, *CXCL1*, *CXCL8*), hsa-miR-146b-5p (*IL6*, *NFKB1*), hsa-miR-98-5p (*IL6*, *CD46*, *CXCL8*), hsa-miR-136-5p (*IL6*, *CD55*), hsa-miR-155-5p (*IL6*, *NFKB1*, *CXCL8*), hsa-miR-1-3p (*IL6*, *CXCL1*, *CXCL8*), hsa-miR-203a-3p (*IL6*, *CXCL8*), and hsa-miR-340-5p (*CD46*, *CD55*) for MPOX-neurological manifestations shared genes (**Figure 9**).

**Figure 10:**
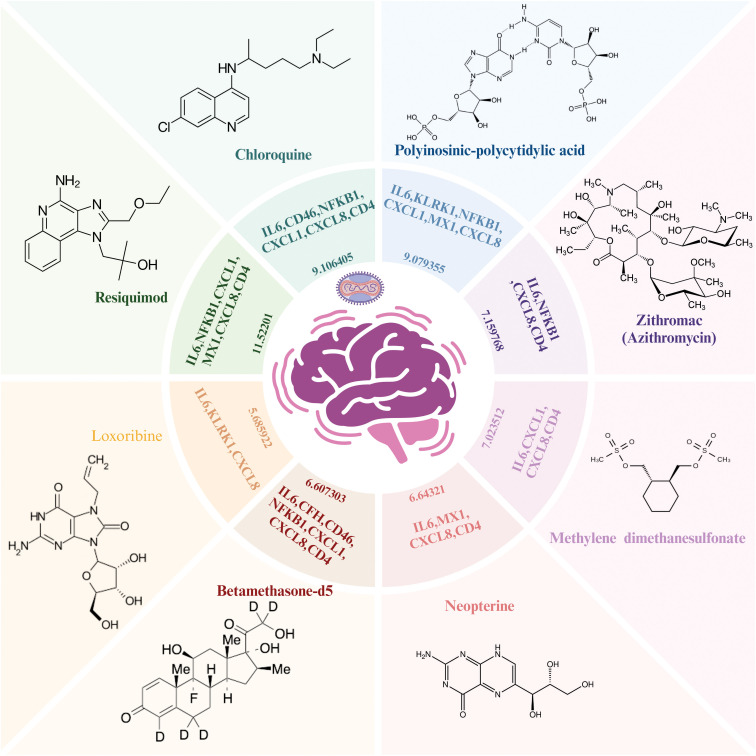
Repurposed drug results for MPOX and its common neurological manifestations. The Enriched drugs are presented based on –Log (p-value) and their primary inputted genes. Created with BioRender.com.

Importantly, based on gene-drug interactions in the Stitch database, we repurposed eight potentially effective drugs including resiquimod (*IL6*, *NFKB1*, *CXCL1*, *MX1*, *CXCL8*, *CD4*), chloroquine (*IL6*, *CD46*, *NFKB1*, *CXCL1*, *CXCL8*, *CD4*), polyinosinic-polycytidylic acid (*IL6*, *KLRK1*, *NFKB1*, *CXCL1*, *MX1*, *CXCL8*), zithromac (*IL6*, *NFKB1*, *CXCL8*, *CD4*), methylene dimethanesulfonate (*IL6*, *NFKB1*, *CXCL1*, *MX1*), neopterin (*IL6*, *MX1*, *CXCL8*, *CD4*), betamethasone-d5 (*IL6*, *CFH*, *CD46*, *NFKB1*, *CXCL1*, *CXCL8*, *CD4*), and loxoribine (*IL6*, *KLRK1*, *CXCL8*) for MPOX-related and its common neurological manifestations-associated genes (**Figure 10**).

## 4 Discussion

Since the World Health Organization’s statement in May 2022, the outbreak of MPOX has expanded across countries, and a wide spectrum of neurological complications have also been documented (Luna et al., 2022; Shafaati and Zandi, 2022; Sepehrinezhad et al., 2023). Furthermore, since August 2024, more than 21,000 new cases were reported in the Democratic Republic of the Congo (Desai et al., 2024; Duarte et al., 2024). This is despite the fact that the potential mechanisms of MPXV neurovirulence and the development of neurological manifestations remain unknown. In the present study, a series of computational methods were used to understand the underlying mechanisms of neurological manifestations of MPOX. Among the key findings, we identified ten shared genes between MPOX and its common neurological manifestations, including inflammatory (*IL6*, *CXCL8*, *NFKB1*), chemotactic (*CXCL, CD4*), antiviral (*MX1, KLRK1*), and complement-regulating genes (*CFH*, *CD55*, *CD46*). These genes were functionally enriched in inflammatory processes, leukocyte adhesion, immunological responses, and regulation of the complement system which demonstrates the multi-faceted host immune engagement during MPXV infection. This gene overlap emphasizes the possibility of a shared inflammatory and complement-mediated pathologic process affecting both systemic and neurological manifestations of MPOX. MPXV infection has been associated with systemic inflammation, cytokine storm, impaired leukocyte function, increased circulatory leukocyte count, aberrant immunological responses, and suppression of the host complement system (Anderson et al., 2003; Liszewski et al., 2006; Hammarlund et al., 2008; Bourquain et al., 2013; Song et al., 2013a; Tree et al., 2015; Xuan et al., 2022; Zandi et al., 2023; Guo et al., 2024).

The network analysis revealed important interactions related to hub genes shared between MPOX and its neurological manifestations (**Figure 1B**, **Table 1**). Topological metrics indicated that IL6, CD4, and CXCL8 were influential nodes with high connectivity (degree) and bridging (betweenness centrality) roles in the protein-protein interaction network. IL6 is a strong pro-inflammatory mediator in MPOX infection that contributes to acute-phase response and cytokine storm (Johnston et al., 2015; Agrati et al., 2023; Meem et al., 2024; Wang et al., 2024). The levels of this cytokine were also correlated with microglia activation and the onset of neuroinflammation (Erta et al., 2012; Bobbo et al., 2019; Kummer et al., 2021). CD4^+^ cells are involved in the recruitment of T-cells, and when this activity is suppressed, it leads to inhibited viral clearance (Swain et al., 2012; Lamens et al., 2025). Patients with MPXV infection exhibited reduced numbers of functional CD4^+^ T-cells in peripheral blood, which correlated with increased disease severity, prolonged fever duration and higher viral loads (Caldrer et al., 2025). CXCL8 is a potent chemotactic factor for neutrophils and other granulocytes that induce local inflammatory responses (Cambier et al., 2023). MX1 and NFKB1 were determined to act as central hubs in our network analysis, despite their moderate connectivity. MX1 inhibits viral mRNA synthesis and disrupts viral particles assembly to exert its antiviral effects (Haller and Kochs, 2011; Verhelst et al., 2012), whereas NFKB1 is the master regulator of inflammatory responses (Karin and Greten, 2005; Bernal-Mizrachi et al., 2006; Liu et al., 2017). This indicates that these two genes provide bottlenecks for antiviral response and neuroinflammation. The identified predicted novel genes generated secondary hubs, indicating the involvement of complement system as a potential mediator of MPXV neuropathology supported by pathway enrichment in **Figure 6**. This network structure demonstrated how MPXV could take advantage of immune hubs in the host to increase neurological damage.

The present study predicted five novel genes for MPOX and its neurological manifestations, including *CFHR3*, *C5AR1*, *C3AR1* (complement-associated genes), *IFNA2*, and *CXCL3*. One of the main arms of innate immunity in viral infections, the complement system mediates numerous important antiviral activities, including the recruitment of neutrophils, the activation of leukocytes, the neutralization of viruses, inflammation, and the formation of membrane attack complexes on infected cells (Mellors et al., 2020; Afzali et al., 2022; Ostrycharz and Hukowska-Szematowicz, 2022). Research has demonstrated that when MPXV infections are present, the host complement system’s activity is significantly inhibited (Estep et al., 2011; Li et al., 2023). The Central African MPXV clade encodes the MOPICE, which inhibits the activity of the complement system by interaction with C4b, C3b, and C5 (Chen et al., 2005; Liszewski et al., 2006). In prairie dogs, deletion of MOPICE from the Congo Basin strain reduced morbidity and mortality of animals 30 days after intranasal inoculation of MPXV (Hudson et al., 2012).

In pathway analysis, prostaglandin signaling was considerably enriched for MPOX and its neurological manifestations. Prostaglandin E2 (PGE_2_) is an arachidonic acid-derived molecule that is produced in response to inflammation or viral infections. Produced PGE2 affects immunological responses in the setting of viral infections (Steer and Corbett, 2003; Pollara et al., 2012; Kim et al., 2017; Sander et al., 2017). As shown in **Figure 4**, PGE_2_ through genes such as *NFKB1*, *IL-6*, and *CXCL8* (*IL-8*) may be responsible for inducing cytokine storm and systemic inflammation after MPXV infection. Also, PGE_2_ may affect the activity of macrophages, monocytes, and neutrophils via *CXCL1* genes in the context of MPXV infection. The LTF danger signal response signaling pathway may be involved in the development of neurological manifestations of MPOX (**Figure 5A**). Following MPXV infections, LTF may activate *NFKB1* via danger signal receptors such as TLR2/4 and CD14 as well as TREM1 and RAGE, leading to the production of IL-6 and IL-8 (**Figure 5A**). Following MPOX infection, activation of TLR4 via the MYD88/IRAK1/IRAK4-TRAF6-TAK1/TAB1/TAB2 signaling pathway may result in nuclear translocation of *NFKB1*, leading to the production of pro-inflammatory cytokines such as IL-6 and TNFα (**Figure 5B**). The TLR family, particularly TLR2 and TLR4, can activate antiviral immune responses by detecting viral capsid proteins (Sartorius et al., 2021; Zhou et al., 2021; Gerber-Tichet et al., 2024). In a silico vaccine design study against MPOX, TLR4 was considered the primary target for docking with the proposed vaccine (Lahimchi et al., 2024). As previously noted, the complement system may get involved during the development of MPOX-related neurological complications. **Figure 6** shows that various MPOX-neurological manifestations related genes, including *CFH*, *CD55*, and *CD46*, can block C3b, CFBb, and CFP genes, which catalyze the formation of C3a and C3b from C3. This suppresses the phagocytosis activity of macrophages and dendritic cells to remove invading infections (inhibit opsonization). A decrease in C3b production also reduces neurogenesis and migration of neural progenitor cells in the hippocampus, as well as the phagocytosis activity of brain microglial cells (**Figure 6**). Furthermore, activation of the *NFKB1* signaling pathway following MPXV infection may create pro-inflammatory cytokines such as IL-6 and TNFα, as well as oxidative indicators such as NOS1 and nitric oxide in brain astrocytes (neuroinflammation). *NFKB1* activation can also have an inhibitory effect on the activity of plasma membrane proteins EAAT2 (SCLC1A2) and EAAT1 (SCLC1A3), which are crucial for astrocytes’ uptake of the excitatory neurotransmitter glutamate from extracellular space. In addition, the predominant phenotype of brain microglia in response to viral derivatives is M1 type, which secretes oxidative markers and produces pro-inflammatory cytokines such as TNFα, IL-1β, IL-1A, and IL-12 through an *NFKB1*-dependent signaling pathway (neuroinflammation). Human astrocytes and, to a lesser extent, microglia have been demonstrated to be susceptible to MPXV infection and replication (Miranzadeh Mahabadi et al., 2024). These results offer functional insight into how MPXV-induced inflammation may impair neurovascular unit and induce glial dysfunction, and ultimately onset of encephalitic symptoms such as headache, and photophobia observed clinically. The enrichment of the complement system in neuronal development and plasticity, supports studies showing that MPXV encodes MOPICE which can inhibit C3b/C4b binding (Chen et al., 2005; Liszewski et al., 2006), so that could inhibit microglial phagocytosis and neurogenesis, giving a credible hypothesis as to how MPXV could change synaptic pruning (elimination of excess synapses) or immune surveillance in the CNS.

The current study also predicted eleven regulatory hub miRNAs, which may play an important role in the development of neurological manifestations following MPOX infection. The generation of pro-inflammatory miR-146a-5p was activated upon activation of *NFKB1* in neural cells, which played essential roles in the course of neurological disorders in the context of viral infections (Taganov et al., 2006; Hill et al., 2015; Lukiw and Pogue, 2020; Pogue and Lukiw, 2021). There is growing evidence that the tumor suppressor and pro-inflammatory microRNA let-7 has a role in antiviral responses and virus replication (Qiu et al., 2017; Zhou et al., 2017; Masyeni et al., 2018; Letafati et al., 2022). Moreover, the regulatory miR-9 inhibited the replication of Herpes simplex virus 1 in mouse primary neurons (Deng et al., 2024). Also, miR-9-5p inhibited apoptosis of mouse dopaminergic neurons via activating β-catenin-SCRIB *in vitro* and *in vivo* (Xiao et al., 2022). These miRNAs, which may modulate immune and inflammatory pathways, may consider as both diagnostic biomarkers and therapeutic targets for mitigating neurological manifestations associated with MPOX.

The enrichment of Th17 cells in the study aligns with their role in driving neuroinflammation via IL-17 production, which activates dendritic cells (DCs), disrupts the integrity of the blood-brain barrier and induces immune cell recruitment (Kebir et al., 2007; Shichita et al., 2009; Huppert et al., 2010; Setiadi et al., 2019). IL-17 causes neuronal injury through both direct pathway as well as indirect mechanisms involving immune cell recruitment and inflammation (Wang et al., 2009; Siffrin et al., 2010; Aghbash et al., 2021). Upregulation of the Th17 pathway has been reported in rhesus macaques infected with MPXV (Aid et al., 2023). IL-17-ativated DCs and viral infections trigger natural killer (NK) cells to secret pro-inflammatory cytokines (i.e. TNFα and IFNγ) (Milovanovic et al., 2020). NK cells are critical for early MPXV control, and their dysfunction may allow for viral dissemination to the CNS. In MPXV-infected rhesus macaques, an increased number of dysfunctional NK cells have been identified in blood and lymph nodes (Song et al., 2013b). Importantly, microglia were the only CNS-specific cell type found, indicating their prominent role in MPXV-associated neuroinflammation. *In vitro* and ex vivo studies indicate that MPXV can penetrate and replicate within human glial cells, particularly astrocytes and microglia (Miranzadeh Mahabadi et al., 2024; Bauer et al., 2023). In response to viral infections, activated microglia secrete pro-inflammatory cytokines that exacerbate neuronal damage and may cause onset of symptoms like headache and photophobia (Chhatbar and Prinz, 2021; O’Brien et al., 2022; Xu et al., 2024). Moreover, microglia can serve as reservoirs for viral entry into the CNS (Ismail et al., 2024; Miranzadeh Mahabadi et al., 2024). These findings reinforce the ability of MPXV to disrupt immune homeostasis at the neurovascular interface, most likely by dysregulating cytokine signaling and pathways involving complement.

The MPXV infection is primarily self-limited, and there are no approved treatments for it. However, several medicines, such as tecovirimat, cidofovir, and brincidofovir, have strong antiviral activity against MPXV in cell cultures, animals, and humans (Andrei and Snoeck, 2010; Merchlinsky et al., 2019; Harris, 2022; O’Laughlin, 2022; Warner et al., 2022; Imran et al., 2023; Shamim et al., 2023). The eight novel medications predicted in this study may be useful in treating MPOX and related neurological manifestations. Resiquimod is a TLR7 and TLR8 agonist that has antiviral and antitumor properties (Bernstein et al., 2001; Pockros et al., 2007; Lee et al., 2014; Gupta et al., 2020). The beneficial effects of Resiquimod on skin lesions have been proven following viral infections (Meyer et al., 2013). A new research team found that resiquimod enhances immunological response in human neuroblastoma cell cultures via TLR7-NFκB-C-C motif chemokine ligand 2 signaling, providing a novel possible therapy approach for neurotropism due to viruses (Kaizuka et al., 2024). Resiquimod modulates the immune response by changing the expression levels of different genes related to inflammation and immunity. Although some specific studies in respect to *IL6*, *NFKB1*, *CXCL1*, *MX1*, *CXCL8*, and *CD4* might be slight, given the known mechanism of action of Resiquimod and general effects of TLR agonists on immune cells, we can deduce expected changes in the expression of these immune-related genes. Toll-like receptor agonists can activate *NF-Kβ* and increase the expression of pro-inflammatory cytokines such as IL-6 and TNFα (Dockrell and Kinghorn, 2001; Medzhitov, 2001; Kirtland et al., 2020). TLR agonists can also activate immune cells to produce pro-inflammatory chemokines, including CXCL1 and CXCL8 (Medzhitov, 2001). Chloroquine is an antimalarial drug that also has antiviral properties. Chloroquine inhibited the release of viral genetic material into host cells via reducing pH (Al-Bari, 2017; Manuja et al., 2023). Chloroquine also inhibited the production of pro-inflammatory cytokines IL-6, TNFα and IL-1β in activated human monocyte/macrophage cultures (Jang et al., 2006). Moreover, anti-tumor agent polyinosinic-polycytidylic acid, a synthetic double-stranded RNA, mimics the features of TLR3 ligands (like viral particles) and binds to this receptor, initiating immunological responses and causes inflammation (Manetti et al., 1995; Fortier et al., 2004; Forte et al., 2012). Polyinosinic-polycytidylic acid has also shown positive effects on wound healing in humans and mice (Lin et al., 2012). Zithromac (azithromycin) is a synthetic macrolide antibiotic that has shown antiviral activity against Enteroviruses, Coronaviruses, Ebola viruses, Picornavirus, Orthomyxovirus, and Flavivirus (Gielen et al., 2010; Madrid et al., 2015; Retallack et al., 2016; Lee et al., 2017; Kawamura et al., 2018; Zeng et al., 2019; Doan et al., 2020; Khoshnood et al., 2022). Neopterin is an organic molecule formed from guanosine triphosphate and is created by macrophages and monocytes upon activation with interferon-gamma that results in an inflammatory state (Murr et al., 2002; Eisenhut, 2013; Heneberk et al., 2023). Neopterin levels in cerebrospinal fluid increased after viral infections of the CNS (Fredrikson et al., 1987; Fuchs et al., 1989; Bociąga-Jasik et al., 2011; Yilmaz et al., 2013; Miyaue et al., 2022). Loxoribine acts as a TLR7 agonist, initiating antiviral responses through the TLR7-MyD88-P50/P65-NFκB signaling pathway (Heil et al., 2003; Dzopalic et al., 2010). The antiviral activity of loxoribine has been reported in various viral infections (Heil et al., 2003; Petro, 2005; Stewart et al., 2012; Aparicio et al., 2014; Enosi Tuipulotu et al., 2018; Girkin et al., 2022; Kayesh et al., 2023). While these candidates require experimental validation, our results provide a basis for rapid therapeutic development for MPOX-associated neuroinflammation.

## 5 Conclusion

In conclusion, the hub genes between MPOX and its main neurological manifestations (i.e. headache, myalgia, fatigue, and photophobia) included complement system, inflammatory, chemotactic receptor, and antiviral-related genes. Also, the predicted novel genes such as CFHR3, C5AR1, C3AR1, IFNA2, and CXCL3 confirmed the importance of the complement system and inflammatory pathways in the development of neurological manifestations of MPOX. This study suggests that immune cells and glial cells, particularly cortical microglia cells, play an essential role in the development of neurological complications associated with MPXV infection. Moreover, our findings suggest that prostaglandin signaling, LTF danger signal response pathway, TLR4 signaling, complement system in neuronal development and plasticity, and neuroinflammation and glutamatergic signaling all play crucial roles in the pathogenesis of MPXV infection in the CNS. The study sheds new light on the pathophysiology of MPOX and implies that targeting the complement system and immunotherapy could be a promising treatment strategy for managing the neurological consequences of MPOX disease. The lack of experimental confirmation of computational conclusions is seen as the study’s key limitation, necessitating additional research in future basic and clinical studies. Furthermore, our work predicted some regulatory microRNAs, which could serve as biomarkers and therapeutic targets for MPOX and its neurological manifestations. We also recommended several drugs that potentially have antiviral properties against MPXV and are useful in treating the primary neurological consequences of MPOX. However, these findings will require in-depth validation in future investigations.

## Data Availability

The original contributions presented in the study are included in the article/**Supplementary Material**. Further inquiries can be directed to the corresponding author.
