# Supplementary material for: A bioinformatic analysis to systematically unveil shared pathways and molecular mechanisms underlying monkeypox and its predominant neurological manifestations
Source: Front Cell Infect Microbiol. 2025 Jul 2;15:1506687. doi: 10.3389/fcimb.2025.1506687 (PMC12263605; doi:10.3389/fcimb.2025.1506687)
Supplement: Supplementary file 1 [file DataSheet1.pdf]

Supplementary Results

A bioinformatic analysis to systematically unveil shared pathways and molecular mechanisms underlying monkeypox and its predominant neurological manifestations

Amir Hossein Barjasteh[1], Hanieh Latifi[1], Ali Sepehrinezhad[2, 3*]

[1]Student Research Committee, Mashhad University of Medical Sciences, Mashhad, Iran

[2]Neuroscience Research Center, Mashhad University of Medical Sciences, Mashhad, Iran

[3]Department of Neuroscience, Faculty of Medicine, Mashhad University of Medical Sciences, Mashhad, Iran

* Correspondence: Department of Neuroscience, Faculty of Medicine, Mashhad University of Medical Sciences, Pardis Campus, Azadi Square, Mashhad, Iran; sepehrinezhada@mums.ac.ir; Tel.: +989227608967.

Supplementary Table S1. Genes associated with monkeypox (MPOX) that are extracted from GeneCards.

Index	Gene Symbol	Gene Full Name
1.	CD4	CD4 Molecule
2.	CYB5R3	Cytochrome B5 Reductase 3
3.	FAR1	Fatty Acyl-CoA Reductase 1
4.	VPS54	VPS54 Subunit Of GARP Complex
5.	GPR182	G Protein-Coupled Receptor 182
6.	CHRFAM7A	CHRNA7 (Exons 5-10) And FAM7A (Exons A-E) Fusion
7.	CLEC17A	C-Type Lectin Domain Containing 17A
8.	DEFB118	Defensin Beta 118
9.	DCANP1	Dendritic Cell Associated Nuclear Protein 1
10.	DEFB127	Defensin Beta 127
11.	ZNF613	Zinc Finger Protein 613
12.	OR10G6	Olfactory Receptor Family 10 Subfamily G Member 6
13.	APOBEC3F	Apolipoprotein B MRNA Editing Enzyme Catalytic Subunit 3F
14.	EXT1	Exostosin Glycosyltransferase 1
15.	TMED10	Transmembrane P24 Trafficking Protein 10
16.	TM9SF2	Transmembrane 9 Superfamily Member 2
17.	NFKB1	Nuclear Factor Kappa B Subunit 1
18.	IL6	Interleukin 6
19.	PPIA	Peptidylprolyl Isomerase A

20.	CXCL8	C-X-C Motif Chemokine Ligand 8
21.	CXCL1	C-X-C Motif Chemokine Ligand 1
22.	TRIM5	Tripartite Motif Containing 5
23.	CD55	CD55 Molecule (Cromer Blood Group)
24.	CD46	CD46 Molecule
25.	CFH	Complement Factor H
26.	HSF1	Heat Shock Transcription Factor 1
27.	LIG4	DNA Ligase 4
28.	CR2	Complement C3d Receptor 2
29.	CR1	Complement C3b/C4b Receptor 1 (Knops Blood Group)
30.	KLRK1	Killer Cell Lectin Like Receptor K1
31.	MX1	MX Dynamin Like GTPase 1
32.	ADPRH	ADP-Ribosylarginine Hydrolase

Supplementary Table S2. Genes associated with headache that are extracted from GeneCards.

Index	Gene Symbol	Gene Full Name
1.	CACNA1A	Calcium Voltage-Gated Channel Subunit Alpha1 A
2.	BRCA2	BRCA2 DNA Repair Associated
3.	TNF	Tumor Necrosis Factor
4.	CLCN1	Chloride Voltage-Gated Channel 1
5.	NOTCH3	Notch Receptor 3
6.	EDNRA	Endothelin Receptor Type A
7.	NPC1	NPC Intracellular Cholesterol Transporter 1
8.	FKRP	Fukutin Related Protein
9.	ESR1	Estrogen Receptor 1
10.	ALPK1	Alpha Kinase 1
11.	MT-ND1	Mitochondrially Encoded NADH:Ubiquinone Oxidoreductase Core Subunit 1
12.	ATP1A2	ATPase Na+/K+ Transporting Subunit Alpha 2
13.	PPOX	Protoporphyrinogen Oxidase
14.	CALCA	Calcitonin Related Polypeptide Alpha
15.	SCN1A	Sodium Voltage-Gated Channel Alpha Subunit 1
16.	CAPN3	Calpain 3
17.	TAB2	TGF-Beta Activated Kinase 1 (MAP3K7) Binding Protein 2
18.	POLG	DNA Polymerase Gamma, Catalytic Subunit
19.	TP53	Tumor Protein P53
20.	HCRTR2	Hypocretin Receptor 2
21.	IL6	Interleukin 6
22.	IL10	Interleukin 10

23.	ALB	Albumin
24.	ACE	Angiotensin I Converting Enzyme
25.	F2	Coagulation Factor II, Thrombin
26.	PIK3CA	Phosphatidylinositol-4,5-Bisphosphate 3-Kinase Catalytic Subunit Alpha
27.	MSX2	Msh Homeobox 2
28.	PTGS2	Prostaglandin-Endoperoxide Synthase 2
29.	PRL	Prolactin
30.	VHL	Von Hippel-Lindau Tumor Suppressor
31.	NF1	Neurofibromin 1
32.	BDNF	Brain Derived Neurotrophic Factor
33.	MAOA	Monoamine Oxidase A
34.	CXCL8	C-X-C Motif Chemokine Ligand 8
35.	SDHB	Succinate Dehydrogenase Complex Iron Sulfur Subunit B
36.	HTR3A	5-Hydroxytryptamine Receptor 3A
37.	TGFB1	Transforming Growth Factor Beta 1
38.	SLC6A4	Solute Carrier Family 6 Member 4
39.	COMT	Catechol-O-Methyltransferase
40.	KCNK18	Potassium Two Pore Domain Channel Subfamily K Member 18
41.	HLA-DQB1	Major Histocompatibility Complex, Class II, DQ Beta 1
42.	IFNG	Interferon Gamma
43.	NOS2	Nitric Oxide Synthase 2
44.	HTR2A	5-Hydroxytryptamine Receptor 2A
45.	PDGFRB	Platelet Derived Growth Factor Receptor Beta
46.	NOS3	Nitric Oxide Synthase 3
47.	NLRP3	NLR Family Pyrin Domain Containing 3
48.	SCN2A	Sodium Voltage-Gated Channel Alpha Subunit 2
49.	NPY	Neuropeptide Y
50.	IL2	Interleukin 2
51.	TTR	Transthyretin
52.	BRAF	B-Raf Proto-Oncogene, Serine/Threonine Kinase
53.	APP	Amyloid Beta Precursor Protein
54.	PDGFB	Platelet Derived Growth Factor Subunit B
55.	TLR4	Toll Like Receptor 4
56.	INS	Insulin
57.	IL1B	Interleukin 1 Beta
58.	SLC2A1	Solute Carrier Family 2 Member 1
59.	MTHFR	Methylenetetrahydrofolate Reductase
60.	COL4A1	Collagen Type IV Alpha 1 Chain
61.	ADH4	Alcohol Dehydrogenase 4 (Class II), Pi Polypeptide
62.	MT-CO1	Mitochondrially Encoded Cytochrome C Oxidase I
63.	MT-ND6	Mitochondrially Encoded NADH:Ubiquinone Oxidoreductase Core Subunit 6

64.	CLOCK	Clock Circadian Regulator
65.	PTEN	Phosphatase And Tensin Homolog
66.	MT-CYB	Mitochondrially Encoded Cytochrome B
67.	PDCD10	Programmed Cell Death 10
68.	CYP1A2	Cytochrome P450 Family 1 Subfamily A Member 2
69.	CCM2	CCM2 Scaffold Protein
70.	HTR1A	5-Hydroxytryptamine Receptor 1A
71.	SLC1A3	Solute Carrier Family 1 Member 3
72.	MEFV	MEFV Innate Immunity Regulator, Pyrin
73.	SMARCB1	SWI/SNF Related, Matrix Associated, Actin Dependent Regulator Of Chromatin, Subfamily B, Member 1
74.	KRIT1	KRIT1 Ankyrin Repeat Containing
75.	AKT1	AKT Serine/Threonine Kinase 1
76.	HCRT	Hypocretin Neuropeptide Precursor
77.	ABCB1	ATP Binding Cassette Subfamily B Member 1
78.	SDHA	Succinate Dehydrogenase Complex Flavoprotein Subunit A
79.	HLA-B	Major Histocompatibility Complex, Class I, B
80.	CRP	C-Reactive Protein
81.	CCK	Cholecystokinin
82.	FBN1	Fibrillin 1
83.	HBB	Hemoglobin Subunit Beta
84.	CCND1	Cyclin D1
85.	HLA-DRB1	Major Histocompatibility Complex, Class II, DR Beta 1
86.	CTNNB1	Catenin Beta 1
87.	ICAM1	Intercellular Adhesion Molecule 1
88.	F5	Coagulation Factor V
89.	GPT	Glutamic--Pyruvic Transaminase
90.	G6PD	Glucose-6-Phosphate Dehydrogenase
91.	APOE	Apolipoprotein E
92.	CBS	Cystathionine Beta-Synthase
93.	KRAS	KRAS Proto-Oncogene, GTPase
94.	JAK2	Janus Kinase 2
95.	TAC1	Tachykinin Precursor 1
96.	NF2	NF2, Moesin-Ezrin-Radixin Like (MERLIN) Tumor Suppressor
97.	SUFU	SUFU Negative Regulator Of Hedgehog Signaling
98.	EGFR	Epidermal Growth Factor Receptor
99.	IDH1	Isocitrate Dehydrogenase (NADP(+)) 1
100.	PPARA	Peroxisome Proliferator Activated Receptor Alpha
101.	IL4	Interleukin 4
102.	IFNB1	Interferon Beta 1
103.	HTR1B	5-Hydroxytryptamine Receptor 1B
104.	EPHB4	EPH Receptor B4

105.	MVK	Mevalonate Kinase
106.	MT-CO2	Mitochondrially Encoded Cytochrome C Oxidase II
107.	VIP	Vasoactive Intestinal Peptide
108.	RET	Ret Proto-Oncogene
109.	MT-ND5	Mitochondrially Encoded NADH:Ubiquinone Oxidoreductase Core Subunit 5
110.	NRAS	NRAS Proto-Oncogene, GTPase
111.	SDHD	Succinate Dehydrogenase Complex Subunit D
112.	EPAS1	Endothelial PAS Domain Protein 1
113.	CCL2	C-C Motif Chemokine Ligand 2
114.	MT-ND4	Mitochondrially Encoded NADH:Ubiquinone Oxidoreductase Core Subunit 4
115.	S100B	S100 Calcium Binding Protein B
116.	APC	APC Regulator Of WNT Signaling Pathway
117.	KIF1B	Kinesin Family Member 1B
118.	CLCN2	Chloride Voltage-Gated Channel 2
119.	ADCYAP1	Adenylate Cyclase Activating Polypeptide 1
120.	GRIN2A	Glutamate Ionotropic Receptor NMDA Type Subunit 2A
121.	SST	Somatostatin
122.	TNFRSF1A	TNF Receptor Superfamily Member 1A
123.	KNG1	Kininogen 1
124.	MEN1	Menin 1
125.	MT-ATP6	Mitochondrially Encoded ATP Synthase Membrane Subunit 6
126.	AGT	Angiotensinogen
127.	ADA2	Adenosine Deaminase 2
128.	RASA1	RAS P21 Protein Activator 1
129.	NGF	Nerve Growth Factor
130.	SLC6A3	Solute Carrier Family 6 Member 3
131.	HTRA1	HtrA Serine Peptidase 1
132.	ANK3	Ankyrin 3
133.	PNOC	Prepronociceptin
134.	MT-CO3	Mitochondrially Encoded Cytochrome C Oxidase III
135.	DRD2	Dopamine Receptor D2
136.	POLGARF	POLG Alternative Reading Frame
137.	NOS1	Nitric Oxide Synthase 1
138.	TWNK	Twinkle MtDNA Helicase
139.	CYP2D6	Cytochrome P450 Family 2 Subfamily D Member 6
140.	TBK1	TANK Binding Kinase 1
141.	SMARCAL1	SWI/SNF Related, Matrix Associated, Actin Dependent Regulator Of Chromatin, Subfamily A Like 1
142.	KIT	KIT Proto-Oncogene, Receptor Tyrosine Kinase
143.	POMC	Proopiomelanocortin
144.	ATM	ATM Serine/Threonine Kinase
145.	STIM1	Stromal Interaction Molecule 1

146.	ENG	Endoglin
147.	IRF3	Interferon Regulatory Factor 3
148.	HRAS	HRas Proto-Oncogene, GTPase
149.	IDH2	Isocitrate Dehydrogenase (NADP(+)) 2
150.	PRDM16	PR/SET Domain 16
151.	GFAP	Glial Fibrillary Acidic Protein
152.	TLR3	Toll Like Receptor 3
153.	CYP3A4	Cytochrome P450 Family 3 Subfamily A Member 4
154.	PSEN1	Presenilin 1
155.	DRD4	Dopamine Receptor D4
156.	CACNA1F	Calcium Voltage-Gated Channel Subunit Alpha1 F
157.	SMO	Smoothened, Frizzled Class Receptor
158.	ATRX	ATRX Chromatin Remodeler
159.	SDHC	Succinate Dehydrogenase Complex Subunit C
160.	PRRT2	Proline Rich Transmembrane Protein 2
161.	FAS	Fas Cell Surface Death Receptor
162.	MYORG	Myogenesis Regulating Glycosidase (Putative)
163.	HTR4	5-Hydroxytryptamine Receptor 4
164.	FGFR2	Fibroblast Growth Factor Receptor 2
165.	TET2	Tet Methylcytosine Dioxygenase 2
166.	FGFR1	Fibroblast Growth Factor Receptor 1
167.	PDE5A	Phosphodiesterase 5A
168.	KCNA1	Potassium Voltage-Gated Channel Subfamily A Member 1
169.	VWF	Von Willebrand Factor
170.	TREX1	Three Prime Repair Exonuclease 1
171.	MSH2	MutS Homolog 2
172.	RNF213	Ring Finger Protein 213
173.	FLT1	Fms Related Receptor Tyrosine Kinase 1
174.	MAX	MYC Associated Factor X
175.	SCN9A	Sodium Voltage-Gated Channel Alpha Subunit 9
176.	TMEM127	Transmembrane Protein 127
177.	HTR1D	5-Hydroxytryptamine Receptor 1D
178.	AIP	Aryl Hydrocarbon Receptor Interacting Protein
179.	GNB3	G Protein Subunit Beta 3
180.	CD36	CD36 Molecule (CD36 Blood Group)
181.	MYCN	MYCN Proto-Oncogene, BHLH Transcription Factor
182.	CFH	Complement Factor H
183.	F3	Coagulation Factor III, Tissue Factor
184.	OPRM1	Opioid Receptor Mu 1
185.	GCG	Glucagon
186.	CACNA1C	Calcium Voltage-Gated Channel Subunit Alpha1 C

187.	FCGR2A	Fc Gamma Receptor IIa
188.	DNMT3A	DNA Methyltransferase 3 Alpha
189.	TERT	Telomerase Reverse Transcriptase
190.	SOD1	Superoxide Dismutase 1
191.	PTPN22	Protein Tyrosine Phosphatase Non-Receptor Type 22
192.	MAOB	Monoamine Oxidase B
193.	MMP9	Matrix Metallopeptidase 9
194.	ATP1A3	ATPase Na+/K+ Transporting Subunit Alpha 3
195.	DKK1	Dickkopf WNT Signaling Pathway Inhibitor 1
196.	TRAF3	TNF Receptor Associated Factor 3
197.	NLRP12	NLR Family Pyrin Domain Containing 12
198.	CALR	Calreticulin
199.	GSTM1	Glutathione S-Transferase Mu 1
200.	MPL	MPL Proto-Oncogene, Thrombopoietin Receptor
201.	THPO	Thrombopoietin
202.	CSNK1D	Casein Kinase 1 Delta
203.	MYD88	MYD88 Innate Immune Signal Transduction Adaptor
204.	TRPM8	Transient Receptor Potential Cation Channel Subfamily M Member 8
205.	IL1A	Interleukin 1 Alpha
206.	SCN5A	Sodium Voltage-Gated Channel Alpha Subunit 5
207.	CYP2C19	Cytochrome P450 Family 2 Subfamily C Member 19
208.	IFNA1	Interferon Alpha 1
209.	TRPV1	Transient Receptor Potential Cation Channel Subfamily V Member 1
210.	UNC93B1	Unc-93 Homolog B1, TLR Signaling Regulator
211.	SH2B3	SH2B Adaptor Protein 3
212.	PHACTR1	Phosphatase And Actin Regulator 1
213.	HFE	Homeostatic Iron Regulator
214.	CACNA1H	Calcium Voltage-Gated Channel Subunit Alpha1 H
215.	ASTN2	Astrotactin 2
216.	ADM	Adrenomedullin
217.	FH	Fumarate Hydratase
218.	PMS2	PMS1 Homolog 2, Mismatch Repair System Component
219.	IFNGR1	Interferon Gamma Receptor 1
220.	ANK1	Ankyrin 1
221.	LDLR	Low Density Lipoprotein Receptor
222.	PPBP	Pro-Platelet Basic Protein
223.	ASXL1	ASXL Transcriptional Regulator 1
224.	CNR1	Cannabinoid Receptor 1
225.	EPOR	Erythropoietin Receptor
226.	PTCH1	Patched 1
227.	CACNB4	Calcium Voltage-Gated Channel Auxiliary Subunit Beta 4

228.	CSF2	Colony Stimulating Factor 2
229.	SDHAF2	Succinate Dehydrogenase Complex Assembly Factor 2
230.	ACE2	Angiotensin Converting Enzyme 2
231.	TRPV4	Transient Receptor Potential Cation Channel Subfamily V Member 4
232.	CD46	CD46 Molecule
233.	EDN1	Endothelin 1
234.	TACR1	Tachykinin Receptor 1
235.	LRRC56	Leucine Rich Repeat Containing 56
236.	CACNA1B	Calcium Voltage-Gated Channel Subunit Alpha1 B
237.	POLE	DNA Polymerase Epsilon, Catalytic Subunit
238.	MTOR	Mechanistic Target Of Rapamycin Kinase
239.	CD40LG	CD40 Ligand
240.	CACNA1S	Calcium Voltage-Gated Channel Subunit Alpha1 S
241.	FHL5	Four And A Half LIM Domains 5
242.	MOG	Myelin Oligodendrocyte Glycoprotein
243.	SLC20A2	Solute Carrier Family 20 Member 2
244.	CACNA1G	Calcium Voltage-Gated Channel Subunit Alpha1 G
245.	WFS1	Wolframin ER Transmembrane Glycoprotein
246.	FMR1	Fragile X Messenger Ribonucleoprotein 1
247.	REN	Renin
248.	P4HA2	Prolyl 4-Hydroxylase Subunit Alpha 2
249.	MEF2D	Myocyte Enhancer Factor 2D
250.	ZFTA	Zinc Finger Translocation Associated
251.	CREBBP	CREB Binding Protein
252.	PYY	Peptide YY
253.	CFI	Complement Factor I
254.	TICAM1	TIR Domain Containing Adaptor Molecule 1
255.	IAPP	Islet Amyloid Polypeptide
256.	ACHE	Acetylcholinesterase (Yt Blood Group)
257.	CACNA1D	Calcium Voltage-Gated Channel Subunit Alpha1 D
258.	GYPC	Glycophorin C (Gerbich Blood Group)
259.	KCNQ2	Potassium Voltage-Gated Channel Subfamily Q Member 2
260.	RAMP1	Receptor Activity Modifying Protein 1
261.	AGTR1	Angiotensin II Receptor Type 1
262.	SLC4A1	Solute Carrier Family 4 Member 1 (Diego Blood Group)
263.	BCAT2	Branched Chain Amino Acid Transaminase 2
264.	CSF3	Colony Stimulating Factor 3
265.	CDKN1A	Cyclin Dependent Kinase Inhibitor 1A
266.	CD4	CD4 Molecule
267.	PDYN	Prodynorphin
268.	H3-3A	H3.3 Histone A

269.	KCNQ3	Potassium Voltage-Gated Channel Subfamily Q Member 3
270.	TSC2	TSC Complex Subunit 2
271.	TRAF7	TNF Receptor Associated Factor 7
272.	MDH2	Malate Dehydrogenase 2
273.	LRP5	LDL Receptor Related Protein 5
274.	OTC	Ornithine Transcarbamylase
275.	RELA	RELA Proto-Oncogene, NF-KB Subunit
276.	DNAH8	Dynein Axonemal Heavy Chain 8
277.	XPR1	Xenotropic And Polytropic Retrovirus Receptor 1
278.	FAAH	Fatty Acid Amide Hydrolase
279.	CR1	Complement C3b/C4b Receptor 1 (Knops Blood Group)
280.	IL1RN	Interleukin 1 Receptor Antagonist
281.	STOX1	Storkhead Box 1
282.	SMARCE1	SWI/SNF Related, Matrix Associated, Actin Dependent Regulator Of Chromatin, Subfamily E, Member 1
283.	ADAMTS13	ADAM Metallopeptidase With Thrombospondin Type 1 Motif 13
284.	RELN	Reelin
285.	PLAT	Plasminogen Activator, Tissue Type
286.	NDUFS1	NADH:Ubiquinone Oxidoreductase Core Subunit S1
287.	HTR2B	5-Hydroxytryptamine Receptor 2B
288.	GYPA	Glycophorin A (MNS Blood Group)
289.	FANCD2	FA Complementation Group D2
290.	GBA1	Glucosylceramidase Beta 1
291.	EPO	Erythropoietin
292.	SLC25A4	Solute Carrier Family 25 Member 4
293.	TLR5	Toll Like Receptor 5
294.	HTR2C	5-Hydroxytryptamine Receptor 2C
295.	CORIN	Corin, Serine Peptidase
296.	GP1BA	Glycoprotein Ib Platelet Subunit Alpha
297.	OXT	Oxytocin/Neurophysin I Prepropeptide
298.	SLC25A11	Solute Carrier Family 25 Member 11
299.	HSPA4	Heat Shock Protein Family A (Hsp70) Member 4
300.	BAP1	BRCA1 Associated Protein 1
301.	IL2RA	Interleukin 2 Receptor Subunit Alpha
302.	APOH	Apolipoprotein H
303.	DLST	Dihydrolipoamide S-Succinyltransferase
304.	LEP	Leptin
305.	JAM2	Junctional Adhesion Molecule 2
306.	CD8A	CD8 Subunit Alpha
307.	STAT4	Signal Transducer And Activator Of Transcription 4
308.	FOXP3	Forkhead Box P3
309.	EP300	E1A Binding Protein P300

310.	IKBKG	Inhibitor Of Nuclear Factor Kappa B Kinase Regulatory Subunit Gamma
311.	ENO2	Enolase 2
312.	CCR1	C-C Motif Chemokine Receptor 1
313.	NAGS	N-Acetylglutamate Synthase
314.	PTPN11	Protein Tyrosine Phosphatase Non-Receptor Type 11
315.	TH	Tyrosine Hydroxylase
316.	GYPB	Glycophorin B (MNS Blood Group)
317.	SETD2	SET Domain Containing 2, Histone Lysine Methyltransferase
318.	CYB5R3	Cytochrome B5 Reductase 3
319.	ERVW-1	Endogenous Retrovirus Group W Member 1, Envelope
320.	SOST	Sclerostin
321.	TYR	Tyrosinase
322.	GDNF	Glial Cell Derived Neurotrophic Factor
323.	IGF1	Insulin Like Growth Factor 1
324.	ANK2	Ankyrin 2
325.	ARMS2	Age-Related Maculopathy Susceptibility 2
326.	IDO1	Indoleamine 2,3-Dioxygenase 1
327.	COL4A2	Collagen Type IV Alpha 2 Chain
328.	DBH	Dopamine Beta-Hydroxylase
329.	F8	Coagulation Factor VIII
330.	GATA2	GATA Binding Protein 2
331.	CCR5	C-C Motif Chemokine Receptor 5
332.	EPM2A	EPM2A Glucan Phosphatase, Laforin
333.	IFIH1	Interferon Induced With Helicase C Domain 1
334.	DEPDC5	DEP Domain Containing 5, GATOR1 Subcomplex Subunit
335.	GRIN2B	Glutamate Ionotropic Receptor NMDA Type Subunit 2B
336.	PKD1	Polycystin 1, Transient Receptor Potential Channel Interacting
337.	CISH	Cytokine Inducible SH2 Containing Protein
338.	ACKR1	Atypical Chemokine Receptor 1 (Duffy Blood Group)
339.	PAX2	Paired Box 2
340.	MN1	MN1 Proto-Oncogene, Transcriptional Regulator
341.	WRN	WRN RecQ Like Helicase
342.	IL17A	Interleukin 17A
343.	SYP	Synaptophysin
344.	LGI1	Leucine Rich Glioma Inactivated 1
345.	CDH23	Cadherin Related 23
346.	IL13	Interleukin 13
347.	ALX4	ALX Homeobox 4
348.	FOS	Fos Proto-Oncogene, AP-1 Transcription Factor Subunit
349.	HTR7	5-Hydroxytryptamine Receptor 7
350.	SCN8A	Sodium Voltage-Gated Channel Alpha Subunit 8

351.	PALB2	Partner And Localizer Of BRCA2
352.	SCN10A	Sodium Voltage-Gated Channel Alpha Subunit 10
353.	JAG1	Jagged Canonical Notch Ligand 1
354.	NOTCH2	Notch Receptor 2
355.	MYC	MYC Proto-Oncogene, BHLH Transcription Factor
356.	CPQ	Carboxypeptidase Q
357.	FBXW7	F-Box And WD Repeat Domain Containing 7
358.	RBFOX3	RNA Binding Fox-1 Homolog 3
359.	PIK3R1	Phosphoinositide-3-Kinase Regulatory Subunit 1
360.	PRKACG	Protein Kinase CAMP-Activated Catalytic Subunit Gamma
361.	FGFR3	Fibroblast Growth Factor Receptor 3
362.	CD209	CD209 Molecule
363.	CREB1	CAMP Responsive Element Binding Protein 1
364.	PTCH2	Patched 2
365.	TFRC	Transferrin Receptor
366.	CRH	Corticotropin Releasing Hormone
367.	HGF	Hepatocyte Growth Factor
368.	NHLRC1	NHL Repeat Containing E3 Ubiquitin Protein Ligase 1
369.	CDH2	Cadherin 2
370.	HRH3	Histamine Receptor H3
371.	GABRG2	Gamma-Aminobutyric Acid Type A Receptor Subunit Gamma2
372.	FCGR2B	Fc Gamma Receptor IIb
373.	MAPKAPK3	MAPK Activated Protein Kinase 3
374.	TIRAP	TIR Domain Containing Adaptor Protein
375.	NTRK2	Neurotrophic Receptor Tyrosine Kinase 2
376.	ELP1	Elongator Acetyltransferase Complex Subunit 1
377.	ADRB2	Adrenoceptor Beta 2
378.	HP	Haptoglobin
379.	GSTP1	Glutathione S-Transferase Pi 1
380.	FLCN	Folliculin
381.	CXCL9	C-X-C Motif Chemokine Ligand 9
382.	HTR1F	5-Hydroxytryptamine Receptor 1F
383.	MT-ATP8	Mitochondrially Encoded ATP Synthase Membrane Subunit 8
384.	SLC24A3	Solute Carrier Family 24 Member 3
385.	CPA6	Carboxypeptidase A6
386.	NSD1	Nuclear Receptor Binding SET Domain Protein 1
387.	KCNK5	Potassium Two Pore Domain Channel Subfamily K Member 5
388.	SCN3A	Sodium Voltage-Gated Channel Alpha Subunit 3
389.	LIG3	DNA Ligase 3
390.	SLC17A5	Solute Carrier Family 17 Member 5
391.	IL23R	Interleukin 23 Receptor

392.	C4A	Complement C4A (Chido/Rodgers Blood Group)
393.	NPPA	Natriuretic Peptide A
394.	MAPT	Microtubule Associated Protein Tau
395.	SUGCT	Succinyl-CoA:Glutarate-CoA Transferase
396.	TGFBR2	Transforming Growth Factor Beta Receptor 2
397.	SPTA1	Spectrin Alpha, Erythrocytic 1
398.	ATP4A	ATPase H+/K+ Transporting Subunit Alpha
399.	ADCYAP1R1	ADCYAP Receptor Type I
400.	ALK	ALK Receptor Tyrosine Kinase
401.	CTLA4	Cytotoxic T-Lymphocyte Associated Protein 4
402.	SLC1A2	Solute Carrier Family 1 Member 2
403.	POLG2	DNA Polymerase Gamma 2, Accessory Subunit
404.	SMARCC1	SWI/SNF Related, Matrix Associated, Actin Dependent Regulator Of Chromatin Subfamily C Member 1
405.	CALM3	Calmodulin 3
406.	CRHR1	Corticotropin Releasing Hormone Receptor 1
407.	CX3CR1	C-X3-C Motif Chemokine Receptor 1
408.	B2M	Beta-2-Microglobulin
409.	ANXA5	Annexin A5
410.	STAT6	Signal Transducer And Activator Of Transcription 6
411.	RIGI	RNA Sensor RIG-I
412.	P2RX3	Purinergic Receptor P2X 3
413.	HBA1	Hemoglobin Subunit Alpha 1
414.	TSPAN2	Tetraspanin 2
415.	ABCD1	ATP Binding Cassette Subfamily D Member 1
416.	HDAC9	Histone Deacetylase 9
417.	PF4	Platelet Factor 4
418.	SMAD3	SMAD Family Member 3
419.	ACVRL1	Activin A Receptor Like Type 1
420.	SLC39A14	Solute Carrier Family 39 Member 14
421.	GLA	Galactosidase Alpha
422.	NFKB1	Nuclear Factor Kappa B Subunit 1
423.	LRP1	LDL Receptor Related Protein 1
424.	JAK1	Janus Kinase 1
425.	DNMT1	DNA Methyltransferase 1
426.	SELE	Selectin E
427.	EDNRB	Endothelin Receptor Type B
428.	SF3B2	Splicing Factor 3b Subunit 2
429.	SCN11A	Sodium Voltage-Gated Channel Alpha Subunit 11
430.	SLC25A13	Solute Carrier Family 25 Member 13
431.	TNFRSF11A	TNF Receptor Superfamily Member 11a
432.	HBA2	Hemoglobin Subunit Alpha 2

433.	TIMP3	TIMP Metallopeptidase Inhibitor 3
434.	VCAM1	Vascular Cell Adhesion Molecule 1
435.	ATRIP	ATR Interacting Protein
436.	DYNC1H1	Dynein Cytoplasmic 1 Heavy Chain 1
437.	LMNA	Lamin A/C
438.	NOD2	Nucleotide Binding Oligomerization Domain Containing 2
439.	PTGS1	Prostaglandin-Endoperoxide Synthase 1
440.	TRPA1	Transient Receptor Potential Cation Channel Subfamily A Member 1
441.	GNAS	GNAS Complex Locus
442.	FLNA	Filamin A
443.	AIF1	Allograft Inflammatory Factor 1
444.	ASS1	Argininosuccinate Synthase 1
445.	FASLG	Fas Ligand
446.	SERPINA1	Serpin Family A Member 1
447.	GRIA1	Glutamate Ionotropic Receptor AMPA Type Subunit 1
448.	PGR	Progesterone Receptor
449.	ACTA2	Actin Alpha 2, Smooth Muscle
450.	PRTN3	Proteinase 3
451.	ORAI1	ORAI Calcium Release-Activated Calcium Modulator 1
452.	CDKN2C	Cyclin Dependent Kinase Inhibitor 2C
453.	CNTNAP2	Contactin Associated Protein 2
454.	DRD1	Dopamine Receptor D1
455.	CCNH	Cyclin H
456.	SCN4A	Sodium Voltage-Gated Channel Alpha Subunit 4
457.	CCDC88C	Coiled-Coil Domain Containing 88C
458.	IL18	Interleukin 18
459.	ATP12A	ATPase H+/K+ Transporting Non-Gastric Alpha2 Subunit
460.	KCNA3	Potassium Voltage-Gated Channel Subfamily A Member 3
461.	CYP2B6	Cytochrome P450 Family 2 Subfamily B Member 6
462.	ADGRE2	Adhesion G Protein-Coupled Receptor E2
463.	APOB	Apolipoprotein B
464.	MBP	Myelin Basic Protein
465.	TUG1	Taurine Up-Regulated 1
466.	HBG2	Hemoglobin Subunit Gamma 2
467.	CYB5A	Cytochrome B5 Type A
468.	SLC4A4	Solute Carrier Family 4 Member 4
469.	SLC19A2	Solute Carrier Family 19 Member 2
470.	ELN	Elastin
471.	SNAP25	Synaptosome Associated Protein 25
472.	CALML3	Calmodulin Like 3
473.	GHRL	Ghrelin And Obestatin Prepropeptide

474.	ERAP1	Endoplasmic Reticulum Aminopeptidase 1
475.	IL12A	Interleukin 12A
476.	UBAC2	UBA Domain Containing 2
477.	KLRC4	Killer Cell Lectin Like Receptor C4
478.	DRD5	Dopamine Receptor D5
479.	SV2A	Synaptic Vesicle Glycoprotein 2A
480.	PPIG	Peptidylprolyl Isomerase G
481.	VDR	Vitamin D Receptor
482.	HLA-DPB1	Major Histocompatibility Complex, Class II, DP Beta 1
483.	CALCRL	Calcitonin Receptor Like Receptor
484.	GRM5	Glutamate Metabotropic Receptor 5
485.	ADIPOQ	Adiponectin, C1Q And Collagen Domain Containing
486.	SQSTM1	Sequestosome 1
487.	VIPR1	Vasoactive Intestinal Peptide Receptor 1
488.	BCL2L1	BCL2 Like 1
489.	TLR7	Toll Like Receptor 7
490.	DRD3	Dopamine Receptor D3
491.	POLR3F	RNA Polymerase III Subunit F
492.	TPH1	Tryptophan Hydroxylase 1
493.	CNNM2	Cyclin And CBS Domain Divalent Metal Cation Transport Mediator 2
494.	CALML5	Calmodulin Like 5
495.	PRKACA	Protein Kinase CAMP-Activated Catalytic Subunit Alpha
496.	CALML4	Calmodulin Like 4
497.	CALML6	Calmodulin Like 6
498.	TBXT	T-Box Transcription Factor T
499.	TARDBP	TAR DNA Binding Protein
500.	INF2	Inverted Formin 2
501.	PRKACB	Protein Kinase CAMP-Activated Catalytic Subunit Beta
502.	RAMP2	Receptor Activity Modifying Protein 2
503.	MPO	Myeloperoxidase
504.	DPH1	Diphthamide Biosynthesis 1
505.	BLTP1	Bridge-Like Lipid Transfer Protein Family Member 1
506.	IGHE	Immunoglobulin Heavy Constant Epsilon
507.	CAV2	Caveolin 2
508.	MIF	Macrophage Migration Inhibitory Factor
509.	SCN1B	Sodium Voltage-Gated Channel Beta Subunit 1
510.	AOC1	Amine Oxidase Copper Containing 1
511.	IL12B	Interleukin 12B
512.	CACNA1E	Calcium Voltage-Gated Channel Subunit Alpha1 E
513.	SRPX2	Sushi Repeat Containing Protein X-Linked 2
514.	ELANE	Elastase, Neutrophil Expressed

515.	CDKN1B	Cyclin Dependent Kinase Inhibitor 1B
516.	SARDH	Sarcosine Dehydrogenase
517.	TRIM71	Tripartite Motif Containing 71
518.	GPR161	G Protein-Coupled Receptor 161
519.	ARRB1	Arrestin Beta 1
520.	SPP1	Secreted Phosphoprotein 1
521.	CYP2C9	Cytochrome P450 Family 2 Subfamily C Member 9
522.	MSH6	MutS Homolog 6
523.	CNTN2	Contactin 2
524.	LIPN	Lipase Family Member N
525.	GATA1	GATA Binding Protein 1
526.	MTDH	Metadherin
527.	GRM2	Glutamate Metabotropic Receptor 2
528.	OPN4	Opsin 4
529.	KMT2D	Lysine Methyltransferase 2D
530.	CHRM2	Cholinergic Receptor Muscarinic 2
531.	STAT1	Signal Transducer And Activator Of Transcription 1
532.	GGT1	Gamma-Glutamyltransferase 1
533.	PRICKLE2	Prickle Planar Cell Polarity Protein 2
534.	VIPR2	Vasoactive Intestinal Peptide Receptor 2
535.	TLR2	Toll Like Receptor 2
536.	EZH2	Enhancer Of Zeste 2 Polycomb Repressive Complex 2 Subunit
537.	CDKN2B	Cyclin Dependent Kinase Inhibitor 2B
538.	VEGFA	Vascular Endothelial Growth Factor A
539.	MGMT	O-6-Methylguanine-DNA Methyltransferase
540.	SERPINC1	Serpin Family C Member 1
541.	SMAD4	SMAD Family Member 4
542.	MLN	Motilin
543.	KRT76	Keratin 76
544.	PLOD1	Procollagen-Lysine,2-Oxoglutarate 5-Dioxygenase 1
545.	ARID2	AT-Rich Interaction Domain 2
546.	DARS2	Aspartyl-TRNA Synthetase 2, Mitochondrial
547.	POMGNT1	Protein O-Linked Mannose N-Acetylglucosaminyltransferase 1 (Beta 1,2-)
548.	IFNA2	Interferon Alpha 2
549.	LZTR1	Leucine Zipper Like Post Translational Regulator 1
550.	CDKN2A	Cyclin Dependent Kinase Inhibitor 2A
551.	IL1RAPL2	Interleukin 1 Receptor Accessory Protein Like 2
552.	HLA-DQA1	Major Histocompatibility Complex, Class II, DQ Alpha 1
553.	COL2A1	Collagen Type II Alpha 1 Chain
554.	RRM2B	Ribonucleotide Reductase Regulatory TP53 Inducible Subunit M2B
555.	ADAMTSL1	ADAMTS Like 1

556.	FURIN	Furin, Paired Basic Amino Acid Cleaving Enzyme
557.	CXCL10	C-X-C Motif Chemokine Ligand 10
558.	TWIST1	Twist Family BHLH Transcription Factor 1
559.	SCT	Secretin
560.	RAMP3	Receptor Activity Modifying Protein 3
561.	ADM2	Adrenomedullin 2
562.	COX5A	Cytochrome C Oxidase Subunit 5A
563.	PCNA	Proliferating Cell Nuclear Antigen
564.	HTR5A	5-Hydroxytryptamine Receptor 5A
565.	C11orf65	Chromosome 11 Open Reading Frame 65
566.	NTRK3	Neurotrophic Receptor Tyrosine Kinase 3
567.	LTA	Lymphotoxin Alpha
568.	IL3	Interleukin 3
569.	COL5A1	Collagen Type V Alpha 1 Chain
570.	GH1	Growth Hormone 1
571.	IFNAR1	Interferon Alpha And Beta Receptor Subunit 1
572.	SNCA	Synuclein Alpha
573.	PRNP	Prion Protein (Kanno Blood Group)
574.	ECM1	Extracellular Matrix Protein 1
575.	KCNK9	Potassium Two Pore Domain Channel Subfamily K Member 9
576.	ARRB2	Arrestin Beta 2
577.	GRIK1	Glutamate Ionotropic Receptor Kainate Type Subunit 1
578.	ASIC3	Acid Sensing Ion Channel Subunit 3
579.	LAPTM4A	Lysosomal Protein Transmembrane 4 Alpha
580.	RYR1	Ryanodine Receptor 1
581.	PANX1	Pannexin 1
582.	GCDH	Glutaryl-CoA Dehydrogenase
583.	PDGFRA	Platelet Derived Growth Factor Receptor Alpha
584.	PRKCD	Protein Kinase C Delta
585.	HSPD1	Heat Shock Protein Family D (Hsp60) Member 1
586.	KCNK10	Potassium Two Pore Domain Channel Subfamily K Member 10
587.	CALCB	Calcitonin Related Polypeptide Beta
588.	ERCC2	ERCC Excision Repair 2, TFIIH Core Complex Helicase Subunit
589.	EGF	Epidermal Growth Factor
590.	ERBB2	Erb-B2 Receptor Tyrosine Kinase 2
591.	NFE2L2	NFE2 Like BZIP Transcription Factor 2
592.	INSL6	Insulin Like 6
593.	SCO2	Synthesis Of Cytochrome C Oxidase 2
594.	MLH1	MutL Homolog 1
595.	CCL3	C-C Motif Chemokine Ligand 3
596.	PKD2	Polycystin 2, Transient Receptor Potential Cation Channel

597.	DICER1	Dicer 1, Ribonuclease III
598.	MMP3	Matrix Metallopeptidase 3
599.	P2RX4	Purinergic Receptor P2X 4
600.	KCNK2	Potassium Two Pore Domain Channel Subfamily K Member 2
601.	HTR1E	5-Hydroxytryptamine Receptor 1E
602.	MUC1	Mucin 1, Cell Surface Associated
603.	AFP	Alpha Fetoprotein
604.	KDR	Kinase Insert Domain Receptor
605.	GNAQ	G Protein Subunit Alpha Q
606.	OAS1	2'-5'-Oligoadenylate Synthetase 1
607.	PIEZO2	Piezo Type Mechanosensitive Ion Channel Component 2
608.	GPX3	Glutathione Peroxidase 3
609.	GDF2	Growth Differentiation Factor 2
610.	VPS4A	Vacuolar Protein Sorting 4 Homolog A
611.	CXCL12	C-X-C Motif Chemokine Ligand 12
612.	FXN	Frataxin
613.	FOXO1	Forkhead Box O1
614.	TMEM67	Transmembrane Protein 67
615.	CALCR	Calcitonin Receptor
616.	P2RX2	Purinergic Receptor P2X 2
617.	TRPV3	Transient Receptor Potential Cation Channel Subfamily V Member 3
618.	ASIC2	Acid Sensing Ion Channel Subunit 2
619.	KCNK4	Potassium Two Pore Domain Channel Subfamily K Member 4
620.	TRPV2	Transient Receptor Potential Cation Channel Subfamily V Member 2
621.	ANKDD1B	Ankyrin Repeat And Death Domain Containing 1B
622.	TUBA1A	Tubulin Alpha 1a
623.	F12	Coagulation Factor XII
624.	DNAH9	Dynein Axonemal Heavy Chain 9
625.	VIM	Vimentin
626.	RAF1	Raf-1 Proto-Oncogene, Serine/Threonine Kinase
627.	TBC1D24	TBC1 Domain Family Member 24
628.	F9	Coagulation Factor IX
629.	OLIG2	Oligodendrocyte Transcription Factor 2
630.	KCNJ5	Potassium Inwardly Rectifying Channel Subfamily J Member 5
631.	SPTAN1	Spectrin Alpha, Non-Erythrocytic 1
632.	ARX	Aristaless Related Homeobox
633.	ANKH	ANKH Inorganic Pyrophosphate Transport Regulator
634.	ERF	ETS2 Repressor Factor
635.	HADHA	Hydroxyacyl-CoA Dehydrogenase Trifunctional Multienzyme Complex Subunit Alpha
636.	OPTN	Optineurin
637.	LEMD3	LEM Domain Containing 3

638.	SETBP1	SET Binding Protein 1
639.	RPL36A-HNRNPH2	RPL36A-HNRNPH2 Readthrough
640.	PECAM1	Platelet And Endothelial Cell Adhesion Molecule 1
641.	MPZ	Myelin Protein Zero
642.	SLC12A3	Solute Carrier Family 12 Member 3
643.	GPR101	G Protein-Coupled Receptor 101
644.	CASP1	Caspase 1
645.	NAB2	NGFI-A Binding Protein 2
646.	CDK2	Cyclin Dependent Kinase 2
647.	STAT3	Signal Transducer And Activator Of Transcription 3
648.	TNNT2	Troponin T2, Cardiac Type
649.	HIF1A	Hypoxia Inducible Factor 1 Subunit Alpha
650.	CDK4	Cyclin Dependent Kinase 4
651.	KAT6B	Lysine Acetyltransferase 6B
652.	IFT56	Intraflagellar Transport 56
653.	ERVFRD-1	Endogenous Retrovirus Group FRD Member 1, Envelope
654.	CHEK2	Checkpoint Kinase 2
655.	RNASEH1	Ribonuclease H1
656.	TTN	Titin
657.	FANCI	FA Complementation Group I
658.	ZIC1	Zic Family Member 1
659.	HSPA8	Heat Shock Protein Family A (Hsp70) Member 8
660.	NR3C1	Nuclear Receptor Subfamily 3 Group C Member 1
661.	CCL5	C-C Motif Chemokine Ligand 5
662.	IL2RB	Interleukin 2 Receptor Subunit Beta
663.	MYH11	Myosin Heavy Chain 11
664.	SYN3	Synapsin III
665.	SERPINE1	Serpin Family E Member 1
666.	BMPR1A	Bone Morphogenetic Protein Receptor Type 1A
667.	SHH	Sonic Hedgehog Signaling Molecule
668.	CSTB	Cystatin B
669.	MPDZ	Multiple PDZ Domain Crumbs Cell Polarity Complex Component
670.	SULT1A3	Sulfotransferase Family 1A Member 3
671.	CASP3	Caspase 3
672.	GAST	Gastrin
673.	NES	Nestin
674.	SRSF2	Serine And Arginine Rich Splicing Factor 2
675.	PUS3	Pseudouridine Synthase 3
676.	PPARG	Peroxisome Proliferator Activated Receptor Gamma
677.	FGF2	Fibroblast Growth Factor 2
678.	ERV3-1	Endogenous Retrovirus Group 3 Member 1, Envelope

679.	DDIT3	DNA Damage Inducible Transcript 3
680.	THBD	Thrombomodulin
681.	MUTYH	MutY DNA Glycosylase
682.	KIF4A	Kinesin Family Member 4A
683.	FSD1L	Fibronectin Type III And SPRY Domain Containing 1 Like
684.	PKHD1	PKHD1 Ciliary IPT Domain Containing Fibrocystin/Polyductin
685.	GRIN1	Glutamate Ionotropic Receptor NMDA Type Subunit 1
686.	CKB	Creatine Kinase B
687.	CPT2	Carnitine Palmitoyltransferase 2
688.	KRT18	Keratin 18
689.	MYB	MYB Proto-Oncogene, Transcription Factor
690.	GPHN	Gephyrin
691.	IL5	Interleukin 5
692.	BPGM	Bisphosphoglycerate Mutase
693.	FGF14	Fibroblast Growth Factor 14
694.	USP8	Ubiquitin Specific Peptidase 8
695.	COL4A4	Collagen Type IV Alpha 4 Chain
696.	NPHS1	NPHS1 Adhesion Molecule, Nephrin
697.	ADAR	Adenosine Deaminase RNA Specific
698.	COQ8B	Coenzyme Q8B
699.	NR1I2	Nuclear Receptor Subfamily 1 Group I Member 2
700.	DSP	Desmoplakin
701.	NQO1	NAD(P)H Quinone Dehydrogenase 1
702.	ASAH1	N-Acylsphingosine Amidohydrolase 1
703.	PSTPIP1	Proline-Serine-Threonine Phosphatase Interacting Protein 1
704.	GAA	Alpha Glucosidase
705.	EPCAM	Epithelial Cell Adhesion Molecule
706.	NBN	Nibrin
707.	IGF1R	Insulin Like Growth Factor 1 Receptor
708.	PRKCH	Protein Kinase C Eta
709.	ADA	Adenosine Deaminase
710.	SELP	Selectin P
711.	MAG	Myelin Associated Glycoprotein
712.	COL1A1	Collagen Type I Alpha 1 Chain
713.	CTCF	CCCTC-Binding Factor
714.	DIAPH1	Diaphanous Related Formin 1
715.	FZD3	Frizzled Class Receptor 3
716.	NID1	Nidogen 1
717.	ARFGEF2	ADP Ribosylation Factor Guanine Nucleotide Exchange Factor 2
718.	TMEM92	Transmembrane Protein 92
719.	F10	Coagulation Factor X

720.	RANBP2	RAN Binding Protein 2
721.	HRH1	Histamine Receptor H1
722.	SERPINA6	Serpin Family A Member 6
723.	GDF6	Growth Differentiation Factor 6
724.	SLX4	SLX4 Structure-Specific Endonuclease Subunit
725.	CHGA	Chromogranin A
726.	AKR1A1	Aldo-Keto Reductase Family 1 Member A1
727.	POLD1	DNA Polymerase Delta 1, Catalytic Subunit
728.	FN1	Fibronectin 1
729.	STAT2	Signal Transducer And Activator Of Transcription 2
730.	SMARCA4	SWI/SNF Related, Matrix Associated, Actin Dependent Regulator Of Chromatin, Subfamily A, Member 4
731.	RPS26	Ribosomal Protein S26
732.	ALOX5AP	Arachidonate 5-Lipoxygenase Activating Protein
733.	IL1R1	Interleukin 1 Receptor Type 1
734.	CASP8	Caspase 8
735.	HDAC3	Histone Deacetylase 3
736.	DNM1L	Dynamin 1 Like
737.	VPS13B	Vacuolar Protein Sorting 13 Homolog B
738.	CFTR	CF Transmembrane Conductance Regulator
739.	IRF4	Interferon Regulatory Factor 4
740.	COL5A2	Collagen Type V Alpha 2 Chain
741.	SAMHD1	SAM And HD Domain Containing Deoxynucleoside Triphosphate Triphosphohydrolase 1
742.	CRY1	Cryptochrome Circadian Regulator 1
743.	IL36RN	Interleukin 36 Receptor Antagonist
744.	PRICKLE1	Prickle Planar Cell Polarity Protein 1
745.	SARS1	Seryl-TRNA Synthetase 1
746.	NEFL	Neurofilament Light Chain
747.	NCAM1	Neural Cell Adhesion Molecule 1
748.	DPP4	Dipeptidyl Peptidase 4
749.	CLCNKB	Chloride Voltage-Gated Channel Kb
750.	WASHC5	WASH Complex Subunit 5
751.	CKMT2	Creatine Kinase, Mitochondrial 2
752.	ITPR3	Inositol 1,4,5-Trisphosphate Receptor Type 3
753.	NPHS2	NPHS2 Stomatin Family Member, Podocin
754.	ITIH4	Inter-Alpha-Trypsin Inhibitor Heavy Chain 4
755.	MAP2K1	Mitogen-Activated Protein Kinase Kinase 1
756.	TIMP1	TIMP Metallopeptidase Inhibitor 1
757.	COL3A1	Collagen Type III Alpha 1 Chain
758.	WT1	WT1 Transcription Factor
759.	COL4A5	Collagen Type IV Alpha 5 Chain

760.	PLCG1	Phospholipase C Gamma 1
761.	RNASEH2B	Ribonuclease H2 Subunit B
762.	KIAA1549	KIAA1549
763.	APOL1	Apolipoprotein L1
764.	LPIN2	Lipin 2
765.	HYLS1	HYLS1 Centriolar And Ciliogenesis Associated
766.	HTR6	5-Hydroxytryptamine Receptor 6
767.	MYH7	Myosin Heavy Chain 7
768.	BAG3	BAG Cochaperone 3
769.	GRM1	Glutamate Metabotropic Receptor 1
770.	ACP5	Acid Phosphatase 5, Tartrate Resistant
771.	KCNH2	Potassium Voltage-Gated Channel Subfamily H Member 2
772.	CACNB2	Calcium Voltage-Gated Channel Auxiliary Subunit Beta 2
773.	CYP7B1	Cytochrome P450 Family 7 Subfamily B Member 1
774.	CDC73	Cell Division Cycle 73
775.	PMS1	PMS1 Homolog 1, Mismatch Repair System Component
776.	ACTA1	Actin Alpha 1, Skeletal Muscle
777.	MAF	MAF BZIP Transcription Factor
778.	KCNA6	Potassium Voltage-Gated Channel Subfamily A Member 6
779.	IER3IP1	Immediate Early Response 3 Interacting Protein 1
780.	SORD	Sorbitol Dehydrogenase
781.	CYP2E1	Cytochrome P450 Family 2 Subfamily E Member 1
782.	EPHX2	Epoxide Hydrolase 2
783.	NAT2	N-Acetyltransferase 2
784.	BCL2	BCL2 Apoptosis Regulator
785.	IGF2	Insulin Like Growth Factor 2
786.	MME	Membrane Metalloendopeptidase
787.	CKM	Creatine Kinase, M-Type
788.	MYH9	Myosin Heavy Chain 9
789.	MCM7	Minichromosome Maintenance Complex Component 7
790.	COL11A1	Collagen Type XI Alpha 1 Chain
791.	U2AF1	U2 Small Nuclear RNA Auxiliary Factor 1
792.	MEOX1	Mesenchyme Homeobox 1
793.	NTRK1	Neurotrophic Receptor Tyrosine Kinase 1
794.	GDF3	Growth Differentiation Factor 3
795.	HRH2	Histamine Receptor H2
796.	TBP	TATA-Box Binding Protein
797.	ZNF365	Zinc Finger Protein 365
798.	PTGES3	Prostaglandin E Synthase 3
799.	UGT1A1	UDP Glucuronosyltransferase Family 1 Member A1
800.	CXCR4	C-X-C Motif Chemokine Receptor 4

801.	CKMT1B	Creatine Kinase, Mitochondrial 1B
802.	MFN2	Mitofusin 2
803.	PARP1	Poly(ADP-Ribose) Polymerase 1
804.	TRH	Thyrotropin Releasing Hormone
805.	GAPDH	Glyceraldehyde-3-Phosphate Dehydrogenase
806.	CCL4	C-C Motif Chemokine Ligand 4
807.	IGFBP3	Insulin Like Growth Factor Binding Protein 3
808.	TNNI3	Troponin I3, Cardiac Type
809.	PIK3CG	Phosphatidylinositol-4,5-Bisphosphate 3-Kinase Catalytic Subunit Gamma
810.	HOXA11	Homeobox A11
811.	CD34	CD34 Molecule
812.	PLAUR	Plasminogen Activator, Urokinase Receptor
813.	PRKN	Parkin RBR E3 Ubiquitin Protein Ligase
814.	PON1	Paraoxonase 1
815.	RNASEH2C	Ribonuclease H2 Subunit C
816.	FGF6	Fibroblast Growth Factor 6
817.	CTNS	Cystinosin, Lysosomal Cystine Transporter
818.	TLR9	Toll Like Receptor 9
819.	ADORA2A	Adenosine A2a Receptor
820.	CD2AP	CD2 Associated Protein
821.	VPS35	VPS35 Retromer Complex Component
822.	TSC1	TSC Complex Subunit 1
823.	PLCE1	Phospholipase C Epsilon 1
824.	RPL11	Ribosomal Protein L11
825.	MAP4K4	Mitogen-Activated Protein Kinase Kinase Kinase Kinase 4
826.	NSD2	Nuclear Receptor Binding SET Domain Protein 2
827.	GUCY1A1	Guanylate Cyclase 1 Soluble Subunit Alpha 1
828.	IL17RD	Interleukin 17 Receptor D
829.	DNAJB11	DnaJ Heat Shock Protein Family (Hsp40) Member B11
830.	PITPNM3	PITPNM Family Member 3
831.	PREX2	Phosphatidylinositol-3,4,5-Trisphosphate Dependent Rac Exchange Factor 2
832.	SCUBE2	Signal Peptide, CUB Domain And EGF Like Domain Containing 2
833.	ZFYVE16	Zinc Finger FYVE-Type Containing 16
834.	PDCD1	Programmed Cell Death 1
835.	CIC	Capicua Transcriptional Repressor
836.	WNK3	WNK Lysine Deficient Protein Kinase 3
837.	MAGED2	MAGE Family Member D2
838.	WDR45	WD Repeat Domain 45
839.	CSF1R	Colony Stimulating Factor 1 Receptor
840.	HNF1A	HNF1 Homeobox A
841.	PCDH19	Protocadherin 19

842.	P2RY11	Purinergic Receptor P2Y11
843.	HLA-A	Major Histocompatibility Complex, Class I, A
844.	GNRH1	Gonadotropin Releasing Hormone 1
845.	IRAK1	Interleukin 1 Receptor Associated Kinase 1
846.	WWOX	WW Domain Containing Oxidoreductase
847.	GHRH	Growth Hormone Releasing Hormone
848.	MBL2	Mannose Binding Lectin 2
849.	MBD6	Methyl-CpG Binding Domain Protein 6
850.	CS	Citrate Synthase
851.	QKI	QKI, KH Domain Containing RNA Binding
852.	AVP	Arginine Vasopressin
853.	CD274	CD274 Molecule
854.	NOP56	NOP56 Ribonucleoprotein
855.	GPI	Glucose-6-Phosphate Isomerase
856.	CC2D2A	Coiled-Coil And C2 Domain Containing 2A
857.	TANGO2	Transport And Golgi Organization 2 Homolog
858.	NAMPT	Nicotinamide Phosphoribosyltransferase
859.	MMP2	Matrix Metallopeptidase 2
860.	HMGCR	3-Hydroxy-3-Methylglutaryl-CoA Reductase
861.	MAP2	Microtubule Associated Protein 2
862.	C3	Complement C3
863.	NEK1	NIMA Related Kinase 1
864.	STARD7	StAR Related Lipid Transfer Domain Containing 7
865.	CTSH	Cathepsin H
866.	GSR	Glutathione-Disulfide Reductase
867.	THBS1	Thrombospondin 1
868.	LTF	Lactotransferrin
869.	SLC6A1	Solute Carrier Family 6 Member 1
870.	PAH	Phenylalanine Hydroxylase
871.	USP48	Ubiquitin Specific Peptidase 48
872.	PTPRC	Protein Tyrosine Phosphatase Receptor Type C
873.	JUN	Jun Proto-Oncogene, AP-1 Transcription Factor Subunit
874.	NRCAM	Neuronal Cell Adhesion Molecule
875.	LGALS3	Galectin 3
876.	DMBT1	Deleted In Malignant Brain Tumors 1
877.	CRB2	Crumbs Cell Polarity Complex Component 2
878.	APOA1	Apolipoprotein A1
879.	FCGR3A	Fc Gamma Receptor IIIa
880.	ANKRD11	Ankyrin Repeat Domain Containing 11
881.	PALS1	Protein Associated With LIN7 1, MAGUK P55 Family Member
882.	FGB	Fibrinogen Beta Chain

883.	DNAH1	Dynein Axonemal Heavy Chain 1
884.	MB	Myoglobin
885.	AMACR	Alpha-Methylacyl-CoA Racemase
886.	OPA1	OPA1 Mitochondrial Dynamin Like GTPase
887.	PGK1	Phosphoglycerate Kinase 1
888.	ACTC1	Actin Alpha Cardiac Muscle 1
889.	LACTB	Lactamase Beta
890.	IFT140	Intraflagellar Transport 140
891.	PROZ	Protein Z, Vitamin K Dependent Plasma Glycoprotein
892.	RBFOX1	RNA Binding Fox-1 Homolog 1
893.	ARMC9	Armadillo Repeat Containing 9
894.	SH2D3A	SH2 Domain Containing 3A
895.	PIK3C2A	Phosphatidylinositol-4-Phosphate 3-Kinase Catalytic Subunit Type 2 Alpha
896.	CDH1	Cadherin 1
897.	CD44	CD44 Molecule (IN Blood Group)
898.	DDC	Dopa Decarboxylase
899.	CD68	CD68 Molecule
900.	MRM2	Mitochondrial RRNA Methyltransferase 2
901.	PTH	Parathyroid Hormone
902.	GJA1	Gap Junction Protein Alpha 1
903.	MTR	5-Methyltetrahydrofolate-Homocysteine Methyltransferase
904.	ADORA1	Adenosine A1 Receptor
905.	HLA-DPA1	Major Histocompatibility Complex, Class II, DP Alpha 1
906.	NUP205	Nucleoporin 205
907.	MLH3	MutL Homolog 3
908.	KITLG	KIT Ligand
909.	BAX	BCL2 Associated X, Apoptosis Regulator
910.	SCARB2	Scavenger Receptor Class B Member 2
911.	PRORP	Protein Only RNase P Catalytic Subunit
912.	CYP11B2	Cytochrome P450 Family 11 Subfamily B Member 2
913.	CYP11B1	Cytochrome P450 Family 11 Subfamily B Member 1
914.	MAGI2	Membrane Associated Guanylate Kinase, WW And PDZ Domain Containing 2
915.	NUP133	Nucleoporin 133
916.	CD86	CD86 Molecule
917.	COQ8A	Coenzyme Q8A
918.	COL1A2	Collagen Type I Alpha 2 Chain
919.	GNB1	G Protein Subunit Beta 1
920.	SGCD	Sarcoglycan Delta
921.	CYCS	Cytochrome C, Somatic
922.	ANGPT2	Angiopoietin 2
923.	ITGB3	Integrin Subunit Beta 3

924.	COQ2	Coenzyme Q2, Polyprenyltransferase
925.	GCH1	GTP Cyclohydrolase 1
926.	AP2S1	Adaptor Related Protein Complex 2 Subunit Sigma 1
927.	MAPK1	Mitogen-Activated Protein Kinase 1
928.	CYP2A6	Cytochrome P450 Family 2 Subfamily A Member 6
929.	COL4A3	Collagen Type IV Alpha 3 Chain
930.	ABCC2	ATP Binding Cassette Subfamily C Member 2
931.	GIP	Gastric Inhibitory Polypeptide
932.	SENP8	SUMO Peptidase Family Member, NEDD8 Specific
933.	GNB2	G Protein Subunit Beta 2
934.	NEU1	Neuraminidase 1
935.	IRF7	Interferon Regulatory Factor 7
936.	RNF168	Ring Finger Protein 168
937.	DDX41	DEAD-Box Helicase 41
938.	PIK3CD	Phosphatidylinositol-4,5-Bisphosphate 3-Kinase Catalytic Subunit Delta
939.	FCGR3B	Fc Gamma Receptor IIIb
940.	C9orf72	C9orf72-SMCR8 Complex Subunit
941.	NXPH4	Neurexophilin 4
942.	SLC46A1	Solute Carrier Family 46 Member 1
943.	SPTB	Spectrin Beta, Erythrocytic
944.	BICC1	BicC Family RNA Binding Protein 1
945.	ADH7	Alcohol Dehydrogenase 7 (Class IV), Mu Or Sigma Polypeptide
946.	SELL	Selectin L
947.	CLEC4M	C-Type Lectin Domain Family 4 Member M
948.	DES	Desmin
949.	RBP4	Retinol Binding Protein 4
950.	CARD9	Caspase Recruitment Domain Family Member 9
951.	ACSF3	Acyl-CoA Synthetase Family Member 3
952.	NDP	Norrin Cystine Knot Growth Factor NDP
953.	ATXN2	Ataxin 2
954.	ESR2	Estrogen Receptor 2
955.	PAPPA	Pappalysin 1
956.	MDM2	MDM2 Proto-Oncogene
957.	GSK3B	Glycogen Synthase Kinase 3 Beta
958.	L1CAM	L1 Cell Adhesion Molecule
959.	CP	Ceruloplasmin
960.	FUT9	Fucosyltransferase 9
961.	HMOX1	Heme Oxygenase 1
962.	MT-ND2	Mitochondrially Encoded NADH:Ubiquinone Oxidoreductase Core Subunit 2
963.	SSTR2	Somatostatin Receptor 2
964.	DNAI1	Dynein Axonemal Intermediate Chain 1

965.	EWSR1	EWS RNA Binding Protein 1
966.	DHFR	Dihydrofolate Reductase
967.	VANGL1	VANGL Planar Cell Polarity Protein 1
968.	SULT2A1	Sulfotransferase Family 2A Member 1
969.	PGF	Placental Growth Factor
970.	RB1	RB Transcriptional Corepressor 1
971.	CD28	CD28 Molecule
972.	BGLAP	Bone Gamma-Carboxyglutamate Protein
973.	NGFR	Nerve Growth Factor Receptor
974.	PIGA	Phosphatidylinositol Glycan Anchor Biosynthesis Class A
975.	EPB42	Erythrocyte Membrane Protein Band 4.2
976.	MRPL44	Mitochondrial Ribosomal Protein L44
977.	NCAPH2	Non-SMC Condensin II Complex Subunit H2
978.	CXCR3	C-X-C Motif Chemokine Receptor 3
979.	CDKN3	Cyclin Dependent Kinase Inhibitor 3
980.	VSX1	Visual System Homeobox 1
981.	HTT	Huntingtin
982.	XDH	Xanthine Dehydrogenase
983.	ACSL4	Acyl-CoA Synthetase Long Chain Family Member 4
984.	KCNT1	Potassium Sodium-Activated Channel Subfamily T Member 1
985.	POT1	Protection Of Telomeres 1
986.	AHDC1	AT-Hook DNA Binding Motif Containing 1
987.	TMEM47	Transmembrane Protein 47
988.	ACRBP	Acrosin Binding Protein
989.	TUNAR	TCL1 Upstream Neural Differentiation-Associated RNA
990.	TPH2	Tryptophan Hydroxylase 2
991.	TMPRSS2	Transmembrane Serine Protease 2
992.	HSP90AA1	Heat Shock Protein 90 Alpha Family Class A Member 1
993.	ARNT	Aryl Hydrocarbon Receptor Nuclear Translocator
994.	SLC6A19	Solute Carrier Family 6 Member 19
995.	TNFSF4	TNF Superfamily Member 4
996.	CLTRN	Collectrin, Amino Acid Transport Regulator
997.	PLEKHG4	Pleckstrin Homology And RhoGEF Domain Containing G4
998.	SPTBN2	Spectrin Beta, Non-Erythrocytic 2
999.	CASR	Calcium Sensing Receptor
1000.	SH2D3C	SH2 Domain Containing 3C
1001.	PCSK6	Proprotein Convertase Subtilisin/Kexin Type 6
1002.	CCL11	C-C Motif Chemokine Ligand 11
1003.	PLG	Plasminogen
1004.	FGF23	Fibroblast Growth Factor 23
1005.	LRRK2	Leucine Rich Repeat Kinase 2

1006.	EIF2AK2	Eukaryotic Translation Initiation Factor 2 Alpha Kinase 2
1007.	SOD2	Superoxide Dismutase 2
1008.	BRD2	Bromodomain Containing 2
1009.	UGT1A6	UDP Glucuronosyltransferase Family 1 Member A6
1010.	UGT1A4	UDP Glucuronosyltransferase Family 1 Member A4
1011.	UGT1A7	UDP Glucuronosyltransferase Family 1 Member A7
1012.	CSN1S1	Casein Alpha S1
1013.	CXCL13	C-X-C Motif Chemokine Ligand 13
1014.	SOX10	SRY-Box Transcription Factor 10
1015.	GANAB	Glucosidase II Alpha Subunit
1016.	ARR3	Arrestin 3
1017.	DOCK6	Dedicator Of Cytokinesis 6
1018.	SLC39A5	Solute Carrier Family 39 Member 5
1019.	AP1S3	Adaptor Related Protein Complex 1 Subunit Sigma 3
1020.	ZNF644	Zinc Finger Protein 644
1021.	KL	Klotho
1022.	DAAM2	Dishevelled Associated Activator Of Morphogenesis 2
1023.	IL15	Interleukin 15
1024.	NABP2	Nucleic Acid Binding Protein 2
1025.	PPARGC1A	PPARG Coactivator 1 Alpha
1026.	PRF1	Perforin 1
1027.	IL7	Interleukin 7
1028.	RBPJ	Recombination Signal Binding Protein For Immunoglobulin Kappa J Region
1029.	CTSS	Cathepsin S
1030.	RHOF	Ras Homolog Family Member F, Filopodia Associated
1031.	IL4R	Interleukin 4 Receptor
1032.	FGA	Fibrinogen Alpha Chain
1033.	PLA2G4A	Phospholipase A2 Group IVA
1034.	GC	GC Vitamin D Binding Protein
1035.	TRPC6	Transient Receptor Potential Cation Channel Subfamily C Member 6
1036.	RNASEH2A	Ribonuclease H2 Subunit A
1037.	LSM11	LSM11, U7 Small Nuclear RNA Associated
1038.	HSPA5	Heat Shock Protein Family A (Hsp70) Member 5
1039.	ITGAM	Integrin Subunit Alpha M
1040.	CHD7	Chromodomain Helicase DNA Binding Protein 7
1041.	SARM1	Sterile Alpha And TIR Motif Containing 1
1042.	ZIC4	Zic Family Member 4
1043.	GTPBP1	GTP Binding Protein 1
1044.	PTX3	Pentraxin 3
1045.	NTF3	Neurotrophin 3
1046.	MT-ND3	Mitochondrially Encoded NADH:Ubiquinone Oxidoreductase Core Subunit 3

1047.	FUS	FUS RNA Binding Protein
1048.	CSF1	Colony Stimulating Factor 1
1049.	TNFSF13B	TNF Superfamily Member 13b
1050.	RARB	Retinoic Acid Receptor Beta
1051.	GAL	Galanin And GMAP Prepropeptide
1052.	CARD14	Caspase Recruitment Domain Family Member 14
1053.	CTSB	Cathepsin B
1054.	CD55	CD55 Molecule (Cromer Blood Group)
1055.	TJP2	Tight Junction Protein 2
1056.	SERPINA3	Serpin Family A Member 3
1057.	SLC6A2	Solute Carrier Family 6 Member 2
1058.	BRCA1	BRCA1 DNA Repair Associated
1059.	RHOA	Ras Homolog Family Member A
1060.	CDH5	Cadherin 5
1061.	GRN	Granulin Precursor
1062.	CST3	Cystatin C
1063.	PLAU	Plasminogen Activator, Urokinase
1064.	CD19	CD19 Molecule
1065.	MECR	Mitochondrial Trans-2-Enoyl-CoA Reductase
1066.	POU1F1	POU Class 1 Homeobox 1
1067.	PTGES	Prostaglandin E Synthase
1068.	VCP	Valosin Containing Protein
1069.	EMP2	Epithelial Membrane Protein 2
1070.	NUP107	Nucleoporin 107
1071.	NUP160	Nucleoporin 160
1072.	NUP85	Nucleoporin 85
1073.	NFKBIA	NFKB Inhibitor Alpha
1074.	MAPK3	Mitogen-Activated Protein Kinase 3
1075.	CASP9	Caspase 9
1076.	KLF4	KLF Transcription Factor 4
1077.	TGFB2	Transforming Growth Factor Beta 2
1078.	PLP1	Proteolipid Protein 1
1079.	TEK	TEK Receptor Tyrosine Kinase
1080.	EZR	Ezrin
1081.	TNFSF10	TNF Superfamily Member 10
1082.	ODC1	Ornithine Decarboxylase 1
1083.	TGFBR1	Transforming Growth Factor Beta Receptor 1
1084.	WNT1	Wnt Family Member 1
1085.	MKI67	Marker Of Proliferation Ki-67
1086.	EGLN1	Egl-9 Family Hypoxia Inducible Factor 1
1087.	TNFRSF10A	TNF Receptor Superfamily Member 10a

1088.	LAMP1	Lysosomal Associated Membrane Protein 1
1089.	NPPB	Natriuretic Peptide B
1090.	BIRC5	Baculoviral IAP Repeat Containing 5
1091.	GP9	Glycoprotein IX Platelet
1092.	SPOP	Speckle Type BTB/POZ Protein
1093.	GP1BB	Glycoprotein Ib Platelet Subunit Beta
1094.	NFIX	Nuclear Factor I X
1095.	SEMA4A	Semaphorin 4A
1096.	MLX	MAX Dimerization Protein MLX
1097.	RPS20	Ribosomal Protein S20
1098.	SH2B1	SH2B Adaptor Protein 1
1099.	ADAMTS3	ADAM Metallopeptidase With Thrombospondin Type 1 Motif 3
1100.	COPB1	COPI Coat Complex Subunit Beta 1
1101.	FAN1	FANCD2 And FANCI Associated Nuclease 1
1102.	PEX11B	Peroxisomal Biogenesis Factor 11 Beta
1103.	SQOR	Sulfide Quinone Oxidoreductase
1104.	FRMD5	FERM Domain Containing 5
1105.	NEFH	Neurofilament Heavy Chain
1106.	TMEM70	Transmembrane Protein 70
1107.	GZMB	Granzyme B
1108.	HMGB1	High Mobility Group Box 1
1109.	RHBG	Rh Family B Glycoprotein
1110.	NEMP1	Nuclear Envelope Integral Membrane Protein 1
1111.	CD40	CD40 Molecule
1112.	GJB1	Gap Junction Protein Beta 1
1113.	MECP2	Methyl-CpG Binding Protein 2
1114.	BCHE	Butyrylcholinesterase
1115.	CTNND2	Catenin Delta 2
1116.	ANKFY1	Ankyrin Repeat And FYVE Domain Containing 1
1117.	MX1	MX Dynamin Like GTPase 1
1118.	GDF15	Growth Differentiation Factor 15
1119.	TCN2	Transcobalamin 2
1120.	AFF2	ALF Transcription Elongation Factor 2
1121.	DCX	Doublecortin
1122.	INSR	Insulin Receptor
1123.	CNTF	Ciliary Neurotrophic Factor
1124.	H3-3B	H3.3 Histone B
1125.	RETN	Resistin
1126.	TXN	Thioredoxin
1127.	MBTPS1	Membrane Bound Transcription Factor Peptidase, Site 1
1128.	CACNB1	Calcium Voltage-Gated Channel Auxiliary Subunit Beta 1

1129.	CACNB3	Calcium Voltage-Gated Channel Auxiliary Subunit Beta 3
1130.	PSEN2	Presenilin 2
1131.	GHR	Growth Hormone Receptor
1132.	ABL1	ABL Proto-Oncogene 1, Non-Receptor Tyrosine Kinase
1133.	GP2	Glycoprotein 2
1134.	PER3	Period Circadian Regulator 3
1135.	PIK3CB	Phosphatidylinositol-4,5-Bisphosphate 3-Kinase Catalytic Subunit Beta
1136.	EIF2B2	Eukaryotic Translation Initiation Factor 2B Subunit Beta
1137.	TJP1	Tight Junction Protein 1
1138.	CHIT1	Chitinase 1
1139.	TLR1	Toll Like Receptor 1
1140.	LIF	LIF Interleukin 6 Family Cytokine
1141.	MYOM2	Myomesin 2
1142.	RTN4	Reticulon 4
1143.	ERBB3	Erb-B2 Receptor Tyrosine Kinase 3
1144.	TNFSF11	TNF Superfamily Member 11
1145.	AQP1	Aquaporin 1 (Colton Blood Group)
1146.	RSAD2	Radical S-Adenosyl Methionine Domain Containing 2
1147.	MYBL1	MYB Proto-Oncogene Like 1
1148.	KYNU	Kynureninase
1149.	ARHGDIA	Rho GDP Dissociation Inhibitor Alpha
1150.	SELENON	Selenoprotein N
1151.	TRANK1	Tetratricopeptide Repeat And Ankyrin Repeat Containing 1
1152.	KRT7	Keratin 7
1153.	SETX	Senataxin
1154.	TAC3	Tachykinin Precursor 3
1155.	HLA-G	Major Histocompatibility Complex, Class I, G
1156.	PPP1R14A	Protein Phosphatase 1 Regulatory Inhibitor Subunit 14A
1157.	REST	RE1 Silencing Transcription Factor
1158.	IFT81	Intraflagellar Transport 81
1159.	MET	MET Proto-Oncogene, Receptor Tyrosine Kinase
1160.	NOTCH1	Notch Receptor 1
1161.	CHI3L1	Chitinase 3 Like 1
1162.	EGR1	Early Growth Response 1
1163.	COQ4	Coenzyme Q4
1164.	SOX9	SRY-Box Transcription Factor 9
1165.	PRKAR1A	Protein Kinase CAMP-Dependent Type I Regulatory Subunit Alpha
1166.	SPAST	Spastin
1167.	RAD51	RAD51 Recombinase
1168.	TFAM	Transcription Factor A, Mitochondrial
1169.	VDAC1	Voltage Dependent Anion Channel 1

1170.	RAB5A	RAB5A, Member RAS Oncogene Family
1171.	RPS27A	Ribosomal Protein S27a
1172.	NAXE	NAD(P)HX Epimerase
1173.	CXCL1	C-X-C Motif Chemokine Ligand 1
1174.	DDX3X	DEAD-Box Helicase 3 X-Linked
1175.	CCR7	C-C Motif Chemokine Receptor 7
1176.	KCNQ1	Potassium Voltage-Gated Channel Subfamily Q Member 1
1177.	GPR182	G Protein-Coupled Receptor 182
1178.	TOP2A	DNA Topoisomerase II Alpha
1179.	BCS1L	BCS1 Homolog, Ubiquinol-Cytochrome C Reductase Complex Chaperone
1180.	CD80	CD80 Molecule
1181.	SAA1	Serum Amyloid A1
1182.	GNAI1	G Protein Subunit Alpha I1
1183.	SRC	SRC Proto-Oncogene, Non-Receptor Tyrosine Kinase
1184.	DHODH	Dihydroorotate Dehydrogenase (Quinone)
1185.	KRT20	Keratin 20
1186.	TOR1A	Torsin Family 1 Member A
1187.	FLT3	Fms Related Receptor Tyrosine Kinase 3
1188.	MT-ND4L	Mitochondrially Encoded NADH:Ubiquinone Oxidoreductase Core Subunit 4L
1189.	YAP1	Yes1 Associated Transcriptional Regulator
1190.	SMARCC2	SWI/SNF Related, Matrix Associated, Actin Dependent Regulator Of Chromatin Subfamily C Member 2
1191.	KIF5A	Kinesin Family Member 5A
1192.	TSPO	Translocator Protein
1193.	POR	Cytochrome P450 Oxidoreductase
1194.	GSS	Glutathione Synthetase
1195.	ABCC3	ATP Binding Cassette Subfamily C Member 3
1196.	NR1I3	Nuclear Receptor Subfamily 1 Group I Member 3
1197.	UGT1A9	UDP Glucuronosyltransferase Family 1 Member A9
1198.	SULT1A1	Sulfotransferase Family 1A Member 1
1199.	SULT1E1	Sulfotransferase Family 1E Member 1
1200.	UGT2B15	UDP Glucuronosyltransferase Family 2 Member B15
1201.	MARK2	Microtubule Affinity Regulating Kinase 2
1202.	HAO1	Hydroxyacid Oxidase 1
1203.	UGT2B4	UDP Glucuronosyltransferase Family 2 Member B4
1204.	ERAS	ES Cell Expressed Ras
1205.	TAS2R39	Taste 2 Receptor Member 39
1206.	SULT1A4	Sulfotransferase Family 1A Member 4
1207.	NPIPB8	Nuclear Pore Complex Interacting Protein Family Member B8
1208.	PML	PML Nuclear Body Scaffold
1209.	GLMN	Glomulin, FKBP Associated Protein
1210.	ESYT1	Extended Synaptotagmin 1

1211.	STIMATE	STIM Activating Enhancer
1212.	RCOR1	REST Corepressor 1
1213.	ASL	Argininosuccinate Lyase
1214.	PRSS57	Serine Protease 57
1215.	ANGPT1	Angiopoietin 1
1216.	DAG1	Dystroglycan 1
1217.	DCN	Decorin
1218.	GLI1	GLI Family Zinc Finger 1
1219.	SOX2	SRY-Box Transcription Factor 2
1220.	NRP1	Neuropilin 1
1221.	TYMP	Thymidine Phosphorylase
1222.	MC1R	Melanocortin 1 Receptor
1223.	PLCB1	Phospholipase C Beta 1
1224.	CHN1	Chimerin 1
1225.	PPP1CB	Protein Phosphatase 1 Catalytic Subunit Beta
1226.	CHD2	Chromodomain Helicase DNA Binding Protein 2
1227.	SF3B1	Splicing Factor 3b Subunit 1
1228.	STRADA	STE20 Related Adaptor Alpha
1229.	HRG	Histidine Rich Glycoprotein
1230.	RLBP1	Retinaldehyde Binding Protein 1
1231.	NDE1	NudE Neurodevelopment Protein 1
1232.	CELA2A	Chymotrypsin Like Elastase 2A
1233.	EDAR	Ectodysplasin A Receptor
1234.	MID1	Midline 1
1235.	POGZ	Pogo Transposable Element Derived With ZNF Domain
1236.	FTCD	Formimidoyltransferase Cyclodeaminase
1237.	MTX2	Metaxin 2
1238.	PIBF1	Progesterone Immunomodulatory Binding Factor 1
1239.	SNIP1	Smad Nuclear Interacting Protein 1
1240.	ALG5	ALG5 Dolichyl-Phosphate Beta-Glucosyltransferase
1241.	ALG9	ALG9 Alpha-1,2-Mannosyltransferase
1242.	MYO18B	Myosin XVIIIB
1243.	ATG9B	Autophagy Related 9B
1244.	CEMIP2	Cell Migration Inducing Hyaluronidase 2
1245.	SZT2	SZT2 Subunit Of KICSTOR Complex
1246.	SCLT1	Sodium Channel And Clathrin Linker 1
1247.	RSRC2	Arginine And Serine Rich Coiled-Coil 2
1248.	DMBX1	Diencephalon/Mesencephalon Homeobox 1
1249.	LOC102724428	Salt Inducible Kinase 1B (Putative)
1250.	ATXN3	Ataxin 3
1251.	TNC	Tenascin C

1252.	CYP51A1	Cytochrome P450 Family 51 Subfamily A Member 1
1253.	COX6A1	Cytochrome C Oxidase Subunit 6A1
1254.	JPH3	Junctophilin 3
1255.	SDR9C7	Short Chain Dehydrogenase/Reductase Family 9C Member 7
1256.	RPS6KB1	Ribosomal Protein S6 Kinase B1
1257.	SIRT1	Sirtuin 1
1258.	IVNS1ABP	Influenza Virus NS1A Binding Protein
1259.	CLEC7A	C-Type Lectin Domain Containing 7A
1260.	TACC1	Transforming Acidic Coiled-Coil Containing Protein 1
1261.	MAPK14	Mitogen-Activated Protein Kinase 14
1262.	ITGA4	Integrin Subunit Alpha 4
1263.	FGF21	Fibroblast Growth Factor 21
1264.	AMPH	Amphiphysin
1265.	PDE4A	Phosphodiesterase 4A
1266.	CD27	CD27 Molecule
1267.	IL12RB1	Interleukin 12 Receptor Subunit Beta 1
1268.	RHD	Rh Blood Group D Antigen
1269.	LCN2	Lipocalin 2
1270.	MAVS	Mitochondrial Antiviral Signaling Protein
1271.	TNFRSF8	TNF Receptor Superfamily Member 8
1272.	HAVCR1	Hepatitis A Virus Cellular Receptor 1
1273.	CYBB	Cytochrome B-245 Beta Chain
1274.	CYP26A1	Cytochrome P450 Family 26 Subfamily A Member 1
1275.	RHOBTB2	Rho Related BTB Domain Containing 2
1276.	PIGT	Phosphatidylinositol Glycan Anchor Biosynthesis Class T
1277.	AMER1	APC Membrane Recruitment Protein 1
1278.	IL6R	Interleukin 6 Receptor
1279.	FOXG1	Forkhead Box G1
1280.	BST2	Bone Marrow Stromal Cell Antigen 2
1281.	E2F1	E2F Transcription Factor 1
1282.	SPRED1	Sprouty Related EVH1 Domain Containing 1
1283.	CTSL	Cathepsin L
1284.	PROM1	Prominin 1
1285.	SAG	S-Antigen Visual Arrestin
1286.	SLC19A3	Solute Carrier Family 19 Member 3
1287.	ANXA3	Annexin A3
1288.	S100A8	S100 Calcium Binding Protein A8
1289.	S100A12	S100 Calcium Binding Protein A12
1290.	SPARC	Secreted Protein Acidic And Cysteine Rich
1291.	ADGRG1	Adhesion G Protein-Coupled Receptor G1
1292.	CHEK1	Checkpoint Kinase 1

1293.	GNA11	G Protein Subunit Alpha 11
1294.	HLA-C	Major Histocompatibility Complex, Class I, C
1295.	MMP1	Matrix Metallopeptidase 1
1296.	PPP1CA	Protein Phosphatase 1 Catalytic Subunit Alpha
1297.	DGUOK	Deoxyguanosine Kinase
1298.	HAMP	Hepcidin Antimicrobial Peptide
1299.	PDPN	Podoplanin
1300.	TKT	Transketolase
1301.	PTK2	Protein Tyrosine Kinase 2
1302.	SSTR5	Somatostatin Receptor 5
1303.	STING1	Stimulator Of Interferon Response CGAMP Interactor 1
1304.	ATL1	Atlastin GTPase 1
1305.	GLUD1	Glutamate Dehydrogenase 1
1306.	NDUFS4	NADH:Ubiquinone Oxidoreductase Subunit S4
1307.	HSPB1	Heat Shock Protein Family B (Small) Member 1
1308.	CCND2	Cyclin D2
1309.	CFHR1	Complement Factor H Related 1
1310.	H3C2	H3 Clustered Histone 2
1311.	MRPS21	Mitochondrial Ribosomal Protein S21
1312.	SNCAIP	Synuclein Alpha Interacting Protein
1313.	NDUFV1	NADH:Ubiquinone Oxidoreductase Core Subunit V1
1314.	IL33	Interleukin 33
1315.	RNASE3	Ribonuclease A Family Member 3
1316.	XRCC3	X-Ray Repair Cross Complementing 3
1317.	ADRB3	Adrenoceptor Beta 3
1318.	BCKDHB	Branched Chain Keto Acid Dehydrogenase E1 Subunit Beta
1319.	ASPH	Aspartate Beta-Hydroxylase
1320.	PVALB	Parvalbumin
1321.	AVPR2	Arginine Vasopressin Receptor 2
1322.	C1QBP	Complement C1q Binding Protein
1323.	SLC36A2	Solute Carrier Family 36 Member 2
1324.	ABCG2	ATP Binding Cassette Subfamily G Member 2 (JR Blood Group)
1325.	SP1	Sp1 Transcription Factor
1326.	BSCL2	BSCL2 Lipid Droplet Biogenesis Associated, Seipin
1327.	SP7	Sp7 Transcription Factor
1328.	CALB2	Calbindin 2
1329.	APTX	Aprataxin
1330.	TNFAIP3	TNF Alpha Induced Protein 3
1331.	FOXO3	Forkhead Box O3
1332.	IGLON5	IgLON Family Member 5
1333.	SEC23B	SEC23 Homolog B, COPII Coat Complex Component

1334.	TLR6	Toll Like Receptor 6
1335.	DHX9	DExH-Box Helicase 9
1336.	SLC11A1	Solute Carrier Family 11 Member 1
1337.	EGLN2	Egl-9 Family Hypoxia Inducible Factor 2
1338.	TP73	Tumor Protein P73
1339.	SURF1	SURF1 Cytochrome C Oxidase Assembly Factor
1340.	MAPRE3	Microtubule Associated Protein RP/EB Family Member 3
1341.	MTRR	5-Methyltetrahydrofolate-Homocysteine Methyltransferase Reductase
1342.	IL11	Interleukin 11
1343.	CYP27B1	Cytochrome P450 Family 27 Subfamily B Member 1
1344.	PRKCB	Protein Kinase C Beta
1345.	MYO1A	Myosin IA
1346.	BACE1	Beta-Secretase 1
1347.	CEP290	Centrosomal Protein 290
1348.	NRXN3	Neurexin 3
1349.	DNAH5	Dynein Axonemal Heavy Chain 5
1350.	LAMP2	Lysosomal Associated Membrane Protein 2
1351.	GJC2	Gap Junction Protein Gamma 2
1352.	BMP2	Bone Morphogenetic Protein 2
1353.	PDSS2	Decaprenyl Diphosphate Synthase Subunit 2
1354.	CLU	Clusterin
1355.	GAD2	Glutamate Decarboxylase 2
1356.	CD276	CD276 Molecule
1357.	UCHL1	Ubiquitin C-Terminal Hydrolase L1
1358.	SHBG	Sex Hormone Binding Globulin
1359.	MYL2	Myosin Light Chain 2
1360.	CASP10	Caspase 10
1361.	ACTN4	Actinin Alpha 4
1362.	FLI1	Fli-1 Proto-Oncogene, ETS Transcription Factor
1363.	ITGA7	Integrin Subunit Alpha 7
1364.	RASGRP1	RAS Guanyl Releasing Protein 1
1365.	SAT1	Spermidine/Spermine N1-Acetyltransferase 1
1366.	TPM3	Tropomyosin 3
1367.	MAP3K20	Mitogen-Activated Protein Kinase Kinase Kinase 20
1368.	PTPRO	Protein Tyrosine Phosphatase Receptor Type O
1369.	TPM2	Tropomyosin 2
1370.	KIF23	Kinesin Family Member 23
1371.	RACGAP1	Rac GTPase Activating Protein 1
1372.	ADRA2B	Adrenoceptor Alpha 2B
1373.	FARSB	Phenylalanyl-TRNA Synthetetase Subunit Beta
1374.	MYO1E	Myosin IE

1375.	ANLN	Anillin, Actin Binding Protein
1376.	NUP93	Nucleoporin 93
1377.	HACD1	3-Hydroxyacyl-CoA Dehydratase 1
1378.	ARHGAP24	Rho GTPase Activating Protein 24
1379.	SAMD12	Sterile Alpha Motif Domain Containing 12
1380.	YEATS2	YEATS Domain Containing 2
1381.	GAPVD1	GTPase Activating Protein And VPS9 Domains 1
1382.	NUP37	Nucleoporin 37
1383.	MARCHF6	Membrane Associated Ring-CH-Type Finger 6
1384.	TBC1D8B	TBC1 Domain Family Member 8B
1385.	XIAP	X-Linked Inhibitor Of Apoptosis
1386.	PINK1	PTEN Induced Kinase 1
1387.	ZNF408	Zinc Finger Protein 408
1388.	ABT1	Activator Of Basal Transcription 1
1389.	CSF3R	Colony Stimulating Factor 3 Receptor
1390.	CLDN5	Claudin 5
1391.	RPGR	Retinitis Pigmentosa GTPase Regulator
1392.	ACADM	Acyl-CoA Dehydrogenase Medium Chain
1393.	TIMP2	TIMP Metallopeptidase Inhibitor 2
1394.	TYMS	Thymidylate Synthetase
1395.	IARS2	Isoleucyl-TRNA Synthetase 2, Mitochondrial
1396.	ATP1A4	ATPase Na+/K+ Transporting Subunit Alpha 4
1397.	SRM	Spermidine Synthase
1398.	CCR3	C-C Motif Chemokine Receptor 3
1399.	SAA4	Serum Amyloid A4, Constitutive
1400.	AFG3L2	AFG3 Like Matrix AAA Peptidase Subunit 2
1401.	RUNX2	RUNX Family Transcription Factor 2
1402.	TBX19	T-Box Transcription Factor 19
1403.	LAMA2	Laminin Subunit Alpha 2
1404.	OTX2	Orthodenticle Homeobox 2
1405.	TBX21	T-Box Transcription Factor 21
1406.	PNMA2	PNMA Family Member 2
1407.	RPRD2	Regulation Of Nuclear Pre-MRNA Domain Containing 2
1408.	NLRC4	NLR Family CARD Domain Containing 4
1409.	CD99	CD99 Molecule (Xg Blood Group)
1410.	SLC18A2	Solute Carrier Family 18 Member A2
1411.	PRKCA	Protein Kinase C Alpha
1412.	ALOX5	Arachidonate 5-Lipoxygenase
1413.	KCNJ10	Potassium Inwardly Rectifying Channel Subfamily J Member 10
1414.	RARA	Retinoic Acid Receptor Alpha
1415.	PAX6	Paired Box 6

1416.	CCNB1	Cyclin B1
1417.	AXIN1	Axin 1
1418.	CD163	CD163 Molecule
1419.	ACO1	Aconitase 1
1420.	RASSF1	Ras Association Domain Family Member 1
1421.	MAFK	MAF BZIP Transcription Factor K
1422.	DTX3	Deltex E3 Ubiquitin Ligase 3
1423.	ENDOV	Endonuclease V
1424.	SLMAP	Sarcolemma Associated Protein
1425.	MYOC	Myocilin
1426.	TREM2	Triggering Receptor Expressed On Myeloid Cells 2
1427.	SMN1	Survival Of Motor Neuron 1, Telomeric
1428.	SMN2	Survival Of Motor Neuron 2, Centromeric
1429.	GDAP1	Ganglioside Induced Differentiation Associated Protein 1
1430.	OFD1	OFD1 Centriole And Centriolar Satellite Protein
1431.	IRF1	Interferon Regulatory Factor 1
1432.	LYZ	Lysozyme
1433.	TLR8	Toll Like Receptor 8
1434.	POU5F1	POU Class 5 Homeobox 1
1435.	ACADVL	Acyl-CoA Dehydrogenase Very Long Chain
1436.	GAP43	Growth Associated Protein 43
1437.	POMT1	Protein O-Mannosyltransferase 1
1438.	UNC13D	Unc-13 Homolog D
1439.	BCR	BCR Activator Of RhoGEF And GTPase
1440.	NKX2-1	NK2 Homeobox 1
1441.	ALAD	Aminolevulinate Dehydratase
1442.	CD38	CD38 Molecule
1443.	TREM1	Triggering Receptor Expressed On Myeloid Cells 1
1444.	COX15	Cytochrome C Oxidase Assembly Homolog COX15
1445.	STOM	Stomatin
1446.	ADAM17	ADAM Metallopeptidase Domain 17
1447.	DPP10	Dipeptidyl Peptidase Like 10
1448.	IL22	Interleukin 22
1449.	TMBIM4	Transmembrane BAX Inhibitor Motif Containing 4
1450.	FGF9	Fibroblast Growth Factor 9
1451.	SLC22A5	Solute Carrier Family 22 Member 5
1452.	P4HB	Prolyl 4-Hydroxylase Subunit Beta
1453.	AARS2	Alanyl-TRNA Synthetase 2, Mitochondrial
1454.	CD59	CD59 Molecule (CD59 Blood Group)
1455.	PROCR	Protein C Receptor
1456.	CACNA2D1	Calcium Voltage-Gated Channel Auxiliary Subunit Alpha2delta 1

1457.	PHOX2B	Paired Like Homeobox 2B
1458.	ABCC1	ATP Binding Cassette Subfamily C Member 1 (ABCC1 Blood Group)
1459.	STAT5A	Signal Transducer And Activator Of Transcription 5A
1460.	ITGAX	Integrin Subunit Alpha X
1461.	ELOB	Elongin B
1462.	IL13RA2	Interleukin 13 Receptor Subunit Alpha 2
1463.	CALB1	Calbindin 1
1464.	KLF1	KLF Transcription Factor 1
1465.	CDK6	Cyclin Dependent Kinase 6
1466.	ABCC8	ATP Binding Cassette Subfamily C Member 8
1467.	CHAT	Choline O-Acetyltransferase
1468.	ATXN7	Ataxin 7
1469.	CFP	Complement Factor Properdin
1470.	CYTH1	Cytohesin 1
1471.	CDK5	Cyclin Dependent Kinase 5
1472.	BIN1	Bridging Integrator 1
1473.	ADH1B	Alcohol Dehydrogenase 1B (Class I), Beta Polypeptide
1474.	FECH	Ferrochelatase
1475.	IL23A	Interleukin 23 Subunit Alpha
1476.	TSPAN31	Tetraspanin 31
1477.	AKT3	AKT Serine/Threonine Kinase 3
1478.	IFITM3	Interferon Induced Transmembrane Protein 3
1479.	LPL	Lipoprotein Lipase
1480.	CTSG	Cathepsin G
1481.	KCND3	Potassium Voltage-Gated Channel Subfamily D Member 3
1482.	DLL4	Delta Like Canonical Notch Ligand 4
1483.	SYNGAP1	Synaptic Ras GTPase Activating Protein 1
1484.	SSBP1	Single Stranded DNA Binding Protein 1
1485.	FBXL4	F-Box And Leucine Rich Repeat Protein 4
1486.	EIF2B4	Eukaryotic Translation Initiation Factor 2B Subunit Delta
1487.	LARS2	Leucyl-TRNA Synthetase 2, Mitochondrial
1488.	INHA	Inhibin Subunit Alpha
1489.	COQ9	Coenzyme Q9
1490.	PDSS1	Decaprenyl Diphosphate Synthase Subunit 1
1491.	YBX1	Y-Box Binding Protein 1
1492.	EIF5B	Eukaryotic Translation Initiation Factor 5B
1493.	ANKRD46	Ankyrin Repeat Domain 46
1494.	RFX6	Regulatory Factor X6
1495.	ALPL	Alkaline Phosphatase, Biomineralization Associated
1496.	BSG	Basigin (Ok Blood Group)
1497.	TRDN	Triadin

1498.	ABCA1	ATP Binding Cassette Subfamily A Member 1
1499.	ARG1	Arginase 1
1500.	CPS1	Carbamoyl-Phosphate Synthase 1
1501.	GPT2	Glutamic--Pyruvic Transaminase 2
1502.	NDUFS7	NADH:Ubiquinone Oxidoreductase Core Subunit S7
1503.	IGFBP2	Insulin Like Growth Factor Binding Protein 2
1504.	PDGFA	Platelet Derived Growth Factor Subunit A
1505.	CUL2	Cullin 2
1506.	MXI1	MAX Interactor 1, Dimerization Protein
1507.	AIM2	Absent In Melanoma 2
1508.	IFNA4	Interferon Alpha 4
1509.	GAMT	Guanidinoacetate N-Methyltransferase
1510.	CLN6	CLN6 Transmembrane ER Protein
1511.	CDC42	Cell Division Cycle 42
1512.	TUBB3	Tubulin Beta 3 Class III
1513.	MRPS2	Mitochondrial Ribosomal Protein S2
1514.	ZFHX2	Zinc Finger Homeobox 2
1515.	ALDH2	Aldehyde Dehydrogenase 2 Family Member
1516.	CRYAB	Crystallin Alpha B
1517.	TF	Transferrin
1518.	SYK	Spleen Associated Tyrosine Kinase
1519.	C9	Complement C9
1520.	TNFRSF10B	TNF Receptor Superfamily Member 10b
1521.	STAT5B	Signal Transducer And Activator Of Transcription 5B
1522.	LEF1	Lymphoid Enhancer Binding Factor 1
1523.	COX10	Cytochrome C Oxidase Assembly Factor Heme A:Farnesyltransferase COX10
1524.	SSTR1	Somatostatin Receptor 1
1525.	ELOC	Elongin C
1526.	MSI1	Musashi RNA Binding Protein 1
1527.	KBTBD4	Kelch Repeat And BTB Domain Containing 4
1528.	NTNG2	Netrin G2
1529.	NR3C2	Nuclear Receptor Subfamily 3 Group C Member 2
1530.	ERBB4	Erb-B2 Receptor Tyrosine Kinase 4
1531.	RNF216	Ring Finger Protein 216
1532.	CDKL5	Cyclin Dependent Kinase Like 5
1533.	BCAR1	BCAR1 Scaffold Protein, Cas Family Member
1534.	ADAMTSL4	ADAMTS Like 4
1535.	SEMA6C	Semaphorin 6C
1536.	CA5A	Carbonic Anhydrase 5A
1537.	TUBB4A	Tubulin Beta 4A Class IVa
1538.	IFNAR2	Interferon Alpha And Beta Receptor Subunit 2

1539.	ENPEP	Glutamyl Aminopeptidase
1540.	GLRX	Glutaredoxin
1541.	TRMU	TRNA Mitochondrial 2-Thiouridylase
1542.	SPCS1	Signal Peptidase Complex Subunit 1
1543.	CYP2C8	Cytochrome P450 Family 2 Subfamily C Member 8
1544.	IRS1	Insulin Receptor Substrate 1
1545.	TGFB3	Transforming Growth Factor Beta 3
1546.	PNMT	Phenylethanolamine N-Methyltransferase
1547.	SOCS3	Suppressor Of Cytokine Signaling 3
1548.	SPG7	SPG7 Matrix AAA Peptidase Subunit, Paraplegin
1549.	CTRL	Chymotrypsin Like
1550.	DPYSL5	Dihydropyrimidinase Like 5
1551.	HDAC1	Histone Deacetylase 1
1552.	MYOD1	Myogenic Differentiation 1
1553.	ATP1B2	ATPase Na+/K+ Transporting Subunit Beta 2
1554.	PTGDS	Prostaglandin D2 Synthase
1555.	HBE1	Hemoglobin Subunit Epsilon 1
1556.	ACVR1	Activin A Receptor Type 1
1557.	AXL	AXL Receptor Tyrosine Kinase
1558.	TRAF6	TNF Receptor Associated Factor 6
1559.	H6PD	Hexose-6-Phosphate Dehydrogenase/Glucose 1-Dehydrogenase
1560.	CCL7	C-C Motif Chemokine Ligand 7
1561.	FOXM1	Forkhead Box M1
1562.	YARS2	Tyrosyl-TRNA Synthetase 2
1563.	CD69	CD69 Molecule
1564.	TSFM	Ts Translation Elongation Factor, Mitochondrial
1565.	MTO1	Mitochondrial TRNA Translation Optimization 1
1566.	EARS2	Glutamyl-TRNA Synthetase 2, Mitochondrial
1567.	YY1AP1	YY1 Associated Protein 1
1568.	AAAS	Aladin WD Repeat Nucleoporin
1569.	STK24	Serine/Threonine Kinase 24
1570.	ANKLE2	Ankyrin Repeat And LEM Domain Containing 2
1571.	ALDH7A1	Aldehyde Dehydrogenase 7 Family Member A1
1572.	CGA	Glycoprotein Hormones, Alpha Polypeptide
1573.	GLI2	GLI Family Zinc Finger 2
1574.	VEGFC	Vascular Endothelial Growth Factor C
1575.	NDUFS2	NADH:Ubiquinone Oxidoreductase Core Subunit S2
1576.	NDUFAF1	NADH:Ubiquinone Oxidoreductase Complex Assembly Factor 1
1577.	PTTG1	PTTG1 Regulator Of Sister Chromatid Separation, Securin
1578.	PROP1	PROP Paired-Like Homeobox 1
1579.	TMEM126B	Transmembrane Protein 126B

1580.	CACNA1I	Calcium Voltage-Gated Channel Subunit Alpha1 I
1581.	AIFM1	Apoptosis Inducing Factor Mitochondria Associated 1
1582.	VLDLR	Very Low Density Lipoprotein Receptor
1583.	DCTN1	Dynactin Subunit 1
1584.	PURA	Purine Rich Element Binding Protein A
1585.	CTSD	Cathepsin D
1586.	EIF2B5	Eukaryotic Translation Initiation Factor 2B Subunit Epsilon
1587.	POLR3B	RNA Polymerase III Subunit B
1588.	BCKDHA	Branched Chain Keto Acid Dehydrogenase E1 Subunit Alpha
1589.	DNAAF2	Dynein Axonemal Assembly Factor 2
1590.	S100A1	S100 Calcium Binding Protein A1
1591.	DMD	Dystrophin
1592.	GAD1	Glutamate Decarboxylase 1
1593.	LRP6	LDL Receptor Related Protein 6
1594.	MYOG	Myogenin
1595.	TCHP	Trichoplein Keratin Filament Binding
1596.	MANBA	Mannosidase Beta
1597.	SLC12A2	Solute Carrier Family 12 Member 2
1598.	TNFRSF11B	TNF Receptor Superfamily Member 11b
1599.	AQP2	Aquaporin 2
1600.	CCN2	Cellular Communication Network Factor 2
1601.	CRX	Cone-Rod Homeobox
1602.	ASMT	Acetylserotonin O-Methyltransferase
1603.	IFNA21	Interferon Alpha 21
1604.	PLA2G6	Phospholipase A2 Group VI
1605.	PKP2	Plakophilin 2
1606.	SDCCAG8	SHH Signaling And Ciliogenesis Regulator SDCCAG8
1607.	LRP4	LDL Receptor Related Protein 4
1608.	ARHGAP1	Rho GTPase Activating Protein 1
1609.	KIF3B	Kinesin Family Member 3B
1610.	PRUNE1	Prune Exopolyphosphatase 1
1611.	CAMK1D	Calcium/Calmodulin Dependent Protein Kinase ID
1612.	PRIM1	DNA Primase Subunit 1
1613.	TARS2	Threonyl-TRNA Synthetase 2, Mitochondrial
1614.	HSD17B6	Hydroxysteroid 17-Beta Dehydrogenase 6
1615.	HJURP	Holliday Junction Recognition Protein
1616.	CCT3	Chaperonin Containing TCP1 Subunit 3
1617.	R3HDM2	R3H Domain Containing 2
1618.	UFL1	UFM1 Specific Ligase 1
1619.	ANKRD52	Ankyrin Repeat Domain 52
1620.	C1orf56	Chromosome 1 Open Reading Frame 56

1621.	MSL3B	MSL Complex Subunit 3B
1622.	SLC12A6	Solute Carrier Family 12 Member 6
1623.	LIN28B	Lin-28 Homolog B
1624.	CDCA7L	Cell Division Cycle Associated 7 Like
1625.	STK26	Serine/Threonine Kinase 26
1626.	STK25	Serine/Threonine Kinase 25
1627.	LEPR	Leptin Receptor
1628.	TNFRSF1B	TNF Receptor Superfamily Member 1B
1629.	PLA2G2A	Phospholipase A2 Group IIA
1630.	CD2	CD2 Molecule
1631.	DLL1	Delta Like Canonical Notch Ligand 1
1632.	AGTR2	Angiotensin II Receptor Type 2
1633.	COX4I2	Cytochrome C Oxidase Subunit 4I2
1634.	HTATIP2	HIV-1 Tat Interactive Protein 2
1635.	RASA2	RAS P21 Protein Activator 2
1636.	EZHIP	EZH Inhibitory Protein
1637.	ACTB	Actin Beta
1638.	MAPK8	Mitogen-Activated Protein Kinase 8
1639.	PRDX5	Peroxiredoxin 5
1640.	IL1RL2	Interleukin 1 Receptor Like 2
1641.	SFRP1	Secreted Frizzled Related Protein 1
1642.	SPI1	Spi-1 Proto-Oncogene
1643.	FCGR1A	Fc Gamma Receptor Ia
1644.	CLEC4G	C-Type Lectin Domain Family 4 Member G
1645.	LGALS13	Galectin 13
1646.	NOTCH4	Notch Receptor 4
1647.	CA3	Carbonic Anhydrase 3
1648.	STK11	Serine/Threonine Kinase 11
1649.	PDE4D	Phosphodiesterase 4D
1650.	TGIF1	TGFB Induced Factor Homeobox 1
1651.	TOP1	DNA Topoisomerase I
1652.	OAS3	2'-5'-Oligoadenylate Synthetase 3
1653.	SHMT1	Serine Hydroxymethyltransferase 1
1654.	GPX4	Glutathione Peroxidase 4
1655.	PAX3	Paired Box 3
1656.	EGLN3	Egl-9 Family Hypoxia Inducible Factor 3
1657.	HSD11B2	Hydroxysteroid 11-Beta Dehydrogenase 2
1658.	SOCS1	Suppressor Of Cytokine Signaling 1
1659.	COX4I1	Cytochrome C Oxidase Subunit 4I1
1660.	SCO1	Synthesis Of Cytochrome C Oxidase 1
1661.	ACAD9	Acyl-CoA Dehydrogenase Family Member 9

1662.	CD63	CD63 Molecule
1663.	IL18R1	Interleukin 18 Receptor 1
1664.	NDUFV2	NADH:Ubiquinone Oxidoreductase Core Subunit V2
1665.	COX6B1	Cytochrome C Oxidase Subunit 6B1
1666.	MRPS22	Mitochondrial Ribosomal Protein S22
1667.	TMEM126A	Transmembrane Protein 126A
1668.	COX6A2	Cytochrome C Oxidase Subunit 6A2
1669.	MTERF1	Mitochondrial Transcription Termination Factor 1
1670.	VHLL	VHL Like
1671.	PAX5	Paired Box 5
1672.	IRF9	Interferon Regulatory Factor 9
1673.	MXRA8	Matrix Remodeling Associated 8
1674.	MARVELD3	MARVEL Domain Containing 3
1675.	PMM2	Phosphomannomutase 2
1676.	CA2	Carbonic Anhydrase 2
1677.	TPI1	Triosephosphate Isomerase 1
1678.	KLRC1	Killer Cell Lectin Like Receptor C1
1679.	AR	Androgen Receptor
1680.	ITGB1	Integrin Subunit Beta 1
1681.	MUSK	Muscle Associated Receptor Tyrosine Kinase
1682.	HPRT1	Hypoxanthine Phosphoribosyltransferase 1
1683.	MSN	Moesin
1684.	BCL2L11	BCL2 Like 11
1685.	RBP3	Retinol Binding Protein 3
1686.	H3C12	H3 Clustered Histone 12
1687.	CLCN6	Chloride Voltage-Gated Channel 6
1688.	BMP1	Bone Morphogenetic Protein 1
1689.	POLR3A	RNA Polymerase III Subunit A
1690.	CYP19A1	Cytochrome P450 Family 19 Subfamily A Member 1
1691.	LYN	LYN Proto-Oncogene, Src Family Tyrosine Kinase
1692.	AURKA	Aurora Kinase A
1693.	ISG15	ISG15 Ubiquitin Like Modifier
1694.	PHEX	Phosphate Regulating Endopeptidase X-Linked
1695.	VAC14	VAC14 Component Of PIKFYVE Complex
1696.	PDGFC	Platelet Derived Growth Factor C
1697.	CRYGC	Crystallin Gamma C
1698.	NR4A2	Nuclear Receptor Subfamily 4 Group A Member 2
1699.	LRP2	LDL Receptor Related Protein 2
1700.	SIL1	SIL1 Nucleotide Exchange Factor
1701.	LBP	Lipopolysaccharide Binding Protein
1702.	KIR3DL1	Killer Cell Immunoglobulin Like Receptor, Three Ig Domains And Long Cytoplasmic Tail 1

1703.	ARSA	Arylsulfatase A
1704.	TMEM216	Transmembrane Protein 216
1705.	SCNN1A	Sodium Channel Epithelial 1 Subunit Alpha
1706.	KCNJ11	Potassium Inwardly Rectifying Channel Subfamily J Member 11
1707.	GABRE	Gamma-Aminobutyric Acid Type A Receptor Subunit Epsilon
1708.	ATXN1	Ataxin 1
1709.	CREB3L1	CAMP Responsive Element Binding Protein 3 Like 1
1710.	KRT8	Keratin 8
1711.	PTPRS	Protein Tyrosine Phosphatase Receptor Type S
1712.	IGFBP1	Insulin Like Growth Factor Binding Protein 1
1713.	NARS2	Asparaginyl-TRNA Synthetase 2, Mitochondrial
1714.	COQ6	Coenzyme Q6, Monooxygenase
1715.	RARS2	Arginyl-TRNA Synthetase 2, Mitochondrial
1716.	TFB1M	Transcription Factor B1, Mitochondrial
1717.	TMPRSS11D	Transmembrane Serine Protease 11D
1718.	DMTN	Dematin Actin Binding Protein
1719.	QTRT1	Queuine TRNA-Ribosyltransferase Catalytic Subunit 1
1720.	LMLN	Leishmanolysin Like Peptidase
1721.	PSMB8	Proteasome 20S Subunit Beta 8
1722.	SCARB1	Scavenger Receptor Class B Member 1
1723.	RPSA	Ribosomal Protein SA
1724.	C5AR1	Complement C5a Receptor 1
1725.	BMI1	BMI1 Proto-Oncogene, Polycomb Ring Finger
1726.	NDUFS8	NADH:Ubiquinone Oxidoreductase Core Subunit S8
1727.	WARS2	Tryptophanyl TRNA Synthetase 2, Mitochondrial
1728.	CLPX	Caseinolytic Mitochondrial Matrix Peptidase Chaperone Subunit X
1729.	NDUFB8	NADH:Ubiquinone Oxidoreductase Subunit B8
1730.	COX8A	Cytochrome C Oxidase Subunit 8A
1731.	YBX3	Y-Box Binding Protein 3
1732.	TIMMDC1	Translocase Of Inner Mitochondrial Membrane Domain Containing 1
1733.	CCM2L	CCM2 Like Scaffold Protein
1734.	NKX2-5	NK2 Homeobox 5
1735.	RDX	Radixin
1736.	IHH	Indian Hedgehog Signaling Molecule
1737.	AMBP	Alpha-1-Microglobulin/Bikunin Precursor
1738.	IL9	Interleukin 9
1739.	HEY1	Hes Related Family BHLH Transcription Factor With YRPW Motif 1
1740.	MICB	MHC Class I Polypeptide-Related Sequence B
1741.	HEY2	Hes Related Family BHLH Transcription Factor With YRPW Motif 2
1742.	PATZ1	POZ/BTB And AT Hook Containing Zinc Finger 1
1743.	FTL	Ferritin Light Chain

1744.	ATG5	Autophagy Related 5
1745.	MYH6	Myosin Heavy Chain 6
1746.	CIITA	Class II Major Histocompatibility Complex Transactivator
1747.	PPIB	Peptidylprolyl Isomerase B
1748.	SPTLC1	Serine Palmitoyltransferase Long Chain Base Subunit 1
1749.	MMP12	Matrix Metallopeptidase 12
1750.	BEST1	Bestrophin 1
1751.	UTS2	Urotensin 2
1752.	STRN	Striatin
1753.	STK36	Serine/Threonine Kinase 36
1754.	TYK2	Tyrosine Kinase 2
1755.	GRB2	Growth Factor Receptor Bound Protein 2
1756.	CD83	CD83 Molecule
1757.	FOXR2	Forkhead Box R2
1758.	PTK2B	Protein Tyrosine Kinase 2 Beta
1759.	PDXK	Pyridoxal Kinase
1760.	CYP1A1	Cytochrome P450 Family 1 Subfamily A Member 1
1761.	IFITM1	Interferon Induced Transmembrane Protein 1
1762.	UBQLN2	Ubiquilin 2
1763.	FOXJ1	Forkhead Box J1
1764.	CD79A	CD79a Molecule
1765.	CTNNA1	Catenin Alpha 1
1766.	PKLR	Pyruvate Kinase L/R
1767.	HBS1L	HBS1 Like Translational GTPase
1768.	IRAK4	Interleukin 1 Receptor Associated Kinase 4
1769.	NR5A1	Nuclear Receptor Subfamily 5 Group A Member 1
1770.	GATA4	GATA Binding Protein 4
1771.	UBQLN4	Ubiquilin 4
1772.	GNAO1	G Protein Subunit Alpha O1
1773.	PMP22	Peripheral Myelin Protein 22
1774.	CCR2	C-C Motif Chemokine Receptor 2
1775.	NLGN1	Neuroligin 1
1776.	SGSH	N-Sulfoglucosamine Sulfohydrolase
1777.	SOS1	SOS Ras/Rac Guanine Nucleotide Exchange Factor 1
1778.	HSP90AB1	Heat Shock Protein 90 Alpha Family Class B Member 1
1779.	RAD50	RAD50 Double Strand Break Repair Protein
1780.	ITGA2	Integrin Subunit Alpha 2
1781.	EPB41	Erythrocyte Membrane Protein Band 4.1
1782.	IFNA5	Interferon Alpha 5
1783.	CLEC6A	C-Type Lectin Domain Containing 6A
1784.	IFNA6	Interferon Alpha 6

1785.	IFNA14	Interferon Alpha 14
1786.	IFNA8	Interferon Alpha 8
1787.	IFNA10	Interferon Alpha 10
1788.	IFNA13	Interferon Alpha 13
1789.	IFNA7	Interferon Alpha 7
1790.	IFNA16	Interferon Alpha 16
1791.	CYP17A1	Cytochrome P450 Family 17 Subfamily A Member 1
1792.	C1R	Complement C1r
1793.	ITGAL	Integrin Subunit Alpha L
1794.	PDE4B	Phosphodiesterase 4B
1795.	MITF	Melanocyte Inducing Transcription Factor
1796.	P2RY12	Purinergic Receptor P2Y12
1797.	AGER	Advanced Glycosylation End-Product Specific Receptor
1798.	KCNA2	Potassium Voltage-Gated Channel Subfamily A Member 2
1799.	GJB2	Gap Junction Protein Beta 2
1800.	PXDN	Peroxidasin
1801.	ACP1	Acid Phosphatase 1
1802.	PLXND1	Plexin D1
1803.	PMF1	Polyamine Modulated Factor 1
1804.	SLC26A11	Solute Carrier Family 26 Member 11
1805.	IRAG1	Inositol 1,4,5-Triphosphate Receptor Associated 1
1806.	SLC25A44	Solute Carrier Family 25 Member 44
1807.	ARF1	ADP Ribosylation Factor 1
1808.	CD14	CD14 Molecule
1809.	HAVCR2	Hepatitis A Virus Cellular Receptor 2
1810.	MCM3	Minichromosome Maintenance Complex Component 3
1811.	BDKRB2	Bradykinin Receptor B2
1812.	EPHX1	Epoxide Hydrolase 1
1813.	KPNB1	Karyopherin Subunit Beta 1
1814.	UQCRC2	Ubiquinol-Cytochrome C Reductase Core Protein 2
1815.	PUS1	Pseudouridine Synthase 1
1816.	ELAC2	ElaC Ribonuclease Z 2
1817.	UQCRB	Ubiquinol-Cytochrome C Reductase Binding Protein
1818.	VARS1	Valyl-TRNA Synthetase 1
1819.	LAG3	Lymphocyte Activating 3
1820.	TRNT1	TRNA Nucleotidyl Transferase 1
1821.	VARS2	Valyl-TRNA Synthetase 2, Mitochondrial
1822.	RAB8A	RAB8A, Member RAS Oncogene Family
1823.	ARL13B	ADP Ribosylation Factor Like GTPase 13B
1824.	GTPBP3	GTP Binding Protein 3, Mitochondrial
1825.	PAPPA2	Pappalysin 2

1826.	CAPRIN1	Cell Cycle Associated Protein 1
1827.	FASTKD2	FAST Kinase Domains 2
1828.	ILF2	Interleukin Enhancer Binding Factor 2
1829.	UCN	Urocortin
1830.	CDK5RAP1	CDK5 Regulatory Subunit Associated Protein 1
1831.	MTUS1	Microtubule Associated Scaffold Protein 1
1832.	OSGEPL1	O-Sialoglycoprotein Endopeptidase Like 1
1833.	MTUS2	Microtubule Associated Scaffold Protein 2
1834.	COA3	Cytochrome C Oxidase Assembly Factor 3
1835.	TRMT61B	TRNA Methyltransferase 61B
1836.	TRIM52	Tripartite Motif Containing 52
1837.	KIF1A	Kinesin Family Member 1A
1838.	AQP4	Aquaporin 4
1839.	CD1A	CD1a Molecule
1840.	CXCR2	C-X-C Motif Chemokine Receptor 2
1841.	PRKG1	Protein Kinase CGMP-Dependent 1
1842.	ANXA2	Annexin A2
1843.	CYP1B1	Cytochrome P450 Family 1 Subfamily B Member 1
1844.	PSMB9	Proteasome 20S Subunit Beta 9
1845.	CXCL11	C-X-C Motif Chemokine Ligand 11
1846.	SYNJ2BP	Synaptojanin 2 Binding Protein
1847.	LDLRAD3	Low Density Lipoprotein Receptor Class A Domain Containing 3
1848.	EIF2B3	Eukaryotic Translation Initiation Factor 2B Subunit Gamma
1849.	EPHB2	EPH Receptor B2
1850.	HSD11B1	Hydroxysteroid 11-Beta Dehydrogenase 1
1851.	JAK3	Janus Kinase 3
1852.	PKM	Pyruvate Kinase M1/2
1853.	SLC11A2	Solute Carrier Family 11 Member 2
1854.	ZEB1	Zinc Finger E-Box Binding Homeobox 1
1855.	F7	Coagulation Factor VII
1856.	RHO	Rhodopsin
1857.	ADD1	Adducin 1
1858.	INVS	Inversin
1859.	JAG2	Jagged Canonical Notch Ligand 2
1860.	RAP1A	RAP1A, Member Of RAS Oncogene Family
1861.	ARID1B	AT-Rich Interaction Domain 1B
1862.	MMACHC	Metabolism Of Cobalamin Associated C
1863.	RBX1	Ring-Box 1
1864.	AMD1	Adenosylmethionine Decarboxylase 1
1865.	CLDN11	Claudin 11
1866.	LARGE1	LARGE Xylosyl- And Glucuronyltransferase 1

1867.	CHGB	Chromogranin B
1868.	LGI2	Leucine Rich Repeat LGI Family Member 2
1869.	ATOH1	Atonal BHLH Transcription Factor 1
1870.	BLZF1	Basic Leucine Zipper Nuclear Factor 1
1871.	RPH3AL	Rabphilin 3A Like (Without C2 Domains)
1872.	CGAS	Cyclic GMP-AMP Synthase
1873.	MAMLD1	Mastermind Like Domain Containing 1
1874.	IFNA17	Interferon Alpha 17
1875.	PYDC1	Pyrin Domain Containing 1
1876.	NNAT	Neuronatin
1877.	PNKP	Polynucleotide Kinase 3'-Phosphatase
1878.	SERPINI1	Serpin Family I Member 1
1879.	NPFF	Neuropeptide FF-Amide Peptide Precursor
1880.	GLP1R	Glucagon Like Peptide 1 Receptor
1881.	SLC40A1	Solute Carrier Family 40 Member 1
1882.	CD47	CD47 Molecule
1883.	HABP2	Hyaluronan Binding Protein 2
1884.	FOXC1	Forkhead Box C1
1885.	ICMT	Isoprenylcysteine Carboxyl Methyltransferase
1886.	RAB1B	RAB1B, Member RAS Oncogene Family
1887.	ARSD	Arylsulfatase D
1888.	SEC22B	SEC22 Homolog B, Vesicle Trafficking Protein
1889.	ZEB2	Zinc Finger E-Box Binding Homeobox 2
1890.	MCOLN1	Mucolipin TRP Cation Channel 1
1891.	OPHN1	Oligophrenin 1
1892.	EIF2B1	Eukaryotic Translation Initiation Factor 2B Subunit Alpha
1893.	NALCN	Sodium Leak Channel, Non-Selective
1894.	TBCK	TBC1 Domain Containing Kinase
1895.	CHMP2B	Charged Multivesicular Body Protein 2B
1896.	VAPB	VAMP Associated Protein B And C
1897.	KLHL3	Kelch Like Family Member 3
1898.	CHCHD10	Coiled-Coil-Helix-Coiled-Coil-Helix Domain Containing 10
1899.	THAP1	THAP Domain Containing 1
1900.	HEXA	Hexosaminidase Subunit Alpha
1901.	SLC29A3	Solute Carrier Family 29 Member 3
1902.	KLLN	Killin, P53 Regulated DNA Replication Inhibitor
1903.	VWA8	Von Willebrand Factor A Domain Containing 8
1904.	KRT14	Keratin 14
1905.	CA9	Carbonic Anhydrase 9
1906.	SYNM	Synemin
1907.	KRT19	Keratin 19

1908.	KCNE3	Potassium Voltage-Gated Channel Subfamily E Regulatory Subunit 3
1909.	PTH1R	Parathyroid Hormone 1 Receptor
1910.	KCNC3	Potassium Voltage-Gated Channel Subfamily C Member 3
1911.	MYH14	Myosin Heavy Chain 14
1912.	PNKD	PNKD Metallo-Beta-Lactamase Domain Containing
1913.	WNT5A	Wnt Family Member 5A
1914.	KDM1A	Lysine Demethylase 1A
1915.	SLC12A1	Solute Carrier Family 12 Member 1
1916.	FZD6	Frizzled Class Receptor 6
1917.	CCNA2	Cyclin A2
1918.	RNASEL	Ribonuclease L
1919.	SSTR3	Somatostatin Receptor 3
1920.	ATXN10	Ataxin 10
1921.	GPX7	Glutathione Peroxidase 7
1922.	GFI1	Growth Factor Independent 1 Transcriptional Repressor
1923.	GPX2	Glutathione Peroxidase 2
1924.	FIP1L1	Factor Interacting With PAPOLA And CPSF1
1925.	GPX5	Glutathione Peroxidase 5
1926.	GPX8	Glutathione Peroxidase 8 (Putative)
1927.	NANOG	Nanog Homeobox
1928.	RNPC3	RNA Binding Region (RNP1, RRM) Containing 3
1929.	GPX6	Glutathione Peroxidase 6
1930.	FGF10	Fibroblast Growth Factor 10
1931.	SERPINE2	Serpin Family E Member 2
1932.	GALC	Galactosylceramidase
1933.	KIDINS220	Kinase D Interacting Substrate 220
1934.	SPAG1	Sperm Associated Antigen 1
1935.	HYDIN	HYDIN Axonemal Central Pair Apparatus Protein
1936.	MCIDAS	Multiciliate Differentiation And DNA Synthesis Associated Cell Cycle Protein
1937.	TBL1X	Transducin Beta Like 1 X-Linked
1938.	ABCA4	ATP Binding Cassette Subfamily A Member 4
1939.	BTK	Bruton Tyrosine Kinase
1940.	AP5M1	Adaptor Related Protein Complex 5 Subunit Mu 1
1941.	AP3B1	Adaptor Related Protein Complex 3 Subunit Beta 1
1942.	GIPR	Gastric Inhibitory Polypeptide Receptor
1943.	FAF1	Fas Associated Factor 1
1944.	TRIM38	Tripartite Motif Containing 38
1945.	ITPK1	Inositol-Tetrakisphosphate 1-Kinase
1946.	MYL6	Myosin Light Chain 6
1947.	TG	Thyroglobulin
1948.	ITGA2B	Integrin Subunit Alpha 2b

1949.	CSNK2A1	Casein Kinase 2 Alpha 1
1950.	ADRB1	Adrenoceptor Beta 1
1951.	CUL3	Cullin 3
1952.	TPT1	Tumor Protein, Translationally-Controlled 1
1953.	TYRO3	TYRO3 Protein Tyrosine Kinase
1954.	XPO1	Exportin 1
1955.	BCL6	BCL6 Transcription Repressor
1956.	NME2	NME/NM23 Nucleoside Diphosphate Kinase 2
1957.	FCER2	Fc Epsilon Receptor II
1958.	TRIM25	Tripartite Motif Containing 25
1959.	APOD	Apolipoprotein D
1960.	DNAJC5	DnaJ Heat Shock Protein Family (Hsp40) Member C5
1961.	KPNA1	Karyopherin Subunit Alpha 1
1962.	NEO1	Neogenin 1
1963.	TPSAB1	Tryptase Alpha/Beta 1
1964.	PRDM2	PR/SET Domain 2
1965.	SLC12A9	Solute Carrier Family 12 Member 9
1966.	XRN1	5'-3' Exoribonuclease 1
1967.	DNMT3B	DNA Methyltransferase 3 Beta
1968.	UBA1	Ubiquitin Like Modifier Activating Enzyme 1
1969.	FGR	FGR Proto-Oncogene, Src Family Tyrosine Kinase
1970.	XRCC6	X-Ray Repair Cross Complementing 6
1971.	ADAM12	ADAM Metallopeptidase Domain 12
1972.	ATP2B2	ATPase Plasma Membrane Ca2+ Transporting 2
1973.	VTN	Vitronectin
1974.	NPHP1	Nephrocystin 1
1975.	PBRM1	Polybromo 1
1976.	OLR1	Oxidized Low Density Lipoprotein Receptor 1
1977.	TBX6	T-Box Transcription Factor 6
1978.	CFHR3	Complement Factor H Related 3
1979.	CYBRD1	Cytochrome B Reductase 1
1980.	CD177	CD177 Molecule
1981.	INSM1	INSM Transcriptional Repressor 1
1982.	MRGPRX2	MAS Related GPR Family Member X2
1983.	CALM1	Calmodulin 1
1984.	CAV3	Caveolin 3
1985.	PPT1	Palmitoyl-Protein Thioesterase 1
1986.	VPS13A	Vacuolar Protein Sorting 13 Homolog A
1987.	KCNV2	Potassium Voltage-Gated Channel Modifier Subfamily V Member 2
1988.	AHSG	Alpha 2-HS Glycoprotein
1989.	C8B	Complement C8 Beta Chain

1990.	EIF2AK3	Eukaryotic Translation Initiation Factor 2 Alpha Kinase 3
1991.	ITPR1	Inositol 1,4,5-Trisphosphate Receptor Type 1
1992.	DLG4	Discs Large MAGUK Scaffold Protein 4
1993.	CDK1	Cyclin Dependent Kinase 1
1994.	LOX	Lysyl Oxidase
1995.	BECN1	Beclin 1
1996.	KCNA4	Potassium Voltage-Gated Channel Subfamily A Member 4
1997.	AKT2	AKT Serine/Threonine Kinase 2
1998.	ADCY5	Adenylate Cyclase 5
1999.	HSPG2	Heparan Sulfate Proteoglycan 2
2000.	ATP2B3	ATPase Plasma Membrane Ca2+ Transporting 3
2001.	MSX1	Msh Homeobox 1
2002.	ATN1	Atrophin 1
2003.	DNTT	DNA Nucleotidylexotransferase
2004.	ARMC5	Armadillo Repeat Containing 5
2005.	ATP1B1	ATPase Na+/K+ Transporting Subunit Beta 1
2006.	CR2	Complement C3d Receptor 2
2007.	DHPS	Deoxyhypusine Synthase
2008.	FCN2	Ficolin 2
2009.	IL17F	Interleukin 17F
2010.	PLAA	Phospholipase A2 Activating Protein
2011.	PPIC	Peptidylprolyl Isomerase C
2012.	PIEZO1	Piezo Type Mechanosensitive Ion Channel Component 1 (Er Blood Group)
2013.	GYPE	Glycophorin E (MNS Blood Group)
2014.	HDAC4	Histone Deacetylase 4
2015.	PLK1	Polo Like Kinase 1
2016.	ITGB2	Integrin Subunit Beta 2
2017.	CASP7	Caspase 7
2018.	PC	Pyruvate Carboxylase
2019.	SCP2	Sterol Carrier Protein 2
2020.	CFLAR	CASP8 And FADD Like Apoptosis Regulator
2021.	CHRNE	Cholinergic Receptor Nicotinic Epsilon Subunit
2022.	STMN1	Stathmin 1
2023.	ACP3	Acid Phosphatase 3
2024.	KLC1	Kinesin Light Chain 1
2025.	KCNJ13	Potassium Inwardly Rectifying Channel Subfamily J Member 13
2026.	MPV17	Mitochondrial Inner Membrane Protein MPV17
2027.	G3BP1	G3BP Stress Granule Assembly Factor 1
2028.	LIN28A	Lin-28 Homolog A
2029.	PPY	Pancreatic Polypeptide
2030.	ANKRD49	Ankyrin Repeat Domain 49

2031.	TUBB	Tubulin Beta Class I
2032.	HNRNPA1	Heterogeneous Nuclear Ribonucleoprotein A1
2033.	PANK2	Pantothenate Kinase 2
2034.	DST	Dystonin
2035.	H3C1	H3 Clustered Histone 1
2036.	CASK	Calcium/Calmodulin Dependent Serine Protein Kinase
2037.	ABCC9	ATP Binding Cassette Subfamily C Member 9
2038.	MAD1L1	Mitotic Arrest Deficient 1 Like 1
2039.	EIF4A3	Eukaryotic Translation Initiation Factor 4A3
2040.	CHRM4	Cholinergic Receptor Muscarinic 4
2041.	MDK	Midkine
2042.	ATG13	Autophagy Related 13
2043.	BCAN	Brevican
2044.	LRRC32	Leucine Rich Repeat Containing 32
2045.	PLEKHA1	Pleckstrin Homology Domain Containing A1
2046.	ARHGEF11	Rho Guanine Nucleotide Exchange Factor 11
2047.	PRCC	Proline Rich Mitotic Checkpoint Control Factor
2048.	BAZ2A	Bromodomain Adjacent To Zinc Finger Domain 2A
2049.	CFDP1	Craniofacial Development Protein 1
2050.	CTDSP2	CTD Small Phosphatase 2
2051.	GATAD2A	GATA Zinc Finger Domain Containing 2A
2052.	HORMAD1	HORMA Domain Containing 1
2053.	PAN2	Poly(A) Specific Ribonuclease Subunit PAN2
2054.	ONECUT2	One Cut Homeobox 2
2055.	SNX8	Sorting Nexin 8
2056.	DCAF5	DDB1 And CUL4 Associated Factor 5
2057.	BTBD16	BTB Domain Containing 16
2058.	RDH16	Retinol Dehydrogenase 16
2059.	NOL4L	Nucleolar Protein 4 Like
2060.	CSTPP1	Centriolar Satellite-Associated Tubulin Polyglutamylase Complex Regulator 1
2061.	GPR149	G Protein-Coupled Receptor 149
2062.	HARBI1	Harbinger Transposase Derived 1
2063.	TSACC	TSSK6 Activating Cochaperone
2064.	EEF1AKMT3	EEF1A Lysine Methyltransferase 3
2065.	LINC02210-CRHR1	LINC02210-CRHR1 Readthrough
2066.	ENSG00000285130	Novel Protein
2067.	ENSG00000286192	Novel Protein
2068.	LOC124903828	Collagen Alpha-5(IV) Chain-Like
2069.	LOC124901819	Uncharacterized LOC124901819
2070.	DAPK1	Death Associated Protein Kinase 1
2071.	CA1	Carbonic Anhydrase 1

2072.	TP63	Tumor Protein P63
2073.	CA8	Carbonic Anhydrase 8
2074.	ABCB7	ATP Binding Cassette Subfamily B Member 7
2075.	CA12	Carbonic Anhydrase 12
2076.	CA14	Carbonic Anhydrase 14
2077.	TIA1	TIA1 Cytotoxic Granule Associated RNA Binding Protein
2078.	CA6	Carbonic Anhydrase 6
2079.	CA5B	Carbonic Anhydrase 5B
2080.	MAML2	Mastermind Like Transcriptional Coactivator 2
2081.	CA13	Carbonic Anhydrase 13
2082.	CA7	Carbonic Anhydrase 7
2083.	GRIA2	Glutamate Ionotropic Receptor AMPA Type Subunit 2
2084.	PIK3C3	Phosphatidylinositol 3-Kinase Catalytic Subunit Type 3
2085.	SPG11	SPG11 Vesicle Trafficking Associated, Spatacsin
2086.	PXDNL	Peroxidasin Like
2087.	CDKN1C	Cyclin Dependent Kinase Inhibitor 1C
2088.	NFIB	Nuclear Factor I B
2089.	CTR9	CTR9 Homolog, Paf1/RNA Polymerase II Complex Component
2090.	DDX10	DEAD-Box Helicase 10
2091.	SGSM1	Small G Protein Signaling Modulator 1
2092.	ZCCHC14	Zinc Finger CCHC-Type Containing 14
2093.	CIART	Circadian Associated Repressor Of Transcription
2094.	ALPP	Alkaline Phosphatase, Placental
2095.	CYP21A2	Cytochrome P450 Family 21 Subfamily A Member 2
2096.	CCL26	C-C Motif Chemokine Ligand 26
2097.	AP4M1	Adaptor Related Protein Complex 4 Subunit Mu 1
2098.	SH3TC2	SH3 Domain And Tetratricopeptide Repeats 2
2099.	AP4E1	Adaptor Related Protein Complex 4 Subunit Epsilon 1
2100.	GRID2	Glutamate Ionotropic Receptor Delta Type Subunit 2
2101.	SMAD9	SMAD Family Member 9
2102.	NLRP1	NLR Family Pyrin Domain Containing 1
2103.	UNC119	Unc-119 Lipid Binding Chaperone
2104.	MPP1	MAGUK P55 Scaffold Protein 1
2105.	CTSK	Cathepsin K
2106.	RAC1	Rac Family Small GTPase 1
2107.	SLC1A1	Solute Carrier Family 1 Member 1
2108.	AKR1B1	Aldo-Keto Reductase Family 1 Member B
2109.	DLX5	Distal-Less Homeobox 5
2110.	ETFA	Electron Transfer Flavoprotein Subunit Alpha
2111.	HMGA2	High Mobility Group AT-Hook 2
2112.	DMP1	Dentin Matrix Acidic Phosphoprotein 1

2113.	EPB41L3	Erythrocyte Membrane Protein Band 4.1 Like 3
2114.	SLC17A6	Solute Carrier Family 17 Member 6
2115.	IFI27	Interferon Alpha Inducible Protein 27
2116.	FAM131B	Family With Sequence Similarity 131 Member B
2117.	PARK7	Parkinsonism Associated Deglycase
2118.	AARS1	Alanyl-TRNA Synthetase 1
2119.	PLOD2	Procollagen-Lysine,2-Oxoglutarate 5-Dioxygenase 2
2120.	PRPH	Peripherin
2121.	RIPK1	Receptor Interacting Serine/Threonine Kinase 1
2122.	ATP7A	ATPase Copper Transporting Alpha
2123.	CDK9	Cyclin Dependent Kinase 9
2124.	KRT5	Keratin 5
2125.	DDX1	DEAD-Box Helicase 1
2126.	MAP4K2	Mitogen-Activated Protein Kinase Kinase Kinase Kinase 2
2127.	SELPLG	Selectin P Ligand
2128.	AAK1	AP2 Associated Kinase 1
2129.	IL16	Interleukin 16
2130.	LHX1	LIM Homeobox 1
2131.	SCRIB	Scribble Planar Cell Polarity Protein
2132.	NAIP	NLR Family Apoptosis Inhibitory Protein
2133.	FUBP1	Far Upstream Element Binding Protein 1
2134.	STRN4	Striatin 4
2135.	TET1	Tet Methylcytosine Dioxygenase 1
2136.	OLIG1	Oligodendrocyte Transcription Factor 1
2137.	COX18	Cytochrome C Oxidase Assembly Factor COX18
2138.	MOB4	MOB Family Member 4, Phocein
2139.	SIKE1	Suppressor Of IKBKE 1
2140.	STRIP1	Striatin Interacting Protein 1
2141.	STRIP2	Striatin Interacting Protein 2
2142.	RIPOR1	RHO Family Interacting Cell Polarization Regulator 1
2143.	HSD17B4	Hydroxysteroid 17-Beta Dehydrogenase 4
2144.	TPP1	Tripeptidyl Peptidase 1
2145.	AP4B1	Adaptor Related Protein Complex 4 Subunit Beta 1
2146.	ADCY10	Adenylate Cyclase 10
2147.	RUNX1	RUNX Family Transcription Factor 1
2148.	TRPC3	Transient Receptor Potential Cation Channel Subfamily C Member 3
2149.	UMPS	Uridine Monophosphate Synthetase
2150.	CD81	CD81 Molecule
2151.	ORC1	Origin Recognition Complex Subunit 1
2152.	PRDX6	Peroxiredoxin 6
2153.	CYP3A5	Cytochrome P450 Family 3 Subfamily A Member 5

2154.	IL10RA	Interleukin 10 Receptor Subunit Alpha
2155.	ABCC5	ATP Binding Cassette Subfamily C Member 5
2156.	ADCY9	Adenylate Cyclase 9
2157.	AGO2	Argonaute RISC Catalytic Component 2
2158.	AICDA	Activation Induced Cytidine Deaminase
2159.	ATP2B4	ATPase Plasma Membrane Ca2+ Transporting 4
2160.	FCGRT	Fc Gamma Receptor And Transporter
2161.	VNN1	Vanin 1
2162.	LILRB1	Leukocyte Immunoglobulin Like Receptor B1
2163.	DDX39B	DExD-Box Helicase 39B
2164.	LAIR1	Leukocyte Associated Immunoglobulin Like Receptor 1
2165.	TRPC1	Transient Receptor Potential Cation Channel Subfamily C Member 1
2166.	ADCY4	Adenylate Cyclase 4
2167.	GLRX5	Glutaredoxin 5
2168.	APEH	Acylaminoacyl-Peptide Hydrolase
2169.	HLA-DRB5	Major Histocompatibility Complex, Class II, DR Beta 5
2170.	LILRB2	Leukocyte Immunoglobulin Like Receptor B2
2171.	LTB	Lymphotoxin Beta
2172.	MAPK8IP3	Mitogen-Activated Protein Kinase 8 Interacting Protein 3
2173.	NCR3	Natural Cytotoxicity Triggering Receptor 3
2174.	RHCE	Rh Blood Group CcEe Antigens
2175.	MICA	MHC Class I Polypeptide-Related Sequence A
2176.	ABO	ABO, Alpha 1-3-N-Acetylgalactosaminyltransferase And Alpha 1-3-Galactosyltransferase
2177.	HM13	Histocompatibility Minor 13
2178.	SENP6	SUMO Specific Peptidase 6
2179.	TIMD4	T Cell Immunoglobulin And Mucin Domain Containing 4
2180.	KIR2DL3	Killer Cell Immunoglobulin Like Receptor, Two Ig Domains And Long Cytoplasmic Tail 3
2181.	SENP7	SUMO Specific Peptidase 7
2182.	TXNL1	Thioredoxin Like 1
2183.	FCRL4	Fc Receptor Like 4
2184.	ABRA	Actin Binding Rho Activating Protein
2185.	CCL3L1	C-C Motif Chemokine Ligand 3 Like 1
2186.	AURKB	Aurora Kinase B
2187.	EIF4E	Eukaryotic Translation Initiation Factor 4E
2188.	PIK3R2	Phosphoinositide-3-Kinase Regulatory Subunit 2
2189.	GLI3	GLI Family Zinc Finger 3
2190.	CAV1	Caveolin 1
2191.	LIG4	DNA Ligase 4
2192.	MAPK7	Mitogen-Activated Protein Kinase 7
2193.	PPM1D	Protein Phosphatase, Mg2+/Mn2+ Dependent 1D

2194.	WEE1	WEE1 G2 Checkpoint Kinase
2195.	CLDN1	Claudin 1
2196.	CTSA	Cathepsin A
2197.	IL7R	Interleukin 7 Receptor
2198.	CHRNA1	Cholinergic Receptor Nicotinic Alpha 1 Subunit
2199.	DGKE	Diacylglycerol Kinase Epsilon
2200.	MC2R	Melanocortin 2 Receptor
2201.	TAT	Tyrosine Aminotransferase
2202.	TGFA	Transforming Growth Factor Alpha
2203.	CUL1	Cullin 1
2204.	HES1	Hes Family BHLH Transcription Factor 1
2205.	MARS1	Methionyl-TRNA Synthetase 1
2206.	BAMBI	BMP And Activin Membrane Bound Inhibitor
2207.	DAXX	Death Domain Associated Protein
2208.	PTPN13	Protein Tyrosine Phosphatase Non-Receptor Type 13
2209.	VEGFB	Vascular Endothelial Growth Factor B
2210.	CHRND	Cholinergic Receptor Nicotinic Delta Subunit
2211.	GNPTAB	N-Acetylglucosamine-1-Phosphate Transferase Subunits Alpha And Beta
2212.	SOCS2	Suppressor Of Cytokine Signaling 2
2213.	BRPF1	Bromodomain And PHD Finger Containing 1
2214.	SPINT2	Serine Peptidase Inhibitor, Kunitz Type 2
2215.	TERF1	Telomeric Repeat Binding Factor 1
2216.	GLIPR1	GLI Pathogenesis Related 1
2217.	OTX1	Orthodenticle Homeobox 1
2218.	RND3	Rho Family GTPase 3
2219.	RNF220	Ring Finger Protein 220
2220.	CFHR5	Complement Factor H Related 5
2221.	IFITM2	Interferon Induced Transmembrane Protein 2
2222.	JKAMP	JNK1/MAPK8 Associated Membrane Protein
2223.	BEND2	BEN Domain Containing 2
2224.	CCDC40	Coiled-Coil Domain 40 Molecular Ruler Complex Subunit
2225.	RSPH4A	Radial Spoke Head Component 4A
2226.	ZIC2	Zic Family Member 2
2227.	NTNG1	Netrin G1
2228.	TTC21B	Tetratricopeptide Repeat Domain 21B
2229.	SMAD2	SMAD Family Member 2
2230.	BMP4	Bone Morphogenetic Protein 4
2231.	WNK1	WNK Lysine Deficient Protein Kinase 1
2232.	GFRA1	GDNF Family Receptor Alpha 1
2233.	STUB1	STIP1 Homology And U-Box Containing Protein 1
2234.	CLCN7	Chloride Voltage-Gated Channel 7

2235.	CNP	2',3'-Cyclic Nucleotide 3' Phosphodiesterase
2236.	WNK4	WNK Lysine Deficient Protein Kinase 4
2237.	GCM2	Glial Cells Missing Transcription Factor 2
2238.	MAP1B	Microtubule Associated Protein 1B
2239.	PFKM	Phosphofructokinase, Muscle
2240.	CCKAR	Cholecystokinin A Receptor
2241.	KCNH1	Potassium Voltage-Gated Channel Subfamily H Member 1
2242.	SLC22A6	Solute Carrier Family 22 Member 6
2243.	WNT2B	Wnt Family Member 2B
2244.	SETDB1	SET Domain Bifurcated Histone Lysine Methyltransferase 1
2245.	DDX20	DEAD-Box Helicase 20
2246.	EVI5	Ecotropic Viral Integration Site 5
2247.	NAV2	Neuron Navigator 2
2248.	SNX27	Sorting Nexin 27
2249.	CLXN	Calaxin
2250.	RER1	Retention In Endoplasmic Reticulum Sorting Receptor 1
2251.	C16orf95	Chromosome 16 Open Reading Frame 95
2252.	IMPDH2	Inosine Monophosphate Dehydrogenase 2
2253.	ALS2	Alsin Rho Guanine Nucleotide Exchange Factor ALS2
2254.	HARS1	Histidyl-TRNA Synthetase 1
2255.	PLEKHG5	Pleckstrin Homology And RhoGEF Domain Containing G5
2256.	DNAAF4	Dynein Axonemal Assembly Factor 4
2257.	DNAAF1	Dynein Axonemal Assembly Factor 1
2258.	RPE65	Retinoid Isomerohydrolase RPE65
2259.	UCP2	Uncoupling Protein 2
2260.	FLNC	Filamin C
2261.	TRPM4	Transient Receptor Potential Cation Channel Subfamily M Member 4
2262.	RARS1	Arginyl-TRNA Synthetase 1
2263.	BBS1	Bardet-Biedl Syndrome 1
2264.	PSAP	Prosaposin
2265.	THRB	Thyroid Hormone Receptor Beta
2266.	WDR73	WD Repeat Domain 73
2267.	FGFR4	Fibroblast Growth Factor Receptor 4
2268.	PTPN6	Protein Tyrosine Phosphatase Non-Receptor Type 6
2269.	SMARCA2	SWI/SNF Related, Matrix Associated, Actin Dependent Regulator Of Chromatin, Subfamily A, Member 2
2270.	NFATC1	Nuclear Factor Of Activated T Cells 1
2271.	SPTBN1	Spectrin Beta, Non-Erythrocytic 1
2272.	ZIC3	Zic Family Member 3
2273.	CX3CL1	C-X3-C Motif Chemokine Ligand 1
2274.	LFNG	LFNG O-Fucosylpeptide 3-Beta-N-Acetylglucosaminyltransferase
2275.	SNAI1	Snail Family Transcriptional Repressor 1

2276.	ACADS	Acyl-CoA Dehydrogenase Short Chain
2277.	TNXB	Tenascin XB
2278.	ACAD8	Acyl-CoA Dehydrogenase Family Member 8
2279.	GJB6	Gap Junction Protein Beta 6
2280.	TNFRSF12A	TNF Receptor Superfamily Member 12A
2281.	DNALI1	Dynein Axonemal Light Intermediate Chain 1
2282.	HIF3A	Hypoxia Inducible Factor 3 Subunit Alpha
2283.	MYH1	Myosin Heavy Chain 1
2284.	PEX16	Peroxisomal Biogenesis Factor 16
2285.	NDRG4	NDRG Family Member 4
2286.	SAA2	Serum Amyloid A2
2287.	MESP2	Mesoderm Posterior BHLH Transcription Factor 2
2288.	KRT75	Keratin 75
2289.	FAM118B	Family With Sequence Similarity 118 Member B
2290.	EFNB2	Ephrin B2
2291.	MGP	Matrix Gla Protein
2292.	GPLD1	Glycosylphosphatidylinositol Specific Phospholipase D1
2293.	SHOC2	SHOC2 Leucine Rich Repeat Scaffold Protein
2294.	BMP10	Bone Morphogenetic Protein 10
2295.	MRS2	Magnesium Transporter MRS2
2296.	OR2AG1	Olfactory Receptor Family 2 Subfamily AG Member 1
2297.	UTS2R	Urotensin 2 Receptor
2298.	RXRB	Retinoid X Receptor Beta
2299.	ADAM10	ADAM Metallopeptidase Domain 10
2300.	MCL1	MCL1 Apoptosis Regulator, BCL2 Family Member
2301.	CADM1	Cell Adhesion Molecule 1
2302.	COX20	Cytochrome C Oxidase Assembly Factor COX20
2303.	FGF13	Fibroblast Growth Factor 13
2304.	ZNF197	Zinc Finger Protein 197
2305.	SLC2A10	Solute Carrier Family 2 Member 10
2306.	CGB7	Chorionic Gonadotropin Subunit Beta 7
2307.	ATP1A1	ATPase Na+/K+ Transporting Subunit Alpha 1
2308.	SGCE	Sarcoglycan Epsilon
2309.	INA	Internexin Neuronal Intermediate Filament Protein Alpha
2310.	ENPP1	Ectonucleotide Pyrophosphatase/Phosphodiesterase 1
2311.	SLC19A1	Solute Carrier Family 19 Member 1
2312.	ALAS2	5'-Aminolevulinate Synthase 2
2313.	IKBKE	Inhibitor Of Nuclear Factor Kappa B Kinase Subunit Epsilon
2314.	MAP3K3	Mitogen-Activated Protein Kinase Kinase Kinase 3
2315.	ATP6V1A	ATPase H+ Transporting V1 Subunit A
2316.	KPNA2	Karyopherin Subunit Alpha 2

2317.	CASQ1	Calsequestrin 1
2318.	SLC25A15	Solute Carrier Family 25 Member 15
2319.	INHBB	Inhibin Subunit Beta B
2320.	IQCB1	IQ Motif Containing B1
2321.	NBEAL2	Neurobeachin Like 2
2322.	NPHP4	Nephrocystin 4
2323.	GATB	Glutamyl-TRNA Amidotransferase Subunit B
2324.	IFT172	Intraflagellar Transport 172
2325.	SOX18	SRY-Box Transcription Factor 18
2326.	F13B	Coagulation Factor XIII B Chain
2327.	NPHP3	Nephrocystin 3
2328.	OTULIN	OTU Deubiquitinase With Linear Linkage Specificity
2329.	RIT2	Ras Like Without CAAX 2
2330.	CDAN1	Codanin 1
2331.	STIM2	Stromal Interaction Molecule 2
2332.	ORAI2	ORAI Calcium Release-Activated Calcium Modulator 2
2333.	WDR81	WD Repeat Domain 81
2334.	NOSTRIN	Nitric Oxide Synthase Trafficking
2335.	PRIMPOL	Primase And DNA Directed Polymerase
2336.	SLFN14	Schlafen Family Member 14
2337.	ORAI3	ORAI Calcium Release-Activated Calcium Modulator 3
2338.	SARAF	Store-Operated Calcium Entry Associated Regulatory Factor
2339.	CRACR2A	Calcium Release Activated Channel Regulator 2A
2340.	STH	Saitohin
2341.	ITK	IL2 Inducible T Cell Kinase
2342.	USP7	Ubiquitin Specific Peptidase 7
2343.	KDM6A	Lysine Demethylase 6A
2344.	TGM2	Transglutaminase 2
2345.	ARSB	Arylsulfatase B
2346.	BRD4	Bromodomain Containing 4
2347.	DPP6	Dipeptidyl Peptidase Like 6
2348.	FHL1	Four And A Half LIM Domains 1
2349.	KDM4B	Lysine Demethylase 4B
2350.	NEK9	NIMA Related Kinase 9
2351.	NT5C2	5'-Nucleotidase, Cytosolic II
2352.	SUCLA2	Succinate-CoA Ligase ADP-Forming Subunit Beta
2353.	HELLS	Helicase, Lymphoid Specific
2354.	HPSE	Heparanase
2355.	MAP2K5	Mitogen-Activated Protein Kinase Kinase 5
2356.	PRMT5	Protein Arginine Methyltransferase 5
2357.	S100A4	S100 Calcium Binding Protein A4

2358.	KMT2C	Lysine Methyltransferase 2C
2359.	LTBP2	Latent Transforming Growth Factor Beta Binding Protein 2
2360.	SOX4	SRY-Box Transcription Factor 4
2361.	KAT8	Lysine Acetyltransferase 8
2362.	KCNH5	Potassium Voltage-Gated Channel Subfamily H Member 5
2363.	MACROH2A1	MacroH2A.1 Histone
2364.	TK2	Thymidine Kinase 2
2365.	CELSR2	Cadherin EGF LAG Seven-Pass G-Type Receptor 2
2366.	HIC1	HIC ZBTB Transcriptional Repressor 1
2367.	LASP1	LIM And SH3 Protein 1
2368.	PLAGL1	PLAG1 Like Zinc Finger 1
2369.	PODXL	Podocalyxin Like
2370.	SERPINA7	Serpin Family A Member 7
2371.	HHIP	Hedgehog Interacting Protein
2372.	KIF14	Kinesin Family Member 14
2373.	SOX11	SRY-Box Transcription Factor 11
2374.	DAB2IP	DAB2 Interacting Protein
2375.	GFI1B	Growth Factor Independent 1B Transcriptional Repressor
2376.	PSMC6	Proteasome 26S Subunit, ATPase 6
2377.	RCVRN	Recoverin
2378.	UHRF1	Ubiquitin Like With PHD And Ring Finger Domains 1
2379.	BTG2	BTG Anti-Proliferation Factor 2
2380.	FUT4	Fucosyltransferase 4
2381.	KLRG1	Killer Cell Lectin Like Receptor G1
2382.	PCGF2	Polycomb Group Ring Finger 2
2383.	ZC3H12A	Zinc Finger CCCH-Type Containing 12A
2384.	SCUBE1	Signal Peptide, CUB Domain And EGF Like Domain Containing 1
2385.	NEURL1	Neuralized E3 Ubiquitin Protein Ligase 1
2386.	ASZ1	Ankyrin Repeat, SAM And Basic Leucine Zipper Domain Containing 1
2387.	BARHL1	BarH Like Homeobox 1
2388.	CTDNEP1	CTD Nuclear Envelope Phosphatase 1
2389.	FBXL17	F-Box And Leucine Rich Repeat Protein 17
2390.	FSTL5	Follistatin Like 5
2391.	L3MBTL3	L3MBTL Histone Methyl-Lysine Binding Protein 3
2392.	SPTBN5	Spectrin Beta, Non-Erythrocytic 5
2393.	TCFL5	Transcription Factor Like 5
2394.	KCTD6	Potassium Channel Tetramerization Domain Containing 6
2395.	SRRM4	Serine/Arginine Repetitive Matrix 4
2396.	KCTD11	Potassium Channel Tetramerization Domain Containing 11
2397.	MXD3	MAX Dimerization Protein 3
2398.	S100A5	S100 Calcium Binding Protein A5

2399.	KCTD21	Potassium Channel Tetramerization Domain Containing 21
2400.	MLC1	Modulator Of VRAC Current 1
2401.	ETHE1	ETHE1 Persulfide Dioxygenase
2402.	MCPH1	Microcephalin 1
2403.	BMP6	Bone Morphogenetic Protein 6
2404.	GSTT1	Glutathione S-Transferase Theta 1
2405.	POLR2A	RNA Polymerase II Subunit A
2406.	AOX1	Aldehyde Oxidase 1
2407.	CAMK2B	Calcium/Calmodulin Dependent Protein Kinase II Beta
2408.	SMC1A	Structural Maintenance Of Chromosomes 1A
2409.	IGHMBP2	Immunoglobulin Mu DNA Binding Protein 2
2410.	CAT	Catalase
2411.	MMP14	Matrix Metallopeptidase 14
2412.	CES1	Carboxylesterase 1
2413.	MAP2K4	Mitogen-Activated Protein Kinase Kinase 4
2414.	HCFC1	Host Cell Factor C1
2415.	KCNB1	Potassium Voltage-Gated Channel Subfamily B Member 1
2416.	PYGM	Glycogen Phosphorylase, Muscle Associated
2417.	DOCK8	Dedicator Of Cytokinesis 8
2418.	IREB2	Iron Responsive Element Binding Protein 2
2419.	LNPEP	Leucyl And Cystinyl Aminopeptidase
2420.	SI	Sucrase-Isomaltase
2421.	SPHK1	Sphingosine Kinase 1
2422.	C5	Complement C5
2423.	CXADR	CXADR Ig-Like Cell Adhesion Molecule
2424.	DROSHA	Drosha Ribonuclease III
2425.	COL11A2	Collagen Type XI Alpha 2 Chain
2426.	MGAM	Maltase-Glucoamylase
2427.	NDUFA9	NADH:Ubiquinone Oxidoreductase Subunit A9
2428.	OCLN	Occludin
2429.	EEA1	Early Endosome Antigen 1
2430.	MARS2	Methionyl-TRNA Synthetase 2, Mitochondrial
2431.	MYO7A	Myosin VIIA
2432.	KLRK1	Killer Cell Lectin Like Receptor K1
2433.	PTN	Pleiotrophin
2434.	SLC13A3	Solute Carrier Family 13 Member 3
2435.	AMY2B	Amylase Alpha 2B
2436.	OMG	Oligodendrocyte Myelin Glycoprotein
2437.	POLR2L	RNA Polymerase II, I And III Subunit L
2438.	STMN2	Stathmin 2
2439.	AMY2A	Amylase Alpha 2A

2440.	ENAM	Enamelin
2441.	IBSP	Integrin Binding Sialoprotein
2442.	MEPE	Matrix Extracellular Phosphoglycoprotein
2443.	UGT3A1	UDP Glycosyltransferase Family 3 Member A1
2444.	UGT3A2	UDP Glycosyltransferase Family 3 Member A2
2445.	DENND2B	DENN Domain Containing 2B
2446.	PMCH	Pro-Melanin Concentrating Hormone
2447.	AMY1B	Amylase Alpha 1B
2448.	RFC1	Replication Factor C Subunit 1
2449.	FIG4	FIG4 Phosphoinositide 5-Phosphatase
2450.	BMPR2	Bone Morphogenetic Protein Receptor Type 2
2451.	IL10RB	Interleukin 10 Receptor Subunit Beta
2452.	PTPRQ	Protein Tyrosine Phosphatase Receptor Type Q
2453.	PDPK1	3-Phosphoinositide Dependent Protein Kinase 1
2454.	AHR	Aryl Hydrocarbon Receptor
2455.	NAGLU	N-Acetyl-Alpha-Glucosaminidase
2456.	SMPD1	Sphingomyelin Phosphodiesterase 1
2457.	NRG1	Neuregulin 1
2458.	SCNN1B	Sodium Channel Epithelial 1 Subunit Beta
2459.	CCND3	Cyclin D3
2460.	PDE6B	Phosphodiesterase 6B
2461.	WNT3A	Wnt Family Member 3A
2462.	DIABLO	Diablo IAP-Binding Mitochondrial Protein
2463.	EIF4EBP1	Eukaryotic Translation Initiation Factor 4E Binding Protein 1
2464.	PLCB4	Phospholipase C Beta 4
2465.	XRCC1	X-Ray Repair Cross Complementing 1
2466.	DNASE1	Deoxyribonuclease 1
2467.	FOXP2	Forkhead Box P2
2468.	HMGCL	3-Hydroxy-3-Methylglutaryl-CoA Lyase
2469.	TRPV5	Transient Receptor Potential Cation Channel Subfamily V Member 5
2470.	ASCL1	Achaete-Scute Family BHLH Transcription Factor 1
2471.	NTSR1	Neurotensin Receptor 1
2472.	PCSK5	Proprotein Convertase Subtilisin/Kexin Type 5
2473.	NUDT6	Nudix Hydrolase 6
2474.	FUNDC2	FUN14 Domain Containing 2
2475.	FGF8	Fibroblast Growth Factor 8
2476.	FGF4	Fibroblast Growth Factor 4
2477.	GARS1	Glycyl-TRNA Synthetase 1
2478.	SNCB	Synuclein Beta
2479.	CNTNAP1	Contactin Associated Protein 1
2480.	DNAH11	Dynein Axonemal Heavy Chain 11

2481.	DNAI2	Dynein Axonemal Intermediate Chain 2
2482.	RSPH1	Radial Spoke Head Component 1
2483.	RSPH9	Radial Spoke Head Component 9
2484.	HDC	Histidine Decarboxylase
2485.	DBT	Dihydrolipoamide Branched Chain Transacylase E2
2486.	ADH1C	Alcohol Dehydrogenase 1C (Class I), Gamma Polypeptide
2487.	AMPD2	Adenosine Monophosphate Deaminase 2
2488.	ERLIN2	ER Lipid Raft Associated 2
2489.	TBX4	T-Box Transcription Factor 4
2490.	TMX2	Thioredoxin Related Transmembrane Protein 2
2491.	ARV1	ARV1 Homolog, Fatty Acid Homeostasis Modulator
2492.	GP6	Glycoprotein VI Platelet
2493.	SLC9A6	Solute Carrier Family 9 Member A6
2494.	PON2	Paraoxonase 2
2495.	CHRNG	Cholinergic Receptor Nicotinic Gamma Subunit
2496.	KIF21A	Kinesin Family Member 21A
2497.	RAB33A	RAB33A, Member RAS Oncogene Family
2498.	SCN2B	Sodium Voltage-Gated Channel Beta Subunit 2
2499.	KCNE1	Potassium Voltage-Gated Channel Subfamily E Regulatory Subunit 1
2500.	SCN3B	Sodium Voltage-Gated Channel Beta Subunit 3
2501.	SCN4B	Sodium Voltage-Gated Channel Beta Subunit 4
2502.	KCNJ3	Potassium Inwardly Rectifying Channel Subfamily J Member 3
2503.	L2HGDH	L-2-Hydroxyglutarate Dehydrogenase
2504.	NOTCH2NLC	Notch 2 N-Terminal Like C
2505.	NR1H2	Nuclear Receptor Subfamily 1 Group H Member 2
2506.	NR0B1	Nuclear Receptor Subfamily 0 Group B Member 1
2507.	PLA2G7	Phospholipase A2 Group VII
2508.	BMPR1B	Bone Morphogenetic Protein Receptor Type 1B
2509.	FOLR1	Folate Receptor Alpha
2510.	MAP2K3	Mitogen-Activated Protein Kinase Kinase 3
2511.	ARG2	Arginase 2
2512.	PHB1	Prohibitin 1
2513.	PYGL	Glycogen Phosphorylase L
2514.	QDPR	Quinoid Dihydropteridine Reductase
2515.	RPS6KB2	Ribosomal Protein S6 Kinase B2
2516.	SERPINF2	Serpin Family F Member 2
2517.	TIE1	Tyrosine Kinase With Immunoglobulin Like And EGF Like Domains 1
2518.	EIF2AK4	Eukaryotic Translation Initiation Factor 2 Alpha Kinase 4
2519.	HBEGF	Heparin Binding EGF Like Growth Factor
2520.	PSMA7	Proteasome 20S Subunit Alpha 7
2521.	SFTPD	Surfactant Protein D

2522.	ADGRE5	Adhesion G Protein-Coupled Receptor E5
2523.	LRP8	LDL Receptor Related Protein 8
2524.	PSMD2	Proteasome 26S Subunit Ubiquitin Receptor, Non-ATPase 2
2525.	RAD51C	RAD51 Paralog C
2526.	FANCM	FA Complementation Group M
2527.	PYGB	Glycogen Phosphorylase B
2528.	RAP1B	RAP1B, Member Of RAS Oncogene Family
2529.	SLC18A1	Solute Carrier Family 18 Member A1
2530.	IL13RA1	Interleukin 13 Receptor Subunit Alpha 1
2531.	LPA	Lipoprotein(A)
2532.	SH3KBP1	SH3 Domain Containing Kinase Binding Protein 1
2533.	SLC30A10	Solute Carrier Family 30 Member 10
2534.	STC1	Stanniocalcin 1
2535.	MLPH	Melanophilin
2536.	DHX58	DExH-Box Helicase 58
2537.	HBD	Hemoglobin Subunit Delta
2538.	MAML1	Mastermind Like Transcriptional Coactivator 1
2539.	KLF2	KLF Transcription Factor 2
2540.	KPNA4	Karyopherin Subunit Alpha 4
2541.	MAML3	Mastermind Like Transcriptional Coactivator 3
2542.	MAZ	MYC Associated Zinc Finger Protein
2543.	NAP1L4	Nucleosome Assembly Protein 1 Like 4
2544.	RAP2A	RAP2A, Member Of RAS Oncogene Family
2545.	WDR1	WD Repeat Domain 1
2546.	EEF1G	Eukaryotic Translation Elongation Factor 1 Gamma
2547.	HSPA14	Heat Shock Protein Family A (Hsp70) Member 14
2548.	ITGB1BP1	Integrin Subunit Beta 1 Binding Protein 1
2549.	NAP1L1	Nucleosome Assembly Protein 1 Like 1
2550.	NAV1	Neuron Navigator 1
2551.	RASIP1	Ras Interacting Protein 1
2552.	ZDHHC19	Zinc Finger DHHC-Type Palmitoyltransferase 19
2553.	G3BP2	G3BP Stress Granule Assembly Factor 2
2554.	ZDHHC2	Zinc Finger DHHC-Type Palmitoyltransferase 2
2555.	HEG1	Heart Development Protein With EGF Like Domains 1
2556.	KRI1	KRI1 Homolog
2557.	ZRSR2	Zinc Finger CCCH-Type, RNA Binding Motif And Serine/Arginine Rich 2
2558.	CCDC83	Coiled-Coil Domain Containing 83
2559.	H3C3	H3 Clustered Histone 3
2560.	ZPLD1	Zona Pellucida Like Domain Containing 1
2561.	NACAD	NAC Alpha Domain Containing
2562.	FHIP1B	FHF Complex Subunit HOOK Interacting Protein 1B

2563.	HIGD1C	HIG1 Hypoxia Inducible Domain Family Member 1C
2564.	CCDC177	Coiled-Coil Domain Containing 177
2565.	TRB	T Cell Receptor Beta Locus
2566.	JUP	Junction Plakoglobin
2567.	TOM1	Target Of Myb1 Membrane Trafficking Protein
2568.	MNX1	Motor Neuron And Pancreas Homeobox 1
2569.	ASXL3	ASXL Transcriptional Regulator 3
2570.	CHRNA4	Cholinergic Receptor Nicotinic Alpha 4 Subunit
2571.	RHEB	Ras Homolog, MTORC1 Binding
2572.	EIF4G1	Eukaryotic Translation Initiation Factor 4 Gamma 1
2573.	SCNN1G	Sodium Channel Epithelial 1 Subunit Gamma
2574.	PCCB	Propionyl-CoA Carboxylase Subunit Beta
2575.	SIGMAR1	Sigma Non-Opioid Intracellular Receptor 1
2576.	TNFRSF13B	TNF Receptor Superfamily Member 13B
2577.	ISL1	ISL LIM Homeobox 1
2578.	PABPN1	Poly(A) Binding Protein Nuclear 1
2579.	PPP2R2B	Protein Phosphatase 2 Regulatory Subunit Bbeta
2580.	PEX19	Peroxisomal Biogenesis Factor 19
2581.	CD1D	CD1d Molecule
2582.	RICTOR	RPTOR Independent Companion Of MTOR Complex 2
2583.	LHX4	LIM Homeobox 4
2584.	MAP1LC3B	Microtubule Associated Protein 1 Light Chain 3 Beta
2585.	PTF1A	Pancreas Associated Transcription Factor 1a
2586.	DNAJC12	DnaJ Heat Shock Protein Family (Hsp40) Member C12
2587.	H3-4	H3.4 Histone, Cluster Member
2588.	HESX1	HESX Homeobox 1
2589.	UBR4	Ubiquitin Protein Ligase E3 Component N-Recognin 4
2590.	CD99L2	CD99 Molecule Like 2
2591.	H3C13	H3 Clustered Histone 13
2592.	H3-5	H3.5 Histone
2593.	H3-7	H3.7 Histone (Putative)
2594.	KCNQ4	Potassium Voltage-Gated Channel Subfamily Q Member 4
2595.	MYLK	Myosin Light Chain Kinase
2596.	KCNK3	Potassium Two Pore Domain Channel Subfamily K Member 3
2597.	ADSL	Adenylosuccinate Lyase
2598.	C1S	Complement C1s
2599.	CCR4	C-C Motif Chemokine Receptor 4
2600.	GDF5	Growth Differentiation Factor 5
2601.	GRIN2C	Glutamate Ionotropic Receptor NMDA Type Subunit 2C
2602.	BCAT1	Branched Chain Amino Acid Transaminase 1
2603.	ITGA1	Integrin Subunit Alpha 1

2604.	KLF6	KLF Transcription Factor 6
2605.	LMX1B	LIM Homeobox Transcription Factor 1 Beta
2606.	SERPINB2	Serpin Family B Member 2
2607.	SLC1A6	Solute Carrier Family 1 Member 6
2608.	ANP32A	Acidic Nuclear Phosphoprotein 32 Family Member A
2609.	EFEMP1	EGF Containing Fibulin Extracellular Matrix Protein 1
2610.	FSCN1	Fascin Actin-Bundling Protein 1
2611.	LTBP3	Latent Transforming Growth Factor Beta Binding Protein 3
2612.	PHF21A	PHD Finger Protein 21A
2613.	PNPLA6	Patatin Like Phospholipase Domain Containing 6
2614.	POLI	DNA Polymerase Iota
2615.	ZP3	Zona Pellucida Glycoprotein 3
2616.	COL9A1	Collagen Type IX Alpha 1 Chain
2617.	HCRTR1	Hypocretin Receptor 1
2618.	PABPC1	Poly(A) Binding Protein Cytoplasmic 1
2619.	SEC24D	SEC24 Homolog D, COPII Coat Complex Component
2620.	ATOX1	Antioxidant 1 Copper Chaperone
2621.	PALLD	Palladin, Cytoskeletal Associated Protein
2622.	SUMF1	Sulfatase Modifying Factor 1
2623.	CELSR1	Cadherin EGF LAG Seven-Pass G-Type Receptor 1
2624.	NEK8	NIMA Related Kinase 8
2625.	PACRG	Parkin Coregulated
2626.	POU4F1	POU Class 4 Homeobox 1
2627.	CPSF4	Cleavage And Polyadenylation Specific Factor 4
2628.	TSLP	Thymic Stromal Lymphopoietin
2629.	ANP32B	Acidic Nuclear Phosphoprotein 32 Family Member B
2630.	CRPPA	CDP-L-Ribitol Pyrophosphorylase A
2631.	DCAF1	DDB1 And CUL4 Associated Factor 1
2632.	KANK2	KN Motif And Ankyrin Repeat Domains 2
2633.	RHOD	Ras Homolog Family Member D
2634.	NRIP2	Nuclear Receptor Interacting Protein 2
2635.	OMP	Olfactory Marker Protein
2636.	BAHD1	Bromo Adjacent Homology Domain Containing 1
2637.	SUSD5	Sushi Domain Containing 5
2638.	ALX3	ALX Homeobox 3
2639.	CGB3	Chorionic Gonadotropin Subunit Beta 3
2640.	CNIH1	Cornichon Family AMPA Receptor Auxiliary Protein 1
2641.	IFITM10	Interferon Induced Transmembrane Protein 10
2642.	FLT4	Fms Related Receptor Tyrosine Kinase 4
2643.	CBL	Cbl Proto-Oncogene
2644.	ANPEP	Alanyl Aminopeptidase, Membrane

2645.	NR2F2	Nuclear Receptor Subfamily 2 Group F Member 2
2646.	TPO	Thyroid Peroxidase
2647.	SMAD6	SMAD Family Member 6
2648.	OCRL	OCRL Inositol Polyphosphate-5-Phosphatase
2649.	VAV1	Vav Guanine Nucleotide Exchange Factor 1
2650.	TRIM21	Tripartite Motif Containing 21
2651.	SERPINB6	Serpin Family B Member 6
2652.	SMAD5	SMAD Family Member 5
2653.	ADGRV1	Adhesion G Protein-Coupled Receptor V1
2654.	EXOSC5	Exosome Component 5
2655.	ID1	Inhibitor Of DNA Binding 1
2656.	FANCB	FA Complementation Group B
2657.	DYNLT1	Dynein Light Chain Tctex-Type 1
2658.	ETF1	Eukaryotic Translation Termination Factor 1
2659.	APBB1IP	Amyloid Beta Precursor Protein Binding Family B Member 1 Interacting Protein
2660.	DNASE1L1	Deoxyribonuclease 1 Like 1
2661.	TBC1D5	TBC1 Domain Family Member 5
2662.	CDK2AP1	Cyclin Dependent Kinase 2 Associated Protein 1
2663.	RABIF	RAB Interacting Factor
2664.	OAZ3	Ornithine Decarboxylase Antizyme 3
2665.	CPN1	Carboxypeptidase N Subunit 1
2666.	CYP2U1	Cytochrome P450 Family 2 Subfamily U Member 1
2667.	MFSD8	Major Facilitator Superfamily Domain Containing 8
2668.	BBS12	Bardet-Biedl Syndrome 12
2669.	SPR	Sepiapterin Reductase
2670.	MSTN	Myostatin
2671.	EGR2	Early Growth Response 2
2672.	GNAL	G Protein Subunit Alpha L
2673.	PMP2	Peripheral Myelin Protein 2
2674.	PEX6	Peroxisomal Biogenesis Factor 6
2675.	ANO3	Anoctamin 3
2676.	NIPA1	NIPA Magnesium Transporter 1
2677.	CCDC103	Coiled-Coil Domain Containing 103
2678.	GABBR1	Gamma-Aminobutyric Acid Type B Receptor Subunit 1
2679.	PRX	Periaxin
2680.	DNAJC13	DnaJ Heat Shock Protein Family (Hsp40) Member C13
2681.	KMT2B	Lysine Methyltransferase 2B
2682.	DNAAF11	Dynein Axonemal Assembly Factor 11
2683.	APEX1	Apurinic/Apyrimidinic Endodeoxyribonuclease 1
2684.	RBCK1	RANBP2-Type And C3HC4-Type Zinc Finger Containing 1
2685.	PCSK1N	Proprotein Convertase Subtilisin/Kexin Type 1 Inhibitor

2686.	PPARD	Peroxisome Proliferator Activated Receptor Delta
2687.	ATP13A2	ATPase Cation Transporting 13A2
2688.	FGF3	Fibroblast Growth Factor 3
2689.	FGF7	Fibroblast Growth Factor 7
2690.	SLPI	Secretory Leukocyte Peptidase Inhibitor
2691.	PRKCG	Protein Kinase C Gamma
2692.	GYS1	Glycogen Synthase 1
2693.	CCNE1	Cyclin E1
2694.	NRXN1	Neurexin 1
2695.	TMPO	Thymopoietin
2696.	WNT10B	Wnt Family Member 10B
2697.	CANX	Calnexin
2698.	GPNMB	Glycoprotein Nmb
2699.	KCNC1	Potassium Voltage-Gated Channel Subfamily C Member 1
2700.	PDE8B	Phosphodiesterase 8B
2701.	PSMA3	Proteasome 20S Subunit Alpha 3
2702.	ABCC4	ATP Binding Cassette Subfamily C Member 4 (PEL Blood Group)
2703.	CLN3	CLN3 Lysosomal/Endosomal Transmembrane Protein, Battenin
2704.	COL17A1	Collagen Type XVII Alpha 1 Chain
2705.	EPRS1	Glutamyl-Prolyl-TRNA Synthetase 1
2706.	ITM2B	Integral Membrane Protein 2B
2707.	PDE11A	Phosphodiesterase 11A
2708.	RB1CC1	RB1 Inducible Coiled-Coil 1
2709.	SIRPA	Signal Regulatory Protein Alpha
2710.	TRPM6	Transient Receptor Potential Cation Channel Subfamily M Member 6
2711.	ARL3	ADP Ribosylation Factor Like GTPase 3
2712.	GRPR	Gastrin Releasing Peptide Receptor
2713.	HLCS	Holocarboxylase Synthetase
2714.	LGALS1	Galectin 1
2715.	NEFM	Neurofilament Medium Chain
2716.	NTHL1	Nth Like DNA Glycosylase 1
2717.	PHOX2A	Paired Like Homeobox 2A
2718.	SFRP4	Secreted Frizzled Related Protein 4
2719.	BPI	Bactericidal Permeability Increasing Protein
2720.	CLN5	CLN5 Intracellular Trafficking Protein
2721.	SHANK3	SH3 And Multiple Ankyrin Repeat Domains 3
2722.	SLC25A6	Solute Carrier Family 25 Member 6
2723.	TSHB	Thyroid Stimulating Hormone Subunit Beta
2724.	FKBP14	FKBP Prolyl Isomerase 14
2725.	GBA2	Glucosylceramidase Beta 2
2726.	KCNAB2	Potassium Voltage-Gated Channel Subfamily A Regulatory Beta Subunit 2

2727.	HIF1AN	Hypoxia Inducible Factor 1 Subunit Alpha Inhibitor
2728.	KCNAB1	Potassium Voltage-Gated Channel Subfamily A Regulatory Beta Subunit 1
2729.	PSIP1	PC4 And SRSF1 Interacting Protein 1
2730.	TTF2	Transcription Termination Factor 2
2731.	WNT16	Wnt Family Member 16
2732.	CD1B	CD1b Molecule
2733.	KCNA10	Potassium Voltage-Gated Channel Subfamily A Member 10
2734.	CD1C	CD1c Molecule
2735.	CD1E	CD1e Molecule
2736.	DUSP19	Dual Specificity Phosphatase 19
2737.	PIGW	Phosphatidylinositol Glycan Anchor Biosynthesis Class W
2738.	DTWD2	DTW Domain Containing 2
2739.	BICRA	BRD4 Interacting Chromatin Remodeling Complex Associated Protein
2740.	NTS	Neurotensin
2741.	LBR	Lamin B Receptor
2742.	SEMA3A	Semaphorin 3A
2743.	SH2D1A	SH2 Domain Containing 1A
2744.	GJA5	Gap Junction Protein Alpha 5
2745.	STXBP2	Syntaxin Binding Protein 2
2746.	TLL1	Tolloid Like 1
2747.	STX11	Syntaxin 11
2748.	RLN2	Relaxin 2
2749.	FGF18	Fibroblast Growth Factor 18
2750.	FGF20	Fibroblast Growth Factor 20
2751.	HPCAL4	Hippocalcin Like 4
2752.	NKX2-6	NK2 Homeobox 6
2753.	IKZF1	IKAROS Family Zinc Finger 1
2754.	VRK1	VRK Serine/Threonine Kinase 1
2755.	SERPINH1	Serpin Family H Member 1
2756.	SERPINF1	Serpin Family F Member 1
2757.	USF3	Upstream Transcription Factor Family Member 3
2758.	GRIA3	Glutamate Ionotropic Receptor AMPA Type Subunit 3
2759.	DNM2	Dynamin 2
2760.	ATP7B	ATPase Copper Transporting Beta
2761.	SLC2A3	Solute Carrier Family 2 Member 3
2762.	PRKAR1B	Protein Kinase CAMP-Dependent Type I Regulatory Subunit Beta
2763.	ANO1	Anoctamin 1
2764.	LITAF	Lipopolysaccharide Induced TNF Factor
2765.	GAN	Gigaxonin
2766.	SBF2	SET Binding Factor 2
2767.	VPS13C	Vacuolar Protein Sorting 13 Homolog C

2768.	NOTCH2NLA	Notch 2 N-Terminal Like A
2769.	DPYD	Dihydropyrimidine Dehydrogenase
2770.	PLCG2	Phospholipase C Gamma 2
2771.	RAC2	Rac Family Small GTPase 2
2772.	GATA3	GATA Binding Protein 3
2773.	TACR3	Tachykinin Receptor 3
2774.	FST	Follistatin
2775.	KCNMA1	Potassium Calcium-Activated Channel Subfamily M Alpha 1
2776.	ACO2	Aconitase 2
2777.	CETP	Cholesteryl Ester Transfer Protein
2778.	COL18A1	Collagen Type XVIII Alpha 1 Chain
2779.	NEDD4L	NEDD4 Like E3 Ubiquitin Protein Ligase
2780.	PGD	Phosphogluconate Dehydrogenase
2781.	PLIN1	Perilipin 1
2782.	TPMT	Thiopurine S-Methyltransferase
2783.	ACVR2A	Activin A Receptor Type 2A
2784.	ETV6	ETS Variant Transcription Factor 6
2785.	GLO1	Glyoxalase I
2786.	LAMA1	Laminin Subunit Alpha 1
2787.	PI4KB	Phosphatidylinositol 4-Kinase Beta
2788.	POLA1	DNA Polymerase Alpha 1, Catalytic Subunit
2789.	EXT2	Exostosin Glycosyltransferase 2
2790.	PAX8	Paired Box 8
2791.	SLC7A7	Solute Carrier Family 7 Member 7
2792.	DGCR8	DGCR8 Microprocessor Complex Subunit
2793.	DISC1	DISC1 Scaffold Protein
2794.	LARS1	Leucyl-TRNA Synthetase 1
2795.	LY96	Lymphocyte Antigen 96
2796.	PVR	PVR Cell Adhesion Molecule
2797.	TNKS2	Tankyrase 2
2798.	TRIM32	Tripartite Motif Containing 32
2799.	ADCY7	Adenylate Cyclase 7
2800.	CES2	Carboxylesterase 2
2801.	COL9A2	Collagen Type IX Alpha 2 Chain
2802.	COMMD1	Copper Metabolism Domain Containing 1
2803.	HTR3B	5-Hydroxytryptamine Receptor 3B
2804.	NDUFA6	NADH:Ubiquinone Oxidoreductase Subunit A6
2805.	NFU1	NFU1 Iron-Sulfur Cluster Scaffold
2806.	RPGRIP1L	RPGRIP1 Like
2807.	S100A6	S100 Calcium Binding Protein A6
2808.	TPR	Translocated Promoter Region, Nuclear Basket Protein

2809.	C7	Complement C7
2810.	DLL3	Delta Like Canonical Notch Ligand 3
2811.	FKTN	Fukutin
2812.	FMOD	Fibromodulin
2813.	IFT88	Intraflagellar Transport 88
2814.	KIF3A	Kinesin Family Member 3A
2815.	MKS1	MKS Transition Zone Complex Subunit 1
2816.	NMT1	N-Myristoyltransferase 1
2817.	NPPC	Natriuretic Peptide C
2818.	PIGK	Phosphatidylinositol Glycan Anchor Biosynthesis Class K
2819.	RPS7	Ribosomal Protein S7
2820.	TCN1	Transcobalamin 1
2821.	CCN1	Cellular Communication Network Factor 1
2822.	CYP2C18	Cytochrome P450 Family 2 Subfamily C Member 18
2823.	DDAH2	DDAH Family Member 2, ADMA-Independent
2824.	KISS1	KiSS-1 Metastasis Suppressor
2825.	RPLP0	Ribosomal Protein Lateral Stalk Subunit P0
2826.	ALX1	ALX Homeobox 1
2827.	C6	Complement C6
2828.	CBY1	Chibby 1, Beta Catenin Antagonist
2829.	CPSF3	Cleavage And Polyadenylation Specific Factor 3
2830.	HPR	Haptoglobin-Related Protein
2831.	P3H2	Prolyl 3-Hydroxylase 2
2832.	SLC35C1	Solute Carrier Family 35 Member C1
2833.	USP18	Ubiquitin Specific Peptidase 18
2834.	ACBD3	Acyl-CoA Binding Domain Containing 3
2835.	MIA2	MIA SH3 Domain ER Export Factor 2
2836.	MLLT3	MLLT3 Super Elongation Complex Subunit
2837.	PJA1	Praja Ring Finger Ubiquitin Ligase 1
2838.	APOL2	Apolipoprotein L2
2839.	CABLES1	Cdk5 And Abl Enzyme Substrate 1
2840.	ERC2	ELKS/RAB6-Interacting/CAST Family Member 2
2841.	GLIS3	GLIS Family Zinc Finger 3
2842.	HYCC1	Hyccin PI4KA Lipid Kinase Complex Subunit 1
2843.	KCTD7	Potassium Channel Tetramerization Domain Containing 7
2844.	PSG1	Pregnancy Specific Beta-1-Glycoprotein 1
2845.	SLC25A38	Solute Carrier Family 25 Member 38
2846.	SPAG6	Sperm Associated Antigen 6
2847.	SRGAP2	SLIT-ROBO Rho GTPase Activating Protein 2
2848.	SRGAP3	SLIT-ROBO Rho GTPase Activating Protein 3
2849.	TBPL1	TATA-Box Binding Protein Like 1

2850.	B4GAT1	Beta-1,4-Glucuronyltransferase 1
2851.	GJC3	Gap Junction Protein Gamma 3
2852.	KIR2DL4	Killer Cell Immunoglobulin Like Receptor, Two Ig Domains And Long Cytoplasmic Tail 4
2853.	NMT2	N-Myristoyltransferase 2
2854.	PIGF	Phosphatidylinositol Glycan Anchor Biosynthesis Class F
2855.	ANKMY1	Ankyrin Repeat And MYND Domain Containing 1
2856.	APOL4	Apolipoprotein L4
2857.	GCM1	Glial Cells Missing Transcription Factor 1
2858.	ISLR2	Immunoglobulin Superfamily Containing Leucine Rich Repeat 2
2859.	PKD1L1	Polycystin 1 Like 1, Transient Receptor Potential Channel Interacting
2860.	RNF130	Ring Finger Protein 130
2861.	SPAG17	Sperm Associated Antigen 17
2862.	TUT1	Terminal Uridylyl Transferase 1, U6 SnRNA-Specific
2863.	CFAP43	Cilia And Flagella Associated Protein 43
2864.	COA5	Cytochrome C Oxidase Assembly Factor 5
2865.	LARP4	La Ribonucleoprotein 4
2866.	SNAPC3	Small Nuclear RNA Activating Complex Polypeptide 3
2867.	VPS72	Vacuolar Protein Sorting 72 Homolog
2868.	ZSWIM6	Zinc Finger SWIM-Type Containing 6
2869.	PLAC1	Placenta Enriched 1
2870.	CLEC5A	C-Type Lectin Domain Containing 5A
2871.	HSH2D	Hematopoietic SH2 Domain Containing
2872.	MAPK1IP1L	Mitogen-Activated Protein Kinase 1 Interacting Protein 1 Like
2873.	CLRN2	Clarin 2
2874.	CBARP	CACN Subunit Beta Associated Regulatory Protein
2875.	SEL1L2	SEL1L2 Adaptor Subunit Of SYVN1 Ubiquitin Ligase
2876.	ZNF469	Zinc Finger Protein 469
2877.	IGHD	Immunoglobulin Heavy Constant Delta
2878.	FREY1	Frey Regulator Of Sperm-Oocyte Fusion 1
2879.	PHGDH	Phosphoglycerate Dehydrogenase
2880.	FKBP5	FKBP Prolyl Isomerase 5
2881.	TFPI	Tissue Factor Pathway Inhibitor
2882.	SUCLG1	Succinate-CoA Ligase GDP/ADP-Forming Subunit Alpha
2883.	TSG101	Tumor Susceptibility 101
2884.	IL12RB2	Interleukin 12 Receptor Subunit Beta 2
2885.	ADGRG6	Adhesion G Protein-Coupled Receptor G6
2886.	DYNLL1	Dynein Light Chain LC8-Type 1
2887.	UMOD	Uromodulin
2888.	AP1S2	Adaptor Related Protein Complex 1 Subunit Sigma 2
2889.	DNA2	DNA Replication Helicase/Nuclease 2
2890.	FABP2	Fatty Acid Binding Protein 2

2891.	SLC35A3	Solute Carrier Family 35 Member A3
2892.	CYGB	Cytoglobin
2893.	ADGRB2	Adhesion G Protein-Coupled Receptor B2
2894.	ADGRF5	Adhesion G Protein-Coupled Receptor F5
2895.	FUT7	Fucosyltransferase 7
2896.	GNA15	G Protein Subunit Alpha 15
2897.	RPL18A	Ribosomal Protein L18a
2898.	ADGRG3	Adhesion G Protein-Coupled Receptor G3
2899.	GTF2H2	General Transcription Factor IIH Subunit 2
2900.	MGME1	Mitochondrial Genome Maintenance Exonuclease 1
2901.	SAP30BP	SAP30 Binding Protein
2902.	ANAPC13	Anaphase Promoting Complex Subunit 13
2903.	SPCS3	Signal Peptidase Complex Subunit 3
2904.	TPSD1	Tryptase Delta 1
2905.	ADGRG5	Adhesion G Protein-Coupled Receptor G5
2906.	ADGRF1	Adhesion G Protein-Coupled Receptor F1
2907.	CHRFAM7A	CHRNA7 (Exons 5-10) And FAM7A (Exons A-E) Fusion
2908.	CCDC85A	Coiled-Coil Domain Containing 85A
2909.	CT47A1	Cancer/Testis Antigen Family 47 Member A1
2910.	CT47A11	Cancer/Testis Antigen Family 47 Member A11
2911.	CT47A6	Cancer/Testis Antigen Family 47 Member A6
2912.	CT47A2	Cancer/Testis Antigen Family 47 Member A2
2913.	CT47A3	Cancer/Testis Antigen Family 47 Member A3
2914.	CT47A5	Cancer/Testis Antigen Family 47 Member A5
2915.	CT47A7	Cancer/Testis Antigen Family 47 Member A7
2916.	CT47A10	Cancer/Testis Antigen Family 47 Member A10
2917.	CT47A12	Cancer/Testis Antigen Family 47 Member A12
2918.	CT47A8	Cancer/Testis Antigen Family 47 Member A8
2919.	CT47A9	Cancer/Testis Antigen Family 47 Member A9
2920.	KLK3	Kallikrein Related Peptidase 3
2921.	ODAD3	Outer Dynein Arm Docking Complex Subunit 3
2922.	DRC1	Dynein Regulatory Complex Subunit 1
2923.	ADD3	Adducin 3
2924.	HGS	Hepatocyte Growth Factor-Regulated Tyrosine Kinase Substrate
2925.	GLUL	Glutamate-Ammonia Ligase
2926.	MERTK	MER Proto-Oncogene, Tyrosine Kinase
2927.	BLK	BLK Proto-Oncogene, Src Family Tyrosine Kinase
2928.	ACTN1	Actinin Alpha 1
2929.	CUX1	Cut Like Homeobox 1
2930.	DDB2	Damage Specific DNA Binding Protein 2
2931.	TGFBR3	Transforming Growth Factor Beta Receptor 3

2932.	CDC7	Cell Division Cycle 7
2933.	FOXP1	Forkhead Box P1
2934.	NSF	N-Ethylmaleimide Sensitive Factor, Vesicle Fusing ATPase
2935.	RBL2	RB Transcriptional Corepressor Like 2
2936.	DUSP10	Dual Specificity Phosphatase 10
2937.	UBE2D3	Ubiquitin Conjugating Enzyme E2 D3
2938.	CDC42BPB	CDC42 Binding Protein Kinase Beta
2939.	GRB10	Growth Factor Receptor Bound Protein 10
2940.	LYVE1	Lymphatic Vessel Endothelial Hyaluronan Receptor 1
2941.	PRKAR2B	Protein Kinase CAMP-Dependent Type II Regulatory Subunit Beta
2942.	CHST6	Carbohydrate Sulfotransferase 6
2943.	GNL3	G Protein Nucleolar 3
2944.	MACF1	Microtubule Actin Crosslinking Factor 1
2945.	UBE3C	Ubiquitin Protein Ligase E3C
2946.	PIP5K1A	Phosphatidylinositol-4-Phosphate 5-Kinase Type 1 Alpha
2947.	AMN	Amnion Associated Transmembrane Protein
2948.	ASH1L	ASH1 Like Histone Lysine Methyltransferase
2949.	GTF2I	General Transcription Factor IIi
2950.	H1-2	H1.2 Linker Histone, Cluster Member
2951.	HSF2	Heat Shock Transcription Factor 2
2952.	OS9	OS9 Endoplasmic Reticulum Lectin
2953.	PABPC4	Poly(A) Binding Protein Cytoplasmic 4
2954.	STK38L	Serine/Threonine Kinase 38 Like
2955.	UBE2V2	Ubiquitin Conjugating Enzyme E2 V2
2956.	ZNF462	Zinc Finger Protein 462
2957.	CEPT1	Choline/Ethanolamine Phosphotransferase 1
2958.	HDGF	Heparin Binding Growth Factor
2959.	HHAT	Hedgehog Acyltransferase
2960.	PANK4	Pantothenate Kinase 4 (Inactive)
2961.	TIMELESS	Timeless Circadian Regulator
2962.	AFF3	ALF Transcription Elongation Factor 3
2963.	BTN2A1	Butyrophilin Subfamily 2 Member A1
2964.	DAP3	Death Associated Protein 3
2965.	DENND1A	DENN Domain Containing 1A
2966.	EMSY	EMSY Transcriptional Repressor, BRCA2 Interacting
2967.	MSTO1	Misato Mitochondrial Distribution And Morphology Regulator 1
2968.	RFC3	Replication Factor C Subunit 3
2969.	SLC6A15	Solute Carrier Family 6 Member 15
2970.	TUFT1	Tuftelin 1
2971.	WDR12	WD Repeat Domain 12
2972.	AMBRA1	Autophagy And Beclin 1 Regulator 1

2973.	ARHGEF16	Rho Guanine Nucleotide Exchange Factor 16
2974.	BMP8A	Bone Morphogenetic Protein 8a
2975.	DGKZ	Diacylglycerol Kinase Zeta
2976.	DRAM2	DNA Damage Regulated Autophagy Modulator 2
2977.	EBF2	EBF Transcription Factor 2
2978.	FAM167A	Family With Sequence Similarity 167 Member A
2979.	FIGNL1	Fidgetin Like 1
2980.	FRAS1	Fraser Extracellular Matrix Complex Subunit 1
2981.	GPR12	G Protein-Coupled Receptor 12
2982.	H1-3	H1.3 Linker Histone, Cluster Member
2983.	MAU2	MAU2 Sister Chromatid Cohesion Factor
2984.	PACSIN3	Protein Kinase C And Casein Kinase Substrate In Neurons 3
2985.	PTBP2	Polypyrimidine Tract Binding Protein 2
2986.	STARD13	StAR Related Lipid Transfer Domain Containing 13
2987.	BTN3A1	Butyrophilin Subfamily 3 Member A1
2988.	BTN3A2	Butyrophilin Subfamily 3 Member A2
2989.	CEP131	Centrosomal Protein 131
2990.	CNPY2	Canopy FGF Signaling Regulator 2
2991.	NACA	Nascent Polypeptide Associated Complex Subunit Alpha
2992.	PGM5	Phosphoglucomutase 5
2993.	TMEM87B	Transmembrane Protein 87B
2994.	ARHGEF25	Rho Guanine Nucleotide Exchange Factor 25
2995.	CHSY3	Chondroitin Sulfate Synthase 3
2996.	DCP1A	Decapping MRNA 1A
2997.	GON4L	Gon-4 Like
2998.	INHBE	Inhibin Subunit Beta E
2999.	MRPL24	Mitochondrial Ribosomal Protein L24
3000.	MYCBP	MYC Binding Protein
3001.	RBMS2	RNA Binding Motif Single Stranded Interacting Protein 2
3002.	SH2B2	SH2B Adaptor Protein 2
3003.	SGIP1	SH3GL Interacting Endocytic Adaptor 1
3004.	SMG5	SMG5 Nonsense Mediated MRNA Decay Factor
3005.	TSKU	Tsukushi, Small Leucine Rich Proteoglycan
3006.	ZNF638	Zinc Finger Protein 638
3007.	BTN2A2	Butyrophilin Subfamily 2 Member A2
3008.	HAPLN2	Hyaluronan And Proteoglycan Link Protein 2
3009.	IL20RB	Interleukin 20 Receptor Subunit Beta
3010.	KCNK17	Potassium Two Pore Domain Channel Subfamily K Member 17
3011.	MRPL1	Mitochondrial Ribosomal Protein L1
3012.	NFE2L3	NFE2 Like BZIP Transcription Factor 3
3013.	PPP2R3A	Protein Phosphatase 2 Regulatory Subunit B"Alpha

3014.	RPAP2	RNA Polymerase II Associated Protein 2
3015.	WRAP73	WD Repeat Containing, Antisense To TP73
3016.	ANAPC4	Anaphase Promoting Complex Subunit 4
3017.	ATP23	ATP23 Metallopeptidase And ATP Synthase Assembly Factor Homolog
3018.	CARF	Calcium Responsive Transcription Factor
3019.	DBX1	Developing Brain Homeobox 1
3020.	EML6	EMAP Like 6
3021.	ETV3	ETS Variant Transcription Factor 3
3022.	ISG20L2	Interferon Stimulated Exonuclease Gene 20 Like 2
3023.	MSL2	MSL Complex Subunit 2
3024.	MYO1H	Myosin IH
3025.	OXCT2	3-Oxoacid CoA-Transferase 2
3026.	SRRM3	Serine/Arginine Repetitive Matrix 3
3027.	ZBTB39	Zinc Finger And BTB Domain Containing 39
3028.	ZC3H10	Zinc Finger CCCH-Type Containing 10
3029.	ZCCHC2	Zinc Finger CCHC-Type Containing 2
3030.	CNOT6L	CCR4-NOT Transcription Complex Subunit 6 Like
3031.	EXD2	Exonuclease 3'-5' Domain Containing 2
3032.	FLACC1	Flagellum Associated Containing Coiled-Coil Domains 1
3033.	GJA9	Gap Junction Protein Alpha 9
3034.	GPATCH4	G-Patch Domain Containing 4 (Gene/Pseudogene)
3035.	MARCHF9	Membrane Associated Ring-CH-Type Finger 9
3036.	MOAP1	Modulator Of Apoptosis 1
3037.	NBEAL1	Neurobeachin Like 1
3038.	NUFIP2	Nuclear FMR1 Interacting Protein 2
3039.	TAFA1	TAFA Chemokine Like Family Member 1
3040.	ZCCHC4	Zinc Finger CCHC-Type Containing 4
3041.	HMGN4	High Mobility Group Nucleosomal Binding Domain 4
3042.	LYSET	Lysosomal Enzyme Trafficking Factor
3043.	RFLNA	Refilin A
3044.	ENTREP1	Endosomal Transmembrane Epsin Interactor 1
3045.	KHDC4	KH Domain Containing 4, Pre-MRNA Splicing Factor
3046.	ST7L	Suppression Of Tumorigenicity 7 Like
3047.	TBPL2	TATA-Box Binding Protein Like 2
3048.	C1orf174	Chromosome 1 Open Reading Frame 174
3049.	CLHC1	Clathrin Heavy Chain Linker Domain Containing 1
3050.	PAQR6	Progestin And AdipoQ Receptor Family Member 6
3051.	METTL25B	Methyltransferase Like 25B
3052.	ZNF391	Zinc Finger Protein 391
3053.	POLR2J2	RNA Polymerase II Subunit J2
3054.	RPL41	Ribosomal Protein L41

3055.	ZNF322	Zinc Finger Protein 322
3056.	INKA2	Inka Box Actin Regulator 2
3057.	PGPEP1L	Pyroglutamyl-Peptidase I Like
3058.	DEFB134	Defensin Beta 134
3059.	ZNF474	Zinc Finger Protein 474
3060.	RIIAD1	Regulatory Subunit Of Type II PKA R-Subunit Domain Containing 1
3061.	C20orf203	Chromosome 20 Open Reading Frame 203
3062.	TMIGD3	Transmembrane And Immunoglobulin Domain Containing 3
3063.	CFAP97D2	CFAP97 Domain Containing 2
3064.	TBC1D7-LOC100130357	TBC1D7-LOC100130357 Readthrough
3065.	ENSG00000285708	Novel Protein
3066.	LOC124903827	Translation Initiation Factor IF-2
3067.	PDE2A	Phosphodiesterase 2A
3068.	PDE3B	Phosphodiesterase 3B
3069.	AGRN	Agrin
3070.	GATA6	GATA Binding Protein 6
3071.	MYBPC3	Myosin Binding Protein C3
3072.	RYR2	Ryanodine Receptor 2
3073.	KCNA5	Potassium Voltage-Gated Channel Subfamily A Member 5
3074.	ATP13A3	ATPase 13A3
3075.	WARS1	Tryptophanyl-TRNA Synthetase 1
3076.	POLR1C	RNA Polymerase I And III Subunit C
3077.	BCAP31	B Cell Receptor Associated Protein 31
3078.	SNX22	Sorting Nexin 22
3079.	GABRA1	Gamma-Aminobutyric Acid Type A Receptor Subunit Alpha1
3080.	F2R	Coagulation Factor II Thrombin Receptor
3081.	PSAT1	Phosphoserine Aminotransferase 1
3082.	FHIT	Fragile Histidine Triad Diadenosine Triphosphatase
3083.	UCP1	Uncoupling Protein 1
3084.	CCL24	C-C Motif Chemokine Ligand 24
3085.	CRCP	CGRP Receptor Component
3086.	EPHA2	EPH Receptor A2
3087.	PPP3CA	Protein Phosphatase 3 Catalytic Subunit Alpha
3088.	ALDOA	Aldolase, Fructose-Bisphosphate A
3089.	GABRB3	Gamma-Aminobutyric Acid Type A Receptor Subunit Beta3
3090.	SLC9A1	Solute Carrier Family 9 Member A1
3091.	VKORC1	Vitamin K Epoxide Reductase Complex Subunit 1
3092.	PRKCZ	Protein Kinase C Zeta
3093.	RRAS2	RAS Related 2
3094.	STX1A	Syntaxin 1A
3095.	ADRA2A	Adrenoceptor Alpha 2A

3096.	BARD1	BRCA1 Associated RING Domain 1
3097.	BMP7	Bone Morphogenetic Protein 7
3098.	HEXB	Hexosaminidase Subunit Beta
3099.	MAPK10	Mitogen-Activated Protein Kinase 10
3100.	NEDD4	NEDD4 E3 Ubiquitin Protein Ligase
3101.	P2RX7	Purinergic Receptor P2X 7
3102.	PCCA	Propionyl-CoA Carboxylase Subunit Alpha
3103.	RAD54L	RAD54 Like
3104.	AGL	Amylo-Alpha-1, 6-Glucosidase, 4-Alpha-Glucanotransferase
3105.	ATP5F1A	ATP Synthase F1 Subunit Alpha
3106.	ERCC1	ERCC Excision Repair 1, Endonuclease Non-Catalytic Subunit
3107.	NME1	NME/NM23 Nucleoside Diphosphate Kinase 1
3108.	PEPD	Peptidase D
3109.	PLD2	Phospholipase D2
3110.	PSMB4	Proteasome 20S Subunit Beta 4
3111.	ROS1	ROS Proto-Oncogene 1, Receptor Tyrosine Kinase
3112.	TCF3	Transcription Factor 3
3113.	ADRA2C	Adrenoceptor Alpha 2C
3114.	AIRE	Autoimmune Regulator
3115.	BUB3	BUB3 Mitotic Checkpoint Protein
3116.	CPOX	Coproporphyrinogen Oxidase
3117.	DCT	Dopachrome Tautomerase
3118.	E2F4	E2F Transcription Factor 4
3119.	FKBP4	FKBP Prolyl Isomerase 4
3120.	FZD7	Frizzled Class Receptor 7
3121.	GBE1	1,4-Alpha-Glucan Branching Enzyme 1
3122.	GYS2	Glycogen Synthase 2
3123.	PGAM1	Phosphoglycerate Mutase 1
3124.	RHOB	Ras Homolog Family Member B
3125.	SHC1	SHC Adaptor Protein 1
3126.	SLC34A2	Solute Carrier Family 34 Member 2
3127.	TBX2	T-Box Transcription Factor 2
3128.	TPK1	Thiamin Pyrophosphokinase 1
3129.	CMA1	Chymase 1
3130.	COL9A3	Collagen Type IX Alpha 3 Chain
3131.	DAB1	DAB Adaptor Protein 1
3132.	DLG1	Discs Large MAGUK Scaffold Protein 1
3133.	EFNA1	Ephrin A1
3134.	GAB1	GRB2 Associated Binding Protein 1
3135.	IGFBP4	Insulin Like Growth Factor Binding Protein 4
3136.	PIK3R3	Phosphoinositide-3-Kinase Regulatory Subunit 3

3137.	PNPT1	Polyribonucleotide Nucleotidyltransferase 1
3138.	PTPRZ1	Protein Tyrosine Phosphatase Receptor Type Z1
3139.	RPTOR	Regulatory Associated Protein Of MTOR Complex 1
3140.	SLC7A5	Solute Carrier Family 7 Member 5
3141.	TTBK2	Tau Tubulin Kinase 2
3142.	AP1B1	Adaptor Related Protein Complex 1 Subunit Beta 1
3143.	CENPF	Centromere Protein F
3144.	E2F3	E2F Transcription Factor 3
3145.	ENPP3	Ectonucleotide Pyrophosphatase/Phosphodiesterase 3
3146.	HSPE1	Heat Shock Protein Family E (Hsp10) Member 1
3147.	NOX4	NADPH Oxidase 4
3148.	PAICS	Phosphoribosylaminoimidazole Carboxylase And Phosphoribosylaminoimidazolesuccinocarboxamide Synthase
3149.	PAX1	Paired Box 1
3150.	RHOC	Ras Homolog Family Member C
3151.	S1PR2	Sphingosine-1-Phosphate Receptor 2
3152.	SLC1A7	Solute Carrier Family 1 Member 7
3153.	TRIM5	Tripartite Motif Containing 5
3154.	ABCD4	ATP Binding Cassette Subfamily D Member 4
3155.	BICD2	BICD Cargo Adaptor 2
3156.	CLN8	CLN8 Transmembrane ER And ERGIC Protein
3157.	DIAPH3	Diaphanous Related Formin 3
3158.	EN2	Engrailed Homeobox 2
3159.	ETS2	ETS Proto-Oncogene 2, Transcription Factor
3160.	GOPC	Golgi Associated PDZ And Coiled-Coil Motif Containing
3161.	IRS4	Insulin Receptor Substrate 4
3162.	RAD52	RAD52 Homolog, DNA Repair Protein
3163.	RECK	Reversion Inducing Cysteine Rich Protein With Kazal Motifs
3164.	RPS16	Ribosomal Protein S16
3165.	RRAS	RAS Related
3166.	VAMP7	Vesicle Associated Membrane Protein 7
3167.	ZBP1	Z-DNA Binding Protein 1
3168.	E2F2	E2F Transcription Factor 2
3169.	PLEK	Pleckstrin
3170.	EIF1AX	Eukaryotic Translation Initiation Factor 1A X-Linked
3171.	ELAVL4	ELAV Like RNA Binding Protein 4
3172.	JPH1	Junctophilin 1
3173.	KIAA0319	KIAA0319
3174.	MRC1	Mannose Receptor C-Type 1
3175.	MSRB2	Methionine Sulfoxide Reductase B2
3176.	NFIC	Nuclear Factor I C
3177.	NLRP7	NLR Family Pyrin Domain Containing 7

3178.	OGA	O-GlcNAcase
3179.	RAPGEF4	Rap Guanine Nucleotide Exchange Factor 4
3180.	RPH3A	Rabphilin 3A
3181.	SPART	Spartin
3182.	AS3MT	Arsenite Methyltransferase
3183.	E2F5	E2F Transcription Factor 5
3184.	MRPS12	Mitochondrial Ribosomal Protein S12
3185.	RHOG	Ras Homolog Family Member G
3186.	ADAP1	ArfGAP With Dual PH Domains 1
3187.	AP5Z1	Adaptor Related Protein Complex 5 Subunit Zeta 1
3188.	CCL22	C-C Motif Chemokine Ligand 22
3189.	DUSP13B	Dual Specificity Phosphatase 13B
3190.	MED27	Mediator Complex Subunit 27
3191.	MX2	MX Dynamin Like GTPase 2
3192.	OASL	2'-5'-Oligoadenylate Synthetase Like
3193.	PENK	Proenkephalin
3194.	TFB2M	Transcription Factor B2, Mitochondrial
3195.	ADGRB3	Adhesion G Protein-Coupled Receptor B3
3196.	ATPAF1	ATP Synthase Mitochondrial F1 Complex Assembly Factor 1
3197.	FCHO2	FCH And Mu Domain Containing Endocytic Adaptor 2
3198.	HEYL	Hes Related Family BHLH Transcription Factor With YRPW Motif Like
3199.	PHF3	PHD Finger Protein 3
3200.	RIPPLY2	Ripply Transcriptional Repressor 2
3201.	SERAC1	Serine Active Site Containing 1
3202.	ADGRB1	Adhesion G Protein-Coupled Receptor B1
3203.	CBX8	Chromobox 8
3204.	EPM2AIP1	EPM2A Interacting Protein 1
3205.	FMNL1	Formin Like 1
3206.	RAB24	RAB24, Member RAS Oncogene Family
3207.	RAB26	RAB26, Member RAS Oncogene Family
3208.	STX17	Syntaxin 17
3209.	ATF5	Activating Transcription Factor 5
3210.	CPLANE1	Ciliogenesis And Planar Polarity Effector Complex Subunit 1
3211.	TCF25	Transcription Factor 25
3212.	CCDC97	Coiled-Coil Domain Containing 97
3213.	DIRAS3	DIRAS Family GTPase 3
3214.	H3C4	H3 Clustered Histone 4
3215.	OR14C36	Olfactory Receptor Family 14 Subfamily C Member 36
3216.	LRRC63	Leucine Rich Repeat Containing 63
3217.	BORCS7	BLOC-1 Related Complex Subunit 7
3218.	MFSD13A	Major Facilitator Superfamily Domain Containing 13A

3219.	PCDH1	Protocadherin 1
3220.	TMEM98	Transmembrane Protein 98
3221.	EBNA1BP2	EBNA1 Binding Protein 2
3222.	HDAC6	Histone Deacetylase 6
3223.	GSN	Gelsolin
3224.	KCNJ1	Potassium Inwardly Rectifying Channel Subfamily J Member 1
3225.	SLC5A2	Solute Carrier Family 5 Member 2
3226.	YWHAQ	Tyrosine 3-Monooxygenase/Tryptophan 5-Monooxygenase Activation Protein Theta
3227.	FGF17	Fibroblast Growth Factor 17
3228.	SORL1	Sortilin Related Receptor 1
3229.	FGF5	Fibroblast Growth Factor 5
3230.	SYN1	Synapsin I
3231.	MFN1	Mitofusin 1
3232.	TFEB	Transcription Factor EB
3233.	FGF16	Fibroblast Growth Factor 16
3234.	FIS1	Fission, Mitochondrial 1
3235.	FGF22	Fibroblast Growth Factor 22
3236.	RGPD3	RANBP2 Like And GRIP Domain Containing 3
3237.	KCNJ8	Potassium Inwardly Rectifying Channel Subfamily J Member 8
3238.	CBFB	Core-Binding Factor Subunit Beta
3239.	YWHAB	Tyrosine 3-Monooxygenase/Tryptophan 5-Monooxygenase Activation Protein Beta
3240.	DLD	Dihydrolipoamide Dehydrogenase
3241.	KARS1	Lysyl-TRNA Synthetase 1
3242.	NDRG1	N-Myc Downstream Regulated 1
3243.	HINT1	Histidine Triad Nucleotide Binding Protein 1
3244.	SLC13A5	Solute Carrier Family 13 Member 5
3245.	TAF15	TATA-Box Binding Protein Associated Factor 15
3246.	CCNO	Cyclin O
3247.	LRP12	LDL Receptor Related Protein 12
3248.	LRSAM1	Leucine Rich Repeat And Sterile Alpha Motif Containing 1
3249.	DOK7	Docking Protein 7
3250.	SLC25A46	Solute Carrier Family 25 Member 46
3251.	TDRKH	Tudor And KH Domain Containing
3252.	ZMYND10	Zinc Finger MYND-Type Containing 10
3253.	CCDC39	Coiled-Coil Domain 39 Molecular Ruler Complex Subunit
3254.	DNAAF3	Dynein Axonemal Assembly Factor 3
3255.	PITX2	Paired Like Homeodomain 2
3256.	CALM2	Calmodulin 2
3257.	KCNE2	Potassium Voltage-Gated Channel Subfamily E Regulatory Subunit 2
3258.	MICAL1	Microtubule Associated Monooxygenase, Calponin And LIM Domain Containing 1

3259.	MYOZ2	Myozenin 2
3260.	KIF20A	Kinesin Family Member 20A
3261.	CFAP45	Cilia And Flagella Associated Protein 45
3262.	CA4	Carbonic Anhydrase 4
3263.	ERG	ETS Transcription Factor ERG
3264.	GNB5	G Protein Subunit Beta 5
3265.	EXOSC3	Exosome Component 3
3266.	TCOF1	Treacle Ribosome Biogenesis Factor 1
3267.	LRAT	Lecithin Retinol Acyltransferase
3268.	CD9	CD9 Molecule
3269.	PREP	Prolyl Endopeptidase
3270.	FANCA	FA Complementation Group A
3271.	ENTPD1	Ectonucleoside Triphosphate Diphosphohydrolase 1
3272.	FTH1	Ferritin Heavy Chain 1
3273.	GCK	Glucokinase
3274.	GRIN2D	Glutamate Ionotropic Receptor NMDA Type Subunit 2D
3275.	LAMB1	Laminin Subunit Beta 1
3276.	GABRA5	Gamma-Aminobutyric Acid Type A Receptor Subunit Alpha5
3277.	IL2RG	Interleukin 2 Receptor Subunit Gamma
3278.	ITGAV	Integrin Subunit Alpha V
3279.	OXTR	Oxytocin Receptor
3280.	A2M	Alpha-2-Macroglobulin
3281.	ABCB11	ATP Binding Cassette Subfamily B Member 11
3282.	SERPING1	Serpin Family G Member 1
3283.	DVL3	Dishevelled Segment Polarity Protein 3
3284.	NUP98	Nucleoporin 98 And 96 Precursor
3285.	DFFA	DNA Fragmentation Factor Subunit Alpha
3286.	LRPAP1	LDL Receptor Related Protein Associated Protein 1
3287.	NPR3	Natriuretic Peptide Receptor 3
3288.	PCNT	Pericentrin
3289.	PTGIS	Prostaglandin I2 Synthase
3290.	SLC31A1	Solute Carrier Family 31 Member 1
3291.	HNF1B	HNF1 Homeobox B
3292.	PCSK2	Proprotein Convertase Subtilisin/Kexin Type 2
3293.	PLEC	Plectin
3294.	POMT2	Protein O-Mannosyltransferase 2
3295.	KCNQ5	Potassium Voltage-Gated Channel Subfamily Q Member 5
3296.	LGMN	Legumain
3297.	MIPEP	Mitochondrial Intermediate Peptidase
3298.	MUC5B	Mucin 5B, Oligomeric Mucus/Gel-Forming
3299.	NFIA	Nuclear Factor I A

3300.	SLC12A7	Solute Carrier Family 12 Member 7
3301.	TULP3	TUB Like Protein 3
3302.	BBS2	Bardet-Biedl Syndrome 2
3303.	BBS4	Bardet-Biedl Syndrome 4
3304.	CXCR5	C-X-C Motif Chemokine Receptor 5
3305.	FREM1	FRAS1 Related Extracellular Matrix 1
3306.	GPSM2	G Protein Signaling Modulator 2
3307.	IL24	Interleukin 24
3308.	METTL3	Methyltransferase 3, N6-Adenosine-Methyltransferase Complex Catalytic Subunit
3309.	SLC6A12	Solute Carrier Family 6 Member 12
3310.	CCL21	C-C Motif Chemokine Ligand 21
3311.	EML1	EMAP Like 1
3312.	FLT3LG	Fms Related Receptor Tyrosine Kinase 3 Ligand
3313.	INSRR	Insulin Receptor Related Receptor
3314.	STX16	Syntaxin 16
3315.	ANKS6	Ankyrin Repeat And Sterile Alpha Motif Domain Containing 6
3316.	BBS7	Bardet-Biedl Syndrome 7
3317.	GLRX3	Glutaredoxin 3
3318.	NBR1	NBR1 Autophagy Cargo Receptor
3319.	OTUD5	OTU Deubiquitinase 5
3320.	QRSL1	Glutaminyl-TRNA Amidotransferase Subunit QRSL1
3321.	RS1	Retinoschisin 1
3322.	SLC39A1	Solute Carrier Family 39 Member 1
3323.	B3GLCT	Beta 3-Glucosyltransferase
3324.	DLG5	Discs Large MAGUK Scaffold Protein 5
3325.	DYNC2H1	Dynein Cytoplasmic 2 Heavy Chain 1
3326.	FAM3B	FAM3 Metabolism Regulating Signaling Molecule B
3327.	LRG1	Leucine Rich Alpha-2-Glycoprotein 1
3328.	MRI1	Methylthioribose-1-Phosphate Isomerase 1
3329.	MT1E	Metallothionein 1E
3330.	TRAPPC2	Trafficking Protein Particle Complex Subunit 2
3331.	ANKK1	Ankyrin Repeat And Kinase Domain Containing 1
3332.	CLEC16A	C-Type Lectin Domain Containing 16A
3333.	CLIC2	Chloride Intracellular Channel 2
3334.	DNAL4	Dynein Axonemal Light Chain 4
3335.	NSUN5	NOP2/Sun RNA Methyltransferase 5
3336.	PAF1	PAF1 Homolog, Paf1/RNA Polymerase II Complex Component
3337.	VPS54	VPS54 Subunit Of GARP Complex
3338.	AHSP	Alpha Hemoglobin Stabilizing Protein
3339.	EEFSEC	Eukaryotic Elongation Factor, Selenocysteine-TRNA Specific
3340.	EXOC1	Exocyst Complex Component 1

3341.	OPN1LW	Opsin 1, Long Wave Sensitive
3342.	POMK	Protein O-Mannose Kinase
3343.	SMG7	SMG7 Nonsense Mediated MRNA Decay Factor
3344.	CTTNBP2	Cortactin Binding Protein 2
3345.	IFT20	Intraflagellar Transport 20
3346.	MPP2	MAGUK P55 Scaffold Protein 2
3347.	MTFR1	Mitochondrial Fission Regulator 1
3348.	OPN1MW	Opsin 1, Medium Wave Sensitive
3349.	POC5	POC5 Centriolar Protein
3350.	S100A3	S100 Calcium Binding Protein A3
3351.	SFMBT1	Scm Like With Four Mbt Domains 1
3352.	TMEM119	Transmembrane Protein 119
3353.	MFSD6	Major Facilitator Superfamily Domain Containing 6
3354.	NXT1	Nuclear Transport Factor 2 Like Export Factor 1
3355.	VPS50	VPS50 Subunit Of EARP/GARPII Complex
3356.	GLIS1	GLIS Family Zinc Finger 1
3357.	KATNIP	Katanin Interacting Protein
3358.	NKX2-8	NK2 Homeobox 8
3359.	NTPCR	Nucleoside-Triphosphatase, Cancer-Related
3360.	CCDC85C	Coiled-Coil Domain Containing 85C
3361.	CMTR1	Cap Methyltransferase 1
3362.	DNAJC28	DnaJ Heat Shock Protein Family (Hsp40) Member C28
3363.	SUCO	SUN Domain Containing Ossification Factor
3364.	TMEM240	Transmembrane Protein 240
3365.	CCDC85B	Coiled-Coil Domain Containing 85B
3366.	CWH43	Cell Wall Biogenesis 43 C-Terminal Homolog
3367.	OBP2A	Odorant Binding Protein 2A
3368.	CENPV	Centromere Protein V
3369.	NACC2	NACC Family Member 2
3370.	PNRC2	Proline Rich Nuclear Receptor Coactivator 2
3371.	JHY	Junctional Cadherin Complex Regulator
3372.	TCF23	Transcription Factor 23
3373.	MYMK	Myomaker, Myoblast Fusion Factor
3374.	LOC102723407	Immunoglobulin Heavy Variable 4-38-2-Like
3375.	ATXN2L	Ataxin 2 Like
3376.	MFRP	Membrane Frizzled-Related Protein
3377.	TMEM41B	Transmembrane Protein 41B
3378.	GPATCH2L	G-Patch Domain Containing 2 Like
3379.	PRSS56	Serine Protease 56
3380.	TMEM41A	Transmembrane Protein 41A
3381.	TMEM64	Transmembrane Protein 64

3382.	NT5E	5'-Nucleotidase Ecto
3383.	GLB1	Galactosidase Beta 1
3384.	PDHA1	Pyruvate Dehydrogenase E1 Subunit Alpha 1
3385.	NTF4	Neurotrophin 4
3386.	TAF1	TATA-Box Binding Protein Associated Factor 1
3387.	ETFDH	Electron Transfer Flavoprotein Dehydrogenase
3388.	THY1	Thy-1 Cell Surface Antigen
3389.	SLC17A7	Solute Carrier Family 17 Member 7
3390.	REEP1	Receptor Accessory Protein 1
3391.	ATXN3L	Ataxin 3 Like
3392.	COG2	Component Of Oligomeric Golgi Complex 2
3393.	IL6ST	Interleukin 6 Cytokine Family Signal Transducer
3394.	NPM1	Nucleophosmin 1
3395.	FASN	Fatty Acid Synthase
3396.	GRIA4	Glutamate Ionotropic Receptor AMPA Type Subunit 4
3397.	SGK1	Serum/Glucocorticoid Regulated Kinase 1
3398.	STXBP1	Syntaxin Binding Protein 1
3399.	MAPK9	Mitogen-Activated Protein Kinase 9
3400.	F13A1	Coagulation Factor XIII A Chain
3401.	KMT2A	Lysine Methyltransferase 2A
3402.	MEF2C	Myocyte Enhancer Factor 2C
3403.	PTPRJ	Protein Tyrosine Phosphatase Receptor Type J
3404.	ACAN	Aggrecan
3405.	CASP2	Caspase 2
3406.	CYBA	Cytochrome B-245 Alpha Chain
3407.	GYG1	Glycogenin 1
3408.	LIFR	LIF Receptor Subunit Alpha
3409.	NOG	Noggin
3410.	VCAN	Versican
3411.	ARID1A	AT-Rich Interaction Domain 1A
3412.	CHRM1	Cholinergic Receptor Muscarinic 1
3413.	CHRNB2	Cholinergic Receptor Nicotinic Beta 2 Subunit
3414.	FDPS	Farnesyl Diphosphate Synthase
3415.	GCGR	Glucagon Receptor
3416.	HNRNPA2B1	Heterogeneous Nuclear Ribonucleoprotein A2/B1
3417.	LOXL2	Lysyl Oxidase Like 2
3418.	CSTA	Cystatin A
3419.	GPD1	Glycerol-3-Phosphate Dehydrogenase 1
3420.	ICOS	Inducible T Cell Costimulator
3421.	IFNGR2	Interferon Gamma Receptor 2
3422.	NRP2	Neuropilin 2

3423.	RPS6	Ribosomal Protein S6
3424.	SARS2	Seryl-TRNA Synthetase 2, Mitochondrial
3425.	SLC1A4	Solute Carrier Family 1 Member 4
3426.	STAG2	STAG2 Cohesin Complex Component
3427.	AGPAT2	1-Acylglycerol-3-Phosphate O-Acyltransferase 2
3428.	CLPB	ClpB Family Mitochondrial Disaggregase
3429.	DUSP3	Dual Specificity Phosphatase 3
3430.	EXO1	Exonuclease 1
3431.	GREM1	Gremlin 1, DAN Family BMP Antagonist
3432.	KREMEN1	Kringle Containing Transmembrane Protein 1
3433.	MCM6	Minichromosome Maintenance Complex Component 6
3434.	MSH3	MutS Homolog 3
3435.	PRMT7	Protein Arginine Methyltransferase 7
3436.	SBDS	SBDS Ribosome Maturation Factor
3437.	SLC12A4	Solute Carrier Family 12 Member 4
3438.	SLC25A19	Solute Carrier Family 25 Member 19
3439.	SLC25A20	Solute Carrier Family 25 Member 20
3440.	ABCA2	ATP Binding Cassette Subfamily A Member 2
3441.	ADH1A	Alcohol Dehydrogenase 1A (Class I), Alpha Polypeptide
3442.	ARSG	Arylsulfatase G
3443.	AUTS2	Activator Of Transcription And Developmental Regulator AUTS2
3444.	BANF1	BAF Nuclear Assembly Factor 1
3445.	BMAL1	Basic Helix-Loop-Helix ARNT Like 1
3446.	C4B	Complement C4B (Chido/Rodgers Blood Group)
3447.	DNASE1L3	Deoxyribonuclease 1 Like 3
3448.	FBN2	Fibrillin 2
3449.	KDM4C	Lysine Demethylase 4C
3450.	KPNA3	Karyopherin Subunit Alpha 3
3451.	MCHR1	Melanin Concentrating Hormone Receptor 1
3452.	MMP20	Matrix Metallopeptidase 20
3453.	MTM1	Myotubularin 1
3454.	PTPN3	Protein Tyrosine Phosphatase Non-Receptor Type 3
3455.	STK39	Serine/Threonine Kinase 39
3456.	AHI1	Abelson Helper Integration Site 1
3457.	APOBEC3G	Apolipoprotein B MRNA Editing Enzyme Catalytic Subunit 3G
3458.	ATP2C2	ATPase Secretory Pathway Ca2+ Transporting 2
3459.	CCT7	Chaperonin Containing TCP1 Subunit 7
3460.	CHST14	Carbohydrate Sulfotransferase 14
3461.	CRHBP	Corticotropin Releasing Hormone Binding Protein
3462.	EFHC1	EF-Hand Domain Containing 1
3463.	ELAVL1	ELAV Like RNA Binding Protein 1

3464.	G6PC3	Glucose-6-Phosphatase Catalytic Subunit 3
3465.	GTF2H1	General Transcription Factor IIH Subunit 1
3466.	HLTF	Helicase Like Transcription Factor
3467.	HOXB13	Homeobox B13
3468.	IL17RB	Interleukin 17 Receptor B
3469.	KIF1C	Kinesin Family Member 1C
3470.	LHX3	LIM Homeobox 3
3471.	MLST8	MTOR Associated Protein, LST8 Homolog
3472.	OSTF1	Osteoclast Stimulating Factor 1
3473.	POLR2B	RNA Polymerase II Subunit B
3474.	RECQL	RecQ Like Helicase
3475.	RECQL4	RecQ Like Helicase 4
3476.	SECISBP2	SECIS Binding Protein 2
3477.	TMLHE	Trimethyllysine Hydroxylase, Epsilon
3478.	CEBPD	CCAAT Enhancer Binding Protein Delta
3479.	DKK2	Dickkopf WNT Signaling Pathway Inhibitor 2
3480.	DNASE2	Deoxyribonuclease 2, Lysosomal
3481.	LRBA	LPS Responsive Beige-Like Anchor Protein
3482.	MADCAM1	Mucosal Vascular Addressin Cell Adhesion Molecule 1
3483.	MLLT10	MLLT10 Histone Lysine Methyltransferase DOT1L Cofactor
3484.	MMRN1	Multimerin 1
3485.	MOV10	Mov10 RNA Helicase
3486.	MUS81	MUS81 Structure-Specific Endonuclease Subunit
3487.	NKX2-2	NK2 Homeobox 2
3488.	RAD51D	RAD51 Paralog D
3489.	RPL4	Ribosomal Protein L4
3490.	SIGLEC8	Sialic Acid Binding Ig Like Lectin 8
3491.	ADAM28	ADAM Metallopeptidase Domain 28
3492.	BAZ1A	Bromodomain Adjacent To Zinc Finger Domain 1A
3493.	CLC	Charcot-Leyden Crystal Galectin
3494.	CLTCL1	Clathrin Heavy Chain Like 1
3495.	DCDC2	Doublecortin Domain Containing 2
3496.	DDHD2	DDHD Domain Containing 2
3497.	EDN2	Endothelin 2
3498.	FXR2	FMR1 Autosomal Homolog 2
3499.	MTFMT	Mitochondrial Methionyl-TRNA Formyltransferase
3500.	PDE4DIP	Phosphodiesterase 4D Interacting Protein
3501.	POMP	Proteasome Maturation Protein
3502.	TNS1	Tensin 1
3503.	UBE3B	Ubiquitin Protein Ligase E3B
3504.	VPS45	Vacuolar Protein Sorting 45 Homolog

3505.	CIDEC	Cell Death Inducing DFFA Like Effector C
3506.	GYG2	Glycogenin 2
3507.	IFIT1	Interferon Induced Protein With Tetratricopeptide Repeats 1
3508.	IFT57	Intraflagellar Transport 57
3509.	PRND	Prion Like Protein Doppel
3510.	REEP2	Receptor Accessory Protein 2
3511.	TNS4	Tensin 4
3512.	UXS1	UDP-Glucuronate Decarboxylase 1
3513.	CEBPZ	CCAAT Enhancer Binding Protein Zeta
3514.	CSMD1	CUB And Sushi Multiple Domains 1
3515.	CTC1	CST Telomere Replication Complex Component 1
3516.	FTMT	Ferritin Mitochondrial
3517.	IFI30	IFI30 Lysosomal Thiol Reductase
3518.	IFT80	Intraflagellar Transport 80
3519.	KPNA6	Karyopherin Subunit Alpha 6
3520.	NDRG2	NDRG Family Member 2
3521.	PPP1R3C	Protein Phosphatase 1 Regulatory Subunit 3C
3522.	PSMG2	Proteasome Assembly Chaperone 2
3523.	RAB3IL1	RAB3A Interacting Protein Like 1
3524.	RIOK3	RIO Kinase 3
3525.	SLC35C2	Solute Carrier Family 35 Member C2
3526.	TIMM9	Translocase Of Inner Mitochondrial Membrane 9
3527.	BPIFA1	BPI Fold Containing Family A Member 1
3528.	CENPC	Centromere Protein C
3529.	CIR1	Corepressor Interacting With RBPJ, CIR1
3530.	CKS2	CDC28 Protein Kinase Regulatory Subunit 2
3531.	CLEC4E	C-Type Lectin Domain Family 4 Member E
3532.	IFNL1	Interferon Lambda 1
3533.	PLB1	Phospholipase B1
3534.	PLEK2	Pleckstrin 2
3535.	PRICKLE3	Prickle Planar Cell Polarity Protein 3
3536.	RASA3	RAS P21 Protein Activator 3
3537.	THOC5	THO Complex Subunit 5
3538.	TIMM10	Translocase Of Inner Mitochondrial Membrane 10
3539.	CDIN1	CDAN1 Interacting Nuclease 1
3540.	CLEC4D	C-Type Lectin Domain Family 4 Member D
3541.	TNS2	Tensin 2
3542.	TNS3	Tensin 3
3543.	ATP13A5	ATPase 13A5
3544.	DIRAS1	DIRAS Family GTPase 1
3545.	DPH7	Diphthamide Biosynthesis 7

3546.	EGFL6	EGF Like Domain Multiple 6
3547.	FBH1	F-Box DNA Helicase 1
3548.	HS3ST4	Heparan Sulfate-Glucosamine 3-Sulfotransferase 4
3549.	NUTM1	NUT Midline Carcinoma Family Member 1
3550.	PRDM8	PR/SET Domain 8
3551.	SLC35B3	Solute Carrier Family 35 Member B3
3552.	SLC43A3	Solute Carrier Family 43 Member 3
3553.	TMEM97	Transmembrane Protein 97
3554.	TRMO	TRNA Methyltransferase O
3555.	ZRANB3	Zinc Finger RANBP2-Type Containing 3
3556.	CAGE1	Cancer Antigen 1
3557.	CMTM7	CKLF Like MARVEL Transmembrane Domain Containing 7
3558.	JAGN1	Jagunal Homolog 1
3559.	STBD1	Starch Binding Domain 1
3560.	USB1	U6 SnRNA Biogenesis Phosphodiesterase 1
3561.	AMN1	Antagonist Of Mitotic Exit Network 1 Homolog
3562.	ARHGEF37	Rho Guanine Nucleotide Exchange Factor 37
3563.	NKAPL	NFKB Activating Protein Like
3564.	SLC35E1	Solute Carrier Family 35 Member E1
3565.	STOML3	Stomatin Like 3
3566.	ZNF2	Zinc Finger Protein 2
3567.	NSG2	Neuronal Vesicle Trafficking Associated 2
3568.	PPP1R3D	Protein Phosphatase 1 Regulatory Subunit 3D
3569.	PRDM10	PR/SET Domain 10
3570.	RSPH6A	Radial Spoke Head 6 Homolog A
3571.	SLC10A5	Solute Carrier Family 10 Member 5
3572.	TRIR	Telomerase RNA Component Interacting RNase
3573.	TXNDC16	Thioredoxin Domain Containing 16
3574.	LRRC66	Leucine Rich Repeat Containing 66
3575.	RADX	RPA1 Related Single Stranded DNA Binding Protein, X-Linked
3576.	SLC49A3	Solute Carrier Family 49 Member 3
3577.	TPTE2	Transmembrane Phosphoinositide 3-Phosphatase And Tensin Homolog 2
3578.	UTF1	Undifferentiated Embryonic Cell Transcription Factor 1
3579.	GRID2IP	Grid2 Interacting Protein
3580.	SLC35A4	Solute Carrier Family 35 Member A4
3581.	PPP1R3G	Protein Phosphatase 1 Regulatory Subunit 3G
3582.	TTC16	Tetratricopeptide Repeat Domain 16
3583.	PLGLB2	Plasminogen Like B2
3584.	PPP1R27	Protein Phosphatase 1 Regulatory Subunit 27
3585.	OR4C12	Olfactory Receptor Family 4 Subfamily C Member 12
3586.	ZSCAN23	Zinc Finger And SCAN Domain Containing 23

3587.	LUZP6	Leucine Zipper Protein 6
3588.	TSHR	Thyroid Stimulating Hormone Receptor
3589.	PFN1	Profilin 1
3590.	ANG	Angiogenin
3591.	MATR3	Matrin 3
3592.	SCAPER	S-Phase Cyclin A Associated Protein In The ER
3593.	GPD1L	Glycerol-3-Phosphate Dehydrogenase 1 Like
3594.	OGG1	8-Oxoguanine DNA Glycosylase
3595.	HNMT	Histamine N-Methyltransferase
3596.	SRF	Serum Response Factor
3597.	CD52	CD52 Molecule
3598.	DPYSL2	Dihydropyrimidinase Like 2
3599.	HDAC2	Histone Deacetylase 2
3600.	PCSK9	Proprotein Convertase Subtilisin/Kexin Type 9
3601.	HSPB8	Heat Shock Protein Family B (Small) Member 8
3602.	SYNJ1	Synaptojanin 1
3603.	FA2H	Fatty Acid 2-Hydroxylase
3604.	ATG7	Autophagy Related 7
3605.	NFASC	Neurofascin
3606.	APCS	Amyloid P Component, Serum
3607.	TOMM40	Translocase Of Outer Mitochondrial Membrane 40
3608.	CHCHD2	Coiled-Coil-Helix-Coiled-Coil-Helix Domain Containing 2
3609.	TMEM106B	Transmembrane Protein 106B
3610.	SCIN	Scinderin
3611.	LRTOMT	Leucine Rich Transmembrane And O-Methyltransferase Domain Containing
3612.	ENO1	Enolase 1
3613.	CCR6	C-C Motif Chemokine Receptor 6
3614.	CEACAM3	CEA Cell Adhesion Molecule 3
3615.	KLRD1	Killer Cell Lectin Like Receptor D1
3616.	IL1RAP	Interleukin 1 Receptor Accessory Protein
3617.	CD5	CD5 Molecule
3618.	IRF2	Interferon Regulatory Factor 2
3619.	PYCARD	PYD And CARD Domain Containing
3620.	ADD2	Adducin 2
3621.	RAB6A	RAB6A, Member RAS Oncogene Family
3622.	CRABP1	Cellular Retinoic Acid Binding Protein 1
3623.	RAB1A	RAB1A, Member RAS Oncogene Family
3624.	RAB33B	RAB33B, Member RAS Oncogene Family
3625.	HBG1	Hemoglobin Subunit Gamma 1
3626.	LAMP3	Lysosomal Associated Membrane Protein 3
3627.	RAB35	RAB35, Member RAS Oncogene Family

3628.	BZW1	Basic Leucine Zipper And W2 Domains 1
3629.	EPDR1	Ependymin Related 1
3630.	IGHM	Immunoglobulin Heavy Constant Mu
3631.	ANKLE1	Ankyrin Repeat And LEM Domain Containing 1
3632.	ASTN1	Astrotactin 1
3633.	CCDC120	Coiled-Coil Domain Containing 120
3634.	IGH	Immunoglobulin Heavy Locus
3635.	FUCA1	Alpha-L-Fucosidase 1
3636.	ALDH18A1	Aldehyde Dehydrogenase 18 Family Member A1
3637.	SRD5A3	Steroid 5 Alpha-Reductase 3
3638.	ERCC8	ERCC Excision Repair 8, CSA Ubiquitin Ligase Complex Subunit
3639.	HECW2	HECT, C2 And WW Domain Containing E3 Ubiquitin Protein Ligase 2
3640.	MICU1	Mitochondrial Calcium Uptake 1
3641.	TRAPPC9	Trafficking Protein Particle Complex Subunit 9
3642.	TUSC3	Tumor Suppressor Candidate 3
3643.	FUZ	Fuzzy Planar Cell Polarity Protein
3644.	TSEN54	TRNA Splicing Endonuclease Subunit 54
3645.	TSPAN1	Tetraspanin 1
3646.	UFSP2	UFM1 Specific Peptidase 2
3647.	WDR62	WD Repeat Domain 62
3648.	ASPM	Assembly Factor For Spindle Microtubules
3649.	RMND1	Required For Meiotic Nuclear Division 1 Homolog
3650.	TMEM94	Transmembrane Protein 94
3651.	TRMT10A	TRNA Methyltransferase 10A
3652.	CFAP96	Cilia And Flagella Associated Protein 96
3653.	PROC	Protein C, Inactivator Of Coagulation Factors Va And VIIIa
3654.	CDK8	Cyclin Dependent Kinase 8
3655.	GABBR2	Gamma-Aminobutyric Acid Type B Receptor Subunit 2
3656.	ALDH3A2	Aldehyde Dehydrogenase 3 Family Member A2
3657.	CHRNA3	Cholinergic Receptor Nicotinic Alpha 3 Subunit
3658.	ANXA11	Annexin A11
3659.	ARHGEF10	Rho Guanine Nucleotide Exchange Factor 10
3660.	GBF1	Golgi Brefeldin A Resistant Guanine Nucleotide Exchange Factor 1
3661.	GLE1	GLE1 RNA Export Mediator
3662.	NGLY1	N-Glycanase 1
3663.	PLD3	Phospholipase D Family Member 3
3664.	CTDP1	CTD Phosphatase Subunit 1
3665.	DEGS1	Delta 4-Desaturase, Sphingolipid 1
3666.	RAB3GAP1	RAB3 GTPase Activating Protein Catalytic Subunit 1
3667.	SLITRK1	SLIT And NTRK Like Family Member 1
3668.	LMO1	LIM Domain Only 1

3669.	PITRM1	Pitrilysin Metallopeptidase 1
3670.	CCNF	Cyclin F
3671.	DNAL1	Dynein Axonemal Light Chain 1
3672.	GEMIN2	Gem Nuclear Organelle Associated Protein 2
3673.	KIF7	Kinesin Family Member 7
3674.	MORC2	MORC Family CW-Type Zinc Finger 2
3675.	NME8	NME/NM23 Family Member 8
3676.	PCDH12	Protocadherin 12
3677.	TTC12	Tetratricopeptide Repeat Domain 12
3678.	CEP104	Centrosomal Protein 104
3679.	GAS8	Growth Arrest Specific 8
3680.	CFAP298	Cilia And Flagella Associated Protein 298
3681.	LSM7	LSM7 Homolog, U6 Small Nuclear RNA And MRNA Degradation Associated
3682.	DNAJB13	DnaJ Heat Shock Protein Family (Hsp40) Member B13
3683.	DRP2	Dystrophin Related Protein 2
3684.	ODAD2	Outer Dynein Arm Docking Complex Subunit 2
3685.	AFG2B	AFG2 AAA ATPase Homolog B
3686.	CCDC65	Coiled-Coil Domain Containing 65
3687.	DNAAF5	Dynein Axonemal Assembly Factor 5
3688.	RSPH3	Radial Spoke Head 3
3689.	C19orf12	Chromosome 19 Open Reading Frame 12
3690.	CASD1	CAS1 Domain Containing 1
3691.	DNAH7	Dynein Axonemal Heavy Chain 7
3692.	ODAD1	Outer Dynein Arm Docking Complex Subunit 1
3693.	CFAP300	Cilia And Flagella Associated Protein 300
3694.	ODAD4	Outer Dynein Arm Docking Complex Subunit 4
3695.	DNAAF6	Dynein Axonemal Assembly Factor 6
3696.	GNE	Glucosamine (UDP-N-Acetyl)-2-Epimerase/N-Acetylmannosamine Kinase
3697.	CAMK2A	Calcium/Calmodulin Dependent Protein Kinase II Alpha
3698.	CAMK2G	Calcium/Calmodulin Dependent Protein Kinase II Gamma
3699.	ATF6	Activating Transcription Factor 6
3700.	IDE	Insulin Degrading Enzyme
3701.	SLC12A5	Solute Carrier Family 12 Member 5
3702.	ERN1	Endoplasmic Reticulum To Nucleus Signaling 1
3703.	FGF1	Fibroblast Growth Factor 1
3704.	KEAP1	Kelch Like ECH Associated Protein 1
3705.	DVL1	Dishevelled Segment Polarity Protein 1
3706.	GHSR	Growth Hormone Secretagogue Receptor
3707.	HADH	Hydroxyacyl-CoA Dehydrogenase
3708.	XBP1	X-Box Binding Protein 1
3709.	BTD	Biotinidase

3710.	CDK5R1	Cyclin Dependent Kinase 5 Regulatory Subunit 1
3711.	IDUA	Alpha-L-Iduronidase
3712.	RBBP8	RB Binding Protein 8, Endonuclease
3713.	ULK1	Unc-51 Like Autophagy Activating Kinase 1
3714.	DNAJC6	DnaJ Heat Shock Protein Family (Hsp40) Member C6
3715.	GHRHR	Growth Hormone Releasing Hormone Receptor
3716.	SNCG	Synuclein Gamma
3717.	SUZ12	SUZ12 Polycomb Repressive Complex 2 Subunit
3718.	PRKRA	Protein Activator Of Interferon Induced Protein Kinase EIF2AK2
3719.	RNF135	Ring Finger Protein 135
3720.	RENBP	Renin Binding Protein
3721.	LGALS4	Galectin 4
3722.	MYO18A	Myosin XVIIIA
3723.	TOR1B	Torsin Family 1 Member B
3724.	HSPB3	Heat Shock Protein Family B (Small) Member 3
3725.	CIZ1	CDKN1A Interacting Zinc Finger Protein 1
3726.	CRLF3	Cytokine Receptor Like Factor 3
3727.	EVI2A	Ecotropic Viral Integration Site 2A
3728.	EVI2B	Ecotropic Viral Integration Site 2B
3729.	PRKCQ	Protein Kinase C Theta
3730.	ATIC	5-Aminoimidazole-4-Carboxamide Ribonucleotide Formyltransferase/IMP Cyclohydrolase
3731.	MAP2K2	Mitogen-Activated Protein Kinase Kinase 2
3732.	ATP2A2	ATPase Sarcoplasmic/Endoplasmic Reticulum Ca2+ Transporting 2
3733.	BLM	BLM RecQ Like Helicase
3734.	HNF4A	Hepatocyte Nuclear Factor 4 Alpha
3735.	PRDX1	Peroxiredoxin 1
3736.	PTPRF	Protein Tyrosine Phosphatase Receptor Type F
3737.	CHRNA2	Cholinergic Receptor Nicotinic Alpha 2 Subunit
3738.	F11	Coagulation Factor XI
3739.	GABRA2	Gamma-Aminobutyric Acid Type A Receptor Subunit Alpha2
3740.	GALK1	Galactokinase 1
3741.	GUSB	Glucuronidase Beta
3742.	MECOM	MDS1 And EVI1 Complex Locus
3743.	PPP2R1A	Protein Phosphatase 2 Scaffold Subunit Aalpha
3744.	RPA1	Replication Protein A1
3745.	ARHGEF2	Rho/Rac Guanine Nucleotide Exchange Factor 2
3746.	ITCH	Itchy E3 Ubiquitin Protein Ligase
3747.	MAPKAPK2	MAPK Activated Protein Kinase 2
3748.	MC4R	Melanocortin 4 Receptor
3749.	PAFAH1B1	Platelet Activating Factor Acetylhydrolase 1b Regulatory Subunit 1
3750.	SERPIND1	Serpin Family D Member 1

3751.	SLC25A12	Solute Carrier Family 25 Member 12
3752.	AOC3	Amine Oxidase Copper Containing 3
3753.	ARHGEF1	Rho Guanine Nucleotide Exchange Factor 1
3754.	EDN3	Endothelin 3
3755.	FOSL1	FOS Like 1, AP-1 Transcription Factor Subunit
3756.	GALNT3	Polypeptide N-Acetylgalactosaminyltransferase 3
3757.	GATM	Glycine Amidinotransferase
3758.	LIPC	Lipase C, Hepatic Type
3759.	NQO2	N-Ribosyldihydronicotinamide:Quinone Dehydrogenase 2
3760.	PDX1	Pancreatic And Duodenal Homeobox 1
3761.	PPIA	Peptidylprolyl Isomerase A
3762.	RIT1	Ras Like Without CAAX 1
3763.	TK1	Thymidine Kinase 1
3764.	UBE2L3	Ubiquitin Conjugating Enzyme E2 L3
3765.	GALM	Galactose Mutarotase
3766.	HMBS	Hydroxymethylbilane Synthase
3767.	MYO9B	Myosin IXB
3768.	NCL	Nucleolin
3769.	PKN1	Protein Kinase N1
3770.	PTPRB	Protein Tyrosine Phosphatase Receptor Type B
3771.	SLC5A7	Solute Carrier Family 5 Member 7
3772.	TAP2	Transporter 2, ATP Binding Cassette Subfamily B Member
3773.	XRCC5	X-Ray Repair Cross Complementing 5
3774.	APLNR	Apelin Receptor
3775.	CTNNA2	Catenin Alpha 2
3776.	FARS2	Phenylalanyl-TRNA Synthetase 2, Mitochondrial
3777.	FCER1A	Fc Epsilon Receptor Ia
3778.	GART	Phosphoribosylglycinamide Formyltransferase, Phosphoribosylglycinamide Synthetase, Phosphoribosylaminoimidazole Synthetase
3779.	GMPPB	GDP-Mannose Pyrophosphorylase B
3780.	HGD	Homogentisate 1,2-Dioxygenase
3781.	IGFBP6	Insulin Like Growth Factor Binding Protein 6
3782.	ITPKC	Inositol-Trisphosphate 3-Kinase C
3783.	MBD4	Methyl-CpG Binding Domain 4, DNA Glycosylase
3784.	NOX1	NADPH Oxidase 1
3785.	RAN	RAN, Member RAS Oncogene Family
3786.	UGT8	UDP Glycosyltransferase 8
3787.	APLP2	Amyloid Beta Precursor Like Protein 2
3788.	CCR8	C-C Motif Chemokine Receptor 8
3789.	CEP164	Centrosomal Protein 164
3790.	FBL	Fibrillarin
3791.	MYO5B	Myosin VB

3792.	TNFRSF25	TNF Receptor Superfamily Member 25
3793.	BRD1	Bromodomain Containing 1
3794.	COCH	Cochlin
3795.	GNA14	G Protein Subunit Alpha 14
3796.	GRP	Gastrin Releasing Peptide
3797.	IFT122	Intraflagellar Transport 122
3798.	MAPKAP1	MAPK Associated Protein 1
3799.	MEIS1	Meis Homeobox 1
3800.	MPDU1	Mannose-P-Dolichol Utilization Defect 1
3801.	MYOT	Myotilin
3802.	PAX4	Paired Box 4
3803.	PDGFD	Platelet Derived Growth Factor D
3804.	PLSCR1	Phospholipid Scramblase 1
3805.	RAG2	Recombination Activating 2
3806.	SCGB1A1	Secretoglobin Family 1A Member 1
3807.	STX1B	Syntaxin 1B
3808.	SYNE1	Spectrin Repeat Containing Nuclear Envelope Protein 1
3809.	TFCP2	Transcription Factor CP2
3810.	UNC13B	Unc-13 Homolog B
3811.	AFG2A	AFG2 AAA ATPase Homolog A
3812.	BBS9	Bardet-Biedl Syndrome 9
3813.	BNIP3	BCL2 Interacting Protein 3
3814.	CCP110	Centriolar Coiled-Coil Protein 110
3815.	DNAJC3	DnaJ Heat Shock Protein Family (Hsp40) Member C3
3816.	EFNB3	Ephrin B3
3817.	GABARAPL1	GABA Type A Receptor Associated Protein Like 1
3818.	KLC2	Kinesin Light Chain 2
3819.	NEU2	Neuraminidase 2
3820.	PCDH10	Protocadherin 10
3821.	POLRMT	RNA Polymerase Mitochondrial
3822.	PRPH2	Peripherin 2
3823.	VEGFD	Vascular Endothelial Growth Factor D
3824.	BIRC6	Baculoviral IAP Repeat Containing 6
3825.	CAMP	Cathelicidin Antimicrobial Peptide
3826.	CELF4	CUGBP Elav-Like Family Member 4
3827.	DLX4	Distal-Less Homeobox 4
3828.	GSTO2	Glutathione S-Transferase Omega 2
3829.	IFT27	Intraflagellar Transport 27
3830.	IK	IK Cytokine
3831.	KLF10	KLF Transcription Factor 10
3832.	LDHC	Lactate Dehydrogenase C

3833.	MSMB	Microseminoprotein Beta
3834.	NDUFA5	NADH:Ubiquinone Oxidoreductase Subunit A5
3835.	NEUROG1	Neurogenin 1
3836.	NXF1	Nuclear RNA Export Factor 1
3837.	RAB3GAP2	RAB3 GTPase Activating Non-Catalytic Protein Subunit 2
3838.	RGMA	Repulsive Guidance Molecule BMP Co-Receptor A
3839.	RPGRIP1	RPGR Interacting Protein 1
3840.	SEPTIN4	Septin 4
3841.	SHANK1	SH3 And Multiple Ankyrin Repeat Domains 1
3842.	SIGLEC7	Sialic Acid Binding Ig Like Lectin 7
3843.	SLC24A1	Solute Carrier Family 24 Member 1
3844.	TCTN3	Tectonic Family Member 3
3845.	TRIM22	Tripartite Motif Containing 22
3846.	AKT1S1	AKT1 Substrate 1
3847.	ARIH1	Ariadne RBR E3 Ubiquitin Protein Ligase 1
3848.	CALCOCO2	Calcium Binding And Coiled-Coil Domain 2
3849.	DOLK	Dolichol Kinase
3850.	DZIP1	DAZ Interacting Zinc Finger Protein 1
3851.	IPO5	Importin 5
3852.	NCR1	Natural Cytotoxicity Triggering Receptor 1
3853.	SLC41A1	Solute Carrier Family 41 Member 1
3854.	SMU1	SMU1 DNA Replication Regulator And Spliceosomal Factor
3855.	STAM	Signal Transducing Adaptor Molecule
3856.	STAU1	Staufen Double-Stranded RNA Binding Protein 1
3857.	TFPI2	Tissue Factor Pathway Inhibitor 2
3858.	TMOD2	Tropomodulin 2
3859.	WDR19	WD Repeat Domain 19
3860.	AOAH	Acyloxyacyl Hydrolase
3861.	CHD6	Chromodomain Helicase DNA Binding Protein 6
3862.	FARP1	FERM, ARH/RhoGEF And Pleckstrin Domain Protein 1
3863.	HERC5	HECT And RLD Domain Containing E3 Ubiquitin Protein Ligase 5
3864.	KIF17	Kinesin Family Member 17
3865.	MORC3	MORC Family CW-Type Zinc Finger 3
3866.	MTIF2	Mitochondrial Translational Initiation Factor 2
3867.	NCR2	Natural Cytotoxicity Triggering Receptor 2
3868.	PGLYRP1	Peptidoglycan Recognition Protein 1
3869.	POU4F2	POU Class 4 Homeobox 2
3870.	SCG2	Secretogranin II
3871.	SV2C	Synaptic Vesicle Glycoprotein 2C
3872.	TAGAP	T Cell Activation RhoGTPase Activating Protein
3873.	TBC1D20	TBC1 Domain Family Member 20

3874.	TGM6	Transglutaminase 6
3875.	B9D1	B9 Domain Containing 1
3876.	BLOC1S5	Biogenesis Of Lysosomal Organelles Complex 1 Subunit 5
3877.	CPNE4	Copine 4
3878.	DDX56	DEAD-Box Helicase 56
3879.	EDC4	Enhancer Of MRNA Decapping 4
3880.	ELP2	Elongator Acetyltransferase Complex Subunit 2
3881.	GIPC2	GIPC PDZ Domain Containing Family Member 2
3882.	GNB1L	G Protein Subunit Beta 1 Like
3883.	GOLGA3	Golgin A3
3884.	IFT52	Intraflagellar Transport 52
3885.	IPO11	Importin 11
3886.	KPNA5	Karyopherin Subunit Alpha 5
3887.	L3MBTL1	L3MBTL Histone Methyl-Lysine Binding Protein 1
3888.	MKRN1	Makorin Ring Finger Protein 1
3889.	NGB	Neuroglobin
3890.	PEX5L	Peroxisomal Biogenesis Factor 5 Like
3891.	RRP1B	Ribosomal RNA Processing 1B
3892.	VGF	VGF Nerve Growth Factor Inducible
3893.	ZBTB7A	Zinc Finger And BTB Domain Containing 7A
3894.	CD300C	CD300c Molecule
3895.	EFHD2	EF-Hand Domain Family Member D2
3896.	HES6	Hes Family BHLH Transcription Factor 6
3897.	MTCH1	Mitochondrial Carrier 1
3898.	RTRAF	RNA Transcription, Translation And Transport Factor
3899.	SCYL3	SCY1 Like Pseudokinase 3
3900.	SHQ1	SHQ1, H/ACA Ribonucleoprotein Assembly Factor
3901.	AKNA	AT-Hook Transcription Factor
3902.	CLRN3	Clarin 3
3903.	DUSP23	Dual Specificity Phosphatase 23
3904.	HTR3C	5-Hydroxytryptamine Receptor 3C
3905.	IFT46	Intraflagellar Transport 46
3906.	LINGO2	Leucine Rich Repeat And Ig Domain Containing 2
3907.	LST1	Leukocyte Specific Transcript 1
3908.	PGP	Phosphoglycolate Phosphatase
3909.	PRAM1	PML-RARA Regulated Adaptor Molecule 1
3910.	PRDM13	PR/SET Domain 13
3911.	RFX4	Regulatory Factor X4
3912.	SHKBP1	SH3KBP1 Binding Protein 1
3913.	SLC6A16	Solute Carrier Family 6 Member 16
3914.	TP53AIP1	Tumor Protein P53 Regulated Apoptosis Inducing Protein 1

3915.	TRAPPC14	Trafficking Protein Particle Complex Subunit 14
3916.	UBFD1	Ubiquitin Family Domain Containing 1
3917.	APOL3	Apolipoprotein L3
3918.	SESTD1	SEC14 And Spectrin Domain Containing 1
3919.	CCDC80	Coiled-Coil Domain Containing 80
3920.	QRICH2	Glutamine Rich 2
3921.	RETNLB	Resistin Like Beta
3922.	SDHAF3	Succinate Dehydrogenase Complex Assembly Factor 3
3923.	SPRN	Shadow Of Prion Protein
3924.	ARHGAP36	Rho GTPase Activating Protein 36
3925.	ARHGEF40	Rho Guanine Nucleotide Exchange Factor 40
3926.	XRRA1	X-Ray Radiation Resistance Associated 1
3927.	FAM184A	Family With Sequence Similarity 184 Member A
3928.	HEPACAM2	HEPACAM Family Member 2
3929.	PDF	Peptide Deformylase, Mitochondrial
3930.	TEX28	Testis Expressed 28
3931.	SERTAD3	SERTA Domain Containing 3
3932.	MAGED4B	MAGE Family Member D4B
3933.	PPP1R14D	Protein Phosphatase 1 Regulatory Inhibitor Subunit 14D
3934.	SAXO4	Stabilizer Of Axonemal Microtubules 4
3935.	YJU2B	YJU2 Splicing Factor Homolog B
3936.	GGTLC3	Gamma-Glutamyltransferase Light Chain Family Member 3
3937.	ERVK3-1	Endogenous Retrovirus Group K3 Member 1
3938.	CYP11A1	Cytochrome P450 Family 11 Subfamily A Member 1
3939.	ADRA1A	Adrenoceptor Alpha 1A
3940.	CNR2	Cannabinoid Receptor 2
3941.	CHRNA7	Cholinergic Receptor Nicotinic Alpha 7 Subunit
3942.	SUOX	Sulfite Oxidase
3943.	CHD8	Chromodomain Helicase DNA Binding Protein 8
3944.	IL32	Interleukin 32
3945.	NRGN	Neurogranin
3946.	SF1	Splicing Factor 1
3947.	TPPP3	Tubulin Polymerization Promoting Protein Family Member 3
3948.	ALDH1A2	Aldehyde Dehydrogenase 1 Family Member A2
3949.	YWHAG	Tyrosine 3-Monooxygenase/Tryptophan 5-Monooxygenase Activation Protein Gamma
3950.	MTHFD1	Methylenetetrahydrofolate Dehydrogenase, Cyclohydrolase And Formyltetrahydrofolate Synthetase 1
3951.	CD33	CD33 Molecule
3952.	F2RL1	F2R Like Trypsin Receptor 1
3953.	LPP	LIM Domain Containing Preferred Translocation Partner In Lipoma
3954.	OCA2	OCA2 Melanosomal Transmembrane Protein

3955.	TMPRSS6	Transmembrane Serine Protease 6
3956.	BCL11A	BCL11 Transcription Factor A
3957.	CASP5	Caspase 5
3958.	FOLR2	Folate Receptor Beta
3959.	VANGL2	VANGL Planar Cell Polarity Protein 2
3960.	TP53RK	TP53 Regulating Kinase
3961.	RPL10A	Ribosomal Protein L10a
3962.	SLC25A32	Solute Carrier Family 25 Member 32
3963.	ERAP2	Endoplasmic Reticulum Aminopeptidase 2
3964.	CCL19	C-C Motif Chemokine Ligand 19
3965.	CD7	CD7 Molecule
3966.	FLVCR2	FLVCR Choline And Putative Heme Transporter 2
3967.	RPL28	Ribosomal Protein L28
3968.	UBA7	Ubiquitin Like Modifier Activating Enzyme 7
3969.	EVL	Enah/Vasp-Like
3970.	PLPBP	Pyridoxal Phosphate Binding Protein
3971.	PTPN4	Protein Tyrosine Phosphatase Non-Receptor Type 4
3972.	WDR36	WD Repeat Domain 36
3973.	GSDMD	Gasdermin D
3974.	NLRP6	NLR Family Pyrin Domain Containing 6
3975.	PLEKHA7	Pleckstrin Homology Domain Containing A7
3976.	ADGRL2	Adhesion G Protein-Coupled Receptor L2
3977.	SPOCK3	SPARC (Osteonectin), Cwcv And Kazal Like Domains Proteoglycan 3
3978.	GET4	Guided Entry Of Tail-Anchored Proteins Factor 4
3979.	NOP53	NOP53 Ribosome Biogenesis Factor
3980.	SLC50A1	Solute Carrier Family 50 Member 1
3981.	SH3D19	SH3 Domain Containing 19
3982.	ZC3H12D	Zinc Finger CCCH-Type Containing 12D
3983.	OR1F1	Olfactory Receptor Family 1 Subfamily F Member 1
3984.	OARD1	O-Acyl-ADP-Ribose Deacylase 1
3985.	ACSM6	Acyl-CoA Synthetase Medium Chain Family Member 6
3986.	C1orf141	Chromosome 1 Open Reading Frame 141
3987.	C10orf53	Chromosome 10 Open Reading Frame 53
3988.	PRDX2	Peroxiredoxin 2
3989.	SLC22A12	Solute Carrier Family 22 Member 12
3990.	TXNDC9	Thioredoxin Domain Containing 9
3991.	GRIK2	Glutamate Ionotropic Receptor Kainate Type Subunit 2
3992.	SYT1	Synaptotagmin 1
3993.	APAF1	Apoptotic Peptidase Activating Factor 1
3994.	ETS1	ETS Proto-Oncogene 1, Transcription Factor
3995.	FYN	FYN Proto-Oncogene, Src Family Tyrosine Kinase

3996.	NPR2	Natriuretic Peptide Receptor 2
3997.	PRPS1	Phosphoribosyl Pyrophosphate Synthetase 1
3998.	SIRT3	Sirtuin 3
3999.	SLC9A3	Solute Carrier Family 9 Member A3
4000.	PTHLH	Parathyroid Hormone Like Hormone
4001.	MTMR2	Myotubularin Related Protein 2
4002.	PTS	6-Pyruvoyltetrahydropterin Synthase
4003.	CNTN1	Contactin 1
4004.	COL6A1	Collagen Type VI Alpha 1 Chain
4005.	EFNB1	Ephrin B1
4006.	EPX	Eosinophil Peroxidase
4007.	H2AX	H2A.X Variant Histone
4008.	PXN	Paxillin
4009.	TCF12	Transcription Factor 12
4010.	TP53BP1	Tumor Protein P53 Binding Protein 1
4011.	AVPR1B	Arginine Vasopressin Receptor 1B
4012.	CNBP	CCHC-Type Zinc Finger Nucleic Acid Binding Protein
4013.	FBXO7	F-Box Protein 7
4014.	IL11RA	Interleukin 11 Receptor Subunit Alpha
4015.	PPP1R1B	Protein Phosphatase 1 Regulatory Inhibitor Subunit 1B
4016.	DNAJB2	DnaJ Heat Shock Protein Family (Hsp40) Member B2
4017.	MDC1	Mediator Of DNA Damage Checkpoint 1
4018.	RNMT	RNA Guanine-7 Methyltransferase
4019.	SLC37A4	Solute Carrier Family 37 Member 4
4020.	TRPM7	Transient Receptor Potential Cation Channel Subfamily M Member 7
4021.	BSND	Barttin CLCNK Type Accessory Subunit Beta
4022.	CLDN16	Claudin 16
4023.	FGFRL1	Fibroblast Growth Factor Receptor Like 1
4024.	HIBCH	3-Hydroxyisobutyryl-CoA Hydrolase
4025.	TAFAZZIN	Tafazzin, Phospholipid-Lysophospholipid Transacylase
4026.	FRS2	Fibroblast Growth Factor Receptor Substrate 2
4027.	UTRN	Utrophin
4028.	ACADL	Acyl-CoA Dehydrogenase Long Chain
4029.	CCL17	C-C Motif Chemokine Ligand 17
4030.	CLCNKA	Chloride Voltage-Gated Channel Ka
4031.	DHTKD1	Dehydrogenase E1 And Transketolase Domain Containing 1
4032.	HSPB2	Heat Shock Protein Family B (Small) Member 2
4033.	NRTN	Neurturin
4034.	SNRNP70	Small Nuclear Ribonucleoprotein U1 Subunit 70
4035.	SYN2	Synapsin II
4036.	PLEKHG2	Pleckstrin Homology And RhoGEF Domain Containing G2

4037.	SACS	Sacsin Molecular Chaperone
4038.	ACBD4	Acyl-CoA Binding Domain Containing 4
4039.	VPREB1	V-Set Pre-B Cell Surrogate Light Chain 1
4040.	SSX2	SSX Family Member 2
4041.	TCTE1	T-Complex-Associated-Testis-Expressed 1
4042.	CAMTA2	Calmodulin Binding Transcription Activator 2
4043.	MROH6	Maestro Heat Like Repeat Family Member 6
4044.	TMEM107	Transmembrane Protein 107
4045.	GNAI2	G Protein Subunit Alpha I2
4046.	SATB1	SATB Homeobox 1
4047.	TACC3	Transforming Acidic Coiled-Coil Containing Protein 3
4048.	CRTAP	Cartilage Associated Protein
4049.	DNAJB6	DnaJ Heat Shock Protein Family (Hsp40) Member B6
4050.	FKBP10	FKBP Prolyl Isomerase 10
4051.	MBTPS2	Membrane Bound Transcription Factor Peptidase, Site 2
4052.	P3H1	Prolyl 3-Hydroxylase 1
4053.	RAPGEF2	Rap Guanine Nucleotide Exchange Factor 2
4054.	SMARCA5	SWI/SNF Related, Matrix Associated, Actin Dependent Regulator Of Chromatin, Subfamily A, Member 5
4055.	TMEM38B	Transmembrane Protein 38B
4056.	UNC13A	Unc-13 Homolog A
4057.	CLEC1A	C-Type Lectin Domain Family 1 Member A
4058.	WDR37	WD Repeat Domain 37
4059.	KDELR2	KDEL Endoplasmic Reticulum Protein Retention Receptor 2
4060.	MESD	Mesoderm Development LRP Chaperone
4061.	TNRC6A	Trinucleotide Repeat Containing Adaptor 6A
4062.	CCDC134	Coiled-Coil Domain Containing 134
4063.	VWA1	Von Willebrand Factor A Domain Containing 1
4064.	IFITM5	Interferon Induced Transmembrane Protein 5
4065.	PGGHG	Protein-Glucosylgalactosylhydroxylysine Glucosidase
4066.	PHLDB1	Pleckstrin Homology Like Domain Family B Member 1
4067.	TENT5A	Terminal Nucleotidyltransferase 5A
4068.	AXDND1	Axonemal Dynein Light Chain Domain Containing 1
4069.	SYCE2	Synaptonemal Complex Central Element Protein 2
4070.	MILR1	Mast Cell Immunoglobulin Like Receptor 1
4071.	SEPTIN14	Septin 14
4072.	ATXN8	Ataxin 8
4073.	COL7A1	Collagen Type VII Alpha 1 Chain
4074.	ELP4	Elongator Acetyltransferase Complex Subunit 4
4075.	MLNR	Motilin Receptor
4076.	SPN	Sialophorin
4077.	CABIN1	Calcineurin Binding Protein 1

4078.	WDR48	WD Repeat Domain 48
4079.	COL6A5	Collagen Type VI Alpha 5 Chain
4080.	HCN4	Hyperpolarization Activated Cyclic Nucleotide Gated Potassium Channel 4
4081.	KCNJ2	Potassium Inwardly Rectifying Channel Subfamily J Member 2
4082.	PRKAG2	Protein Kinase AMP-Activated Non-Catalytic Subunit Gamma 2
4083.	DSC2	Desmocollin 2
4084.	SNTA1	Syntrophin Alpha 1
4085.	TBX5	T-Box Transcription Factor 5
4086.	BCL10	BCL10 Immune Signaling Adaptor
4087.	CASQ2	Calsequestrin 2
4088.	HYOU1	Hypoxia Up-Regulated 1
4089.	NUP155	Nucleoporin 155
4090.	SLC6A5	Solute Carrier Family 6 Member 5
4091.	CITED2	Cbp/P300 Interacting Transactivator With Glu/Asp Rich Carboxy-Terminal Domain 2
4092.	AKAP9	A-Kinase Anchoring Protein 9
4093.	BVES	Blood Vessel Epicardial Substance
4094.	MYL4	Myosin Light Chain 4
4095.	TBX20	T-Box Transcription Factor 20
4096.	TNNC1	Troponin C1, Slow Skeletal And Cardiac Type
4097.	MGAT2	Alpha-1,6-Mannosyl-Glycoprotein 2-Beta-N-Acetylglucosaminyltransferase
4098.	MYPN	Myopalladin
4099.	RFX5	Regulatory Factor X5
4100.	SRCAP	Snf2 Related CREBBP Activator Protein
4101.	GATA5	GATA Binding Protein 5
4102.	NOS1AP	Nitric Oxide Synthase 1 Adaptor Protein
4103.	RFXANK	Regulatory Factor X Associated Ankyrin Containing Protein
4104.	SLC4A3	Solute Carrier Family 4 Member 3
4105.	NCKAP1L	NCK Associated Protein 1 Like
4106.	DBR1	Debranching RNA Lariats 1
4107.	RFXAP	Regulatory Factor X Associated Protein
4108.	RNF125	Ring Finger Protein 125
4109.	TECRL	Trans-2,3-Enoyl-CoA Reductase Like
4110.	KCNE5	Potassium Voltage-Gated Channel Subfamily E Regulatory Subunit 5
4111.	RANGRF	RAN Guanine Nucleotide Release Factor
4112.	ALG10B	ALG10 Alpha-1,2-Glucosyltransferase B
4113.	ABAT	4-Aminobutyrate Aminotransferase
4114.	AVPR1A	Arginine Vasopressin Receptor 1A
4115.	GABRA3	Gamma-Aminobutyric Acid Type A Receptor Subunit Alpha3
4116.	DPAGT1	Dolichyl-Phosphate N-Acetylglucosaminephosphotransferase 1
4117.	FSHB	Follicle Stimulating Hormone Subunit Beta
4118.	PSMA2	Proteasome 20S Subunit Alpha 2

4119.	PTPA	Protein Phosphatase 2 Phosphatase Activator
4120.	SRD5A1	Steroid 5 Alpha-Reductase 1
4121.	CACNA2D2	Calcium Voltage-Gated Channel Auxiliary Subunit Alpha2delta 2
4122.	DYSF	Dysferlin
4123.	COIL	Coilin
4124.	OPRD1	Opioid Receptor Delta 1
4125.	OPRK1	Opioid Receptor Kappa 1
4126.	OPA3	Outer Mitochondrial Membrane Lipid Metabolism Regulator OPA3
4127.	INTS5	Integrator Complex Subunit 5
4128.	CAPN1	Calpain 1
4129.	NCSTN	Nicastrin
4130.	CASP6	Caspase 6
4131.	UBE3A	Ubiquitin Protein Ligase E3A
4132.	ALDH5A1	Aldehyde Dehydrogenase 5 Family Member A1
4133.	FKBP1A	FKBP Prolyl Isomerase 1A
4134.	IDS	Iduronate 2-Sulfatase
4135.	RAB7A	RAB7A, Member RAS Oncogene Family
4136.	SIRT2	Sirtuin 2
4137.	AMPD1	Adenosine Monophosphate Deaminase 1
4138.	HTRA2	HtrA Serine Peptidase 2
4139.	LMNB1	Lamin B1
4140.	SLC6A8	Solute Carrier Family 6 Member 8
4141.	TUBB2A	Tubulin Beta 2A Class IIa
4142.	UBE2N	Ubiquitin Conjugating Enzyme E2 N
4143.	ACOX1	Acyl-CoA Oxidase 1
4144.	CFL1	Cofilin 1
4145.	CYSLTR2	Cysteinyl Leukotriene Receptor 2
4146.	EIF2S1	Eukaryotic Translation Initiation Factor 2 Subunit Alpha
4147.	HADHB	Hydroxyacyl-CoA Dehydrogenase Trifunctional Multienzyme Complex Subunit Beta
4148.	NDUFS3	NADH:Ubiquinone Oxidoreductase Core Subunit S3
4149.	NHERF1	NHERF Family PDZ Scaffold Protein 1
4150.	TGM1	Transglutaminase 1
4151.	ABCA7	ATP Binding Cassette Subfamily A Member 7
4152.	ABCD3	ATP Binding Cassette Subfamily D Member 3
4153.	ADARB1	Adenosine Deaminase RNA Specific B1
4154.	ATF1	Activating Transcription Factor 1
4155.	BAD	BCL2 Associated Agonist Of Cell Death
4156.	BTRC	Beta-Transducin Repeat Containing E3 Ubiquitin Protein Ligase
4157.	FGD4	FYVE, RhoGEF And PH Domain Containing 4
4158.	HAAO	3-Hydroxyanthranilate 3,4-Dioxygenase
4159.	NAGA	Alpha-N-Acetylgalactosaminidase

4160.	NLGN3	Neuroligin 3
4161.	PICALM	Phosphatidylinositol Binding Clathrin Assembly Protein
4162.	PSMD4	Proteasome 26S Subunit Ubiquitin Receptor, Non-ATPase 4
4163.	RAG1	Recombination Activating 1
4164.	S1PR1	Sphingosine-1-Phosphate Receptor 1
4165.	ACADSB	Acyl-CoA Dehydrogenase Short/Branched Chain
4166.	APH1A	Aph-1 Homolog A, Gamma-Secretase Subunit
4167.	BST1	Bone Marrow Stromal Cell Antigen 1
4168.	ETFB	Electron Transfer Flavoprotein Subunit Beta
4169.	GFM1	G Elongation Factor Mitochondrial 1
4170.	LATS1	Large Tumor Suppressor Kinase 1
4171.	LATS2	Large Tumor Suppressor Kinase 2
4172.	LGR5	Leucine Rich Repeat Containing G Protein-Coupled Receptor 5
4173.	NDUFA12	NADH:Ubiquinone Oxidoreductase Subunit A12
4174.	NLGN4X	Neuroligin 4 X-Linked
4175.	NPC2	NPC Intracellular Cholesterol Transporter 2
4176.	PAX7	Paired Box 7
4177.	PEX5	Peroxisomal Biogenesis Factor 5
4178.	PEX7	Peroxisomal Biogenesis Factor 7
4179.	RHOT1	Ras Homolog Family Member T1
4180.	RIPK3	Receptor Interacting Serine/Threonine Kinase 3
4181.	SFPQ	Splicing Factor Proline And Glutamine Rich
4182.	SLC18A3	Solute Carrier Family 18 Member A3
4183.	SREBF2	Sterol Regulatory Element Binding Transcription Factor 2
4184.	TYROBP	Transmembrane Immune Signaling Adaptor TYROBP
4185.	UBQLN1	Ubiquilin 1
4186.	YARS1	Tyrosyl-TRNA Synthetase 1
4187.	CCNA1	Cyclin A1
4188.	CDH17	Cadherin 17
4189.	GDF11	Growth Differentiation Factor 11
4190.	ID2	Inhibitor Of DNA Binding 2
4191.	IVD	Isovaleryl-CoA Dehydrogenase
4192.	MLKL	Mixed Lineage Kinase Domain Like Pseudokinase
4193.	NDUFS6	NADH:Ubiquinone Oxidoreductase Subunit S6
4194.	PLS3	Plastin 3
4195.	PSMC4	Proteasome 26S Subunit, ATPase 4
4196.	ABCD2	ATP Binding Cassette Subfamily D Member 2
4197.	ACD	ACD Shelterin Complex Subunit And Telomerase Recruitment Factor
4198.	ARHGEF9	Cdc42 Guanine Nucleotide Exchange Factor 9
4199.	BLVRB	Biliverdin Reductase B
4200.	CD58	CD58 Molecule

4201.	HOXD13	Homeobox D13
4202.	MMADHC	Metabolism Of Cobalamin Associated D
4203.	NDUFAF4	NADH:Ubiquinone Oxidoreductase Complex Assembly Factor 4
4204.	PAWR	Pro-Apoptotic WT1 Regulator
4205.	PEX10	Peroxisomal Biogenesis Factor 10
4206.	PLEKHM1	Pleckstrin Homology And RUN Domain Containing M1
4207.	RAPSN	Receptor Associated Protein Of The Synapse
4208.	SDCBP	Syndecan Binding Protein
4209.	TTPA	Alpha Tocopherol Transfer Protein
4210.	ADARB2	Adenosine Deaminase RNA Specific B2 (Inactive)
4211.	APOC1	Apolipoprotein C1
4212.	ASGR1	Asialoglycoprotein Receptor 1
4213.	HPCA	Hippocalcin
4214.	MC3R	Melanocortin 3 Receptor
4215.	NDUFAF2	NADH:Ubiquinone Oxidoreductase Complex Assembly Factor 2
4216.	NDUFB3	NADH:Ubiquinone Oxidoreductase Subunit B3
4217.	POLR3K	RNA Polymerase III Subunit K
4218.	REG1A	Regenerating Family Member 1 Alpha
4219.	WWC1	WW And C2 Domain Containing 1
4220.	COLQ	Collagen Like Tail Subunit Of Asymmetric Acetylcholinesterase
4221.	DLX3	Distal-Less Homeobox 3
4222.	ETV5	ETS Variant Transcription Factor 5
4223.	FOXRED1	FAD Dependent Oxidoreductase Domain Containing 1
4224.	H2BC21	H2B Clustered Histone 21
4225.	LSM2	LSM2 Homolog, U6 Small Nuclear RNA And MRNA Degradation Associated
4226.	MBNL1	Muscleblind Like Splicing Regulator 1
4227.	MPZL1	Myelin Protein Zero Like 1
4228.	NBAS	NBAS Subunit Of NRZ Tethering Complex
4229.	NLRP5	NLR Family Pyrin Domain Containing 5
4230.	NRXN2	Neurexin 2
4231.	PEX12	Peroxisomal Biogenesis Factor 12
4232.	PEX26	Peroxisomal Biogenesis Factor 26
4233.	SUN2	Sad1 And UNC84 Domain Containing 2
4234.	TACO1	Translational Activator Of Cytochrome C Oxidase I
4235.	TUBGCP2	Tubulin Gamma Complex Component 2
4236.	AADAT	Aminoadipate Aminotransferase
4237.	APBB3	Amyloid Beta Precursor Protein Binding Family B Member 3
4238.	EXOSC10	Exosome Component 10
4239.	MFF	Mitochondrial Fission Factor
4240.	MMAA	Metabolism Of Cobalamin Associated A
4241.	PBX2	PBX Homeobox 2

4242.	PEX13	Peroxisomal Biogenesis Factor 13
4243.	RAB39B	RAB39B, Member RAS Oncogene Family
4244.	ZFYVE26	Zinc Finger FYVE-Type Containing 26
4245.	AMOT	Angiomotin
4246.	APOBEC1	Apolipoprotein B MRNA Editing Enzyme Catalytic Subunit 1
4247.	MMEL1	Membrane Metalloendopeptidase Like 1
4248.	MOB1A	MOB Kinase Activator 1A
4249.	NDUFA11	NADH:Ubiquinone Oxidoreductase Subunit A11
4250.	SPG21	SPG21 Abhydrolase Domain Containing, Maspardin
4251.	TBC1D7	TBC1 Domain Family Member 7
4252.	BTBD9	BTB Domain Containing 9
4253.	CCNL2	Cyclin L2
4254.	EPN1	Epsin 1
4255.	FXYD1	FXYD Domain Containing Ion Transport Regulator 1
4256.	HLF	HLF Transcription Factor, PAR BZIP Family Member
4257.	NDUFAF5	NADH:Ubiquinone Oxidoreductase Complex Assembly Factor 5
4258.	RBM26	RNA Binding Motif Protein 26
4259.	ZC4H2	Zinc Finger C4H2-Type Containing
4260.	ACMSD	Aminocarboxymuconate Semialdehyde Decarboxylase
4261.	DAND5	DAN Domain BMP Antagonist Family Member 5
4262.	LAS1L	LAS1 Like Ribosome Biogenesis Factor
4263.	NDUFAF6	NADH:Ubiquinone Oxidoreductase Complex Assembly Factor 6
4264.	PDZD2	PDZ Domain Containing 2
4265.	SAV1	Salvador Family WW Domain Containing Protein 1
4266.	TMEM230	Transmembrane Protein 230
4267.	ZNRF3	Zinc And Ring Finger 3
4268.	CNKSR3	CNKSR Family Member 3
4269.	GATD3	Glutamine Amidotransferase Class 1 Domain Containing 3
4270.	RNF112	Ring Finger Protein 112
4271.	SDHAF1	Succinate Dehydrogenase Complex Assembly Factor 1
4272.	SLCO6A1	Solute Carrier Organic Anion Transporter Family Member 6A1
4273.	TMEM30B	Transmembrane Protein 30B
4274.	CORT	Cortistatin
4275.	DEF8	Differentially Expressed In FDCP 8 Homolog
4276.	MTMR8	Myotubularin Related Protein 8
4277.	TOR1AIP2	Torsin 1A Interacting Protein 2
4278.	ZNF330	Zinc Finger Protein 330
4279.	MPND	MPN Domain Containing
4280.	MORN1	MORN Repeat Containing 1
4281.	NLRP13	NLR Family Pyrin Domain Containing 13
4282.	ASB12	Ankyrin Repeat And SOCS Box Containing 12

4283.	MAP6D1	MAP6 Domain Containing 1
4284.	TP53TG5	TP53 Target 5
4285.	CTXN3	Cortexin 3
4286.	DUX4	Double Homeobox 4
4287.	RERGL	RERG Like
4288.	NBPF9	NBPF Member 9
4289.	SAA2-SAA4	SAA2-SAA4 Readthrough
4290.	NBPF19	NBPF Member 19
4291.	LOC128031833	Uncharacterized LOC128031833
4292.	PTGDR	Prostaglandin D2 Receptor
4293.	CYSLTR1	Cysteinyl Leukotriene Receptor 1
4294.	PPAT	Phosphoribosyl Pyrophosphate Amidotransferase
4295.	PDE4C	Phosphodiesterase 4C
4296.	SVEP1	Sushi, Von Willebrand Factor Type A, EGF And Pentraxin Domain Containing 1
4297.	TOP6BL	TOP6B Like Initiator Of Meiotic Double Strand Breaks
4298.	ZAP70	Zeta Chain Of T Cell Receptor Associated Protein Kinase 70
4299.	PIM1	Pim-1 Proto-Oncogene, Serine/Threonine Kinase
4300.	PRKD1	Protein Kinase D1
4301.	BRIP1	BRCA1 Interacting Helicase 1
4302.	ABCC6	ATP Binding Cassette Subfamily C Member 6
4303.	FANCC	FA Complementation Group C
4304.	KCNN4	Potassium Calcium-Activated Channel Subfamily N Member 4
4305.	LCAT	Lecithin-Cholesterol Acyltransferase
4306.	RPS19	Ribosomal Protein S19
4307.	FAH	Fumarylacetoacetate Hydrolase
4308.	FANCL	FA Complementation Group L
4309.	IL17RA	Interleukin 17 Receptor A
4310.	MS4A1	Membrane Spanning 4-Domains A1
4311.	NPR1	Natriuretic Peptide Receptor 1
4312.	ADCY6	Adenylate Cyclase 6
4313.	ANTXR2	ANTXR Cell Adhesion Molecule 2
4314.	BIRC3	Baculoviral IAP Repeat Containing 3
4315.	CD79B	CD79b Molecule
4316.	FUT2	Fucosyltransferase 2 (H Blood Group)
4317.	GUCY2D	Guanylate Cyclase 2D, Retinal
4318.	RARG	Retinoic Acid Receptor Gamma
4319.	RDH5	Retinol Dehydrogenase 5
4320.	CHD4	Chromodomain Helicase DNA Binding Protein 4
4321.	FAM20C	FAM20C Golgi Associated Secretory Pathway Kinase
4322.	GRM6	Glutamate Metabotropic Receptor 6
4323.	LTBP1	Latent Transforming Growth Factor Beta Binding Protein 1

4324.	PTPRN	Protein Tyrosine Phosphatase Receptor Type N
4325.	REV3L	REV3 Like, DNA Directed Polymerase Zeta Catalytic Subunit
4326.	STEAP3	STEAP3 Metalloreductase
4327.	TFR2	Transferrin Receptor 2
4328.	CYP26B1	Cytochrome P450 Family 26 Subfamily B Member 1
4329.	DVL2	Dishevelled Segment Polarity Protein 2
4330.	FANCG	FA Complementation Group G
4331.	HJV	Hemojuvelin BMP Co-Receptor
4332.	IL3RA	Interleukin 3 Receptor Subunit Alpha
4333.	MLXIPL	MLX Interacting Protein Like
4334.	PRKCSH	PRKCSH Beta Subunit Of Glucosidase II
4335.	PTK7	Protein Tyrosine Kinase 7 (Inactive)
4336.	SEC63	SEC63 Homolog, Protein Translocation Regulator
4337.	SOD3	Superoxide Dismutase 3
4338.	ADAMTS10	ADAM Metallopeptidase With Thrombospondin Type 1 Motif 10
4339.	ALG8	ALG8 Alpha-1,3-Glucosyltransferase
4340.	ATG16L1	Autophagy Related 16 Like 1
4341.	FANCE	FA Complementation Group E
4342.	GZMA	Granzyme A
4343.	LOXL1	Lysyl Oxidase Like 1
4344.	NLRP2	NLR Family Pyrin Domain Containing 2
4345.	NOD1	Nucleotide Binding Oligomerization Domain Containing 1
4346.	PDCD1LG2	Programmed Cell Death 1 Ligand 2
4347.	PMPCA	Peptidase, Mitochondrial Processing Subunit Alpha
4348.	RASGRF1	Ras Protein Specific Guanine Nucleotide Releasing Factor 1
4349.	RPS3	Ribosomal Protein S3
4350.	RTEL1	Regulator Of Telomere Elongation Helicase 1
4351.	SDC1	Syndecan 1
4352.	TRPM1	Transient Receptor Potential Cation Channel Subfamily M Member 1
4353.	ACKR3	Atypical Chemokine Receptor 3
4354.	CD164	CD164 Molecule
4355.	CNGB3	Cyclic Nucleotide Gated Channel Subunit Beta 3
4356.	FANCF	FA Complementation Group F
4357.	FAR1	Fatty Acyl-CoA Reductase 1
4358.	HEPH	Hephaestin
4359.	LOXL3	Lysyl Oxidase Like 3
4360.	OSGEP	O-Sialoglycoprotein Endopeptidase
4361.	PKD2L1	Polycystin 2 Like 1, Transient Receptor Potential Cation Channel
4362.	SLC23A2	Solute Carrier Family 23 Member 2
4363.	SPEG	Striated Muscle Enriched Protein Kinase
4364.	WNT9B	Wnt Family Member 9B

4365.	ACAA1	Acetyl-CoA Acyltransferase 1
4366.	GUCY1B1	Guanylate Cyclase 1 Soluble Subunit Beta 1
4367.	PARD3	Par-3 Family Cell Polarity Regulator
4368.	SERPINB3	Serpin Family B Member 3
4369.	STRA6	Signaling Receptor And Transporter Of Retinol STRA6
4370.	CCRL2	C-C Motif Chemokine Receptor Like 2
4371.	CPSF1	Cleavage And Polyadenylation Specific Factor 1
4372.	DACT1	Dishevelled Binding Antagonist Of Beta Catenin 1
4373.	ELF4	E74 Like ETS Transcription Factor 4
4374.	GJD2	Gap Junction Protein Delta 2
4375.	LUM	Lumican
4376.	MYO1C	Myosin IC
4377.	NFE2	Nuclear Factor, Erythroid 2
4378.	PARS2	Prolyl-TRNA Synthetase 2, Mitochondrial
4379.	PCBP2	Poly(RC) Binding Protein 2
4380.	RBP1	Retinol Binding Protein 1
4381.	SLC25A37	Solute Carrier Family 25 Member 37
4382.	SLC30A1	Solute Carrier Family 30 Member 1
4383.	STEAP1	STEAP Family Member 1
4384.	TMCO1	Transmembrane And Coiled-Coil Domains 1
4385.	ADAMTS17	ADAM Metallopeptidase With Thrombospondin Type 1 Motif 17
4386.	COG5	Component Of Oligomeric Golgi Complex 5
4387.	DTX1	Deltex E3 Ubiquitin Ligase 1
4388.	HAS1	Hyaluronan Synthase 1
4389.	JDP2	Jun Dimerization Protein 2
4390.	MOXD1	Monooxygenase DBH Like 1
4391.	NEK7	NIMA Related Kinase 7
4392.	OBSCN	Obscurin, Cytoskeletal Calmodulin And Titin-Interacting RhoGEF
4393.	ORM1	Orosomucoid 1
4394.	PCLO	Piccolo Presynaptic Cytomatrix Protein
4395.	STEAP2	STEAP2 Metalloreductase
4396.	THSD1	Thrombospondin Type 1 Domain Containing 1
4397.	ARHGEF17	Rho Guanine Nucleotide Exchange Factor 17
4398.	CXCL6	C-X-C Motif Chemokine Ligand 6
4399.	GRHL3	Grainyhead Like Transcription Factor 3
4400.	LRIT3	Leucine Rich Repeat, Ig-Like And Transmembrane Domains 3
4401.	NLRC5	NLR Family CARD Domain Containing 5
4402.	NYX	Nyctalopin
4403.	ORM2	Orosomucoid 2
4404.	PIDD1	P53-Induced Death Domain Protein 1
4405.	RBP2	Retinol Binding Protein 2

4406.	SCARA5	Scavenger Receptor Class A Member 5
4407.	SDR16C5	Short Chain Dehydrogenase/Reductase Family 16C Member 5
4408.	SEC61B	SEC61 Translocon Subunit Beta
4409.	SRRT	Serrate, RNA Effector Molecule
4410.	SUMO4	Small Ubiquitin Like Modifier 4
4411.	TGOLN2	Trans-Golgi Network Protein 2
4412.	TRAM1	Translocation Associated Membrane Protein 1
4413.	TRIM31	Tripartite Motif Containing 31
4414.	ACCS	1-Aminocyclopropane-1-Carboxylate Synthase Homolog (Inactive)
4415.	BCO1	Beta-Carotene Oxygenase 1
4416.	COX17	Cytochrome C Oxidase Copper Chaperone COX17
4417.	CTRB1	Chymotrypsinogen B1
4418.	CXXC5	CXXC Finger Protein 5
4419.	GPS1	G Protein Pathway Suppressor 1
4420.	IFNLR1	Interferon Lambda Receptor 1
4421.	MYRF	Myelin Regulatory Factor
4422.	REXO4	REX4 Homolog, 3'-5' Exonuclease
4423.	RNF144B	Ring Finger Protein 144B
4424.	RPL36	Ribosomal Protein L36
4425.	SELENOP	Selenoprotein P
4426.	SHROOM3	Shroom Family Member 3
4427.	SLC25A28	Solute Carrier Family 25 Member 28
4428.	SLC31A2	Solute Carrier Family 31 Member 2
4429.	SLC38A3	Solute Carrier Family 38 Member 3
4430.	DTX3L	Deltex E3 Ubiquitin Ligase 3L
4431.	DZIP1L	DAZ Interacting Zinc Finger Protein 1 Like
4432.	EMILIN2	Elastin Microfibril Interfacer 2
4433.	FBXL5	F-Box And Leucine Rich Repeat Protein 5
4434.	KIR2DL1	Killer Cell Immunoglobulin Like Receptor, Two Ig Domains And Long Cytoplasmic Tail 1
4435.	PKD2L2	Polycystin 2 Like 2, Transient Receptor Potential Cation Channel
4436.	TMCC2	Transmembrane And Coiled-Coil Domain Family 2
4437.	TXNRD3	Thioredoxin Reductase 3
4438.	WRNIP1	WRN Helicase Interacting Protein 1
4439.	ADAT3	Adenosine Deaminase TRNA Specific 3
4440.	CHMP6	Charged Multivesicular Body Protein 6
4441.	COL20A1	Collagen Type XX Alpha 1 Chain
4442.	DCAF13	DDB1 And CUL4 Associated Factor 13
4443.	ZXDC	ZXD Family Zinc Finger C
4444.	CHTOP	Chromatin Target Of PRMT1
4445.	DHFR2	Dihydrofolate Reductase 2
4446.	DNAJC24	DnaJ Heat Shock Protein Family (Hsp40) Member C24

4447.	NLRP11	NLR Family Pyrin Domain Containing 11
4448.	SLC48A1	Solute Carrier Family 48 Member 1
4449.	CTRB2	Chymotrypsinogen B2
4450.	ERFE	Erythroferrone
4451.	PKDREJ	Polycystin Family Receptor For Egg Jelly
4452.	PTTG2	Pituitary Tumor-Transforming 2
4453.	ZNF200	Zinc Finger Protein 200
4454.	ANGPTL8	Angiopoietin Like 8
4455.	DPH3	Diphthamide Biosynthesis 3
4456.	PRPF40B	Pre-MRNA Processing Factor 40 Homolog B
4457.	SLC66A2	Solute Carrier Family 66 Member 2
4458.	BROX	BRO1 Domain And CAAX Motif Containing
4459.	CCDC174	Coiled-Coil Domain Containing 174
4460.	CHCHD5	Coiled-Coil-Helix-Coiled-Coil-Helix Domain Containing 5
4461.	CLEC17A	C-Type Lectin Domain Containing 17A
4462.	CYS1	Cystin 1
4463.	DEFB118	Defensin Beta 118
4464.	CMC4	C-X9-C Motif Containing 4
4465.	DCANP1	Dendritic Cell Associated Nuclear Protein 1
4466.	PKD1L3	Polycystin 1 Like 3, Transient Receptor Potential Channel Interacting
4467.	SMTNL1	Smoothelin Like 1
4468.	CRIP3	Cysteine Rich Protein 3
4469.	ISX	Intestine Specific Homeobox
4470.	OR4C16	Olfactory Receptor Family 4 Subfamily C Member 16
4471.	OR51I1	Olfactory Receptor Family 51 Subfamily I Member 1
4472.	RNF222	Ring Finger Protein 222
4473.	C19orf33	Chromosome 19 Open Reading Frame 33
4474.	DEFB127	Defensin Beta 127
4475.	ZNF613	Zinc Finger Protein 613
4476.	OR1L1	Olfactory Receptor Family 1 Subfamily L Member 1
4477.	OR10G6	Olfactory Receptor Family 10 Subfamily G Member 6
4478.	CCER2	Coiled-Coil Glutamate Rich Protein 2
4479.	PMF1-BGLAP	PMF1-BGLAP Readthrough
4480.	ZC3H11B	Zinc Finger CCCH-Type Containing 11B
4481.	ATR	ATR Serine/Threonine Kinase
4482.	HK1	Hexokinase 1
4483.	LDHA	Lactate Dehydrogenase A
4484.	MMP13	Matrix Metallopeptidase 13
4485.	PRKDC	Protein Kinase, DNA-Activated, Catalytic Subunit
4486.	RPS6KA1	Ribosomal Protein S6 Kinase A1
4487.	SCD	Stearoyl-CoA Desaturase

4488.	TBXA2R	Thromboxane A2 Receptor
4489.	DNM1	Dynamin 1
4490.	GLS	Glutaminase
4491.	GSK3A	Glycogen Synthase Kinase 3 Alpha
4492.	PRKAA2	Protein Kinase AMP-Activated Catalytic Subunit Alpha 2
4493.	ATP2A1	ATPase Sarcoplasmic/Endoplasmic Reticulum Ca2+ Transporting 1
4494.	CPT1A	Carnitine Palmitoyltransferase 1A
4495.	MAP3K5	Mitogen-Activated Protein Kinase Kinase Kinase 5
4496.	MRE11	MRE11 Homolog, Double Strand Break Repair Nuclease
4497.	PIKFYVE	Phosphoinositide Kinase, FYVE-Type Zinc Finger Containing
4498.	CAPN2	Calpain 2
4499.	CAST	Calpastatin
4500.	CSNK1E	Casein Kinase 1 Epsilon
4501.	ESRRB	Estrogen Related Receptor Beta
4502.	GNRHR	Gonadotropin Releasing Hormone Receptor
4503.	HSPA9	Heat Shock Protein Family A (Hsp70) Member 9
4504.	MMP8	Matrix Metallopeptidase 8
4505.	PDP1	Pyruvate Dehydrogenase Phosphatase Catalytic Subunit 1
4506.	PRMT1	Protein Arginine Methyltransferase 1
4507.	TUBA4A	Tubulin Alpha 4a
4508.	UBE2I	Ubiquitin Conjugating Enzyme E2 I
4509.	ATF4	Activating Transcription Factor 4
4510.	CARD11	Caspase Recruitment Domain Family Member 11
4511.	COMP	Cartilage Oligomeric Matrix Protein
4512.	CYP27A1	Cytochrome P450 Family 27 Subfamily A Member 1
4513.	HCN1	Hyperpolarization Activated Cyclic Nucleotide Gated Potassium Channel 1
4514.	HSPA1A	Heat Shock Protein Family A (Hsp70) Member 1A
4515.	HUWE1	HECT, UBA And WWE Domain Containing E3 Ubiquitin Protein Ligase 1
4516.	IL5RA	Interleukin 5 Receptor Subunit Alpha
4517.	IRF5	Interferon Regulatory Factor 5
4518.	LHCGR	Luteinizing Hormone/Choriogonadotropin Receptor
4519.	NFATC2	Nuclear Factor Of Activated T Cells 2
4520.	OGDH	Oxoglutarate Dehydrogenase
4521.	PLCB3	Phospholipase C Beta 3
4522.	SLC2A9	Solute Carrier Family 2 Member 9
4523.	SPTLC2	Serine Palmitoyltransferase Long Chain Base Subunit 2
4524.	SUMO1	Small Ubiquitin Like Modifier 1
4525.	CTSC	Cathepsin C
4526.	FADD	Fas Associated Via Death Domain
4527.	GABRA4	Gamma-Aminobutyric Acid Type A Receptor Subunit Alpha4
4528.	GRM4	Glutamate Metabotropic Receptor 4

4529.	GRM7	Glutamate Metabotropic Receptor 7
4530.	HSD3B2	Hydroxy-Delta-5-Steroid Dehydrogenase, 3 Beta- And Steroid Delta-Isomerase 2
4531.	IRS2	Insulin Receptor Substrate 2
4532.	LONP1	Lon Peptidase 1, Mitochondrial
4533.	MMUT	Methylmalonyl-CoA Mutase
4534.	PRSS8	Serine Protease 8
4535.	PSMA6	Proteasome 20S Subunit Alpha 6
4536.	PSMB1	Proteasome 20S Subunit Beta 1
4537.	PSPH	Phosphoserine Phosphatase
4538.	PTPRD	Protein Tyrosine Phosphatase Receptor Type D
4539.	RAB11A	RAB11A, Member RAS Oncogene Family
4540.	RTN4R	Reticulon 4 Receptor
4541.	UQCRFS1	Ubiquinol-Cytochrome C Reductase, Rieske Iron-Sulfur Polypeptide 1
4542.	YWHAH	Tyrosine 3-Monooxygenase/Tryptophan 5-Monooxygenase Activation Protein Eta
4543.	ABCB6	ATP Binding Cassette Subfamily B Member 6 (LAN Blood Group)
4544.	BGN	Biglycan
4545.	CD82	CD82 Molecule
4546.	CDH13	Cadherin 13
4547.	CHD1	Chromodomain Helicase DNA Binding Protein 1
4548.	COL6A2	Collagen Type VI Alpha 2 Chain
4549.	CTPS1	CTP Synthase 1
4550.	DNAJB1	DnaJ Heat Shock Protein Family (Hsp40) Member B1
4551.	EFEMP2	EGF Containing Fibulin Extracellular Matrix Protein 2
4552.	ETV1	ETS Variant Transcription Factor 1
4553.	FPR1	Formyl Peptide Receptor 1
4554.	GRM3	Glutamate Metabotropic Receptor 3
4555.	GRM8	Glutamate Metabotropic Receptor 8
4556.	GSTO1	Glutathione S-Transferase Omega 1
4557.	ILK	Integrin Linked Kinase
4558.	ISCU	Iron-Sulfur Cluster Assembly Enzyme
4559.	KLK6	Kallikrein Related Peptidase 6
4560.	KMO	Kynurenine 3-Monooxygenase
4561.	NCOR1	Nuclear Receptor Corepressor 1
4562.	NFS1	NFS1 Cysteine Desulfurase
4563.	NT5C3A	5'-Nucleotidase, Cytosolic IIIA
4564.	PCBD1	Pterin-4 Alpha-Carbinolamine Dehydratase 1
4565.	PLOD3	Procollagen-Lysine,2-Oxoglutarate 5-Dioxygenase 3
4566.	PPIF	Peptidylprolyl Isomerase F
4567.	PPP3R1	Protein Phosphatase 3 Regulatory Subunit B, Alpha
4568.	PSENEN	Presenilin Enhancer, Gamma-Secretase Subunit
4569.	RNASET2	Ribonuclease T2

4570.	SFTPA1	Surfactant Protein A1
4571.	SLC27A2	Solute Carrier Family 27 Member 2
4572.	STAR	Steroidogenic Acute Regulatory Protein
4573.	TDP1	Tyrosyl-DNA Phosphodiesterase 1
4574.	TLE1	TLE Family Member 1, Transcriptional Corepressor
4575.	TNFRSF4	TNF Receptor Superfamily Member 4
4576.	TUBA1B	Tubulin Alpha 1b
4577.	TUBB4B	Tubulin Beta 4B Class IVb
4578.	UBB	Ubiquitin B
4579.	USF1	Upstream Transcription Factor 1
4580.	ADAMTS4	ADAM Metallopeptidase With Thrombospondin Type 1 Motif 4
4581.	AIMP2	Aminoacyl TRNA Synthetase Complex Interacting Multifunctional Protein 2
4582.	AP2M1	Adaptor Related Protein Complex 2 Subunit Mu 1
4583.	APBB1	Amyloid Beta Precursor Protein Binding Family B Member 1
4584.	FBXW11	F-Box And WD Repeat Domain Containing 11
4585.	G6PC1	Glucose-6-Phosphatase Catalytic Subunit 1
4586.	GAK	Cyclin G Associated Kinase
4587.	HSD3B1	Hydroxy-Delta-5-Steroid Dehydrogenase, 3 Beta- And Steroid Delta-Isomerase 1
4588.	IFI16	Interferon Gamma Inducible Protein 16
4589.	LEFTY2	Left-Right Determination Factor 2
4590.	MOCS2	Molybdenum Cofactor Synthesis 2
4591.	PDIA3	Protein Disulfide Isomerase Family A Member 3
4592.	PER2	Period Circadian Regulator 2
4593.	PSMA5	Proteasome 20S Subunit Alpha 5
4594.	RCAN1	Regulator Of Calcineurin 1
4595.	SKIC2	SKI2 Subunit Of Superkiller Complex
4596.	SLC26A2	Solute Carrier Family 26 Member 2
4597.	SLC32A1	Solute Carrier Family 32 Member 1
4598.	SOX3	SRY-Box Transcription Factor 3
4599.	SYT2	Synaptotagmin 2
4600.	ZYX	Zyxin
4601.	CCS	Copper Chaperone For Superoxide Dismutase
4602.	CHD3	Chromodomain Helicase DNA Binding Protein 3
4603.	CHL1	Cell Adhesion Molecule L1 Like
4604.	COPA	COPI Coat Complex Subunit Alpha
4605.	CTNNA3	Catenin Alpha 3
4606.	DLG2	Discs Large MAGUK Scaffold Protein 2
4607.	DOCK3	Dedicator Of Cytokinesis 3
4608.	EHHADH	Enoyl-CoA Hydratase And 3-Hydroxyacyl CoA Dehydrogenase
4609.	ETV4	ETS Variant Transcription Factor 4
4610.	FLII	FLII Actin Remodeling Protein

4611.	HAX1	HCLS1 Associated Protein X-1
4612.	HIP1	Huntingtin Interacting Protein 1
4613.	IGBP1	Immunoglobulin Binding Protein 1
4614.	JUND	JunD Proto-Oncogene, AP-1 Transcription Factor Subunit
4615.	KRT13	Keratin 13
4616.	MCCC1	Methylcrotonyl-CoA Carboxylase Subunit 1
4617.	NPTX2	Neuronal Pentraxin 2
4618.	P4HA1	Prolyl 4-Hydroxylase Subunit Alpha 1
4619.	POSTN	Periostin
4620.	PSMA1	Proteasome 20S Subunit Alpha 1
4621.	PSMB10	Proteasome 20S Subunit Beta 10
4622.	PSMC1	Proteasome 26S Subunit, ATPase 1
4623.	PSMC5	Proteasome 26S Subunit, ATPase 5
4624.	PSMD3	Proteasome 26S Subunit, Non-ATPase 3
4625.	PTGDR2	Prostaglandin D2 Receptor 2
4626.	RNF8	Ring Finger Protein 8
4627.	SBF1	SET Binding Factor 1
4628.	SHANK2	SH3 And Multiple Ankyrin Repeat Domains 2
4629.	TRADD	TNFRSF1A Associated Via Death Domain
4630.	TRHR	Thyrotropin Releasing Hormone Receptor
4631.	UBTF	Upstream Binding Transcription Factor
4632.	VPS11	VPS11 Core Subunit Of CORVET And HOPS Complexes
4633.	XYLT1	Xylosyltransferase 1
4634.	ADAMTS2	ADAM Metallopeptidase With Thrombospondin Type 1 Motif 2
4635.	ATP5F1E	ATP Synthase F1 Subunit Epsilon
4636.	BCL2A1	BCL2 Related Protein A1
4637.	CHM	CHM Rab Escort Protein
4638.	CLDN14	Claudin 14
4639.	COL10A1	Collagen Type X Alpha 1 Chain
4640.	DDX17	DEAD-Box Helicase 17
4641.	DPF2	Double PHD Fingers 2
4642.	FXYD2	FXYD Domain Containing Ion Transport Regulator 2
4643.	GFRA3	GDNF Family Receptor Alpha 3
4644.	GIGYF2	GRB10 Interacting GYF Protein 2
4645.	GLUD2	Glutamate Dehydrogenase 2
4646.	GPAA1	Glycosylphosphatidylinositol Anchor Attachment 1
4647.	MRPS16	Mitochondrial Ribosomal Protein S16
4648.	PARP4	Poly(ADP-Ribose) Polymerase Family Member 4
4649.	PBK	PDZ Binding Kinase
4650.	PSMD1	Proteasome 26S Subunit, Non-ATPase 1
4651.	PSMD9	Proteasome 26S Subunit, Non-ATPase 9

4652.	PTPRU	Protein Tyrosine Phosphatase Receptor Type U
4653.	RAB10	RAB10, Member RAS Oncogene Family
4654.	RHBDF2	Rhomboid 5 Homolog 2
4655.	SEPTIN9	Septin 9
4656.	SERPINB1	Serpin Family B Member 1
4657.	SRGAP1	SLIT-ROBO Rho GTPase Activating Protein 1
4658.	TFF3	Trefoil Factor 3
4659.	TIMM8A	Translocase Of Inner Mitochondrial Membrane 8A
4660.	TNNT1	Troponin T1, Slow Skeletal Type
4661.	TRIP11	Thyroid Hormone Receptor Interactor 11
4662.	APBA1	Amyloid Beta Precursor Protein Binding Family A Member 1
4663.	ARTN	Artemin
4664.	COL12A1	Collagen Type XII Alpha 1 Chain
4665.	D2HGDH	D-2-Hydroxyglutarate Dehydrogenase
4666.	DGKQ	Diacylglycerol Kinase Theta
4667.	EIF6	Eukaryotic Translation Initiation Factor 6
4668.	ELOVL1	ELOVL Fatty Acid Elongase 1
4669.	HEPACAM	Hepatic And Glial Cell Adhesion Molecule
4670.	LPO	Lactoperoxidase
4671.	MAGT1	Magnesium Transporter 1
4672.	OTOF	Otoferlin
4673.	PANK1	Pantothenate Kinase 1
4674.	RNASE2	Ribonuclease A Family Member 2
4675.	RNF19A	Ring Finger Protein 19A, RBR E3 Ubiquitin Protein Ligase
4676.	SIGLEC1	Sialic Acid Binding Ig Like Lectin 1
4677.	SLC39A13	Solute Carrier Family 39 Member 13
4678.	SMPD3	Sphingomyelin Phosphodiesterase 3
4679.	VPS29	VPS29 Retromer Complex Component
4680.	VPS41	VPS41 Subunit Of HOPS Complex
4681.	VSNL1	Visinin Like 1
4682.	ACSBG1	Acyl-CoA Synthetase Bubblegum Family Member 1
4683.	B3GALT6	Beta-1,3-Galactosyltransferase 6
4684.	B4GALT7	Beta-1,4-Galactosyltransferase 7
4685.	BRD7	Bromodomain Containing 7
4686.	CAMTA1	Calmodulin Binding Transcription Activator 1
4687.	COLGALT1	Collagen Beta(1-O)Galactosyltransferase 1
4688.	DHX36	DEAH-Box Helicase 36
4689.	FBXO32	F-Box Protein 32
4690.	GALNT12	Polypeptide N-Acetylgalactosaminyltransferase 12
4691.	HAP1	Huntingtin Associated Protein 1
4692.	IFIT3	Interferon Induced Protein With Tetratricopeptide Repeats 3

4693.	KLB	Klotho Beta
4694.	KLK13	Kallikrein Related Peptidase 13
4695.	LMBRD1	LMBR1 Domain Containing 1
4696.	LMOD3	Leiomodin 3
4697.	LRRK1	Leucine Rich Repeat Kinase 1
4698.	MLANA	Melan-A
4699.	MT3	Metallothionein 3
4700.	NOL3	Nucleolar Protein 3
4701.	NPAS2	Neuronal PAS Domain Protein 2
4702.	NRL	Neural Retina Leucine Zipper
4703.	OXSR1	Oxidative Stress Responsive Kinase 1
4704.	PHF1	PHD Finger Protein 1
4705.	RBBP5	RB Binding Protein 5, Histone Lysine Methyltransferase Complex Subunit
4706.	RPN2	Ribophorin II
4707.	RTN2	Reticulon 2
4708.	SEC24B	SEC24 Homolog B, COPII Coat Complex Component
4709.	TCTN2	Tectonic Family Member 2
4710.	TOMM20	Translocase Of Outer Mitochondrial Membrane 20
4711.	TPPP	Tubulin Polymerization Promoting Protein
4712.	TSC22D3	TSC22 Domain Family Member 3
4713.	USF2	Upstream Transcription Factor 2, C-Fos Interacting
4714.	VPS26A	VPS26 Retromer Complex Component A
4715.	AFAP1	Actin Filament Associated Protein 1
4716.	AP4S1	Adaptor Related Protein Complex 4 Subunit Sigma 1
4717.	ARFGAP3	ADP Ribosylation Factor GTPase Activating Protein 3
4718.	ATCAY	ATCAY Kinesin Light Chain Interacting Caytaxin
4719.	ATPAF2	ATP Synthase Mitochondrial F1 Complex Assembly Factor 2
4720.	CD207	CD207 Molecule
4721.	CEACAM8	CEA Cell Adhesion Molecule 8
4722.	COA6	Cytochrome C Oxidase Assembly Factor 6
4723.	CREB3L2	CAMP Responsive Element Binding Protein 3 Like 2
4724.	DLGAP3	DLG Associated Protein 3
4725.	DNAJA2	DnaJ Heat Shock Protein Family (Hsp40) Member A2
4726.	DSPP	Dentin Sialophosphoprotein
4727.	FBXO3	F-Box Protein 3
4728.	IMMP2L	Inner Mitochondrial Membrane Peptidase Subunit 2
4729.	KCNJ16	Potassium Inwardly Rectifying Channel Subfamily J Member 16
4730.	KRT12	Keratin 12
4731.	KRT3	Keratin 3
4732.	KYAT1	Kynurenine Aminotransferase 1
4733.	NFYC	Nuclear Transcription Factor Y Subunit Gamma

4734.	PLA2R1	Phospholipase A2 Receptor 1
4735.	RETREG1	Reticulophagy Regulator 1
4736.	RNGTT	RNA Guanylyltransferase And 5'-Phosphatase
4737.	SERPINB9	Serpin Family B Member 9
4738.	SFTPA2	Surfactant Protein A2
4739.	SOCS6	Suppressor Of Cytokine Signaling 6
4740.	SOSTDC1	Sclerostin Domain Containing 1
4741.	SSTR4	Somatostatin Receptor 4
4742.	SUPV3L1	Suv3 Like RNA Helicase
4743.	TEKT3	Tektin 3
4744.	TIMM17A	Translocase Of Inner Mitochondrial Membrane 17A
4745.	TKTL1	Transketolase Like 1
4746.	TP53I3	Tumor Protein P53 Inducible Protein 3
4747.	TTC19	Tetratricopeptide Repeat Domain 19
4748.	UBR5	Ubiquitin Protein Ligase E3 Component N-Recognin 5
4749.	XPO5	Exportin 5
4750.	AP2A1	Adaptor Related Protein Complex 2 Subunit Alpha 1
4751.	ARSK	Arylsulfatase Family Member K
4752.	AZIN1	Antizyme Inhibitor 1
4753.	BRD9	Bromodomain Containing 9
4754.	DENR	Density Regulated Re-Initiation And Release Factor
4755.	DLGAP2	DLG Associated Protein 2
4756.	EMP3	Epithelial Membrane Protein 3 (MAM Blood Group)
4757.	FRMD4A	FERM Domain Containing 4A
4758.	GH2	Growth Hormone 2
4759.	GNLY	Granulysin
4760.	IFIT2	Interferon Induced Protein With Tetratricopeptide Repeats 2
4761.	ISYNA1	Inositol-3-Phosphate Synthase 1
4762.	LRP10	LDL Receptor Related Protein 10
4763.	LYRM4	LYR Motif Containing 4
4764.	LYRM7	LYR Motif Containing 7
4765.	MAEA	Macrophage Erythroblast Attacher, E3 Ubiquitin Ligase
4766.	MBNL2	Muscleblind Like Splicing Regulator 2
4767.	MIA3	MIA SH3 Domain ER Export Factor 3
4768.	MUC16	Mucin 16, Cell Surface Associated
4769.	OAS2	2'-5'-Oligoadenylate Synthetase 2
4770.	PIPOX	Pipecolic Acid And Sarcosine Oxidase
4771.	RTN4IP1	Reticulon 4 Interacting Protein 1
4772.	SH3GL2	SH3 Domain Containing GRB2 Like 2, Endophilin A1
4773.	SLC25A17	Solute Carrier Family 25 Member 17
4774.	SS18	SS18 Subunit Of BAF Chromatin Remodeling Complex

4775.	TMED1	Transmembrane P24 Trafficking Protein 1
4776.	TSSK1B	Testis Specific Serine Kinase 1B
4777.	ACER3	Alkaline Ceramidase 3
4778.	ADPRH	ADP-Ribosylarginine Hydrolase
4779.	ATL2	Atlastin GTPase 2
4780.	ATP6V1E2	ATPase H+ Transporting V1 Subunit E2
4781.	CELF5	CUGBP Elav-Like Family Member 5
4782.	COX14	Cytochrome C Oxidase Assembly Factor COX14
4783.	CSNK1A1L	Casein Kinase 1 Alpha 1 Like
4784.	DIP2B	Disco Interacting Protein 2 Homolog B
4785.	DNAH10	Dynein Axonemal Heavy Chain 10
4786.	DNAH2	Dynein Axonemal Heavy Chain 2
4787.	DRAP1	DR1 Associated Protein 1
4788.	FNBP1	Formin Binding Protein 1
4789.	GORAB	Golgin, RAB6 Interacting
4790.	H2AC20	H2A Clustered Histone 20
4791.	MOBP	Myelin Associated Oligodendrocyte Basic Protein
4792.	NDOR1	NADPH Dependent Diflavin Oxidoreductase 1
4793.	P3H4	Prolyl 3-Hydroxylase Family Member 4 (Inactive)
4794.	PITPNM1	Phosphatidylinositol Transfer Protein Membrane Associated 1
4795.	PNCK	Pregnancy Up-Regulated Nonubiquitous CaM Kinase
4796.	POLR3C	RNA Polymerase III Subunit C
4797.	PRDM5	PR/SET Domain 5
4798.	PRPF40A	Pre-MRNA Processing Factor 40 Homolog A
4799.	PRPS1L1	Phosphoribosyl Pyrophosphate Synthetase 1 Like 1
4800.	PRUNE2	Prune Homolog 2 With BCH Domain
4801.	RILPL1	Rab Interacting Lysosomal Protein Like 1
4802.	SFXN2	Sideroflexin 2
4803.	SPIB	Spi-B Transcription Factor
4804.	SRGN	Serglycin
4805.	TAF12	TATA-Box Binding Protein Associated Factor 12
4806.	TMEM38A	Transmembrane Protein 38A
4807.	UTP6	UTP6 Small Subunit Processome Component
4808.	VPS16	VPS16 Core Subunit Of CORVET And HOPS Complexes
4809.	ZFYVE27	Zinc Finger FYVE-Type Containing 27
4810.	AP2A2	Adaptor Related Protein Complex 2 Subunit Alpha 2
4811.	ARFGAP2	ADP Ribosylation Factor GTPase Activating Protein 2
4812.	ATAD5	ATPase Family AAA Domain Containing 5
4813.	CNNM3	Cyclin And CBS Domain Divalent Metal Cation Transport Mediator 3
4814.	GLOD4	Glyoxalase Domain Containing 4
4815.	GTDC1	Glycosyltransferase Like Domain Containing 1

4816.	IFI44	Interferon Induced Protein 44
4817.	IFNK	Interferon Kappa
4818.	ITFG1	Integrin Alpha FG-GAP Repeat Containing 1
4819.	KIAA0586	KIAA0586
4820.	MRPL2	Mitochondrial Ribosomal Protein L2
4821.	MRPL46	Mitochondrial Ribosomal Protein L46
4822.	MUC7	Mucin 7, Secreted
4823.	NID2	Nidogen 2
4824.	NIPSNAP3B	Nipsnap Homolog 3B
4825.	PHACTR2	Phosphatase And Actin Regulator 2
4826.	PSPN	Persephin
4827.	RRAGB	Ras Related GTP Binding B
4828.	SLC9C1	Solute Carrier Family 9 Member C1
4829.	TAPT1	Transmembrane Anterior Posterior Transformation 1
4830.	TGDS	TDP-Glucose 4,6-Dehydratase
4831.	TRIM13	Tripartite Motif Containing 13
4832.	TTLL4	Tubulin Tyrosine Ligase Like 4
4833.	TTYH1	Tweety Family Member 1
4834.	WNK2	WNK Lysine Deficient Protein Kinase 2
4835.	ADAP2	ArfGAP With Dual PH Domains 2
4836.	AHCTF1	AT-Hook Containing Transcription Factor 1
4837.	CARMIL2	Capping Protein Regulator And Myosin 1 Linker 2
4838.	CCNB3	Cyclin B3
4839.	CREB3L4	CAMP Responsive Element Binding Protein 3 Like 4
4840.	CSMD3	CUB And Sushi Multiple Domains 3
4841.	DNHD1	Dynein Heavy Chain Domain 1
4842.	ETV7	ETS Variant Transcription Factor 7
4843.	FKBP11	FKBP Prolyl Isomerase 11
4844.	FRA10AC1	FRA10A Associated CGG Repeat 1
4845.	HBQ1	Hemoglobin Subunit Theta 1
4846.	MRPL42	Mitochondrial Ribosomal Protein L42
4847.	NOMO1	NODAL Modulator 1
4848.	NUP35	Nucleoporin 35
4849.	RAB29	RAB29, Member RAS Oncogene Family
4850.	STRA8	Stimulated By Retinoic Acid 8
4851.	TEFM	Transcription Elongation Factor, Mitochondrial
4852.	TMEM175	Transmembrane Protein 175
4853.	TTLL6	Tubulin Tyrosine Ligase Like 6
4854.	ATXN7L2	Ataxin 7 Like 2
4855.	CLUH	Clustered Mitochondria Homolog
4856.	GDAP1L1	Ganglioside Induced Differentiation Associated Protein 1 Like 1

4857.	KCNV1	Potassium Voltage-Gated Channel Modifier Subfamily V Member 1
4858.	KIF27	Kinesin Family Member 27
4859.	RND1	Rho Family GTPase 1
4860.	TRIM56	Tripartite Motif Containing 56
4861.	ANKS4B	Ankyrin Repeat And Sterile Alpha Motif Domain Containing 4B
4862.	FAM8A1	Family With Sequence Similarity 8 Member A1
4863.	H2AC11	H2A Clustered Histone 11
4864.	H2AC4	H2A Clustered Histone 4
4865.	IFI44L	Interferon Induced Protein 44 Like
4866.	IFNE	Interferon Epsilon
4867.	MALSU1	Mitochondrial Assembly Of Ribosomal Large Subunit 1
4868.	MPV17L2	MPV17 Mitochondrial Inner Membrane Protein Like 2
4869.	NOMO2	NODAL Modulator 2
4870.	PDCD2L	Programmed Cell Death 2 Like
4871.	PLBD2	Phospholipase B Domain Containing 2
4872.	RAB11FIP4	RAB11 Family Interacting Protein 4
4873.	RNASEK	Ribonuclease K
4874.	SRCIN1	SRC Kinase Signaling Inhibitor 1
4875.	SSX1	SSX Family Member 1
4876.	TIMM21	Translocase Of Inner Mitochondrial Membrane 21
4877.	TRIM58	Tripartite Motif Containing 58
4878.	UAP1L1	UDP-N-Acetylglucosamine Pyrophosphorylase 1 Like 1
4879.	ZNF446	Zinc Finger Protein 446
4880.	ZNF490	Zinc Finger Protein 490
4881.	CCDC14	Coiled-Coil Domain Containing 14
4882.	COPRS	Coordinator Of PRMT5 And Differentiation Stimulator
4883.	CREG2	Cellular Repressor Of E1A Stimulated Genes 2
4884.	DALRD3	DALR Anticodon Binding Domain Containing 3
4885.	DMRTB1	DMRT Like Family B With Proline Rich C-Terminal 1
4886.	FAM149B1	Family With Sequence Similarity 149 Member B1
4887.	FAXDC2	Fatty Acid Hydroxylase Domain Containing 2
4888.	GAS2L3	Growth Arrest Specific 2 Like 3
4889.	H2AC1	H2A Clustered Histone 1
4890.	H2AC13	H2A Clustered Histone 13
4891.	H2AC25	H2A Clustered Histone 25
4892.	H2AC6	H2A Clustered Histone 6
4893.	LENG1	Leukocyte Receptor Cluster Member 1
4894.	MARVELD1	MARVEL Domain Containing 1
4895.	SYCN	Syncollin
4896.	TAF1D	TATA-Box Binding Protein Associated Factor, RNA Polymerase I Subunit D
4897.	BEAN1	Brain Expressed Associated With NEDD4 1

4898.	H2AC12	H2A Clustered Histone 12
4899.	H2AC16	H2A Clustered Histone 16
4900.	HAGHL	Hydroxyacylglutathione Hydrolase Like
4901.	LRRC37B	Leucine Rich Repeat Containing 37B
4902.	NOMO3	NODAL Modulator 3
4903.	OR10J1	Olfactory Receptor Family 10 Subfamily J Member 1
4904.	PCP2	Purkinje Cell Protein 2
4905.	TLCD3A	TLC Domain Containing 3A
4906.	TM2D2	TM2 Domain Containing 2
4907.	ZCCHC3	Zinc Finger CCHC-Type Containing 3
4908.	ZNF706	Zinc Finger Protein 706
4909.	ZNF713	Zinc Finger Protein 713
4910.	ATP5MJ	ATP Synthase Membrane Subunit J
4911.	F8A1	Coagulation Factor VIII Associated 1
4912.	H2AC14	H2A Clustered Histone 14
4913.	H2AC17	H2A Clustered Histone 17
4914.	TMEM177	Transmembrane Protein 177
4915.	ZNF232	Zinc Finger Protein 232
4916.	C11orf68	Chromosome 11 Open Reading Frame 68
4917.	H2AC15	H2A Clustered Histone 15
4918.	H2AC18	H2A Clustered Histone 18
4919.	H2AC21	H2A Clustered Histone 21
4920.	NXPH2	Neurexophilin 2
4921.	C1orf94	Chromosome 1 Open Reading Frame 94
4922.	CFAP161	Cilia And Flagella Associated Protein 161
4923.	OPRPN	Opiorphin Prepropeptide
4924.	RSPH10B	Radial Spoke Head 10 Homolog B
4925.	SCGB1D1	Secretoglobin Family 1D Member 1
4926.	HMSD	Histocompatibility Minor Serpin Domain Containing
4927.	OR2T34	Olfactory Receptor Family 2 Subfamily T Member 34
4928.	PPAN-P2RY11	PPAN-P2RY11 Readthrough
4929.	H2AC19	H2A Clustered Histone 19
4930.	TMEM244	Transmembrane Protein 244
4931.	ARL14EPL	ADP Ribosylation Factor Like GTPase 14 Effector Protein Like
4932.	FXYD6-FXYD2	FXYD6-FXYD2 Readthrough
4933.	EPHA4	EPH Receptor A4
4934.	NR1H3	Nuclear Receptor Subfamily 1 Group H Member 3
4935.	SGPL1	Sphingosine-1-Phosphate Lyase 1
4936.	TNK2	Tyrosine Kinase Non Receptor 2
4937.	EMD	Emerin
4938.	KIF5B	Kinesin Family Member 5B

4939.	SATB2	SATB Homeobox 2
4940.	DAO	D-Amino Acid Oxidase
4941.	MED12	Mediator Complex Subunit 12
4942.	MYO5A	Myosin VA
4943.	PDHX	Pyruvate Dehydrogenase Complex Component X
4944.	PON3	Paraoxonase 3
4945.	SEPSECS	Sep (O-Phosphoserine) TRNA:Sec (Selenocysteine) TRNA Synthase
4946.	ARHGAP31	Rho GTPase Activating Protein 31
4947.	GM2A	Ganglioside GM2 Activator
4948.	LDB3	LIM Domain Binding 3
4949.	SLC25A24	Solute Carrier Family 25 Member 24
4950.	TBR1	T-Box Brain Transcription Factor 1
4951.	TBX18	T-Box Transcription Factor 18
4952.	TFG	Trafficking From ER To Golgi Regulator
4953.	ADGRL1	Adhesion G Protein-Coupled Receptor L1
4954.	CC2D1A	Coiled-Coil And C2 Domain Containing 1A
4955.	CDK20	Cyclin Dependent Kinase 20
4956.	DHX30	DExH-Box Helicase 30
4957.	KMT2E	Lysine Methyltransferase 2E (Inactive)
4958.	NBEA	Neurobeachin
4959.	PIGQ	Phosphatidylinositol Glycan Anchor Biosynthesis Class Q
4960.	RNF14	Ring Finger Protein 14
4961.	ALMS1	ALMS1 Centrosome And Basal Body Associated Protein
4962.	GOLGA2	Golgin A2
4963.	HACE1	HECT Domain And Ankyrin Repeat Containing E3 Ubiquitin Protein Ligase 1
4964.	OTUD6B	OTU Deubiquitinase 6B
4965.	TRIM2	Tripartite Motif Containing 2
4966.	UBE4A	Ubiquitination Factor E4A
4967.	ARF3	ADP Ribosylation Factor 3
4968.	BRWD1	Bromodomain And WD Repeat Domain Containing 1
4969.	CHAF1B	Chromatin Assembly Factor 1 Subunit B
4970.	DCTN2	Dynactin Subunit 2
4971.	MCM3AP	Minichromosome Maintenance Complex Component 3 Associated Protein
4972.	MED25	Mediator Complex Subunit 25
4973.	PPM1K	Protein Phosphatase, Mg2+/Mn2+ Dependent 1K
4974.	SYNE2	Spectrin Repeat Containing Nuclear Envelope Protein 2
4975.	USP11	Ubiquitin Specific Peptidase 11
4976.	MAGEL2	MAGE Family Member L2
4977.	SETD1B	SET Domain Containing 1B, Histone Lysine Methyltransferase
4978.	SMNDC1	Survival Motor Neuron Domain Containing 1
4979.	SPTLC3	Serine Palmitoyltransferase Long Chain Base Subunit 3

4980.	TCF20	Transcription Factor 20
4981.	TMEM63A	Transmembrane Protein 63A
4982.	U2AF2	U2 Small Nuclear RNA Auxiliary Factor 2
4983.	GLT8D1	Glycosyltransferase 8 Domain Containing 1
4984.	NME5	NME/NM23 Family Member 5
4985.	TTC14	Tetratricopeptide Repeat Domain 14
4986.	C12orf57	Chromosome 12 Open Reading Frame 57
4987.	HIVEP1	HIVEP Zinc Finger 1
4988.	MRPL39	Mitochondrial Ribosomal Protein L39
4989.	NEK10	NIMA Related Kinase 10
4990.	SPEF2	Sperm Flagellar 2
4991.	TANC2	Tetratricopeptide Repeat, Ankyrin Repeat And Coiled-Coil Containing 2
4992.	ZDHHC15	Zinc Finger DHHC-Type Palmitoyltransferase 15
4993.	CFAP410	Cilia And Flagella Associated Protein 410
4994.	FBXO38	F-Box Protein 38
4995.	ROGDI	Rogdi Atypical Leucine Zipper
4996.	SCAF4	SR-Related CTD Associated Factor 4
4997.	CEP128	Centrosomal Protein 128
4998.	DNAJB5	DnaJ Heat Shock Protein Family (Hsp40) Member B5
4999.	THTPA	Thiamine Triphosphatase
5000.	GAS2L2	Growth Arrest Specific 2 Like 2
5001.	DAW1	Dynein Assembly Factor With WD Repeats 1
5002.	FERRY3	FERRY Endosomal RAB5 Effector Complex Subunit 3
5003.	RNF217	Ring Finger Protein 217
5004.	CCDC181	Coiled-Coil Domain Containing 181
5005.	CFAP221	Cilia And Flagella Associated Protein 221
5006.	CFAP57	Cilia And Flagella Associated Protein 57
5007.	CFAP74	Cilia And Flagella Associated Protein 74
5008.	C2orf88	Chromosome 2 Open Reading Frame 88
5009.	SMIM10L2A	Small Integral Membrane Protein 10 Like 2A
5010.	CCDC196	Coiled-Coil Domain Containing 196
5011.	RXRA	Retinoid X Receptor Alpha
5012.	RXRG	Retinoid X Receptor Gamma
5013.	GABRQ	Gamma-Aminobutyric Acid Type A Receptor Subunit Theta
5014.	MMP17	Matrix Metallopeptidase 17
5015.	MARCHF4	Membrane Associated Ring-CH-Type Finger 4
5016.	PCSK1	Proprotein Convertase Subtilisin/Kexin Type 1
5017.	CD247	CD247 Molecule
5018.	CHRM3	Cholinergic Receptor Muscarinic 3
5019.	PBX1	PBX Homeobox 1
5020.	CPE	Carboxypeptidase E

5021.	EEF2	Eukaryotic Translation Elongation Factor 2
5022.	FGG	Fibrinogen Gamma Chain
5023.	INPPL1	Inositol Polyphosphate Phosphatase Like 1
5024.	OAT	Ornithine Aminotransferase
5025.	PDE3A	Phosphodiesterase 3A
5026.	TAP1	Transporter 1, ATP Binding Cassette Subfamily B Member
5027.	CIT	Citron Rho-Interacting Serine/Threonine Kinase
5028.	DKC1	Dyskerin Pseudouridine Synthase 1
5029.	EEF1A2	Eukaryotic Translation Elongation Factor 1 Alpha 2
5030.	FLNB	Filamin B
5031.	GPC3	Glypican 3
5032.	IRAK3	Interleukin 1 Receptor Associated Kinase 3
5033.	MAP2K6	Mitogen-Activated Protein Kinase Kinase 6
5034.	PTPN2	Protein Tyrosine Phosphatase Non-Receptor Type 2
5035.	REL	REL Proto-Oncogene, NF-KB Subunit
5036.	SLC2A4	Solute Carrier Family 2 Member 4
5037.	ADH5	Alcohol Dehydrogenase 5 (Class III), Chi Polypeptide
5038.	ARHGAP26	Rho GTPase Activating Protein 26
5039.	ATF2	Activating Transcription Factor 2
5040.	CEBPA	CCAAT Enhancer Binding Protein Alpha
5041.	CEL	Carboxyl Ester Lipase
5042.	DLK1	Delta Like Non-Canonical Notch Ligand 1
5043.	DSG2	Desmoglein 2
5044.	GABRD	Gamma-Aminobutyric Acid Type A Receptor Subunit Delta
5045.	GMNN	Geminin DNA Replication Inhibitor
5046.	IRF8	Interferon Regulatory Factor 8
5047.	KAT6A	Lysine Acetyltransferase 6A
5048.	LAMB2	Laminin Subunit Beta 2
5049.	PARP2	Poly(ADP-Ribose) Polymerase 2
5050.	PRCP	Prolylcarboxypeptidase
5051.	TCF7L2	Transcription Factor 7 Like 2
5052.	TEC	Tec Protein Tyrosine Kinase
5053.	ANTXR1	ANTXR Cell Adhesion Molecule 1
5054.	APOA2	Apolipoprotein A2
5055.	AQP3	Aquaporin 3 (Gill Blood Group)
5056.	ATP6AP2	ATPase H+ Transporting Accessory Protein 2
5057.	CRYAA	Crystallin Alpha A
5058.	HLA-DRA	Major Histocompatibility Complex, Class II, DR Alpha
5059.	IL21	Interleukin 21
5060.	MAT1A	Methionine Adenosyltransferase 1A
5061.	MCM5	Minichromosome Maintenance Complex Component 5

5062.	NAT1	N-Acetyltransferase 1
5063.	NRF1	Nuclear Respiratory Factor 1
5064.	PTPRN2	Protein Tyrosine Phosphatase Receptor Type N2
5065.	RORB	RAR Related Orphan Receptor B
5066.	SLC34A1	Solute Carrier Family 34 Member 1
5067.	SMAD1	SMAD Family Member 1
5068.	SOX5	SRY-Box Transcription Factor 5
5069.	TNKS	Tankyrase
5070.	TSPAN7	Tetraspanin 7
5071.	UBC	Ubiquitin C
5072.	A4GALT	Alpha 1,4-Galactosyltransferase (P1PK Blood Group)
5073.	CBLB	Cbl Proto-Oncogene B
5074.	COQ7	Coenzyme Q7, Hydroxylase
5075.	CRBN	Cereblon
5076.	FABP3	Fatty Acid Binding Protein 3
5077.	FCN3	Ficolin 3
5078.	GNAT1	G Protein Subunit Alpha Transducin 1
5079.	GNAT2	G Protein Subunit Alpha Transducin 2
5080.	KCND2	Potassium Voltage-Gated Channel Subfamily D Member 2
5081.	LAMA5	Laminin Subunit Alpha 5
5082.	MATK	Megakaryocyte-Associated Tyrosine Kinase
5083.	NEUROD1	Neuronal Differentiation 1
5084.	NUP214	Nucleoporin 214
5085.	OSM	Oncostatin M
5086.	PARN	Poly(A)-Specific Ribonuclease
5087.	PAX9	Paired Box 9
5088.	PDE6C	Phosphodiesterase 6C
5089.	RORC	RAR Related Orphan Receptor C
5090.	RUNX3	RUNX Family Transcription Factor 3
5091.	SALL4	Spalt Like Transcription Factor 4
5092.	SEC61A1	SEC61 Translocon Subunit Alpha 1
5093.	SLC20A1	Solute Carrier Family 20 Member 1
5094.	SMYD2	SET And MYND Domain Containing 2
5095.	TXK	TXK Tyrosine Kinase
5096.	TXNRD1	Thioredoxin Reductase 1
5097.	BACH2	BTB Domain And CNC Homolog 2
5098.	CAPN5	Calpain 5
5099.	CCL20	C-C Motif Chemokine Ligand 20
5100.	DUT	Deoxyuridine Triphosphatase
5101.	ERLIN1	ER Lipid Raft Associated 1
5102.	GGH	Gamma-Glutamyl Hydrolase

5103.	GOSR2	Golgi SNAP Receptor Complex Member 2
5104.	GRK1	G Protein-Coupled Receptor Kinase 1
5105.	LCT	Lactase
5106.	MTHFS	Methenyltetrahydrofolate Synthetase
5107.	PCDH15	Protocadherin Related 15
5108.	PLA2G10	Phospholipase A2 Group X
5109.	PMEL	Premelanosome Protein
5110.	PTPRK	Protein Tyrosine Phosphatase Receptor Type K
5111.	RHAG	Rh Associated Glycoprotein
5112.	ROM1	Retinal Outer Segment Membrane Protein 1
5113.	RPL35A	Ribosomal Protein L35a
5114.	SETD1A	SET Domain Containing 1A, Histone Lysine Methyltransferase
5115.	SLC44A1	Solute Carrier Family 44 Member 1
5116.	SLC52A3	Solute Carrier Family 52 Member 3
5117.	SOX17	SRY-Box Transcription Factor 17
5118.	TGM3	Transglutaminase 3
5119.	TNFRSF10D	TNF Receptor Superfamily Member 10d
5120.	TRAF1	TNF Receptor Associated Factor 1
5121.	TRPC4	Transient Receptor Potential Cation Channel Subfamily C Member 4
5122.	UFD1	Ubiquitin Recognition Factor In ER Associated Degradation 1
5123.	WNT7B	Wnt Family Member 7B
5124.	WWP2	WW Domain Containing E3 Ubiquitin Protein Ligase 2
5125.	ABI1	Abl Interactor 1
5126.	ALDH9A1	Aldehyde Dehydrogenase 9 Family Member A1
5127.	APOA4	Apolipoprotein A4
5128.	ASAH2	N-Acylsphingosine Amidohydrolase 2
5129.	BDKRB1	Bradykinin Receptor B1
5130.	BMP3	Bone Morphogenetic Protein 3
5131.	CARS2	Cysteinyl-TRNA Synthetase 2, Mitochondrial
5132.	CDS1	CDP-Diacylglycerol Synthase 1
5133.	CNDP2	Carnosine Dipeptidase 2
5134.	CRTC1	CREB Regulated Transcription Coactivator 1
5135.	DEK	DEK Proto-Oncogene
5136.	GMDS	GDP-Mannose 4,6-Dehydratase
5137.	GRIN3B	Glutamate Ionotropic Receptor NMDA Type Subunit 3B
5138.	HPX	Hemopexin
5139.	ICAM3	Intercellular Adhesion Molecule 3
5140.	IL18BP	Interleukin 18 Binding Protein
5141.	IL1RL1	Interleukin 1 Receptor Like 1
5142.	KCNH6	Potassium Voltage-Gated Channel Subfamily H Member 6
5143.	KERA	Keratocan

5144.	MLYCD	Malonyl-CoA Decarboxylase
5145.	PAPSS1	3'-Phosphoadenosine 5'-Phosphosulfate Synthase 1
5146.	PGAP1	Post-GPI Attachment To Proteins Inositol Deacylase 1
5147.	POU2F1	POU Class 2 Homeobox 1
5148.	PPP6C	Protein Phosphatase 6 Catalytic Subunit
5149.	RPS13	Ribosomal Protein S13
5150.	RPS14	Ribosomal Protein S14
5151.	SCAP	SREBF Chaperone
5152.	SIX6	SIX Homeobox 6
5153.	SLC22A7	Solute Carrier Family 22 Member 7
5154.	SLC34A3	Solute Carrier Family 34 Member 3
5155.	SLC45A2	Solute Carrier Family 45 Member 2
5156.	SLC4A2	Solute Carrier Family 4 Member 2
5157.	SRR	Serine Racemase
5158.	TINF2	TERF1 Interacting Nuclear Factor 2
5159.	TMC8	Transmembrane Channel Like 8
5160.	UBASH3B	Ubiquitin Associated And SH3 Domain Containing B
5161.	UGT1A10	UDP Glucuronosyltransferase Family 1 Member A10
5162.	XPNPEP3	X-Prolyl Aminopeptidase 3
5163.	ANKRD26	Ankyrin Repeat Domain Containing 26
5164.	ATG3	Autophagy Related 3
5165.	BAZ1B	Bromodomain Adjacent To Zinc Finger Domain 1B
5166.	CAPN10	Calpain 10
5167.	CCNT1	Cyclin T1
5168.	CCR9	C-C Motif Chemokine Receptor 9
5169.	CDKAL1	CDK5 Regulatory Subunit Associated Protein 1 Like 1
5170.	CEP55	Centrosomal Protein 55
5171.	EXOC5	Exocyst Complex Component 5
5172.	FOXF1	Forkhead Box F1
5173.	FTSJ1	FtsJ RNA 2'-O-Methyltransferase 1
5174.	ICA1	Islet Cell Autoantigen 1
5175.	ITGAE	Integrin Subunit Alpha E
5176.	MAPRE2	Microtubule Associated Protein RP/EB Family Member 2
5177.	MKKS	MKKS Centrosomal Shuttling Protein
5178.	NR4A3	Nuclear Receptor Subfamily 4 Group A Member 3
5179.	ONECUT1	One Cut Homeobox 1
5180.	PDK2	Pyruvate Dehydrogenase Kinase 2
5181.	RUNX1T1	RUNX1 Partner Transcriptional Co-Repressor 1
5182.	SRP72	Signal Recognition Particle 72
5183.	SRSF6	Serine And Arginine Rich Splicing Factor 6
5184.	ACR	Acrosin

5185.	ALG6	ALG6 Alpha-1,3-Glucosyltransferase
5186.	ATP8B1	ATPase Phospholipid Transporting 8B1
5187.	BAG2	BAG Cochaperone 2
5188.	BFSP2	Beaded Filament Structural Protein 2
5189.	CACNG3	Calcium Voltage-Gated Channel Auxiliary Subunit Gamma 3
5190.	CDH16	Cadherin 16
5191.	CLDN19	Claudin 19
5192.	CRYBA4	Crystallin Beta A4
5193.	CRYBB2	Crystallin Beta B2
5194.	ELMO1	Engulfment And Cell Motility 1
5195.	EN1	Engrailed Homeobox 1
5196.	EXOSC8	Exosome Component 8
5197.	FLAD1	Flavin Adenine Dinucleotide Synthetase 1
5198.	FLVCR1	FLVCR Choline And Heme Transporter 1
5199.	FSCN2	Fascin Actin-Bundling Protein 2, Retinal
5200.	GLIS2	GLIS Family Zinc Finger 2
5201.	GRK4	G Protein-Coupled Receptor Kinase 4
5202.	HLA-DMA	Major Histocompatibility Complex, Class II, DM Alpha
5203.	HLA-DMB	Major Histocompatibility Complex, Class II, DM Beta
5204.	HSD3B7	Hydroxy-Delta-5-Steroid Dehydrogenase, 3 Beta- And Steroid Delta-Isomerase 7
5205.	IL15RA	Interleukin 15 Receptor Subunit Alpha
5206.	IL37	Interleukin 37
5207.	KCNC2	Potassium Voltage-Gated Channel Subfamily C Member 2
5208.	MLF1	Myeloid Leukemia Factor 1
5209.	NPRL2	NPR2 Like, GATOR1 Complex Subunit
5210.	OGN	Osteoglycin
5211.	OPN1SW	Opsin 1, Short Wave Sensitive
5212.	OSBP	Oxysterol Binding Protein
5213.	PARP3	Poly(ADP-Ribose) Polymerase Family Member 3
5214.	PHF6	PHD Finger Protein 6
5215.	PLAG1	PLAG1 Zinc Finger
5216.	PLXNC1	Plexin C1
5217.	PTPRR	Protein Tyrosine Phosphatase Receptor Type R
5218.	RBPMS	RNA Binding Protein, MRNA Processing Factor
5219.	RPL22	Ribosomal Protein L22
5220.	RPLP2	Ribosomal Protein Lateral Stalk Subunit P2
5221.	SEC14L2	SEC14 Like Lipid Binding 2
5222.	SENP1	SUMO Specific Peptidase 1
5223.	SLC6A20	Solute Carrier Family 6 Member 20
5224.	SMPD4	Sphingomyelin Phosphodiesterase 4
5225.	SNTB1	Syntrophin Beta 1

5226.	STN1	STN1 Subunit Of CST Complex
5227.	SUCLG2	Succinate-CoA Ligase GDP-Forming Subunit Beta
5228.	SYNGR1	Synaptogyrin 1
5229.	TET3	Tet Methylcytosine Dioxygenase 3
5230.	TKFC	Triokinase And FMN Cyclase
5231.	TMC1	Transmembrane Channel Like 1
5232.	TNFRSF10C	TNF Receptor Superfamily Member 10c
5233.	VSX2	Visual System Homeobox 2
5234.	WWP1	WW Domain Containing E3 Ubiquitin Protein Ligase 1
5235.	ZDHHC9	Zinc Finger DHHC-Type Palmitoyltransferase 9
5236.	ASXL2	ASXL Transcriptional Regulator 2
5237.	ATOH7	Atonal BHLH Transcription Factor 7
5238.	B9D2	B9 Domain Containing 2
5239.	BLOC1S6	Biogenesis Of Lysosomal Organelles Complex 1 Subunit 6
5240.	BOLA3	BolA Family Member 3
5241.	BRAT1	BRCA1 Associated ATM Activator 1
5242.	CARD8	Caspase Recruitment Domain Family Member 8
5243.	CLTB	Clathrin Light Chain B
5244.	CTF1	Cardiotrophin 1
5245.	DEFB1	Defensin Beta 1
5246.	DOC2A	Double C2 Domain Alpha
5247.	DPH2	Diphthamide Biosynthesis 2
5248.	DTNB	Dystrobrevin Beta
5249.	EFL1	Elongation Factor Like GTPase 1
5250.	ELP3	Elongator Acetyltransferase Complex Subunit 3
5251.	EMC1	ER Membrane Protein Complex Subunit 1
5252.	IL36G	Interleukin 36 Gamma
5253.	KCNIP1	Potassium Voltage-Gated Channel Interacting Protein 1
5254.	KCNT2	Potassium Sodium-Activated Channel Subfamily T Member 2
5255.	KLRB1	Killer Cell Lectin Like Receptor B1
5256.	LALBA	Lactalbumin Alpha
5257.	LGALS9	Galectin 9
5258.	MAB21L1	Mab-21 Like 1
5259.	MAST1	Microtubule Associated Serine/Threonine Kinase 1
5260.	MNDA	Myeloid Cell Nuclear Differentiation Antigen
5261.	NPLOC4	NPL4 Homolog, Ubiquitin Recognition Factor
5262.	OPTC	Opticin
5263.	PARP10	Poly(ADP-Ribose) Polymerase Family Member 10
5264.	PARP6	Poly(ADP-Ribose) Polymerase Family Member 6
5265.	PARP9	Poly(ADP-Ribose) Polymerase Family Member 9
5266.	PDCL	Phosducin Like

5267.	PDE6H	Phosphodiesterase 6H
5268.	PDLIM5	PDZ And LIM Domain 5
5269.	PITPNB	Phosphatidylinositol Transfer Protein Beta
5270.	PKN3	Protein Kinase N3
5271.	PRELP	Proline And Arginine Rich End Leucine Rich Repeat Protein
5272.	RBFOX2	RNA Binding Fox-1 Homolog 2
5273.	SAP30	Sin3A Associated Protein 30
5274.	SIX2	SIX Homeobox 2
5275.	SLC52A1	Solute Carrier Family 52 Member 1
5276.	SLC9A7	Solute Carrier Family 9 Member A7
5277.	SLITRK6	SLIT And NTRK Like Family Member 6
5278.	SPTBN4	Spectrin Beta, Non-Erythrocytic 4
5279.	SRA1	Steroid Receptor RNA Activator 1
5280.	TLR10	Toll Like Receptor 10
5281.	TMOD1	Tropomodulin 1
5282.	TNIP1	TNFAIP3 Interacting Protein 1
5283.	TPRKB	TP53RK Binding Protein
5284.	TRMT1	TRNA Methyltransferase 1
5285.	TSR1	TSR1 Ribosome Maturation Factor
5286.	UBASH3A	Ubiquitin Associated And SH3 Domain Containing A
5287.	UROC1	Urocanate Hydratase 1
5288.	USH2A	Usherin
5289.	VRK2	VRK Serine/Threonine Kinase 2
5290.	ACSL6	Acyl-CoA Synthetase Long Chain Family Member 6
5291.	ACSM1	Acyl-CoA Synthetase Medium Chain Family Member 1
5292.	ARID4B	AT-Rich Interaction Domain 4B
5293.	ARL6IP1	ADP Ribosylation Factor Like GTPase 6 Interacting Protein 1
5294.	BOK	BCL2 Family Apoptosis Regulator BOK
5295.	CDH9	Cadherin 9
5296.	CNMD	Chondromodulin
5297.	DEFA5	Defensin Alpha 5
5298.	DPH5	Diphthamide Biosynthesis 5
5299.	DR1	Down-Regulator Of Transcription 1
5300.	ESAM	Endothelial Cell Adhesion Molecule
5301.	FAT4	FAT Atypical Cadherin 4
5302.	FERMT2	FERM Domain Containing Kindlin 2
5303.	FMN1	Formin 1
5304.	FOLR3	Folate Receptor Gamma
5305.	G6PC2	Glucose-6-Phosphatase Catalytic Subunit 2
5306.	GIMAP5	GTPase, IMAP Family Member 5
5307.	GRIN3A	Glutamate Ionotropic Receptor NMDA Type Subunit 3A

5308.	HLA-DQA2	Major Histocompatibility Complex, Class II, DQ Alpha 2
5309.	HS6ST2	Heparan Sulfate 6-O-Sulfotransferase 2
5310.	IBA57	Iron-Sulfur Cluster Assembly Factor IBA57
5311.	IL18RAP	Interleukin 18 Receptor Accessory Protein
5312.	IL1F10	Interleukin 1 Family Member 10
5313.	IL36A	Interleukin 36 Alpha
5314.	INTS8	Integrator Complex Subunit 8
5315.	IRX5	Iroquois Homeobox 5
5316.	NSMCE3	NSE3 Homolog, SMC5-SMC6 Complex Component
5317.	PARP12	Poly(ADP-Ribose) Polymerase Family Member 12
5318.	PARP14	Poly(ADP-Ribose) Polymerase Family Member 14
5319.	PRELID1	PRELI Domain Containing 1
5320.	PUS10	Pseudouridine Synthase 10
5321.	RASD1	Ras Related Dexamethasone Induced 1
5322.	RDH8	Retinol Dehydrogenase 8
5323.	RIN3	Ras And Rab Interactor 3
5324.	RNF113A	Ring Finger Protein 113A
5325.	RNLS	Renalase, FAD Dependent Amine Oxidase
5326.	SELENOI	Selenoprotein I
5327.	SLC52A2	Solute Carrier Family 52 Member 2
5328.	SPON2	Spondin 2
5329.	THEMIS	Thymocyte Selection Associated
5330.	TMEM231	Transmembrane Protein 231
5331.	TMOD3	Tropomodulin 3
5332.	TRIM39	Tripartite Motif Containing 39
5333.	UBXN6	UBX Domain Protein 6
5334.	UGT1A8	UDP Glucuronosyltransferase Family 1 Member A8
5335.	ULK4	Unc-51 Like Kinase 4
5336.	ZC3HAV1	Zinc Finger CCCH-Type Containing, Antiviral 1
5337.	ZCCHC8	Zinc Finger CCHC-Type Containing 8
5338.	ACSM3	Acyl-CoA Synthetase Medium Chain Family Member 3
5339.	APLN	Apelin
5340.	BMP2K	BMP2 Inducible Kinase
5341.	CABP4	Calcium Binding Protein 4
5342.	CD248	CD248 Molecule
5343.	COPS7A	COP9 Signalosome Subunit 7A
5344.	EMB	Embigin
5345.	GAS7	Growth Arrest Specific 7
5346.	GZF1	GDNF Inducible Zinc Finger Protein 1
5347.	HOXA9	Homeobox A9
5348.	IL26	Interleukin 26

5349.	IMMP1L	Inner Mitochondrial Membrane Peptidase Subunit 1
5350.	ITIH2	Inter-Alpha-Trypsin Inhibitor Heavy Chain 2
5351.	KIF6	Kinesin Family Member 6
5352.	LACC1	Laccase Domain Containing 1
5353.	LAGE3	L Antigen Family Member 3
5354.	LARP1	La Ribonucleoprotein 1, Translational Regulator
5355.	MEX3C	Mex-3 RNA Binding Family Member C
5356.	MTMR10	Myotubularin Related Protein 10
5357.	NPRL3	NPR3 Like, GATOR1 Complex Subunit
5358.	OSBP2	Oxysterol Binding Protein 2
5359.	PPFIA2	PTPRF Interacting Protein Alpha 2
5360.	PRSS16	Serine Protease 16
5361.	RGS1	Regulator Of G Protein Signaling 1
5362.	RPS21	Ribosomal Protein S21
5363.	SAMD9L	Sterile Alpha Motif Domain Containing 9 Like
5364.	SAMM50	SAMM50 Sorting And Assembly Machinery Component
5365.	SGSM3	Small G Protein Signaling Modulator 3
5366.	SLC25A31	Solute Carrier Family 25 Member 31
5367.	ST18	ST18 C2H2C-Type Zinc Finger Transcription Factor
5368.	SUCNR1	Succinate Receptor 1
5369.	USP42	Ubiquitin Specific Peptidase 42
5370.	ZNF687	Zinc Finger Protein 687
5371.	ADAD1	Adenosine Deaminase Domain Containing 1
5372.	ATP13A4	ATPase 13A4
5373.	BRWD3	Bromodomain And WD Repeat Domain Containing 3
5374.	CPPED1	Calcineurin Like Phosphoesterase Domain Containing 1
5375.	DCSTAMP	Dendrocyte Expressed Seven Transmembrane Protein
5376.	DEFB4A	Defensin Beta 4A
5377.	DPM2	Dolichyl-Phosphate Mannosyltransferase Subunit 2, Regulatory
5378.	FCHSD1	FCH And Double SH3 Domains 1
5379.	GIMAP2	GTPase, IMAP Family Member 2
5380.	GIMAP4	GTPase, IMAP Family Member 4
5381.	GPR158	G Protein-Coupled Receptor 158
5382.	ICAM4	Intercellular Adhesion Molecule 4 (Landsteiner-Wiener Blood Group)
5383.	IFI6	Interferon Alpha Inducible Protein 6
5384.	KIF12	Kinesin Family Member 12
5385.	MKX	Mohawk Homeobox
5386.	NDUFB4	NADH:Ubiquinone Oxidoreductase Subunit B4
5387.	NT5C1B	5'-Nucleotidase, Cytosolic IB
5388.	NUTF2	Nuclear Transport Factor 2
5389.	PARP15	Poly(ADP-Ribose) Polymerase Family Member 15

5390.	PARP16	Poly(ADP-Ribose) Polymerase Family Member 16
5391.	PFN4	Profilin Family Member 4
5392.	PIK3AP1	Phosphoinositide-3-Kinase Adaptor Protein 1
5393.	PNPLA4	Patatin Like Phospholipase Domain Containing 4
5394.	POM121	POM121 Transmembrane Nucleoporin
5395.	PPP1R21	Protein Phosphatase 1 Regulatory Subunit 21
5396.	RBM20	RNA Binding Motif Protein 20
5397.	SAMD9	Sterile Alpha Motif Domain Containing 9
5398.	SLC22A14	Solute Carrier Family 22 Member 14
5399.	SPIRE1	Spire Type Actin Nucleation Factor 1
5400.	TMEM11	Transmembrane Protein 11
5401.	TTF1	Transcription Termination Factor 1
5402.	ZBTB38	Zinc Finger And BTB Domain Containing 38
5403.	ZFR	Zinc Finger RNA Binding Protein
5404.	AGBL3	AGBL Carboxypeptidase 3
5405.	ALKBH8	AlkB Homolog 8, TRNA Methyltransferase
5406.	ANKS3	Ankyrin Repeat And Sterile Alpha Motif Domain Containing 3
5407.	AQP11	Aquaporin 11
5408.	ARHGAP42	Rho GTPase Activating Protein 42
5409.	CLUL1	Clusterin Like 1
5410.	DCD	Dermcidin
5411.	EPYC	Epiphycan
5412.	FNDC3B	Fibronectin Type III Domain Containing 3B
5413.	HEBP1	Heme Binding Protein 1
5414.	HYAL3	Hyaluronidase 3
5415.	IL36B	Interleukin 36 Beta
5416.	LETM2	Leucine Zipper And EF-Hand Containing Transmembrane Protein 2
5417.	LNPK	Lunapark, ER Junction Formation Factor
5418.	NDUFAF7	NADH:Ubiquinone Oxidoreductase Complex Assembly Factor 7
5419.	PCARE	Photoreceptor Cilium Actin Regulator
5420.	PCMTD1	Protein-L-Isoaspartate (D-Aspartate) O-Methyltransferase Domain Containing 1
5421.	PHF10	PHD Finger Protein 10
5422.	PRICKLE4	Prickle Planar Cell Polarity Protein 4
5423.	PSTPIP2	Proline-Serine-Threonine Phosphatase Interacting Protein 2
5424.	PURB	Purine Rich Element Binding Protein B
5425.	RAX2	Retina And Anterior Neural Fold Homeobox 2
5426.	SLC46A3	Solute Carrier Family 46 Member 3
5427.	STYX	Serine/Threonine/Tyrosine Interacting Protein
5428.	TECPR2	Tectonin Beta-Propeller Repeat Containing 2
5429.	TMC2	Transmembrane Channel Like 2
5430.	TRIM69	Tripartite Motif Containing 69

5431.	XCL1	X-C Motif Chemokine Ligand 1
5432.	CSN2	Casein Beta
5433.	DNASE1L2	Deoxyribonuclease 1 Like 2
5434.	ELP5	Elongator Acetyltransferase Complex Subunit 5
5435.	FAM124A	Family With Sequence Similarity 124 Member A
5436.	NRBP1	Nuclear Receptor Binding Protein 1
5437.	SHISA6	Shisa Family Member 6
5438.	TIPARP	TCDD Inducible Poly(ADP-Ribose) Polymerase
5439.	TTLL12	Tubulin Tyrosine Ligase Like 12
5440.	UMODL1	Uromodulin Like 1
5441.	ARSI	Arylsulfatase Family Member I
5442.	AUP1	AUP1 Lipid Droplet Regulating VLDL Assembly Factor
5443.	CAMSAP2	Calmodulin Regulated Spectrin Associated Protein Family Member 2
5444.	CFAP418	Cilia And Flagella Associated Protein 418
5445.	CHCHD7	Coiled-Coil-Helix-Coiled-Coil-Helix Domain Containing 7
5446.	COMMD5	COMM Domain Containing 5
5447.	ELAPOR2	Endosome-Lysosome Associated Apoptosis And Autophagy Regulator Family Member 2
5448.	ERI3	ERI1 Exoribonuclease Family Member 3
5449.	FABP12	Fatty Acid Binding Protein 12
5450.	FAM53B	Family With Sequence Similarity 53 Member B
5451.	FKBPL	FKBP Prolyl Isomerase Like
5452.	IZUMO4	IZUMO Family Member 4
5453.	MAP3K15	Mitogen-Activated Protein Kinase Kinase Kinase 15
5454.	MDFI	MyoD Family Inhibitor
5455.	MREG	Melanoregulin
5456.	NANP	N-Acetylneuraminic Acid Phosphatase
5457.	NOP9	NOP9 Nucleolar Protein
5458.	NPBWR1	Neuropeptides B And W Receptor 1
5459.	PHF14	PHD Finger Protein 14
5460.	PPP4R3B	Protein Phosphatase 4 Regulatory Subunit 3B
5461.	RASAL3	RAS Protein Activator Like 3
5462.	REG3G	Regenerating Family Member 3 Gamma
5463.	SCHIP1	Schwannomin Interacting Protein 1
5464.	SLC25A2	Solute Carrier Family 25 Member 2
5465.	SLC46A2	Solute Carrier Family 46 Member 2
5466.	ZBTB14	Zinc Finger And BTB Domain Containing 14
5467.	ANKRD55	Ankyrin Repeat Domain 55
5468.	CCDC102B	Coiled-Coil Domain Containing 102B
5469.	CCDC136	Coiled-Coil Domain Containing 136
5470.	CCDC180	Coiled-Coil Domain Containing 180
5471.	DEDD	Death Effector Domain Containing

5472.	ELP6	Elongator Acetyltransferase Complex Subunit 6
5473.	FAM168B	Family With Sequence Similarity 168 Member B
5474.	H3C6	H3 Clustered Histone 6
5475.	HTN3	Histatin 3
5476.	KIR2DS4	Killer Cell Immunoglobulin Like Receptor, Two Ig Domains And Short Cytoplasmic Tail 4
5477.	KLHDC7A	Kelch Domain Containing 7A
5478.	MED8	Mediator Complex Subunit 8
5479.	MTMR11	Myotubularin Related Protein 11
5480.	PIP5KL1	Phosphatidylinositol-4-Phosphate 5-Kinase Like 1
5481.	SCNM1	Sodium Channel Modifier 1
5482.	SPINK4	Serine Peptidase Inhibitor Kazal Type 4
5483.	URM1	Ubiquitin Related Modifier 1
5484.	YPEL1	Yippee Like 1
5485.	ZMAT4	Zinc Finger Matrin-Type 4
5486.	ACTRT1	Actin Related Protein T1
5487.	ANKAR	Ankyrin And Armadillo Repeat Containing
5488.	CCDC148	Coiled-Coil Domain Containing 148
5489.	EEIG1	Estrogen-Induced Osteoclastogenesis Regulator 1
5490.	GPRIN2	G Protein Regulated Inducer Of Neurite Outgrowth 2
5491.	LDAH	Lipid Droplet Associated Hydrolase
5492.	PEDS1	Plasmanylethanolamine Desaturase 1
5493.	PFN3	Profilin 3
5494.	SPAG7	Sperm Associated Antigen 7
5495.	TIGD1	Tigger Transposable Element Derived 1
5496.	YDJC	YdjC Chitooligosaccharide Deacetylase Homolog
5497.	ZNF621	Zinc Finger Protein 621
5498.	CD300LD	CD300 Molecule Like Family Member D
5499.	DCDC1	Doublecortin Domain Containing 1
5500.	DEFB125	Defensin Beta 125
5501.	KTI12	KTI12 Chromatin Associated Homolog
5502.	OCM	Oncomodulin
5503.	PIERCE1	Piercer Of Microtubule Wall 1
5504.	RFX8	Regulatory Factor X8
5505.	SHFL	Shiftless Antiviral Inhibitor Of Ribosomal Frameshifting
5506.	C1QTNF9B	C1q And TNF Related 9B
5507.	CST11	Cystatin 11
5508.	GML	Glycosylphosphatidylinositol Anchored Molecule Like
5509.	GOLGA8B	Golgin A8 Family Member B
5510.	LCE3C	Late Cornified Envelope 3C
5511.	OR10S1	Olfactory Receptor Family 10 Subfamily S Member 1
5512.	OR52B2	Olfactory Receptor Family 52 Subfamily B Member 2

5513.	PPP1R3E	Protein Phosphatase 1 Regulatory Subunit 3E
5514.	SLC35E2B	Solute Carrier Family 35 Member E2B
5515.	ZNF444	Zinc Finger Protein 444
5516.	ZNF681	Zinc Finger Protein 681
5517.	BLID	BH3-Like Motif Containing, Cell Death Inducer
5518.	BNIP5	BCL2 Interacting Protein 5
5519.	C12orf75	Chromosome 12 Open Reading Frame 75
5520.	DGAT2L6	Diacylglycerol O-Acyltransferase 2 Like 6
5521.	GK3	Glycerol Kinase 3
5522.	OR13H1	Olfactory Receptor Family 13 Subfamily H Member 1
5523.	RIPPLY1	Ripply Transcriptional Repressor 1
5524.	SMIM11	Small Integral Membrane Protein 11
5525.	TOMM20L	Translocase Of Outer Mitochondrial Membrane 20 Like
5526.	DEPDC4	DEP Domain Containing 4
5527.	OR8H2	Olfactory Receptor Family 8 Subfamily H Member 2
5528.	CCDC175	Coiled-Coil Domain Containing 175
5529.	HOATZ	HOATZ Cilia And Flagella Associated Protein
5530.	LCE3B	Late Cornified Envelope 3B
5531.	OR6P1	Olfactory Receptor Family 6 Subfamily P Member 1
5532.	VCX2	Variable Charge X-Linked 2
5533.	VCX3A	Variable Charge X-Linked 3A
5534.	ZCCHC18	Zinc Finger CCHC-Type Containing 18
5535.	ZNF789	Zinc Finger Protein 789
5536.	MGAM2	Maltase-Glucoamylase 2 (Putative)
5537.	NPIPB3	Nuclear Pore Complex Interacting Protein Family Member B3
5538.	OR8H3	Olfactory Receptor Family 8 Subfamily H Member 3
5539.	ANKRD66	Ankyrin Repeat Domain 66
5540.	APELA	Apelin Receptor Early Endogenous Ligand
5541.	MT1HL1	Metallothionein 1H Like 1
5542.	NT5C1B-RDH14	NT5C1B-RDH14 Readthrough
5543.	SPATA31A3	SPATA31 Subfamily A Member 3
5544.	TRA	T Cell Receptor Alpha Locus
5545.	TRIM39-RPP21	TRIM39-RPP21 Readthrough
5546.	IGKV1D-33	Immunoglobulin Kappa Variable 1D-33
5547.	ETDC	Embryonic Testis Differentiation Homolog C
5548.	RIPPLY2-CYB5R4	RIPPLY2-CYB5R4 Readthrough
5549.	LOC102723553	Small Integral Membrane Protein 11B
5550.	FSHR	Follicle Stimulating Hormone Receptor
5551.	PNLIP	Pancreatic Lipase
5552.	WAS	WASP Actin Nucleation Promoting Factor
5553.	ALDOB	Aldolase, Fructose-Bisphosphate B

5554.	FMO3	Flavin Containing Dimethylaniline Monoxygenase 3
5555.	ADORA3	Adenosine A3 Receptor
5556.	GCLC	Glutamate-Cysteine Ligase Catalytic Subunit
5557.	B3GAT1	Beta-1,3-Glucuronyltransferase 1
5558.	GCLM	Glutamate-Cysteine Ligase Modifier Subunit
5559.	GSTA1	Glutathione S-Transferase Alpha 1
5560.	GUK1	Guanylate Kinase 1
5561.	RAI1	Retinoic Acid Induced 1
5562.	ZDHHC24	Zinc Finger DHHC-Type Containing 24
5563.	IKBKB	Inhibitor Of Nuclear Factor Kappa B Kinase Subunit Beta
5564.	NFKB2	Nuclear Factor Kappa B Subunit 2
5565.	PAK1	P21 (RAC1) Activated Kinase 1
5566.	ADK	Adenosine Kinase
5567.	CHUK	Component Of Inhibitor Of Nuclear Factor Kappa B Kinase Complex
5568.	DYRK1A	Dual Specificity Tyrosine Phosphorylation Regulated Kinase 1A
5569.	ACVR2B	Activin A Receptor Type 2B
5570.	AXIN2	Axin 2
5571.	BUB1B	BUB1 Mitotic Checkpoint Serine/Threonine Kinase B
5572.	CSNK1A1	Casein Kinase 1 Alpha 1
5573.	ECE1	Endothelin Converting Enzyme 1
5574.	RRM1	Ribonucleotide Reductase Catalytic Subunit M1
5575.	SLC2A2	Solute Carrier Family 2 Member 2
5576.	YWHAE	Tyrosine 3-Monooxygenase/Tryptophan 5-Monooxygenase Activation Protein Epsilon
5577.	CSNK2B	Casein Kinase 2 Beta
5578.	PRLR	Prolactin Receptor
5579.	PTGER2	Prostaglandin E Receptor 2
5580.	TUBG1	Tubulin Gamma 1
5581.	ABCA3	ATP Binding Cassette Subfamily A Member 3
5582.	ACAT1	Acetyl-CoA Acetyltransferase 1
5583.	ALDH1A1	Aldehyde Dehydrogenase 1 Family Member A1
5584.	AQP5	Aquaporin 5
5585.	BIRC2	Baculoviral IAP Repeat Containing 2
5586.	CDC25A	Cell Division Cycle 25A
5587.	CDC25C	Cell Division Cycle 25C
5588.	CYLD	CYLD Lysine 63 Deubiquitinase
5589.	DMPK	DM1 Protein Kinase
5590.	FOLH1	Folate Hydrolase 1
5591.	GRK2	G Protein-Coupled Receptor Kinase 2
5592.	HSF1	Heat Shock Transcription Factor 1
5593.	IGF2R	Insulin Like Growth Factor 2 Receptor
5594.	KRT1	Keratin 1

5595.	MMP7	Matrix Metallopeptidase 7
5596.	PAK3	P21 (RAC1) Activated Kinase 3
5597.	PI4KA	Phosphatidylinositol 4-Kinase Alpha
5598.	PIN1	Peptidylprolyl Cis/Trans Isomerase, NIMA-Interacting 1
5599.	PLK4	Polo Like Kinase 4
5600.	PRKAB1	Protein Kinase AMP-Activated Non-Catalytic Subunit Beta 1
5601.	RRM2	Ribonucleotide Reductase Regulatory Subunit M2
5602.	ST3GAL5	ST3 Beta-Galactoside Alpha-2,3-Sialyltransferase 5
5603.	TCF4	Transcription Factor 4
5604.	VCL	Vinculin
5605.	YY1	YY1 Transcription Factor
5606.	ACTG1	Actin Gamma 1
5607.	ACTN2	Actinin Alpha 2
5608.	ANXA1	Annexin A1
5609.	AURKC	Aurora Kinase C
5610.	CAMKK2	Calcium/Calmodulin Dependent Protein Kinase Kinase 2
5611.	EPHA3	EPH Receptor A3
5612.	FBLN5	Fibulin 5
5613.	FEN1	Flap Structure-Specific Endonuclease 1
5614.	FTO	FTO Alpha-Ketoglutarate Dependent Dioxygenase
5615.	GFPT1	Glutamine--Fructose-6-Phosphate Transaminase 1
5616.	GLDC	Glycine Decarboxylase
5617.	HSD17B10	Hydroxysteroid 17-Beta Dehydrogenase 10
5618.	HSP90B1	Heat Shock Protein 90 Beta Family Member 1
5619.	KCNJ6	Potassium Inwardly Rectifying Channel Subfamily J Member 6
5620.	KDM5C	Lysine Demethylase 5C
5621.	LIG1	DNA Ligase 1
5622.	MAP2K7	Mitogen-Activated Protein Kinase Kinase 7
5623.	MAPK12	Mitogen-Activated Protein Kinase 12
5624.	MAPK13	Mitogen-Activated Protein Kinase 13
5625.	MMP19	Matrix Metallopeptidase 19
5626.	OGT	O-Linked N-Acetylglucosamine (GlcNAc) Transferase
5627.	PEBP1	Phosphatidylethanolamine Binding Protein 1
5628.	PTPN12	Protein Tyrosine Phosphatase Non-Receptor Type 12
5629.	SLC5A1	Solute Carrier Family 5 Member 1
5630.	SLCO1B1	Solute Carrier Organic Anion Transporter Family Member 1B1
5631.	CASP4	Caspase 4
5632.	CHRNA5	Cholinergic Receptor Nicotinic Alpha 5 Subunit
5633.	CLPP	Caseinolytic Mitochondrial Matrix Peptidase Proteolytic Subunit
5634.	CSNK2A2	Casein Kinase 2 Alpha 2
5635.	DLAT	Dihydrolipoamide S-Acetyltransferase

5636.	ECHS1	Enoyl-CoA Hydratase, Short Chain 1
5637.	EEF2K	Eukaryotic Elongation Factor 2 Kinase
5638.	FLRT3	Fibronectin Leucine Rich Transmembrane Protein 3
5639.	FPR2	Formyl Peptide Receptor 2
5640.	GALT	Galactose-1-Phosphate Uridylyltransferase
5641.	GRK6	G Protein-Coupled Receptor Kinase 6
5642.	HCN2	Hyperpolarization Activated Cyclic Nucleotide Gated Potassium And Sodium Channel 2
5643.	HMOX2	Heme Oxygenase 2
5644.	HNRNPK	Heterogeneous Nuclear Ribonucleoprotein K
5645.	LINGO1	Leucine Rich Repeat And Ig Domain Containing 1
5646.	LPAR1	Lysophosphatidic Acid Receptor 1
5647.	MAPK11	Mitogen-Activated Protein Kinase 11
5648.	MEF2A	Myocyte Enhancer Factor 2A
5649.	MSR1	Macrophage Scavenger Receptor 1
5650.	NAA10	N-Alpha-Acetyltransferase 10, NatA Catalytic Subunit
5651.	NR1D1	Nuclear Receptor Subfamily 1 Group D Member 1
5652.	PADI4	Peptidyl Arginine Deiminase 4
5653.	PCK2	Phosphoenolpyruvate Carboxykinase 2, Mitochondrial
5654.	PIK3R4	Phosphoinositide-3-Kinase Regulatory Subunit 4
5655.	PPP2R5D	Protein Phosphatase 2 Regulatory Subunit B'Delta
5656.	PYCR2	Pyrroline-5-Carboxylate Reductase 2
5657.	RELB	RELB Proto-Oncogene, NF-KB Subunit
5658.	SIAH1	Siah E3 Ubiquitin Protein Ligase 1
5659.	SIN3A	SIN3 Transcription Regulator Family Member A
5660.	SORT1	Sortilin 1
5661.	ST3GAL3	ST3 Beta-Galactoside Alpha-2,3-Sialyltransferase 3
5662.	TALDO1	Transaldolase 1
5663.	TNFRSF13C	TNF Receptor Superfamily Member 13C
5664.	TRAF2	TNF Receptor Associated Factor 2
5665.	TXNRD2	Thioredoxin Reductase 2
5666.	XPA	XPA, DNA Damage Recognition And Repair Factor
5667.	AIMP1	Aminoacyl TRNA Synthetase Complex Interacting Multifunctional Protein 1
5668.	BAK1	BCL2 Antagonist/Killer 1
5669.	CACNG2	Calcium Voltage-Gated Channel Auxiliary Subunit Gamma 2
5670.	CAMK4	Calcium/Calmodulin Dependent Protein Kinase IV
5671.	CCKBR	Cholecystokinin B Receptor
5672.	CEBPB	CCAAT Enhancer Binding Protein Beta
5673.	CELF2	CUGBP Elav-Like Family Member 2
5674.	CFD	Complement Factor D
5675.	CHKB	Choline Kinase Beta
5676.	CHRNB1	Cholinergic Receptor Nicotinic Beta 1 Subunit

5677.	CHRNB4	Cholinergic Receptor Nicotinic Beta 4 Subunit
5678.	COASY	Coenzyme A Synthase
5679.	COL6A3	Collagen Type VI Alpha 3 Chain
5680.	DCLRE1C	DNA Cross-Link Repair 1C
5681.	DHCR24	24-Dehydrocholesterol Reductase
5682.	EIF2AK1	Eukaryotic Translation Initiation Factor 2 Alpha Kinase 1
5683.	ENPP2	Ectonucleotide Pyrophosphatase/Phosphodiesterase 2
5684.	FXR1	FMR1 Autosomal Homolog 1
5685.	GJA8	Gap Junction Protein Alpha 8
5686.	GLRA2	Glycine Receptor Alpha 2
5687.	GRIK5	Glutamate Ionotropic Receptor Kainate Type Subunit 5
5688.	HDAC11	Histone Deacetylase 11
5689.	MAD2L1	Mitotic Arrest Deficient 2 Like 1
5690.	MADD	MAP Kinase Activating Death Domain
5691.	MAPK8IP1	Mitogen-Activated Protein Kinase 8 Interacting Protein 1
5692.	MARK4	Microtubule Affinity Regulating Kinase 4
5693.	ME2	Malic Enzyme 2
5694.	MYH2	Myosin Heavy Chain 2
5695.	MYOCD	Myocardin
5696.	NAE1	NEDD8 Activating Enzyme E1 Subunit 1
5697.	NNMT	Nicotinamide N-Methyltransferase
5698.	NONO	Non-POU Domain Containing Octamer Binding
5699.	PDE10A	Phosphodiesterase 10A
5700.	PEX1	Peroxisomal Biogenesis Factor 1
5701.	PSMC3	Proteasome 26S Subunit, ATPase 3
5702.	RBBP4	RB Binding Protein 4, Chromatin Remodeling Factor
5703.	RPA2	Replication Protein A2
5704.	RUVBL1	RuvB Like AAA ATPase 1
5705.	SLC1A5	Solute Carrier Family 1 Member 5
5706.	SLC7A11	Solute Carrier Family 7 Member 11
5707.	SNRPN	Small Nuclear Ribonucleoprotein Polypeptide N
5708.	SOAT1	Sterol O-Acyltransferase 1
5709.	TEAD1	TEA Domain Transcription Factor 1
5710.	TIAM1	TIAM Rac1 Associated GEF 1
5711.	TUBA8	Tubulin Alpha 8
5712.	TUBB1	Tubulin Beta 1 Class VI
5713.	TUBB2B	Tubulin Beta 2B Class IIb
5714.	UBE2D1	Ubiquitin Conjugating Enzyme E2 D1
5715.	UGCG	UDP-Glucose Ceramide Glucosyltransferase
5716.	VAMP2	Vesicle Associated Membrane Protein 2
5717.	VDAC2	Voltage Dependent Anion Channel 2

5718.	ADAM22	ADAM Metallopeptidase Domain 22
5719.	AMFR	Autocrine Motility Factor Receptor
5720.	ANXA6	Annexin A6
5721.	BCKDK	Branched Chain Keto Acid Dehydrogenase Kinase
5722.	BCL11B	BCL11 Transcription Factor B
5723.	BLVRA	Biliverdin Reductase A
5724.	CCT5	Chaperonin Containing TCP1 Subunit 5
5725.	CDK13	Cyclin Dependent Kinase 13
5726.	CHKA	Choline Kinase Alpha
5727.	CNTFR	Ciliary Neurotrophic Factor Receptor
5728.	CRK	CRK Proto-Oncogene, Adaptor Protein
5729.	CYC1	Cytochrome C1
5730.	DTNA	Dystrobrevin Alpha
5731.	EIF4A2	Eukaryotic Translation Initiation Factor 4A2
5732.	ENTPD3	Ectonucleoside Triphosphate Diphosphohydrolase 3
5733.	ESD	Esterase D
5734.	FABP1	Fatty Acid Binding Protein 1
5735.	GABRA6	Gamma-Aminobutyric Acid Type A Receptor Subunit Alpha6
5736.	GADD45A	Growth Arrest And DNA Damage Inducible Alpha
5737.	GALE	UDP-Galactose-4-Epimerase
5738.	GALR3	Galanin Receptor 3
5739.	GDI1	GDP Dissociation Inhibitor 1
5740.	IQGAP1	IQ Motif Containing GTPase Activating Protein 1
5741.	LTC4S	Leukotriene C4 Synthase
5742.	MYH3	Myosin Heavy Chain 3
5743.	MYT1L	Myelin Transcription Factor 1 Like
5744.	NDUFA13	NADH:Ubiquinone Oxidoreductase Subunit A13
5745.	NDUFB9	NADH:Ubiquinone Oxidoreductase Subunit B9
5746.	NUP62	Nucleoporin 62
5747.	PDK3	Pyruvate Dehydrogenase Kinase 3
5748.	PPID	Peptidylprolyl Isomerase D
5749.	PPP3CB	Protein Phosphatase 3 Catalytic Subunit Beta
5750.	PSMA4	Proteasome 20S Subunit Alpha 4
5751.	PSMB5	Proteasome 20S Subunit Beta 5
5752.	PSMD7	Proteasome 26S Subunit, Non-ATPase 7
5753.	RAD17	RAD17 Checkpoint Clamp Loader Component
5754.	ROBO3	Roundabout Guidance Receptor 3
5755.	ROBO4	Roundabout Guidance Receptor 4
5756.	SCN7A	Sodium Voltage-Gated Channel Alpha Subunit 7
5757.	SERPINB5	Serpin Family B Member 5
5758.	SFTPB	Surfactant Protein B

5759.	SGCG	Sarcoglycan Gamma
5760.	SLC25A5	Solute Carrier Family 25 Member 5
5761.	SLC33A1	Solute Carrier Family 33 Member 1
5762.	SLC39A8	Solute Carrier Family 39 Member 8
5763.	SNAI2	Snail Family Transcriptional Repressor 2
5764.	SPHK2	Sphingosine Kinase 2
5765.	TUBB6	Tubulin Beta 6 Class V
5766.	UQCRC1	Ubiquinol-Cytochrome C Reductase Core Protein 1
5767.	AMPD3	Adenosine Monophosphate Deaminase 3
5768.	APBA2	Amyloid Beta Precursor Protein Binding Family A Member 2
5769.	ASPA	Aspartoacylase
5770.	BACE2	Beta-Secretase 2
5771.	BAIAP2	BAR/IMD Domain Containing Adaptor Protein 2
5772.	BLMH	Bleomycin Hydrolase
5773.	BPTF	Bromodomain PHD Finger Transcription Factor
5774.	CCNC	Cyclin C
5775.	CIB1	Calcium And Integrin Binding 1
5776.	CNOT1	CCR4-NOT Transcription Complex Subunit 1
5777.	COPS5	COP9 Signalosome Subunit 5
5778.	CRYZ	Crystallin Zeta
5779.	CSNK1G1	Casein Kinase 1 Gamma 1
5780.	CUL4A	Cullin 4A
5781.	CYFIP2	Cytoplasmic FMR1 Interacting Protein 2
5782.	CYP46A1	Cytochrome P450 Family 46 Subfamily A Member 1
5783.	DFFB	DNA Fragmentation Factor Subunit Beta
5784.	DTNBP1	Dystrobrevin Binding Protein 1
5785.	EXTL3	Exostosin Like Glycosyltransferase 3
5786.	FADS1	Fatty Acid Desaturase 1
5787.	FBP2	Fructose-Bisphosphatase 2
5788.	FLOT1	Flotillin 1
5789.	GABARAP	GABA Type A Receptor-Associated Protein
5790.	GABARAPL2	GABA Type A Receptor Associated Protein Like 2
5791.	GALR1	Galanin Receptor 1
5792.	GNB4	G Protein Subunit Beta 4
5793.	GPR37	G Protein-Coupled Receptor 37
5794.	HAND2	Heart And Neural Crest Derivatives Expressed 2
5795.	HDAC10	Histone Deacetylase 10
5796.	HNRNPDL	Heterogeneous Nuclear Ribonucleoprotein D Like
5797.	HOMER1	Homer Scaffold Protein 1
5798.	IRF6	Interferon Regulatory Factor 6
5799.	KANK1	KN Motif And Ankyrin Repeat Domains 1

5800.	KCNN3	Potassium Calcium-Activated Channel Subfamily N Member 3
5801.	KDSR	3-Ketodihydrosphingosine Reductase
5802.	KHDRBS1	KH RNA Binding Domain Containing, Signal Transduction Associated 1
5803.	KIF5C	Kinesin Family Member 5C
5804.	MFGE8	Milk Fat Globule EGF And Factor V/VIII Domain Containing
5805.	MGLL	Monoglyceride Lipase
5806.	MIB1	MIB E3 Ubiquitin Protein Ligase 1
5807.	MYF6	Myogenic Factor 6
5808.	NDUFA1	NADH:Ubiquinone Oxidoreductase Subunit A1
5809.	NDUFA10	NADH:Ubiquinone Oxidoreductase Subunit A10
5810.	NDUFA8	NADH:Ubiquinone Oxidoreductase Subunit A8
5811.	NDUFB10	NADH:Ubiquinone Oxidoreductase Subunit B10
5812.	NECTIN2	Nectin Cell Adhesion Molecule 2
5813.	NODAL	Nodal Growth Differentiation Factor
5814.	NPY5R	Neuropeptide Y Receptor Y5
5815.	NRG2	Neuregulin 2
5816.	NUDT1	Nudix Hydrolase 1
5817.	PADI2	Peptidyl Arginine Deiminase 2
5818.	PAPOLA	Poly(A) Polymerase Alpha
5819.	PCSK7	Proprotein Convertase Subtilisin/Kexin Type 7
5820.	PDGFRL	Platelet Derived Growth Factor Receptor Like
5821.	PGC	Progastricsin
5822.	PIK3R5	Phosphoinositide-3-Kinase Regulatory Subunit 5
5823.	PIP5K1B	Phosphatidylinositol-4-Phosphate 5-Kinase Type 1 Beta
5824.	PMPCB	Peptidase, Mitochondrial Processing Subunit Beta
5825.	PNPLA3	Patatin Like Phospholipase Domain Containing 3
5826.	PSMB2	Proteasome 20S Subunit Beta 2
5827.	QPCT	Glutaminyl-Peptide Cyclotransferase
5828.	RAB3A	RAB3A, Member RAS Oncogene Family
5829.	RBM8A	RNA Binding Motif Protein 8A
5830.	RCC1	Regulator Of Chromosome Condensation 1
5831.	RERE	Arginine-Glutamic Acid Dipeptide Repeats
5832.	RUVBL2	RuvB Like AAA ATPase 2
5833.	SGCA	Sarcoglycan Alpha
5834.	SLC26A5	Solute Carrier Family 26 Member 5
5835.	SMC4	Structural Maintenance Of Chromosomes 4
5836.	SNAP91	Synaptosome Associated Protein 91
5837.	SNRPB	Small Nuclear Ribonucleoprotein Polypeptides B And B1
5838.	SSB	Small RNA Binding Exonuclease Protection Factor La
5839.	ST3GAL4	ST3 Beta-Galactoside Alpha-2,3-Sialyltransferase 4
5840.	SYCP3	Synaptonemal Complex Protein 3

5841.	TARS1	Threonyl-TRNA Synthetase 1
5842.	TMED10	Transmembrane P24 Trafficking Protein 10
5843.	UCP3	Uncoupling Protein 3
5844.	VAPA	VAMP Associated Protein A
5845.	VAV3	Vav Guanine Nucleotide Exchange Factor 3
5846.	AADAC	Arylacetamide Deacetylase
5847.	ABHD12	Abhydrolase Domain Containing 12, Lysophospholipase
5848.	ADAM19	ADAM Metallopeptidase Domain 19
5849.	AP2B1	Adaptor Related Protein Complex 2 Subunit Beta 1
5850.	APH1B	Aph-1 Homolog B, Gamma-Secretase Subunit
5851.	ARHGAP4	Rho GTPase Activating Protein 4
5852.	ASCC1	Activating Signal Cointegrator 1 Complex Subunit 1
5853.	ATP11A	ATPase Phospholipid Transporting 11A
5854.	ATP5F1B	ATP Synthase F1 Subunit Beta
5855.	AUH	AU RNA Binding Methylglutaconyl-CoA Hydratase
5856.	CAPNS1	Calpain Small Subunit 1
5857.	CCNE2	Cyclin E2
5858.	CDK5RAP2	CDK5 Regulatory Subunit Associated Protein 2
5859.	CLSPN	Claspin
5860.	CNTN4	Contactin 4
5861.	CNTN6	Contactin 6
5862.	CRELD1	Cysteine Rich With EGF Like Domains 1
5863.	DBN1	Drebrin 1
5864.	DGAT2	Diacylglycerol O-Acyltransferase 2
5865.	DHDDS	Dehydrodolichyl Diphosphate Synthase Subunit
5866.	DHH	Desert Hedgehog Signaling Molecule
5867.	DLG3	Discs Large MAGUK Scaffold Protein 3
5868.	DLGAP1	DLG Associated Protein 1
5869.	DNMBP	Dynamin Binding Protein
5870.	DSC3	Desmocollin 3
5871.	DYNC1I2	Dynein Cytoplasmic 1 Intermediate Chain 2
5872.	FIBP	FGF1 Intracellular Binding Protein
5873.	FOXO4	Forkhead Box O4
5874.	GIT1	GIT ArfGAP 1
5875.	GRID1	Glutamate Ionotropic Receptor Delta Type Subunit 1
5876.	HCCS	Holocytochrome C Synthase
5877.	HNRNPC	Heterogeneous Nuclear Ribonucleoprotein C
5878.	HNRNPH1	Heterogeneous Nuclear Ribonucleoprotein H1
5879.	HOXB1	Homeobox B1
5880.	KCNMB1	Potassium Calcium-Activated Channel Subfamily M Regulatory Beta Subunit 1
5881.	KCNN2	Potassium Calcium-Activated Channel Subfamily N Member 2

5882.	LMX1A	LIM Homeobox Transcription Factor 1 Alpha
5883.	LNX1	Ligand Of Numb-Protein X 1
5884.	MEGF10	Multiple EGF Like Domains 10
5885.	MVP	Major Vault Protein
5886.	NDUFA4	NDUFA4 Mitochondrial Complex Associated
5887.	NLGN2	Neuroligin 2
5888.	ODF2	Outer Dense Fiber Of Sperm Tails 2
5889.	PIGR	Polymeric Immunoglobulin Receptor
5890.	PREPL	Prolyl Endopeptidase Like
5891.	PSMB6	Proteasome 20S Subunit Beta 6
5892.	PSMC2	Proteasome 26S Subunit, ATPase 2
5893.	PSMD8	Proteasome 26S Subunit, Non-ATPase 8
5894.	PZP	PZP Alpha-2-Macroglobulin Like
5895.	RANGAP1	Ran GTPase Activating Protein 1
5896.	RP2	RP2 Activator Of ARL3 GTPase
5897.	RTN3	Reticulon 3
5898.	RYR3	Ryanodine Receptor 3
5899.	SFTPC	Surfactant Protein C
5900.	SH3GL3	SH3 Domain Containing GRB2 Like 3, Endophilin A3
5901.	SLC22A8	Solute Carrier Family 22 Member 8
5902.	SLC26A8	Solute Carrier Family 26 Member 8
5903.	SNAP29	Synaptosome Associated Protein 29
5904.	SNRPE	Small Nuclear Ribonucleoprotein Polypeptide E
5905.	SYVN1	Synoviolin 1
5906.	TCAP	Titin-Cap
5907.	TCERG1	Transcription Elongation Regulator 1
5908.	TCL1A	TCL1 Family AKT Coactivator A
5909.	TLN1	Talin 1
5910.	TMPRSS3	Transmembrane Serine Protease 3
5911.	TNPO1	Transportin 1
5912.	TOPBP1	DNA Topoisomerase II Binding Protein 1
5913.	TRIP4	Thyroid Hormone Receptor Interactor 4
5914.	TUBA1C	Tubulin Alpha 1c
5915.	UBE2G2	Ubiquitin Conjugating Enzyme E2 G2
5916.	UBE2J1	Ubiquitin Conjugating Enzyme E2 J1
5917.	UBE2K	Ubiquitin Conjugating Enzyme E2 K
5918.	UFM1	Ubiquitin Fold Modifier 1
5919.	ZMYND11	Zinc Finger MYND-Type Containing 11
5920.	ABCB10	ATP Binding Cassette Subfamily B Member 10
5921.	ACBD5	Acyl-CoA Binding Domain Containing 5
5922.	AGGF1	Angiogenic Factor With G-Patch And FHA Domains 1

5923.	ANKS1B	Ankyrin Repeat And Sterile Alpha Motif Domain Containing 1B
5924.	ANO6	Anoctamin 6
5925.	ATP5MC3	ATP Synthase Membrane Subunit C Locus 3
5926.	BCL3	BCL3 Transcription Coactivator
5927.	CADM3	Cell Adhesion Molecule 3
5928.	CD6	CD6 Molecule
5929.	CELF1	CUGBP Elav-Like Family Member 1
5930.	CYFIP1	Cytoplasmic FMR1 Interacting Protein 1
5931.	CYP4A11	Cytochrome P450 Family 4 Subfamily A Member 11
5932.	DHRS9	Dehydrogenase/Reductase 9
5933.	GSTA4	Glutathione S-Transferase Alpha 4
5934.	HNF4G	Hepatocyte Nuclear Factor 4 Gamma
5935.	HNRNPR	Heterogeneous Nuclear Ribonucleoprotein R
5936.	IL17B	Interleukin 17B
5937.	KIFAP3	Kinesin Associated Protein 3
5938.	LEFTY1	Left-Right Determination Factor 1
5939.	LIPT1	Lipoyltransferase 1
5940.	MBD1	Methyl-CpG Binding Domain Protein 1
5941.	NAAA	N-Acylethanolamine Acid Amidase
5942.	NDUFA2	NADH:Ubiquinone Oxidoreductase Subunit A2
5943.	NEB	Nebulin
5944.	PARL	Presenilin Associated Rhomboid Like
5945.	PPCDC	Phosphopantothenoylcysteine Decarboxylase
5946.	PPP1R9B	Protein Phosphatase 1 Regulatory Subunit 9B
5947.	PRG2	Proteoglycan 2, Pro Eosinophil Major Basic Protein
5948.	PSMB3	Proteasome 20S Subunit Beta 3
5949.	PSMD6	Proteasome 26S Subunit, Non-ATPase 6
5950.	PTPN5	Protein Tyrosine Phosphatase Non-Receptor Type 5
5951.	RARRES1	Retinoic Acid Receptor Responder 1
5952.	RFX1	Regulatory Factor X1
5953.	RPL7A	Ribosomal Protein L7a
5954.	SGCB	Sarcoglycan Beta
5955.	SLC22A11	Solute Carrier Family 22 Member 11
5956.	SLC6A11	Solute Carrier Family 6 Member 11
5957.	SLC8A2	Solute Carrier Family 8 Member A2
5958.	SMCHD1	Structural Maintenance Of Chromosomes Flexible Hinge Domain Containing 1
5959.	TBCE	Tubulin Folding Cofactor E
5960.	TRAPPC6B	Trafficking Protein Particle Complex Subunit 6B
5961.	TXN2	Thioredoxin 2
5962.	UBE2E3	Ubiquitin Conjugating Enzyme E2 E3
5963.	UBE2J2	Ubiquitin Conjugating Enzyme E2 J2

5964.	USP24	Ubiquitin Specific Peptidase 24
5965.	WDR35	WD Repeat Domain 35
5966.	XK	X-Linked Kx Blood Group Antigen, Kell And VPS13A Binding Protein
5967.	ADGRL3	Adhesion G Protein-Coupled Receptor L3
5968.	ALDH1L1	Aldehyde Dehydrogenase 1 Family Member L1
5969.	ALG14	ALG14 UDP-N-Acetylglucosaminyltransferase Subunit
5970.	ANO5	Anoctamin 5
5971.	AOPEP	Aminopeptidase O (Putative)
5972.	APBA3	Amyloid Beta Precursor Protein Binding Family A Member 3
5973.	APLP1	Amyloid Beta Precursor Like Protein 1
5974.	ATG12	Autophagy Related 12
5975.	AZIN2	Antizyme Inhibitor 2
5976.	BBS5	Bardet-Biedl Syndrome 5
5977.	BOC	BOC Cell Adhesion Associated, Oncogene Regulated
5978.	CADM2	Cell Adhesion Molecule 2
5979.	CCBE1	Collagen And Calcium Binding EGF Domains 1
5980.	CDK11B	Cyclin Dependent Kinase 11B
5981.	CEP120	Centrosomal Protein 120
5982.	CHD5	Chromodomain Helicase DNA Binding Protein 5
5983.	CNKSR2	Connector Enhancer Of Kinase Suppressor Of Ras 2
5984.	CNNM4	Cyclin And CBS Domain Divalent Metal Cation Transport Mediator 4
5985.	COL13A1	Collagen Type XIII Alpha 1 Chain
5986.	COPS3	COP9 Signalosome Subunit 3
5987.	CPZ	Carboxypeptidase Z
5988.	CSGALNACT1	Chondroitin Sulfate N-Acetylgalactosaminyltransferase 1
5989.	CYTH2	Cytohesin 2
5990.	DHRS3	Dehydrogenase/Reductase 3
5991.	DNAJC7	DnaJ Heat Shock Protein Family (Hsp40) Member C7
5992.	DYNC2LI1	Dynein Cytoplasmic 2 Light Intermediate Chain 1
5993.	FAM20A	FAM20A Golgi Associated Secretory Pathway Pseudokinase
5994.	FCER1G	Fc Epsilon Receptor Ig
5995.	FOXH1	Forkhead Box H1
5996.	GALNT11	Polypeptide N-Acetylgalactosaminyltransferase 11
5997.	GALR2	Galanin Receptor 2
5998.	GCKR	Glucokinase Regulator
5999.	GFM2	GTP Dependent Ribosome Recycling Factor Mitochondrial 2
6000.	GOLM1	Golgi Membrane Protein 1
6001.	HNRNPH2	Heterogeneous Nuclear Ribonucleoprotein H2
6002.	IFT43	Intraflagellar Transport 43
6003.	ISCA2	Iron-Sulfur Cluster Assembly 2
6004.	ISG20	Interferon Stimulated Exonuclease Gene 20

6005.	KCTD17	Potassium Channel Tetramerization Domain Containing 17
6006.	LIN7A	Lin-7 Homolog A, Crumbs Cell Polarity Complex Component
6007.	LSM1	LSM1 Homolog, MRNA Degradation Associated
6008.	LSM4	LSM4 Homolog, U6 Small Nuclear RNA And MRNA Degradation Associated
6009.	MANF	Mesencephalic Astrocyte Derived Neurotrophic Factor
6010.	MED13	Mediator Complex Subunit 13
6011.	MEOX2	Mesenchyme Homeobox 2
6012.	MIP	Major Intrinsic Protein Of Lens Fiber
6013.	MMP21	Matrix Metallopeptidase 21
6014.	MUC5AC	Mucin 5AC, Oligomeric Mucus/Gel-Forming
6015.	MYF5	Myogenic Factor 5
6016.	NCKAP1	NCK Associated Protein 1
6017.	NDUFB11	NADH:Ubiquinone Oxidoreductase Subunit B11
6018.	NDUFB7	NADH:Ubiquinone Oxidoreductase Subunit B7
6019.	NDUFC2	NADH:Ubiquinone Oxidoreductase Subunit C2
6020.	NEDD8	NEDD8 Ubiquitin Like Modifier
6021.	NELL2	Neural EGFL Like 2
6022.	NUBPL	NUBP Iron-Sulfur Cluster Assembly Factor, Mitochondrial
6023.	PAEP	Progestagen Associated Endometrial Protein
6024.	PAXIP1	PAX Interacting Protein 1
6025.	PIGN	Phosphatidylinositol Glycan Anchor Biosynthesis Class N
6026.	PITX3	Paired Like Homeodomain 3
6027.	PLCZ1	Phospholipase C Zeta 1
6028.	PSMD13	Proteasome 26S Subunit, Non-ATPase 13
6029.	RFX2	Regulatory Factor X2
6030.	RFX3	Regulatory Factor X3
6031.	SCFD1	Sec1 Family Domain Containing 1
6032.	SLC17A1	Solute Carrier Family 17 Member 1
6033.	SLC38A2	Solute Carrier Family 38 Member 2
6034.	SLC9A9	Solute Carrier Family 9 Member A9
6035.	SLITRK2	SLIT And NTRK Like Family Member 2
6036.	SNX5	Sorting Nexin 5
6037.	SRSF7	Serine And Arginine Rich Splicing Factor 7
6038.	SYT11	Synaptotagmin 11
6039.	TIAL1	TIA1 Cytotoxic Granule Associated RNA Binding Protein Like 1
6040.	TMPRSS4	Transmembrane Serine Protease 4
6041.	TMTC1	Transmembrane O-Mannosyltransferase Targeting Cadherins 1
6042.	TNFRSF19	TNF Receptor Superfamily Member 19
6043.	TNRC6B	Trinucleotide Repeat Containing Adaptor 6B
6044.	TOR1AIP1	Torsin 1A Interacting Protein 1
6045.	TRA2B	Transformer 2 Beta Homolog

6046.	UBE2E2	Ubiquitin Conjugating Enzyme E2 E2
6047.	UBE4B	Ubiquitination Factor E4B
6048.	ZPR1	ZPR1 Zinc Finger
6049.	AK7	Adenylate Kinase 7
6050.	AKAP3	A-Kinase Anchoring Protein 3
6051.	ANO10	Anoctamin 10
6052.	ANO2	Anoctamin 2
6053.	APBB2	Amyloid Beta Precursor Protein Binding Family B Member 2
6054.	ARHGAP32	Rho GTPase Activating Protein 32
6055.	ATL3	Atlastin GTPase 3
6056.	C1D	C1D Nuclear Receptor Corepressor
6057.	C2CD3	C2 Domain Containing 3 Centriole Elongation Regulator
6058.	CASZ1	Castor Zinc Finger 1
6059.	CATSPER1	Cation Channel Sperm Associated 1
6060.	CCL8	C-C Motif Chemokine Ligand 8
6061.	CDK11A	Cyclin Dependent Kinase 11A
6062.	CELSR3	Cadherin EGF LAG Seven-Pass G-Type Receptor 3
6063.	CENPB	Centromere Protein B
6064.	CEP135	Centrosomal Protein 135
6065.	CFC1	Cryptic, EGF-CFC Family Member 1
6066.	CLIP2	CAP-Gly Domain Containing Linker Protein 2
6067.	CLSTN1	Calsyntenin 1
6068.	CYP4F3	Cytochrome P450 Family 4 Subfamily F Member 3
6069.	DAPK2	Death Associated Protein Kinase 2
6070.	DCAF17	DDB1 And CUL4 Associated Factor 17
6071.	DGKH	Diacylglycerol Kinase Eta
6072.	DISP1	Dispatched RND Transporter Family Member 1
6073.	DPY19L2	Dpy-19 Like 2
6074.	DYNLRB2	Dynein Light Chain Roadblock-Type 2
6075.	EIF3M	Eukaryotic Translation Initiation Factor 3 Subunit M
6076.	GAL3ST1	Galactose-3-O-Sulfotransferase 1
6077.	GAR1	GAR1 Ribonucleoprotein
6078.	GEMIN4	Gem Nuclear Organelle Associated Protein 4
6079.	GLG1	Golgi Glycoprotein 1
6080.	HIP1R	Huntingtin Interacting Protein 1 Related
6081.	HNRNPA3	Heterogeneous Nuclear Ribonucleoprotein A3
6082.	HOXA2	Homeobox A2
6083.	HRK	Harakiri, BCL2 Interacting Protein
6084.	HS3ST3B1	Heparan Sulfate-Glucosamine 3-Sulfotransferase 3B1
6085.	IFT74	Intraflagellar Transport 74
6086.	IQSEC1	IQ Motif And Sec7 Domain ArfGEF 1

6087.	IRF2BPL	Interferon Regulatory Factor 2 Binding Protein Like
6088.	ISCA1	Iron-Sulfur Cluster Assembly 1
6089.	ITGA11	Integrin Subunit Alpha 11
6090.	IVL	Involucrin
6091.	KANSL1	KAT8 Regulatory NSL Complex Subunit 1
6092.	KCNIP3	Potassium Voltage-Gated Channel Interacting Protein 3
6093.	KCNS1	Potassium Voltage-Gated Channel Modifier Subfamily S Member 1
6094.	LIN7C	Lin-7 Homolog C, Crumbs Cell Polarity Complex Component
6095.	LSM3	LSM3 Homolog, U6 Small Nuclear RNA And MRNA Degradation Associated
6096.	LYST	Lysosomal Trafficking Regulator
6097.	MAP1A	Microtubule Associated Protein 1A
6098.	MTMR1	Myotubularin Related Protein 1
6099.	NDUFAF3	NADH:Ubiquinone Oxidoreductase Complex Assembly Factor 3
6100.	NEUROG2	Neurogenin 2
6101.	NHLH2	Nescient Helix-Loop-Helix 2
6102.	NKX6-2	NK6 Homeobox 2
6103.	NME7	NME/NM23 Family Member 7
6104.	NPY4R	Neuropeptide Y Receptor Y4
6105.	PAPOLG	Poly(A) Polymerase Gamma
6106.	PCMT1	Protein-L-Isoaspartate (D-Aspartate) O-Methyltransferase
6107.	PIR	Pirin
6108.	PLA2G3	Phospholipase A2 Group III
6109.	RAB18	RAB18, Member RAS Oncogene Family
6110.	RELT	RELT TNF Receptor
6111.	RIF1	Replication Timing Regulatory Factor 1
6112.	RSU1	Ras Suppressor Protein 1
6113.	SASH1	SAM And SH3 Domain Containing 1
6114.	SH2D2A	SH2 Domain Containing 2A
6115.	SLC22A13	Solute Carrier Family 22 Member 13
6116.	SLC35B2	Solute Carrier Family 35 Member B2
6117.	SLITRK5	SLIT And NTRK Like Family Member 5
6118.	SNRPD1	Small Nuclear Ribonucleoprotein D1 Polypeptide
6119.	SNRPD2	Small Nuclear Ribonucleoprotein D2 Polypeptide
6120.	SNX1	Sorting Nexin 1
6121.	SRSF3	Serine And Arginine Rich Splicing Factor 3
6122.	STRAP	Serine/Threonine Kinase Receptor Associated Protein
6123.	TAAR1	Trace Amine Associated Receptor 1
6124.	TCTN1	Tectonic Family Member 1
6125.	TLN2	Talin 2
6126.	TRAF3IP1	TRAF3 Interacting Protein 1
6127.	TRIM9	Tripartite Motif Containing 9

6128.	TTBK1	Tau Tubulin Kinase 1
6129.	TXNIP	Thioredoxin Interacting Protein
6130.	UBE2L6	Ubiquitin Conjugating Enzyme E2 L6
6131.	ZDHHC13	Zinc Finger DHHC-Type Palmitoyltransferase 13
6132.	ADGRG2	Adhesion G Protein-Coupled Receptor G2
6133.	AK8	Adenylate Kinase 8
6134.	ARID3B	AT-Rich Interaction Domain 3B
6135.	ASIC4	Acid Sensing Ion Channel Subunit Family Member 4
6136.	ATP8A2	ATPase Phospholipid Transporting 8A2
6137.	BTG1	BTG Anti-Proliferation Factor 1
6138.	CALY	Calcyon Neuron Specific Vesicular Protein
6139.	CCDC115	Coiled-Coil Domain Containing 115
6140.	CDNF	Cerebral Dopamine Neurotrophic Factor
6141.	CETN1	Centrin 1
6142.	CH25H	Cholesterol 25-Hydroxylase
6143.	CLDN6	Claudin 6
6144.	CLEC1B	C-Type Lectin Domain Family 1 Member B
6145.	CNTN3	Contactin 3
6146.	COL25A1	Collagen Type XXV Alpha 1 Chain
6147.	COTL1	Coactosin Like F-Actin Binding Protein 1
6148.	CRY2	Cryptochrome Circadian Regulator 2
6149.	DLX6	Distal-Less Homeobox 6
6150.	DTX2	Deltex E3 Ubiquitin Ligase 2
6151.	ENC1	Ectodermal-Neural Cortex 1
6152.	EPB41L2	Erythrocyte Membrane Protein Band 4.1 Like 2
6153.	ERAL1	Era Like 12S Mitochondrial RRNA Chaperone 1
6154.	ERBIN	Erbb2 Interacting Protein
6155.	EVC	EvC Ciliary Complex Subunit 1
6156.	EXTL1	Exostosin Like Glycosyltransferase 1
6157.	FBXO31	F-Box Protein 31
6158.	FCHO1	FCH And Mu Domain Containing Endocytic Adaptor 1
6159.	FCRL3	Fc Receptor Like 3
6160.	FGGY	FGGY Carbohydrate Kinase Domain Containing
6161.	FREM2	FRAS1 Related Extracellular Matrix 2
6162.	GLRX2	Glutaredoxin 2
6163.	GPM6B	Glycoprotein M6B
6164.	HBZ	Hemoglobin Subunit Zeta
6165.	HLA-F	Major Histocompatibility Complex, Class I, F
6166.	HOXD10	Homeobox D10
6167.	IER3	Immediate Early Response 3
6168.	IL31	Interleukin 31

6169.	KCNMB4	Potassium Calcium-Activated Channel Subfamily M Regulatory Beta Subunit 4
6170.	KLHL40	Kelch Like Family Member 40
6171.	LIN7B	Lin-7 Homolog B, Crumbs Cell Polarity Complex Component
6172.	LYAR	Ly1 Antibody Reactive
6173.	MAGI1	Membrane Associated Guanylate Kinase, WW And PDZ Domain Containing 1
6174.	MNS1	Meiosis Specific Nuclear Structural 1
6175.	MPHOSPH8	M-Phase Phosphoprotein 8
6176.	MTMR7	Myotubularin Related Protein 7
6177.	NAT8L	N-Acetyltransferase 8 Like
6178.	NAXD	NAD(P)HX Dehydratase
6179.	NBL1	NBL1, DAN Family BMP Antagonist
6180.	NEMF	Nuclear Export Mediator Factor
6181.	NEU4	Neuraminidase 4
6182.	NIPA2	NIPA Magnesium Transporter 2
6183.	NLGN4Y	Neuroligin 4 Y-Linked
6184.	NSRP1	Nuclear Speckle Splicing Regulatory Protein 1
6185.	NUP54	Nucleoporin 54
6186.	OLFM1	Olfactomedin 1
6187.	PFN2	Profilin 2
6188.	PPP6R2	Protein Phosphatase 6 Regulatory Subunit 2
6189.	PRDM6	PR/SET Domain 6
6190.	PRTG	Protogenin
6191.	RAB3IP	RAB3A Interacting Protein
6192.	RABEP1	Rabaptin, RAB GTPase Binding Effector Protein 1
6193.	RAP1GAP	RAP1 GTPase Activating Protein
6194.	RASD2	RASD Family Member 2
6195.	SEPTIN2	Septin 2
6196.	SEPTIN5	Septin 5
6197.	SLC30A6	Solute Carrier Family 30 Member 6
6198.	SMYD1	SET And MYND Domain Containing 1
6199.	SNAPIN	SNAP Associated Protein
6200.	SNX2	Sorting Nexin 2
6201.	SNX3	Sorting Nexin 3
6202.	SOX1	SRY-Box Transcription Factor 1
6203.	SRRM2	Serine/Arginine Repetitive Matrix 2
6204.	SRY	Sex Determining Region Y
6205.	SV2B	Synaptic Vesicle Glycoprotein 2B
6206.	SYNCRIP	Synaptotagmin Binding Cytoplasmic RNA Interacting Protein
6207.	TIMM13	Translocase Of Inner Mitochondrial Membrane 13
6208.	TMBIM6	Transmembrane BAX Inhibitor Motif Containing 6
6209.	TOR2A	Torsin Family 2 Member A

6210.	TOX3	TOX High Mobility Group Box Family Member 3
6211.	ULBP1	UL16 Binding Protein 1
6212.	ULBP2	UL16 Binding Protein 2
6213.	UQCC2	Ubiquinol-Cytochrome C Reductase Complex Assembly Factor 2
6214.	VPS39	VPS39 Subunit Of HOPS Complex
6215.	WWTR1	WW Domain Containing Transcription Regulator 1
6216.	ZDHHC17	Zinc Finger DHHC-Type Palmitoyltransferase 17
6217.	AAR2	AAR2 Splicing Factor
6218.	AATK	Apoptosis Associated Tyrosine Kinase
6219.	ACAP3	ArfGAP With Coiled-Coil, Ankyrin Repeat And PH Domains 3
6220.	AHNAK	AHNAK Nucleoprotein
6221.	AKAP4	A-Kinase Anchoring Protein 4
6222.	ATP5PD	ATP Synthase Peripheral Stalk Subunit D
6223.	AVIL	Advillin
6224.	BCL2L12	BCL2 Like 12
6225.	CACYBP	Calcyclin Binding Protein
6226.	CCL18	C-C Motif Chemokine Ligand 18
6227.	CENPS	Centromere Protein S
6228.	CEP83	Centrosomal Protein 83
6229.	CEP97	Centrosomal Protein 97
6230.	CFAP53	Cilia And Flagella Associated Protein 53
6231.	CKAP2L	Cytoskeleton Associated Protein 2 Like
6232.	CLUAP1	Clusterin Associated Protein 1
6233.	CRTAC1	Cartilage Acidic Protein 1
6234.	CSH1	Chorionic Somatomammotropin Hormone 1
6235.	DERL1	Derlin 1
6236.	DIRAS2	DIRAS Family GTPase 2
6237.	DPCD	Deleted In Primary Ciliary Dyskinesia Homolog (Mouse)
6238.	DPY30	Dpy-30 Histone Methyltransferase Complex Regulatory Subunit
6239.	DRC3	Dynein Regulatory Complex Subunit 3
6240.	DYNC2I1	Dynein 2 Intermediate Chain 1
6241.	DYNC2I2	Dynein 2 Intermediate Chain 2
6242.	DYNLL2	Dynein Light Chain LC8-Type 2
6243.	FRMD6	FERM Domain Containing 6
6244.	GABRR3	Gamma-Aminobutyric Acid Type A Receptor Subunit Rho3
6245.	GDF1	Growth Differentiation Factor 1
6246.	GNPTG	N-Acetylglucosamine-1-Phosphate Transferase Subunit Gamma
6247.	GSE1	Gse1 Coiled-Coil Protein
6248.	HIKESHI	Heat Shock Protein Nuclear Import Factor Hikeshi
6249.	HOXD8	Homeobox D8
6250.	IDO2	Indoleamine 2,3-Dioxygenase 2

6251.	JOSD1	Josephin Domain Containing 1
6252.	KCNMB3	Potassium Calcium-Activated Channel Subfamily M Regulatory Beta Subunit 3
6253.	KIF21B	Kinesin Family Member 21B
6254.	LRIG3	Leucine Rich Repeats And Immunoglobulin Like Domains 3
6255.	LSM5	LSM5 Homolog, U6 Small Nuclear RNA And MRNA Degradation Associated
6256.	LSM6	LSM6 Homolog, U6 Small Nuclear RNA And MRNA Degradation Associated
6257.	MIEF2	Mitochondrial Elongation Factor 2
6258.	MLEC	Malectin
6259.	MMP23B	Matrix Metallopeptidase 23B
6260.	MPHOSPH9	M-Phase Phosphoprotein 9
6261.	ODF1	Outer Dense Fiber Of Sperm Tails 1
6262.	P3H3	Prolyl 3-Hydroxylase 3
6263.	PCDHA3	Protocadherin Alpha 3
6264.	PF4V1	Platelet Factor 4 Variant 1
6265.	PLEKHB2	Pleckstrin Homology Domain Containing B2
6266.	PM20D1	Peptidase M20 Domain Containing 1
6267.	POC1A	POC1 Centriolar Protein A
6268.	POLR2K	RNA Polymerase II, I And III Subunit K
6269.	PPP1R10	Protein Phosphatase 1 Regulatory Subunit 10
6270.	PTCHD1	Patched Domain Containing 1
6271.	QSOX2	Quiescin Sulfhydryl Oxidase 2
6272.	RMDN3	Regulator Of Microtubule Dynamics 3
6273.	RXYLT1	Ribitol Xylosyltransferase 1
6274.	SASS6	SAS-6 Centriolar Assembly Protein
6275.	SEPTIN12	Septin 12
6276.	SIPA1L2	Signal Induced Proliferation Associated 1 Like 2
6277.	SLC28A2	Solute Carrier Family 28 Member 2
6278.	SNRPB2	Small Nuclear Ribonucleoprotein Polypeptide B2
6279.	SNRPD3	Small Nuclear Ribonucleoprotein D3 Polypeptide
6280.	SNRPG	Small Nuclear Ribonucleoprotein Polypeptide G
6281.	SNX14	Sorting Nexin 14
6282.	SORCS2	Sortilin Related VPS10 Domain Containing Receptor 2
6283.	SORCS3	Sortilin Related VPS10 Domain Containing Receptor 3
6284.	TEKT1	Tektin 1
6285.	TENM1	Teneurin Transmembrane Protein 1
6286.	TEX11	Testis Expressed 11
6287.	TMEM237	Transmembrane Protein 237
6288.	TRAPPC11	Trafficking Protein Particle Complex Subunit 11
6289.	UBL4A	Ubiquitin Like 4A
6290.	WDR72	WD Repeat Domain 72
6291.	YIF1B	Yip1 Interacting Factor Homolog B, Membrane Trafficking Protein

6292.	ZNF292	Zinc Finger Protein 292
6293.	ZNF746	Zinc Finger Protein 746
6294.	ZNF804A	Zinc Finger Protein 804A
6295.	CARD16	Caspase Recruitment Domain Family Member 16
6296.	CCDC47	Coiled-Coil Domain Containing 47
6297.	CCDC62	Coiled-Coil Domain Containing 62
6298.	CCDC8	Coiled-Coil Domain Containing 8
6299.	CD24	CD24 Molecule
6300.	CDC14B	Cell Division Cycle 14B
6301.	CDIP1	Cell Death Inducing P53 Target 1
6302.	CFAP65	Cilia And Flagella Associated Protein 65
6303.	CHERP	Calcium Homeostasis Endoplasmic Reticulum Protein
6304.	CNTLN	Centlein
6305.	DHX32	DEAH-Box Helicase 32 (Putative)
6306.	DMXL2	Dmx Like 2
6307.	DNAH17	Dynein Axonemal Heavy Chain 17
6308.	DNAJC30	DnaJ Heat Shock Protein Family (Hsp40) Member C30
6309.	FAM107A	Family With Sequence Similarity 107 Member A
6310.	FAM83H	Family With Sequence Similarity 83 Member H
6311.	FBXL13	F-Box And Leucine Rich Repeat Protein 13
6312.	GEMIN8	Gem Nuclear Organelle Associated Protein 8
6313.	GIGYF1	GRB10 Interacting GYF Protein 1
6314.	IFT25	Intraflagellar Transport 25
6315.	IFT70B	Intraflagellar Transport 70B
6316.	INPP5F	Inositol Polyphosphate-5-Phosphatase F
6317.	JOSD2	Josephin Domain Containing 2
6318.	KCNMB2	Potassium Calcium-Activated Channel Subfamily M Regulatory Beta Subunit 2
6319.	KIF2B	Kinesin Family Member 2B
6320.	LHX6	LIM Homeobox 6
6321.	LYPD1	LY6/PLAUR Domain Containing 1
6322.	MAL	Mal, T Cell Differentiation Protein
6323.	MBNL3	Muscleblind Like Splicing Regulator 3
6324.	MIEF1	Mitochondrial Elongation Factor 1
6325.	MINDY1	MINDY Lysine 48 Deubiquitinase 1
6326.	MYL6B	Myosin Light Chain 6B
6327.	OSBPL7	Oxysterol Binding Protein Like 7
6328.	PIH1D1	PIH1 Domain Containing 1
6329.	PPHLN1	Periphilin 1
6330.	PRDM12	PR/SET Domain 12
6331.	PROK1	Prokineticin 1
6332.	RAB9B	RAB9B, Member RAS Oncogene Family

6333.	RNASE4	Ribonuclease A Family Member 4
6334.	ROPN1L	Rhophilin Associated Tail Protein 1 Like
6335.	SEPTIN1	Septin 1
6336.	SERPINB4	Serpin Family B Member 4
6337.	SFXN5	Sideroflexin 5
6338.	SH2D1B	SH2 Domain Containing 1B
6339.	SKOR1	SKI Family Transcriptional Corepressor 1
6340.	SLC2A4RG	SLC2A4 Regulator
6341.	SMPX	Small Muscle Protein X-Linked
6342.	SNRPF	Small Nuclear Ribonucleoprotein Polypeptide F
6343.	SP140	SP140 Nuclear Body Protein
6344.	SPAG16	Sperm Associated Antigen 16
6345.	SPATA16	Spermatogenesis Associated 16
6346.	SPATA22	Spermatogenesis Associated 22
6347.	SSH2	Slingshot Protein Phosphatase 2
6348.	STAC3	SH3 And Cysteine Rich Domain 3
6349.	TAS2R38	Taste 2 Receptor Member 38
6350.	TEKT4	Tektin 4
6351.	TIMM10B	Translocase Of Inner Mitochondrial Membrane 10B
6352.	TIMM8B	Translocase Of Inner Mitochondrial Membrane 8 Homolog B
6353.	TM2D1	TM2 Domain Containing 1
6354.	TMEM163	Transmembrane Protein 163
6355.	TNFSF8	TNF Superfamily Member 8
6356.	TTC21A	Tetratricopeptide Repeat Domain 21A
6357.	TTC29	Tetratricopeptide Repeat Domain 29
6358.	TTLL3	Tubulin Tyrosine Ligase Like 3
6359.	TUBAL3	Tubulin Alpha Like 3
6360.	TUBGCP5	Tubulin Gamma Complex Component 5
6361.	UBE2W	Ubiquitin Conjugating Enzyme E2 W
6362.	ZNF346	Zinc Finger Protein 346
6363.	ANKRD50	Ankyrin Repeat Domain Containing 50
6364.	ARMC2	Armadillo Repeat Containing 2
6365.	ATXN7L1	Ataxin 7 Like 1
6366.	CALHM1	Calcium Homeostasis Modulator 1
6367.	CEP89	Centrosomal Protein 89
6368.	CFAP251	Cilia And Flagella Associated Protein 251
6369.	CFAP91	Cilia And Flagella Associated Protein 91
6370.	CMIP	C-Maf Inducing Protein
6371.	COA7	Cytochrome C Oxidase Assembly Factor 7
6372.	CSRNP3	Cysteine And Serine Rich Nuclear Protein 3
6373.	DCHS2	Dachsous Cadherin-Related 2

6374.	ESX1	ESX Homeobox 1
6375.	FITM2	Fat Storage Inducing Transmembrane Protein 2
6376.	FOXD4	Forkhead Box D4
6377.	GCN1	GCN1 Activator Of EIF2AK4
6378.	GFOD1	Gfo/Idh/MocA-Like Oxidoreductase Domain Containing 1
6379.	HPDL	4-Hydroxyphenylpyruvate Dioxygenase Like
6380.	HUNK	Hormonally Up-Regulated Neu-Associated Kinase
6381.	IQCA1	IQ Motif Containing With AAA Domain 1
6382.	KLHL14	Kelch Like Family Member 14
6383.	MFHAS1	Multifunctional ROCO Family Signaling Regulator 1
6384.	MTIF3	Mitochondrial Translational Initiation Factor 3
6385.	NAGPA	N-Acetylglucosamine-1-Phosphodiester Alpha-N-Acetylglucosaminidase
6386.	NDFIP2	Nedd4 Family Interacting Protein 2
6387.	NPAT	Nuclear Protein, Coactivator Of Histone Transcription
6388.	NSA2	NSA2 Ribosome Biogenesis Factor
6389.	OPALIN	Oligodendrocytic Myelin Paranodal And Inner Loop Protein
6390.	PI15	Peptidase Inhibitor 15
6391.	RAB11FIP3	RAB11 Family Interacting Protein 3
6392.	RIC1	RIC1 Homolog, RAB6A GEF Complex Partner 1
6393.	SAMD4B	Sterile Alpha Motif Domain Containing 4B
6394.	SLC38A5	Solute Carrier Family 38 Member 5
6395.	SUGP1	SURP And G-Patch Domain Containing 1
6396.	SYNC	Syncoilin, Intermediate Filament Protein
6397.	TBATA	Thymus, Brain And Testes Associated
6398.	TEKT2	Tektin 2
6399.	TOR3A	Torsin Family 3 Member A
6400.	TRAFD1	TRAF-Type Zinc Finger Domain Containing 1
6401.	TTC17	Tetratricopeptide Repeat Domain 17
6402.	TTC4	Tetratricopeptide Repeat Domain 4
6403.	TTLL1	TTL Family Tubulin Polyglutamylase Complex Subunit L1
6404.	TTLL9	Tubulin Tyrosine Ligase Like 9
6405.	UBQLN3	Ubiquilin 3
6406.	VPS26C	VPS26 Endosomal Protein Sorting Factor C
6407.	WASHC4	WASH Complex Subunit 4
6408.	WDR41	WD Repeat Domain 41
6409.	WDR74	WD Repeat Domain 74
6410.	ZACN	Zinc Activated Ion Channel
6411.	ZBTB48	Zinc Finger And BTB Domain Containing 48
6412.	ZNF142	Zinc Finger Protein 142
6413.	ZUP1	Zinc Finger Containing Ubiquitin Peptidase 1
6414.	AFG1L	AFG1 Like ATPase

6415.	CCDC63	Coiled-Coil Domain Containing 63
6416.	CFAP58	Cilia And Flagella Associated Protein 58
6417.	CFAP70	Cilia And Flagella Associated Protein 70
6418.	DCAKD	Dephospho-CoA Kinase Domain Containing
6419.	DOP1B	DOP1 Leucine Zipper Like Protein B
6420.	DUSP26	Dual Specificity Phosphatase 26
6421.	EFCAB2	EF-Hand Calcium Binding Domain 2
6422.	ENKUR	Enkurin, TRPC Channel Interacting Protein
6423.	ETAA1	ETAA1 Activator Of ATR Kinase
6424.	EXOC3L2	Exocyst Complex Component 3 Like 2
6425.	GSX2	GS Homeobox 2
6426.	GTF3C4	General Transcription Factor IIIC Subunit 4
6427.	IRX2	Iroquois Homeobox 2
6428.	KIAA0753	KIAA0753
6429.	LRGUK	Leucine Rich Repeats And Guanylate Kinase Domain Containing
6430.	MTRFR	Mitochondrial Translation Release Factor In Rescue
6431.	PLSCR4	Phospholipid Scramblase 4
6432.	RFX7	Regulatory Factor X7
6433.	RPAP3	RNA Polymerase II Associated Protein 3
6434.	RTBDN	Retbindin
6435.	SNX6	Sorting Nexin 6
6436.	TMEM8B	Transmembrane Protein 8B
6437.	TSGA10	Testis Specific 10
6438.	TUBG2	Tubulin Gamma 2
6439.	WDR7	WD Repeat Domain 7
6440.	ARMC12	Armadillo Repeat Containing 12
6441.	CCDC42	Coiled-Coil Domain Containing 42
6442.	CFAP20	Cilia And Flagella Associated Protein 20
6443.	CFAP52	Cilia And Flagella Associated Protein 52
6444.	CFAP61	Cilia And Flagella Associated Protein 61
6445.	CFAP69	Cilia And Flagella Associated Protein 69
6446.	DMRT3	Doublesex And Mab-3 Related Transcription Factor 3
6447.	DNAAF10	Dynein Axonemal Assembly Factor 10
6448.	FAM163A	Family With Sequence Similarity 163 Member A
6449.	FBF1	Fas Binding Factor 1
6450.	FSIP2	Fibrous Sheath Interacting Protein 2
6451.	IFT22	Intraflagellar Transport 22
6452.	IQCG	IQ Motif Containing G
6453.	IQUB	IQ Motif And Ubiquitin Domain Containing
6454.	LRRC17	Leucine Rich Repeat Containing 17
6455.	LRRC23	Leucine Rich Repeat Containing 23

6456.	MTCP1	Mature T Cell Proliferation 1
6457.	NBPF3	NBPF Member 3
6458.	PAPOLB	Poly(A) Polymerase Beta
6459.	PSD2	Pleckstrin And Sec7 Domain Containing 2
6460.	R3HCC1L	R3H Domain And Coiled-Coil Containing 1 Like
6461.	RNF32	Ring Finger Protein 32
6462.	SLF2	SMC5-SMC6 Complex Localization Factor 2
6463.	SMCR8	SMCR8-C9orf72 Complex Subunit
6464.	SSUH2	Ssu-2 Homolog
6465.	TASOR	Transcription Activation Suppressor
6466.	TMCO3	Transmembrane And Coiled-Coil Domains 3
6467.	TXNDC2	Thioredoxin Domain Containing 2
6468.	VPS35L	VPS35 Endosomal Protein Sorting Factor Like
6469.	ARMC3	Armadillo Repeat Containing 3
6470.	ARSH	Arylsulfatase Family Member H
6471.	CCDC146	Coiled-Coil Domain Containing 146
6472.	CCDC9	Coiled-Coil Domain Containing 9
6473.	CDR1	Cerebellar Degeneration Related 1
6474.	CFAP263	Cilia And Flagella Associated Protein 263
6475.	CFAP44	Cilia And Flagella Associated Protein 44
6476.	COA8	Cytochrome C Oxidase Assembly Factor 8
6477.	CSH2	Chorionic Somatomammotropin Hormone 2
6478.	DMXL1	Dmx Like 1
6479.	DNAH3	Dynein Axonemal Heavy Chain 3
6480.	DYNLT2	Dynein Light Chain Tctex-Type 2
6481.	ECHDC3	Enoyl-CoA Hydratase Domain Containing 3
6482.	GPATCH8	G-Patch Domain Containing 8
6483.	H3C14	H3 Clustered Histone 14
6484.	HEXD	Hexosaminidase D
6485.	KBTBD13	Kelch Repeat And BTB Domain Containing 13
6486.	LIME1	Lck Interacting Transmembrane Adaptor 1
6487.	MS4A4A	Membrane Spanning 4-Domains A4A
6488.	MTARC2	Mitochondrial Amidoxime Reducing Component 2
6489.	MTPN	Myotrophin
6490.	NHLH1	Nescient Helix-Loop-Helix 1
6491.	PIH1D2	PIH1 Domain Containing 2
6492.	PTRHD1	Peptidyl-TRNA Hydrolase Domain Containing 1
6493.	RIC8B	RIC8 Guanine Nucleotide Exchange Factor B
6494.	RUFY2	RUN And FYVE Domain Containing 2
6495.	SELENOT	Selenoprotein T
6496.	SPEM1	Spermatid Maturation 1

6497.	SYT12	Synaptotagmin 12
6498.	USP40	Ubiquitin Specific Peptidase 40
6499.	VPS26B	VPS26 Retromer Complex Component B
6500.	XKR6	XK Related 6
6501.	ZNF395	Zinc Finger Protein 395
6502.	AMTN	Amelotin
6503.	AP1AR	Adaptor Related Protein Complex 1 Associated Regulatory Protein
6504.	ARL13A	ADP Ribosylation Factor Like GTPase 13A
6505.	BTBD8	BTB Domain Containing 8
6506.	C2CD5	C2 Calcium Dependent Domain Containing 5
6507.	CCSAP	Centriole, Cilia And Spindle Associated Protein
6508.	CFAP47	Cilia And Flagella Associated Protein 47
6509.	CLDN8	Claudin 8
6510.	DCUN1D4	Defective In Cullin Neddylation 1 Domain Containing 4
6511.	DEUP1	Deuterosome Assembly Protein 1
6512.	DNAH14	Dynein Axonemal Heavy Chain 14
6513.	DNAH6	Dynein Axonemal Heavy Chain 6
6514.	DNAI3	Dynein Axonemal Intermediate Chain 3
6515.	DYNLT5	Dynein Light Chain Tctex-Type Family Member 5
6516.	ENTR1	Endosome Associated Trafficking Regulator 1
6517.	EPPK1	Epiplakin 1
6518.	FBXO33	F-Box Protein 33
6519.	GCNA	Germ Cell Nuclear Acidic Peptidase
6520.	H4C8	H4 Clustered Histone 8
6521.	IQCD	IQ Motif Containing D
6522.	LRRC46	Leucine Rich Repeat Containing 46
6523.	LRRC74A	Leucine Rich Repeat Containing 74A
6524.	MALL	Mal, T Cell Differentiation Protein Like
6525.	MIX23	Mitochondrial Matrix Import Factor 23
6526.	NDUFAF8	NADH:Ubiquinone Oxidoreductase Complex Assembly Factor 8
6527.	NME9	NME/NM23 Family Member 9
6528.	ODAPH	Odontogenesis Associated Phosphoprotein
6529.	PASD1	PAS Domain Containing Repressor 1
6530.	POLR2M	RNA Polymerase II Subunit M
6531.	PRAF2	PRA1 Domain Family Member 2
6532.	RBMY1A1	RNA Binding Motif Protein Y-Linked Family 1 Member A1
6533.	RIC3	RIC3 Acetylcholine Receptor Chaperone
6534.	SLC45A1	Solute Carrier Family 45 Member 1
6535.	SPEF1	Sperm Flagellar 1
6536.	TMEM158	Transmembrane Protein 158
6537.	TPPP2	Tubulin Polymerization Promoting Protein Family Member 2

6538.	TRIM66	Tripartite Motif Containing 66
6539.	CFAP184	Cilia And Flagella Associated Protein 184
6540.	CIMAP1A	Ciliary Microtubule Associated Protein 1A
6541.	CLDN17	Claudin 17
6542.	COPS9	COP9 Signalosome Subunit 9
6543.	DENND11	DENN Domain Containing 11
6544.	DIPK1C	Divergent Protein Kinase Domain 1C
6545.	DNAH12	Dynein Axonemal Heavy Chain 12
6546.	DNAI4	Dynein Axonemal Intermediate Chain 4
6547.	DRC7	Dynein Regulatory Complex Subunit 7
6548.	IHO1	Interactor Of HORMAD1 1
6549.	MALRD1	MAM And LDL Receptor Class A Domain Containing 1
6550.	MFSD14A	Major Facilitator Superfamily Domain Containing 14A
6551.	MROH8	Maestro Heat Like Repeat Family Member 8
6552.	SBSN	Suprabasin
6553.	TSPAN16	Tetraspanin 16
6554.	TVP23C	Trans-Golgi Network Vesicle Protein 23 Homolog C
6555.	WASHC1	WASH Complex Subunit 1
6556.	CCDC87	Coiled-Coil Domain Containing 87
6557.	CDCP2	CUB Domain Containing Protein 2
6558.	CDRT4	CMT1A Duplicated Region Transcript 4
6559.	CFAP276	Cilia And Flagella Associated Protein 276
6560.	CFAP92	Cilia And Flagella Associated Protein 92 (Putative)
6561.	LHFPL6	LHFPL Tetraspan Subfamily Member 6
6562.	NBPF1	NBPF Member 1
6563.	NBPF15	NBPF Member 15
6564.	NBPF6	NBPF Member 6
6565.	ODF4	Outer Dense Fiber Of Sperm Tails 4
6566.	PCIF1	Phosphorylated CTD Interacting Factor 1
6567.	PTPN20	Protein Tyrosine Phosphatase Non-Receptor Type 20
6568.	RFLNB	Refilin B
6569.	SDE2	SDE2 Telomere Maintenance Homolog
6570.	SERF1A	Small EDRK-Rich Factor 1A
6571.	TMEM187	Transmembrane Protein 187
6572.	TVP23A	Trans-Golgi Network Vesicle Protein 23 Homolog A
6573.	WDR93	WD Repeat Domain 93
6574.	CDRT15	CMT1A Duplicated Region Transcript 15
6575.	CFAP119	Cilia And Flagella Associated Protein 119
6576.	CFAP126	Cilia And Flagella Associated Protein 126
6577.	CFAP54	Cilia And Flagella Associated Protein 54
6578.	CRYGN	Crystallin Gamma N

6579.	DNAAF8	Dynein Axonemal Assembly Factor 8
6580.	GMNC	Geminin Coiled-Coil Domain Containing
6581.	NBPF11	NBPF Member 11
6582.	PRB4	Proline Rich Protein BstNI Subfamily 4
6583.	SAGE1	Sarcoma Antigen 1
6584.	TMEM35A	Transmembrane Protein 35A
6585.	TMSB15A	Thymosin Beta 15A
6586.	ZNF441	Zinc Finger Protein 441
6587.	NBPF12	NBPF Member 12
6588.	BEX5	Brain Expressed X-Linked 5
6589.	CFAP206	Cilia And Flagella Associated Protein 206
6590.	FBXW10B	F-Box And WD Repeat Domain Containing 10B
6591.	NBPF10	NBPF Member 10
6592.	NBPF4	NBPF Member 4
6593.	ZNF429	Zinc Finger Protein 429
6594.	CITED4	Cbp/P300 Interacting Transactivator With Glu/Asp Rich Carboxy-Terminal Domain 4
6595.	FBXO48	F-Box Protein 48
6596.	H4C15	H4 Clustered Histone 15
6597.	KRTAP12-2	Keratin Associated Protein 12-2
6598.	NBPF14	NBPF Member 14
6599.	OR52I2	Olfactory Receptor Family 52 Subfamily I Member 2
6600.	OPN1MW2	Opsin 1, Medium Wave Sensitive 2
6601.	OR2L3	Olfactory Receptor Family 2 Subfamily L Member 3
6602.	PAGE2	PAGE Family Member 2
6603.	SPATA31A1	SPATA31 Subfamily A Member 1
6604.	NBPF8	NBPF Member 8
6605.	MTLN	Mitoregulin
6606.	NBPF20	NBPF Member 20
6607.	TMEM35B	Transmembrane Protein 35B
6608.	MYMX	Myomixer, Myoblast Fusion Factor
6609.	CCDC192	Coiled-Coil Domain Containing 192
6610.	CFAP298-TCP10L	CFAP298-TCP10L Readthrough
6611.	MICOS10-NBL1	MICOS10-NBL1 Readthrough
6612.	OPN1MW3	Opsin 1, Medium Wave Sensitive 3
6613.	TVP23C-CDRT4	TVP23C-CDRT4 Readthrough
6614.	NBPF26	NBPF Member 26
6615.	ASDURF	ASNSD1 Upstream Open Reading Frame
6616.	ROR2	Receptor Tyrosine Kinase Like Orphan Receptor 2
6617.	CD22	CD22 Molecule
6618.	MTNR1B	Melatonin Receptor 1B
6619.	NRIP1	Nuclear Receptor Interacting Protein 1

6620.	USP1	Ubiquitin Specific Peptidase 1
6621.	CANT1	Calcium Activated Nucleotidase 1
6622.	CBR1	Carbonyl Reductase 1
6623.	CEACAM5	CEA Cell Adhesion Molecule 5
6624.	EYA1	EYA Transcriptional Coactivator And Phosphatase 1
6625.	GAS6	Growth Arrest Specific 6
6626.	SLC22A2	Solute Carrier Family 22 Member 2
6627.	IGFBP5	Insulin Like Growth Factor Binding Protein 5
6628.	LRPPRC	Leucine Rich Pentatricopeptide Repeat Containing
6629.	STIP1	Stress Induced Phosphoprotein 1
6630.	LGALS3BP	Galectin 3 Binding Protein
6631.	TRAF4	TNF Receptor Associated Factor 4
6632.	ZMYM2	Zinc Finger MYM-Type Containing 2
6633.	ACSL5	Acyl-CoA Synthetase Long Chain Family Member 5
6634.	CD160	CD160 Molecule
6635.	CSRP1	Cysteine And Glycine Rich Protein 1
6636.	DIO2	Iodothyronine Deiodinase 2
6637.	MTNR1A	Melatonin Receptor 1A
6638.	NISCH	Nischarin
6639.	RARRES2	Retinoic Acid Receptor Responder 2
6640.	RIN2	Ras And Rab Interactor 2
6641.	CA10	Carbonic Anhydrase 10
6642.	CEP43	Centrosomal Protein 43
6643.	TNFSF12	TNF Superfamily Member 12
6644.	BCAS3	BCAS3 Microtubule Associated Cell Migration Factor
6645.	LCN1	Lipocalin 1
6646.	SLC13A1	Solute Carrier Family 13 Member 1
6647.	RBM3	RNA Binding Motif Protein 3
6648.	TAAR8	Trace Amine Associated Receptor 8
6649.	TRG	T Cell Receptor Gamma Locus
6650.	NCOR2	Nuclear Receptor Corepressor 2

Supplementary Table S3. Genes associated with myalgia that are extracted from GeneCards.

Index	Gene Symbol	Gene Full Name
1.	MLIP	Muscular LMNA Interacting Protein
2.	RYR1	Ryanodine Receptor 1

3.	TNFRSF1A	TNF Receptor Superfamily Member 1A
4.	NLRP3	NLR Family Pyrin Domain Containing 3
5.	MEFV	MEFV Innate Immunity Regulator, Pyrin
6.	DMD	Dystrophin
7.	CPT2	Carnitine Palmitoyltransferase 2
8.	IL6	Interleukin 6
9.	NLRC4	NLR Family CARD Domain Containing 4
10.	CAPN3	Calpain 3
11.	AMPD1	Adenosine Monophosphate Deaminase 1
12.	NLRP12	NLR Family Pyrin Domain Containing 12
13.	IFNG	Interferon Gamma
14.	TNF	Tumor Necrosis Factor
15.	CAV3	Caveolin 3
16.	ANO5	Anoctamin 5
17.	DYSF	Dysferlin
18.	HLA-DRB1	Major Histocompatibility Complex, Class II, DR Beta 1
19.	ADA2	Adenosine Deaminase 2
20.	OBSCN	Obscurin, Cytoskeletal Calmodulin And Titin-Interacting RhoGEF
21.	FKRP	Fukutin Related Protein
22.	CCDC78	Coiled-Coil Domain Containing 78
23.	ENO3	Enolase 3
24.	IL5	Interleukin 5
25.	SDHA	Succinate Dehydrogenase Complex Flavoprotein Subunit A
26.	IL10	Interleukin 10
27.	MT-CO1	Mitochondrially Encoded Cytochrome C Oxidase I
28.	TGFB1	Transforming Growth Factor Beta 1
29.	AMPD3	Adenosine Monophosphate Deaminase 3
30.	LAMA2	Laminin Subunit Alpha 2
31.	CASQ1	Calsequestrin 1
32.	PGAM2	Phosphoglycerate Mutase 2
33.	HLA-B	Major Histocompatibility Complex, Class I, B
34.	FDX2	Ferredoxin 2
35.	MVK	Mevalonate Kinase
36.	SCN4A	Sodium Voltage-Gated Channel Alpha Subunit 4
37.	ATP2A1	ATPase Sarcoplasmic/Endoplasmic Reticulum Ca2+ Transporting 1
38.	CKM	Creatine Kinase, M-Type
39.	PYGM	Glycogen Phosphorylase, Muscle Associated
40.	CXCL8	C-X-C Motif Chemokine Ligand 8
41.	IL1B	Interleukin 1 Beta
42.	TMEM126B	Transmembrane Protein 126B
43.	HLA-DQA1	Major Histocompatibility Complex, Class II, DQ Alpha 1

44.	MYOT	Myotilin
45.	CRP	C-Reactive Protein
46.	STIM1	Stromal Interaction Molecule 1
47.	FLNC	Filamin C
48.	BMP6	Bone Morphogenetic Protein 6
49.	IDO1	Indoleamine 2,3-Dioxygenase 1
50.	ICAM1	Intercellular Adhesion Molecule 1
51.	CRH	Corticotropin Releasing Hormone
52.	POLG	DNA Polymerase Gamma, Catalytic Subunit
53.	IL18	Interleukin 18
54.	ACAD9	Acyl-CoA Dehydrogenase Family Member 9
55.	MT-CYB	Mitochondrially Encoded Cytochrome B
56.	ACADVL	Acyl-CoA Dehydrogenase Very Long Chain
57.	HADHA	Hydroxyacyl-CoA Dehydrogenase Trifunctional Multienzyme Complex Subunit Alpha
58.	LPIN2	Lipin 2
59.	HNMT	Histamine N-Methyltransferase
60.	HADHB	Hydroxyacyl-CoA Dehydrogenase Trifunctional Multienzyme Complex Subunit Beta
61.	LMNA	Lamin A/C
62.	CKMT2	Creatine Kinase, Mitochondrial 2
63.	CHKB	Choline Kinase Beta
64.	SLC12A3	Solute Carrier Family 12 Member 3
65.	CKB	Creatine Kinase B
66.	CKMT1B	Creatine Kinase, Mitochondrial 1B
67.	CD4	CD4 Molecule
68.	MT-ND2	Mitochondrially Encoded NADH:Ubiquinone Oxidoreductase Core Subunit 2
69.	IFNA1	Interferon Alpha 1
70.	OXTR	Oxytocin Receptor
71.	TRAPPC11	Trafficking Protein Particle Complex Subunit 11
72.	MT-CO3	Mitochondrially Encoded Cytochrome C Oxidase III
73.	CBS	Cystathionine Beta-Synthase
74.	AHR	Aryl Hydrocarbon Receptor
75.	SLCO1B1	Solute Carrier Organic Anion Transporter Family Member 1B1
76.	ORAI1	ORAI Calcium Release-Activated Calcium Modulator 1
77.	G6PD	Glucose-6-Phosphate Dehydrogenase
78.	IL1A	Interleukin 1 Alpha
79.	CPN1	Carboxypeptidase N Subunit 1
80.	TNXB	Tenascin XB
81.	LPIN1	Lipin 1
82.	SLC4A1	Solute Carrier Family 4 Member 1 (Diego Blood Group)
83.	NOD2	Nucleotide Binding Oligomerization Domain Containing 2

84.	CD36	CD36 Molecule (CD36 Blood Group)
85.	SELENON	Selenoprotein N
86.	MTMR14	Myotubularin Related Protein 14
87.	PRTN3	Proteinase 3
88.	CD8A	CD8 Subunit Alpha
89.	BIN1	Bridging Integrator 1
90.	PFKM	Phosphofructokinase, Muscle
91.	LDHA	Lactate Dehydrogenase A
92.	MIF	Macrophage Migration Inhibitory Factor
93.	GPT	Glutamic--Pyruvic Transaminase
94.	IL17A	Interleukin 17A
95.	SARDH	Sarcosine Dehydrogenase
96.	FCGR2A	Fc Gamma Receptor IIa
97.	ERVW-1	Endogenous Retrovirus Group W Member 1, Envelope
98.	MT-ND4	Mitochondrially Encoded NADH:Ubiquinone Oxidoreductase Core Subunit 4
99.	CSF3	Colony Stimulating Factor 3
100.	HBB	Hemoglobin Subunit Beta
101.	GYPC	Glycophorin C (Gerbich Blood Group)
102.	KY	Kyphoscoliosis Peptidase
103.	CCL2	C-C Motif Chemokine Ligand 2
104.	TTN	Titin
105.	NDUFS1	NADH:Ubiquinone Oxidoreductase Core Subunit S1
106.	CLCN1	Chloride Voltage-Gated Channel 1
107.	IFNB1	Interferon Beta 1
108.	KBTBD13	Kelch Repeat And BTB Domain Containing 13
109.	C4A	Complement C4A (Chido/Rodgers Blood Group)
110.	DNASE1L3	Deoxyribonuclease 1 Like 3
111.	HPDL	4-Hydroxyphenylpyruvate Dioxygenase Like
112.	IL2	Interleukin 2
113.	PIK3C2A	Phosphatidylinositol-4-Phosphate 3-Kinase Catalytic Subunit Type 2 Alpha
114.	MYH7	Myosin Heavy Chain 7
115.	ACADM	Acyl-CoA Dehydrogenase Medium Chain
116.	ALB	Albumin
117.	MT-ATP6	Mitochondrially Encoded ATP Synthase Membrane Subunit 6
118.	MT-ND1	Mitochondrially Encoded NADH:Ubiquinone Oxidoreductase Core Subunit 1
119.	CXCL10	C-X-C Motif Chemokine Ligand 10
120.	ASPH	Aspartate Beta-Hydroxylase
121.	TRIM32	Tripartite Motif Containing 32
122.	RRM2B	Ribonucleotide Reductase Regulatory TP53 Inducible Subunit M2B
123.	DES	Desmin
124.	PSTPIP1	Proline-Serine-Threonine Phosphatase Interacting Protein 1

125.	DBNL	Drebrin Like
126.	MT-ND5	Mitochondrially Encoded NADH:Ubiquinone Oxidoreductase Core Subunit 5
127.	MT-ND6	Mitochondrially Encoded NADH:Ubiquinone Oxidoreductase Core Subunit 6
128.	IL1R1	Interleukin 1 Receptor Type 1
129.	PPARG	Peroxisome Proliferator Activated Receptor Gamma
130.	IL4	Interleukin 4
131.	IKBKG	Inhibitor Of Nuclear Factor Kappa B Kinase Regulatory Subunit Gamma
132.	CTLA4	Cytotoxic T-Lymphocyte Associated Protein 4
133.	DNM2	Dynamin 2
134.	CRPPA	CDP-L-Ribitol Pyrophosphorylase A
135.	TLR4	Toll Like Receptor 4
136.	NDUFS4	NADH:Ubiquinone Oxidoreductase Subunit S4
137.	TWNK	Twinkle MtDNA Helicase
138.	RIGI	RNA Sensor RIG-I
139.	FKTN	Fukutin
140.	IL2RA	Interleukin 2 Receptor Subunit Alpha
141.	LDB3	LIM Domain Binding 3
142.	CACNA1S	Calcium Voltage-Gated Channel Subunit Alpha1 S
143.	NDUFV1	NADH:Ubiquinone Oxidoreductase Core Subunit V1
144.	NDUFAF1	NADH:Ubiquinone Oxidoreductase Complex Assembly Factor 1
145.	MT-ND3	Mitochondrially Encoded NADH:Ubiquinone Oxidoreductase Core Subunit 3
146.	FCGR2B	Fc Gamma Receptor IIb
147.	GYPA	Glycophorin A (MNS Blood Group)
148.	GYPB	Glycophorin B (MNS Blood Group)
149.	IL1RN	Interleukin 1 Receptor Antagonist
150.	ACE2	Angiotensin Converting Enzyme 2
151.	HSPD1	Heat Shock Protein Family D (Hsp60) Member 1
152.	F3	Coagulation Factor III, Tissue Factor
153.	NOS2	Nitric Oxide Synthase 2
154.	CR1	Complement C3b/C4b Receptor 1 (Knops Blood Group)
155.	MT-CO2	Mitochondrially Encoded Cytochrome C Oxidase II
156.	TRDN	Triadin
157.	SLC2A1	Solute Carrier Family 2 Member 1
158.	OTULIN	OTU Deubiquitinase With Linear Linkage Specificity
159.	UBA1	Ubiquitin Like Modifier Activating Enzyme 1
160.	SGCA	Sarcoglycan Alpha
161.	POMT1	Protein O-Mannosyltransferase 1
162.	CASP1	Caspase 1
163.	IFIH1	Interferon Induced With Helicase C Domain 1
164.	PRG2	Proteoglycan 2, Pro Eosinophil Major Basic Protein
165.	PHKA1	Phosphorylase Kinase Regulatory Subunit Alpha 1

166.	GYG1	Glycogenin 1
167.	ETFDH	Electron Transfer Flavoprotein Dehydrogenase
168.	POLGARF	POLG Alternative Reading Frame
169.	NDUFS2	NADH:Ubiquinone Oxidoreductase Core Subunit S2
170.	NDUFV2	NADH:Ubiquinone Oxidoreductase Core Subunit V2
171.	TIMMDC1	Translocase Of Inner Mitochondrial Membrane Domain Containing 1
172.	ANK1	Ankyrin 1
173.	F2	Coagulation Factor II, Thrombin
174.	MATR3	Matrin 3
175.	POLG2	DNA Polymerase Gamma 2, Accessory Subunit
176.	SLC2A2	Solute Carrier Family 2 Member 2
177.	DAG1	Dystroglycan 1
178.	IDO2	Indoleamine 2,3-Dioxygenase 2
179.	IFNGR1	Interferon Gamma Receptor 1
180.	FURIN	Furin, Paired Basic Amino Acid Cleaving Enzyme
181.	STRN4	Striatin 4
182.	MYF6	Myogenic Factor 6
183.	DGUOK	Deoxyguanosine Kinase
184.	RYR2	Ryanodine Receptor 2
185.	ACE	Angiotensin I Converting Enzyme
186.	MPO	Myeloperoxidase
187.	FMR1	Fragile X Messenger Ribonucleoprotein 1
188.	PTGS2	Prostaglandin-Endoperoxide Synthase 2
189.	MB	Myoglobin
190.	GYS1	Glycogen Synthase 1
191.	KIT	KIT Proto-Oncogene, Receptor Tyrosine Kinase
192.	HLA-DPB1	Major Histocompatibility Complex, Class II, DP Beta 1
193.	CNBP	CCHC-Type Zinc Finger Nucleic Acid Binding Protein
194.	SCN9A	Sodium Voltage-Gated Channel Alpha Subunit 9
195.	MAPKAPK3	MAPK Activated Protein Kinase 3
196.	CISH	Cytokine Inducible SH2 Containing Protein
197.	ACKR1	Atypical Chemokine Receptor 1 (Duffy Blood Group)
198.	TIRAP	TIR Domain Containing Adaptor Protein
199.	CFAP92	Cilia And Flagella Associated Protein 92 (Putative)
200.	CSF2	Colony Stimulating Factor 2
201.	MEGF10	Multiple EGF Like Domains 10
202.	TNPO3	Transportin 3
203.	PDGFRA	Platelet Derived Growth Factor Receptor Alpha
204.	CAVIN1	Caveolae Associated Protein 1
205.	CCL3	C-C Motif Chemokine Ligand 3
206.	CTNNB1	Catenin Beta 1

207.	FAS	Fas Cell Surface Death Receptor
208.	DNAJB6	DnaJ Heat Shock Protein Family (Hsp40) Member B6
209.	INS	Insulin
210.	GAA	Alpha Glucosidase
211.	RNASEH1	Ribonuclease H1
212.	NDUFAF5	NADH:Ubiquinone Oxidoreductase Complex Assembly Factor 5
213.	VCP	Valosin Containing Protein
214.	IL12B	Interleukin 12B
215.	IL2RB	Interleukin 2 Receptor Subunit Beta
216.	TLR3	Toll Like Receptor 3
217.	TCAP	Titin-Cap
218.	IFNA2	Interferon Alpha 2
219.	ACADL	Acyl-CoA Dehydrogenase Long Chain
220.	ETFB	Electron Transfer Flavoprotein Subunit Beta
221.	PTPN22	Protein Tyrosine Phosphatase Non-Receptor Type 22
222.	TK2	Thymidine Kinase 2
223.	ETFA	Electron Transfer Flavoprotein Subunit Alpha
224.	CHCHD10	Coiled-Coil-Helix-Coiled-Coil-Helix Domain Containing 10
225.	ERVFRD-1	Endogenous Retrovirus Group FRD Member 1, Envelope
226.	ACTA1	Actin Alpha 1, Skeletal Muscle
227.	HNRNPA1	Heterogeneous Nuclear Ribonucleoprotein A1
228.	NDUFS3	NADH:Ubiquinone Oxidoreductase Core Subunit S3
229.	CFD	Complement Factor D
230.	NDUFA13	NADH:Ubiquinone Oxidoreductase Subunit A13
231.	NDUFB9	NADH:Ubiquinone Oxidoreductase Subunit B9
232.	NDUFA1	NADH:Ubiquinone Oxidoreductase Subunit A1
233.	NDUFB10	NADH:Ubiquinone Oxidoreductase Subunit B10
234.	NDUFS6	NADH:Ubiquinone Oxidoreductase Subunit S6
235.	NDUFAF4	NADH:Ubiquinone Oxidoreductase Complex Assembly Factor 4
236.	NDUFAF2	NADH:Ubiquinone Oxidoreductase Complex Assembly Factor 2
237.	NDUFB3	NADH:Ubiquinone Oxidoreductase Subunit B3
238.	FOXRED1	FAD Dependent Oxidoreductase Domain Containing 1
239.	NDUFB11	NADH:Ubiquinone Oxidoreductase Subunit B11
240.	NUBPL	NUBP Iron-Sulfur Cluster Assembly Factor, Mitochondrial
241.	NDUFAF3	NADH:Ubiquinone Oxidoreductase Complex Assembly Factor 3
242.	NDUFA11	NADH:Ubiquinone Oxidoreductase Subunit A11
243.	TLR2	Toll Like Receptor 2
244.	IFNAR1	Interferon Alpha And Beta Receptor Subunit 1
245.	MYOM2	Myomesin 2
246.	SLC25A4	Solute Carrier Family 25 Member 4
247.	DNA2	DNA Replication Helicase/Nuclease 2

248.	SPTA1	Spectrin Alpha, Erythrocytic 1
249.	ERV3-1	Endogenous Retrovirus Group 3 Member 1, Envelope
250.	IL1RL2	Interleukin 1 Receptor Like 2
251.	POMC	Proopiomelanocortin
252.	TLR7	Toll Like Receptor 7
253.	SGCB	Sarcoglycan Beta
254.	SGCG	Sarcoglycan Gamma
255.	PNPLA2	Patatin Like Phospholipase Domain Containing 2
256.	IRF3	Interferon Regulatory Factor 3
257.	DNM1L	Dynamin 1 Like
258.	GK	Glycerol Kinase
259.	PHKA2	Phosphorylase Kinase Regulatory Subunit Alpha 2
260.	GMPPB	GDP-Mannose Pyrophosphorylase B
261.	CR2	Complement C3d Receptor 2
262.	IL36RN	Interleukin 36 Receptor Antagonist
263.	MTTP	Microsomal Triglyceride Transfer Protein
264.	MAPK1	Mitogen-Activated Protein Kinase 1
265.	RBCK1	RANBP2-Type And C3HC4-Type Zinc Finger Containing 1
266.	CYP3A4	Cytochrome P450 Family 3 Subfamily A Member 4
267.	PHKG2	Phosphorylase Kinase Catalytic Subunit Gamma 2
268.	VCAM1	Vascular Cell Adhesion Molecule 1
269.	MBTPS1	Membrane Bound Transcription Factor Peptidase, Site 1
270.	CCL5	C-C Motif Chemokine Ligand 5
271.	CD40LG	CD40 Ligand
272.	COQ2	Coenzyme Q2, Polyprenyltransferase
273.	PRKCD	Protein Kinase C Delta
274.	C1R	Complement C1r
275.	C1QA	Complement C1q A Chain
276.	DNASE1	Deoxyribonuclease 1
277.	LAMA5	Laminin Subunit Alpha 5
278.	ERCC8	ERCC Excision Repair 8, CSA Ubiquitin Ligase Complex Subunit
279.	SLC25A10	Solute Carrier Family 25 Member 10
280.	IL3	Interleukin 3
281.	MSTO1	Misato Mitochondrial Distribution And Morphology Regulator 1
282.	CS	Citrate Synthase
283.	GTPBP1	GTP Binding Protein 1
284.	GP2	Glycoprotein 2
285.	SH2D3A	SH2 Domain Containing 3A
286.	COX6B1	Cytochrome C Oxidase Subunit 6B1
287.	SVIL	Supervillin
288.	BDNF	Brain Derived Neurotrophic Factor

289.	HSPG2	Heparan Sulfate Proteoglycan 2
290.	LMNB1	Lamin B1
291.	CMC4	C-X9-C Motif Containing 4
292.	AGL	Amylo-Alpha-1, 6-Glucosidase, 4-Alpha-Glucanotransferase
293.	SGCD	Sarcoglycan Delta
294.	HP	Haptoglobin
295.	PGK1	Phosphoglycerate Kinase 1
296.	PLEKHG4	Pleckstrin Homology And RhoGEF Domain Containing G4
297.	MTM1	Myotubularin 1
298.	CARD14	Caspase Recruitment Domain Family Member 14
299.	HMGCL	3-Hydroxy-3-Methylglutaryl-CoA Lyase
300.	AFF2	ALF Transcription Elongation Factor 2
301.	HMGCR	3-Hydroxy-3-Methylglutaryl-CoA Reductase
302.	ACADS	Acyl-CoA Dehydrogenase Short Chain
303.	MIEF2	Mitochondrial Elongation Factor 2
304.	MYH2	Myosin Heavy Chain 2
305.	ANK2	Ankyrin 2
306.	CLEC4M	C-Type Lectin Domain Family 4 Member M
307.	LBR	Lamin B Receptor
308.	OPA1	OPA1 Mitochondrial Dynamin Like GTPase
309.	PHKB	Phosphorylase Kinase Regulatory Subunit Beta
310.	SPTB	Spectrin Beta, Erythrocytic
311.	SH3TC2	SH3 Domain And Tetratricopeptide Repeats 2
312.	ZNF469	Zinc Finger Protein 469
313.	TFRC	Transferrin Receptor
314.	TRIM21	Tripartite Motif Containing 21
315.	TLR5	Toll Like Receptor 5
316.	IL33	Interleukin 33
317.	SH2D3C	SH2 Domain Containing 3C
318.	SELP	Selectin P
319.	AGTR1	Angiotensin II Receptor Type 1
320.	CD68	CD68 Molecule
321.	ACTN3	Actinin Alpha 3
322.	TNNT3	Troponin T3, Fast Skeletal Type
323.	THBD	Thrombomodulin
324.	RHOF	Ras Homolog Family Member F, Filopodia Associated
325.	HLA-DQB1	Major Histocompatibility Complex, Class II, DQ Beta 1
326.	CPT1A	Carnitine Palmitoyltransferase 1A
327.	MYBPC1	Myosin Binding Protein C1
328.	FLAD1	Flavin Adenine Dinucleotide Synthetase 1
329.	SELE	Selectin E

330.	BSCL2	BSCL2 Lipid Droplet Biogenesis Associated, Seipin
331.	CD209	CD209 Molecule
332.	AGT	Angiotensinogen
333.	COMT	Catechol-O-Methyltransferase
334.	ALDOA	Aldolase, Fructose-Bisphosphate A
335.	GLA	Galactosidase Alpha
336.	ACP5	Acid Phosphatase 5, Tartrate Resistant
337.	ERAP1	Endoplasmic Reticulum Aminopeptidase 1
338.	EXT2	Exostosin Glycosyltransferase 2
339.	BVES	Blood Vessel Epicardial Substance
340.	HADH	Hydroxyacyl-CoA Dehydrogenase
341.	HTRA1	HtrA Serine Peptidase 1
342.	IL13	Interleukin 13
343.	SLC22A5	Solute Carrier Family 22 Member 5
344.	SAA1	Serum Amyloid A1
345.	GNE	Glucosamine (UDP-N-Acetyl)-2-Epimerase/N-Acetylmannosamine Kinase
346.	STAT4	Signal Transducer And Activator Of Transcription 4
347.	PRDM5	PR/SET Domain 5
348.	SLC25A20	Solute Carrier Family 25 Member 20
349.	SPTBN2	Spectrin Beta, Non-Erythrocytic 2
350.	MAPRE3	Microtubule Associated Protein RP/EB Family Member 3
351.	TPM2	Tropomyosin 2
352.	MYH14	Myosin Heavy Chain 14
353.	CYTH1	Cytohesin 1
354.	NEB	Nebulin
355.	TNNI2	Troponin I2, Fast Skeletal Type
356.	ABCB1	ATP Binding Cassette Subfamily B Member 1
357.	SLC25A42	Solute Carrier Family 25 Member 42
358.	TLR1	Toll Like Receptor 1
359.	TMPRSS2	Transmembrane Serine Protease 2
360.	TLR9	Toll Like Receptor 9
361.	LTA	Lymphotoxin Alpha
362.	KNG1	Kininogen 1
363.	AGPAT2	1-Acylglycerol-3-Phosphate O-Acyltransferase 2
364.	CYP3A5	Cytochrome P450 Family 3 Subfamily A Member 5
365.	FCGR3A	Fc Gamma Receptor IIIa
366.	HLA-A	Major Histocompatibility Complex, Class I, A
367.	FCGR3B	Fc Gamma Receptor IIIb
368.	GARS1	Glycyl-TRNA Synthetase 1
369.	STAT1	Signal Transducer And Activator Of Transcription 1
370.	NLRP1	NLR Family Pyrin Domain Containing 1

371.	DPM3	Dolichyl-Phosphate Mannosyltransferase Subunit 3, Regulatory
372.	CCL11	C-C Motif Chemokine Ligand 11
373.	ADA	Adenosine Deaminase
374.	TNFAIP3	TNF Alpha Induced Protein 3
375.	IGF1	Insulin Like Growth Factor 1
376.	SOD1	Superoxide Dismutase 1
377.	DHFR	Dihydrofolate Reductase
378.	SLC36A2	Solute Carrier Family 36 Member 2
379.	P4HA2	Prolyl 4-Hydroxylase Subunit Alpha 2
380.	FBN1	Fibrillin 1
381.	STING1	Stimulator Of Interferon Response CGAMP Interactor 1
382.	APOE	Apolipoprotein E
383.	FASLG	Fas Ligand
384.	SCN11A	Sodium Voltage-Gated Channel Alpha Subunit 11
385.	CCR5	C-C Motif Chemokine Receptor 5
386.	LTF	Lactotransferrin
387.	MAP2K3	Mitogen-Activated Protein Kinase Kinase 3
388.	CACNG2	Calcium Voltage-Gated Channel Auxiliary Subunit Gamma 2
389.	GADD45G	Growth Arrest And DNA Damage Inducible Gamma
390.	RNASE3	Ribonuclease A Family Member 3
391.	MBL2	Mannose Binding Lectin 2
392.	TP53	Tumor Protein P53
393.	HARS1	Histidyl-TRNA Synthetase 1
394.	CD28	CD28 Molecule
395.	CYP2D6	Cytochrome P450 Family 2 Subfamily D Member 6
396.	ACAD8	Acyl-CoA Dehydrogenase Family Member 8
397.	PPP1CA	Protein Phosphatase 1 Catalytic Subunit Alpha
398.	PRSS57	Serine Protease 57
399.	BST2	Bone Marrow Stromal Cell Antigen 2
400.	MYOD1	Myogenic Differentiation 1
401.	REN	Renin
402.	SCN8A	Sodium Voltage-Gated Channel Alpha Subunit 8
403.	MMP9	Matrix Metallopeptidase 9
404.	FHL1	Four And A Half LIM Domains 1
405.	MORC3	MORC Family CW-Type Zinc Finger 3
406.	MMP3	Matrix Metallopeptidase 3
407.	TMBIM4	Transmembrane BAX Inhibitor Motif Containing 4
408.	GAPDH	Glyceraldehyde-3-Phosphate Dehydrogenase
409.	MFN2	Mitofusin 2
410.	IL1RAP	Interleukin 1 Receptor Accessory Protein
411.	TNFRSF10A	TNF Receptor Superfamily Member 10a

412.	STAC3	SH3 And Cysteine Rich Domain 3
413.	LGALS3	Galectin 3
414.	SAA2	Serum Amyloid A2
415.	IGHD	Immunoglobulin Heavy Constant Delta
416.	IRF7	Interferon Regulatory Factor 7
417.	SFTPD	Surfactant Protein D
418.	KCNJ18	Potassium Inwardly Rectifying Channel Subfamily J Member 18
419.	CAV1	Caveolin 1
420.	FXN	Frataxin
421.	RAD51	RAD51 Recombinase
422.	APC	APC Regulator Of WNT Signaling Pathway
423.	CD247	CD247 Molecule
424.	DCC	DCC Netrin 1 Receptor
425.	EXT1	Exostosin Glycosyltransferase 1
426.	PTPN2	Protein Tyrosine Phosphatase Non-Receptor Type 2
427.	NTN1	Netrin 1
428.	TET2	Tet Methylcytosine Dioxygenase 2
429.	ALDH4A1	Aldehyde Dehydrogenase 4 Family Member A1
430.	ASXL1	ASXL Transcriptional Regulator 1
431.	IL23R	Interleukin 23 Receptor
432.	POLR3A	RNA Polymerase III Subunit A
433.	CCR1	C-C Motif Chemokine Receptor 1
434.	GP1BB	Glycoprotein Ib Platelet Subunit Beta
435.	IL12A	Interleukin 12A
436.	IRF4	Interferon Regulatory Factor 4
437.	TBX1	T-Box Transcription Factor 1
438.	CLCNKB	Chloride Voltage-Gated Channel Kb
439.	HIRA	Histone Cell Cycle Regulator
440.	SEC24C	SEC24 Homolog C, COPII Coat Complex Component
441.	UFD1	Ubiquitin Recognition Factor In ER Associated Degradation 1
442.	JMJD1C	Jumonji Domain Containing 1C
443.	MLX	MAX Dimerization Protein MLX
444.	ALMS1	ALMS1 Centrosome And Basal Body Associated Protein
445.	HLA-DPA1	Major Histocompatibility Complex, Class II, DP Alpha 1
446.	EPB42	Erythrocyte Membrane Protein Band 4.2
447.	RREB1	Ras Responsive Element Binding Protein 1
448.	SRSF2	Serine And Arginine Rich Splicing Factor 2
449.	ARVCF	ARVCF Delta Catenin Family Member
450.	DNAL4	Dynein Axonemal Light Chain 4
451.	VWA1	Von Willebrand Factor A Domain Containing 1
452.	PRORP	Protein Only RNase P Catalytic Subunit

453.	UBAC2	UBA Domain Containing 2
454.	KLRC4	Killer Cell Lectin Like Receptor C4
455.	ANKRD55	Ankyrin Repeat Domain 55
456.	IGKC	Immunoglobulin Kappa Constant
457.	IGHG2	Immunoglobulin Heavy Constant Gamma 2 (G2m Marker)
458.	PECAM1	Platelet And Endothelial Cell Adhesion Molecule 1
459.	PPBP	Pro-Platelet Basic Protein
460.	CFTR	CF Transmembrane Conductance Regulator
461.	SERPINA1	Serpin Family A Member 1
462.	PYROXD1	Pyridine Nucleotide-Disulphide Oxidoreductase Domain 1
463.	PDCD1	Programmed Cell Death 1
464.	TNFSF13B	TNF Superfamily Member 13b
465.	POMGNT1	Protein O-Linked Mannose N-Acetylglucosaminyltransferase 1 (Beta 1,2-)
466.	POMT2	Protein O-Mannosyltransferase 2
467.	SSUH2	Ssu-2 Homolog
468.	XDH	Xanthine Dehydrogenase
469.	IL7	Interleukin 7
470.	PPP1R14A	Protein Phosphatase 1 Regulatory Inhibitor Subunit 14A
471.	PLEC	Plectin
472.	APOA1	Apolipoprotein A1
473.	MT-ATP8	Mitochondrially Encoded ATP Synthase Membrane Subunit 8
474.	SLC6A4	Solute Carrier Family 6 Member 4
475.	COX5A	Cytochrome C Oxidase Subunit 5A
476.	DMPK	DM1 Protein Kinase
477.	NPC1	NPC Intracellular Cholesterol Transporter 1
478.	PLAT	Plasminogen Activator, Tissue Type
479.	TPI1	Triosephosphate Isomerase 1
480.	LCN2	Lipocalin 2
481.	HAVCR1	Hepatitis A Virus Cellular Receptor 1
482.	ITIH4	Inter-Alpha-Trypsin Inhibitor Heavy Chain 4
483.	NLRP7	NLR Family Pyrin Domain Containing 7
484.	ANKRD46	Ankyrin Repeat Domain 46
485.	CD19	CD19 Molecule
486.	EEF2	Eukaryotic Translation Elongation Factor 2
487.	VWF	Von Willebrand Factor
488.	CD86	CD86 Molecule
489.	CXCR3	C-X-C Motif Chemokine Receptor 3
490.	CD80	CD80 Molecule
491.	LAMP1	Lysosomal Associated Membrane Protein 1
492.	PYCARD	PYD And CARD Domain Containing
493.	AIM2	Absent In Melanoma 2

494.	RHD	Rh Blood Group D Antigen
495.	SCN5A	Sodium Voltage-Gated Channel Alpha Subunit 5
496.	IL1RAPL2	Interleukin 1 Receptor Accessory Protein Like 2
497.	DNAH8	Dynein Axonemal Heavy Chain 8
498.	POMGNT2	Protein O-Linked Mannose N-Acetylglucosaminyltransferase 2 (Beta 1,4-)
499.	POMK	Protein O-Mannose Kinase
500.	RXYLT1	Ribitol Xylosyltransferase 1
501.	NOS3	Nitric Oxide Synthase 3
502.	SERPINE1	Serpin Family E Member 1
503.	EDN1	Endothelin 1
504.	TNFRSF1B	TNF Receptor Superfamily Member 1B
505.	PKLR	Pyruvate Kinase L/R
506.	ABCC8	ATP Binding Cassette Subfamily C Member 8
507.	CYP2B6	Cytochrome P450 Family 2 Subfamily B Member 6
508.	CYP2C19	Cytochrome P450 Family 2 Subfamily C Member 19
509.	PRKG1	Protein Kinase CGMP-Dependent 1
510.	CYP2C8	Cytochrome P450 Family 2 Subfamily C Member 8
511.	LARS2	Leucyl-TRNA Synthetase 2, Mitochondrial
512.	C1QBP	Complement C1q Binding Protein
513.	AKT2	AKT Serine/Threonine Kinase 2
514.	INSR	Insulin Receptor
515.	PLIN1	Perilipin 1
516.	APOH	Apolipoprotein H
517.	GPLD1	Glycosylphosphatidylinositol Specific Phospholipase D1
518.	SCN2A	Sodium Voltage-Gated Channel Alpha Subunit 2
519.	MMP1	Matrix Metallopeptidase 1
520.	TARDBP	TAR DNA Binding Protein
521.	PRF1	Perforin 1
522.	UTRN	Utrophin
523.	CXCL9	C-X-C Motif Chemokine Ligand 9
524.	EXOSC10	Exosome Component 10
525.	FIP1L1	Factor Interacting With PAPOLA And CPSF1
526.	COL1A1	Collagen Type I Alpha 1 Chain
527.	ELANE	Elastase, Neutrophil Expressed
528.	FOXP3	Forkhead Box P3
529.	DHODH	Dihydroorotate Dehydrogenase (Quinone)
530.	CCR7	C-C Motif Chemokine Receptor 7
531.	CHIT1	Chitinase 1
532.	HLA-C	Major Histocompatibility Complex, Class I, C
533.	TCN2	Transcobalamin 2
534.	CAV2	Caveolin 2

535.	CYP51A1	Cytochrome P450 Family 51 Subfamily A Member 1
536.	SSB	Small RNA Binding Exonuclease Protection Factor La
537.	SENP8	SUMO Peptidase Family Member, NEDD8 Specific
538.	KLHL40	Kelch Like Family Member 40
539.	MYD88	MYD88 Innate Immune Signal Transduction Adaptor
540.	HSPA4	Heat Shock Protein Family A (Hsp70) Member 4
541.	ATP12A	ATPase H+/K+ Transporting Non-Gastric Alpha2 Subunit
542.	UNC13D	Unc-13 Homolog D
543.	KCNJ2	Potassium Inwardly Rectifying Channel Subfamily J Member 2
544.	MSTN	Myostatin
545.	MDM4	MDM4 Regulator Of P53
546.	MOCOS	Molybdenum Cofactor Sulfurase
547.	DSE	Dermatan Sulfate Epimerase
548.	MRPS2	Mitochondrial Ribosomal Protein S2
549.	ADRB2	Adrenoceptor Beta 2
550.	ACHE	Acetylcholinesterase (Yt Blood Group)
551.	B2M	Beta-2-Microglobulin
552.	C3	Complement C3
553.	GZMB	Granzyme B
554.	MYBPC3	Myosin Binding Protein C3
555.	HSP90AA1	Heat Shock Protein 90 Alpha Family Class A Member 1
556.	LPIN3	Lipin 3
557.	MYH3	Myosin Heavy Chain 3
558.	HNRNPH2	Heterogeneous Nuclear Ribonucleoprotein H2
559.	TBP	TATA-Box Binding Protein
560.	THBS1	Thrombospondin 1
561.	CPOX	Coproporphyrinogen Oxidase
562.	DYNLT1	Dynein Light Chain Tctex-Type 1
563.	ICMT	Isoprenylcysteine Carboxyl Methyltransferase
564.	NEK7	NIMA Related Kinase 7
565.	GSDMD	Gasdermin D
566.	NLRP6	NLR Family Pyrin Domain Containing 6
567.	PSTPIP2	Proline-Serine-Threonine Phosphatase Interacting Protein 2
568.	CHKA	Choline Kinase Alpha
569.	SFTPC	Surfactant Protein C
570.	EARS2	Glutamyl-TRNA Synthetase 2, Mitochondrial
571.	SYK	Spleen Associated Tyrosine Kinase
572.	CTSL	Cathepsin L
573.	XRCC6	X-Ray Repair Cross Complementing 6
574.	DHPS	Deoxyhypusine Synthase
575.	WDR1	WD Repeat Domain 1

576.	PPARA	Peroxisome Proliferator Activated Receptor Alpha
577.	CASP3	Caspase 3
578.	ALAD	Aminolevulinate Dehydratase
579.	MTO1	Mitochondrial TRNA Translation Optimization 1
580.	NLRP5	NLR Family Pyrin Domain Containing 5
581.	NLRP13	NLR Family Pyrin Domain Containing 13
582.	CACNA1A	Calcium Voltage-Gated Channel Subunit Alpha1 A
583.	GBE1	1,4-Alpha-Glucan Branching Enzyme 1
584.	KCNE3	Potassium Voltage-Gated Channel Subfamily E Regulatory Subunit 3
585.	CHRND	Cholinergic Receptor Nicotinic Delta Subunit
586.	ADSS1	Adenylosuccinate Synthase 1
587.	MAPT	Microtubule Associated Protein Tau
588.	MYL2	Myosin Light Chain 2
589.	TTR	Transthyretin
590.	CCR3	C-C Motif Chemokine Receptor 3
591.	PTX3	Pentraxin 3
592.	TARS1	Threonyl-TRNA Synthetase 1
593.	MYPN	Myopalladin
594.	RPLP0	Ribosomal Protein Lateral Stalk Subunit P0
595.	SSBP1	Single Stranded DNA Binding Protein 1
596.	STIM2	Stromal Interaction Molecule 2
597.	SLC17A5	Solute Carrier Family 17 Member 5
598.	HTR3A	5-Hydroxytryptamine Receptor 3A
599.	IFNA5	Interferon Alpha 5
600.	ZMPSTE24	Zinc Metallopeptidase STE24
601.	TEK	TEK Receptor Tyrosine Kinase
602.	FLT1	Fms Related Receptor Tyrosine Kinase 1
603.	SCARB1	Scavenger Receptor Class B Member 1
604.	ANGPT1	Angiopoietin 1
605.	HRH1	Histamine Receptor H1
606.	PHKG1	Phosphorylase Kinase Catalytic Subunit Gamma 1
607.	RAPSN	Receptor Associated Protein Of The Synapse
608.	CTSB	Cathepsin B
609.	CXCR2	C-X-C Motif Chemokine Receptor 2
610.	TLR8	Toll Like Receptor 8
611.	STAT2	Signal Transducer And Activator Of Transcription 2
612.	ITGAM	Integrin Subunit Alpha M
613.	IL4R	Interleukin 4 Receptor
614.	C5AR1	Complement C5a Receptor 1
615.	CLEC7A	C-Type Lectin Domain Containing 7A
616.	HFE	Homeostatic Iron Regulator

617.	QDPR	Quinoid Dihydropteridine Reductase
618.	TFAM	Transcription Factor A, Mitochondrial
619.	VDAC1	Voltage Dependent Anion Channel 1
620.	H6PD	Hexose-6-Phosphate Dehydrogenase/Glucose 1-Dehydrogenase
621.	OAS1	2'-5'-Oligoadenylate Synthetase 1
622.	PLN	Phospholamban
623.	IL22	Interleukin 22
624.	NAT2	N-Acetyltransferase 2
625.	ADAMTSL1	ADAMTS Like 1
626.	CCL4	C-C Motif Chemokine Ligand 4
627.	MAVS	Mitochondrial Antiviral Signaling Protein
628.	RAB1B	RAB1B, Member RAS Oncogene Family
629.	TRMU	TRNA Mitochondrial 2-Thiouridylase
630.	ARSD	Arylsulfatase D
631.	SEC22B	SEC22 Homolog B, Vesicle Trafficking Protein
632.	TPM3	Tropomyosin 3
633.	SERPINC1	Serpin Family C Member 1
634.	PRKN	Parkin RBR E3 Ubiquitin Protein Ligase
635.	CFH	Complement Factor H
636.	TYMP	Thymidine Phosphorylase
637.	IL12RB1	Interleukin 12 Receptor Subunit Beta 1
638.	PYGL	Glycogen Phosphorylase L
639.	AARS1	Alanyl-TRNA Synthetase 1
640.	IL15	Interleukin 15
641.	LARGE1	LARGE Xylosyl- And Glucuronyltransferase 1
642.	PDSS2	Decaprenyl Diphosphate Synthase Subunit 2
643.	POGLUT1	Protein O-Glucosyltransferase 1
644.	ALG14	ALG14 UDP-N-Acetylglucosaminyltransferase Subunit
645.	SYNC	Syncoilin, Intermediate Filament Protein
646.	KLHL3	Kelch Like Family Member 3
647.	RLN2	Relaxin 2
648.	RNF31	Ring Finger Protein 31
649.	DPP4	Dipeptidyl Peptidase 4
650.	ATP4A	ATPase H+/K+ Transporting Subunit Alpha
651.	CTRL	Chymotrypsin Like
652.	MLPH	Melanophilin
653.	APEH	Acylaminoacyl-Peptide Hydrolase
654.	EVL	Enah/Vasp-Like
655.	SENP6	SUMO Specific Peptidase 6
656.	CCDC83	Coiled-Coil Domain Containing 83
657.	FHIP1B	FHF Complex Subunit HOOK Interacting Protein 1B

658.	CYP2C9	Cytochrome P450 Family 2 Subfamily C Member 9
659.	MTHFR	Methylenetetrahydrofolate Reductase
660.	LEP	Leptin
661.	SQSTM1	Sequestosome 1
662.	GNAT2	G Protein Subunit Alpha Transducin 2
663.	PDE6C	Phosphodiesterase 6C
664.	CNGB3	Cyclic Nucleotide Gated Channel Subunit Beta 3
665.	PDE6H	Phosphodiesterase 6H
666.	ARV1	ARV1 Homolog, Fatty Acid Homeostasis Modulator
667.	CD300LD	CD300 Molecule Like Family Member D
668.	ABCC9	ATP Binding Cassette Subfamily C Member 9
669.	FLT3	Fms Related Receptor Tyrosine Kinase 3
670.	PTPN6	Protein Tyrosine Phosphatase Non-Receptor Type 6
671.	CYCS	Cytochrome C, Somatic
672.	CD27	CD27 Molecule
673.	PPARGC1A	PPARG Coactivator 1 Alpha
674.	RPSA	Ribosomal Protein SA
675.	MOG	Myelin Oligodendrocyte Glycoprotein
676.	HLA-G	Major Histocompatibility Complex, Class I, G
677.	AMBP	Alpha-1-Microglobulin/Bikunin Precursor
678.	IL9	Interleukin 9
679.	TRPV5	Transient Receptor Potential Cation Channel Subfamily V Member 5
680.	NDUFB8	NADH:Ubiquinone Oxidoreductase Subunit B8
681.	PF4	Platelet Factor 4
682.	RYR3	Ryanodine Receptor 3
683.	SURF1	SURF1 Cytochrome C Oxidase Assembly Factor
684.	MT-ND4L	Mitochondrially Encoded NADH:Ubiquinone Oxidoreductase Core Subunit 4L
685.	LYN	LYN Proto-Oncogene, Src Family Tyrosine Kinase
686.	HTR3B	5-Hydroxytryptamine Receptor 3B
687.	JAK2	Janus Kinase 2
688.	CASR	Calcium Sensing Receptor
689.	PPIB	Peptidylprolyl Isomerase B
690.	FLNA	Filamin A
691.	GP9	Glycoprotein IX Platelet
692.	HSPB8	Heat Shock Protein Family B (Small) Member 8
693.	TBX21	T-Box Transcription Factor 21
694.	ACTC1	Actin Alpha Cardiac Muscle 1
695.	RAB5A	RAB5A, Member RAS Oncogene Family
696.	SCO2	Synthesis Of Cytochrome C Oxidase 2
697.	BAG3	BAG Cochaperone 3
698.	COQ8A	Coenzyme Q8A

699.	RPS27A	Ribosomal Protein S27a
700.	AARS2	Alanyl-TRNA Synthetase 2, Mitochondrial
701.	COQ9	Coenzyme Q9
702.	CXCL11	C-X-C Motif Chemokine Ligand 11
703.	PDSS1	Decaprenyl Diphosphate Synthase Subunit 1
704.	RNPC3	RNA Binding Region (RNP1, RRM) Containing 3
705.	ACAD10	Acyl-CoA Dehydrogenase Family Member 10
706.	ZNF436	Zinc Finger Protein 436
707.	ACAD11	Acyl-CoA Dehydrogenase Family Member 11
708.	ZNF44	Zinc Finger Protein 44
709.	ZNF569	Zinc Finger Protein 569
710.	ZNF629	Zinc Finger Protein 629
711.	ZNF501	Zinc Finger Protein 501
712.	ZNF763	Zinc Finger Protein 763
713.	SPAST	Spastin
714.	SLPI	Secretory Leukocyte Peptidase Inhibitor
715.	SLC13A5	Solute Carrier Family 13 Member 5
716.	RHOBTB1	Rho Related BTB Domain Containing 1
717.	SUSD1	Sushi Domain Containing 1
718.	CIDEC	Cell Death Inducing DFFA Like Effector C
719.	CYP1A1	Cytochrome P450 Family 1 Subfamily A Member 1
720.	APOL3	Apolipoprotein L3
721.	STX16	Syntaxin 16
722.	TGM1	Transglutaminase 1
723.	C9orf72	C9orf72-SMCR8 Complex Subunit
724.	EMD	Emerin
725.	KCNA1	Potassium Voltage-Gated Channel Subfamily A Member 1
726.	PTPRC	Protein Tyrosine Phosphatase Receptor Type C
727.	CD40	CD40 Molecule
728.	GSR	Glutathione-Disulfide Reductase
729.	ANGPT2	Angiopoietin 2
730.	CDH1	Cadherin 1
731.	P4HB	Prolyl 4-Hydroxylase Subunit Beta
732.	PLG	Plasminogen
733.	CP	Ceruloplasmin
734.	ODC1	Ornithine Decarboxylase 1
735.	SLC12A1	Solute Carrier Family 12 Member 1
736.	CRYAB	Crystallin Alpha B
737.	FLNB	Filamin B
738.	PSMB9	Proteasome 20S Subunit Beta 9
739.	CHI3L1	Chitinase 3 Like 1

740.	KARS1	Lysyl-TRNA Synthetase 1
741.	PTH	Parathyroid Hormone
742.	TLR6	Toll Like Receptor 6
743.	CD2	CD2 Molecule
744.	CD47	CD47 Molecule
745.	CTSG	Cathepsin G
746.	ATP2B4	ATPase Plasma Membrane Ca2+ Transporting 4
747.	COX10	Cytochrome C Oxidase Assembly Factor Heme A:Farnesyltransferase COX10
748.	DHX9	DExH-Box Helicase 9
749.	EPO	Erythropoietin
750.	G6PC1	Glucose-6-Phosphatase Catalytic Subunit 1
751.	PUS1	Pseudouridine Synthase 1
752.	SELL	Selectin L
753.	SPG7	SPG7 Matrix AAA Peptidase Subunit, Paraplegin
754.	COQ6	Coenzyme Q6, Monooxygenase
755.	IL17F	Interleukin 17F
756.	STOM	Stomatin
757.	MICB	MHC Class I Polypeptide-Related Sequence B
758.	RSAD2	Radical S-Adenosyl Methionine Domain Containing 2
759.	AIF1	Allograft Inflammatory Factor 1
760.	ALG3	ALG3 Alpha-1,3- Mannosyltransferase
761.	KIR3DL1	Killer Cell Immunoglobulin Like Receptor, Three Ig Domains And Long Cytoplasmic Tail 1
762.	PIEZO1	Piezo Type Mechanosensitive Ion Channel Component 1 (Er Blood Group)
763.	SRM	Spermidine Synthase
764.	COQ4	Coenzyme Q4
765.	COQ8B	Coenzyme Q8B
766.	DECR1	2,4-Dienoyl-CoA Reductase 1
767.	OAS3	2'-5'-Oligoadenylate Synthetase 3
768.	TMPRSS11D	Transmembrane Serine Protease 11D
769.	TSLP	Thymic Stromal Lymphopoietin
770.	DMTN	Dematin Actin Binding Protein
771.	MYO9A	Myosin IXA
772.	CFAP53	Cilia And Flagella Associated Protein 53
773.	IFNA6	Interferon Alpha 6
774.	PIGW	Phosphatidylinositol Glycan Anchor Biosynthesis Class W
775.	IFNA14	Interferon Alpha 14
776.	IFNA21	Interferon Alpha 21
777.	IFNA8	Interferon Alpha 8
778.	IFNA10	Interferon Alpha 10
779.	MYOZ1	Myozenin 1
780.	IFNA13	Interferon Alpha 13

781.	IFNA17	Interferon Alpha 17
782.	IFNA7	Interferon Alpha 7
783.	IFNA16	Interferon Alpha 16
784.	HTR1A	5-Hydroxytryptamine Receptor 1A
785.	GGT1	Gamma-Glutamyltransferase 1
786.	HSPA5	Heat Shock Protein Family A (Hsp70) Member 5
787.	PTK2B	Protein Tyrosine Kinase 2 Beta
788.	NEU1	Neuraminidase 1
789.	GALNT3	Polypeptide N-Acetylgalactosaminyltransferase 3
790.	FCER2	Fc Epsilon Receptor II
791.	PCSK6	Proprotein Convertase Subtilisin/Kexin Type 6
792.	BCR	BCR Activator Of RhoGEF And GTPase
793.	COL3A1	Collagen Type III Alpha 1 Chain
794.	COL5A2	Collagen Type V Alpha 2 Chain
795.	TANGO2	Transport And Golgi Organization 2 Homolog
796.	SCN10A	Sodium Voltage-Gated Channel Alpha Subunit 10
797.	HMOX1	Heme Oxygenase 1
798.	ATP2A2	ATPase Sarcoplasmic/Endoplasmic Reticulum Ca2+ Transporting 2
799.	AXL	AXL Receptor Tyrosine Kinase
800.	GLUD1	Glutamate Dehydrogenase 1
801.	CD55	CD55 Molecule (Cromer Blood Group)
802.	CYBB	Cytochrome B-245 Beta Chain
803.	TBK1	TANK Binding Kinase 1
804.	ADAMTS13	ADAM Metallopeptidase With Thrombospondin Type 1 Motif 13
805.	ANXA5	Annexin A5
806.	TYRO3	TYRO3 Protein Tyrosine Kinase
807.	ISG15	ISG15 Ubiquitin Like Modifier
808.	NDUFS7	NADH:Ubiquinone Oxidoreductase Core Subunit S7
809.	SLC19A3	Solute Carrier Family 19 Member 3
810.	CX3CR1	C-X3-C Motif Chemokine Receptor 1
811.	HABP2	Hyaluronan Binding Protein 2
812.	UQCRC2	Ubiquinol-Cytochrome C Reductase Core Protein 2
813.	ATP2A3	ATPase Sarcoplasmic/Endoplasmic Reticulum Ca2+ Transporting 3
814.	NDUFS8	NADH:Ubiquinone Oxidoreductase Core Subunit S8
815.	ANK3	Ankyrin 3
816.	GSTM1	Glutathione S-Transferase Mu 1
817.	TNFRSF25	TNF Receptor Superfamily Member 25
818.	TREM1	Triggering Receptor Expressed On Myeloid Cells 1
819.	FCGR1A	Fc Gamma Receptor Ia
820.	IRS4	Insulin Receptor Substrate 4
821.	ESCO2	Establishment Of Sister Chromatid Cohesion N-Acetyltransferase 2

822.	RCN2	Reticulocalbin 2
823.	CCL22	C-C Motif Chemokine Ligand 22
824.	EEF1G	Eukaryotic Translation Elongation Factor 1 Gamma
825.	HSPA14	Heat Shock Protein Family A (Hsp70) Member 14
826.	RASGRP4	RAS Guanyl Releasing Protein 4
827.	ESCO1	Establishment Of Sister Chromatid Cohesion N-Acetyltransferase 1
828.	ASZ1	Ankyrin Repeat, SAM And Basic Leucine Zipper Domain Containing 1
829.	CCAR1	Cell Division Cycle And Apoptosis Regulator 1
830.	SPNS1	SPNS Lysolipid Transporter 1, Lysophospholipid
831.	MXRA8	Matrix Remodeling Associated 8
832.	JSRP1	Junctional Sarcoplasmic Reticulum Protein 1
833.	MARVELD3	MARVEL Domain Containing 3
834.	SLN	Sarcolipin
835.	PET117	PET117 Cytochrome C Oxidase Chaperone
836.	BICRAL	BICRA Like Chromatin Remodeling Complex Associated Protein
837.	HIGD1C	HIG1 Hypoxia Inducible Domain Family Member 1C
838.	CCDC177	Coiled-Coil Domain Containing 177
839.	DDX3X	DEAD-Box Helicase 3 X-Linked
840.	ERF	ETS2 Repressor Factor
841.	TXNL1	Thioredoxin Like 1
842.	ADIPOQ	Adiponectin, C1Q And Collagen Domain Containing
843.	PURA	Purine Rich Element Binding Protein A
844.	LMNB2	Lamin B2
845.	SLC6A3	Solute Carrier Family 6 Member 3
846.	ADRB1	Adrenoceptor Beta 1
847.	VEGFA	Vascular Endothelial Growth Factor A
848.	PRKAG2	Protein Kinase AMP-Activated Non-Catalytic Subunit Gamma 2
849.	FOS	Fos Proto-Oncogene, AP-1 Transcription Factor Subunit
850.	FXR1	FMR1 Autosomal Homolog 1
851.	GDAP1	Ganglioside Induced Differentiation Associated Protein 1
852.	SNCA	Synuclein Alpha
853.	ANPEP	Alanyl Aminopeptidase, Membrane
854.	FOXO3	Forkhead Box O3
855.	PDP1	Pyruvate Dehydrogenase Phosphatase Catalytic Subunit 1
856.	SLC40A1	Solute Carrier Family 40 Member 1
857.	KITLG	KIT Ligand
858.	PDE6B	Phosphodiesterase 6B
859.	TOR1A	Torsin Family 1 Member A
860.	BCS1L	BCS1 Homolog, Ubiquinol-Cytochrome C Reductase Complex Chaperone
861.	CXCL12	C-X-C Motif Chemokine Ligand 12
862.	HAVCR2	Hepatitis A Virus Cellular Receptor 2

863.	TIMP1	TIMP Metallopeptidase Inhibitor 1
864.	CD163	CD163 Molecule
865.	LIF	LIF Interleukin 6 Family Cytokine
866.	UBB	Ubiquitin B
867.	CCL7	C-C Motif Chemokine Ligand 7
868.	CLPB	ClpB Family Mitochondrial Disaggregase
869.	DARS2	Aspartyl-TRNA Synthetase 2, Mitochondrial
870.	MGAM	Maltase-Glucoamylase
871.	NDUFA9	NADH:Ubiquinone Oxidoreductase Subunit A9
872.	YARS2	Tyrosyl-TRNA Synthetase 2
873.	CNTF	Ciliary Neurotrophic Factor
874.	EEA1	Early Endosome Antigen 1
875.	GNPTAB	N-Acetylglucosamine-1-Phosphate Transferase Subunits Alpha And Beta
876.	HSPE1	Heat Shock Protein Family E (Hsp10) Member 1
877.	COX15	Cytochrome C Oxidase Assembly Homolog COX15
878.	COX20	Cytochrome C Oxidase Assembly Factor COX20
879.	IARS2	Isoleucyl-TRNA Synthetase 2, Mitochondrial
880.	PDCD10	Programmed Cell Death 10
881.	SLC17A6	Solute Carrier Family 17 Member 6
882.	VAMP7	Vesicle Associated Membrane Protein 7
883.	CCL17	C-C Motif Chemokine Ligand 17
884.	LTB	Lymphotoxin Beta
885.	TMEM70	Transmembrane Protein 70
886.	TOMM20	Translocase Of Outer Mitochondrial Membrane 20
887.	CYP3A7	Cytochrome P450 Family 3 Subfamily A Member 7
888.	FBXL4	F-Box And Leucine Rich Repeat Protein 4
889.	RBFOX2	RNA Binding Fox-1 Homolog 2
890.	SYNPO2	Synaptopodin 2
891.	FCHO2	FCH And Mu Domain Containing Endocytic Adaptor 2
892.	RAB24	RAB24, Member RAS Oncogene Family
893.	RAB26	RAB26, Member RAS Oncogene Family
894.	STX17	Syntaxin 17
895.	ABRA	Actin Binding Rho Activating Protein
896.	COX18	Cytochrome C Oxidase Assembly Factor COX18
897.	LRRC63	Leucine Rich Repeat Containing 63
898.	LIMS3	LIM Zinc Finger Domain Containing 3
899.	TPM1	Tropomyosin 1
900.	SCN1A	Sodium Voltage-Gated Channel Alpha Subunit 1
901.	CD274	CD274 Molecule
902.	ATXN10	Ataxin 10
903.	TNNT1	Troponin T1, Slow Skeletal Type

904.	CELF1	CUGBP Elav-Like Family Member 1
905.	LMOD3	Leiomodin 3
906.	MBNL1	Muscleblind Like Splicing Regulator 1
907.	MBNL2	Muscleblind Like Splicing Regulator 2
908.	NT5C1A	5'-Nucleotidase, Cytosolic IA
909.	NAV1	Neuron Navigator 1
910.	MBNL3	Muscleblind Like Splicing Regulator 3
911.	TNFRSF11B	TNF Receptor Superfamily Member 11b
912.	APOB	Apolipoprotein B
913.	ASTN2	Astrotactin 2
914.	PIK3CA	Phosphatidylinositol-4,5-Bisphosphate 3-Kinase Catalytic Subunit Alpha
915.	LPL	Lipoprotein Lipase
916.	PFKL	Phosphofructokinase, Liver Type
917.	DTNBP1	Dystrobrevin Binding Protein 1
918.	XK	X-Linked Kx Blood Group Antigen, Kell And VPS13A Binding Protein
919.	ACTN2	Actinin Alpha 2
920.	CYP24A1	Cytochrome P450 Family 24 Subfamily A Member 1
921.	PTHLH	Parathyroid Hormone Like Hormone
922.	IGHE	Immunoglobulin Heavy Constant Epsilon
923.	HRAS	HRas Proto-Oncogene, GTPase
924.	COL2A1	Collagen Type II Alpha 1 Chain
925.	COL5A1	Collagen Type V Alpha 1 Chain
926.	FBN2	Fibrillin 2
927.	FKBP14	FKBP Prolyl Isomerase 14
928.	NALCN	Sodium Leak Channel, Non-Selective
929.	RPL36A-HNRNPH2	RPL36A-HNRNPH2 Readthrough
930.	LOC112694756	Uncharaterized LOC112694756
931.	XPO1	Exportin 1
932.	CYP27B1	Cytochrome P450 Family 27 Subfamily B Member 1
933.	DDX1	DEAD-Box Helicase 1
934.	GC	GC Vitamin D Binding Protein
935.	KPNA2	Karyopherin Subunit Alpha 2
936.	KPNB1	Karyopherin Subunit Beta 1
937.	VAV1	Vav Guanine Nucleotide Exchange Factor 1
938.	KPNA1	Karyopherin Subunit Alpha 1
939.	LGMN	Legumain
940.	INSRR	Insulin Receptor Related Receptor
941.	CBY1	Chibby 1, Beta Catenin Antagonist
942.	SPAG1	Sperm Associated Antigen 1
943.	CALML4	Calmodulin Like 4
944.	EEFSEC	Eukaryotic Elongation Factor, Selenocysteine-TRNA Specific

945.	MTFR1	Mitochondrial Fission Regulator 1
946.	GLIS1	GLIS Family Zinc Finger 1
947.	LDLRAD3	Low Density Lipoprotein Receptor Class A Domain Containing 3
948.	SPRY3	Sprouty RTK Signaling Antagonist 3
949.	PDGFRB	Platelet Derived Growth Factor Receptor Beta
950.	LIPE	Lipase E, Hormone Sensitive Type
951.	HNRNPA2B1	Heterogeneous Nuclear Ribonucleoprotein A2/B1
952.	PPP2R2B	Protein Phosphatase 2 Regulatory Subunit Bbeta
953.	SERPINA3	Serpin Family A Member 3
954.	CD69	CD69 Molecule
955.	HNRNPH1	Heterogeneous Nuclear Ribonucleoprotein H1
956.	JPH3	Junctophilin 3
957.	ALPL	Alkaline Phosphatase, Biomineralization Associated
958.	BCL2	BCL2 Apoptosis Regulator
959.	MX1	MX Dynamin Like GTPase 1
960.	ASAH1	N-Acylsphingosine Amidohydrolase 1
961.	DLG4	Discs Large MAGUK Scaffold Protein 4
962.	PKD1	Polycystin 1, Transient Receptor Potential Channel Interacting
963.	COL6A1	Collagen Type VI Alpha 1 Chain
964.	COL6A2	Collagen Type VI Alpha 2 Chain
965.	RELN	Reelin
966.	ADGRG6	Adhesion G Protein-Coupled Receptor G6
967.	SETBP1	SET Binding Protein 1
968.	TIA1	TIA1 Cytotoxic Granule Associated RNA Binding Protein
969.	ASCC1	Activating Signal Cointegrator 1 Complex Subunit 1
970.	TOR1AIP1	Torsin 1A Interacting Protein 1
971.	CXCR4	C-X-C Motif Chemokine Receptor 4
972.	TYK2	Tyrosine Kinase 2
973.	NFE2L2	NFE2 Like BZIP Transcription Factor 2
974.	ADAM17	ADAM Metallopeptidase Domain 17
975.	CSNK2A1	Casein Kinase 2 Alpha 1
976.	FN1	Fibronectin 1
977.	GSTP1	Glutathione S-Transferase Pi 1
978.	STAT6	Signal Transducer And Activator Of Transcription 6
979.	TYMS	Thymidylate Synthetase
980.	ABCA1	ATP Binding Cassette Subfamily A Member 1
981.	LRRK2	Leucine Rich Repeat Kinase 2
982.	CD44	CD44 Molecule (IN Blood Group)
983.	GLB1	Galactosidase Beta 1
984.	ABCC1	ATP Binding Cassette Subfamily C Member 1 (ABCC1 Blood Group)
985.	AQP1	Aquaporin 1 (Colton Blood Group)

986.	ENG	Endoglin
987.	EPOR	Erythropoietin Receptor
988.	GCH1	GTP Cyclohydrolase 1
989.	GPX4	Glutathione Peroxidase 4
990.	KL	Klotho
991.	MMP8	Matrix Metallopeptidase 8
992.	SLC9A3	Solute Carrier Family 9 Member A3
993.	TPT1	Tumor Protein, Translationally-Controlled 1
994.	CD38	CD38 Molecule
995.	CD59	CD59 Molecule (CD59 Blood Group)
996.	ENPEP	Glutamyl Aminopeptidase
997.	FGF23	Fibroblast Growth Factor 23
998.	IRF1	Interferon Regulatory Factor 1
999.	KCNB1	Potassium Voltage-Gated Channel Subfamily B Member 1
1000.	MMP12	Matrix Metallopeptidase 12
1001.	P2RX7	Purinergic Receptor P2X 7
1002.	PDXK	Pyridoxal Kinase
1003.	PLA2G2A	Phospholipase A2 Group IIA
1004.	PTGS1	Prostaglandin-Endoperoxide Synthase 1
1005.	TRAF3	TNF Receptor Associated Factor 3
1006.	TRAF6	TNF Receptor Associated Factor 6
1007.	TXN	Thioredoxin
1008.	UMPS	Uridine Monophosphate Synthetase
1009.	CARD9	Caspase Recruitment Domain Family Member 9
1010.	CD81	CD81 Molecule
1011.	CYB5R3	Cytochrome B5 Reductase 3
1012.	GJB2	Gap Junction Protein Beta 2
1013.	ORC1	Origin Recognition Complex Subunit 1
1014.	PHB1	Prohibitin 1
1015.	PRDX6	Peroxiredoxin 6
1016.	RBP4	Retinol Binding Protein 4
1017.	SCP2	Sterol Carrier Protein 2
1018.	SPP1	Secreted Phosphoprotein 1
1019.	TJP1	Tight Junction Protein 1
1020.	BSG	Basigin (Ok Blood Group)
1021.	COX4I1	Cytochrome C Oxidase Subunit 4I1
1022.	EIF2AK4	Eukaryotic Translation Initiation Factor 2 Alpha Kinase 4
1023.	IL10RA	Interleukin 10 Receptor Subunit Alpha
1024.	MBP	Myelin Basic Protein
1025.	NME2	NME/NM23 Nucleoside Diphosphate Kinase 2
1026.	NRP2	Neuropilin 2

1027.	PLAUR	Plasminogen Activator, Urokinase Receptor
1028.	POLR2A	RNA Polymerase II Subunit A
1029.	PRDX5	Peroxiredoxin 5
1030.	RAP1A	RAP1A, Member Of RAS Oncogene Family
1031.	SCO1	Synthesis Of Cytochrome C Oxidase 1
1032.	SHMT1	Serine Hydroxymethyltransferase 1
1033.	SLC34A1	Solute Carrier Family 34 Member 1
1034.	ADCY9	Adenylate Cyclase 9
1035.	AGO2	Argonaute RISC Catalytic Component 2
1036.	AICDA	Activation Induced Cytidine Deaminase
1037.	FCGRT	Fc Gamma Receptor And Transporter
1038.	FCN2	Ficolin 2
1039.	GDF15	Growth Differentiation Factor 15
1040.	GLRX	Glutaredoxin
1041.	HAMP	Hepcidin Antimicrobial Peptide
1042.	IARS1	Isoleucyl-TRNA Synthetase 1
1043.	NPPB	Natriuretic Peptide B
1044.	NR1I2	Nuclear Receptor Subfamily 1 Group I Member 2
1045.	TRIM25	Tripartite Motif Containing 25
1046.	VNN1	Vanin 1
1047.	CD2AP	CD2 Associated Protein
1048.	CSRP3	Cysteine And Glycine Rich Protein 3
1049.	DNAJC5	DnaJ Heat Shock Protein Family (Hsp40) Member C5
1050.	HBA1	Hemoglobin Subunit Alpha 1
1051.	LILRB1	Leukocyte Immunoglobulin Like Receptor B1
1052.	MUC5B	Mucin 5B, Oligomeric Mucus/Gel-Forming
1053.	PHEX	Phosphate Regulating Endopeptidase X-Linked
1054.	PROCR	Protein C Receptor
1055.	PYGB	Glycogen Phosphorylase B
1056.	SETD1A	SET Domain Containing 1A, Histone Lysine Methyltransferase
1057.	CCT7	Chaperonin Containing TCP1 Subunit 7
1058.	DDX39B	DExD-Box Helicase 39B
1059.	EXOSC9	Exosome Component 9
1060.	HBA2	Hemoglobin Subunit Alpha 2
1061.	HBG2	Hemoglobin Subunit Gamma 2
1062.	IFITM3	Interferon Induced Transmembrane Protein 3
1063.	KIF1C	Kinesin Family Member 1C
1064.	KLRC1	Killer Cell Lectin Like Receptor C1
1065.	LAG3	Lymphocyte Activating 3
1066.	LAIR1	Leukocyte Associated Immunoglobulin Like Receptor 1
1067.	PCYT2	Phosphate Cytidylyltransferase 2, Ethanolamine

1068.	PDPN	Podoplanin
1069.	POLR2B	RNA Polymerase II Subunit B
1070.	SECISBP2	SECIS Binding Protein 2
1071.	SERPINB1	Serpin Family B Member 1
1072.	SH3KBP1	SH3 Domain Containing Kinase Binding Protein 1
1073.	SLC34A3	Solute Carrier Family 34 Member 3
1074.	SPEG	Striated Muscle Enriched Protein Kinase
1075.	TRNT1	TRNA Nucleotidyl Transferase 1
1076.	VARS2	Valyl-TRNA Synthetase 2, Mitochondrial
1077.	G3BP1	G3BP Stress Granule Assembly Factor 1
1078.	HBS1L	HBS1 Like Translational GTPase
1079.	RPL4	Ribosomal Protein L4
1080.	SPG11	SPG11 Vesicle Trafficking Associated, Spatacsin
1081.	CD1A	CD1a Molecule
1082.	FGF21	Fibroblast Growth Factor 21
1083.	FUT9	Fucosyltransferase 9
1084.	GTPBP3	GTP Binding Protein 3, Mitochondrial
1085.	HLA-DRB5	Major Histocompatibility Complex, Class II, DR Beta 5
1086.	IL23A	Interleukin 23 Subunit Alpha
1087.	LILRB2	Leukocyte Immunoglobulin Like Receptor B2
1088.	NCR3	Natural Cytotoxicity Triggering Receptor 3
1089.	RHCE	Rh Blood Group CcEe Antigens
1090.	CLEC4G	C-Type Lectin Domain Family 4 Member G
1091.	CPQ	Carboxypeptidase Q
1092.	ECSIT	ECSIT Signaling Integrator
1093.	FASTKD2	FAST Kinase Domains 2
1094.	KPNA4	Karyopherin Subunit Alpha 4
1095.	NAIP	NLR Family Apoptosis Inhibitory Protein
1096.	NAP1L4	Nucleosome Assembly Protein 1 Like 4
1097.	SFTPA2	Surfactant Protein A2
1098.	ABO	ABO, Alpha 1-3-N-Acetylgalactosaminyltransferase And Alpha 1-3-Galactosyltransferase
1099.	CD101	CD101 Molecule
1100.	CLEC6A	C-Type Lectin Domain Containing 6A
1101.	HBE1	Hemoglobin Subunit Epsilon 1
1102.	HM13	Histocompatibility Minor 13
1103.	RNASEH2B	Ribonuclease H2 Subunit B
1104.	RNF170	Ring Finger Protein 170
1105.	SCUBE1	Signal Peptide, CUB Domain And EGF Like Domain Containing 1
1106.	TIMD4	T Cell Immunoglobulin And Mucin Domain Containing 4
1107.	UCN	Urocortin
1108.	UGT3A2	UDP Glycosyltransferase Family 3 Member A2

1109.	CLEC4E	C-Type Lectin Domain Family 4 Member E
1110.	HSPB7	Heat Shock Protein Family B (Small) Member 7
1111.	KIR2DL3	Killer Cell Immunoglobulin Like Receptor, Two Ig Domains And Long Cytoplasmic Tail 3
1112.	NAP1L1	Nucleosome Assembly Protein 1 Like 1
1113.	SENP7	SUMO Specific Peptidase 7
1114.	ZDHHC19	Zinc Finger DHHC-Type Palmitoyltransferase 19
1115.	CGAS	Cyclic GMP-AMP Synthase
1116.	FCRL4	Fc Receptor Like 4
1117.	G3BP2	G3BP Stress Granule Assembly Factor 2
1118.	ZDHHC2	Zinc Finger DHHC-Type Palmitoyltransferase 2
1119.	MTERF1	Mitochondrial Transcription Termination Factor 1
1120.	NRAP	Nebulin Related Anchoring Protein
1121.	XRN1	5'-3' Exoribonuclease 1
1122.	XIRP1	Xin Actin Binding Repeat Containing 1
1123.	XIRP2	Xin Actin Binding Repeat Containing 2
1124.	LIPJ	Lipase Family Member J
1125.	LIPM	Lipase Family Member M
1126.	RMDN2	Regulator Of Microtubule Dynamics 2
1127.	TIGD4	Tigger Transposable Element Derived 4
1128.	GYPE	Glycophorin E (MNS Blood Group)
1129.	CCL3L1	C-C Motif Chemokine Ligand 3 Like 1
1130.	CHRM3	Cholinergic Receptor Muscarinic 3
1131.	AIRE	Autoimmune Regulator
1132.	PCYT1A	Phosphate Cytidylyltransferase 1A, Choline
1133.	CHRNE	Cholinergic Receptor Nicotinic Epsilon Subunit
1134.	HTT	Huntingtin
1135.	ELN	Elastin
1136.	NOP56	NOP56 Ribonucleoprotein
1137.	ATXN7	Ataxin 7
1138.	HNRNPDL	Heterogeneous Nuclear Ribonucleoprotein D Like
1139.	KHDRBS1	KH RNA Binding Domain Containing, Signal Transduction Associated 1
1140.	SMN1	Survival Of Motor Neuron 1, Telomeric
1141.	SNRNP70	Small Nuclear Ribonucleoprotein U1 Subunit 70
1142.	AGPAT1	1-Acylglycerol-3-Phosphate O-Acyltransferase 1
1143.	SSPN	Sarcospan
1144.	MTUS1	Microtubule Associated Scaffold Protein 1
1145.	MTUS2	Microtubule Associated Scaffold Protein 2
1146.	GAREM2	GRB2 Associated Regulator Of MAPK1 Subtype 2
1147.	UNC45B	Unc-45 Myosin Chaperone B
1148.	NDUFAF7	NADH:Ubiquinone Oxidoreductase Complex Assembly Factor 7
1149.	LACTB	Lactamase Beta

1150.	MMP2	Matrix Metallopeptidase 2
1151.	IL37	Interleukin 37
1152.	GAD1	Glutamate Decarboxylase 1
1153.	TH	Tyrosine Hydroxylase
1154.	DRD2	Dopamine Receptor D2
1155.	PKM	Pyruvate Kinase M1/2
1156.	VKORC1	Vitamin K Epoxide Reductase Complex Subunit 1
1157.	CHRM2	Cholinergic Receptor Muscarinic 2
1158.	LIPA	Lipase A, Lysosomal Acid Type
1159.	PON1	Paraoxonase 1
1160.	ADRA2A	Adrenoceptor Alpha 2A
1161.	CETP	Cholesteryl Ester Transfer Protein
1162.	GAL	Galanin And GMAP Prepropeptide
1163.	TXNRD2	Thioredoxin Reductase 2
1164.	GYS2	Glycogen Synthase 2
1165.	PFKP	Phosphofructokinase, Platelet
1166.	DISC1	DISC1 Scaffold Protein
1167.	SLC12A4	Solute Carrier Family 12 Member 4
1168.	OXT	Oxytocin/Neurophysin I Prepropeptide
1169.	ESR1	Estrogen Receptor 1
1170.	DTNA	Dystrobrevin Alpha
1171.	HTR2A	5-Hydroxytryptamine Receptor 2A
1172.	JAK1	Janus Kinase 1
1173.	SREBF1	Sterol Regulatory Element Binding Transcription Factor 1
1174.	DCN	Decorin
1175.	TNNT2	Troponin T2, Cardiac Type
1176.	ATXN3	Ataxin 3
1177.	MYH6	Myosin Heavy Chain 6
1178.	SUCLA2	Succinate-CoA Ligase ADP-Forming Subunit Beta
1179.	ATXN1	Ataxin 1
1180.	ATXN2	Ataxin 2
1181.	KCNA3	Potassium Voltage-Gated Channel Subfamily A Member 3
1182.	PABPN1	Poly(A) Binding Protein Nuclear 1
1183.	S100A9	S100 Calcium Binding Protein A9
1184.	DAB1	DAB Adaptor Protein 1
1185.	FABP4	Fatty Acid Binding Protein 4
1186.	TRIM33	Tripartite Motif Containing 33
1187.	MPV17	Mitochondrial Inner Membrane Protein MPV17
1188.	LAPTM4A	Lysosomal Protein Transmembrane 4 Alpha
1189.	MYBPC2	Myosin Binding Protein C2
1190.	NOTCH2NLC	Notch 2 N-Terminal Like C

1191.	PCSK9	Proprotein Convertase Subtilisin/Kexin Type 9
1192.	BGLAP	Bone Gamma-Carboxyglutamate Protein
1193.	MYLK	Myosin Light Chain Kinase
1194.	OPRM1	Opioid Receptor Mu 1
1195.	SDHB	Succinate Dehydrogenase Complex Iron Sulfur Subunit B
1196.	CACNA1D	Calcium Voltage-Gated Channel Subunit Alpha1 D
1197.	CD99	CD99 Molecule (Xg Blood Group)
1198.	ATP1A2	ATPase Na+/K+ Transporting Subunit Alpha 2
1199.	COL6A3	Collagen Type VI Alpha 3 Chain
1200.	DYNC1H1	Dynein Cytoplasmic 1 Heavy Chain 1
1201.	GLE1	GLE1 RNA Export Mediator
1202.	PIEZO2	Piezo Type Mechanosensitive Ion Channel Component 2
1203.	FAM111B	FAM111 Trypsin Like Peptidase B
1204.	HSPB1	Heat Shock Protein Family B (Small) Member 1
1205.	GATA3	GATA Binding Protein 3
1206.	SIRT1	Sirtuin 1
1207.	HMGB1	High Mobility Group Box 1
1208.	CLU	Clusterin
1209.	PINK1	PTEN Induced Kinase 1
1210.	TRPV6	Transient Receptor Potential Cation Channel Subfamily V Member 6
1211.	DOCK8	Dedicator Of Cytokinesis 8
1212.	NEFH	Neurofilament Heavy Chain
1213.	ASL	Argininosuccinate Lyase
1214.	BDKRB2	Bradykinin Receptor B2
1215.	CST3	Cystatin C
1216.	CEACAM5	CEA Cell Adhesion Molecule 5
1217.	CYP2R1	Cytochrome P450 Family 2 Subfamily R Member 1
1218.	NARS2	Asparaginyl-TRNA Synthetase 2, Mitochondrial
1219.	NDUFA12	NADH:Ubiquinone Oxidoreductase Subunit A12
1220.	WARS2	Tryptophanyl TRNA Synthetase 2, Mitochondrial
1221.	COX6A1	Cytochrome C Oxidase Subunit 6A1
1222.	ELAC2	ElaC Ribonuclease Z 2
1223.	NDUFA10	NADH:Ubiquinone Oxidoreductase Subunit A10
1224.	NDUFA6	NADH:Ubiquinone Oxidoreductase Subunit A6
1225.	NDUFA8	NADH:Ubiquinone Oxidoreductase Subunit A8
1226.	TMEM43	Transmembrane Protein 43
1227.	TSFM	Ts Translation Elongation Factor, Mitochondrial
1228.	UQCRB	Ubiquinol-Cytochrome C Reductase Binding Protein
1229.	VARS1	Valyl-TRNA Synthetase 1
1230.	COX4I2	Cytochrome C Oxidase Subunit 4I2
1231.	MRPS22	Mitochondrial Ribosomal Protein S22

1232.	RARS2	Arginyl-TRNA Synthetase 2, Mitochondrial
1233.	CDC27	Cell Division Cycle 27
1234.	CYP2C18	Cytochrome P450 Family 2 Subfamily C Member 18
1235.	NDUFA2	NADH:Ubiquinone Oxidoreductase Subunit A2
1236.	CALML3	Calmodulin Like 3
1237.	CHCHD2	Coiled-Coil-Helix-Coiled-Coil-Helix Domain Containing 2
1238.	COX8A	Cytochrome C Oxidase Subunit 8A
1239.	MTFMT	Mitochondrial Methionyl-TRNA Formyltransferase
1240.	MYOZ2	Myozenin 2
1241.	NDUFB7	NADH:Ubiquinone Oxidoreductase Subunit B7
1242.	NDUFC2	NADH:Ubiquinone Oxidoreductase Subunit C2
1243.	TFB1M	Transcription Factor B1, Mitochondrial
1244.	TMEM126A	Transmembrane Protein 126A
1245.	CCL24	C-C Motif Chemokine Ligand 24
1246.	FAM3B	FAM3 Metabolism Regulating Signaling Molecule B
1247.	KCNK5	Potassium Two Pore Domain Channel Subfamily K Member 5
1248.	CALML5	Calmodulin Like 5
1249.	NSMCE3	NSE3 Homolog, SMC5-SMC6 Complex Component
1250.	PDLIM3	PDZ And LIM Domain 3
1251.	PUS3	Pseudouridine Synthase 3
1252.	TIMM9	Translocase Of Inner Mitochondrial Membrane 9
1253.	CCL13	C-C Motif Chemokine Ligand 13
1254.	COX6A2	Cytochrome C Oxidase Subunit 6A2
1255.	MRM2	Mitochondrial RRNA Methyltransferase 2
1256.	QTRT1	Queuine TRNA-Ribosyltransferase Catalytic Subunit 1
1257.	TIMM10	Translocase Of Inner Mitochondrial Membrane 10
1258.	CDK5RAP1	CDK5 Regulatory Subunit Associated Protein 1
1259.	CHCHD4	Coiled-Coil-Helix-Coiled-Coil-Helix Domain Containing 4
1260.	CTTNBP2	Cortactin Binding Protein 2
1261.	HCFC2	Host Cell Factor C2
1262.	MPP2	MAGUK P55 Scaffold Protein 2
1263.	MTCH1	Mitochondrial Carrier 1
1264.	NDUFAF6	NADH:Ubiquinone Oxidoreductase Complex Assembly Factor 6
1265.	OSGEPL1	O-Sialoglycoprotein Endopeptidase Like 1
1266.	SYNM	Synemin
1267.	PGP	Phosphoglycolate Phosphatase
1268.	PRAM1	PML-RARA Regulated Adaptor Molecule 1
1269.	CALML6	Calmodulin Like 6
1270.	COA3	Cytochrome C Oxidase Assembly Factor 3
1271.	DNAJC28	DnaJ Heat Shock Protein Family (Hsp40) Member C28
1272.	VWA8	Von Willebrand Factor A Domain Containing 8

1273.	TRMT61B	TRNA Methyltransferase 61B
1274.	NDUFAF8	NADH:Ubiquinone Oxidoreductase Complex Assembly Factor 8
1275.	PDF	Peptide Deformylase, Mitochondrial
1276.	CYP19A1	Cytochrome P450 Family 19 Subfamily A Member 1
1277.	NPY	Neuropeptide Y
1278.	FAM131B	Family With Sequence Similarity 131 Member B
1279.	CD79A	CD79a Molecule
1280.	CDH5	Cadherin 5
1281.	CXCL1	C-X-C Motif Chemokine Ligand 1
1282.	GLRX5	Glutaredoxin 5
1283.	PCDH1	Protocadherin 1
1284.	NGF	Nerve Growth Factor
1285.	APP	Amyloid Beta Precursor Protein
1286.	IL6R	Interleukin 6 Receptor
1287.	MME	Membrane Metalloendopeptidase
1288.	CACNA1C	Calcium Voltage-Gated Channel Subunit Alpha1 C
1289.	NCAM1	Neural Cell Adhesion Molecule 1
1290.	COL1A2	Collagen Type I Alpha 2 Chain
1291.	GRM5	Glutamate Metabotropic Receptor 5
1292.	MUC1	Mucin 1, Cell Surface Associated
1293.	WT1	WT1 Transcription Factor
1294.	IL5RA	Interleukin 5 Receptor Subunit Alpha
1295.	KCNA2	Potassium Voltage-Gated Channel Subfamily A Member 2
1296.	SLC2A4	Solute Carrier Family 2 Member 4
1297.	CNTNAP2	Contactin Associated Protein 2
1298.	GCDH	Glutaryl-CoA Dehydrogenase
1299.	LAMP2	Lysosomal Associated Membrane Protein 2
1300.	MTMR2	Myotubularin Related Protein 2
1301.	SCN1B	Sodium Voltage-Gated Channel Beta Subunit 1
1302.	EPX	Eosinophil Peroxidase
1303.	LGI1	Leucine Rich Glioma Inactivated 1
1304.	ACADSB	Acyl-CoA Dehydrogenase Short/Branched Chain
1305.	DGCR8	DGCR8 Microprocessor Complex Subunit
1306.	SCN7A	Sodium Voltage-Gated Channel Alpha Subunit 7
1307.	SAE1	SUMO1 Activating Enzyme Subunit 1
1308.	UCP1	Uncoupling Protein 1
1309.	HNRNPC	Heterogeneous Nuclear Ribonucleoprotein C
1310.	SYNE1	Spectrin Repeat Containing Nuclear Envelope Protein 1
1311.	CYFIP1	Cytoplasmic FMR1 Interacting Protein 1
1312.	FXR2	FMR1 Autosomal Homolog 2
1313.	KIF21A	Kinesin Family Member 21A

1314.	SIX5	SIX Homeobox 5
1315.	CCL26	C-C Motif Chemokine Ligand 26
1316.	HNRNPA3	Heterogeneous Nuclear Ribonucleoprotein A3
1317.	MYH1	Myosin Heavy Chain 1
1318.	SLC52A1	Solute Carrier Family 52 Member 1
1319.	MYH8	Myosin Heavy Chain 8
1320.	B4GAT1	Beta-1,4-Glucuronyltransferase 1
1321.	ZCCHC13	Zinc Finger CCHC-Type Containing 13
1322.	YIPF7	Yip1 Domain Family Member 7
1323.	MYMX	Myomixer, Myoblast Fusion Factor
1324.	TPMT	Thiopurine S-Methyltransferase
1325.	MUTYH	MutY DNA Glycosylase
1326.	POPDC3	Popeye Domain Containing 3
1327.	KDR	Kinase Insert Domain Receptor
1328.	PIK3CD	Phosphatidylinositol-4,5-Bisphosphate 3-Kinase Catalytic Subunit Delta
1329.	LEPR	Leptin Receptor
1330.	ACACA	Acetyl-CoA Carboxylase Alpha
1331.	NR3C1	Nuclear Receptor Subfamily 3 Group C Member 1
1332.	PIK3CG	Phosphatidylinositol-4,5-Bisphosphate 3-Kinase Catalytic Subunit Gamma
1333.	PIK3CB	Phosphatidylinositol-4,5-Bisphosphate 3-Kinase Catalytic Subunit Beta
1334.	PIK3C2B	Phosphatidylinositol-4-Phosphate 3-Kinase Catalytic Subunit Type 2 Beta
1335.	MTOR	Mechanistic Target Of Rapamycin Kinase
1336.	EGFR	Epidermal Growth Factor Receptor
1337.	AKT1	AKT Serine/Threonine Kinase 1
1338.	ITGB2	Integrin Subunit Beta 2
1339.	MUSK	Muscle Associated Receptor Tyrosine Kinase
1340.	SPTAN1	Spectrin Alpha, Non-Erythrocytic 1
1341.	COL4A1	Collagen Type IV Alpha 1 Chain
1342.	ENO1	Enolase 1
1343.	KCNJ5	Potassium Inwardly Rectifying Channel Subfamily J Member 5
1344.	SCN3A	Sodium Voltage-Gated Channel Alpha Subunit 3
1345.	SLC11A1	Solute Carrier Family 11 Member 1
1346.	ANO1	Anoctamin 1
1347.	CD34	CD34 Molecule
1348.	FUS	FUS RNA Binding Protein
1349.	PRODH	Proline Dehydrogenase 1
1350.	SI	Sucrase-Isomaltase
1351.	TGFA	Transforming Growth Factor Alpha
1352.	CHRNB1	Cholinergic Receptor Nicotinic Beta 1 Subunit
1353.	PRL	Prolactin
1354.	RBBP8	RB Binding Protein 8, Endonuclease

1355.	SUCLG1	Succinate-CoA Ligase GDP/ADP-Forming Subunit Alpha
1356.	CHD8	Chromodomain Helicase DNA Binding Protein 8
1357.	PVR	PVR Cell Adhesion Molecule
1358.	TBC1D4	TBC1 Domain Family Member 4
1359.	CALD1	Caldesmon 1
1360.	CD5	CD5 Molecule
1361.	CFHR1	Complement Factor H Related 1
1362.	CLPX	Caseinolytic Mitochondrial Matrix Peptidase Chaperone Subunit X
1363.	CYP26A1	Cytochrome P450 Family 26 Subfamily A Member 1
1364.	DPP10	Dipeptidyl Peptidase Like 10
1365.	GCG	Glucagon
1366.	IVNS1ABP	Influenza Virus NS1A Binding Protein
1367.	PLAA	Phospholipase A2 Activating Protein
1368.	SBF1	SET Binding Factor 1
1369.	SLC52A3	Solute Carrier Family 52 Member 3
1370.	TAC1	Tachykinin Precursor 1
1371.	B3GALNT2	Beta-1,3-N-Acetylgalactosaminyltransferase 2
1372.	LIMS2	LIM Zinc Finger Domain Containing 2
1373.	POLRMT	RNA Polymerase Mitochondrial
1374.	RNASE2	Ribonuclease A Family Member 2
1375.	SIGLEC8	Sialic Acid Binding Ig Like Lectin 8
1376.	SRSF6	Serine And Arginine Rich Splicing Factor 6
1377.	CLDN5	Claudin 5
1378.	DPYSL5	Dihydropyrimidinase Like 5
1379.	HTATIP2	HIV-1 Tat Interactive Protein 2
1380.	LRP12	LDL Receptor Related Protein 12
1381.	SMN2	Survival Of Motor Neuron 2, Centromeric
1382.	SUN2	Sad1 And UNC84 Domain Containing 2
1383.	SYNE2	Spectrin Repeat Containing Nuclear Envelope Protein 2
1384.	YBX3	Y-Box Binding Protein 3
1385.	CENPB	Centromere Protein B
1386.	CIAO1	Cytosolic Iron-Sulfur Assembly Component 1
1387.	HACD1	3-Hydroxyacyl-CoA Dehydratase 1
1388.	MTMR1	Myotubularin Related Protein 1
1389.	NUBP1	NUBP Iron-Sulfur Cluster Assembly Factor 1, Cytosolic
1390.	PLA2R1	Phospholipase A2 Receptor 1
1391.	MMS19	MMS19 Homolog, Cytosolic Iron-Sulfur Assembly Component
1392.	CAVIN3	Caveolae Associated Protein 3
1393.	MGME1	Mitochondrial Genome Maintenance Exonuclease 1
1394.	RO60	Ro60, Y RNA Binding Protein
1395.	DMWD	DM1 Locus, WD Repeat Containing

1396.	LCLAT1	Lysocardiolipin Acyltransferase 1
1397.	CIAO2B	Cytosolic Iron-Sulfur Assembly Component 2B
1398.	IGLON5	IgLON Family Member 5
1399.	LRRC39	Leucine Rich Repeat Containing 39
1400.	CAVIN4	Caveolae Associated Protein 4
1401.	CIAO2A	Cytosolic Iron-Sulfur Assembly Component 2A
1402.	OR5AS1	Olfactory Receptor Family 5 Subfamily AS Member 1
1403.	OR5D14	Olfactory Receptor Family 5 Subfamily D Member 14
1404.	RIMBP3B	RIMS Binding Protein 3B
1405.	TRB	T Cell Receptor Beta Locus
1406.	PRKCA	Protein Kinase C Alpha
1407.	C1S	Complement C1s
1408.	PAX7	Paired Box 7
1409.	AMD1	Adenosylmethionine Decarboxylase 1
1410.	PTMA	Prothymosin Alpha
1411.	FBXO32	F-Box Protein 32
1412.	NQO1	NAD(P)H Quinone Dehydrogenase 1
1413.	TDO2	Tryptophan 2,3-Dioxygenase
1414.	PLCG2	Phospholipase C Gamma 2
1415.	RET	Ret Proto-Oncogene
1416.	TGFBR1	Transforming Growth Factor Beta Receptor 1
1417.	GRIN2B	Glutamate Ionotropic Receptor NMDA Type Subunit 2B
1418.	TGFBR2	Transforming Growth Factor Beta Receptor 2
1419.	AR	Androgen Receptor
1420.	TGFB2	Transforming Growth Factor Beta 2
1421.	IKZF1	IKAROS Family Zinc Finger 1
1422.	GPI	Glucose-6-Phosphate Isomerase
1423.	CRKL	CRK Like Proto-Oncogene, Adaptor Protein
1424.	HNF1A	HNF1 Homeobox A
1425.	PLOD1	Procollagen-Lysine,2-Oxoglutarate 5-Dioxygenase 1
1426.	ARID1B	AT-Rich Interaction Domain 1B
1427.	EFEMP1	EGF Containing Fibulin Extracellular Matrix Protein 1
1428.	ELP1	Elongator Acetyltransferase Complex Subunit 1
1429.	LTBP2	Latent Transforming Growth Factor Beta Binding Protein 2
1430.	CNTNAP1	Contactin Associated Protein 1
1431.	PPP1R3A	Protein Phosphatase 1 Regulatory Subunit 3A
1432.	BICD2	BICD Cargo Adaptor 2
1433.	TLN1	Talin 1
1434.	SPECC1L	Sperm Antigen With Calponin Homology And Coiled-Coil Domains 1 Like
1435.	LRRC56	Leucine Rich Repeat Containing 56
1436.	BLTP1	Bridge-Like Lipid Transfer Protein Family Member 1

1437.	LDLRAD2	Low Density Lipoprotein Receptor Class A Domain Containing 2
1438.	MILR1	Mast Cell Immunoglobulin Like Receptor 1
1439.	IGK	Immunoglobulin Kappa Locus
1440.	AVP	Arginine Vasopressin
1441.	IRF9	Interferon Regulatory Factor 9
1442.	CALM3	Calmodulin 3
1443.	UNC119	Unc-119 Lipid Binding Chaperone
1444.	SCGB1A1	Secretoglobin Family 1A Member 1
1445.	DEFB4A	Defensin Beta 4A
1446.	ENPP1	Ectonucleotide Pyrophosphatase/Phosphodiesterase 1
1447.	BTK	Bruton Tyrosine Kinase
1448.	MDM2	MDM2 Proto-Oncogene
1449.	STAT3	Signal Transducer And Activator Of Transcription 3
1450.	CDKN2A	Cyclin Dependent Kinase Inhibitor 2A
1451.	EGF	Epidermal Growth Factor
1452.	NOTCH3	Notch Receptor 3
1453.	PTCH1	Patched 1
1454.	EIF2AK2	Eukaryotic Translation Initiation Factor 2 Alpha Kinase 2
1455.	GBA1	Glucosylceramidase Beta 1
1456.	KCNQ1	Potassium Voltage-Gated Channel Subfamily Q Member 1
1457.	NOS1	Nitric Oxide Synthase 1
1458.	FST	Follistatin
1459.	TNNI3	Troponin I3, Cardiac Type
1460.	F9	Coagulation Factor IX
1461.	KCNJ1	Potassium Inwardly Rectifying Channel Subfamily J Member 1
1462.	KCNQ2	Potassium Voltage-Gated Channel Subfamily Q Member 2
1463.	TGM2	Transglutaminase 2
1464.	WNK1	WNK Lysine Deficient Protein Kinase 1
1465.	COMP	Cartilage Oligomeric Matrix Protein
1466.	GJB1	Gap Junction Protein Beta 1
1467.	GLRB	Glycine Receptor Beta
1468.	KCNJ10	Potassium Inwardly Rectifying Channel Subfamily J Member 10
1469.	MECP2	Methyl-CpG Binding Protein 2
1470.	PAX3	Paired Box 3
1471.	SMARCB1	SWI/SNF Related, Matrix Associated, Actin Dependent Regulator Of Chromatin, Subfamily B, Member 1
1472.	SYP	Synaptophysin
1473.	ECHS1	Enoyl-CoA Hydratase, Short Chain 1
1474.	HNRNPK	Heterogeneous Nuclear Ribonucleoprotein K
1475.	SNTA1	Syntrophin Alpha 1
1476.	AFG3L2	AFG3 Like Matrix AAA Peptidase Subunit 2
1477.	CACNA1F	Calcium Voltage-Gated Channel Subunit Alpha1 F

1478.	CELF2	CUGBP Elav-Like Family Member 2
1479.	CSF1	Colony Stimulating Factor 1
1480.	DROSHA	Drosha Ribonuclease III
1481.	EIF4A1	Eukaryotic Translation Initiation Factor 4A1
1482.	FIG4	FIG4 Phosphoinositide 5-Phosphatase
1483.	NR0B1	Nuclear Receptor Subfamily 0 Group B Member 1
1484.	WNK4	WNK Lysine Deficient Protein Kinase 4
1485.	AMPH	Amphiphysin
1486.	EIF4A2	Eukaryotic Translation Initiation Factor 4A2
1487.	IL1RAPL1	Interleukin 1 Receptor Accessory Protein Like 1
1488.	MSH3	MutS Homolog 3
1489.	DNTT	DNA Nucleotidylexotransferase
1490.	EHHADH	Enoyl-CoA Hydratase And 3-Hydroxyacyl CoA Dehydrogenase
1491.	FBL	Fibrillarin
1492.	IVD	Isovaleryl-CoA Dehydrogenase
1493.	M6PR	Mannose-6-Phosphate Receptor, Cation Dependent
1494.	MFN1	Mitofusin 1
1495.	STK39	Serine/Threonine Kinase 39
1496.	MPDU1	Mannose-P-Dolichol Utilization Defect 1
1497.	PPIG	Peptidylprolyl Isomerase G
1498.	RBMX	RNA Binding Motif Protein X-Linked
1499.	TRIP4	Thyroid Hormone Receptor Interactor 4
1500.	CD83	CD83 Molecule
1501.	CEBPD	CCAAT Enhancer Binding Protein Delta
1502.	COQ5	Coenzyme Q5, Methyltransferase
1503.	CXCL13	C-X-C Motif Chemokine Ligand 13
1504.	CALB2	Calbindin 2
1505.	GCM2	Glial Cells Missing Transcription Factor 2
1506.	GK2	Glycerol Kinase 2
1507.	LSM2	LSM2 Homolog, U6 Small Nuclear RNA And MRNA Degradation Associated
1508.	STX11	Syntaxin 11
1509.	SBF2	SET Binding Factor 2
1510.	SCARF2	Scavenger Receptor Class F Member 2
1511.	SPTBN4	Spectrin Beta, Non-Erythrocytic 4
1512.	GPAT3	Glycerol-3-Phosphate Acyltransferase 3
1513.	ARHGEF5	Rho Guanine Nucleotide Exchange Factor 5
1514.	DIP2B	Disco Interacting Protein 2 Homolog B
1515.	EGFLAM	EGF Like, Fibronectin Type III And Laminin G Domains
1516.	FAM111A	FAM111 Trypsin Like Peptidase A
1517.	LY86	Lymphocyte Antigen 86
1518.	RILPL1	Rab Interacting Lysosomal Protein Like 1

1519.	ZC4H2	Zinc Finger C4H2-Type Containing
1520.	CD99L2	CD99 Molecule Like 2
1521.	CAVIN2	Caveolae Associated Protein 2
1522.	LIPT2	Lipoyl(Octanoyl) Transferase 2
1523.	SLC35F3	Solute Carrier Family 35 Member F3
1524.	SSX2	SSX Family Member 2
1525.	CDC42SE1	CDC42 Small Effector 1
1526.	CFAP47	Cilia And Flagella Associated Protein 47
1527.	FIBIN	Fin Bud Initiation Factor Homolog
1528.	NOTCH2NLA	Notch 2 N-Terminal Like A
1529.	TMEM185A	Transmembrane Protein 185A
1530.	DUX4	Double Homeobox 4
1531.	BRAF	B-Raf Proto-Oncogene, Serine/Threonine Kinase
1532.	ALK	ALK Receptor Tyrosine Kinase
1533.	RNASEL	Ribonuclease L
1534.	GNRH1	Gonadotropin Releasing Hormone 1
1535.	RARA	Retinoic Acid Receptor Alpha
1536.	AKT3	AKT Serine/Threonine Kinase 3
1537.	CCND1	Cyclin D1
1538.	CDK4	Cyclin Dependent Kinase 4
1539.	NTRK2	Neurotrophic Receptor Tyrosine Kinase 2
1540.	FLT4	Fms Related Receptor Tyrosine Kinase 4
1541.	ITGB3	Integrin Subunit Beta 3
1542.	MYC	MYC Proto-Oncogene, BHLH Transcription Factor
1543.	NFKB1	Nuclear Factor Kappa B Subunit 1
1544.	NTRK3	Neurotrophic Receptor Tyrosine Kinase 3
1545.	PTEN	Phosphatase And Tensin Homolog
1546.	SMAD3	SMAD Family Member 3
1547.	BMPR1A	Bone Morphogenetic Protein Receptor Type 1A
1548.	CALR	Calreticulin
1549.	CDH2	Cadherin 2
1550.	GRIN2A	Glutamate Ionotropic Receptor NMDA Type Subunit 2A
1551.	LCK	LCK Proto-Oncogene, Src Family Tyrosine Kinase
1552.	PDGFB	Platelet Derived Growth Factor Subunit B
1553.	RELA	RELA Proto-Oncogene, NF-KB Subunit
1554.	SMO	Smoothened, Frizzled Class Receptor
1555.	ACVRL1	Activin A Receptor Like Type 1
1556.	BMPR2	Bone Morphogenetic Protein Receptor Type 2
1557.	DSP	Desmoplakin
1558.	EDNRB	Endothelin Receptor Type B
1559.	JAG1	Jagged Canonical Notch Ligand 1

1560.	KCNH2	Potassium Voltage-Gated Channel Subfamily H Member 2
1561.	MMP13	Matrix Metallopeptidase 13
1562.	MMP14	Matrix Metallopeptidase 14
1563.	NFKBIA	NFKB Inhibitor Alpha
1564.	RIPK1	Receptor Interacting Serine/Threonine Kinase 1
1565.	SHH	Sonic Hedgehog Signaling Molecule
1566.	SLC12A2	Solute Carrier Family 12 Member 2
1567.	UGT1A1	UDP Glucuronosyltransferase Family 1 Member A1
1568.	AXIN2	Axin 2
1569.	GATA4	GATA Binding Protein 4
1570.	GRIA1	Glutamate Ionotropic Receptor AMPA Type Subunit 1
1571.	HNF4A	Hepatocyte Nuclear Factor 4 Alpha
1572.	IGF2	Insulin Like Growth Factor 2
1573.	LRP2	LDL Receptor Related Protein 2
1574.	MAOA	Monoamine Oxidase A
1575.	MSH6	MutS Homolog 6
1576.	NRG1	Neuregulin 1
1577.	NTRK1	Neurotrophic Receptor Tyrosine Kinase 1
1578.	PMS2	PMS1 Homolog 2, Mismatch Repair System Component
1579.	SLC9A1	Solute Carrier Family 9 Member A1
1580.	ALOX5	Arachidonate 5-Lipoxygenase
1581.	ATP1A3	ATPase Na+/K+ Transporting Subunit Alpha 3
1582.	GFAP	Glial Fibrillary Acidic Protein
1583.	KCNMA1	Potassium Calcium-Activated Channel Subfamily M Alpha 1
1584.	MSH2	MutS Homolog 2
1585.	NRXN1	Neurexin 1
1586.	PKD2	Polycystin 2, Transient Receptor Potential Cation Channel
1587.	SLC1A3	Solute Carrier Family 1 Member 3
1588.	SMPD1	Sphingomyelin Phosphodiesterase 1
1589.	TOP1	DNA Topoisomerase I
1590.	TRPV4	Transient Receptor Potential Cation Channel Subfamily V Member 4
1591.	TUBG1	Tubulin Gamma 1
1592.	ACTA2	Actin Alpha 2, Smooth Muscle
1593.	ADCY5	Adenylate Cyclase 5
1594.	ALDH7A1	Aldehyde Dehydrogenase 7 Family Member A1
1595.	ANGPTL3	Angiopoietin Like 3
1596.	ARSA	Arylsulfatase A
1597.	CAPN2	Calpain 2
1598.	CYLD	CYLD Lysine 63 Deubiquitinase
1599.	EIF4G1	Eukaryotic Translation Initiation Factor 4 Gamma 1
1600.	ENO2	Enolase 2

1601.	F5	Coagulation Factor V
1602.	FUCA1	Alpha-L-Fucosidase 1
1603.	GALNS	Galactosamine (N-Acetyl)-6-Sulfatase
1604.	GUSB	Glucuronidase Beta
1605.	IDS	Iduronate 2-Sulfatase
1606.	KCNN4	Potassium Calcium-Activated Channel Subfamily N Member 4
1607.	KCNQ3	Potassium Voltage-Gated Channel Subfamily Q Member 3
1608.	LCAT	Lecithin-Cholesterol Acyltransferase
1609.	LOX	Lysyl Oxidase
1610.	LRP1	LDL Receptor Related Protein 1
1611.	MEF2C	Myocyte Enhancer Factor 2C
1612.	MTR	5-Methyltetrahydrofolate-Homocysteine Methyltransferase
1613.	NKX2-1	NK2 Homeobox 1
1614.	PAH	Phenylalanine Hydroxylase
1615.	PI4KA	Phosphatidylinositol 4-Kinase Alpha
1616.	PSAP	Prosaposin
1617.	VCL	Vinculin
1618.	YY1	YY1 Transcription Factor
1619.	ACAN	Aggrecan
1620.	ACTN4	Actinin Alpha 4
1621.	AQP2	Aquaporin 2
1622.	ARSB	Arylsulfatase B
1623.	AXIN1	Axin 1
1624.	CACNB4	Calcium Voltage-Gated Channel Auxiliary Subunit Beta 4
1625.	CDC45	Cell Division Cycle 45
1626.	COL18A1	Collagen Type XVIII Alpha 1 Chain
1627.	CTNNA1	Catenin Alpha 1
1628.	CTSA	Cathepsin A
1629.	FTL	Ferritin Light Chain
1630.	GAMT	Guanidinoacetate N-Methyltransferase
1631.	HSD17B4	Hydroxysteroid 17-Beta Dehydrogenase 4
1632.	IL7R	Interleukin 7 Receptor
1633.	ITGA7	Integrin Subunit Alpha 7
1634.	KCNA4	Potassium Voltage-Gated Channel Subfamily A Member 4
1635.	KCND3	Potassium Voltage-Gated Channel Subfamily D Member 3
1636.	MYH11	Myosin Heavy Chain 11
1637.	NPHS1	NPHS1 Adhesion Molecule, Nephrin
1638.	NR4A1	Nuclear Receptor Subfamily 4 Group A Member 1
1639.	RXRB	Retinoid X Receptor Beta
1640.	SERPING1	Serpin Family G Member 1
1641.	SLC6A2	Solute Carrier Family 6 Member 2

1642.	TMPO	Thymopoietin
1643.	TNFRSF11A	TNF Receptor Superfamily Member 11a
1644.	TNFRSF13B	TNF Receptor Superfamily Member 13B
1645.	TP63	Tumor Protein P63
1646.	TRPC3	Transient Receptor Potential Cation Channel Subfamily C Member 3
1647.	AGRN	Agrin
1648.	ALAS2	5'-Aminolevulinate Synthase 2
1649.	ALDH18A1	Aldehyde Dehydrogenase 18 Family Member A1
1650.	ALDH3A2	Aldehyde Dehydrogenase 3 Family Member A2
1651.	ARF1	ADP Ribosylation Factor 1
1652.	ARRB2	Arrestin Beta 2
1653.	BTD	Biotinidase
1654.	CALM1	Calmodulin 1
1655.	CCN2	Cellular Communication Network Factor 2
1656.	CCR4	C-C Motif Chemokine Receptor 4
1657.	CD14	CD14 Molecule
1658.	CD22	CD22 Molecule
1659.	CHD7	Chromodomain Helicase DNA Binding Protein 7
1660.	CLPP	Caseinolytic Mitochondrial Matrix Peptidase Proteolytic Subunit
1661.	COL4A3	Collagen Type IV Alpha 3 Chain
1662.	DPP6	Dipeptidyl Peptidase Like 6
1663.	EPB41	Erythrocyte Membrane Protein Band 4.1
1664.	FUT2	Fucosyltransferase 2 (H Blood Group)
1665.	GLO1	Glyoxalase I
1666.	GPT2	Glutamic--Pyruvic Transaminase 2
1667.	IDUA	Alpha-L-Iduronidase
1668.	IREB2	Iron Responsive Element Binding Protein 2
1669.	IRF8	Interferon Regulatory Factor 8
1670.	ITGAX	Integrin Subunit Alpha X
1671.	KCNC1	Potassium Voltage-Gated Channel Subfamily C Member 1
1672.	KCNC3	Potassium Voltage-Gated Channel Subfamily C Member 3
1673.	LOXL2	Lysyl Oxidase Like 2
1674.	MYL3	Myosin Light Chain 3
1675.	NDRG1	N-Myc Downstream Regulated 1
1676.	NEFL	Neurofilament Light Chain
1677.	NKX2-5	NK2 Homeobox 5
1678.	PDE4A	Phosphodiesterase 4A
1679.	PI4KB	Phosphatidylinositol 4-Kinase Beta
1680.	POLD1	DNA Polymerase Delta 1, Catalytic Subunit
1681.	RAB11A	RAB11A, Member RAS Oncogene Family
1682.	RIPK2	Receptor Interacting Serine/Threonine Kinase 2

1683.	RTN4R	Reticulon 4 Receptor
1684.	SAG	S-Antigen Visual Arrestin
1685.	SGSH	N-Sulfoglucosamine Sulfohydrolase
1686.	SKI	SKI Proto-Oncogene
1687.	SLC25A1	Solute Carrier Family 25 Member 1
1688.	SPTBN1	Spectrin Beta, Non-Erythrocytic 1
1689.	TBX5	T-Box Transcription Factor 5
1690.	TRIM24	Tripartite Motif Containing 24
1691.	ZIC3	Zic Family Member 3
1692.	ADD1	Adducin 1
1693.	AGA	Aspartylglucosaminidase
1694.	APTX	Aprataxin
1695.	BGN	Biglycan
1696.	CALM2	Calmodulin 2
1697.	CLN3	CLN3 Lysosomal/Endosomal Transmembrane Protein, Battenin
1698.	COL17A1	Collagen Type XVII Alpha 1 Chain
1699.	DCLRE1C	DNA Cross-Link Repair 1C
1700.	E2F4	E2F Transcription Factor 4
1701.	EGR2	Early Growth Response 2
1702.	EPHX1	Epoxide Hydrolase 1
1703.	FGD4	FYVE, RhoGEF And PH Domain Containing 4
1704.	GALC	Galactosylceramidase
1705.	GJA5	Gap Junction Protein Alpha 5
1706.	HCRTR2	Hypocretin Receptor 2
1707.	HELLS	Helicase, Lymphoid Specific
1708.	IGFBP2	Insulin Like Growth Factor Binding Protein 2
1709.	KLK6	Kallikrein Related Peptidase 6
1710.	KMT2D	Lysine Methyltransferase 2D
1711.	MAP3K20	Mitogen-Activated Protein Kinase Kinase Kinase 20
1712.	MTRR	5-Methyltetrahydrofolate-Homocysteine Methyltransferase Reductase
1713.	NAGA	Alpha-N-Acetylgalactosaminidase
1714.	NPPA	Natriuretic Peptide A
1715.	OCRL	OCRL Inositol Polyphosphate-5-Phosphatase
1716.	PEX1	Peroxisomal Biogenesis Factor 1
1717.	PNPO	Pyridoxamine 5'-Phosphate Oxidase
1718.	RAG1	Recombination Activating 1
1719.	SLC6A5	Solute Carrier Family 6 Member 5
1720.	SNAI1	Snail Family Transcriptional Repressor 1
1721.	SNRPN	Small Nuclear Ribonucleoprotein Polypeptide N
1722.	STXBP2	Syntaxin Binding Protein 2
1723.	TBX2	T-Box Transcription Factor 2

1724.	TNFRSF9	TNF Receptor Superfamily Member 9
1725.	TRPM6	Transient Receptor Potential Cation Channel Subfamily M Member 6
1726.	TUBA8	Tubulin Alpha 8
1727.	UGCG	UDP-Glucose Ceramide Glucosyltransferase
1728.	A4GALT	Alpha 1,4-Galactosyltransferase (P1PK Blood Group)
1729.	ADAMTS1	ADAM Metallopeptidase With Thrombospondin Type 1 Motif 1
1730.	ALDH6A1	Aldehyde Dehydrogenase 6 Family Member A1
1731.	ANP32A	Acidic Nuclear Phosphoprotein 32 Family Member A
1732.	CASP5	Caspase 5
1733.	CCR6	C-C Motif Chemokine Receptor 6
1734.	CFP	Complement Factor Properdin
1735.	COL11A2	Collagen Type XI Alpha 2 Chain
1736.	COL4A4	Collagen Type IV Alpha 4 Chain
1737.	COL4A5	Collagen Type IV Alpha 5 Chain
1738.	CRK	CRK Proto-Oncogene, Adaptor Protein
1739.	DLG1	Discs Large MAGUK Scaffold Protein 1
1740.	DPEP1	Dipeptidase 1
1741.	EBP	EBP Cholestenol Delta-Isomerase
1742.	FUT8	Fucosyltransferase 8
1743.	HMGA2	High Mobility Group AT-Hook 2
1744.	IL12RB2	Interleukin 12 Receptor Subunit Beta 2
1745.	IL18R1	Interleukin 18 Receptor 1
1746.	IL3RA	Interleukin 3 Receptor Subunit Alpha
1747.	LZTR1	Leucine Zipper Like Post Translational Regulator 1
1748.	MCOLN1	Mucolipin TRP Cation Channel 1
1749.	MKI67	Marker Of Proliferation Ki-67
1750.	MMP10	Matrix Metallopeptidase 10
1751.	MOCS2	Molybdenum Cofactor Synthesis 2
1752.	NAGLU	N-Acetyl-Alpha-Glucosaminidase
1753.	NARS1	Asparaginyl-TRNA Synthetase 1
1754.	NLGN4X	Neuroligin 4 X-Linked
1755.	NPC2	NPC Intracellular Cholesterol Transporter 2
1756.	NTHL1	Nth Like DNA Glycosylase 1
1757.	P2RX3	Purinergic Receptor P2X 3
1758.	PEX19	Peroxisomal Biogenesis Factor 19
1759.	PHOX2A	Paired Like Homeobox 2A
1760.	PLP1	Proteolipid Protein 1
1761.	RAD51C	RAD51 Paralog C
1762.	RBP3	Retinol Binding Protein 3
1763.	ROBO3	Roundabout Guidance Receptor 3
1764.	RORC	RAR Related Orphan Receptor C

1765.	STK24	Serine/Threonine Kinase 24
1766.	THY1	Thy-1 Cell Surface Antigen
1767.	TOP3A	DNA Topoisomerase III Alpha
1768.	TXK	TXK Tyrosine Kinase
1769.	ADCY7	Adenylate Cyclase 7
1770.	ADD3	Adducin 3
1771.	ARHGAP31	Rho GTPase Activating Protein 31
1772.	ATN1	Atrophin 1
1773.	C4B	Complement C4B (Chido/Rodgers Blood Group)
1774.	CDKN3	Cyclin Dependent Kinase Inhibitor 3
1775.	CYSLTR1	Cysteinyl Leukotriene Receptor 1
1776.	DNM3	Dynamin 3
1777.	DSG1	Desmoglein 1
1778.	DST	Dystonin
1779.	FBLN2	Fibulin 2
1780.	FGF14	Fibroblast Growth Factor 14
1781.	GLP2R	Glucagon Like Peptide 2 Receptor
1782.	HARS2	Histidyl-TRNA Synthetase 2, Mitochondrial
1783.	HCRTR1	Hypocretin Receptor 1
1784.	IFT81	Intraflagellar Transport 81
1785.	KLF1	KLF Transcription Factor 1
1786.	KRT7	Keratin 7
1787.	LITAF	Lipopolysaccharide Induced TNF Factor
1788.	LOXL1	Lysyl Oxidase Like 1
1789.	MYO5B	Myosin VB
1790.	NLRP2	NLR Family Pyrin Domain Containing 2
1791.	NPHS2	NPHS2 Stomatin Family Member, Podocin
1792.	PAM	Peptidylglycine Alpha-Amidating Monooxygenase
1793.	PDGFRL	Platelet Derived Growth Factor Receptor Like
1794.	PNPLA3	Patatin Like Phospholipase Domain Containing 3
1795.	PTGDR2	Prostaglandin D2 Receptor 2
1796.	RHAG	Rh Associated Glycoprotein
1797.	RNF168	Ring Finger Protein 168
1798.	RNMT	RNA Guanine-7 Methyltransferase
1799.	SLC26A5	Solute Carrier Family 26 Member 5
1800.	SLC37A4	Solute Carrier Family 37 Member 4
1801.	SOX17	SRY-Box Transcription Factor 17
1802.	SPI1	Spi-1 Proto-Oncogene
1803.	TBX20	T-Box Transcription Factor 20
1804.	TFEB	Transcription Factor EB
1805.	TPX2	TPX2 Microtubule Nucleation Factor

1806.	UGT1A6	UDP Glucuronosyltransferase Family 1 Member A6
1807.	UMOD	Uromodulin
1808.	VAC14	VAC14 Component Of PIKFYVE Complex
1809.	XYLT1	Xylosyltransferase 1
1810.	ADD2	Adducin 2
1811.	AGK	Acylglycerol Kinase
1812.	BBS10	Bardet-Biedl Syndrome 10
1813.	BBS2	Bardet-Biedl Syndrome 2
1814.	BPI	Bactericidal Permeability Increasing Protein
1815.	BSND	Barttin CLCNK Type Accessory Subunit Beta
1816.	CBX5	Chromobox 5
1817.	CEP290	Centrosomal Protein 290
1818.	CERT1	Ceramide Transporter 1
1819.	CHST14	Carbohydrate Sulfotransferase 14
1820.	CLDN11	Claudin 11
1821.	CLDN16	Claudin 16
1822.	CRB2	Crumbs Cell Polarity Complex Component 2
1823.	CRELD1	Cysteine Rich With EGF Like Domains 1
1824.	CXCR5	C-X-C Motif Chemokine Receptor 5
1825.	DGAT2	Diacylglycerol O-Acyltransferase 2
1826.	DGCR2	DiGeorge Syndrome Critical Region Gene 2
1827.	DOCK6	Dedicator Of Cytokinesis 6
1828.	FUT6	Fucosyltransferase 6
1829.	FXYD2	FXYD Domain Containing Ion Transport Regulator 2
1830.	IL16	Interleukin 16
1831.	IL18BP	Interleukin 18 Binding Protein
1832.	KLRK1	Killer Cell Lectin Like Receptor K1
1833.	LMAN2L	Lectin, Mannose Binding 2 Like
1834.	MTMR3	Myotubularin Related Protein 3
1835.	NBEAL2	Neurobeachin Like 2
1836.	NPPC	Natriuretic Peptide C
1837.	OSGEP	O-Sialoglycoprotein Endopeptidase
1838.	PNKD	PNKD Metallo-Beta-Lactamase Domain Containing
1839.	PNPLA8	Patatin Like Phospholipase Domain Containing 8
1840.	PVALB	Parvalbumin
1841.	RAB6A	RAB6A, Member RAS Oncogene Family
1842.	RANBP1	RAN Binding Protein 1
1843.	RHOBTB2	Rho Related BTB Domain Containing 2
1844.	RPS7	Ribosomal Protein S7
1845.	SAR1B	Secretion Associated Ras Related GTPase 1B
1846.	SEC23B	SEC23 Homolog B, COPII Coat Complex Component

1847.	SEPTIN9	Septin 9
1848.	SHANK3	SH3 And Multiple Ankyrin Repeat Domains 3
1849.	SLC25A32	Solute Carrier Family 25 Member 32
1850.	SNAP29	Synaptosome Associated Protein 29
1851.	TAFAZZIN	Tafazzin, Phospholipid-Lysophospholipid Transacylase
1852.	TARS2	Threonyl-TRNA Synthetase 2, Mitochondrial
1853.	TIMP2	TIMP Metallopeptidase Inhibitor 2
1854.	TRPC1	Transient Receptor Potential Cation Channel Subfamily C Member 1
1855.	TTPA	Alpha Tocopherol Transfer Protein
1856.	UBASH3B	Ubiquitin Associated And SH3 Domain Containing B
1857.	UGT1A10	UDP Glucuronosyltransferase Family 1 Member A10
1858.	UGT1A4	UDP Glucuronosyltransferase Family 1 Member A4
1859.	VPS13A	Vacuolar Protein Sorting 13 Homolog A
1860.	WNK3	WNK Lysine Deficient Protein Kinase 3
1861.	ADCY4	Adenylate Cyclase 4
1862.	ASIC3	Acid Sensing Ion Channel Subunit 3
1863.	BAZ1B	Bromodomain Adjacent To Zinc Finger Domain 1B
1864.	ERAP2	Endoplasmic Reticulum Aminopeptidase 2
1865.	GBA2	Glucosylceramidase Beta 2
1866.	IFT172	Intraflagellar Transport 172
1867.	KCNAB2	Potassium Voltage-Gated Channel Subfamily A Regulatory Beta Subunit 2
1868.	MAGED2	MAGE Family Member D2
1869.	P2RX6	Purinergic Receptor P2X 6
1870.	PRX	Periaxin
1871.	RAB1A	RAB1A, Member RAS Oncogene Family
1872.	RAB33B	RAB33B, Member RAS Oncogene Family
1873.	RAB8A	RAB8A, Member RAS Oncogene Family
1874.	RAD51D	RAD51 Paralog D
1875.	REEP1	Receptor Accessory Protein 1
1876.	RFX1	Regulatory Factor X1
1877.	RPS6KA6	Ribosomal Protein S6 Kinase A6
1878.	STX5	Syntaxin 5
1879.	TOMM40	Translocase Of Outer Mitochondrial Membrane 40
1880.	TRIM63	Tripartite Motif Containing 63
1881.	YBX1	Y-Box Binding Protein 1
1882.	ANKLE2	Ankyrin Repeat And LEM Domain Containing 2
1883.	BBS1	Bardet-Biedl Syndrome 1
1884.	BBS7	Bardet-Biedl Syndrome 7
1885.	CAPZB	Capping Actin Protein Of Muscle Z-Line Subunit Beta
1886.	CCRL2	C-C Motif Chemokine Receptor Like 2
1887.	CLC	Charcot-Leyden Crystal Galectin

1888.	CLCNKA	Chloride Voltage-Gated Channel Ka
1889.	CLTCL1	Clathrin Heavy Chain Like 1
1890.	CRYGC	Crystallin Gamma C
1891.	F13B	Coagulation Factor XIII B Chain
1892.	FIS1	Fission, Mitochondrial 1
1893.	GAN	Gigaxonin
1894.	GJD2	Gap Junction Protein Delta 2
1895.	GPR161	G Protein-Coupled Receptor 161
1896.	GTF2I	General Transcription Factor IIi
1897.	HBG1	Hemoglobin Subunit Gamma 1
1898.	HCRT	Hypocretin Neuropeptide Precursor
1899.	IFT27	Intraflagellar Transport 27
1900.	IL15RA	Interleukin 15 Receptor Subunit Alpha
1901.	JPH1	Junctophilin 1
1902.	KCNAB1	Potassium Voltage-Gated Channel Subfamily A Regulatory Beta Subunit 1
1903.	KLB	Klotho Beta
1904.	KMT2B	Lysine Methyltransferase 2B
1905.	LRSAM1	Leucine Rich Repeat And Sterile Alpha Motif Containing 1
1906.	MAPK8IP3	Mitogen-Activated Protein Kinase 8 Interacting Protein 3
1907.	MED15	Mediator Complex Subunit 15
1908.	MYF5	Myogenic Factor 5
1909.	MYOG	Myogenin
1910.	NUCB1	Nucleobindin 1
1911.	OGA	O-GlcNAcase
1912.	OXSR1	Oxidative Stress Responsive Kinase 1
1913.	PDIA2	Protein Disulfide Isomerase Family A Member 2
1914.	PGK2	Phosphoglycerate Kinase 2
1915.	PSIP1	PC4 And SRSF1 Interacting Protein 1
1916.	QRSL1	Glutaminyl-TRNA Amidotransferase Subunit QRSL1
1917.	RAB35	RAB35, Member RAS Oncogene Family
1918.	RFK	Riboflavin Kinase
1919.	RFX2	Regulatory Factor X2
1920.	RFX3	Regulatory Factor X3
1921.	RPS9	Ribosomal Protein S9
1922.	SCRIB	Scribble Planar Cell Polarity Protein
1923.	SLC4A5	Solute Carrier Family 4 Member 5
1924.	SNRPA	Small Nuclear Ribonucleoprotein Polypeptide A
1925.	SUN1	Sad1 And UNC84 Domain Containing 1
1926.	TMSB4X	Thymosin Beta 4 X-Linked
1927.	UGT1A7	UDP Glucuronosyltransferase Family 1 Member A7
1928.	ZFPM2	Zinc Finger Protein, FOG Family Member 2

1929.	ACBD3	Acyl-CoA Binding Domain Containing 3
1930.	AFAP1	Actin Filament Associated Protein 1
1931.	AIFM3	Apoptosis Inducing Factor Mitochondria Associated 3
1932.	ANKRD11	Ankyrin Repeat Domain Containing 11
1933.	BLOC1S6	Biogenesis Of Lysosomal Organelles Complex 1 Subunit 6
1934.	CAPRIN1	Cell Cycle Associated Protein 1
1935.	CD177	CD177 Molecule
1936.	CD52	CD52 Molecule
1937.	CDAN1	Codanin 1
1938.	CNN1	Calponin 1
1939.	COG5	Component Of Oligomeric Golgi Complex 5
1940.	CPSF4	Cleavage And Polyadenylation Specific Factor 4
1941.	DNAJA2	DnaJ Heat Shock Protein Family (Hsp40) Member A2
1942.	DOK7	Docking Protein 7
1943.	FHL3	Four And A Half LIM Domains 3
1944.	GYG2	Glycogenin 2
1945.	HSD17B13	Hydroxysteroid 17-Beta Dehydrogenase 13
1946.	IFT74	Intraflagellar Transport 74
1947.	IL32	Interleukin 32
1948.	ILF2	Interleukin Enhancer Binding Factor 2
1949.	KCNA6	Potassium Voltage-Gated Channel Subfamily A Member 6
1950.	KCNJ16	Potassium Inwardly Rectifying Channel Subfamily J Member 16
1951.	KLRG1	Killer Cell Lectin Like Receptor G1
1952.	KRT12	Keratin 12
1953.	KRT3	Keratin 3
1954.	KRT86	Keratin 86
1955.	LGI2	Leucine Rich Repeat LGI Family Member 2
1956.	MBOAT7	Membrane Bound O-Acyltransferase Domain Containing 7
1957.	MFF	Mitochondrial Fission Factor
1958.	MICA	MHC Class I Polypeptide-Related Sequence A
1959.	MYOM1	Myomesin 1
1960.	NPTXR	Neuronal Pentraxin Receptor
1961.	PIGV	Phosphatidylinositol Glycan Anchor Biosynthesis Class V
1962.	PLPBP	Pyridoxal Phosphate Binding Protein
1963.	PPL	Periplakin
1964.	RPL27A	Ribosomal Protein L27a
1965.	SAA4	Serum Amyloid A4, Constitutive
1966.	SLC29A4	Solute Carrier Family 29 Member 4
1967.	TFDP2	Transcription Factor Dp-2
1968.	THSD4	Thrombospondin Type 1 Domain Containing 4
1969.	TMOD1	Tropomodulin 1

1970.	TMOD2	Tropomodulin 2
1971.	TOP3B	DNA Topoisomerase III Beta
1972.	UFSP2	UFM1 Specific Peptidase 2
1973.	ZC3H12A	Zinc Finger CCCH-Type Containing 12A
1974.	ZDHHC8	Zinc Finger DHHC-Type Palmitoyltransferase 8
1975.	ANP32B	Acidic Nuclear Phosphoprotein 32 Family Member B
1976.	COQ3	Coenzyme Q3, Methyltransferase
1977.	DHX29	DExH-Box Helicase 29
1978.	EOGT	EGF Domain Specific O-Linked N-Acetylglucosamine Transferase
1979.	ERAL1	Era Like 12S Mitochondrial RRNA Chaperone 1
1980.	FAU	FAU Ubiquitin Like And Ribosomal Protein S30 Fusion
1981.	FBN3	Fibrillin 3
1982.	GALNT16	Polypeptide N-Acetylgalactosaminyltransferase 16
1983.	GNG3	G Protein Subunit Gamma 3
1984.	GNLY	Granulysin
1985.	GZMH	Granzyme H
1986.	GZMM	Granzyme M
1987.	HLA-DQA2	Major Histocompatibility Complex, Class II, DQ Alpha 2
1988.	IL1F10	Interleukin 1 Family Member 10
1989.	KCNA10	Potassium Voltage-Gated Channel Subfamily A Member 10
1990.	KCTD13	Potassium Channel Tetramerization Domain Containing 13
1991.	KDELR1	KDEL Endoplasmic Reticulum Protein Retention Receptor 1
1992.	KLHL22	Kelch Like Family Member 22
1993.	KPNA6	Karyopherin Subunit Alpha 6
1994.	MYO18B	Myosin XVIIIB
1995.	NDFIP1	Nedd4 Family Interacting Protein 1
1996.	NLRC5	NLR Family CARD Domain Containing 5
1997.	NSFL1C	NSFL1 Cofactor
1998.	OAS2	2'-5'-Oligoadenylate Synthetase 2
1999.	ORAI2	ORAI Calcium Release-Activated Calcium Modulator 2
2000.	POP1	POP1 Homolog, Ribonuclease P/MRP Subunit
2001.	PSMG1	Proteasome Assembly Chaperone 1
2002.	SEPTIN5	Septin 5
2003.	SHARPIN	SHANK Associated RH Domain Interactor
2004.	SLC52A2	Solute Carrier Family 52 Member 2
2005.	SMYD1	SET And MYND Domain Containing 1
2006.	SNAPIN	SNAP Associated Protein
2007.	SUMO4	Small Ubiquitin Like Modifier 4
2008.	TBC1D5	TBC1 Domain Family Member 5
2009.	THSD7A	Thrombospondin Type 1 Domain Containing 7A
2010.	TMOD3	Tropomodulin 3

2011.	TRIM39	Tripartite Motif Containing 39
2012.	TRMT2A	TRNA Methyltransferase 2 Homolog A
2013.	UGT1A8	UDP Glucuronosyltransferase Family 1 Member A8
2014.	UQCC2	Ubiquinol-Cytochrome C Reductase Complex Assembly Factor 2
2015.	VPS39	VPS39 Subunit Of HOPS Complex
2016.	ZDHHC5	Zinc Finger DHHC-Type Palmitoyltransferase 5
2017.	ZNF74	Zinc Finger Protein 74
2018.	DCHS1	Dachsous Cadherin-Related 1
2019.	DNAAF2	Dynein Axonemal Assembly Factor 2
2020.	EDC4	Enhancer Of MRNA Decapping 4
2021.	EVPL	Envoplakin
2022.	GNB1L	G Protein Subunit Beta 1 Like
2023.	GPAT4	Glycerol-3-Phosphate Acyltransferase 4
2024.	IL26	Interleukin 26
2025.	KLHL41	Kelch Like Family Member 41
2026.	KLHL9	Kelch Like Family Member 9
2027.	LACC1	Laccase Domain Containing 1
2028.	MMP26	Matrix Metallopeptidase 26
2029.	RASA3	RAS P21 Protein Activator 3
2030.	RPP30	Ribonuclease P/MRP Subunit P30
2031.	SULT1A3	Sulfotransferase Family 1A Member 3
2032.	TIMM22	Translocase Of Inner Mitochondrial Membrane 22
2033.	TSSK2	Testis Specific Serine Kinase 2
2034.	VCPIP1	Valosin Containing Protein Interacting Protein 1
2035.	YIF1B	Yip1 Interacting Factor Homolog B, Membrane Trafficking Protein
2036.	ZDHHC15	Zinc Finger DHHC-Type Palmitoyltransferase 15
2037.	ZNF804A	Zinc Finger Protein 804A
2038.	ANKRD49	Ankyrin Repeat Domain 49
2039.	BRWD3	Bromodomain And WD Repeat Domain Containing 3
2040.	CDIN1	CDAN1 Interacting Nuclease 1
2041.	CEP192	Centrosomal Protein 192
2042.	CKAP2	Cytoskeleton Associated Protein 2
2043.	CPXM1	Carboxypeptidase X, M14 Family Member 1
2044.	DGCR6	DiGeorge Syndrome Critical Region Gene 6
2045.	ESYT1	Extended Synaptotagmin 1
2046.	EXOSC4	Exosome Component 4
2047.	FOXE3	Forkhead Box E3
2048.	GIMAP2	GTPase, IMAP Family Member 2
2049.	GIMAP4	GTPase, IMAP Family Member 4
2050.	HOXC9	Homeobox C9
2051.	ICAM4	Intercellular Adhesion Molecule 4 (Landsteiner-Wiener Blood Group)

2052.	IFT25	Intraflagellar Transport 25
2053.	KIR2DL1	Killer Cell Immunoglobulin Like Receptor, Two Ig Domains And Long Cytoplasmic Tail 1
2054.	LAS1L	LAS1 Like Ribosome Biogenesis Factor
2055.	LILRB3	Leukocyte Immunoglobulin Like Receptor B3
2056.	LIN9	Lin-9 DREAM MuvB Core Complex Component
2057.	MAMLD1	Mastermind Like Domain Containing 1
2058.	MIEF1	Mitochondrial Elongation Factor 1
2059.	MRPL40	Mitochondrial Ribosomal Protein L40
2060.	NADK2	NAD Kinase 2, Mitochondrial
2061.	OMP	Olfactory Marker Protein
2062.	PEX11A	Peroxisomal Biogenesis Factor 11 Alpha
2063.	PGM5	Phosphoglucomutase 5
2064.	PNMA2	PNMA Family Member 2
2065.	POLR2H	RNA Polymerase II, I And III Subunit H
2066.	PTBP3	Polypyrimidine Tract Binding Protein 3
2067.	RABIF	RAB Interacting Factor
2068.	SNRPC	Small Nuclear Ribonucleoprotein Polypeptide C
2069.	TOMM70	Translocase Of Outer Mitochondrial Membrane 70
2070.	BAHD1	Bromo Adjacent Homology Domain Containing 1
2071.	CEP19	Centrosomal Protein 19
2072.	CEP89	Centrosomal Protein 89
2073.	DRAXIN	Dorsal Inhibitory Axon Guidance Protein
2074.	FITM2	Fat Storage Inducing Transmembrane Protein 2
2075.	GON4L	Gon-4 Like
2076.	IFNA4	Interferon Alpha 4
2077.	IGHM	Immunoglobulin Heavy Constant Mu
2078.	NDFIP2	Nedd4 Family Interacting Protein 2
2079.	PAXBP1	PAX3 And PAX7 Binding Protein 1
2080.	PMCH	Pro-Melanin Concentrating Hormone
2081.	RNF112	Ring Finger Protein 112
2082.	SGTB	Small Glutamine Rich Tetratricopeptide Repeat Co-Chaperone Beta
2083.	SLFN14	Schlafen Family Member 14
2084.	SPCS1	Signal Peptidase Complex Subunit 1
2085.	SPTBN5	Spectrin Beta, Non-Erythrocytic 5
2086.	TCFL5	Transcription Factor Like 5
2087.	TEFM	Transcription Elongation Factor, Mitochondrial
2088.	ZNHIT3	Zinc Finger HIT-Type Containing 3
2089.	ZUP1	Zinc Finger Containing Ubiquitin Peptidase 1
2090.	DGCR6L	DiGeorge Syndrome Critical Region Gene 6 Like
2091.	DNAJC17	DnaJ Heat Shock Protein Family (Hsp40) Member C17
2092.	LGALSL	Galectin Like

2093.	NLRP11	NLR Family Pyrin Domain Containing 11
2094.	PTGR3	Prostaglandin Reductase 3
2095.	ZNF7	Zinc Finger Protein 7
2096.	AMN1	Antagonist Of Mitotic Exit Network 1 Homolog
2097.	ANKLE1	Ankyrin Repeat And LEM Domain Containing 1
2098.	COL24A1	Collagen Type XXIV Alpha 1 Chain
2099.	DCAF5	DDB1 And CUL4 Associated Factor 5
2100.	DTWD2	DTW Domain Containing 2
2101.	FERRY3	FERRY Endosomal RAB5 Effector Complex Subunit 3
2102.	IFI44L	Interferon Induced Protein 44 Like
2103.	LMLN	Leishmanolysin Like Peptidase
2104.	MOGAT1	Monoacylglycerol O-Acyltransferase 1
2105.	MRPS21	Mitochondrial Ribosomal Protein S21
2106.	MZB1	Marginal Zone B And B1 Cell Specific Protein
2107.	NIM1K	NIM1 Serine/Threonine Protein Kinase
2108.	OR1F1	Olfactory Receptor Family 1 Subfamily F Member 1
2109.	ORAI3	ORAI Calcium Release-Activated Calcium Modulator 3
2110.	PEX11G	Peroxisomal Biogenesis Factor 11 Gamma
2111.	PHAX	Phosphorylated Adaptor For RNA Export
2112.	PSMB11	Proteasome Subunit Beta 11
2113.	RABL2A	RAB, Member Of RAS Oncogene Family Like 2A
2114.	RNF39	Ring Finger Protein 39
2115.	SARAF	Store-Operated Calcium Entry Associated Regulatory Factor
2116.	SPATA2	Spermatogenesis Associated 2
2117.	TARS3	Threonyl-TRNA Synthetase 3
2118.	THAP7	THAP Domain Containing 7
2119.	TMEM260	Transmembrane Protein 260
2120.	TNMD	Tenomodulin
2121.	TOMM22	Translocase Of Outer Mitochondrial Membrane 22
2122.	ZNF200	Zinc Finger Protein 200
2123.	BLOC1S4	Biogenesis Of Lysosomal Organelles Complex 1 Subunit 4
2124.	CCDC180	Coiled-Coil Domain Containing 180
2125.	CNIH1	Cornichon Family AMPA Receptor Auxiliary Protein 1
2126.	COMMD3	COMM Domain Containing 3
2127.	CRACR2A	Calcium Release Activated Channel Regulator 2A
2128.	FAM149B1	Family With Sequence Similarity 149 Member B1
2129.	INTS2	Integrator Complex Subunit 2
2130.	LILRA6	Leukocyte Immunoglobulin Like Receptor A6
2131.	LRTOMT	Leucine Rich Transmembrane And O-Methyltransferase Domain Containing
2132.	NICN1	Nicolin 1, Tubulin Polyglutamylase Complex Subunit
2133.	OTULINL	OTU Deubiquitinase With Linear Linkage Specificity Like

2134.	PRR12	Proline Rich 12
2135.	RBFA	Ribosome Binding Factor A
2136.	SH3TC1	SH3 Domain And Tetratricopeptide Repeats 1
2137.	SLC66A2	Solute Carrier Family 66 Member 2
2138.	SMCO4	Single-Pass Membrane Protein With Coiled-Coil Domains 4
2139.	SPAG8	Sperm Associated Antigen 8
2140.	TBL1Y	Transducin Beta Like 1 Y-Linked
2141.	AKIRIN1	Akirin 1
2142.	AMIGO3	Adhesion Molecule With Ig Like Domain 3
2143.	GOLGA7B	Golgin A7 Family Member B
2144.	GRAMD2B	GRAM Domain Containing 2B
2145.	KLHDC10	Kelch Domain Containing 10
2146.	LRRC66	Leucine Rich Repeat Containing 66
2147.	MTNAP1	Mitochondrial Nucleoid Associated Protein 1
2148.	OR10J1	Olfactory Receptor Family 10 Subfamily J Member 1
2149.	PSMG4	Proteasome Assembly Chaperone 4
2150.	PYDC1	Pyrin Domain Containing 1
2151.	RADX	RPA1 Related Single Stranded DNA Binding Protein, X-Linked
2152.	RAVER2	Ribonucleoprotein, PTB Binding 2
2153.	SHISA2	Shisa Family Member 2
2154.	TM6SF2	Transmembrane 6 Superfamily Member 2
2155.	TMEM26	Transmembrane Protein 26
2156.	URB1	URB1 Ribosome Biogenesis Homolog
2157.	ZNF546	Zinc Finger Protein 546
2158.	CNPY1	Canopy FGF Signaling Regulator 1
2159.	COPS9	COP9 Signalosome Subunit 9
2160.	DIPK1C	Divergent Protein Kinase Domain 1C
2161.	GLIPR1L2	GLIPR1 Like 2
2162.	LRRC58	Leucine Rich Repeat Containing 58
2163.	NNAT	Neuronatin
2164.	C1orf141	Chromosome 1 Open Reading Frame 141
2165.	CDHR4	Cadherin Related Family Member 4
2166.	CIMAP3	Ciliary Microtubule Associated Protein 3
2167.	CNBD2	Cyclic Nucleotide Binding Domain Containing 2
2168.	ETFRF1	Electron Transfer Flavoprotein Regulatory Factor 1
2169.	HSBP1L1	Heat Shock Factor Binding Protein 1 Like 1
2170.	LDAF1	Lipid Droplet Assembly Factor 1
2171.	MARCHF3	Membrane Associated Ring-CH-Type Finger 3
2172.	RAD21L1	RAD21 Cohesin Complex Component Like 1
2173.	TRIM52	Tripartite Motif Containing 52
2174.	C12orf75	Chromosome 12 Open Reading Frame 75

2175.	CFAP73	Cilia And Flagella Associated Protein 73
2176.	GAB4	GRB2 Associated Binding Protein Family Member 4
2177.	KLHL33	Kelch Like Family Member 33
2178.	MINAR2	Membrane Integral NOTCH2 Associated Receptor 2
2179.	MYMK	Myomaker, Myoblast Fusion Factor
2180.	PLGLB2	Plasminogen Like B2
2181.	TEX38	Testis Expressed 38
2182.	SCGB1D1	Secretoglobin Family 1D Member 1
2183.	C18orf63	Chromosome 18 Open Reading Frame 63
2184.	C22orf15	Chromosome 22 Open Reading Frame 15
2185.	OR2T34	Olfactory Receptor Family 2 Subfamily T Member 34
2186.	SMIM21	Small Integral Membrane Protein 21
2187.	ZFTRAF1	Zinc Finger TRAF-Type Containing 1
2188.	FREY1	Frey Regulator Of Sperm-Oocyte Fusion 1
2189.	RIMBP3C	RIMS Binding Protein 3C
2190.	SPMIP10	Sperm Microtubule Inner Protein 10
2191.	STIMATE	STIM Activating Enhancer
2192.	C5orf63	Chromosome 5 Open Reading Frame 63
2193.	LRRC74B	Leucine Rich Repeat Containing 74B
2194.	MEIKIN	Meiotic Kinetochore Factor
2195.	TDRD15	Tudor Domain Containing 15
2196.	TRIM75	Tripartite Motif Containing 75
2197.	TRIM39-RPP21	TRIM39-RPP21 Readthrough
2198.	SCART1	Scavenger Receptor Family Member Expressed On T Cells 1
2199.	PMIS2	PMIS2 Transmembrane Protein
2200.	HSPA1A	Heat Shock Protein Family A (Hsp70) Member 1A
2201.	EFNB1	Ephrin B1
2202.	EPHB1	EPH Receptor B1
2203.	CMPK1	Cytidine/Uridine Monophosphate Kinase 1
2204.	MET	MET Proto-Oncogene, Receptor Tyrosine Kinase
2205.	ROR2	Receptor Tyrosine Kinase Like Orphan Receptor 2
2206.	ALDH5A1	Aldehyde Dehydrogenase 5 Family Member A1
2207.	ATP2B3	ATPase Plasma Membrane Ca2+ Transporting 3
2208.	KIF5B	Kinesin Family Member 5B
2209.	FZD3	Frizzled Class Receptor 3
2210.	HINT1	Histidine Triad Nucleotide Binding Protein 1
2211.	DVL2	Dishevelled Segment Polarity Protein 2
2212.	SEPSECS	Sep (O-Phosphoserine) TRNA:Sec (Selenocysteine) TRNA Synthase
2213.	CHRNG	Cholinergic Receptor Nicotinic Gamma Subunit
2214.	MYL1	Myosin Light Chain 1
2215.	CEBPE	CCAAT Enhancer Binding Protein Epsilon

2216.	ZBTB20	Zinc Finger And BTB Domain Containing 20
2217.	CEP63	Centrosomal Protein 63
2218.	GLDN	Gliomedin
2219.	KLHL7	Kelch Like Family Member 7
2220.	LGI4	Leucine Rich Repeat LGI Family Member 4
2221.	USH2A	Usherin
2222.	ASPM	Assembly Factor For Spindle Microtubules
2223.	ERGIC1	Endoplasmic Reticulum-Golgi Intermediate Compartment 1
2224.	MYL11	Myosin Light Chain 11
2225.	DOCK11	Dedicator Of Cytokinesis 11
2226.	TOR1AIP2	Torsin 1A Interacting Protein 2
2227.	CAT	Catalase
2228.	ITGA2B	Integrin Subunit Alpha 2b
2229.	MAPK14	Mitogen-Activated Protein Kinase 14
2230.	PLAU	Plasminogen Activator, Urokinase
2231.	PPP3CA	Protein Phosphatase 3 Catalytic Subunit Alpha
2232.	ADK	Adenosine Kinase
2233.	PHGDH	Phosphoglycerate Dehydrogenase
2234.	ARG1	Arginase 1
2235.	HSPA8	Heat Shock Protein Family A (Hsp70) Member 8
2236.	IFNAR2	Interferon Alpha And Beta Receptor Subunit 2
2237.	VDR	Vitamin D Receptor
2238.	CD46	CD46 Molecule
2239.	FKBP1A	FKBP Prolyl Isomerase 1A
2240.	GP1BA	Glycoprotein Ib Platelet Subunit Alpha
2241.	IMPDH2	Inosine Monophosphate Dehydrogenase 2
2242.	PARK7	Parkinsonism Associated Deglycase
2243.	SIRT3	Sirtuin 3
2244.	ACO2	Aconitase 2
2245.	CTSS	Cathepsin S
2246.	IRAK3	Interleukin 1 Receptor Associated Kinase 3
2247.	NPR1	Natriuretic Peptide Receptor 1
2248.	SERPINH1	Serpin Family H Member 1
2249.	ADAR	Adenosine Deaminase RNA Specific
2250.	ADH5	Alcohol Dehydrogenase 5 (Class III), Chi Polypeptide
2251.	ADM	Adrenomedullin
2252.	ATG5	Autophagy Related 5
2253.	IKBKE	Inhibitor Of Nuclear Factor Kappa B Kinase Subunit Epsilon
2254.	MASP2	MBL Associated Serine Protease 2
2255.	MC2R	Melanocortin 2 Receptor
2256.	PSPH	Phosphoserine Phosphatase

2257.	SERPINF2	Serpin Family F Member 2
2258.	ABCA4	ATP Binding Cassette Subfamily A Member 4
2259.	ALOX12	Arachidonate 12-Lipoxygenase, 12S Type
2260.	GPD1	Glycerol-3-Phosphate Dehydrogenase 1
2261.	NT5C3A	5'-Nucleotidase, Cytosolic IIIA
2262.	PKP2	Plakophilin 2
2263.	SELPLG	Selectin P Ligand
2264.	SFTPA1	Surfactant Protein A1
2265.	UBC	Ubiquitin C
2266.	ACO1	Aconitase 1
2267.	CD63	CD63 Molecule
2268.	FABP3	Fatty Acid Binding Protein 3
2269.	LBP	Lipopolysaccharide Binding Protein
2270.	SAMHD1	SAM And HD Domain Containing Deoxynucleoside Triphosphate Triphosphohydrolase 1
2271.	SFTPB	Surfactant Protein B
2272.	SLC29A3	Solute Carrier Family 29 Member 3
2273.	UQCRC1	Ubiquinol-Cytochrome C Reductase Core Protein 1
2274.	DBT	Dihydrolipoamide Branched Chain Transacylase E2
2275.	AAK1	AP2 Associated Kinase 1
2276.	CD164	CD164 Molecule
2277.	SCAP	SREBF Chaperone
2278.	SPRY1	Sprouty RTK Signaling Antagonist 1
2279.	NDUFAB1	NADH:Ubiquinone Oxidoreductase Subunit AB1
2280.	PPIC	Peptidylprolyl Isomerase C
2281.	ACR	Acrosin
2282.	ALPK1	Alpha Kinase 1
2283.	ATXN2L	Ataxin 2 Like
2284.	CAPZA1	Capping Actin Protein Of Muscle Z-Line Subunit Alpha 1
2285.	CXCL2	C-X-C Motif Chemokine Ligand 2
2286.	CXCL5	C-X-C Motif Chemokine Ligand 5
2287.	ETF1	Eukaryotic Translation Termination Factor 1
2288.	HAP1	Huntingtin Associated Protein 1
2289.	MPP1	MAGUK P55 Scaffold Protein 1
2290.	NBR1	NBR1 Autophagy Cargo Receptor
2291.	NDUFA5	NADH:Ubiquinone Oxidoreductase Subunit A5
2292.	PPIL1	Peptidylprolyl Isomerase Like 1
2293.	PSMC6	Proteasome 26S Subunit, ATPase 6
2294.	RCVRN	Recoverin
2295.	RIT2	Ras Like Without CAAX 2
2296.	RNF220	Ring Finger Protein 220
2297.	TBL1X	Transducin Beta Like 1 X-Linked

2298.	CAPZA2	Capping Actin Protein Of Muscle Z-Line Subunit Alpha 2
2299.	CARD8	Caspase Recruitment Domain Family Member 8
2300.	DEFB1	Defensin Beta 1
2301.	FKBP1B	FKBP Prolyl Isomerase 1B
2302.	FUT7	Fucosyltransferase 7
2303.	KCNIP1	Potassium Voltage-Gated Channel Interacting Protein 1
2304.	NDUFS5	NADH:Ubiquinone Oxidoreductase Subunit S5
2305.	NDUFV3	NADH:Ubiquinone Oxidoreductase Subunit V3
2306.	NFYC	Nuclear Transcription Factor Y Subunit Gamma
2307.	RAB5C	RAB5C, Member RAS Oncogene Family
2308.	RPS11	Ribosomal Protein S11
2309.	SRA1	Steroid Receptor RNA Activator 1
2310.	XPO5	Exportin 5
2311.	APBB1IP	Amyloid Beta Precursor Protein Binding Family B Member 1 Interacting Protein
2312.	CNMD	Chondromodulin
2313.	CXCL6	C-X-C Motif Chemokine Ligand 6
2314.	DNASE1L1	Deoxyribonuclease 1 Like 1
2315.	ERC2	ELKS/RAB6-Interacting/CAST Family Member 2
2316.	HYCC1	Hyccin PI4KA Lipid Kinase Complex Subunit 1
2317.	LARGE2	LARGE Xylosyl- And Glucuronyltransferase 2
2318.	NDUFB6	NADH:Ubiquinone Oxidoreductase Subunit B6
2319.	NEBL	Nebulette
2320.	NSUN5	NOP2/Sun RNA Methyltransferase 5
2321.	PATZ1	POZ/BTB And AT Hook Containing Zinc Finger 1
2322.	RASD1	Ras Related Dexamethasone Induced 1
2323.	RPL18A	Ribosomal Protein L18a
2324.	SPON2	Spondin 2
2325.	CCL18	C-C Motif Chemokine Ligand 18
2326.	CTRB1	Chymotrypsinogen B1
2327.	HSPB6	Heat Shock Protein Family B (Small) Member 6
2328.	IL27	Interleukin 27
2329.	NFYB	Nuclear Transcription Factor Y Subunit Beta
2330.	RPL36	Ribosomal Protein L36
2331.	RPS21	Ribosomal Protein S21
2332.	SAP30BP	SAP30 Binding Protein
2333.	SLC38A3	Solute Carrier Family 38 Member 3
2334.	ANAPC13	Anaphase Promoting Complex Subunit 13
2335.	BZW1	Basic Leucine Zipper And W2 Domains 1
2336.	CLEC4D	C-Type Lectin Domain Family 4 Member D
2337.	EPDR1	Ependymin Related 1
2338.	PIK3AP1	Phosphoinositide-3-Kinase Adaptor Protein 1

2339.	RBM20	RNA Binding Motif Protein 20
2340.	SPCS3	Signal Peptidase Complex Subunit 3
2341.	TMOD4	Tropomodulin 4
2342.	TXNRD3	Thioredoxin Reductase 3
2343.	GET4	Guided Entry Of Tail-Anchored Proteins Factor 4
2344.	MFSD6	Major Facilitator Superfamily Domain Containing 6
2345.	PRDM13	PR/SET Domain 13
2346.	SLC35F6	Solute Carrier Family 35 Member F6
2347.	TMEM41B	Transmembrane Protein 41B
2348.	SH3D19	SH3 Domain Containing 19
2349.	ZC3H12D	Zinc Finger CCCH-Type Containing 12D
2350.	ANKRD44	Ankyrin Repeat Domain 44
2351.	AP5M1	Adaptor Related Protein Complex 5 Subunit Mu 1
2352.	CCDC120	Coiled-Coil Domain Containing 120
2353.	CTRB2	Chymotrypsinogen B2
2354.	FABP12	Fatty Acid Binding Protein 12
2355.	FAM53B	Family With Sequence Similarity 53 Member B
2356.	MOSPD3	Motile Sperm Domain Containing 3
2357.	NUDT10	Nudix Hydrolase 10
2358.	REPIN1	Replication Initiator 1
2359.	TAAR6	Trace Amine Associated Receptor 6
2360.	DALRD3	DALR Anticodon Binding Domain Containing 3
2361.	GPATCH2L	G-Patch Domain Containing 2 Like
2362.	MROH7	Maestro Heat Like Repeat Family Member 7
2363.	MRPL36	Mitochondrial Ribosomal Protein L36
2364.	OARD1	O-Acyl-ADP-Ribose Deacylase 1
2365.	CENPV	Centromere Protein V
2366.	CHCHD5	Coiled-Coil-Helix-Coiled-Coil-Helix Domain Containing 5
2367.	OAZ3	Ornithine Decarboxylase Antizyme 3
2368.	SPAG7	Sperm Associated Antigen 7
2369.	STH	Saitohin
2370.	SYT10	Synaptotagmin 10
2371.	TMEM41A	Transmembrane Protein 41A
2372.	ASPDH	Aspartate Dehydrogenase Domain Containing
2373.	TMEM64	Transmembrane Protein 64
2374.	OR4C16	Olfactory Receptor Family 4 Subfamily C Member 16
2375.	OR51I1	Olfactory Receptor Family 51 Subfamily I Member 1
2376.	MGAM2	Maltase-Glucoamylase 2 (Putative)
2377.	PRKAA2	Protein Kinase AMP-Activated Catalytic Subunit Alpha 2
2378.	HTR1D	5-Hydroxytryptamine Receptor 1D
2379.	PDE5A	Phosphodiesterase 5A

2380.	DBH	Dopamine Beta-Hydroxylase
2381.	LDLR	Low Density Lipoprotein Receptor
2382.	PIK3R1	Phosphoinositide-3-Kinase Regulatory Subunit 1
2383.	DDC	Dopa Decarboxylase
2384.	GSK3B	Glycogen Synthase Kinase 3 Beta
2385.	FASN	Fatty Acid Synthase
2386.	PIK3C3	Phosphatidylinositol 3-Kinase Catalytic Subunit Type 3
2387.	RARB	Retinoic Acid Receptor Beta
2388.	CHAT	Choline O-Acetyltransferase
2389.	HIF1A	Hypoxia Inducible Factor 1 Subunit Alpha
2390.	RXRA	Retinoid X Receptor Alpha
2391.	ADORA1	Adenosine A1 Receptor
2392.	GABRA2	Gamma-Aminobutyric Acid Type A Receptor Subunit Alpha2
2393.	IRS1	Insulin Receptor Substrate 1
2394.	OAT	Ornithine Aminotransferase
2395.	PCK1	Phosphoenolpyruvate Carboxykinase 1
2396.	CYP2E1	Cytochrome P450 Family 2 Subfamily E Member 1
2397.	GNB3	G Protein Subunit Beta 3
2398.	HTR7	5-Hydroxytryptamine Receptor 7
2399.	NOTCH4	Notch Receptor 4
2400.	PRKAA1	Protein Kinase AMP-Activated Catalytic Subunit Alpha 1
2401.	UCP2	Uncoupling Protein 2
2402.	ABCG5	ATP Binding Cassette Subfamily G Member 5
2403.	APOC3	Apolipoprotein C3
2404.	CCKAR	Cholecystokinin A Receptor
2405.	CHRM1	Cholinergic Receptor Muscarinic 1
2406.	CYP1A2	Cytochrome P450 Family 1 Subfamily A Member 2
2407.	DRD4	Dopamine Receptor D4
2408.	GABRA4	Gamma-Aminobutyric Acid Type A Receptor Subunit Alpha4
2409.	GNAO1	G Protein Subunit Alpha O1
2410.	LIPC	Lipase C, Hepatic Type
2411.	RARG	Retinoic Acid Receptor Gamma
2412.	SCARB2	Scavenger Receptor Class B Member 2
2413.	TCF7L2	Transcription Factor 7 Like 2
2414.	ADORA3	Adenosine A3 Receptor
2415.	APOA2	Apolipoprotein A2
2416.	APOL1	Apolipoprotein L1
2417.	CCKBR	Cholecystokinin B Receptor
2418.	CHRNA7	Cholinergic Receptor Nicotinic Alpha 7 Subunit
2419.	DAO	D-Amino Acid Oxidase
2420.	GAD2	Glutamate Decarboxylase 2

2421.	SSTR3	Somatostatin Receptor 3
2422.	ACACB	Acetyl-CoA Carboxylase Beta
2423.	CRHR1	Corticotropin Releasing Hormone Receptor 1
2424.	DRD3	Dopamine Receptor D3
2425.	HRH2	Histamine Receptor H2
2426.	ADRB3	Adrenoceptor Beta 3
2427.	CCK	Cholecystokinin
2428.	CRHR2	Corticotropin Releasing Hormone Receptor 2
2429.	CYP7A1	Cytochrome P450 Family 7 Subfamily A Member 1
2430.	HRH3	Histamine Receptor H3
2431.	LIPF	Lipase F, Gastric Type
2432.	LIPG	Lipase G, Endothelial Type
2433.	MDH1	Malate Dehydrogenase 1
2434.	NPY5R	Neuropeptide Y Receptor Y5
2435.	OLR1	Oxidized Low Density Lipoprotein Receptor 1
2436.	PRKAG1	Protein Kinase AMP-Activated Non-Catalytic Subunit Gamma 1
2437.	RETN	Resistin
2438.	ADIPOR2	Adiponectin Receptor 2
2439.	ANKRD1	Ankyrin Repeat Domain 1
2440.	AOC1	Amine Oxidase Copper Containing 1
2441.	APOA4	Apolipoprotein A4
2442.	GHRL	Ghrelin And Obestatin Prepropeptide
2443.	HTR6	5-Hydroxytryptamine Receptor 6
2444.	PYY	Peptide YY
2445.	RXRG	Retinoid X Receptor Gamma
2446.	SLC39A7	Solute Carrier Family 39 Member 7
2447.	SST	Somatostatin
2448.	MC3R	Melanocortin 3 Receptor
2449.	MCPH1	Microcephalin 1
2450.	TNFAIP6	TNF Alpha Induced Protein 6
2451.	APOM	Apolipoprotein M
2452.	GHRH	Growth Hormone Releasing Hormone
2453.	APOL2	Apolipoprotein L2
2454.	CALY	Calcyon Neuron Specific Vesicular Protein
2455.	PIK3C2G	Phosphatidylinositol-4-Phosphate 3-Kinase Catalytic Subunit Type 2 Gamma
2456.	AVEN	Apoptosis And Caspase Activation Inhibitor
2457.	TADA2A	Transcriptional Adaptor 2A
2458.	APOC4	Apolipoprotein C4
2459.	APOF	Apolipoprotein F
2460.	APOL5	Apolipoprotein L5
2461.	VHL	Von Hippel-Lindau Tumor Suppressor

2462.	NR1H2	Nuclear Receptor Subfamily 1 Group H Member 2
2463.	CALCA	Calcitonin Related Polypeptide Alpha
2464.	COG2	Component Of Oligomeric Golgi Complex 2
2465.	FGFR1	Fibroblast Growth Factor Receptor 1
2466.	IGF1R	Insulin Like Growth Factor 1 Receptor
2467.	ABL1	ABL Proto-Oncogene 1, Non-Receptor Tyrosine Kinase
2468.	CASP8	Caspase 8
2469.	CSF1R	Colony Stimulating Factor 1 Receptor
2470.	KRAS	KRAS Proto-Oncogene, GTPase
2471.	PSEN1	Presenilin 1
2472.	ACVR1	Activin A Receptor Type 1
2473.	BAX	BCL2 Associated X, Apoptosis Regulator
2474.	FGFR4	Fibroblast Growth Factor Receptor 4
2475.	FOXO1	Forkhead Box O1
2476.	SOS1	SOS Ras/Rac Guanine Nucleotide Exchange Factor 1
2477.	VIM	Vimentin
2478.	AIFM1	Apoptosis Inducing Factor Mitochondria Associated 1
2479.	GRM1	Glutamate Metabotropic Receptor 1
2480.	PGR	Progesterone Receptor
2481.	PRKCG	Protein Kinase C Gamma
2482.	PSMB8	Proteasome 20S Subunit Beta 8
2483.	SLC1A2	Solute Carrier Family 1 Member 2
2484.	TUBB3	Tubulin Beta 3 Class III
2485.	ACTB	Actin Beta
2486.	ACVR2B	Activin A Receptor Type 2B
2487.	CACNA1B	Calcium Voltage-Gated Channel Subunit Alpha1 B
2488.	CACNA1G	Calcium Voltage-Gated Channel Subunit Alpha1 G
2489.	CACNA1H	Calcium Voltage-Gated Channel Subunit Alpha1 H
2490.	DNM1	Dynamin 1
2491.	ESR2	Estrogen Receptor 2
2492.	GRIN2D	Glutamate Ionotropic Receptor NMDA Type Subunit 2D
2493.	GSN	Gelsolin
2494.	ITGB1	Integrin Subunit Beta 1
2495.	ITPR1	Inositol 1,4,5-Trisphosphate Receptor Type 1
2496.	JUN	Jun Proto-Oncogene, AP-1 Transcription Factor Subunit
2497.	LAMB1	Laminin Subunit Beta 1
2498.	MTAP	Methylthioadenosine Phosphorylase
2499.	NR5A1	Nuclear Receptor Subfamily 5 Group A Member 1
2500.	SLC1A1	Solute Carrier Family 1 Member 1
2501.	SNAP25	Synaptosome Associated Protein 25
2502.	STAT5B	Signal Transducer And Activator Of Transcription 5B

2503.	TNC	Tenascin C
2504.	TNFSF11	TNF Superfamily Member 11
2505.	GLUL	Glutamate-Ammonia Ligase
2506.	PIKFYVE	Phosphoinositide Kinase, FYVE-Type Zinc Finger Containing
2507.	PRNP	Prion Protein (Kanno Blood Group)
2508.	ALDH1A1	Aldehyde Dehydrogenase 1 Family Member A1
2509.	BACE1	Beta-Secretase 1
2510.	F7	Coagulation Factor VII
2511.	F8	Coagulation Factor VIII
2512.	GLI1	GLI Family Zinc Finger 1
2513.	GNRHR	Gonadotropin Releasing Hormone Receptor
2514.	NF1	Neurofibromin 1
2515.	NFATC1	Nuclear Factor Of Activated T Cells 1
2516.	PLA2G4A	Phospholipase A2 Group IVA
2517.	TRPA1	Transient Receptor Potential Cation Channel Subfamily A Member 1
2518.	TRPV1	Transient Receptor Potential Cation Channel Subfamily V Member 1
2519.	ANXA1	Annexin A1
2520.	BMP7	Bone Morphogenetic Protein 7
2521.	CACNA1E	Calcium Voltage-Gated Channel Subunit Alpha1 E
2522.	CBFB	Core-Binding Factor Subunit Beta
2523.	DGAT1	Diacylglycerol O-Acyltransferase 1
2524.	FH	Fumarate Hydratase
2525.	FOXA2	Forkhead Box A2
2526.	LIFR	LIF Receptor Subunit Alpha
2527.	MAG	Myelin Associated Glycoprotein
2528.	MYCN	MYCN Proto-Oncogene, BHLH Transcription Factor
2529.	NFATC2	Nuclear Factor Of Activated T Cells 2
2530.	PCCB	Propionyl-CoA Carboxylase Subunit Beta
2531.	PIP5K1C	Phosphatidylinositol-4-Phosphate 5-Kinase Type 1 Gamma
2532.	PLA2G6	Phospholipase A2 Group VI
2533.	POR	Cytochrome P450 Oxidoreductase
2534.	RBPJ	Recombination Signal Binding Protein For Immunoglobulin Kappa J Region
2535.	SMAD9	SMAD Family Member 9
2536.	ADCY6	Adenylate Cyclase 6
2537.	ARG2	Arginase 2
2538.	CA8	Carbonic Anhydrase 8
2539.	CACNA1I	Calcium Voltage-Gated Channel Subunit Alpha1 I
2540.	CDK7	Cyclin Dependent Kinase 7
2541.	CEBPA	CCAAT Enhancer Binding Protein Alpha
2542.	CHRNA1	Cholinergic Receptor Nicotinic Alpha 1 Subunit
2543.	EWSR1	EWS RNA Binding Protein 1

2544.	GATM	Glycine Amidinotransferase
2545.	GDF5	Growth Differentiation Factor 5
2546.	KCNE1	Potassium Voltage-Gated Channel Subfamily E Regulatory Subunit 1
2547.	KCNJ11	Potassium Inwardly Rectifying Channel Subfamily J Member 11
2548.	PSMB4	Proteasome 20S Subunit Beta 4
2549.	TACR1	Tachykinin Receptor 1
2550.	WWOX	WW Domain Containing Oxidoreductase
2551.	ABHD5	Abhydrolase Domain Containing 5, Lysophosphatidic Acid Acyltransferase
2552.	AMH	Anti-Mullerian Hormone
2553.	AOX1	Aldehyde Oxidase 1
2554.	ASIC1	Acid Sensing Ion Channel Subunit 1
2555.	CNTN1	Contactin 1
2556.	CRAT	Carnitine O-Acetyltransferase
2557.	DKK1	Dickkopf WNT Signaling Pathway Inhibitor 1
2558.	DNAJB1	DnaJ Heat Shock Protein Family (Hsp40) Member B1
2559.	FAM20C	FAM20C Golgi Associated Secretory Pathway Kinase
2560.	FDFT1	Farnesyl-Diphosphate Farnesyltransferase 1
2561.	GCLC	Glutamate-Cysteine Ligase Catalytic Subunit
2562.	GFER	Growth Factor, Augmenter Of Liver Regeneration
2563.	GRID2	Glutamate Ionotropic Receptor Delta Type Subunit 2
2564.	ISCU	Iron-Sulfur Cluster Assembly Enzyme
2565.	KLF5	KLF Transcription Factor 5
2566.	LMX1B	LIM Homeobox Transcription Factor 1 Beta
2567.	MAOB	Monoamine Oxidase B
2568.	MED12	Mediator Complex Subunit 12
2569.	NCOA2	Nuclear Receptor Coactivator 2
2570.	PITX1	Paired Like Homeodomain 1
2571.	SCN4B	Sodium Voltage-Gated Channel Beta Subunit 4
2572.	SDHD	Succinate Dehydrogenase Complex Subunit D
2573.	SLC1A6	Solute Carrier Family 1 Member 6
2574.	TSPO	Translocator Protein
2575.	VIP	Vasoactive Intestinal Peptide
2576.	ACP1	Acid Phosphatase 1
2577.	ANXA11	Annexin A11
2578.	CALB1	Calbindin 1
2579.	CFL2	Cofilin 2
2580.	COQ7	Coenzyme Q7, Hydroxylase
2581.	DCK	Deoxycytidine Kinase
2582.	FHIT	Fragile Histidine Triad Diadenosine Triphosphatase
2583.	GAP43	Growth Associated Protein 43
2584.	GH1	Growth Hormone 1

2585.	GREM1	Gremlin 1, DAN Family BMP Antagonist
2586.	HLCS	Holocarboxylase Synthetase
2587.	IGFBP1	Insulin Like Growth Factor Binding Protein 1
2588.	KCND2	Potassium Voltage-Gated Channel Subfamily D Member 2
2589.	MCCC2	Methylcrotonyl-CoA Carboxylase Subunit 2
2590.	MYOF	Myoferlin
2591.	OSM	Oncostatin M
2592.	SGCE	Sarcoglycan Epsilon
2593.	SLC25A19	Solute Carrier Family 25 Member 19
2594.	SLC26A2	Solute Carrier Family 26 Member 2
2595.	SOX6	SRY-Box Transcription Factor 6
2596.	TRPV3	Transient Receptor Potential Cation Channel Subfamily V Member 3
2597.	TTBK2	Tau Tubulin Kinase 2
2598.	ACTG2	Actin Gamma 2, Smooth Muscle
2599.	ADCYAP1	Adenylate Cyclase Activating Polypeptide 1
2600.	BACE2	Beta-Secretase 2
2601.	BANF1	BAF Nuclear Assembly Factor 1
2602.	BCKDHA	Branched Chain Keto Acid Dehydrogenase E1 Subunit Alpha
2603.	CA14	Carbonic Anhydrase 14
2604.	DMP1	Dentin Matrix Acidic Phosphoprotein 1
2605.	FLCN	Folliculin
2606.	GIPC1	GIPC PDZ Domain Containing Family Member 1
2607.	HOXA11	Homeobox A11
2608.	IGHMBP2	Immunoglobulin Mu DNA Binding Protein 2
2609.	KCNJ12	Potassium Inwardly Rectifying Channel Subfamily J Member 12
2610.	KCNJ8	Potassium Inwardly Rectifying Channel Subfamily J Member 8
2611.	MCCC1	Methylcrotonyl-CoA Carboxylase Subunit 1
2612.	MYL9	Myosin Light Chain 9
2613.	PREP	Prolyl Endopeptidase
2614.	PRG4	Proteoglycan 4
2615.	PSMC4	Proteasome 26S Subunit, ATPase 4
2616.	PTPN3	Protein Tyrosine Phosphatase Non-Receptor Type 3
2617.	RICTOR	RPTOR Independent Companion Of MTOR Complex 2
2618.	SETX	Senataxin
2619.	SLCO2A1	Solute Carrier Organic Anion Transporter Family Member 2A1
2620.	TFE3	Transcription Factor Binding To IGHM Enhancer 3
2621.	CILP	Cartilage Intermediate Layer Protein
2622.	DHX16	DEAH-Box Helicase 16
2623.	GPAA1	Glycosylphosphatidylinositol Anchor Attachment 1
2624.	GPAM	Glycerol-3-Phosphate Acyltransferase, Mitochondrial
2625.	MATN1	Matrilin 1

2626.	MICU1	Mitochondrial Calcium Uptake 1
2627.	MLYCD	Malonyl-CoA Decarboxylase
2628.	NES	Nestin
2629.	PALLD	Palladin, Cytoskeletal Associated Protein
2630.	PLIN2	Perilipin 2
2631.	POU1F1	POU Class 1 Homeobox 1
2632.	PRRT2	Proline Rich Transmembrane Protein 2
2633.	SIL1	SIL1 Nucleotide Exchange Factor
2634.	VPS13B	Vacuolar Protein Sorting 13 Homolog B
2635.	ANO6	Anoctamin 6
2636.	COL12A1	Collagen Type XII Alpha 1 Chain
2637.	GOLGA2	Golgin A2
2638.	HOXA10	Homeobox A10
2639.	KCNK18	Potassium Two Pore Domain Channel Subfamily K Member 18
2640.	KCNV2	Potassium Voltage-Gated Channel Modifier Subfamily V Member 2
2641.	LMOD1	Leiomodin 1
2642.	MUS81	MUS81 Structure-Specific Endonuclease Subunit
2643.	PLEK	Pleckstrin
2644.	POU2F2	POU Class 2 Homeobox 2
2645.	PRSS2	Serine Protease 2
2646.	SMCHD1	Structural Maintenance Of Chromosomes Flexible Hinge Domain Containing 1
2647.	TRIM2	Tripartite Motif Containing 2
2648.	TRIM71	Tripartite Motif Containing 71
2649.	TRPM3	Transient Receptor Potential Cation Channel Subfamily M Member 3
2650.	CACNG1	Calcium Voltage-Gated Channel Auxiliary Subunit Gamma 1
2651.	COLQ	Collagen Like Tail Subunit Of Asymmetric Acetylcholinesterase
2652.	CYGB	Cytoglobin
2653.	FLVCR2	FLVCR Choline And Putative Heme Transporter 2
2654.	FYCO1	FYVE And Coiled-Coil Domain Autophagy Adaptor 1
2655.	HFM1	Helicase For Meiosis 1
2656.	HSPB2	Heat Shock Protein Family B (Small) Member 2
2657.	KCNC2	Potassium Voltage-Gated Channel Subfamily C Member 2
2658.	MLF1	Myeloid Leukemia Factor 1
2659.	MTMR4	Myotubularin Related Protein 4
2660.	PIGN	Phosphatidylinositol Glycan Anchor Biosynthesis Class N
2661.	POLD3	DNA Polymerase Delta 3, Accessory Subunit
2662.	PPCS	Phosphopantothenoylcysteine Synthetase
2663.	SMPD4	Sphingomyelin Phosphodiesterase 4
2664.	SNTB1	Syntrophin Beta 1
2665.	TBC1D24	TBC1 Domain Family Member 24
2666.	ANO2	Anoctamin 2

2667.	ASPSCR1	ASPSCR1 Tether For SLC2A4, UBX Domain Containing
2668.	BBOX1	Gamma-Butyrobetaine Hydroxylase 1
2669.	BRAT1	BRCA1 Associated ATM Activator 1
2670.	CA5B	Carbonic Anhydrase 5B
2671.	CCL8	C-C Motif Chemokine Ligand 8
2672.	CIDEA	Cell Death Inducing DFFA Like Effector A
2673.	CSGALNACT2	Chondroitin Sulfate N-Acetylgalactosaminyltransferase 2
2674.	ECE2	Endothelin Converting Enzyme 2
2675.	ECEL1	Endothelin Converting Enzyme Like 1
2676.	EPG5	Ectopic P-Granules 5 Autophagy Tethering Factor
2677.	EXTL2	Exostosin Like Glycosyltransferase 2
2678.	LMF1	Lipase Maturation Factor 1
2679.	MAGEL2	MAGE Family Member L2
2680.	MTMR6	Myotubularin Related Protein 6
2681.	NMNAT2	Nicotinamide Nucleotide Adenylyltransferase 2
2682.	OPA3	Outer Mitochondrial Membrane Lipid Metabolism Regulator OPA3
2683.	PIAS4	Protein Inhibitor Of Activated STAT 4
2684.	PNOC	Prepronociceptin
2685.	PPP1R8	Protein Phosphatase 1 Regulatory Subunit 8
2686.	PPP2R3C	Protein Phosphatase 2 Regulatory Subunit B"Gamma
2687.	PTP4A2	Protein Tyrosine Phosphatase 4A2
2688.	RIF1	Replication Timing Regulatory Factor 1
2689.	SNTB2	Syntrophin Beta 2
2690.	TPM4	Tropomyosin 4
2691.	UBR4	Ubiquitin Protein Ligase E3 Component N-Recognin 4
2692.	AGPAT3	1-Acylglycerol-3-Phosphate O-Acyltransferase 3
2693.	AGPAT4	1-Acylglycerol-3-Phosphate O-Acyltransferase 4
2694.	AGPAT5	1-Acylglycerol-3-Phosphate O-Acyltransferase 5
2695.	COL25A1	Collagen Type XXV Alpha 1 Chain
2696.	CTR9	CTR9 Homolog, Paf1/RNA Polymerase II Complex Component
2697.	DNAJB4	DnaJ Heat Shock Protein Family (Hsp40) Member B4
2698.	DR1	Down-Regulator Of Transcription 1
2699.	DUSP13B	Dual Specificity Phosphatase 13B
2700.	EME1	Essential Meiotic Structure-Specific Endonuclease 1
2701.	EXTL1	Exostosin Like Glycosyltransferase 1
2702.	FGGY	FGGY Carbohydrate Kinase Domain Containing
2703.	GBX2	Gastrulation Brain Homeobox 2
2704.	GDF7	Growth Differentiation Factor 7
2705.	LIPN	Lipase Family Member N
2706.	MEPE	Matrix Extracellular Phosphoglycoprotein
2707.	MGAT5B	Alpha-1,6-Mannosylglycoprotein 6-Beta-N-Acetylglucosaminyltransferase B

2708.	MYH4	Myosin Heavy Chain 4
2709.	NAXD	NAD(P)HX Dehydratase
2710.	NDRG2	NDRG Family Member 2
2711.	PSMG2	Proteasome Assembly Chaperone 2
2712.	TBPL1	TATA-Box Binding Protein Like 1
2713.	TFB2M	Transcription Factor B2, Mitochondrial
2714.	ABI2	Abl Interactor 2
2715.	AMBRA1	Autophagy And Beclin 1 Regulator 1
2716.	ATP6V0E1	ATPase H+ Transporting V0 Subunit E1
2717.	CA13	Carbonic Anhydrase 13
2718.	EIF3K	Eukaryotic Translation Initiation Factor 3 Subunit K
2719.	KCNE4	Potassium Voltage-Gated Channel Subfamily E Regulatory Subunit 4
2720.	KPNA5	Karyopherin Subunit Alpha 5
2721.	MTMR12	Myotubularin Related Protein 12
2722.	MTMR9	Myotubularin Related Protein 9
2723.	PDZD8	PDZ Domain Containing 8
2724.	PLEK2	Pleckstrin 2
2725.	SFXN2	Sideroflexin 2
2726.	SLC37A1	Solute Carrier Family 37 Member 1
2727.	TAMM41	TAM41 Mitochondrial Translocator Assembly And Maintenance Homolog
2728.	TMEM38A	Transmembrane Protein 38A
2729.	TNNC2	Troponin C2, Fast Skeletal Type
2730.	CHPF2	Chondroitin Polymerizing Factor 2
2731.	GATC	Glutamyl-TRNA Amidotransferase Subunit C
2732.	ISLR2	Immunoglobulin Superfamily Containing Leucine Rich Repeat 2
2733.	LPCAT4	Lysophosphatidylcholine Acyltransferase 4
2734.	MICOS13	Mitochondrial Contact Site And Cristae Organizing System Subunit 13
2735.	MKX	Mohawk Homeobox
2736.	NDNF	Neuron Derived Neurotrophic Factor
2737.	SMPX	Small Muscle Protein X-Linked
2738.	TLE5	TLE Family Member 5, Transcriptional Modulator
2739.	TMEM11	Transmembrane Protein 11
2740.	TRMT10A	TRNA Methyltransferase 10A
2741.	ATF5	Activating Transcription Factor 5
2742.	BCORL1	BCL6 Corepressor Like 1
2743.	COA5	Cytochrome C Oxidase Assembly Factor 5
2744.	EMP1	Epithelial Membrane Protein 1
2745.	NAV3	Neuron Navigator 3
2746.	NRSN2	Neurensin 2
2747.	NXPH4	Neurexophilin 4
2748.	PXYLP1	2-Phosphoxylose Phosphatase 1

2749.	SDHAF1	Succinate Dehydrogenase Complex Assembly Factor 1
2750.	SERTAD1	SERTA Domain Containing 1
2751.	SLC37A3	Solute Carrier Family 37 Member 3
2752.	SYT14	Synaptotagmin 14
2753.	VMA21	Vacuolar ATPase Assembly Factor VMA21
2754.	VPS13C	Vacuolar Protein Sorting 13 Homolog C
2755.	VPS13D	Vacuolar Protein Sorting 13 Homolog D
2756.	VTA1	Vesicle Trafficking 1
2757.	ZFP42	ZFP42 Zinc Finger Protein
2758.	MTRFR	Mitochondrial Translation Release Factor In Rescue
2759.	POPDC2	Popeye Domain Containing 2
2760.	TRAT1	T Cell Receptor Associated Transmembrane Adaptor 1
2761.	ZBTB42	Zinc Finger And BTB Domain Containing 42
2762.	BLTP3B	Bridge-Like Lipid Transfer Protein Family Member 3B
2763.	ERAS	ES Cell Expressed Ras
2764.	MAGEB4	MAGE Family Member B4
2765.	MBOAT2	Membrane Bound O-Acyltransferase Domain Containing 2
2766.	MIER1	MIER1 Transcriptional Regulator
2767.	MYOM3	Myomesin 3
2768.	RNF32	Ring Finger Protein 32
2769.	SSX1	SSX Family Member 1
2770.	TIMM21	Translocase Of Inner Mitochondrial Membrane 21
2771.	YIPF1	Yip1 Domain Family Member 1
2772.	ATAD3C	ATPase Family AAA Domain Containing 3C
2773.	BLTP2	Bridge-Like Lipid Transfer Protein Family Member 2
2774.	DNAJB7	DnaJ Heat Shock Protein Family (Hsp40) Member B7
2775.	DPPA3	Developmental Pluripotency Associated 3
2776.	GPAT2	Glycerol-3-Phosphate Acyltransferase 2, Mitochondrial
2777.	H2AC1	H2A Clustered Histone 1
2778.	NEUROD6	Neuronal Differentiation 6
2779.	PRDM10	PR/SET Domain 10
2780.	CMTM1	CKLF Like MARVEL Transmembrane Domain Containing 1
2781.	SCX	Scleraxis BHLH Transcription Factor
2782.	TMEM266	Transmembrane Protein 266
2783.	VGLL2	Vestigial Like Family Member 2
2784.	CXorf66	Chromosome X Open Reading Frame 66
2785.	SMIM43	Small Integral Membrane Protein 43
2786.	MBD3L5	Methyl-CpG Binding Domain Protein 3 Like 5
2787.	TRG	T Cell Receptor Gamma Locus
2788.	TRD	T Cell Receptor Delta Locus
2789.	YWHAE	Tyrosine 3-Monooxygenase/Tryptophan 5-Monooxygenase Activation Protein Epsilon

2790.	ACAT1	Acetyl-CoA Acetyltransferase 1
2791.	YWHAG	Tyrosine 3-Monooxygenase/Tryptophan 5-Monooxygenase Activation Protein Gamma
2792.	HMGA1	High Mobility Group AT-Hook 1
2793.	ITGAL	Integrin Subunit Alpha L
2794.	YWHAB	Tyrosine 3-Monooxygenase/Tryptophan 5-Monooxygenase Activation Protein Beta
2795.	YWHAQ	Tyrosine 3-Monooxygenase/Tryptophan 5-Monooxygenase Activation Protein Theta
2796.	YWHAH	Tyrosine 3-Monooxygenase/Tryptophan 5-Monooxygenase Activation Protein Eta
2797.	YWHAZ	Tyrosine 3-Monooxygenase/Tryptophan 5-Monooxygenase Activation Protein Zeta
2798.	ACAT2	Acetyl-CoA Acetyltransferase 2
2799.	HTR2C	5-Hydroxytryptamine Receptor 2C
2800.	IL1R2	Interleukin 1 Receptor Type 2
2801.	RARS1	Arginyl-TRNA Synthetase 1
2802.	SOAT1	Sterol O-Acyltransferase 1
2803.	ACP3	Acid Phosphatase 3
2804.	SOAT2	Sterol O-Acyltransferase 2
2805.	DNMT1	DNA Methyltransferase 1
2806.	GNAS	GNAS Complex Locus
2807.	CYP17A1	Cytochrome P450 Family 17 Subfamily A Member 1
2808.	TUBB	Tubulin Beta Class I
2809.	AKR1B1	Aldo-Keto Reductase Family 1 Member B
2810.	SORD	Sorbitol Dehydrogenase
2811.	HSP90B1	Heat Shock Protein 90 Beta Family Member 1
2812.	ERCC1	ERCC Excision Repair 1, Endonuclease Non-Catalytic Subunit
2813.	TPH1	Tryptophan Hydroxylase 1
2814.	ABCB7	ATP Binding Cassette Subfamily B Member 7
2815.	DPAGT1	Dolichyl-Phosphate N-Acetylglucosaminephosphotransferase 1
2816.	HMBS	Hydroxymethylbilane Synthase
2817.	HTR1B	5-Hydroxytryptamine Receptor 1B
2818.	TG	Thyroglobulin
2819.	TUBA1B	Tubulin Alpha 1b
2820.	HTR4	5-Hydroxytryptamine Receptor 4
2821.	THPO	Thrombopoietin
2822.	GTF2E2	General Transcription Factor IIE Subunit 2
2823.	IL11	Interleukin 11
2824.	LGALS3BP	Galectin 3 Binding Protein
2825.	TCL1A	TCL1 Family AKT Coactivator A
2826.	NEK3	NIMA Related Kinase 3
2827.	ABCE1	ATP Binding Cassette Subfamily E Member 1
2828.	RLN1	Relaxin 1

2829.	CTAG1B	Cancer/Testis Antigen 1B

Supplementary Table S4. Genes associated with fatigue that are extracted from GeneCards.

Index	Gene Symbol	Gene Full Name
1.	IL6	Interleukin 6
2.	TNF	Tumor Necrosis Factor
3.	IL10	Interleukin 10
4.	NR3C1	Nuclear Receptor Subfamily 3 Group C Member 1
5.	IL1B	Interleukin 1 Beta
6.	SERPINA6	Serpin Family A Member 6
7.	IFNG	Interferon Gamma
8.	CRP	C-Reactive Protein
9.	TP53	Tumor Protein P53
10.	HLA-DRB1	Major Histocompatibility Complex, Class II, DR Beta 1
11.	IL2	Interleukin 2
12.	INS	Insulin
13.	POMC	Proopiomelanocortin
14.	ALB	Albumin
15.	IGF1	Insulin Like Growth Factor 1
16.	CXCL8	C-X-C Motif Chemokine Ligand 8
17.	BRAF	B-Raf Proto-Oncogene, Serine/Threonine Kinase
18.	KRAS	KRAS Proto-Oncogene, GTPase
19.	TGFB1	Transforming Growth Factor Beta 1
20.	IL4	Interleukin 4
21.	CTLA4	Cytotoxic T-Lymphocyte Associated Protein 4
22.	RNASEL	Ribonuclease L
23.	PTPN22	Protein Tyrosine Phosphatase Non-Receptor Type 22
24.	SLC6A4	Solute Carrier Family 6 Member 4
25.	CRH	Corticotropin Releasing Hormone
26.	KIT	KIT Proto-Oncogene, Receptor Tyrosine Kinase
27.	MAOA	Monoamine Oxidase A
28.	AMPD1	Adenosine Monophosphate Deaminase 1
29.	HFE	Homeostatic Iron Regulator
30.	TTN	Titin
31.	IL1A	Interleukin 1 Alpha
32.	PIK3CA	Phosphatidylinositol-4,5-Bisphosphate 3-Kinase Catalytic Subunit Alpha
33.	STIM1	Stromal Interaction Molecule 1
34.	BRCA2	BRCA2 DNA Repair Associated
35.	DMD	Dystrophin

36.	JAK2	Janus Kinase 2
37.	BDNF	Brain Derived Neurotrophic Factor
38.	HLA-B	Major Histocompatibility Complex, Class I, B
39.	DMGDH	Dimethylglycine Dehydrogenase
40.	CCND1	Cyclin D1
41.	FLT3	Fms Related Receptor Tyrosine Kinase 3
42.	ATM	ATM Serine/Threonine Kinase
43.	TET2	Tet Methylcytosine Dioxygenase 2
44.	NRAS	NRAS Proto-Oncogene, GTPase
45.	B2M	Beta-2-Microglobulin
46.	BRCA1	BRCA1 DNA Repair Associated
47.	EIF2AK2	Eukaryotic Translation Initiation Factor 2 Alpha Kinase 2
48.	HRAS	HRas Proto-Oncogene, GTPase
49.	G6PD	Glucose-6-Phosphate Dehydrogenase
50.	IL17A	Interleukin 17A
51.	CBS	Cystathionine Beta-Synthase
52.	DNMT3A	DNA Methyltransferase 3 Alpha
53.	SCN5A	Sodium Voltage-Gated Channel Alpha Subunit 5
54.	TAFAZZIN	Tafazzin, Phospholipid-Lysophospholipid Transacylase
55.	TERT	Telomerase Reverse Transcriptase
56.	HBB	Hemoglobin Subunit Beta
57.	BMP6	Bone Morphogenetic Protein 6
58.	PTPN11	Protein Tyrosine Phosphatase Non-Receptor Type 11
59.	SOD1	Superoxide Dismutase 1
60.	FGFR1	Fibroblast Growth Factor Receptor 1
61.	IL1RN	Interleukin 1 Receptor Antagonist
62.	HTR1A	5-Hydroxytryptamine Receptor 1A
63.	TUG1	Taurine Up-Regulated 1
64.	CD4	CD4 Molecule
65.	NLRP3	NLR Family Pyrin Domain Containing 3
66.	GH1	Growth Hormone 1
67.	SDHB	Succinate Dehydrogenase Complex Iron Sulfur Subunit B
68.	PYGM	Glycogen Phosphorylase, Muscle Associated
69.	CTNNB1	Catenin Beta 1
70.	MEN1	Menin 1
71.	CDKN2A	Cyclin Dependent Kinase Inhibitor 2A
72.	MYD88	MYD88 Innate Immune Signal Transduction Adaptor
73.	TRPM3	Transient Receptor Potential Cation Channel Subfamily M Member 3
74.	PRL	Prolactin
75.	RET	Ret Proto-Oncogene
76.	COMT	Catechol-O-Methyltransferase

77.	IGF2	Insulin Like Growth Factor 2
78.	MYH7	Myosin Heavy Chain 7
79.	ACE	Angiotensin I Converting Enzyme
80.	CD8A	CD8 Subunit Alpha
81.	AMPD3	Adenosine Monophosphate Deaminase 3
82.	EPO	Erythropoietin
83.	IFNA1	Interferon Alpha 1
84.	NPM1	Nucleophosmin 1
85.	GBA1	Glucosylceramidase Beta 1
86.	TNNI3	Troponin I3, Cardiac Type
87.	SCN4A	Sodium Voltage-Gated Channel Alpha Subunit 4
88.	EGFR	Epidermal Growth Factor Receptor
89.	RUNX1	RUNX Family Transcription Factor 1
90.	TLR4	Toll Like Receptor 4
91.	STAT3	Signal Transducer And Activator Of Transcription 3
92.	IDH1	Isocitrate Dehydrogenase (NADP(+)) 1
93.	LMNA	Lamin A/C
94.	GATA2	GATA Binding Protein 2
95.	SLC6A2	Solute Carrier Family 6 Member 2
96.	GPT	Glutamic--Pyruvic Transaminase
97.	RYR1	Ryanodine Receptor 1
98.	PDCD1	Programmed Cell Death 1
99.	NF1	Neurofibromin 1
100.	SLC12A3	Solute Carrier Family 12 Member 3
101.	SLC4A1	Solute Carrier Family 4 Member 1 (Diego Blood Group)
102.	SH2B3	SH2B Adaptor Protein 3
103.	TNNT2	Troponin T2, Cardiac Type
104.	PFKM	Phosphofructokinase, Muscle
105.	ALK	ALK Receptor Tyrosine Kinase
106.	CALR	Calreticulin
107.	FGFR2	Fibroblast Growth Factor Receptor 2
108.	POLG	DNA Polymerase Gamma, Catalytic Subunit
109.	MET	MET Proto-Oncogene, Receptor Tyrosine Kinase
110.	CDK4	Cyclin Dependent Kinase 4
111.	ORAI1	ORAI Calcium Release-Activated Calcium Modulator 1
112.	IDH2	Isocitrate Dehydrogenase (NADP(+)) 2
113.	HBA1	Hemoglobin Subunit Alpha 1
114.	ESR1	Estrogen Receptor 1
115.	GNAS	GNAS Complex Locus
116.	BCR	BCR Activator Of RhoGEF And GTPase
117.	CAPN3	Calpain 3

118.	HTR2A	5-Hydroxytryptamine Receptor 2A
119.	PRKAR1A	Protein Kinase CAMP-Dependent Type I Regulatory Subunit Alpha
120.	AGRN	Agrin
121.	SETBP1	SET Binding Protein 1
122.	MPL	MPL Proto-Oncogene, Thrombopoietin Receptor
123.	HBA2	Hemoglobin Subunit Alpha 2
124.	SMAD4	SMAD Family Member 4
125.	ITGAM	Integrin Subunit Alpha M
126.	ERBB2	Erb-B2 Receptor Tyrosine Kinase 2
127.	SUN2	Sad1 And UNC84 Domain Containing 2
128.	TF	Transferrin
129.	CFI	Complement Factor I
130.	IRF4	Interferon Regulatory Factor 4
131.	TPH2	Tryptophan Hydroxylase 2
132.	LRRK2	Leucine Rich Repeat Kinase 2
133.	RAPSN	Receptor Associated Protein Of The Synapse
134.	IGFBP1	Insulin Like Growth Factor Binding Protein 1
135.	PGK1	Phosphoglycerate Kinase 1
136.	FAS	Fas Cell Surface Death Receptor
137.	NKX2-1	NK2 Homeobox 1
138.	MT-ND1	Mitochondrially Encoded NADH:Ubiquinone Oxidoreductase Core Subunit 1
139.	MT-CO3	Mitochondrially Encoded Cytochrome C Oxidase III
140.	VIP	Vasoactive Intestinal Peptide
141.	BCL2	BCL2 Apoptosis Regulator
142.	PDGFRB	Platelet Derived Growth Factor Receptor Beta
143.	SRSF2	Serine And Arginine Rich Splicing Factor 2
144.	MYH6	Myosin Heavy Chain 6
145.	LRRC56	Leucine Rich Repeat Containing 56
146.	TTR	Transthyretin
147.	ABL1	ABL Proto-Oncogene 1, Non-Receptor Tyrosine Kinase
148.	STAT1	Signal Transducer And Activator Of Transcription 1
149.	UGT1A1	UDP Glucuronosyltransferase Family 1 Member A1
150.	AURKA	Aurora Kinase A
151.	PRTN3	Proteinase 3
152.	CSF3R	Colony Stimulating Factor 3 Receptor
153.	RTEL1	Regulator Of Telomere Elongation Helicase 1
154.	IFNA2	Interferon Alpha 2
155.	PALB2	Partner And Localizer Of BRCA2
156.	FBN1	Fibrillin 1
157.	TFR2	Transferrin Receptor 2
158.	MYC	MYC Proto-Oncogene, BHLH Transcription Factor

159.	TGFBR2	Transforming Growth Factor Beta Receptor 2
160.	CAV3	Caveolin 3
161.	ELANE	Elastase, Neutrophil Expressed
162.	ABCC9	ATP Binding Cassette Subfamily C Member 9
163.	ATRX	ATRX Chromatin Remodeler
164.	DES	Desmin
165.	MAP2K1	Mitogen-Activated Protein Kinase Kinase 1
166.	MT-CYB	Mitochondrially Encoded Cytochrome B
167.	CD79A	CD79a Molecule
168.	CHAT	Choline O-Acetyltransferase
169.	ACTC1	Actin Alpha Cardiac Muscle 1
170.	PDGFRA	Platelet Derived Growth Factor Receptor Alpha
171.	AIRE	Autoimmune Regulator
172.	DBH	Dopamine Beta-Hydroxylase
173.	SYT2	Synaptotagmin 2
174.	FGFR3	Fibroblast Growth Factor Receptor 3
175.	CDC73	Cell Division Cycle 73
176.	AKT1	AKT Serine/Threonine Kinase 1
177.	PNKD	PNKD Metallo-Beta-Lactamase Domain Containing
178.	SLC16A1	Solute Carrier Family 16 Member 1
179.	CSF3	Colony Stimulating Factor 3
180.	MAOB	Monoamine Oxidase B
181.	FCGR3B	Fc Gamma Receptor IIIb
182.	LEP	Leptin
183.	ATXN2	Ataxin 2
184.	NKX2-5	NK2 Homeobox 5
185.	CHRNE	Cholinergic Receptor Nicotinic Epsilon Subunit
186.	RARA	Retinoic Acid Receptor Alpha
187.	IL1R1	Interleukin 1 Receptor Type 1
188.	SPP1	Secreted Phosphoprotein 1
189.	ASXL1	ASXL Transcriptional Regulator 1
190.	PSAP	Prosaposin
191.	CHEK2	Checkpoint Kinase 2
192.	FLNC	Filamin C
193.	HLA-DQB1	Major Histocompatibility Complex, Class II, DQ Beta 1
194.	TH	Tyrosine Hydroxylase
195.	TRAPPC11	Trafficking Protein Particle Complex Subunit 11
196.	SF3B1	Splicing Factor 3b Subunit 1
197.	CD40LG	CD40 Ligand
198.	MB	Myoglobin
199.	MTHFR	Methylenetetrahydrofolate Reductase

200.	FUS	FUS RNA Binding Protein
201.	STAT5B	Signal Transducer And Activator Of Transcription 5B
202.	ICAM1	Intercellular Adhesion Molecule 1
203.	LDB3	LIM Domain Binding 3
204.	CR2	Complement C3d Receptor 2
205.	VEGFA	Vascular Endothelial Growth Factor A
206.	LAMA2	Laminin Subunit Alpha 2
207.	EZH2	Enhancer Of Zeste 2 Polycomb Repressive Complex 2 Subunit
208.	NOS2	Nitric Oxide Synthase 2
209.	ACE2	Angiotensin Converting Enzyme 2
210.	EPOR	Erythropoietin Receptor
211.	MTOR	Mechanistic Target Of Rapamycin Kinase
212.	TCF3	Transcription Factor 3
213.	FIP1L1	Factor Interacting With PAPOLA And CPSF1
214.	CREBBP	CREB Binding Protein
215.	MLH1	MutL Homolog 1
216.	JAK1	Janus Kinase 1
217.	HAMP	Hepcidin Antimicrobial Peptide
218.	KMT2A	Lysine Methyltransferase 2A
219.	PTGS2	Prostaglandin-Endoperoxide Synthase 2
220.	MYBPC3	Myosin Binding Protein C3
221.	MYL2	Myosin Light Chain 2
222.	ALAS2	5'-Aminolevulinate Synthase 2
223.	SPTA1	Spectrin Alpha, Erythrocytic 1
224.	TRHR	Thyrotropin Releasing Hormone Receptor
225.	SDHA	Succinate Dehydrogenase Complex Flavoprotein Subunit A
226.	ADCYAP1	Adenylate Cyclase Activating Polypeptide 1
227.	APOE	Apolipoprotein E
228.	ETV6	ETS Variant Transcription Factor 6
229.	CLCN1	Chloride Voltage-Gated Channel 1
230.	SRC	SRC Proto-Oncogene, Non-Receptor Tyrosine Kinase
231.	BCOR	BCL6 Corepressor
232.	CSRP3	Cysteine And Glycine Rich Protein 3
233.	RAF1	Raf-1 Proto-Oncogene, Serine/Threonine Kinase
234.	ANKRD1	Ankyrin Repeat Domain 1
235.	ACTA1	Actin Alpha 1, Skeletal Muscle
236.	SOCS1	Suppressor Of Cytokine Signaling 1
237.	IFNGR1	Interferon Gamma Receptor 1
238.	CFH	Complement Factor H
239.	MAPT	Microtubule Associated Protein Tau
240.	IFNB1	Interferon Beta 1

241.	IL6R	Interleukin 6 Receptor
242.	HLA-DPB1	Major Histocompatibility Complex, Class II, DP Beta 1
243.	AIP	Aryl Hydrocarbon Receptor Interacting Protein
244.	HTR3A	5-Hydroxytryptamine Receptor 3A
245.	U2AF1	U2 Small Nuclear RNA Auxiliary Factor 1
246.	BAG3	BAG Cochaperone 3
247.	CDH23	Cadherin Related 23
248.	MSH2	MutS Homolog 2
249.	HBG2	Hemoglobin Subunit Gamma 2
250.	DNAH8	Dynein Axonemal Heavy Chain 8
251.	HSP90AA1	Heat Shock Protein 90 Alpha Family Class A Member 1
252.	FANCD2	FA Complementation Group D2
253.	ABCD1	ATP Binding Cassette Subfamily D Member 1
254.	SLC17A5	Solute Carrier Family 17 Member 5
255.	PTEN	Phosphatase And Tensin Homolog
256.	CASQ1	Calsequestrin 1
257.	DRD2	Dopamine Receptor D2
258.	ISCU	Iron-Sulfur Cluster Assembly Enzyme
259.	HJV	Hemojuvelin BMP Co-Receptor
260.	IL13	Interleukin 13
261.	CPT2	Carnitine Palmitoyltransferase 2
262.	INSL6	Insulin Like 6
263.	C4A	Complement C4A (Chido/Rodgers Blood Group)
264.	ACTN2	Actinin Alpha 2
265.	BARD1	BRCA1 Associated RING Domain 1
266.	MECP2	Methyl-CpG Binding Protein 2
267.	PPARG	Peroxisome Proliferator Activated Receptor Gamma
268.	TLR7	Toll Like Receptor 7
269.	GATA1	GATA Binding Protein 1
270.	SELL	Selectin L
271.	NR4A2	Nuclear Receptor Subfamily 4 Group A Member 2
272.	WT1	WT1 Transcription Factor
273.	TNPO3	Transportin 3
274.	SCN11A	Sodium Voltage-Gated Channel Alpha Subunit 11
275.	TCAP	Titin-Cap
276.	IRF5	Interferon Regulatory Factor 5
277.	CDKN2C	Cyclin Dependent Kinase Inhibitor 2C
278.	SLC6A3	Solute Carrier Family 6 Member 3
279.	TPM1	Tropomyosin 1
280.	LTA	Lymphotoxin Alpha
281.	BCL6	BCL6 Transcription Repressor

282.	CXCL10	C-X-C Motif Chemokine Ligand 10
283.	ADRB2	Adrenoceptor Beta 2
284.	MAPK1	Mitogen-Activated Protein Kinase 1
285.	RECQL4	RecQ Like Helicase 4
286.	FKTN	Fukutin
287.	PML	PML Nuclear Body Scaffold
288.	TNFAIP3	TNF Alpha Induced Protein 3
289.	KCNJ5	Potassium Inwardly Rectifying Channel Subfamily J Member 5
290.	REN	Renin
291.	FOXP3	Forkhead Box P3
292.	POU1F1	POU Class 1 Homeobox 1
293.	MC2R	Melanocortin 2 Receptor
294.	SGK1	Serum/Glucocorticoid Regulated Kinase 1
295.	F2	Coagulation Factor II, Thrombin
296.	BAP1	BRCA1 Associated Protein 1
297.	PSEN1	Presenilin 1
298.	MBL2	Mannose Binding Lectin 2
299.	TNXB	Tenascin XB
300.	CBFB	Core-Binding Factor Subunit Beta
301.	FKRP	Fukutin Related Protein
302.	CASR	Calcium Sensing Receptor
303.	FLNA	Filamin A
304.	CS	Citrate Synthase
305.	EPCAM	Epithelial Cell Adhesion Molecule
306.	HSPD1	Heat Shock Protein Family D (Hsp60) Member 1
307.	PIK3CG	Phosphatidylinositol-4,5-Bisphosphate 3-Kinase Catalytic Subunit Gamma
308.	MYO9A	Myosin IXA
309.	IL12RB1	Interleukin 12 Receptor Subunit Beta 1
310.	FBXW7	F-Box And WD Repeat Domain Containing 7
311.	PRRT2	Proline Rich Transmembrane Protein 2
312.	NFKB1	Nuclear Factor Kappa B Subunit 1
313.	LAMP2	Lysosomal Associated Membrane Protein 2
314.	TARDBP	TAR DNA Binding Protein
315.	SERPINA1	Serpin Family A Member 1
316.	PMS2	PMS1 Homolog 2, Mismatch Repair System Component
317.	FCGR2B	Fc Gamma Receptor IIb
318.	VAMP1	Vesicle Associated Membrane Protein 1
319.	SLC5A7	Solute Carrier Family 5 Member 7
320.	STAT4	Signal Transducer And Activator Of Transcription 4
321.	SLC40A1	Solute Carrier Family 40 Member 1
322.	CACNA1S	Calcium Voltage-Gated Channel Subunit Alpha1 S

323.	SLC11A1	Solute Carrier Family 11 Member 1
324.	CD46	CD46 Molecule
325.	CSF2	Colony Stimulating Factor 2
326.	VHL	Von Hippel-Lindau Tumor Suppressor
327.	BLK	BLK Proto-Oncogene, Src Family Tyrosine Kinase
328.	CALCA	Calcitonin Related Polypeptide Alpha
329.	MEFV	MEFV Innate Immunity Regulator, Pyrin
330.	HSPA4	Heat Shock Protein Family A (Hsp70) Member 4
331.	TYMS	Thymidylate Synthetase
332.	IL5	Interleukin 5
333.	PSEN2	Presenilin 2
334.	DOK7	Docking Protein 7
335.	SLC25A1	Solute Carrier Family 25 Member 1
336.	ETS1	ETS Proto-Oncogene 1, Transcription Factor
337.	COL1A2	Collagen Type I Alpha 2 Chain
338.	PROP1	PROP Paired-Like Homeobox 1
339.	HCRT	Hypocretin Neuropeptide Precursor
340.	SELE	Selectin E
341.	INSR	Insulin Receptor
342.	CP	Ceruloplasmin
343.	MT-ATP6	Mitochondrially Encoded ATP Synthase Membrane Subunit 6
344.	POLG2	DNA Polymerase Gamma 2, Accessory Subunit
345.	GFAP	Glial Fibrillary Acidic Protein
346.	DSP	Desmoplakin
347.	MPO	Myeloperoxidase
348.	IL36RN	Interleukin 36 Receptor Antagonist
349.	TBX5	T-Box Transcription Factor 5
350.	DNAJB6	DnaJ Heat Shock Protein Family (Hsp40) Member B6
351.	NLRC4	NLR Family CARD Domain Containing 4
352.	CLCNKB	Chloride Voltage-Gated Channel Kb
353.	SLCO1C1	Solute Carrier Organic Anion Transporter Family Member 1C1
354.	MYPN	Myopalladin
355.	IL7R	Interleukin 7 Receptor
356.	NCOR2	Nuclear Receptor Corepressor 2
357.	STK11	Serine/Threonine Kinase 11
358.	FHIT	Fragile Histidine Triad Diadenosine Triphosphatase
359.	BMP2	Bone Morphogenetic Protein 2
360.	MT-CO1	Mitochondrially Encoded Cytochrome C Oxidase I
361.	TNIP1	TNFAIP3 Interacting Protein 1
362.	EPAS1	Endothelial PAS Domain Protein 1
363.	CDKN1B	Cyclin Dependent Kinase Inhibitor 1B

364.	GCH1	GTP Cyclohydrolase 1
365.	CRBN	Cereblon
366.	CDKN1A	Cyclin Dependent Kinase Inhibitor 1A
367.	IL1RAPL2	Interleukin 1 Receptor Accessory Protein Like 2
368.	C11orf65	Chromosome 11 Open Reading Frame 65
369.	KMT2D	Lysine Methyltransferase 2D
370.	SF3B2	Splicing Factor 3b Subunit 2
371.	VPS13C	Vacuolar Protein Sorting 13 Homolog C
372.	FASLG	Fas Ligand
373.	USP8	Ubiquitin Specific Peptidase 8
374.	TGFB2	Transforming Growth Factor Beta 2
375.	P4HA2	Prolyl 4-Hydroxylase Subunit Alpha 2
376.	PGM1	Phosphoglucomutase 1
377.	TBP	TATA-Box Binding Protein
378.	APOH	Apolipoprotein H
379.	TBX20	T-Box Transcription Factor 20
380.	MST1R	Macrophage Stimulating 1 Receptor
381.	RXRA	Retinoid X Receptor Alpha
382.	ERF	ETS2 Repressor Factor
383.	CKM	Creatine Kinase, M-Type
384.	COL1A1	Collagen Type I Alpha 1 Chain
385.	DNASE1L1	Deoxyribonuclease 1 Like 1
386.	GCK	Glucokinase
387.	CCL5	C-C Motif Chemokine Ligand 5
388.	NPPB	Natriuretic Peptide B
389.	IGFBP3	Insulin Like Growth Factor Binding Protein 3
390.	TNFSF13B	TNF Superfamily Member 13b
391.	CD69	CD69 Molecule
392.	GFPT1	Glutamine--Fructose-6-Phosphate Transaminase 1
393.	MSH6	MutS Homolog 6
394.	IGHG1	Immunoglobulin Heavy Constant Gamma 1 (G1m Marker)
395.	DRD3	Dopamine Receptor D3
396.	DNASE1	Deoxyribonuclease 1
397.	SPTB	Spectrin Beta, Erythrocytic
398.	LHX4	LIM Homeobox 4
399.	TET3	Tet Methylcytosine Dioxygenase 3
400.	C4B	Complement C4B (Chido/Rodgers Blood Group)
401.	TMEM126B	Transmembrane Protein 126B
402.	LIG4	DNA Ligase 4
403.	FCGR2A	Fc Gamma Receptor IIa
404.	TOP2A	DNA Topoisomerase II Alpha

405.	LDHA	Lactate Dehydrogenase A
406.	HIF1A	Hypoxia Inducible Factor 1 Subunit Alpha
407.	NSD1	Nuclear Receptor Binding SET Domain Protein 1
408.	SNCA	Synuclein Alpha
409.	CAT	Catalase
410.	ATXN3	Ataxin 3
411.	SGCD	Sarcoglycan Delta
412.	IRAK1	Interleukin 1 Receptor Associated Kinase 1
413.	ENG	Endoglin
414.	RRM2B	Ribonucleotide Reductase Regulatory TP53 Inducible Subunit M2B
415.	TCF4	Transcription Factor 4
416.	NOS1	Nitric Oxide Synthase 1
417.	HDAC4	Histone Deacetylase 4
418.	CEBPA	CCAAT Enhancer Binding Protein Alpha
419.	GATA6	GATA Binding Protein 6
420.	APC	APC Regulator Of WNT Signaling Pathway
421.	MUC5B	Mucin 5B, Oligomeric Mucus/Gel-Forming
422.	BMPR2	Bone Morphogenetic Protein Receptor Type 2
423.	HLA-DQA1	Major Histocompatibility Complex, Class II, DQ Alpha 1
424.	IKBKG	Inhibitor Of Nuclear Factor Kappa B Kinase Regulatory Subunit Gamma
425.	GATA4	GATA Binding Protein 4
426.	HNF4A	Hepatocyte Nuclear Factor 4 Alpha
427.	TRH	Thyrotropin Releasing Hormone
428.	CLCN2	Chloride Voltage-Gated Channel 2
429.	VCL	Vinculin
430.	SLC18A3	Solute Carrier Family 18 Member A3
431.	IL15	Interleukin 15
432.	TNFRSF1A	TNF Receptor Superfamily Member 1A
433.	ACAD9	Acyl-CoA Dehydrogenase Family Member 9
434.	DDX41	DEAD-Box Helicase 41
435.	NUP214	Nucleoporin 214
436.	AP2S1	Adaptor Related Protein Complex 2 Subunit Sigma 1
437.	CASP3	Caspase 3
438.	ARMC5	Armadillo Repeat Containing 5
439.	TFRC	Transferrin Receptor
440.	ACADM	Acyl-CoA Dehydrogenase Medium Chain
441.	MT-ATP8	Mitochondrially Encoded ATP Synthase Membrane Subunit 8
442.	MAPK3	Mitogen-Activated Protein Kinase 3
443.	SLC2A1	Solute Carrier Family 2 Member 1
444.	IL6ST	Interleukin 6 Cytokine Family Signal Transducer
445.	CDH1	Cadherin 1

446.	LTF	Lactotransferrin
447.	RB1	RB Transcriptional Corepressor 1
448.	HSPB1	Heat Shock Protein Family B (Small) Member 1
449.	POLD1	DNA Polymerase Delta 1, Catalytic Subunit
450.	LHX3	LIM Homeobox 3
451.	SCNN1G	Sodium Channel Epithelial 1 Subunit Gamma
452.	HLA-DPA1	Major Histocompatibility Complex, Class II, DP Alpha 1
453.	PRKN	Parkin RBR E3 Ubiquitin Protein Ligase
454.	TGFBR1	Transforming Growth Factor Beta Receptor 1
455.	PLCG2	Phospholipase C Gamma 2
456.	CASP8	Caspase 8
457.	UBE2L3	Ubiquitin Conjugating Enzyme E2 L3
458.	ZBTB16	Zinc Finger And BTB Domain Containing 16
459.	MUS81	MUS81 Structure-Specific Endonuclease Subunit
460.	EME1	Essential Meiotic Structure-Specific Endonuclease 1
461.	GYPC	Glycophorin C (Gerbich Blood Group)
462.	ERCC4	ERCC Excision Repair 4, Endonuclease Catalytic Subunit
463.	ADAMTS13	ADAM Metallopeptidase With Thrombospondin Type 1 Motif 13
464.	MICU1	Mitochondrial Calcium Uptake 1
465.	BRIP1	BRCA1 Interacting Helicase 1
466.	YAP1	Yes1 Associated Transcriptional Regulator
467.	PTH	Parathyroid Hormone
468.	CHRNA1	Cholinergic Receptor Nicotinic Alpha 1 Subunit
469.	MT-ND5	Mitochondrially Encoded NADH:Ubiquinone Oxidoreductase Core Subunit 5
470.	COLQ	Collagen Like Tail Subunit Of Asymmetric Acetylcholinesterase
471.	CACNA1A	Calcium Voltage-Gated Channel Subunit Alpha1 A
472.	LBR	Lamin B Receptor
473.	SFTPA2	Surfactant Protein A2
474.	P2RX7	Purinergic Receptor P2X 7
475.	RETN	Resistin
476.	PPOX	Protoporphyrinogen Oxidase
477.	KCNE1	Potassium Voltage-Gated Channel Subfamily E Regulatory Subunit 1
478.	BTNL2	Butyrophilin Like 2
479.	PARN	Poly(A)-Specific Ribonuclease
480.	POLD3	DNA Polymerase Delta 3, Accessory Subunit
481.	KMT2C	Lysine Methyltransferase 2C
482.	BCORL1	BCL6 Corepressor Like 1
483.	CIITA	Class II Major Histocompatibility Complex Transactivator
484.	NFE2L2	NFE2 Like BZIP Transcription Factor 2
485.	IL18	Interleukin 18
486.	COL2A1	Collagen Type II Alpha 1 Chain

487.	NBN	Nibrin
488.	FGF2	Fibroblast Growth Factor 2
489.	SOD2	Superoxide Dismutase 2
490.	CCL2	C-C Motif Chemokine Ligand 2
491.	VDR	Vitamin D Receptor
492.	OPTN	Optineurin
493.	HCN4	Hyperpolarization Activated Cyclic Nucleotide Gated Potassium Channel 4
494.	KDM5C	Lysine Demethylase 5C
495.	TRNT1	TRNA Nucleotidyl Transferase 1
496.	IL3	Interleukin 3
497.	DSPP	Dentin Sialophosphoprotein
498.	ADA2	Adenosine Deaminase 2
499.	PIGA	Phosphatidylinositol Glycan Anchor Biosynthesis Class A
500.	NOTCH2NLC	Notch 2 N-Terminal Like C
501.	SCNN1A	Sodium Channel Epithelial 1 Subunit Alpha
502.	SCNN1B	Sodium Channel Epithelial 1 Subunit Beta
503.	SST	Somatostatin
504.	COL6A2	Collagen Type VI Alpha 2 Chain
505.	PHKA2	Phosphorylase Kinase Regulatory Subunit Alpha 2
506.	IL10RB	Interleukin 10 Receptor Subunit Beta
507.	IL12A	Interleukin 12A
508.	CUBN	Cubilin
509.	MT-ND4	Mitochondrially Encoded NADH:Ubiquinone Oxidoreductase Core Subunit 4
510.	HMGB1	High Mobility Group Box 1
511.	MUTYH	MutY DNA Glycosylase
512.	TLR3	Toll Like Receptor 3
513.	PIK3C2A	Phosphatidylinositol-4-Phosphate 3-Kinase Catalytic Subunit Type 2 Alpha
514.	H3C1	H3 Clustered Histone 1
515.	NPPA	Natriuretic Peptide A
516.	DYSF	Dysferlin
517.	CHRND	Cholinergic Receptor Nicotinic Delta Subunit
518.	CDKN2B	Cyclin Dependent Kinase Inhibitor 2B
519.	ERCC2	ERCC Excision Repair 2, TFIIH Core Complex Helicase Subunit
520.	DSG2	Desmoglein 2
521.	EPB41	Erythrocyte Membrane Protein Band 4.1
522.	RBM20	RNA Binding Motif Protein 20
523.	TNFSF4	TNF Superfamily Member 4
524.	JAZF1	JAZF Zinc Finger 1
525.	F3	Coagulation Factor III, Tissue Factor
526.	NEFL	Neurofilament Light Chain
527.	DTNA	Dystrobrevin Alpha

528.	MYCN	MYCN Proto-Oncogene, BHLH Transcription Factor
529.	MLLT10	MLLT10 Histone Lysine Methyltransferase DOT1L Cofactor
530.	KDR	Kinase Insert Domain Receptor
531.	NPY	Neuropeptide Y
532.	ABCC8	ATP Binding Cassette Subfamily C Member 8
533.	PKLR	Pyruvate Kinase L/R
534.	HP	Haptoglobin
535.	GAA	Alpha Glucosidase
536.	FGF23	Fibroblast Growth Factor 23
537.	SMARCA4	SWI/SNF Related, Matrix Associated, Actin Dependent Regulator Of Chromatin, Subfamily A, Member 4
538.	WWOX	WW Domain Containing Oxidoreductase
539.	ADA	Adenosine Deaminase
540.	ABCB1	ATP Binding Cassette Subfamily B Member 1
541.	POMGNT1	Protein O-Linked Mannose N-Acetylglucosaminyltransferase 1 (Beta 1,2-)
542.	TMPO	Thymopoietin
543.	NEXN	Nexilin F-Actin Binding Protein
544.	MMEL1	Membrane Metalloendopeptidase Like 1
545.	IL2RB	Interleukin 2 Receptor Subunit Beta
546.	CACNB4	Calcium Voltage-Gated Channel Auxiliary Subunit Beta 4
547.	GLA	Galactosidase Alpha
548.	GNAI2	G Protein Subunit Alpha I2
549.	RAD51C	RAD51 Paralog C
550.	AP1S3	Adaptor Related Protein Complex 1 Subunit Sigma 3
551.	SLC11A2	Solute Carrier Family 11 Member 2
552.	HNF1A	HNF1 Homeobox A
553.	TREX1	Three Prime Repair Exonuclease 1
554.	PXK	PX Domain Containing Serine/Threonine Kinase Like
555.	BANK1	B Cell Scaffold Protein With Ankyrin Repeats 1
556.	HTR3B	5-Hydroxytryptamine Receptor 3B
557.	PIK3R2	Phosphoinositide-3-Kinase Regulatory Subunit 2
558.	SAMHD1	SAM And HD Domain Containing Deoxynucleoside Triphosphate Triphosphohydrolase 1
559.	DIS3	DIS3 Homolog, Exosome Endoribonuclease And 3'-5' Exoribonuclease
560.	ARID4A	AT-Rich Interaction Domain 4A
561.	KCNJ18	Potassium Inwardly Rectifying Channel Subfamily J Member 18
562.	KCNJ1	Potassium Inwardly Rectifying Channel Subfamily J Member 1
563.	TEK	TEK Receptor Tyrosine Kinase
564.	TMPRSS6	Transmembrane Serine Protease 6
565.	TPO	Thyroid Peroxidase
566.	TSC1	TSC Complex Subunit 1
567.	IRF1	Interferon Regulatory Factor 1

568.	XIAP	X-Linked Inhibitor Of Apoptosis
569.	GABRA1	Gamma-Aminobutyric Acid Type A Receptor Subunit Alpha1
570.	DNAJC13	DnaJ Heat Shock Protein Family (Hsp40) Member C13
571.	PHKG2	Phosphorylase Kinase Catalytic Subunit Gamma 2
572.	KCNE3	Potassium Voltage-Gated Channel Subfamily E Regulatory Subunit 3
573.	SNAP25	Synaptosome Associated Protein 25
574.	IFNAR2	Interferon Alpha And Beta Receptor Subunit 2
575.	SVIL	Supervillin
576.	SDHC	Succinate Dehydrogenase Complex Subunit C
577.	NDUFS4	NADH:Ubiquinone Oxidoreductase Subunit S4
578.	MAPK14	Mitogen-Activated Protein Kinase 14
579.	PON1	Paraoxonase 1
580.	SELENON	Selenoprotein N
581.	OPA1	OPA1 Mitochondrial Dynamin Like GTPase
582.	PDSS1	Decaprenyl Diphosphate Synthase Subunit 1
583.	PHKA1	Phosphorylase Kinase Regulatory Subunit Alpha 1
584.	CRHR1	Corticotropin Releasing Hormone Receptor 1
585.	BIRC3	Baculoviral IAP Repeat Containing 3
586.	TNNC1	Troponin C1, Slow Skeletal And Cardiac Type
587.	AMN	Amnion Associated Transmembrane Protein
588.	SGCB	Sarcoglycan Beta
589.	VCP	Valosin Containing Protein
590.	RPL5	Ribosomal Protein L5
591.	FANCG	FA Complementation Group G
592.	RAD51	RAD51 Recombinase
593.	SMAD3	SMAD Family Member 3
594.	DPP4	Dipeptidyl Peptidase 4
595.	IL7	Interleukin 7
596.	MST1	Macrophage Stimulating 1
597.	RPS20	Ribosomal Protein S20
598.	STN1	STN1 Subunit Of CST Complex
599.	MECOM	MDS1 And EVI1 Complex Locus
600.	GABRD	Gamma-Aminobutyric Acid Type A Receptor Subunit Delta
601.	TG	Thyroglobulin
602.	GSR	Glutathione-Disulfide Reductase
603.	GMPPB	GDP-Mannose Pyrophosphorylase B
604.	SLC22A4	Solute Carrier Family 22 Member 4
605.	CRYAB	Crystallin Alpha B
606.	ATRIP	ATR Interacting Protein
607.	DNAJC21	DnaJ Heat Shock Protein Family (Hsp40) Member C21
608.	FANCA	FA Complementation Group A

609.	THPO	Thrombopoietin
610.	EMD	Emerin
611.	FXN	Frataxin
612.	APOB	Apolipoprotein B
613.	COL5A1	Collagen Type V Alpha 1 Chain
614.	KIF1B	Kinesin Family Member 1B
615.	TLL1	Tolloid Like 1
616.	SQSTM1	Sequestosome 1
617.	GGT1	Gamma-Glutamyltransferase 1
618.	PTPRC	Protein Tyrosine Phosphatase Receptor Type C
619.	DISC1	DISC1 Scaffold Protein
620.	SLC16A4	Solute Carrier Family 16 Member 4
621.	PRDM16	PR/SET Domain 16
622.	GNB2	G Protein Subunit Beta 2
623.	TWNK	Twinkle MtDNA Helicase
624.	VWF	Von Willebrand Factor
625.	PICALM	Phosphatidylinositol Binding Clathrin Assembly Protein
626.	CD36	CD36 Molecule (CD36 Blood Group)
627.	PPARGC1A	PPARG Coactivator 1 Alpha
628.	RHOA	Ras Homolog Family Member A
629.	IDO1	Indoleamine 2,3-Dioxygenase 1
630.	TBL1XR1	TBL1X/Y Related 1
631.	NUMA1	Nuclear Mitotic Apparatus Protein 1
632.	KIAA0319L	KIAA0319 Like
633.	IRF2BP2	Interferon Regulatory Factor 2 Binding Protein 2
634.	NABP1	Nucleic Acid Binding Protein 1
635.	ANXA11	Annexin A11
636.	CKB	Creatine Kinase B
637.	TAB2	TGF-Beta Activated Kinase 1 (MAP3K7) Binding Protein 2
638.	CYLD	CYLD Lysine 63 Deubiquitinase
639.	LATS1	Large Tumor Suppressor Kinase 1
640.	H1-4	H1.4 Linker Histone, Cluster Member
641.	TRAF5	TNF Receptor Associated Factor 5
642.	EIF1AX	Eukaryotic Translation Initiation Factor 1A X-Linked
643.	FAT1	FAT Atypical Cadherin 1
644.	MGA	MAX Dimerization Protein MGA
645.	P2RY8	P2Y Receptor Family Member 8
646.	FAM135A	Family With Sequence Similarity 135 Member A
647.	H2AC16	H2A Clustered Histone 16
648.	H2AC17	H2A Clustered Histone 17
649.	XPO1	Exportin 1

650.	HLA-A	Major Histocompatibility Complex, Class I, A
651.	SPAST	Spastin
652.	CD34	CD34 Molecule
653.	HBG1	Hemoglobin Subunit Gamma 1
654.	CYP3A4	Cytochrome P450 Family 3 Subfamily A Member 4
655.	ACTA2	Actin Alpha 2, Smooth Muscle
656.	NEFH	Neurofilament Heavy Chain
657.	PLEC	Plectin
658.	PIK3R1	Phosphoinositide-3-Kinase Regulatory Subunit 1
659.	LPL	Lipoprotein Lipase
660.	C3	Complement C3
661.	BMPR1A	Bone Morphogenetic Protein Receptor Type 1A
662.	GDF15	Growth Differentiation Factor 15
663.	ERCC6L2	ERCC Excision Repair 6 Like 2
664.	NPHP1	Nephrocystin 1
665.	CEP290	Centrosomal Protein 290
666.	GPI	Glucose-6-Phosphate Isomerase
667.	SLC12A1	Solute Carrier Family 12 Member 1
668.	BSND	Barttin CLCNK Type Accessory Subunit Beta
669.	PRKACA	Protein Kinase CAMP-Activated Catalytic Subunit Alpha
670.	KDM1A	Lysine Demethylase 1A
671.	PHKB	Phosphorylase Kinase Regulatory Subunit Beta
672.	MMP9	Matrix Metallopeptidase 9
673.	CD274	CD274 Molecule
674.	TXNRD2	Thioredoxin Reductase 2
675.	SERPINE1	Serpin Family E Member 1
676.	HTR2C	5-Hydroxytryptamine Receptor 2C
677.	MFSD11	Major Facilitator Superfamily Domain Containing 11
678.	CBL	Cbl Proto-Oncogene
679.	HACE1	HECT Domain And Ankyrin Repeat Containing E3 Ubiquitin Protein Ligase 1
680.	CD28	CD28 Molecule
681.	PRF1	Perforin 1
682.	BTK	Bruton Tyrosine Kinase
683.	CHGA	Chromogranin A
684.	MAPK8	Mitogen-Activated Protein Kinase 8
685.	CHD7	Chromodomain Helicase DNA Binding Protein 7
686.	CKMT2	Creatine Kinase, Mitochondrial 2
687.	USP48	Ubiquitin Specific Peptidase 48
688.	IFIH1	Interferon Induced With Helicase C Domain 1
689.	TPI1	Triosephosphate Isomerase 1
690.	SDHD	Succinate Dehydrogenase Complex Subunit D

691.	SEC23B	SEC23 Homolog B, COPII Coat Complex Component
692.	IL2RA	Interleukin 2 Receptor Subunit Alpha
693.	JUN	Jun Proto-Oncogene, AP-1 Transcription Factor Subunit
694.	TNFRSF17	TNF Receptor Superfamily Member 17
695.	DAG1	Dystroglycan 1
696.	MUC1	Mucin 1, Cell Surface Associated
697.	RPS15	Ribosomal Protein S15
698.	OXTR	Oxytocin Receptor
699.	CILK1	Ciliogenesis Associated Kinase 1
700.	MLLT3	MLLT3 Super Elongation Complex Subunit
701.	AGK	Acylglycerol Kinase
702.	SLC2A4	Solute Carrier Family 2 Member 4
703.	POMT1	Protein O-Mannosyltransferase 1
704.	ERCC5	ERCC Excision Repair 5, Endonuclease
705.	PTPN3	Protein Tyrosine Phosphatase Non-Receptor Type 3
706.	ANK1	Ankyrin 1
707.	TNFRSF10A	TNF Receptor Superfamily Member 10a
708.	IKZF1	IKAROS Family Zinc Finger 1
709.	MT-ND2	Mitochondrially Encoded NADH:Ubiquinone Oxidoreductase Core Subunit 2
710.	CASK	Calcium/Calmodulin Dependent Serine Protein Kinase
711.	CACNA1H	Calcium Voltage-Gated Channel Subunit Alpha1 H
712.	CD79B	CD79b Molecule
713.	TFEB	Transcription Factor EB
714.	SLC2A2	Solute Carrier Family 2 Member 2
715.	CD19	CD19 Molecule
716.	IFI27	Interferon Alpha Inducible Protein 27
717.	CKMT1B	Creatine Kinase, Mitochondrial 1B
718.	MLIP	Muscular LMNA Interacting Protein
719.	OPRM1	Opioid Receptor Mu 1
720.	FKBP5	FKBP Prolyl Isomerase 5
721.	CYP11B2	Cytochrome P450 Family 11 Subfamily B Member 2
722.	HTR2B	5-Hydroxytryptamine Receptor 2B
723.	TPM2	Tropomyosin 2
724.	NOTCH3	Notch Receptor 3
725.	PARP1	Poly(ADP-Ribose) Polymerase 1
726.	HDAC9	Histone Deacetylase 9
727.	PAX8	Paired Box 8
728.	HESX1	HESX Homeobox 1
729.	PALLD	Palladin, Cytoskeletal Associated Protein
730.	RABL3	RAB, Member Of RAS Oncogene Family Like 3
731.	IL1R2	Interleukin 1 Receptor Type 2

732.	TKT	Transketolase
733.	FGF21	Fibroblast Growth Factor 21
734.	GJA5	Gap Junction Protein Alpha 5
735.	IL16	Interleukin 16
736.	COQ2	Coenzyme Q2, Polyprenyltransferase
737.	C17orf107	Chromosome 17 Open Reading Frame 107
738.	BCL2L1	BCL2 Like 1
739.	LPP	LIM Domain Containing Preferred Translocation Partner In Lipoma
740.	MLF1	Myeloid Leukemia Factor 1
741.	TGM6	Transglutaminase 6
742.	HTT	Huntingtin
743.	KRT18	Keratin 18
744.	SYP	Synaptophysin
745.	SMAD2	SMAD Family Member 2
746.	CHKA	Choline Kinase Alpha
747.	SCO2	Synthesis Of Cytochrome C Oxidase 2
748.	CXCL12	C-X-C Motif Chemokine Ligand 12
749.	NEK1	NIMA Related Kinase 1
750.	TNFSF15	TNF Superfamily Member 15
751.	GPR35	G Protein-Coupled Receptor 35
752.	SPIB	Spi-B Transcription Factor
753.	MCL1	MCL1 Apoptosis Regulator, BCL2 Family Member
754.	KCNJ11	Potassium Inwardly Rectifying Channel Subfamily J Member 11
755.	MT-ND6	Mitochondrially Encoded NADH:Ubiquinone Oxidoreductase Core Subunit 6
756.	IGH	Immunoglobulin Heavy Locus
757.	HTR7	5-Hydroxytryptamine Receptor 7
758.	HTR5A	5-Hydroxytryptamine Receptor 5A
759.	HTR1E	5-Hydroxytryptamine Receptor 1E
760.	PDGFB	Platelet Derived Growth Factor Subunit B
761.	STAR	Steroidogenic Acute Regulatory Protein
762.	TLR2	Toll Like Receptor 2
763.	IRF8	Interferon Regulatory Factor 8
764.	SLC25A4	Solute Carrier Family 25 Member 4
765.	ERVW-1	Endogenous Retrovirus Group W Member 1, Envelope
766.	ANXA5	Annexin A5
767.	PLOD1	Procollagen-Lysine,2-Oxoglutarate 5-Dioxygenase 1
768.	MPZ	Myelin Protein Zero
769.	RBBP8	RB Binding Protein 8, Endonuclease
770.	CREB1	CAMP Responsive Element Binding Protein 1
771.	IQCB1	IQ Motif Containing B1
772.	NPHP4	Nephrocystin 4

773.	EFHC1	EF-Hand Domain Containing 1
774.	FANCE	FA Complementation Group E
775.	HTR4	5-Hydroxytryptamine Receptor 4
776.	UBA1	Ubiquitin Like Modifier Activating Enzyme 1
777.	CYP2D6	Cytochrome P450 Family 2 Subfamily D Member 6
778.	TBK1	TANK Binding Kinase 1
779.	CITED2	Cbp/P300 Interacting Transactivator With Glu/Asp Rich Carboxy-Terminal Domain 2
780.	IGHM	Immunoglobulin Heavy Constant Mu
781.	KLRK1	Killer Cell Lectin Like Receptor K1
782.	PDX1	Pancreatic And Duodenal Homeobox 1
783.	SNTA1	Syntrophin Alpha 1
784.	TOP1	DNA Topoisomerase I
785.	TPM3	Tropomyosin 3
786.	HGF	Hepatocyte Growth Factor
787.	FANCI	FA Complementation Group I
788.	PAGE2B	PAGE Family Member 2B
789.	CD86	CD86 Molecule
790.	CFTR	CF Transmembrane Conductance Regulator
791.	EIF2AK4	Eukaryotic Translation Initiation Factor 2 Alpha Kinase 4
792.	SPI1	Spi-1 Proto-Oncogene
793.	VCAM1	Vascular Cell Adhesion Molecule 1
794.	HOXB13	Homeobox B13
795.	SFTPC	Surfactant Protein C
796.	STEAP3	STEAP3 Metalloreductase
797.	MT-CO2	Mitochondrially Encoded Cytochrome C Oxidase II
798.	MYH14	Myosin Heavy Chain 14
799.	FANCC	FA Complementation Group C
800.	HTR6	5-Hydroxytryptamine Receptor 6
801.	TLR1	Toll Like Receptor 1
802.	CCR5	C-C Motif Chemokine Receptor 5
803.	CD38	CD38 Molecule
804.	ADRB1	Adrenoceptor Beta 1
805.	CYCS	Cytochrome C, Somatic
806.	SBDS	SBDS Ribosome Maturation Factor
807.	BAX	BCL2 Associated X, Apoptosis Regulator
808.	CCL3	C-C Motif Chemokine Ligand 3
809.	MTM1	Myotubularin 1
810.	CD244	CD244 Molecule
811.	TYMP	Thymidine Phosphorylase
812.	AGER	Advanced Glycosylation End-Product Specific Receptor
813.	TPMT	Thiopurine S-Methyltransferase

814.	F5	Coagulation Factor V
815.	ITGA7	Integrin Subunit Alpha 7
816.	COL4A5	Collagen Type IV Alpha 5 Chain
817.	SLC18A2	Solute Carrier Family 18 Member A2
818.	ENPP1	Ectonucleotide Pyrophosphatase/Phosphodiesterase 1
819.	RIGI	RNA Sensor RIG-I
820.	PKP2	Plakophilin 2
821.	GRM5	Glutamate Metabotropic Receptor 5
822.	INVS	Inversin
823.	MT-ND3	Mitochondrially Encoded NADH:Ubiquinone Oxidoreductase Core Subunit 3
824.	CSF1R	Colony Stimulating Factor 1 Receptor
825.	TRPA1	Transient Receptor Potential Cation Channel Subfamily A Member 1
826.	POT1	Protection Of Telomeres 1
827.	NEK8	NIMA Related Kinase 8
828.	IL17F	Interleukin 17F
829.	DEK	DEK Proto-Oncogene
830.	CYP19A1	Cytochrome P450 Family 19 Subfamily A Member 1
831.	SSTR5	Somatostatin Receptor 5
832.	RPS26	Ribosomal Protein S26
833.	HBD	Hemoglobin Subunit Delta
834.	UGT1A7	UDP Glucuronosyltransferase Family 1 Member A7
835.	SSUH2	Ssu-2 Homolog
836.	AGT	Angiotensinogen
837.	MUSK	Muscle Associated Receptor Tyrosine Kinase
838.	SOX2	SRY-Box Transcription Factor 2
839.	TNK2	Tyrosine Kinase Non Receptor 2
840.	KLK2	Kallikrein Related Peptidase 2
841.	RFC1	Replication Factor C Subunit 1
842.	SH3GL1	SH3 Domain Containing GRB2 Like 1, Endophilin A2
843.	NSD3	Nuclear Receptor Binding SET Domain Protein 3
844.	CHIC2	Cysteine Rich Hydrophobic Domain 2
845.	NOTCH2NLA	Notch 2 N-Terminal Like A
846.	ATXN8	Ataxin 8
847.	KLHL3	Kelch Like Family Member 3
848.	ALPL	Alkaline Phosphatase, Biomineralization Associated
849.	RPN1	Ribophorin I
850.	BGLAP	Bone Gamma-Carboxyglutamate Protein
851.	PVALB	Parvalbumin
852.	SCARB2	Scavenger Receptor Class B Member 2
853.	RPLP0	Ribosomal Protein Lateral Stalk Subunit P0
854.	CD40	CD40 Molecule

855.	IKZF3	IKAROS Family Zinc Finger 3
856.	EGF	Epidermal Growth Factor
857.	IL12B	Interleukin 12B
858.	POLGARF	POLG Alternative Reading Frame
859.	KCNQ1	Potassium Voltage-Gated Channel Subfamily Q Member 1
860.	ABCC2	ATP Binding Cassette Subfamily C Member 2
861.	SLC26A4	Solute Carrier Family 26 Member 4
862.	TENM4	Teneurin Transmembrane Protein 4
863.	PHOX2B	Paired Like Homeobox 2B
864.	PLN	Phospholamban
865.	SEMA4D	Semaphorin 4D
866.	LIN28B	Lin-28 Homolog B
867.	LMO1	LIM Domain Only 1
868.	POU2AF1	POU Class 2 Homeobox Associating Factor 1
869.	DNM1L	Dynamin 1 Like
870.	CCR1	C-C Motif Chemokine Receptor 1
871.	PSTPIP1	Proline-Serine-Threonine Phosphatase Interacting Protein 1
872.	PIEZO1	Piezo Type Mechanosensitive Ion Channel Component 1 (Er Blood Group)
873.	PTGS1	Prostaglandin-Endoperoxide Synthase 1
874.	FGF10	Fibroblast Growth Factor 10
875.	SLC34A1	Solute Carrier Family 34 Member 1
876.	FLCN	Folliculin
877.	UGT1A6	UDP Glucuronosyltransferase Family 1 Member A6
878.	GCM2	Glial Cells Missing Transcription Factor 2
879.	FANCM	FA Complementation Group M
880.	SLX4	SLX4 Structure-Specific Endonuclease Subunit
881.	FN1	Fibronectin 1
882.	FTL	Ferritin Light Chain
883.	GABRA3	Gamma-Aminobutyric Acid Type A Receptor Subunit Alpha3
884.	LMNB1	Lamin B1
885.	BCL10	BCL10 Immune Signaling Adaptor
886.	SGCA	Sarcoglycan Alpha
887.	CSF1	Colony Stimulating Factor 1
888.	PRKCD	Protein Kinase C Delta
889.	KRT8	Keratin 8
890.	HAVCR2	Hepatitis A Virus Cellular Receptor 2
891.	IL23A	Interleukin 23 Subunit Alpha
892.	EDNRA	Endothelin Receptor Type A
893.	FAAH	Fatty Acid Amide Hydrolase
894.	CXCR4	C-X-C Motif Chemokine Receptor 4
895.	EGR1	Early Growth Response 1

896.	CHCHD10	Coiled-Coil-Helix-Coiled-Coil-Helix Domain Containing 10
897.	HNF1B	HNF1 Homeobox B
898.	SH2D1A	SH2 Domain Containing 1A
899.	DRD4	Dopamine Receptor D4
900.	TSHR	Thyroid Stimulating Hormone Receptor
901.	IGF1R	Insulin Like Growth Factor 1 Receptor
902.	RPS6KB1	Ribosomal Protein S6 Kinase B1
903.	MDM2	MDM2 Proto-Oncogene
904.	ERBB3	Erb-B2 Receptor Tyrosine Kinase 3
905.	COL13A1	Collagen Type XIII Alpha 1 Chain
906.	CASP1	Caspase 1
907.	IL37	Interleukin 37
908.	MIPEP	Mitochondrial Intermediate Peptidase
909.	NEUROD1	Neuronal Differentiation 1
910.	PAX4	Paired Box 4
911.	HNRNPA1	Heterogeneous Nuclear Ribonucleoprotein A1
912.	HMOX1	Heme Oxygenase 1
913.	PADI4	Peptidyl Arginine Deiminase 4
914.	ADCY10	Adenylate Cyclase 10
915.	MAGED2	MAGE Family Member D2
916.	EPB42	Erythrocyte Membrane Protein Band 4.2
917.	PLAT	Plasminogen Activator, Tissue Type
918.	CPT1A	Carnitine Palmitoyltransferase 1A
919.	ASS1	Argininosuccinate Synthase 1
920.	OAS1	2'-5'-Oligoadenylate Synthetase 1
921.	DNMT1	DNA Methyltransferase 1
922.	CD80	CD80 Molecule
923.	NFKBIA	NFKB Inhibitor Alpha
924.	KCNJ2	Potassium Inwardly Rectifying Channel Subfamily J Member 2
925.	THBD	Thrombomodulin
926.	CYP21A2	Cytochrome P450 Family 21 Subfamily A Member 2
927.	ACTN3	Actinin Alpha 3
928.	SI	Sucrase-Isomaltase
929.	AGTR1	Angiotensin II Receptor Type 1
930.	STAT6	Signal Transducer And Activator Of Transcription 6
931.	ABCA3	ATP Binding Cassette Subfamily A Member 3
932.	ATP7A	ATPase Copper Transporting Alpha
933.	TSHB	Thyroid Stimulating Hormone Subunit Beta
934.	CEP164	Centrosomal Protein 164
935.	NEB	Nebulin
936.	MINK1	Misshapen Like Kinase 1

937.	ATP13A2	ATPase Cation Transporting 13A2
938.	FBN2	Fibrillin 2
939.	FCGR3A	Fc Gamma Receptor IIIa
940.	NOD2	Nucleotide Binding Oligomerization Domain Containing 2
941.	ATP7B	ATPase Copper Transporting Beta
942.	UGT1A10	UDP Glucuronosyltransferase Family 1 Member A10
943.	UGT1A4	UDP Glucuronosyltransferase Family 1 Member A4
944.	MUC2	Mucin 2, Oligomeric Mucus/Gel-Forming
945.	UGT1A8	UDP Glucuronosyltransferase Family 1 Member A8
946.	SOS1	SOS Ras/Rac Guanine Nucleotide Exchange Factor 1
947.	COMP	Cartilage Oligomeric Matrix Protein
948.	SLC33A1	Solute Carrier Family 33 Member 1
949.	MATR3	Matrin 3
950.	ARSA	Arylsulfatase A
951.	ACHE	Acetylcholinesterase (Yt Blood Group)
952.	SCN1B	Sodium Voltage-Gated Channel Beta Subunit 1
953.	KITLG	KIT Ligand
954.	NFKBIL1	NFKB Inhibitor Like 1
955.	CCK	Cholecystokinin
956.	MMP2	Matrix Metallopeptidase 2
957.	TBX19	T-Box Transcription Factor 19
958.	CHRNG	Cholinergic Receptor Nicotinic Gamma Subunit
959.	TRAF3	TNF Receptor Associated Factor 3
960.	CXCR3	C-X-C Motif Chemokine Receptor 3
961.	HADHA	Hydroxyacyl-CoA Dehydrogenase Trifunctional Multienzyme Complex Subunit Alpha
962.	ROS1	ROS Proto-Oncogene 1, Receptor Tyrosine Kinase
963.	FIG4	FIG4 Phosphoinositide 5-Phosphatase
964.	MVK	Mevalonate Kinase
965.	STAT5A	Signal Transducer And Activator Of Transcription 5A
966.	GHRL	Ghrelin And Obestatin Prepropeptide
967.	PON3	Paraoxonase 3
968.	SFTPA1	Surfactant Protein A1
969.	UNC13A	Unc-13 Homolog A
970.	TREM2	Triggering Receptor Expressed On Myeloid Cells 2
971.	KCNN4	Potassium Calcium-Activated Channel Subfamily N Member 4
972.	GAPDH	Glyceraldehyde-3-Phosphate Dehydrogenase
973.	TSC2	TSC Complex Subunit 2
974.	DRD1	Dopamine Receptor D1
975.	COL5A2	Collagen Type V Alpha 2 Chain
976.	ITGB1	Integrin Subunit Beta 1
977.	STXBP5L	Syntaxin Binding Protein 5L

978.	CHRM1	Cholinergic Receptor Muscarinic 1
979.	IGHE	Immunoglobulin Heavy Constant Epsilon
980.	MAP2K2	Mitogen-Activated Protein Kinase Kinase 2
981.	AKT2	AKT Serine/Threonine Kinase 2
982.	SLCO1B1	Solute Carrier Organic Anion Transporter Family Member 1B1
983.	VAPB	VAMP Associated Protein B And C
984.	PITX2	Paired Like Homeodomain 2
985.	AGL	Amylo-Alpha-1, 6-Glucosidase, 4-Alpha-Glucanotransferase
986.	SHBG	Sex Hormone Binding Globulin
987.	TMEM43	Transmembrane Protein 43
988.	CASP9	Caspase 9
989.	NTRK2	Neurotrophic Receptor Tyrosine Kinase 2
990.	PIK3CD	Phosphatidylinositol-4,5-Bisphosphate 3-Kinase Catalytic Subunit Delta
991.	ITGA4	Integrin Subunit Alpha 4
992.	PIK3CB	Phosphatidylinositol-4,5-Bisphosphate 3-Kinase Catalytic Subunit Beta
993.	HMGCR	3-Hydroxy-3-Methylglutaryl-CoA Reductase
994.	TJP2	Tight Junction Protein 2
995.	CHKB	Choline Kinase Beta
996.	ITPR3	Inositol 1,4,5-Trisphosphate Receptor Type 3
997.	DIPK1A	Divergent Protein Kinase Domain 1A
998.	TAPBPL	TAP Binding Protein Like
999.	SELP	Selectin P
1000.	DCTN1	Dynactin Subunit 1
1001.	HAND2	Heart And Neural Crest Derivatives Expressed 2
1002.	APOL1	Apolipoprotein L1
1003.	INTS7	Integrator Complex Subunit 7
1004.	AFP	Alpha Fetoprotein
1005.	IRS1	Insulin Receptor Substrate 1
1006.	C1R	Complement C1r
1007.	ATP1B1	ATPase Na+/K+ Transporting Subunit Beta 1
1008.	IL11	Interleukin 11
1009.	SAMD9L	Sterile Alpha Motif Domain Containing 9 Like
1010.	MASP2	MBL Associated Serine Protease 2
1011.	MME	Membrane Metalloendopeptidase
1012.	ATP13A3	ATPase 13A3
1013.	HMGCL	3-Hydroxy-3-Methylglutaryl-CoA Lyase
1014.	NRG1	Neuregulin 1
1015.	TLR8	Toll Like Receptor 8
1016.	GNRHR	Gonadotropin Releasing Hormone Receptor
1017.	PMP22	Peripheral Myelin Protein 22
1018.	PDE11A	Phosphodiesterase 11A

1019.	RASSF1	Ras Association Domain Family Member 1
1020.	GZMB	Granzyme B
1021.	KCNQ3	Potassium Voltage-Gated Channel Subfamily Q Member 3
1022.	RPL11	Ribosomal Protein L11
1023.	CD44	CD44 Molecule (IN Blood Group)
1024.	IL21	Interleukin 21
1025.	IL18R1	Interleukin 18 Receptor 1
1026.	KCNA5	Potassium Voltage-Gated Channel Subfamily A Member 5
1027.	MYO1A	Myosin IA
1028.	KRT7	Keratin 7
1029.	CD68	CD68 Molecule
1030.	SHH	Sonic Hedgehog Signaling Molecule
1031.	SYNJ1	Synaptojanin 1
1032.	SERPINA7	Serpin Family A Member 7
1033.	POLE	DNA Polymerase Epsilon, Catalytic Subunit
1034.	MFN2	Mitofusin 2
1035.	ABCG2	ATP Binding Cassette Subfamily G Member 2 (JR Blood Group)
1036.	BIRC5	Baculoviral IAP Repeat Containing 5
1037.	JUP	Junction Plakoglobin
1038.	ERVFRD-1	Endogenous Retrovirus Group FRD Member 1, Envelope
1039.	TNFSF10	TNF Superfamily Member 10
1040.	TNFRSF8	TNF Receptor Superfamily Member 8
1041.	TRPM7	Transient Receptor Potential Cation Channel Subfamily M Member 7
1042.	NDUFS1	NADH:Ubiquinone Oxidoreductase Core Subunit S1
1043.	TTC21B	Tetratricopeptide Repeat Domain 21B
1044.	NPHP3	Nephrocystin 3
1045.	IFNAR2-IL10RB	IFNAR2-IL10RB Readthrough
1046.	PNPLA8	Patatin Like Phospholipase Domain Containing 8
1047.	ATP4B	ATPase H+/K+ Transporting Subunit Beta
1048.	IL18BP	Interleukin 18 Binding Protein
1049.	ATP5F1D	ATP Synthase F1 Subunit Delta
1050.	ACADVL	Acyl-CoA Dehydrogenase Very Long Chain
1051.	SYNE1	Spectrin Repeat Containing Nuclear Envelope Protein 1
1052.	ANO5	Anoctamin 5
1053.	CDK2	Cyclin Dependent Kinase 2
1054.	NGF	Nerve Growth Factor
1055.	CAPRIN1	Cell Cycle Associated Protein 1
1056.	PTPN1	Protein Tyrosine Phosphatase Non-Receptor Type 1
1057.	ERBB4	Erb-B2 Receptor Tyrosine Kinase 4
1058.	NFKB2	Nuclear Factor Kappa B Subunit 2
1059.	FH	Fumarate Hydratase

1060.	ERV3-1	Endogenous Retrovirus Group 3 Member 1, Envelope
1061.	LYN	LYN Proto-Oncogene, Src Family Tyrosine Kinase
1062.	TLN1	Talin 1
1063.	WRN	WRN RecQ Like Helicase
1064.	DNAJB1	DnaJ Heat Shock Protein Family (Hsp40) Member B1
1065.	LPIN2	Lipin 2
1066.	ALDOA	Aldolase, Fructose-Bisphosphate A
1067.	ABCC1	ATP Binding Cassette Subfamily C Member 1 (ABCC1 Blood Group)
1068.	ENO2	Enolase 2
1069.	CYP17A1	Cytochrome P450 Family 17 Subfamily A Member 1
1070.	SLC12A4	Solute Carrier Family 12 Member 4
1071.	SFTPD	Surfactant Protein D
1072.	WFS1	Wolframin ER Transmembrane Glycoprotein
1073.	NSD2	Nuclear Receptor Binding SET Domain Protein 2
1074.	CFHR5	Complement Factor H Related 5
1075.	ARAF	A-Raf Proto-Oncogene, Serine/Threonine Kinase
1076.	HBZ	Hemoglobin Subunit Zeta
1077.	CCL4	C-C Motif Chemokine Ligand 4
1078.	TLR9	Toll Like Receptor 9
1079.	SYK	Spleen Associated Tyrosine Kinase
1080.	TRIM21	Tripartite Motif Containing 21
1081.	MGMT	O-6-Methylguanine-DNA Methyltransferase
1082.	TK2	Thymidine Kinase 2
1083.	ANG	Angiogenin
1084.	ARL11	ADP Ribosylation Factor Like GTPase 11
1085.	IGHV3-21	Immunoglobulin Heavy Variable 3-21
1086.	KL	Klotho
1087.	MYH11	Myosin Heavy Chain 11
1088.	SLC3A1	Solute Carrier Family 3 Member 1
1089.	ALS2	Alsin Rho Guanine Nucleotide Exchange Factor ALS2
1090.	SDC1	Syndecan 1
1091.	GHRH	Growth Hormone Releasing Hormone
1092.	DNAJC6	DnaJ Heat Shock Protein Family (Hsp40) Member C6
1093.	PON2	Paraoxonase 2
1094.	JPH2	Junctophilin 2
1095.	TTK	TTK Protein Kinase
1096.	SLC2A10	Solute Carrier Family 2 Member 10
1097.	SPG11	SPG11 Vesicle Trafficking Associated, Spatacsin
1098.	ABCB4	ATP Binding Cassette Subfamily B Member 4
1099.	PC	Pyruvate Carboxylase
1100.	TIMMDC1	Translocase Of Inner Mitochondrial Membrane Domain Containing 1

1101.	CXCL9	C-X-C Motif Chemokine Ligand 9
1102.	MYH2	Myosin Heavy Chain 2
1103.	LCAT	Lecithin-Cholesterol Acyltransferase
1104.	CYB5R3	Cytochrome B5 Reductase 3
1105.	DGKE	Diacylglycerol Kinase Epsilon
1106.	CYB5A	Cytochrome B5 Type A
1107.	TFE3	Transcription Factor Binding To IGHM Enhancer 3
1108.	RAD51D	RAD51 Paralog D
1109.	NDUFB11	NADH:Ubiquinone Oxidoreductase Subunit B11
1110.	KLF11	KLF Transcription Factor 11
1111.	NCAM1	Neural Cell Adhesion Molecule 1
1112.	GAST	Gastrin
1113.	NOS3	Nitric Oxide Synthase 3
1114.	CDK6	Cyclin Dependent Kinase 6
1115.	GLUL	Glutamate-Ammonia Ligase
1116.	LMOD2	Leiomodin 2
1117.	SLC25A13	Solute Carrier Family 25 Member 13
1118.	OTOF	Otoferlin
1119.	MMACHC	Metabolism Of Cobalamin Associated C
1120.	ADGRE2	Adhesion G Protein-Coupled Receptor E2
1121.	RHAG	Rh Associated Glycoprotein
1122.	MSTN	Myostatin
1123.	DMP1	Dentin Matrix Acidic Phosphoprotein 1
1124.	LTBP4	Latent Transforming Growth Factor Beta Binding Protein 4
1125.	XRCC2	X-Ray Repair Cross Complementing 2
1126.	FOXE1	Forkhead Box E1
1127.	CD5	CD5 Molecule
1128.	IRAK4	Interleukin 1 Receptor Associated Kinase 4
1129.	CORIN	Corin, Serine Peptidase
1130.	SDCCAG8	SHH Signaling And Ciliogenesis Regulator SDCCAG8
1131.	PIGM	Phosphatidylinositol Glycan Anchor Biosynthesis Class M
1132.	TRAF3IP1	TRAF3 Interacting Protein 1
1133.	WDR19	WD Repeat Domain 19
1134.	NAF1	Nuclear Assembly Factor 1 Ribonucleoprotein
1135.	YY1	YY1 Transcription Factor
1136.	CHMP2B	Charged Multivesicular Body Protein 2B
1137.	GPR101	G Protein-Coupled Receptor 101
1138.	PPARA	Peroxisome Proliferator Activated Receptor Alpha
1139.	APP	Amyloid Beta Precursor Protein
1140.	SLC2A3	Solute Carrier Family 2 Member 3
1141.	RPS17	Ribosomal Protein S17

1142.	ITGB2	Integrin Subunit Beta 2
1143.	LIF	LIF Interleukin 6 Family Cytokine
1144.	CYP1A2	Cytochrome P450 Family 1 Subfamily A Member 2
1145.	SMN1	Survival Of Motor Neuron 1, Telomeric
1146.	CCN6	Cellular Communication Network Factor 6
1147.	MRAP	Melanocortin 2 Receptor Accessory Protein
1148.	EDN1	Endothelin 1
1149.	FCGR1A	Fc Gamma Receptor Ia
1150.	PFN1	Profilin 1
1151.	CD27	CD27 Molecule
1152.	CENPA	Centromere Protein A
1153.	BCL2L11	BCL2 Like 11
1154.	CUL3	Cullin 3
1155.	FANCL	FA Complementation Group L
1156.	IRS2	Insulin Receptor Substrate 2
1157.	KLF6	KLF Transcription Factor 6
1158.	VPS4A	Vacuolar Protein Sorting 4 Homolog A
1159.	PIEZO2	Piezo Type Mechanosensitive Ion Channel Component 2
1160.	SLC52A1	Solute Carrier Family 52 Member 1
1161.	IFNAR1	Interferon Alpha And Beta Receptor Subunit 1
1162.	PSMC3	Proteasome 26S Subunit, ATPase 3
1163.	LTBP2	Latent Transforming Growth Factor Beta Binding Protein 2
1164.	BIVM-ERCC5	BIVM-ERCC5 Readthrough
1165.	GSK3B	Glycogen Synthase Kinase 3 Beta
1166.	DPYD	Dihydropyrimidine Dehydrogenase
1167.	P2RX5	Purinergic Receptor P2X 5
1168.	RPS27A	Ribosomal Protein S27a
1169.	GARS1	Glycyl-TRNA Synthetase 1
1170.	NOTCH1	Notch Receptor 1
1171.	KLK3	Kallikrein Related Peptidase 3
1172.	PAX5	Paired Box 5
1173.	CFLAR	CASP8 And FADD Like Apoptosis Regulator
1174.	TNFRSF10B	TNF Receptor Superfamily Member 10b
1175.	GRB2	Growth Factor Receptor Bound Protein 2
1176.	ITGAL	Integrin Subunit Alpha L
1177.	CASP10	Caspase 10
1178.	CBLIF	Cobalamin Binding Intrinsic Factor
1179.	AHI1	Abelson Helper Integration Site 1
1180.	NTRK1	Neurotrophic Receptor Tyrosine Kinase 1
1181.	C9orf72	C9orf72-SMCR8 Complex Subunit
1182.	GRM1	Glutamate Metabotropic Receptor 1

1183.	CCL11	C-C Motif Chemokine Ligand 11
1184.	P2RX4	Purinergic Receptor P2X 4
1185.	GLI2	GLI Family Zinc Finger 2
1186.	KCNJ3	Potassium Inwardly Rectifying Channel Subfamily J Member 3
1187.	MAX	MYC Associated Factor X
1188.	FHL2	Four And A Half LIM Domains 2
1189.	PREPL	Prolyl Endopeptidase Like
1190.	RPL3L	Ribosomal Protein L3 Like
1191.	ETFDH	Electron Transfer Flavoprotein Dehydrogenase
1192.	PKD1	Polycystin 1, Transient Receptor Potential Channel Interacting
1193.	SGCG	Sarcoglycan Gamma
1194.	MBP	Myelin Basic Protein
1195.	ANK2	Ankyrin 2
1196.	MDM4	MDM4 Regulator Of P53
1197.	MAP3K20	Mitogen-Activated Protein Kinase Kinase Kinase 20
1198.	HACD1	3-Hydroxyacyl-CoA Dehydratase 1
1199.	CHRNB1	Cholinergic Receptor Nicotinic Beta 1 Subunit
1200.	PCNA	Proliferating Cell Nuclear Antigen
1201.	LOC102723407	Immunoglobulin Heavy Variable 4-38-2-Like
1202.	FOS	Fos Proto-Oncogene, AP-1 Transcription Factor Subunit
1203.	FKBP14	FKBP Prolyl Isomerase 14
1204.	B3GAT1	Beta-1,3-Glucuronyltransferase 1
1205.	CHIT1	Chitinase 1
1206.	GNRH1	Gonadotropin Releasing Hormone 1
1207.	ELN	Elastin
1208.	SLC34A2	Solute Carrier Family 34 Member 2
1209.	THBS1	Thrombospondin 1
1210.	IVNS1ABP	Influenza Virus NS1A Binding Protein
1211.	IL23R	Interleukin 23 Receptor
1212.	GUSB	Glucuronidase Beta
1213.	PTK2	Protein Tyrosine Kinase 2
1214.	GAD1	Glutamate Decarboxylase 1
1215.	SCN9A	Sodium Voltage-Gated Channel Alpha Subunit 9
1216.	ERCC1	ERCC Excision Repair 1, Endonuclease Non-Catalytic Subunit
1217.	CCR7	C-C Motif Chemokine Receptor 7
1218.	MYOM2	Myomesin 2
1219.	THRB	Thyroid Hormone Receptor Beta
1220.	PPP2R5D	Protein Phosphatase 2 Regulatory Subunit B'Delta
1221.	FMR1	Fragile X Messenger Ribonucleoprotein 1
1222.	RHEB	Ras Homolog, MTORC1 Binding
1223.	TACR3	Tachykinin Receptor 3

1224.	HMGA1	High Mobility Group AT-Hook 1
1225.	RBPJ	Recombination Signal Binding Protein For Immunoglobulin Kappa J Region
1226.	SMAD6	SMAD Family Member 6
1227.	CEL	Carboxyl Ester Lipase
1228.	ERCC6	ERCC Excision Repair 6, Chromatin Remodeling Factor
1229.	SFPQ	Splicing Factor Proline And Glutamine Rich
1230.	MAD2L2	Mitotic Arrest Deficient 2 Like 2
1231.	SLC22A18	Solute Carrier Family 22 Member 18
1232.	RPS7	Ribosomal Protein S7
1233.	SLC30A8	Solute Carrier Family 30 Member 8
1234.	CASZ1	Castor Zinc Finger 1
1235.	ZNF469	Zinc Finger Protein 469
1236.	CCND2	Cyclin D2
1237.	IFNA5	Interferon Alpha 5
1238.	TNFRSF1B	TNF Receptor Superfamily Member 1B
1239.	ALG14	ALG14 UDP-N-Acetylglucosaminyltransferase Subunit
1240.	NDUFV1	NADH:Ubiquinone Oxidoreductase Core Subunit V1
1241.	DNM2	Dynamin 2
1242.	AAAS	Aladin WD Repeat Nucleoporin
1243.	COL9A2	Collagen Type IX Alpha 2 Chain
1244.	S100B	S100 Calcium Binding Protein B
1245.	ANPEP	Alanyl Aminopeptidase, Membrane
1246.	CHRNA4	Cholinergic Receptor Nicotinic Alpha 4 Subunit
1247.	NDUFV2	NADH:Ubiquinone Oxidoreductase Core Subunit V2
1248.	ADIPOQ	Adiponectin, C1Q And Collagen Domain Containing
1249.	HSPA5	Heat Shock Protein Family A (Hsp70) Member 5
1250.	TIMP1	TIMP Metallopeptidase Inhibitor 1
1251.	TNFRSF13B	TNF Receptor Superfamily Member 13B
1252.	CCNA2	Cyclin A2
1253.	GPT2	Glutamic--Pyruvic Transaminase 2
1254.	TNFRSF4	TNF Receptor Superfamily Member 4
1255.	SOCS3	Suppressor Of Cytokine Signaling 3
1256.	RYR2	Ryanodine Receptor 2
1257.	SIRT1	Sirtuin 1
1258.	NDUFB3	NADH:Ubiquinone Oxidoreductase Subunit B3
1259.	NUBPL	NUBP Iron-Sulfur Cluster Assembly Factor, Mitochondrial
1260.	SOX3	SRY-Box Transcription Factor 3
1261.	PDHA1	Pyruvate Dehydrogenase E1 Subunit Alpha 1
1262.	MALT1	MALT1 Paracaspase
1263.	DLST	Dihydrolipoamide S-Succinyltransferase
1264.	PRPH	Peripherin

1265.	SDHAF2	Succinate Dehydrogenase Complex Assembly Factor 2
1266.	ATP11A	ATPase Phospholipid Transporting 11A
1267.	PMS1	PMS1 Homolog 1, Mismatch Repair System Component
1268.	MLH3	MutL Homolog 3
1269.	TMEM127	Transmembrane Protein 127
1270.	GATA5	GATA Binding Protein 5
1271.	EIF2AK3	Eukaryotic Translation Initiation Factor 2 Alpha Kinase 3
1272.	ASPH	Aspartate Beta-Hydroxylase
1273.	FCER2	Fc Epsilon Receptor II
1274.	TNFSF13	TNF Superfamily Member 13
1275.	JAG1	Jagged Canonical Notch Ligand 1
1276.	TOMM40	Translocase Of Outer Mitochondrial Membrane 40
1277.	ACTB	Actin Beta
1278.	CD33	CD33 Molecule
1279.	LAMP1	Lysosomal Associated Membrane Protein 1
1280.	DHFR	Dihydrofolate Reductase
1281.	UTRN	Utrophin
1282.	TFAM	Transcription Factor A, Mitochondrial
1283.	CHEK1	Checkpoint Kinase 1
1284.	CAV1	Caveolin 1
1285.	RPS19	Ribosomal Protein S19
1286.	LIPC	Lipase C, Hepatic Type
1287.	RBM15	RNA Binding Motif Protein 15
1288.	SLC22A5	Solute Carrier Family 22 Member 5
1289.	CRAT	Carnitine O-Acetyltransferase
1290.	ALPP	Alkaline Phosphatase, Placental
1291.	ZNF350	Zinc Finger Protein 350
1292.	UNC45B	Unc-45 Myosin Chaperone B
1293.	LRIF1	Ligand Dependent Nuclear Receptor Interacting Factor 1
1294.	EIF4EBP1	Eukaryotic Translation Initiation Factor 4E Binding Protein 1
1295.	SIN3A	SIN3 Transcription Regulator Family Member A
1296.	SPTBN1	Spectrin Beta, Non-Erythrocytic 1
1297.	CHUK	Component Of Inhibitor Of Nuclear Factor Kappa B Kinase Complex
1298.	DNTT	DNA Nucleotidylexotransferase
1299.	PDE5A	Phosphodiesterase 5A
1300.	DGUOK	Deoxyguanosine Kinase
1301.	FLT1	Fms Related Receptor Tyrosine Kinase 1
1302.	MYB	MYB Proto-Oncogene, Transcription Factor
1303.	TNFRSF11B	TNF Receptor Superfamily Member 11b
1304.	GDAP1	Ganglioside Induced Differentiation Associated Protein 1
1305.	TOR1AIP1	Torsin 1A Interacting Protein 1

1306.	PSMA7	Proteasome 20S Subunit Alpha 7
1307.	TIA1	TIA1 Cytotoxic Granule Associated RNA Binding Protein
1308.	SCN1A	Sodium Voltage-Gated Channel Alpha Subunit 1
1309.	CDKN3	Cyclin Dependent Kinase Inhibitor 3
1310.	CYBRD1	Cytochrome B Reductase 1
1311.	SETX	Senataxin
1312.	SPARC	Secreted Protein Acidic And Cysteine Rich
1313.	CR1	Complement C3b/C4b Receptor 1 (Knops Blood Group)
1314.	SCT	Secretin
1315.	MYOT	Myotilin
1316.	ACO1	Aconitase 1
1317.	GCGR	Glucagon Receptor
1318.	GNB1	G Protein Subunit Beta 1
1319.	MSR1	Macrophage Scavenger Receptor 1
1320.	SUPT5H	SPT5 Homolog, DSIF Elongation Factor Subunit
1321.	RIOX2	Ribosomal Oxygenase 2
1322.	JRK	Jrk Helix-Turn-Helix Protein
1323.	CFAP92	Cilia And Flagella Associated Protein 92 (Putative)
1324.	TINCR	TINCR Ubiquitin Domain Containing
1325.	BLACAT1	BLACAT1 Overlapping LEMD1 Locus
1326.	SMIM31	Small Integral Membrane Protein 31
1327.	KCNA3	Potassium Voltage-Gated Channel Subfamily A Member 3
1328.	MYL4	Myosin Light Chain 4
1329.	FAM13A	Family With Sequence Similarity 13 Member A
1330.	MAP3K8	Mitogen-Activated Protein Kinase Kinase Kinase 8
1331.	DNASE1L3	Deoxyribonuclease 1 Like 3
1332.	NNT	Nicotinamide Nucleotide Transhydrogenase
1333.	CRHR2	Corticotropin Releasing Hormone Receptor 2
1334.	CA3	Carbonic Anhydrase 3
1335.	CD59	CD59 Molecule (CD59 Blood Group)
1336.	IREB2	Iron Responsive Element Binding Protein 2
1337.	KRT20	Keratin 20
1338.	RPS14	Ribosomal Protein S14
1339.	FANCB	FA Complementation Group B
1340.	RARB	Retinoic Acid Receptor Beta
1341.	LGALS3	Galectin 3
1342.	CEACAM5	CEA Cell Adhesion Molecule 5
1343.	GCG	Glucagon
1344.	WAS	WASP Actin Nucleation Promoting Factor
1345.	TYK2	Tyrosine Kinase 2
1346.	CACNA1C	Calcium Voltage-Gated Channel Subunit Alpha1 C

1347.	AVP	Arginine Vasopressin
1348.	FURIN	Furin, Paired Basic Amino Acid Cleaving Enzyme
1349.	MTNR1B	Melatonin Receptor 1B
1350.	CRPPA	CDP-L-Ribitol Pyrophosphorylase A
1351.	SCN8A	Sodium Voltage-Gated Channel Alpha Subunit 8
1352.	OTX2	Orthodenticle Homeobox 2
1353.	CYP27B1	Cytochrome P450 Family 27 Subfamily B Member 1
1354.	HSD11B2	Hydroxysteroid 11-Beta Dehydrogenase 2
1355.	PRKCA	Protein Kinase C Alpha
1356.	MMP3	Matrix Metallopeptidase 3
1357.	CD55	CD55 Molecule (Cromer Blood Group)
1358.	CNTF	Ciliary Neurotrophic Factor
1359.	ITGAX	Integrin Subunit Alpha X
1360.	ACVR1B	Activin A Receptor Type 1B
1361.	FGF14	Fibroblast Growth Factor 14
1362.	STK33	Serine/Threonine Kinase 33
1363.	AAR2	AAR2 Splicing Factor
1364.	MYRF	Myelin Regulatory Factor
1365.	TMEM71	Transmembrane Protein 71
1366.	TICAM1	TIR Domain Containing Adaptor Molecule 1
1367.	DOLK	Dolichol Kinase
1368.	TOR1A	Torsin Family 1 Member A
1369.	CD300C	CD300c Molecule
1370.	GYG1	Glycogenin 1
1371.	ABCB11	ATP Binding Cassette Subfamily B Member 11
1372.	KLF1	KLF Transcription Factor 1
1373.	ABCE1	ATP Binding Cassette Subfamily E Member 1
1374.	GDNF	Glial Cell Derived Neurotrophic Factor
1375.	NDUFA11	NADH:Ubiquinone Oxidoreductase Subunit A11
1376.	KCNK9	Potassium Two Pore Domain Channel Subfamily K Member 9
1377.	NR0B1	Nuclear Receptor Subfamily 0 Group B Member 1
1378.	SCN2B	Sodium Voltage-Gated Channel Beta Subunit 2
1379.	SCN3B	Sodium Voltage-Gated Channel Beta Subunit 3
1380.	XPA	XPA, DNA Damage Recognition And Repair Factor
1381.	KCNE2	Potassium Voltage-Gated Channel Subfamily E Regulatory Subunit 2
1382.	FAN1	FANCD2 And FANCI Associated Nuclease 1
1383.	BCS1L	BCS1 Homolog, Ubiquinol-Cytochrome C Reductase Complex Chaperone
1384.	KRT19	Keratin 19
1385.	DCN	Decorin
1386.	HPRT1	Hypoxanthine Phosphoribosyltransferase 1
1387.	CD22	CD22 Molecule

1388.	GSTM1	Glutathione S-Transferase Mu 1
1389.	PARK7	Parkinsonism Associated Deglycase
1390.	E2F1	E2F Transcription Factor 1
1391.	CXCR5	C-X-C Motif Chemokine Receptor 5
1392.	HDC	Histidine Decarboxylase
1393.	PDLIM3	PDZ And LIM Domain 3
1394.	PLG	Plasminogen
1395.	TNFSF11	TNF Superfamily Member 11
1396.	VIM	Vimentin
1397.	HNRNPDL	Heterogeneous Nuclear Ribonucleoprotein D Like
1398.	IMPDH2	Inosine Monophosphate Dehydrogenase 2
1399.	SPTLC1	Serine Palmitoyltransferase Long Chain Base Subunit 1
1400.	TRIM32	Tripartite Motif Containing 32
1401.	SSB	Small RNA Binding Exonuclease Protection Factor La
1402.	GHR	Growth Hormone Receptor
1403.	TACR1	Tachykinin Receptor 1
1404.	CHRNB2	Cholinergic Receptor Nicotinic Beta 2 Subunit
1405.	TCF7L2	Transcription Factor 7 Like 2
1406.	RPS24	Ribosomal Protein S24
1407.	RPL35A	Ribosomal Protein L35a
1408.	NDUFAF1	NADH:Ubiquinone Oxidoreductase Complex Assembly Factor 1
1409.	NEBL	Nebulette
1410.	PRCC	Proline Rich Mitotic Checkpoint Control Factor
1411.	TSR2	TSR2 Ribosome Maturation Factor
1412.	HDAC1	Histone Deacetylase 1
1413.	MX1	MX Dynamin Like GTPase 1
1414.	RPL36A-HNRNPH2	RPL36A-HNRNPH2 Readthrough
1415.	LMOD3	Leiomodin 3
1416.	ITGA5	Integrin Subunit Alpha 5
1417.	XPR1	Xenotropic And Polytropic Retrovirus Receptor 1
1418.	SGO1	Shugoshin 1
1419.	CCND3	Cyclin D3
1420.	ANKRD2	Ankyrin Repeat Domain 2
1421.	SELENOP	Selenoprotein P
1422.	MOK	MOK Protein Kinase
1423.	ANKRD23	Ankyrin Repeat Domain 23
1424.	XDH	Xanthine Dehydrogenase
1425.	PTHLH	Parathyroid Hormone Like Hormone
1426.	ITGB3	Integrin Subunit Beta 3
1427.	SERPINC1	Serpin Family C Member 1
1428.	FOXO3	Forkhead Box O3

1429.	SLC25A26	Solute Carrier Family 25 Member 26
1430.	ATP1A1	ATPase Na+/K+ Transporting Subunit Alpha 1
1431.	LCK	LCK Proto-Oncogene, Src Family Tyrosine Kinase
1432.	HLA-C	Major Histocompatibility Complex, Class I, C
1433.	BCL2A1	BCL2 Related Protein A1
1434.	COL3A1	Collagen Type III Alpha 1 Chain
1435.	PHEX	Phosphate Regulating Endopeptidase X-Linked
1436.	ABCB7	ATP Binding Cassette Subfamily B Member 7
1437.	OXT	Oxytocin/Neurophysin I Prepropeptide
1438.	DDC	Dopa Decarboxylase
1439.	H6PD	Hexose-6-Phosphate Dehydrogenase/Glucose 1-Dehydrogenase
1440.	NAGLU	N-Acetyl-Alpha-Glucosaminidase
1441.	LAMA4	Laminin Subunit Alpha 4
1442.	NUP155	Nucleoporin 155
1443.	SCN4B	Sodium Voltage-Gated Channel Beta Subunit 4
1444.	KIF23	Kinesin Family Member 23
1445.	MMADHC	Metabolism Of Cobalamin Associated D
1446.	PTCH1	Patched 1
1447.	MIF	Macrophage Migration Inhibitory Factor
1448.	TXN	Thioredoxin
1449.	SLC20A2	Solute Carrier Family 20 Member 2
1450.	TBXAS1	Thromboxane A Synthase 1
1451.	MLKL	Mixed Lineage Kinase Domain Like Pseudokinase
1452.	TBX18	T-Box Transcription Factor 18
1453.	ERCC8	ERCC Excision Repair 8, CSA Ubiquitin Ligase Complex Subunit
1454.	RINT1	RAD50 Interactor 1
1455.	LY86	Lymphocyte Antigen 86
1456.	ZAP70	Zeta Chain Of T Cell Receptor Associated Protein Kinase 70
1457.	ENO1	Enolase 1
1458.	CALB2	Calbindin 2
1459.	PINK1	PTEN Induced Kinase 1
1460.	MYL9	Myosin Light Chain 9
1461.	NPAS2	Neuronal PAS Domain Protein 2
1462.	SESN3	Sestrin 3
1463.	SARDH	Sarcosine Dehydrogenase
1464.	HMGA2	High Mobility Group AT-Hook 2
1465.	VTN	Vitronectin
1466.	PUS1	Pseudouridine Synthase 1
1467.	TLR5	Toll Like Receptor 5
1468.	CD2	CD2 Molecule
1469.	CTSG	Cathepsin G

1470.	ADORA3	Adenosine A3 Receptor
1471.	EPX	Eosinophil Peroxidase
1472.	MTUS2	Microtubule Associated Scaffold Protein 2
1473.	MYOD1	Myogenic Differentiation 1
1474.	ADRA2A	Adrenoceptor Alpha 2A
1475.	PKM	Pyruvate Kinase M1/2
1476.	EP300	E1A Binding Protein P300
1477.	GLI1	GLI Family Zinc Finger 1
1478.	IRF3	Interferon Regulatory Factor 3
1479.	ETFA	Electron Transfer Flavoprotein Subunit Alpha
1480.	SURF1	SURF1 Cytochrome C Oxidase Assembly Factor
1481.	DPAGT1	Dolichyl-Phosphate N-Acetylglucosaminephosphotransferase 1
1482.	CHRNA2	Cholinergic Receptor Nicotinic Alpha 2 Subunit
1483.	GABRG2	Gamma-Aminobutyric Acid Type A Receptor Subunit Gamma2
1484.	SMARCAL1	SWI/SNF Related, Matrix Associated, Actin Dependent Regulator Of Chromatin, Subfamily A Like 1
1485.	DRD5	Dopamine Receptor D5
1486.	C1QA	Complement C1q A Chain
1487.	NONO	Non-POU Domain Containing Octamer Binding
1488.	NDUFS2	NADH:Ubiquinone Oxidoreductase Core Subunit S2
1489.	RPS10	Ribosomal Protein S10
1490.	DEPDC5	DEP Domain Containing 5, GATOR1 Subcomplex Subunit
1491.	KCNT1	Potassium Sodium-Activated Channel Subfamily T Member 1
1492.	RPL26	Ribosomal Protein L26
1493.	RPS27	Ribosomal Protein S27
1494.	TSFM	Ts Translation Elongation Factor, Mitochondrial
1495.	FANCF	FA Complementation Group F
1496.	IFT140	Intraflagellar Transport 140
1497.	IFT172	Intraflagellar Transport 172
1498.	ASPSCR1	ASPSCR1 Tether For SLC2A4, UBX Domain Containing
1499.	EFL1	Elongation Factor Like GTPase 1
1500.	RPS28	Ribosomal Protein S28
1501.	CTC1	CST Telomere Replication Complex Component 1
1502.	FAM111B	FAM111 Trypsin Like Peptidase B
1503.	GPR151	G Protein-Coupled Receptor 151
1504.	CCDC183	Coiled-Coil Domain Containing 183
1505.	CXCL13	C-X-C Motif Chemokine Ligand 13
1506.	ITGA2B	Integrin Subunit Alpha 2b
1507.	TCL1A	TCL1 Family AKT Coactivator A
1508.	IL4R	Interleukin 4 Receptor
1509.	GRIK2	Glutamate Ionotropic Receptor Kainate Type Subunit 2
1510.	FCN1	Ficolin 1

1511.	GRIN2B	Glutamate Ionotropic Receptor NMDA Type Subunit 2B
1512.	FLT4	Fms Related Receptor Tyrosine Kinase 4
1513.	FBXL4	F-Box And Leucine Rich Repeat Protein 4
1514.	MDH2	Malate Dehydrogenase 2
1515.	BLNK	B Cell Linker
1516.	TAF15	TATA-Box Binding Protein Associated Factor 15
1517.	IGLL1	Immunoglobulin Lambda Like Polypeptide 1
1518.	NKX2-6	NK2 Homeobox 6
1519.	CFAP410	Cilia And Flagella Associated Protein 410
1520.	IGKC	Immunoglobulin Kappa Constant
1521.	SUPT20H	SPT20 Homolog, SAGA Complex Component
1522.	F8	Coagulation Factor VIII
1523.	NGFR	Nerve Growth Factor Receptor
1524.	EWSR1	EWS RNA Binding Protein 1
1525.	PF4	Platelet Factor 4
1526.	NR3C2	Nuclear Receptor Subfamily 3 Group C Member 2
1527.	ATP12A	ATPase H+/K+ Transporting Non-Gastric Alpha2 Subunit
1528.	IFNA17	Interferon Alpha 17
1529.	IFNA16	Interferon Alpha 16
1530.	CYP11A1	Cytochrome P450 Family 11 Subfamily A Member 1
1531.	MAG	Myelin Associated Glycoprotein
1532.	BMI1	BMI1 Proto-Oncogene, Polycomb Ring Finger
1533.	YARS2	Tyrosyl-TRNA Synthetase 2
1534.	KCNA1	Potassium Voltage-Gated Channel Subfamily A Member 1
1535.	TSPAN1	Tetraspanin 1
1536.	GNE	Glucosamine (UDP-N-Acetyl)-2-Epimerase/N-Acetylmannosamine Kinase
1537.	PIGT	Phosphatidylinositol Glycan Anchor Biosynthesis Class T
1538.	VEGFC	Vascular Endothelial Growth Factor C
1539.	IFNA10	Interferon Alpha 10
1540.	ERCC3	ERCC Excision Repair 3, TFIIH Core Complex Helicase Subunit
1541.	FOXA2	Forkhead Box A2
1542.	DDB2	Damage Specific DNA Binding Protein 2
1543.	C1QBP	Complement C1q Binding Protein
1544.	UBQLN2	Ubiquilin 2
1545.	NAGS	N-Acetylglutamate Synthase
1546.	PROKR2	Prokineticin Receptor 2
1547.	ADORA2A	Adenosine A2a Receptor
1548.	LRP2	LDL Receptor Related Protein 2
1549.	BSCL2	BSCL2 Lipid Droplet Biogenesis Associated, Seipin
1550.	FHL1	Four And A Half LIM Domains 1
1551.	COL6A3	Collagen Type VI Alpha 3 Chain

1552.	ATP1A3	ATPase Na+/K+ Transporting Subunit Alpha 3
1553.	SET	SET Nuclear Proto-Oncogene
1554.	CYP51A1	Cytochrome P450 Family 51 Subfamily A Member 1
1555.	FOXO1	Forkhead Box O1
1556.	PLK1	Polo Like Kinase 1
1557.	HSPA8	Heat Shock Protein Family A (Hsp70) Member 8
1558.	RAB5A	RAB5A, Member RAS Oncogene Family
1559.	CCL18	C-C Motif Chemokine Ligand 18
1560.	IFNA6	Interferon Alpha 6
1561.	IFNA21	Interferon Alpha 21
1562.	IFNA8	Interferon Alpha 8
1563.	IFNA4	Interferon Alpha 4
1564.	IFNA13	Interferon Alpha 13
1565.	IFNA7	Interferon Alpha 7
1566.	JAK3	Janus Kinase 3
1567.	DICER1	Dicer 1, Ribonuclease III
1568.	TNFRSF25	TNF Receptor Superfamily Member 25
1569.	ZRSR2	Zinc Finger CCCH-Type, RNA Binding Motif And Serine/Arginine Rich 2
1570.	HNRNPA2B1	Heterogeneous Nuclear Ribonucleoprotein A2/B1
1571.	NES	Nestin
1572.	MTUS1	Microtubule Associated Scaffold Protein 1
1573.	CDH2	Cadherin 2
1574.	TGM2	Transglutaminase 2
1575.	ACP5	Acid Phosphatase 5, Tartrate Resistant
1576.	RBP4	Retinol Binding Protein 4
1577.	SLC37A4	Solute Carrier Family 37 Member 4
1578.	LRP4	LDL Receptor Related Protein 4
1579.	PER3	Period Circadian Regulator 3
1580.	LOX	Lysyl Oxidase
1581.	AQP2	Aquaporin 2
1582.	PLA2G6	Phospholipase A2 Group VI
1583.	CD58	CD58 Molecule
1584.	TRAF1	TNF Receptor Associated Factor 1
1585.	AIFM1	Apoptosis Inducing Factor Mitochondria Associated 1
1586.	TRPV4	Transient Receptor Potential Cation Channel Subfamily V Member 4
1587.	PPP2R1A	Protein Phosphatase 2 Scaffold Subunit Aalpha
1588.	COL4A4	Collagen Type IV Alpha 4 Chain
1589.	GMPPA	GDP-Mannose Pyrophosphorylase A
1590.	ALDH4A1	Aldehyde Dehydrogenase 4 Family Member A1
1591.	AHCY	Adenosylhomocysteinase
1592.	CARTPT	CART Prepropeptide

1593.	ATP5MF	ATP Synthase Membrane Subunit F
1594.	SSTR2	Somatostatin Receptor 2
1595.	HSP90AB1	Heat Shock Protein 90 Alpha Family Class B Member 1
1596.	BECN1	Beclin 1
1597.	PYY	Peptide YY
1598.	TPH1	Tryptophan Hydroxylase 1
1599.	YWHAE	Tyrosine 3-Monooxygenase/Tryptophan 5-Monooxygenase Activation Protein Epsilon
1600.	MITF	Melanocyte Inducing Transcription Factor
1601.	CDK12	Cyclin Dependent Kinase 12
1602.	CYP2A6	Cytochrome P450 Family 2 Subfamily A Member 6
1603.	IGFBP7	Insulin Like Growth Factor Binding Protein 7
1604.	UQCRFS1	Ubiquinol-Cytochrome C Reductase, Rieske Iron-Sulfur Polypeptide 1
1605.	EXT2	Exostosin Glycosyltransferase 2
1606.	IGF2BP2	Insulin Like Growth Factor 2 MRNA Binding Protein 2
1607.	NDUFB9	NADH:Ubiquinone Oxidoreductase Subunit B9
1608.	PRMT7	Protein Arginine Methyltransferase 7
1609.	UBE2T	Ubiquitin Conjugating Enzyme E2 T
1610.	EYA4	EYA Transcriptional Coactivator And Phosphatase 4
1611.	MIB1	MIB E3 Ubiquitin Protein Ligase 1
1612.	MYO7A	Myosin VIIA
1613.	NDUFA1	NADH:Ubiquinone Oxidoreductase Subunit A1
1614.	RPL15	Ribosomal Protein L15
1615.	TCF7	Transcription Factor 7
1616.	SRP54	Signal Recognition Particle 54
1617.	CDKAL1	CDK5 Regulatory Subunit Associated Protein 1 Like 1
1618.	RPS29	Ribosomal Protein S29
1619.	PLAG1	PLAG1 Zinc Finger
1620.	RFWD3	Ring Finger And WD Repeat Domain 3
1621.	RPL31	Ribosomal Protein L31
1622.	SRA1	Steroid Receptor RNA Activator 1
1623.	NDUFAF6	NADH:Ubiquinone Oxidoreductase Complex Assembly Factor 6
1624.	DMTF1	Cyclin D Binding Myb Like Transcription Factor 1
1625.	MYH7B	Myosin Heavy Chain 7B
1626.	C12orf43	Chromosome 12 Open Reading Frame 43
1627.	MMP1	Matrix Metallopeptidase 1
1628.	MMP14	Matrix Metallopeptidase 14
1629.	KNG1	Kininogen 1
1630.	S100A9	S100 Calcium Binding Protein A9
1631.	ACP1	Acid Phosphatase 1
1632.	PTPN6	Protein Tyrosine Phosphatase Non-Receptor Type 6
1633.	CALML3	Calmodulin Like 3

1634.	CTSD	Cathepsin D
1635.	FGF8	Fibroblast Growth Factor 8
1636.	CDKN1C	Cyclin Dependent Kinase Inhibitor 1C
1637.	IL3RA	Interleukin 3 Receptor Subunit Alpha
1638.	PIK3C3	Phosphatidylinositol 3-Kinase Catalytic Subunit Type 3
1639.	APAF1	Apoptotic Peptidase Activating Factor 1
1640.	F9	Coagulation Factor IX
1641.	CDK1	Cyclin Dependent Kinase 1
1642.	MYLK	Myosin Light Chain Kinase
1643.	CNBP	CCHC-Type Zinc Finger Nucleic Acid Binding Protein
1644.	GYPA	Glycophorin A (MNS Blood Group)
1645.	COX10	Cytochrome C Oxidase Assembly Factor Heme A:Farnesyltransferase COX10
1646.	PGF	Placental Growth Factor
1647.	NAB2	NGFI-A Binding Protein 2
1648.	KLRC4	Killer Cell Lectin Like Receptor C4
1649.	SLC29A1	Solute Carrier Family 29 Member 1 (Augustine Blood Group)
1650.	HDAC2	Histone Deacetylase 2
1651.	CTAG1B	Cancer/Testis Antigen 1B
1652.	RNASE3	Ribonuclease A Family Member 3
1653.	STOM	Stomatin
1654.	DIABLO	Diablo IAP-Binding Mitochondrial Protein
1655.	PNPLA3	Patatin Like Phospholipase Domain Containing 3
1656.	BIN1	Bridging Integrator 1
1657.	GNAI1	G Protein Subunit Alpha I1
1658.	INHBB	Inhibin Subunit Beta B
1659.	NMUR1	Neuromedin U Receptor 1
1660.	SESN1	Sestrin 1
1661.	SLC29A2	Solute Carrier Family 29 Member 2
1662.	CLU	Clusterin
1663.	CD70	CD70 Molecule
1664.	ANGPT1	Angiopoietin 1
1665.	SORL1	Sortilin Related Receptor 1
1666.	JAG2	Jagged Canonical Notch Ligand 2
1667.	FOXP1	Forkhead Box P1
1668.	RACGAP1	Rac GTPase Activating Protein 1
1669.	GATAD1	GATA Zinc Finger Domain Containing 1
1670.	ATP4A	ATPase H+/K+ Transporting Subunit Alpha
1671.	HBS1L	HBS1 Like Translational GTPase
1672.	SLC10A1	Solute Carrier Family 10 Member 1
1673.	LDLR	Low Density Lipoprotein Receptor
1674.	LEF1	Lymphoid Enhancer Binding Factor 1

1675.	ATG5	Autophagy Related 5
1676.	NR5A1	Nuclear Receptor Subfamily 5 Group A Member 1
1677.	CALM3	Calmodulin 3
1678.	CALML4	Calmodulin Like 4
1679.	ITK	IL2 Inducible T Cell Kinase
1680.	PECAM1	Platelet And Endothelial Cell Adhesion Molecule 1
1681.	RO60	Ro60, Y RNA Binding Protein
1682.	LAMB2	Laminin Subunit Beta 2
1683.	LIG3	DNA Ligase 3
1684.	NARS2	Asparaginyl-TRNA Synthetase 2, Mitochondrial
1685.	ALG2	ALG2 Alpha-1,3/1,6-Mannosyltransferase
1686.	BCHE	Butyrylcholinesterase
1687.	MMP12	Matrix Metallopeptidase 12
1688.	GP2	Glycoprotein 2
1689.	LCN2	Lipocalin 2
1690.	G6PC1	Glucose-6-Phosphatase Catalytic Subunit 1
1691.	NR1H4	Nuclear Receptor Subfamily 1 Group H Member 4
1692.	ACADS	Acyl-CoA Dehydrogenase Short Chain
1693.	H2AX	H2A.X Variant Histone
1694.	PRKCB	Protein Kinase C Beta
1695.	CACNA1D	Calcium Voltage-Gated Channel Subunit Alpha1 D
1696.	KIF5A	Kinesin Family Member 5A
1697.	BMP4	Bone Morphogenetic Protein 4
1698.	ALOX5	Arachidonate 5-Lipoxygenase
1699.	SP1	Sp1 Transcription Factor
1700.	ETFB	Electron Transfer Flavoprotein Subunit Beta
1701.	SLC9A1	Solute Carrier Family 9 Member A1
1702.	SMN2	Survival Of Motor Neuron 2, Centromeric
1703.	DYNC1H1	Dynein Cytoplasmic 1 Heavy Chain 1
1704.	POMT2	Protein O-Mannosyltransferase 2
1705.	DPM3	Dolichyl-Phosphate Mannosyltransferase Subunit 3, Regulatory
1706.	SERPINH1	Serpin Family H Member 1
1707.	CFL2	Cofilin 2
1708.	KLHL41	Kelch Like Family Member 41
1709.	HSPG2	Heparan Sulfate Proteoglycan 2
1710.	DNMT3B	DNA Methyltransferase 3 Beta
1711.	ANKK1	Ankyrin Repeat And Kinase Domain Containing 1
1712.	PCCB	Propionyl-CoA Carboxylase Subunit Beta
1713.	UGT1A9	UDP Glucuronosyltransferase Family 1 Member A9
1714.	FANCD2OS	FANCD2 Opposite Strand
1715.	APOA1	Apolipoprotein A1

1716.	HLA-G	Major Histocompatibility Complex, Class I, G
1717.	ADRA1A	Adrenoceptor Alpha 1A
1718.	ERVK-6	Endogenous Retrovirus Group K Member 6, Envelope
1719.	TRPV1	Transient Receptor Potential Cation Channel Subfamily V Member 1
1720.	EIF4E	Eukaryotic Translation Initiation Factor 4E
1721.	PLEKHM1	Pleckstrin Homology And RUN Domain Containing M1
1722.	MORC2	MORC Family CW-Type Zinc Finger 2
1723.	NTRK3	Neurotrophic Receptor Tyrosine Kinase 3
1724.	SPTAN1	Spectrin Alpha, Non-Erythrocytic 1
1725.	MKI67	Marker Of Proliferation Ki-67
1726.	MUC5AC	Mucin 5AC, Oligomeric Mucus/Gel-Forming
1727.	CALML5	Calmodulin Like 5
1728.	CALML6	Calmodulin Like 6
1729.	ADORA1	Adenosine A1 Receptor
1730.	ISG15	ISG15 Ubiquitin Like Modifier
1731.	PLA2G2A	Phospholipase A2 Group IIA
1732.	CTSB	Cathepsin B
1733.	WNK4	WNK Lysine Deficient Protein Kinase 4
1734.	CFHR1	Complement Factor H Related 1
1735.	TMEM70	Transmembrane Protein 70
1736.	STAC3	SH3 And Cysteine Rich Domain 3
1737.	CDA	Cytidine Deaminase
1738.	PLCG1	Phospholipase C Gamma 1
1739.	DAXX	Death Domain Associated Protein
1740.	FGF7	Fibroblast Growth Factor 7
1741.	H3-3B	H3.3 Histone B
1742.	SPN	Sialophorin
1743.	IFNA14	Interferon Alpha 14
1744.	CEACAM3	CEA Cell Adhesion Molecule 3
1745.	NEK9	NIMA Related Kinase 9
1746.	ADGRG6	Adhesion G Protein-Coupled Receptor G6
1747.	SETD2	SET Domain Containing 2, Histone Lysine Methyltransferase
1748.	FECH	Ferrochelatase
1749.	SERPINA3	Serpin Family A Member 3
1750.	PGR	Progesterone Receptor
1751.	CXCR1	C-X-C Motif Chemokine Receptor 1
1752.	PRKD1	Protein Kinase D1
1753.	UCP3	Uncoupling Protein 3
1754.	CD247	CD247 Molecule
1755.	NUP98	Nucleoporin 98 And 96 Precursor
1756.	BAK1	BCL2 Antagonist/Killer 1

1757.	GLB1	Galactosidase Beta 1
1758.	CHI3L1	Chitinase 3 Like 1
1759.	PDGFA	Platelet Derived Growth Factor Subunit A
1760.	SNRNP70	Small Nuclear Ribonucleoprotein U1 Subunit 70
1761.	CA2	Carbonic Anhydrase 2
1762.	MUC16	Mucin 16, Cell Surface Associated
1763.	CX3CR1	C-X3-C Motif Chemokine Receptor 1
1764.	DKK1	Dickkopf WNT Signaling Pathway Inhibitor 1
1765.	OSM	Oncostatin M
1766.	MS4A1	Membrane Spanning 4-Domains A1
1767.	CD209	CD209 Molecule
1768.	ENPP2	Ectonucleotide Pyrophosphatase/Phosphodiesterase 2
1769.	CDX2	Caudal Type Homeobox 2
1770.	CYP2B6	Cytochrome P450 Family 2 Subfamily B Member 6
1771.	PPIB	Peptidylprolyl Isomerase B
1772.	CD9	CD9 Molecule
1773.	NQO1	NAD(P)H Quinone Dehydrogenase 1
1774.	MEIS1	Meis Homeobox 1
1775.	RHD	Rh Blood Group D Antigen
1776.	CD7	CD7 Molecule
1777.	CYP2C19	Cytochrome P450 Family 2 Subfamily C Member 19
1778.	CCDC78	Coiled-Coil Domain Containing 78
1779.	PRKDC	Protein Kinase, DNA-Activated, Catalytic Subunit
1780.	PRKAG2	Protein Kinase AMP-Activated Non-Catalytic Subunit Gamma 2
1781.	HBEGF	Heparin Binding EGF Like Growth Factor
1782.	TNFRSF9	TNF Receptor Superfamily Member 9
1783.	PNMT	Phenylethanolamine N-Methyltransferase
1784.	SAA1	Serum Amyloid A1
1785.	IGSF1	Immunoglobulin Superfamily Member 1
1786.	HSPB3	Heat Shock Protein Family B (Small) Member 3
1787.	DUT	Deoxyuridine Triphosphatase
1788.	TRAP1	TNF Receptor Associated Protein 1
1789.	APLP1	Amyloid Beta Precursor Like Protein 1
1790.	CST7	Cystatin F
1791.	PDCD2	Programmed Cell Death 2
1792.	NIPA1	NIPA Magnesium Transporter 1
1793.	MGME1	Mitochondrial Genome Maintenance Exonuclease 1
1794.	NPC1	NPC Intracellular Cholesterol Transporter 1
1795.	STUB1	STIP1 Homology And U-Box Containing Protein 1
1796.	GTPBP1	GTP Binding Protein 1
1797.	HOXA9	Homeobox A9

1798.	H3C12	H3 Clustered Histone 12
1799.	H3-7	H3.7 Histone (Putative)
1800.	ATP1A2	ATPase Na+/K+ Transporting Subunit Alpha 2
1801.	IL1RAP	Interleukin 1 Receptor Accessory Protein
1802.	HBE1	Hemoglobin Subunit Epsilon 1
1803.	GSTP1	Glutathione S-Transferase Pi 1
1804.	CXADR	CXADR Ig-Like Cell Adhesion Molecule
1805.	IL10RA	Interleukin 10 Receptor Subunit Alpha
1806.	CYP24A1	Cytochrome P450 Family 24 Subfamily A Member 1
1807.	GIMAP5	GTPase, IMAP Family Member 5
1808.	SCN2A	Sodium Voltage-Gated Channel Alpha Subunit 2
1809.	GAD2	Glutamate Decarboxylase 2
1810.	PTX3	Pentraxin 3
1811.	AXL	AXL Receptor Tyrosine Kinase
1812.	CCNB1	Cyclin B1
1813.	PVR	PVR Cell Adhesion Molecule
1814.	ANK3	Ankyrin 3
1815.	NAT2	N-Acetyltransferase 2
1816.	NTS	Neurotensin
1817.	SCGB1A1	Secretoglobin Family 1A Member 1
1818.	RUNX1T1	RUNX1 Partner Transcriptional Co-Repressor 1
1819.	SIGMAR1	Sigma Non-Opioid Intracellular Receptor 1
1820.	RYR3	Ryanodine Receptor 3
1821.	SPEG	Striated Muscle Enriched Protein Kinase
1822.	SPTBN4	Spectrin Beta, Non-Erythrocytic 4
1823.	POMK	Protein O-Mannose Kinase
1824.	ODC1	Ornithine Decarboxylase 1
1825.	SSPN	Sarcospan
1826.	ESR2	Estrogen Receptor 2
1827.	IHH	Indian Hedgehog Signaling Molecule
1828.	NT5E	5'-Nucleotidase Ecto
1829.	KCNQ2	Potassium Voltage-Gated Channel Subfamily Q Member 2
1830.	C5AR1	Complement C5a Receptor 1
1831.	BPGM	Bisphosphoglycerate Mutase
1832.	CYP26A1	Cytochrome P450 Family 26 Subfamily A Member 1
1833.	IL22	Interleukin 22
1834.	RNPC3	RNA Binding Region (RNP1, RRM) Containing 3
1835.	GJA1	Gap Junction Protein Alpha 1
1836.	GRN	Granulin Precursor
1837.	OGG1	8-Oxoguanine DNA Glycosylase
1838.	TNFRSF13C	TNF Receptor Superfamily Member 13C

1839.	LGI1	Leucine Rich Glioma Inactivated 1
1840.	NTF3	Neurotrophin 3
1841.	PLAUR	Plasminogen Activator, Urokinase Receptor
1842.	SIRPA	Signal Regulatory Protein Alpha
1843.	SNAI1	Snail Family Transcriptional Repressor 1
1844.	CD1D	CD1d Molecule
1845.	BPI	Bactericidal Permeability Increasing Protein
1846.	CLEC4M	C-Type Lectin Domain Family 4 Member M
1847.	RAB8A	RAB8A, Member RAS Oncogene Family
1848.	CDAN1	Codanin 1
1849.	BLZF1	Basic Leucine Zipper Nuclear Factor 1
1850.	PXDNL	Peroxidasin Like
1851.	LGALS1	Galectin 1
1852.	SFTPB	Surfactant Protein B
1853.	CD177	CD177 Molecule
1854.	CD47	CD47 Molecule
1855.	IL12RB2	Interleukin 12 Receptor Subunit Beta 2
1856.	TBX1	T-Box Transcription Factor 1
1857.	LARGE1	LARGE Xylosyl- And Glucuronyltransferase 1
1858.	HDAC6	Histone Deacetylase 6
1859.	IKBKB	Inhibitor Of Nuclear Factor Kappa B Kinase Subunit Beta
1860.	AHSG	Alpha 2-HS Glycoprotein
1861.	MICB	MHC Class I Polypeptide-Related Sequence B
1862.	COL4A1	Collagen Type IV Alpha 1 Chain
1863.	LRBA	LPS Responsive Beige-Like Anchor Protein
1864.	SGCE	Sarcoglycan Epsilon
1865.	ACVRL1	Activin A Receptor Like Type 1
1866.	GNPTAB	N-Acetylglucosamine-1-Phosphate Transferase Subunits Alpha And Beta
1867.	SLAMF1	Signaling Lymphocytic Activation Molecule Family Member 1
1868.	AARS1	Alanyl-TRNA Synthetase 1
1869.	LAMA5	Laminin Subunit Alpha 5
1870.	PRKACB	Protein Kinase CAMP-Activated Catalytic Subunit Beta
1871.	CCN2	Cellular Communication Network Factor 2
1872.	PRKACG	Protein Kinase CAMP-Activated Catalytic Subunit Gamma
1873.	VDAC1	Voltage Dependent Anion Channel 1
1874.	PRDM1	PR/SET Domain 1
1875.	RUNX3	RUNX Family Transcription Factor 3
1876.	KIR3DL1	Killer Cell Immunoglobulin Like Receptor, Three Ig Domains And Long Cytoplasmic Tail 1
1877.	H3-4	H3.4 Histone, Cluster Member
1878.	H3C13	H3 Clustered Histone 13
1879.	H3-5	H3.5 Histone

1880.	SCN10A	Sodium Voltage-Gated Channel Alpha Subunit 10
1881.	MBTPS1	Membrane Bound Transcription Factor Peptidase, Site 1
1882.	PDP1	Pyruvate Dehydrogenase Phosphatase Catalytic Subunit 1
1883.	ANXA2	Annexin A2
1884.	ALAD	Aminolevulinate Dehydratase
1885.	HSD3B2	Hydroxy-Delta-5-Steroid Dehydrogenase, 3 Beta- And Steroid Delta-Isomerase 2
1886.	ATXN1	Ataxin 1
1887.	CEBPB	CCAAT Enhancer Binding Protein Beta
1888.	FCGRT	Fc Gamma Receptor And Transporter
1889.	THY1	Thy-1 Cell Surface Antigen
1890.	VPS35	VPS35 Retromer Complex Component
1891.	DNAJC19	DnaJ Heat Shock Protein Family (Hsp40) Member C19
1892.	HNRNPC	Heterogeneous Nuclear Ribonucleoprotein C
1893.	CTSK	Cathepsin K
1894.	TBX21	T-Box Transcription Factor 21
1895.	TAC1	Tachykinin Precursor 1
1896.	IL33	Interleukin 33
1897.	CENPB	Centromere Protein B
1898.	GIP	Gastric Inhibitory Polypeptide
1899.	ADSS1	Adenylosuccinate Synthase 1
1900.	PTK2B	Protein Tyrosine Kinase 2 Beta
1901.	RPS6KA3	Ribosomal Protein S6 Kinase A3
1902.	ROCK1	Rho Associated Coiled-Coil Containing Protein Kinase 1
1903.	DHCR7	7-Dehydrocholesterol Reductase
1904.	PLA2G4A	Phospholipase A2 Group IVA
1905.	GPD2	Glycerol-3-Phosphate Dehydrogenase 2
1906.	PCCA	Propionyl-CoA Carboxylase Subunit Alpha
1907.	GATM	Glycine Amidinotransferase
1908.	KIF5B	Kinesin Family Member 5B
1909.	NDUFS3	NADH:Ubiquinone Oxidoreductase Core Subunit S3
1910.	PIAS1	Protein Inhibitor Of Activated STAT 1
1911.	PPP2R1B	Protein Phosphatase 2 Scaffold Subunit Abeta
1912.	CORO1A	Coronin 1A
1913.	MAPK8IP1	Mitogen-Activated Protein Kinase 8 Interacting Protein 1
1914.	NLGN3	Neuroligin 3
1915.	TRPS1	Transcriptional Repressor GATA Binding 1
1916.	ADNP	Activity Dependent Neuroprotector Homeobox
1917.	APPL1	Adaptor Protein, Phosphotyrosine Interacting With PH Domain And Leucine Zipper 1
1918.	CANT1	Calcium Activated Nucleotidase 1
1919.	CLTC	Clathrin Heavy Chain
1920.	COL7A1	Collagen Type VII Alpha 1 Chain

1921.	NDUFA13	NADH:Ubiquinone Oxidoreductase Subunit A13
1922.	PFAS	Phosphoribosylformylglycinamidine Synthase
1923.	TBC1D4	TBC1 Domain Family Member 4
1924.	EHHADH	Enoyl-CoA Hydratase And 3-Hydroxyacyl CoA Dehydrogenase
1925.	EPHA5	EPH Receptor A5
1926.	FLII	FLII Actin Remodeling Protein
1927.	NDUFB10	NADH:Ubiquinone Oxidoreductase Subunit B10
1928.	NDUFS6	NADH:Ubiquinone Oxidoreductase Subunit S6
1929.	PPP1R3A	Protein Phosphatase 1 Regulatory Subunit 3A
1930.	RPL18	Ribosomal Protein L18
1931.	RPL35	Ribosomal Protein L35
1932.	SLCO2A1	Solute Carrier Organic Anion Transporter Family Member 2A1
1933.	GPSM2	G Protein Signaling Modulator 2
1934.	JAM2	Junctional Adhesion Molecule 2
1935.	NDUFAF4	NADH:Ubiquinone Oxidoreductase Complex Assembly Factor 4
1936.	PACS1	Phosphofurin Acidic Cluster Sorting Protein 1
1937.	POGZ	Pogo Transposable Element Derived With ZNF Domain
1938.	PTF1A	Pancreas Associated Transcription Factor 1a
1939.	RPL27	Ribosomal Protein L27
1940.	RPS15A	Ribosomal Protein S15a
1941.	HOXA13	Homeobox A13
1942.	KCNH8	Potassium Voltage-Gated Channel Subfamily H Member 8
1943.	MKKS	MKKS Centrosomal Shuttling Protein
1944.	MRTFA	Myocardin Related Transcription Factor A
1945.	NDUFAF2	NADH:Ubiquinone Oxidoreductase Complex Assembly Factor 2
1946.	PPP1R13L	Protein Phosphatase 1 Regulatory Subunit 13 Like
1947.	SLC25A10	Solute Carrier Family 25 Member 10
1948.	AOPEP	Aminopeptidase O (Putative)
1949.	FOXRED1	FAD Dependent Oxidoreductase Domain Containing 1
1950.	GPR65	G Protein-Coupled Receptor 65
1951.	INCENP	Inner Centromere Protein
1952.	PRSS21	Serine Protease 21
1953.	RPL8	Ribosomal Protein L8
1954.	SLMAP	Sarcolemma Associated Protein
1955.	TGM4	Transglutaminase 4
1956.	ADAMTS9	ADAM Metallopeptidase With Thrombospondin Type 1 Motif 9
1957.	INTU	Inturned Planar Cell Polarity Protein
1958.	NDUFAF3	NADH:Ubiquinone Oxidoreductase Complex Assembly Factor 3
1959.	RPL9	Ribosomal Protein L9
1960.	SLC30A5	Solute Carrier Family 30 Member 5
1961.	SRP19	Signal Recognition Particle 19

1962.	TECTA	Tectorin Alpha
1963.	TOE1	Target Of EGR1, Exonuclease
1964.	MIB2	MIB E3 Ubiquitin Protein Ligase 2
1965.	MYH8	Myosin Heavy Chain 8
1966.	NCAPH2	Non-SMC Condensin II Complex Subunit H2
1967.	RMND1	Required For Meiotic Nuclear Division 1 Homolog
1968.	CABP4	Calcium Binding Protein 4
1969.	CEP85L	Centrosomal Protein 85 Like
1970.	DLEC1	DLEC1 Cilia And Flagella Associated Protein
1971.	NDUFAF5	NADH:Ubiquinone Oxidoreductase Complex Assembly Factor 5
1972.	RBPJL	Recombination Signal Binding Protein For Immunoglobulin Kappa J Region Like
1973.	SHROOM2	Shroom Family Member 2
1974.	C1GALT1C1	C1GALT1 Specific Chaperone 1
1975.	CFHR4	Complement Factor H Related 4
1976.	LRFN4	Leucine Rich Repeat And Fibronectin Type III Domain Containing 4
1977.	MYORG	Myogenesis Regulating Glycosidase (Putative)
1978.	SRPRA	SRP Receptor Subunit Alpha
1979.	UGT1A3	UDP Glucuronosyltransferase Family 1 Member A3
1980.	ZNF276	Zinc Finger Protein 276
1981.	CPLANE1	Ciliogenesis And Planar Polarity Effector Complex Subunit 1
1982.	DNHD1	Dynein Heavy Chain Domain 1
1983.	FAM136A	Family With Sequence Similarity 136 Member A
1984.	RBM48	RNA Binding Motif Protein 48
1985.	IRX2	Iroquois Homeobox 2
1986.	IQCG	IQ Motif Containing G
1987.	PLEKHM2	Pleckstrin Homology And RUN Domain Containing M2
1988.	RLIG1	RNA 5'-Phosphate And 3'-OH Ligase 1
1989.	CCDC186	Coiled-Coil Domain Containing 186
1990.	FAM186B	Family With Sequence Similarity 186 Member B
1991.	HEATR3	HEAT Repeat Containing 3
1992.	MXRA5	Matrix Remodeling Associated 5
1993.	OTOG	Otogelin
1994.	UGT1A5	UDP Glucuronosyltransferase Family 1 Member A5
1995.	ZNF528	Zinc Finger Protein 528
1996.	MYZAP	Myocardial Zonula Adherens Protein
1997.	ZMAT1	Zinc Finger Matrin-Type 1
1998.	MILR1	Mast Cell Immunoglobulin Like Receptor 1
1999.	TEN1	TEN1 Subunit Of CST Complex
2000.	AKR7L	Aldo-Keto Reductase Family 7 Like (Gene/Pseudogene)
2001.	PGBD3	PiggyBac Transposable Element Derived 3
2002.	DRICH1	Aspartate Rich 1

2003.	GCOM1	GCOM1, MYZAP-POLR2M Combined Locus
2004.	NUTM2B	NUT Family Member 2B
2005.	IQANK1	IQ Motif And Ankyrin Repeat Containing 1
2006.	RPS10-NUDT3	RPS10-NUDT3 Readthrough
2007.	SMIM30	Small Integral Membrane Protein 30
2008.	TUNAR	TCL1 Upstream Neural Differentiation-Associated RNA
2009.	ERVH48-1	Endogenous Retrovirus Group 48 Member 1, Envelope
2010.	NUTM2E	NUT Family Member 2E
2011.	TMEM238L	Transmembrane Protein 238 Like
2012.	TBCEL-TECTA	TBCEL-TECTA Readthrough
2013.	MMP13	Matrix Metallopeptidase 13
2014.	FTH1	Ferritin Heavy Chain 1
2015.	WNK1	WNK Lysine Deficient Protein Kinase 1
2016.	C1S	Complement C1s
2017.	CALB1	Calbindin 1
2018.	TRPV5	Transient Receptor Potential Cation Channel Subfamily V Member 5
2019.	CLCNKA	Chloride Voltage-Gated Channel Ka
2020.	ATP8B1	ATPase Phospholipid Transporting 8B1
2021.	KLRD1	Killer Cell Lectin Like Receptor D1
2022.	SLC1A2	Solute Carrier Family 1 Member 2
2023.	BIRC2	Baculoviral IAP Repeat Containing 2
2024.	MTR	5-Methyltetrahydrofolate-Homocysteine Methyltransferase
2025.	TSPO	Translocator Protein
2026.	GRIN2A	Glutamate Ionotropic Receptor NMDA Type Subunit 2A
2027.	CDK5	Cyclin Dependent Kinase 5
2028.	UCHL1	Ubiquitin C-Terminal Hydrolase L1
2029.	PLAU	Plasminogen Activator, Urokinase
2030.	LEPR	Leptin Receptor
2031.	PRNP	Prion Protein (Kanno Blood Group)
2032.	ZEB1	Zinc Finger E-Box Binding Homeobox 1
2033.	GFRA1	GDNF Family Receptor Alpha 1
2034.	GJB2	Gap Junction Protein Beta 2
2035.	XRCC1	X-Ray Repair Cross Complementing 1
2036.	ATG7	Autophagy Related 7
2037.	PRAME	PRAME Nuclear Receptor Transcriptional Regulator
2038.	ADAR	Adenosine Deaminase RNA Specific
2039.	SLC10A2	Solute Carrier Family 10 Member 2
2040.	IL21R	Interleukin 21 Receptor
2041.	SLC19A1	Solute Carrier Family 19 Member 1
2042.	P2RX3	Purinergic Receptor P2X 3
2043.	SCARB1	Scavenger Receptor Class B Member 1

2044.	NLRP12	NLR Family Pyrin Domain Containing 12
2045.	SPG7	SPG7 Matrix AAA Peptidase Subunit, Paraplegin
2046.	GIPC1	GIPC PDZ Domain Containing Family Member 1
2047.	RNASEH1	Ribonuclease H1
2048.	EPHA4	EPH Receptor A4
2049.	XPC	XPC Complex Subunit, DNA Damage Recognition And Repair Factor
2050.	DAO	D-Amino Acid Oxidase
2051.	ERAP1	Endoplasmic Reticulum Aminopeptidase 1
2052.	ARNT2	Aryl Hydrocarbon Receptor Nuclear Translocator 2
2053.	DPP9	Dipeptidyl Peptidase 9
2054.	GLE1	GLE1 RNA Export Mediator
2055.	KPNA3	Karyopherin Subunit Alpha 3
2056.	SEMA4A	Semaphorin 4A
2057.	SLC25A11	Solute Carrier Family 25 Member 11
2058.	MLX	MAX Dimerization Protein MLX
2059.	PODXL	Podocalyxin Like
2060.	SLC39A7	Solute Carrier Family 39 Member 7
2061.	WIPF1	WAS/WASL Interacting Protein Family Member 1
2062.	CCNF	Cyclin F
2063.	PHIP	Pleckstrin Homology Domain Interacting Protein
2064.	PPCS	Phosphopantothenoylcysteine Synthetase
2065.	UNC93B1	Unc-93 Homolog B1, TLR Signaling Regulator
2066.	GLT8D1	Glycosyltransferase 8 Domain Containing 1
2067.	LRRC8A	Leucine Rich Repeat Containing 8 VRAC Subunit A
2068.	CAP2	Cyclase Associated Actin Cytoskeleton Regulatory Protein 2
2069.	UBAC2	UBA Domain Containing 2
2070.	BAG5	BAG Cochaperone 5
2071.	VEZF1	Vascular Endothelial Zinc Finger 1
2072.	TAF1A	TATA-Box Binding Protein Associated Factor, RNA Polymerase I Subunit A
2073.	IGHG2	Immunoglobulin Heavy Constant Gamma 2 (G2m Marker)
2074.	CTNNA1	Catenin Alpha 1
2075.	DDIT3	DNA Damage Inducible Transcript 3
2076.	GK	Glycerol Kinase
2077.	MOG	Myelin Oligodendrocyte Glycoprotein
2078.	PXN	Paxillin
2079.	SV2A	Synaptic Vesicle Glycoprotein 2A
2080.	FSCN1	Fascin Actin-Bundling Protein 1
2081.	POSTN	Periostin
2082.	RNASE2	Ribonuclease A Family Member 2
2083.	VEGFD	Vascular Endothelial Growth Factor D
2084.	CD1A	CD1a Molecule

2085.	MAVS	Mitochondrial Antiviral Signaling Protein
2086.	CLEC12A	C-Type Lectin Domain Family 12 Member A
2087.	SMPD1	Sphingomyelin Phosphodiesterase 1
2088.	MMP8	Matrix Metallopeptidase 8
2089.	CCR4	C-C Motif Chemokine Receptor 4
2090.	CCR2	C-C Motif Chemokine Receptor 2
2091.	HPSE	Heparanase
2092.	PPIG	Peptidylprolyl Isomerase G
2093.	TNFRSF6B	TNF Receptor Superfamily Member 6b
2094.	CXCL5	C-X-C Motif Chemokine Ligand 5
2095.	BMP1	Bone Morphogenetic Protein 1
2096.	WNT1	Wnt Family Member 1
2097.	CNTN2	Contactin 2
2098.	MARS1	Methionyl-TRNA Synthetase 1
2099.	COQ8A	Coenzyme Q8A
2100.	KLRC1	Killer Cell Lectin Like Receptor C1
2101.	ANGPT2	Angiopoietin 2
2102.	CYBB	Cytochrome B-245 Beta Chain
2103.	CYP11B1	Cytochrome P450 Family 11 Subfamily B Member 1
2104.	DLAT	Dihydrolipoamide S-Acetyltransferase
2105.	PANK2	Pantothenate Kinase 2
2106.	MGAM	Maltase-Glucoamylase
2107.	SRF	Serum Response Factor
2108.	YBX3	Y-Box Binding Protein 3
2109.	PTH1R	Parathyroid Hormone 1 Receptor
2110.	CYP2C9	Cytochrome P450 Family 2 Subfamily C Member 9
2111.	CD14	CD14 Molecule
2112.	CCNE1	Cyclin E1
2113.	KCNJ10	Potassium Inwardly Rectifying Channel Subfamily J Member 10
2114.	KLF4	KLF Transcription Factor 4
2115.	CCR3	C-C Motif Chemokine Receptor 3
2116.	RARG	Retinoic Acid Receptor Gamma
2117.	TGFA	Transforming Growth Factor Alpha
2118.	MAP2	Microtubule Associated Protein 2
2119.	TP73	Tumor Protein P73
2120.	RECK	Reversion Inducing Cysteine Rich Protein With Kazal Motifs
2121.	SERPINB1	Serpin Family B Member 1
2122.	NCR3	Natural Cytotoxicity Triggering Receptor 3
2123.	SLC25A37	Solute Carrier Family 25 Member 37
2124.	EVPL	Envoplakin
2125.	SLC25A28	Solute Carrier Family 25 Member 28

2126.	HRH2	Histamine Receptor H2
2127.	TCN2	Transcobalamin 2
2128.	PYROXD1	Pyridine Nucleotide-Disulphide Oxidoreductase Domain 1
2129.	RAC1	Rac Family Small GTPase 1
2130.	P2RY12	Purinergic Receptor P2Y12
2131.	HARS1	Histidyl-TRNA Synthetase 1
2132.	IGFBP2	Insulin Like Growth Factor Binding Protein 2
2133.	VAV1	Vav Guanine Nucleotide Exchange Factor 1
2134.	IL1RL2	Interleukin 1 Receptor Like 2
2135.	HTR1B	5-Hydroxytryptamine Receptor 1B
2136.	ESRRB	Estrogen Related Receptor Beta
2137.	ACADL	Acyl-CoA Dehydrogenase Long Chain
2138.	CD99	CD99 Molecule (Xg Blood Group)
2139.	DCK	Deoxycytidine Kinase
2140.	PPBP	Pro-Platelet Basic Protein
2141.	CYP1A1	Cytochrome P450 Family 1 Subfamily A Member 1
2142.	CNR1	Cannabinoid Receptor 1
2143.	ERFE	Erythroferrone
2144.	ADSL	Adenylosuccinate Lyase
2145.	AVPR2	Arginine Vasopressin Receptor 2
2146.	CCKAR	Cholecystokinin A Receptor
2147.	STX16	Syntaxin 16
2148.	GPC3	Glypican 3
2149.	NPHS1	NPHS1 Adhesion Molecule, Nephrin
2150.	TNFRSF11A	TNF Receptor Superfamily Member 11a
2151.	DBT	Dihydrolipoamide Branched Chain Transacylase E2
2152.	HAX1	HCLS1 Associated Protein X-1
2153.	MFN1	Mitofusin 1
2154.	PAM	Peptidylglycine Alpha-Amidating Monooxygenase
2155.	S100A8	S100 Calcium Binding Protein A8
2156.	MPV17	Mitochondrial Inner Membrane Protein MPV17
2157.	TIMP2	TIMP Metallopeptidase Inhibitor 2
2158.	ITGAE	Integrin Subunit Alpha E
2159.	YBX1	Y-Box Binding Protein 1
2160.	CLN3	CLN3 Lysosomal/Endosomal Transmembrane Protein, Battenin
2161.	MCOLN1	Mucolipin TRP Cation Channel 1
2162.	FGFR4	Fibroblast Growth Factor Receptor 4
2163.	CDC42	Cell Division Cycle 42
2164.	RAD50	RAD50 Double Strand Break Repair Protein
2165.	LIG1	DNA Ligase 1
2166.	PLIN1	Perilipin 1

2167.	APEX1	Apurinic/Apyrimidinic Endodeoxyribonuclease 1
2168.	ABCC3	ATP Binding Cassette Subfamily C Member 3
2169.	ADD1	Adducin 1
2170.	ISL1	ISL LIM Homeobox 1
2171.	SULT2A1	Sulfotransferase Family 2A Member 1
2172.	AIM2	Absent In Melanoma 2
2173.	SP100	SP100 Nuclear Antigen
2174.	DMBT1	Deleted In Malignant Brain Tumors 1
2175.	OPA3	Outer Mitochondrial Membrane Lipid Metabolism Regulator OPA3
2176.	TIMM50	Translocase Of Inner Mitochondrial Membrane 50
2177.	SERAC1	Serine Active Site Containing 1
2178.	TAMM41	TAM41 Mitochondrial Translocator Assembly And Maintenance Homolog
2179.	APOO	Apolipoprotein O
2180.	MICOS13	Mitochondrial Contact Site And Cristae Organizing System Subunit 13
2181.	SLC12A6	Solute Carrier Family 12 Member 6
2182.	MMP7	Matrix Metallopeptidase 7
2183.	SLC9A3	Solute Carrier Family 9 Member A3
2184.	POU5F1	POU Class 5 Homeobox 1
2185.	XBP1	X-Box Binding Protein 1
2186.	ADM	Adrenomedullin
2187.	NME1	NME/NM23 Nucleoside Diphosphate Kinase 1
2188.	UROD	Uroporphyrinogen Decarboxylase
2189.	GC	GC Vitamin D Binding Protein
2190.	HMMR	Hyaluronan Mediated Motility Receptor
2191.	PXDN	Peroxidasin
2192.	SMAD7	SMAD Family Member 7
2193.	FBXO7	F-Box Protein 7
2194.	GAP43	Growth Associated Protein 43
2195.	CYP7A1	Cytochrome P450 Family 7 Subfamily A Member 1
2196.	NOX4	NADPH Oxidase 4
2197.	CRHBP	Corticotropin Releasing Hormone Binding Protein
2198.	GFI1	Growth Factor Independent 1 Transcriptional Repressor
2199.	IAPP	Islet Amyloid Polypeptide
2200.	TRDN	Triadin
2201.	LAMP3	Lysosomal Associated Membrane Protein 3
2202.	TMBIM4	Transmembrane BAX Inhibitor Motif Containing 4
2203.	BCL2L2	BCL2 Like 2
2204.	ITGA2	Integrin Subunit Alpha 2
2205.	IFNGR2	Interferon Gamma Receptor 2
2206.	CCR6	C-C Motif Chemokine Receptor 6
2207.	CD1C	CD1c Molecule

2208.	HSD11B1	Hydroxysteroid 11-Beta Dehydrogenase 1
2209.	AGXT	Alanine--Glyoxylate Aminotransferase
2210.	TJP1	Tight Junction Protein 1
2211.	POLR2L	RNA Polymerase II, I And III Subunit L
2212.	F12	Coagulation Factor XII
2213.	CFP	Complement Factor Properdin
2214.	COL6A1	Collagen Type VI Alpha 1 Chain
2215.	COQ5	Coenzyme Q5, Methyltransferase
2216.	GATA3	GATA Binding Protein 3
2217.	NCL	Nucleolin
2218.	GPX3	Glutathione Peroxidase 3
2219.	HLA-E	Major Histocompatibility Complex, Class I, E
2220.	CDK9	Cyclin Dependent Kinase 9
2221.	PAWR	Pro-Apoptotic WT1 Regulator
2222.	TCN1	Transcobalamin 1
2223.	ANKRD26	Ankyrin Repeat Domain Containing 26
2224.	PAX6	Paired Box 6
2225.	TARS1	Threonyl-TRNA Synthetase 1
2226.	HNRNPH2	Heterogeneous Nuclear Ribonucleoprotein H2
2227.	NRP1	Neuropilin 1
2228.	UCP2	Uncoupling Protein 2
2229.	EPHA7	EPH Receptor A7
2230.	DDX20	DEAD-Box Helicase 20
2231.	SKAP1	Src Kinase Associated Phosphoprotein 1
2232.	ISOC1	Isochorismatase Domain Containing 1
2233.	C1orf162	Chromosome 1 Open Reading Frame 162
2234.	INKA2	Inka Box Actin Regulator 2
2235.	KRTAP4-6	Keratin Associated Protein 4-6
2236.	KRTAP4-5	Keratin Associated Protein 4-5
2237.	TMIGD3	Transmembrane And Immunoglobulin Domain Containing 3
2238.	HEXB	Hexosaminidase Subunit Beta
2239.	MSH3	MutS Homolog 3
2240.	F10	Coagulation Factor X
2241.	PRKG1	Protein Kinase CGMP-Dependent 1
2242.	GABBR1	Gamma-Aminobutyric Acid Type B Receptor Subunit 1
2243.	F2R	Coagulation Factor II Thrombin Receptor
2244.	KIR2DL1	Killer Cell Immunoglobulin Like Receptor, Two Ig Domains And Long Cytoplasmic Tail 1
2245.	ARID1A	AT-Rich Interaction Domain 1A
2246.	AP3B1	Adaptor Related Protein Complex 3 Subunit Beta 1
2247.	HNRNPK	Heterogeneous Nuclear Ribonucleoprotein K
2248.	SLPI	Secretory Leukocyte Peptidase Inhibitor

2249.	KLRG1	Killer Cell Lectin Like Receptor G1
2250.	KCNH2	Potassium Voltage-Gated Channel Subfamily H Member 2
2251.	NF2	NF2, Moesin-Ezrin-Radixin Like (MERLIN) Tumor Suppressor
2252.	PNP	Purine Nucleoside Phosphorylase
2253.	SLC1A3	Solute Carrier Family 1 Member 3
2254.	F7	Coagulation Factor VII
2255.	TP63	Tumor Protein P63
2256.	MYL3	Myosin Light Chain 3
2257.	CX3CL1	C-X3-C Motif Chemokine Ligand 1
2258.	CXCL1	C-X-C Motif Chemokine Ligand 1
2259.	GIPR	Gastric Inhibitory Polypeptide Receptor
2260.	IL9	Interleukin 9
2261.	RHOT1	Ras Homolog Family Member T1
2262.	CLPX	Caseinolytic Mitochondrial Matrix Peptidase Chaperone Subunit X
2263.	IL32	Interleukin 32
2264.	CSN1S1	Casein Alpha S1
2265.	IGK	Immunoglobulin Kappa Locus
2266.	PLA2G7	Phospholipase A2 Group VII
2267.	CDC25A	Cell Division Cycle 25A
2268.	SLC5A2	Solute Carrier Family 5 Member 2
2269.	GNA11	G Protein Subunit Alpha 11
2270.	NOG	Noggin
2271.	PGD	Phosphogluconate Dehydrogenase
2272.	TRAF6	TNF Receptor Associated Factor 6
2273.	DPP6	Dipeptidyl Peptidase Like 6
2274.	APTX	Aprataxin
2275.	BAD	BCL2 Associated Agonist Of Cell Death
2276.	HLA-DRA	Major Histocompatibility Complex, Class II, DR Alpha
2277.	TP53BP1	Tumor Protein P53 Binding Protein 1
2278.	AGTR2	Angiotensin II Receptor Type 2
2279.	AICDA	Activation Induced Cytidine Deaminase
2280.	CHD8	Chromodomain Helicase DNA Binding Protein 8
2281.	NR1I2	Nuclear Receptor Subfamily 1 Group I Member 2
2282.	ADAMTSL1	ADAMTS Like 1
2283.	GRP	Gastrin Releasing Peptide
2284.	SLAMF7	SLAM Family Member 7
2285.	SLC34A3	Solute Carrier Family 34 Member 3
2286.	ANOS1	Anosmin 1
2287.	FGF20	Fibroblast Growth Factor 20
2288.	MRC1	Mannose Receptor C-Type 1
2289.	MUC4	Mucin 4, Cell Surface Associated

2290.	PPY	Pancreatic Polypeptide
2291.	NUDT10	Nudix Hydrolase 10
2292.	MEF2C	Myocyte Enhancer Factor 2C
2293.	CXCR2	C-X-C Motif Chemokine Receptor 2
2294.	TGFB3	Transforming Growth Factor Beta 3
2295.	CARD11	Caspase Recruitment Domain Family Member 11
2296.	FUT2	Fucosyltransferase 2 (H Blood Group)
2297.	GAL	Galanin And GMAP Prepropeptide
2298.	GHRHR	Growth Hormone Releasing Hormone Receptor
2299.	MSLN	Mesothelin
2300.	NLRP1	NLR Family Pyrin Domain Containing 1
2301.	REG1A	Regenerating Family Member 1 Alpha
2302.	SCTR	Secretin Receptor
2303.	AP1S1	Adaptor Related Protein Complex 1 Subunit Sigma 1
2304.	MYOG	Myogenin
2305.	CCL22	C-C Motif Chemokine Ligand 22
2306.	CD1B	CD1b Molecule
2307.	CD1E	CD1e Molecule
2308.	CLEC11A	C-Type Lectin Domain Containing 11A
2309.	SMO	Smoothened, Frizzled Class Receptor
2310.	WEE1	WEE1 G2 Checkpoint Kinase
2311.	KLK6	Kallikrein Related Peptidase 6
2312.	NCOR1	Nuclear Receptor Corepressor 1
2313.	TAL1	TAL BHLH Transcription Factor 1, Erythroid Differentiation Factor
2314.	CD52	CD52 Molecule
2315.	FUT4	Fucosyltransferase 4
2316.	GPX8	Glutathione Peroxidase 8 (Putative)
2317.	STH	Saitohin
2318.	PSMD3	Proteasome 26S Subunit, Non-ATPase 3
2319.	TRIO	Trio Rho Guanine Nucleotide Exchange Factor
2320.	NAXE	NAD(P)HX Epimerase
2321.	RIN2	Ras And Rab Interactor 2
2322.	HEPHL1	Hephaestin Like 1
2323.	CARMIL2	Capping Protein Regulator And Myosin 1 Linker 2
2324.	POLR3GL	RNA Polymerase III Subunit GL
2325.	GLUD1	Glutamate Dehydrogenase 1
2326.	PSMB8	Proteasome 20S Subunit Beta 8
2327.	FGA	Fibrinogen Alpha Chain
2328.	ANXA1	Annexin A1
2329.	CNTNAP2	Contactin Associated Protein 2
2330.	PDE8B	Phosphodiesterase 8B

2331.	SKP2	S-Phase Kinase Associated Protein 2
2332.	BSG	Basigin (Ok Blood Group)
2333.	TRPM6	Transient Receptor Potential Cation Channel Subfamily M Member 6
2334.	SNAI2	Snail Family Transcriptional Repressor 2
2335.	PREP	Prolyl Endopeptidase
2336.	PMAIP1	Phorbol-12-Myristate-13-Acetate-Induced Protein 1
2337.	CEP70	Centrosomal Protein 70
2338.	DLD	Dihydrolipoamide Dehydrogenase
2339.	LRP12	LDL Receptor Related Protein 12
2340.	EMILIN1	Elastin Microfibril Interfacer 1
2341.	BUB1	BUB1 Mitotic Checkpoint Serine/Threonine Kinase
2342.	SIN3B	SIN3 Transcription Regulator Family Member B
2343.	XK	X-Linked Kx Blood Group Antigen, Kell And VPS13A Binding Protein
2344.	PDE4A	Phosphodiesterase 4A
2345.	ASAH1	N-Acylsphingosine Amidohydrolase 1
2346.	IDS	Iduronate 2-Sulfatase
2347.	ZIC3	Zic Family Member 3
2348.	UGCG	UDP-Glucose Ceramide Glucosyltransferase
2349.	PLEK	Pleckstrin
2350.	NAV1	Neuron Navigator 1
2351.	RRM2	Ribonucleotide Reductase Regulatory Subunit M2
2352.	F13B	Coagulation Factor XIII B Chain
2353.	EXOSC10	Exosome Component 10
2354.	PDPK1	3-Phosphoinositide Dependent Protein Kinase 1
2355.	NR1H2	Nuclear Receptor Subfamily 1 Group H Member 2
2356.	PTMA	Prothymosin Alpha
2357.	SDHAF1	Succinate Dehydrogenase Complex Assembly Factor 1
2358.	SIRT3	Sirtuin 3
2359.	TMPRSS2	Transmembrane Serine Protease 2
2360.	UMPS	Uridine Monophosphate Synthetase
2361.	AHR	Aryl Hydrocarbon Receptor
2362.	EHMT2	Euchromatic Histone Lysine Methyltransferase 2
2363.	OTC	Ornithine Transcarbamylase
2364.	PROM1	Prominin 1
2365.	KAT6A	Lysine Acetyltransferase 6A
2366.	TWIST1	Twist Family BHLH Transcription Factor 1
2367.	CCKBR	Cholecystokinin B Receptor
2368.	RARS1	Arginyl-TRNA Synthetase 1
2369.	ASCL1	Achaete-Scute Family BHLH Transcription Factor 1
2370.	CCNA1	Cyclin A1
2371.	GPX7	Glutathione Peroxidase 7

2372.	SLC46A1	Solute Carrier Family 46 Member 1
2373.	GPX2	Glutathione Peroxidase 2
2374.	CHCHD2	Coiled-Coil-Helix-Coiled-Coil-Helix Domain Containing 2
2375.	SLC25A38	Solute Carrier Family 25 Member 38
2376.	TET1	Tet Methylcytosine Dioxygenase 1
2377.	H2AC20	H2A Clustered Histone 20
2378.	GPX6	Glutathione Peroxidase 6
2379.	H2AC18	H2A Clustered Histone 18
2380.	ARHGAP26	Rho GTPase Activating Protein 26
2381.	EPHB2	EPH Receptor B2
2382.	CACNA1G	Calcium Voltage-Gated Channel Subunit Alpha1 G
2383.	TYR	Tyrosinase
2384.	ALDH7A1	Aldehyde Dehydrogenase 7 Family Member A1
2385.	AXIN1	Axin 1
2386.	CSK	C-Terminal Src Kinase
2387.	FES	FES Proto-Oncogene, Tyrosine Kinase
2388.	GLP1R	Glucagon Like Peptide 1 Receptor
2389.	NEDD4L	NEDD4 Like E3 Ubiquitin Protein Ligase
2390.	SUMO1	Small Ubiquitin Like Modifier 1
2391.	EZR	Ezrin
2392.	SERPINF2	Serpin Family F Member 2
2393.	CD163	CD163 Molecule
2394.	CSF2RA	Colony Stimulating Factor 2 Receptor Subunit Alpha
2395.	F2RL1	F2R Like Trypsin Receptor 1
2396.	RPS6	Ribosomal Protein S6
2397.	S100A4	S100 Calcium Binding Protein A4
2398.	AGPAT2	1-Acylglycerol-3-Phosphate O-Acyltransferase 2
2399.	POLI	DNA Polymerase Iota
2400.	APCS	Amyloid P Component, Serum
2401.	UROS	Uroporphyrinogen III Synthase
2402.	G6PC3	Glucose-6-Phosphatase Catalytic Subunit 3
2403.	SLC39A4	Solute Carrier Family 39 Member 4
2404.	NR0B2	Nuclear Receptor Subfamily 0 Group B Member 2
2405.	SOX11	SRY-Box Transcription Factor 11
2406.	ELAVL4	ELAV Like RNA Binding Protein 4
2407.	GK2	Glycerol Kinase 2
2408.	MPIG6B	Megakaryocyte And Platelet Inhibitory Receptor G6b
2409.	UBA7	Ubiquitin Like Modifier Activating Enzyme 7
2410.	BMP10	Bone Morphogenetic Protein 10
2411.	HLA-DQA2	Major Histocompatibility Complex, Class II, DQ Alpha 2
2412.	SULT1A3	Sulfotransferase Family 1A Member 3

2413.	DEFB4A	Defensin Beta 4A
2414.	FYN	FYN Proto-Oncogene, Src Family Tyrosine Kinase
2415.	IDUA	Alpha-L-Iduronidase
2416.	ADARB1	Adenosine Deaminase RNA Specific B1
2417.	LARS2	Leucyl-TRNA Synthetase 2, Mitochondrial
2418.	CMA1	Chymase 1
2419.	TNFRSF18	TNF Receptor Superfamily Member 18
2420.	NBEAL2	Neurobeachin Like 2
2421.	SIGLEC1	Sialic Acid Binding Ig Like Lectin 1
2422.	CLCN6	Chloride Voltage-Gated Channel 6
2423.	KBTBD13	Kelch Repeat And BTB Domain Containing 13
2424.	EFEMP1	EGF Containing Fibulin Extracellular Matrix Protein 1
2425.	BTD	Biotinidase
2426.	HADH	Hydroxyacyl-CoA Dehydrogenase
2427.	ATR	ATR Serine/Threonine Kinase
2428.	NOTCH2	Notch Receptor 2
2429.	MATK	Megakaryocyte-Associated Tyrosine Kinase
2430.	GZMA	Granzyme A
2431.	STK39	Serine/Threonine Kinase 39
2432.	UMOD	Uromodulin
2433.	CD84	CD84 Molecule
2434.	CLDN16	Claudin 16
2435.	WNK3	WNK Lysine Deficient Protein Kinase 3
2436.	PDLIM7	PDZ And LIM Domain 7
2437.	HLA-DMA	Major Histocompatibility Complex, Class II, DM Alpha
2438.	HLA-DMB	Major Histocompatibility Complex, Class II, DM Beta
2439.	CLEC16A	C-Type Lectin Domain Containing 16A
2440.	FCRL3	Fc Receptor Like 3
2441.	KCNK18	Potassium Two Pore Domain Channel Subfamily K Member 18
2442.	EGLN1	Egl-9 Family Hypoxia Inducible Factor 1
2443.	ARG1	Arginase 1
2444.	RRM1	Ribonucleotide Reductase Catalytic Subunit M1
2445.	GHSR	Growth Hormone Secretagogue Receptor
2446.	GCDH	Glutaryl-CoA Dehydrogenase
2447.	GPNMB	Glycoprotein Nmb
2448.	SOX10	SRY-Box Transcription Factor 10
2449.	ABCC4	ATP Binding Cassette Subfamily C Member 4 (PEL Blood Group)
2450.	GALC	Galactosylceramidase
2451.	SSTR3	Somatostatin Receptor 3
2452.	SEPSECS	Sep (O-Phosphoserine) TRNA:Sec (Selenocysteine) TRNA Synthase
2453.	ACAD8	Acyl-CoA Dehydrogenase Family Member 8

2454.	EEA1	Early Endosome Antigen 1
2455.	SOCS2	Suppressor Of Cytokine Signaling 2
2456.	COQ9	Coenzyme Q9
2457.	TFF3	Trefoil Factor 3
2458.	LSM2	LSM2 Homolog, U6 Small Nuclear RNA And MRNA Degradation Associated
2459.	COQ8B	Coenzyme Q8B
2460.	MEPE	Matrix Extracellular Phosphoglycoprotein
2461.	SLC51A	Solute Carrier Family 51 Member A
2462.	CD99L2	CD99 Molecule Like 2
2463.	FGF4	Fibroblast Growth Factor 4
2464.	SSTR1	Somatostatin Receptor 1
2465.	RPS6KA1	Ribosomal Protein S6 Kinase A1
2466.	ATP2A1	ATPase Sarcoplasmic/Endoplasmic Reticulum Ca2+ Transporting 1
2467.	SPR	Sepiapterin Reductase
2468.	FASN	Fatty Acid Synthase
2469.	HDAC3	Histone Deacetylase 3
2470.	STAT2	Signal Transducer And Activator Of Transcription 2
2471.	CAPN2	Calpain 2
2472.	EIF4G1	Eukaryotic Translation Initiation Factor 4 Gamma 1
2473.	ACO2	Aconitase 2
2474.	CA9	Carbonic Anhydrase 9
2475.	DKC1	Dyskerin Pseudouridine Synthase 1
2476.	FAH	Fumarylacetoacetate Hydrolase
2477.	REL	REL Proto-Oncogene, NF-KB Subunit
2478.	RUNX2	RUNX Family Transcription Factor 2
2479.	XRCC5	X-Ray Repair Cross Complementing 5
2480.	CD276	CD276 Molecule
2481.	DOT1L	DOT1 Like Histone Lysine Methyltransferase
2482.	CEBPE	CCAAT Enhancer Binding Protein Epsilon
2483.	HEPH	Hephaestin
2484.	TINF2	TERF1 Interacting Nuclear Factor 2
2485.	GLRX5	Glutaredoxin 5
2486.	HOXA10	Homeobox A10
2487.	UBE2J2	Ubiquitin Conjugating Enzyme E2 J2
2488.	GPX5	Glutathione Peroxidase 5
2489.	MLLT1	MLLT1 Super Elongation Complex Subunit
2490.	AFF1	ALF Transcription Elongation Factor 1
2491.	ASB2	Ankyrin Repeat And SOCS Box Containing 2
2492.	G6PC2	Glucose-6-Phosphatase Catalytic Subunit 2
2493.	ZFPM1	Zinc Finger Protein, FOG Family Member 1
2494.	KLRC2	Killer Cell Lectin Like Receptor C2

2495.	MAGEA1	MAGE Family Member A1
2496.	HBQ1	Hemoglobin Subunit Theta 1
2497.	PRAM1	PML-RARA Regulated Adaptor Molecule 1
2498.	TMEM175	Transmembrane Protein 175
2499.	ATG13	Autophagy Related 13
2500.	CISH	Cytokine Inducible SH2 Containing Protein
2501.	PNPLA2	Patatin Like Phospholipase Domain Containing 2
2502.	MTMR14	Myotubularin Related Protein 14
2503.	FXR1	FMR1 Autosomal Homolog 1
2504.	PABPN1	Poly(A) Binding Protein Nuclear 1
2505.	BVES	Blood Vessel Epicardial Substance
2506.	ASCC1	Activating Signal Cointegrator 1 Complex Subunit 1
2507.	SLC25A32	Solute Carrier Family 25 Member 32
2508.	COL12A1	Collagen Type XII Alpha 1 Chain
2509.	POLRMT	RNA Polymerase Mitochondrial
2510.	REEP1	Receptor Accessory Protein 1
2511.	COX11	Cytochrome C Oxidase Copper Chaperone COX11
2512.	HPDL	4-Hydroxyphenylpyruvate Dioxygenase Like
2513.	AMD1	Adenosylmethionine Decarboxylase 1
2514.	COIL	Coilin
2515.	SMARCA2	SWI/SNF Related, Matrix Associated, Actin Dependent Regulator Of Chromatin, Subfamily A, Member 2
2516.	BACE1	Beta-Secretase 1
2517.	MAP2K4	Mitogen-Activated Protein Kinase Kinase 4
2518.	NCOA3	Nuclear Receptor Coactivator 3
2519.	CACNA2D1	Calcium Voltage-Gated Channel Auxiliary Subunit Alpha2delta 1
2520.	HEXA	Hexosaminidase Subunit Alpha
2521.	MAPK10	Mitogen-Activated Protein Kinase 10
2522.	ACVR2A	Activin A Receptor Type 2A
2523.	GALNT3	Polypeptide N-Acetylgalactosaminyltransferase 3
2524.	NDRG1	N-Myc Downstream Regulated 1
2525.	PPIA	Peptidylprolyl Isomerase A
2526.	PRSS8	Serine Protease 8
2527.	PYGL	Glycogen Phosphorylase L
2528.	TRAF2	TNF Receptor Associated Factor 2
2529.	CNTN1	Contactin 1
2530.	EGR2	Early Growth Response 2
2531.	HELLS	Helicase, Lymphoid Specific
2532.	OCA2	OCA2 Melanosomal Transmembrane Protein
2533.	SNRPN	Small Nuclear Ribonucleoprotein Polypeptide N
2534.	EXO1	Exonuclease 1
2535.	GADD45A	Growth Arrest And DNA Damage Inducible Alpha

2536.	IL1RAPL1	Interleukin 1 Receptor Accessory Protein Like 1
2537.	LCP2	Lymphocyte Cytosolic Protein 2
2538.	MPI	Mannose Phosphate Isomerase
2539.	CNTNAP1	Contactin Associated Protein 1
2540.	COMMD1	Copper Metabolism Domain Containing 1
2541.	FLG	Filaggrin
2542.	RNMT	RNA Guanine-7 Methyltransferase
2543.	SPINK1	Serine Peptidase Inhibitor Kazal Type 1
2544.	TPX2	TPX2 Microtubule Nucleation Factor
2545.	CRELD1	Cysteine Rich With EGF Like Domains 1
2546.	PDSS2	Decaprenyl Diphosphate Synthase Subunit 2
2547.	SCNN1D	Sodium Channel Epithelial 1 Subunit Delta
2548.	TERF1	Telomeric Repeat Binding Factor 1
2549.	BCL3	BCL3 Transcription Coactivator
2550.	CEACAM6	CEA Cell Adhesion Molecule 6
2551.	KISS1	KiSS-1 Metastasis Suppressor
2552.	SLC39A13	Solute Carrier Family 39 Member 13
2553.	GPBAR1	G Protein-Coupled Bile Acid Receptor 1
2554.	IL15RA	Interleukin 15 Receptor Subunit Alpha
2555.	LMBRD1	LMBR1 Domain Containing 1
2556.	MPZL1	Myelin Protein Zero Like 1
2557.	NUP210	Nucleoporin 210
2558.	S100A1	S100 Calcium Binding Protein A1
2559.	TBCB	Tubulin Folding Cofactor B
2560.	CIDEC	Cell Death Inducing DFFA Like Effector C
2561.	DMTN	Dematin Actin Binding Protein
2562.	FGGY	FGGY Carbohydrate Kinase Domain Containing
2563.	SUMO4	Small Ubiquitin Like Modifier 4
2564.	SP140	SP140 Nuclear Body Protein
2565.	PRDM10	PR/SET Domain 10
2566.	HSPA1A	Heat Shock Protein Family A (Hsp70) Member 1A
2567.	PDHX	Pyruvate Dehydrogenase Complex Component X
2568.	WNT7B	Wnt Family Member 7B
2569.	PCSK9	Proprotein Convertase Subtilisin/Kexin Type 9
2570.	KCNMA1	Potassium Calcium-Activated Channel Subfamily M Alpha 1
2571.	CETP	Cholesteryl Ester Transfer Protein
2572.	VIPR1	Vasoactive Intestinal Peptide Receptor 1
2573.	TLR6	Toll Like Receptor 6
2574.	LGR5	Leucine Rich Repeat Containing G Protein-Coupled Receptor 5
2575.	NME4	NME/NM23 Nucleoside Diphosphate Kinase 4
2576.	PLA2G10	Phospholipase A2 Group X

2577.	CLDN11	Claudin 11
2578.	IFITM3	Interferon Induced Transmembrane Protein 3
2579.	RHOBTB2	Rho Related BTB Domain Containing 2
2580.	ZBTB18	Zinc Finger And BTB Domain Containing 18
2581.	CRLS1	Cardiolipin Synthase 1
2582.	IMMT	Inner Membrane Mitochondrial Protein
2583.	ISG20	Interferon Stimulated Exonuclease Gene 20
2584.	SLC35A2	Solute Carrier Family 35 Member A2
2585.	CD72	CD72 Molecule
2586.	HAS1	Hyaluronan Synthase 1
2587.	MCU	Mitochondrial Calcium Uniporter
2588.	TSLP	Thymic Stromal Lymphopoietin
2589.	PRELID1	PRELI Domain Containing 1
2590.	PTPMT1	Protein Tyrosine Phosphatase Mitochondrial 1
2591.	CHCHD3	Coiled-Coil-Helix-Coiled-Coil-Helix Domain Containing 3
2592.	PLSCR3	Phospholipid Scramblase 3
2593.	LCLAT1	Lysocardiolipin Acyltransferase 1
2594.	MICU2	Mitochondrial Calcium Uptake 2
2595.	SAMD9	Sterile Alpha Motif Domain Containing 9
2596.	SYNJ2BP	Synaptojanin 2 Binding Protein
2597.	APOOL	Apolipoprotein O Like
2598.	PRKAA1	Protein Kinase AMP-Activated Catalytic Subunit Alpha 1
2599.	COX5A	Cytochrome C Oxidase Subunit 5A
2600.	ACADSB	Acyl-CoA Dehydrogenase Short/Branched Chain
2601.	SAA4	Serum Amyloid A4, Constitutive
2602.	LIPA	Lipase A, Lysosomal Acid Type
2603.	SMAD9	SMAD Family Member 9
2604.	SGSH	N-Sulfoglucosamine Sulfohydrolase
2605.	NPC2	NPC Intracellular Cholesterol Transporter 2
2606.	KCNJ12	Potassium Inwardly Rectifying Channel Subfamily J Member 12
2607.	GBA2	Glucosylceramidase Beta 2
2608.	CCL17	C-C Motif Chemokine Ligand 17
2609.	POMGNT2	Protein O-Linked Mannose N-Acetylglucosaminyltransferase 2 (Beta 1,4-)
2610.	RXYLT1	Ribitol Xylosyltransferase 1
2611.	AARS2	Alanyl-TRNA Synthetase 2, Mitochondrial
2612.	MEGF10	Multiple EGF Like Domains 10
2613.	VPS13A	Vacuolar Protein Sorting 13 Homolog A
2614.	EARS2	Glutamyl-TRNA Synthetase 2, Mitochondrial
2615.	SYNE2	Spectrin Repeat Containing Nuclear Envelope Protein 2
2616.	OBSCN	Obscurin, Cytoskeletal Calmodulin And Titin-Interacting RhoGEF
2617.	TRMU	TRNA Mitochondrial 2-Thiouridylase

2618.	ITPKC	Inositol-Trisphosphate 3-Kinase C
2619.	PCSK1	Proprotein Convertase Subtilisin/Kexin Type 1
2620.	PBX1	PBX Homeobox 1
2621.	FSHR	Follicle Stimulating Hormone Receptor
2622.	KEAP1	Kelch Like ECH Associated Protein 1
2623.	SIRT2	Sirtuin 2
2624.	FGR	FGR Proto-Oncogene, Src Family Tyrosine Kinase
2625.	HSP90B1	Heat Shock Protein 90 Beta Family Member 1
2626.	KCNA2	Potassium Voltage-Gated Channel Subfamily A Member 2
2627.	OGDH	Oxoglutarate Dehydrogenase
2628.	SMARCB1	SWI/SNF Related, Matrix Associated, Actin Dependent Regulator Of Chromatin, Subfamily B, Member 1
2629.	XRCC6	X-Ray Repair Cross Complementing 6
2630.	CPOX	Coproporphyrinogen Oxidase
2631.	CST3	Cystatin C
2632.	NHP2	NHP2 Ribonucleoprotein
2633.	RAG1	Recombination Activating 1
2634.	SHC1	SHC Adaptor Protein 1
2635.	SYN1	Synapsin I
2636.	NR5A2	Nuclear Receptor Subfamily 5 Group A Member 2
2637.	SFRP1	Secreted Frizzled Related Protein 1
2638.	NOP10	NOP10 Ribonucleoprotein
2639.	BBC3	BCL2 Binding Component 3
2640.	SFRP2	Secreted Frizzled Related Protein 2
2641.	AIF1	Allograft Inflammatory Factor 1
2642.	FLVCR1	FLVCR Choline And Heme Transporter 1
2643.	NRTN	Neurturin
2644.	TNFRSF10C	TNF Receptor Superfamily Member 10c
2645.	TOMM20	Translocase Of Outer Mitochondrial Membrane 20
2646.	CEACAM8	CEA Cell Adhesion Molecule 8
2647.	CPQ	Carboxypeptidase Q
2648.	MICA	MHC Class I Polypeptide-Related Sequence A
2649.	IGLON5	IgLON Family Member 5
2650.	GSTT1	Glutathione S-Transferase Theta 1
2651.	TM6SF2	Transmembrane 6 Superfamily Member 2
2652.	TRB	T Cell Receptor Beta Locus
2653.	CHST11	Carbohydrate Sulfotransferase 11
2654.	CD320	CD320 Molecule
2655.	STAG2	STAG2 Cohesin Complex Component
2656.	LAG3	Lymphocyte Activating 3
2657.	PNOC	Prepronociceptin
2658.	SYPL2	Synaptophysin Like 2

2659.	FPGS	Folylpolyglutamate Synthase
2660.	TNC	Tenascin C
2661.	CLOCK	Clock Circadian Regulator
2662.	LRP5	LDL Receptor Related Protein 5
2663.	CHRNA3	Cholinergic Receptor Nicotinic Alpha 3 Subunit
2664.	H3-3A	H3.3 Histone A
2665.	HPS5	HPS5 Biogenesis Of Lysosomal Organelles Complex 2 Subunit 2
2666.	FLAD1	Flavin Adenine Dinucleotide Synthetase 1
2667.	HPS6	HPS6 Biogenesis Of Lysosomal Organelles Complex 2 Subunit 3
2668.	ASTN2	Astrotactin 2
2669.	ANO1	Anoctamin 1
2670.	KARS1	Lysyl-TRNA Synthetase 1
2671.	FGF5	Fibroblast Growth Factor 5
2672.	GREM1	Gremlin 1, DAN Family BMP Antagonist
2673.	CCL26	C-C Motif Chemokine Ligand 26
2674.	SYNC	Syncoilin, Intermediate Filament Protein
2675.	CHRM3	Cholinergic Receptor Muscarinic 3
2676.	CASP2	Caspase 2
2677.	NR1I3	Nuclear Receptor Subfamily 1 Group I Member 3
2678.	LY6E	Lymphocyte Antigen 6 Family Member E
2679.	CYP2E1	Cytochrome P450 Family 2 Subfamily E Member 1
2680.	BCL11A	BCL11 Transcription Factor A
2681.	AR	Androgen Receptor
2682.	GNAQ	G Protein Subunit Alpha Q
2683.	MTAP	Methylthioadenosine Phosphorylase
2684.	GP1BA	Glycoprotein Ib Platelet Subunit Alpha
2685.	ITGAV	Integrin Subunit Alpha V
2686.	ATP2B3	ATPase Plasma Membrane Ca2+ Transporting 3
2687.	DROSHA	Drosha Ribonuclease III
2688.	INPP5D	Inositol Polyphosphate-5-Phosphatase D
2689.	BACH2	BTB Domain And CNC Homolog 2
2690.	CCL20	C-C Motif Chemokine Ligand 20
2691.	HAVCR1	Hepatitis A Virus Cellular Receptor 1
2692.	DDX39B	DExD-Box Helicase 39B
2693.	PGK2	Phosphoglycerate Kinase 2
2694.	CXCL16	C-X-C Motif Chemokine Ligand 16
2695.	STIM2	Stromal Interaction Molecule 2
2696.	IBA57	Iron-Sulfur Cluster Assembly Factor IBA57
2697.	PRRC2A	Proline Rich Coiled-Coil 2A
2698.	ANKRD46	Ankyrin Repeat Domain 46
2699.	DMPK	DM1 Protein Kinase

2700.	MEF2A	Myocyte Enhancer Factor 2A
2701.	AKT3	AKT Serine/Threonine Kinase 3
2702.	ACVR1	Activin A Receptor Type 1
2703.	AXIN2	Axin 2
2704.	CASP7	Caspase 7
2705.	PRKCZ	Protein Kinase C Zeta
2706.	PRLR	Prolactin Receptor
2707.	FGF1	Fibroblast Growth Factor 1
2708.	L1CAM	L1 Cell Adhesion Molecule
2709.	MYH9	Myosin Heavy Chain 9
2710.	UBE2I	Ubiquitin Conjugating Enzyme E2 I
2711.	BMP7	Bone Morphogenetic Protein 7
2712.	CRKL	CRK Like Proto-Oncogene, Adaptor Protein
2713.	IL5RA	Interleukin 5 Receptor Subunit Alpha
2714.	LAMC2	Laminin Subunit Gamma 2
2715.	MC4R	Melanocortin 4 Receptor
2716.	PAX3	Paired Box 3
2717.	VCAN	Versican
2718.	YWHAB	Tyrosine 3-Monooxygenase/Tryptophan 5-Monooxygenase Activation Protein Beta
2719.	CARD9	Caspase Recruitment Domain Family Member 9
2720.	CGA	Glycoprotein Hormones, Alpha Polypeptide
2721.	CLEC7A	C-Type Lectin Domain Containing 7A
2722.	LOXL2	Lysyl Oxidase Like 2
2723.	QDPR	Quinoid Dihydropteridine Reductase
2724.	TACSTD2	Tumor Associated Calcium Signal Transducer 2
2725.	BTRC	Beta-Transducin Repeat Containing E3 Ubiquitin Protein Ligase
2726.	OCRL	OCRL Inositol Polyphosphate-5-Phosphatase
2727.	COL11A2	Collagen Type XI Alpha 2 Chain
2728.	DHX9	DExH-Box Helicase 9
2729.	FGF19	Fibroblast Growth Factor 19
2730.	GRPR	Gastrin Releasing Peptide Receptor
2731.	TREH	Trehalase
2732.	VEGFB	Vascular Endothelial Growth Factor B
2733.	ATXN10	Ataxin 10
2734.	ATXN7	Ataxin 7
2735.	CARD14	Caspase Recruitment Domain Family Member 14
2736.	COQ6	Coenzyme Q6, Monooxygenase
2737.	HCLS1	Hematopoietic Cell-Specific Lyn Substrate 1
2738.	IGFBP5	Insulin Like Growth Factor Binding Protein 5
2739.	MSH5	MutS Homolog 5
2740.	RHOC	Ras Homolog Family Member C

2741.	RNF168	Ring Finger Protein 168
2742.	S100A6	S100 Calcium Binding Protein A6
2743.	ABCD4	ATP Binding Cassette Subfamily D Member 4
2744.	ATL1	Atlastin GTPase 1
2745.	ATOX1	Antioxidant 1 Copper Chaperone
2746.	DNA2	DNA Replication Helicase/Nuclease 2
2747.	NMT1	N-Myristoyltransferase 1
2748.	PDPN	Podoplanin
2749.	SLC4A2	Solute Carrier Family 4 Member 2
2750.	BNIP3	BCL2 Interacting Protein 3
2751.	CD83	CD83 Molecule
2752.	B3GALT6	Beta-1,3-Galactosyltransferase 6
2753.	CAMP	Cathelicidin Antimicrobial Peptide
2754.	DNAL1	Dynein Axonemal Light Chain 1
2755.	SNRPA	Small Nuclear Ribonucleoprotein Polypeptide A
2756.	SRM	Spermidine Synthase
2757.	CALCOCO2	Calcium Binding And Coiled-Coil Domain 2
2758.	CD200	CD200 Molecule
2759.	COQ4	Coenzyme Q4
2760.	CYB561	Cytochrome B561
2761.	IBTK	Inhibitor Of Bruton Tyrosine Kinase
2762.	TMOD2	Tropomodulin 2
2763.	CLEC6A	C-Type Lectin Domain Containing 6A
2764.	CYP8B1	Cytochrome P450 Family 8 Subfamily B Member 1
2765.	DEFA1	Defensin Alpha 1
2766.	OXA1L	OXA1L Mitochondrial Inner Membrane Protein
2767.	IFNL3	Interferon Lambda 3
2768.	ESX1	ESX Homeobox 1
2769.	SLC51B	SLC51 Subunit Beta
2770.	WDFY4	WDFY Family Member 4
2771.	LMLN	Leishmanolysin Like Peptidase
2772.	LRRC18	Leucine Rich Repeat Containing 18
2773.	LIAT1	Ligand Of ATE1
2774.	PYDC1	Pyrin Domain Containing 1
2775.	C1orf141	Chromosome 1 Open Reading Frame 141
2776.	PHKG1	Phosphorylase Kinase Catalytic Subunit Gamma 1
2777.	RELA	RELA Proto-Oncogene, NF-KB Subunit
2778.	SLC12A2	Solute Carrier Family 12 Member 2
2779.	AKR1A1	Aldo-Keto Reductase Family 1 Member A1
2780.	H2BC21	H2B Clustered Histone 21
2781.	PHF6	PHD Finger Protein 6

2782.	LINGO2	Leucine Rich Repeat And Ig Domain Containing 2
2783.	BLM	BLM RecQ Like Helicase
2784.	GRIA1	Glutamate Ionotropic Receptor AMPA Type Subunit 1
2785.	GYS1	Glycogen Synthase 1
2786.	VKORC1	Vitamin K Epoxide Reductase Complex Subunit 1
2787.	MRE11	MRE11 Homolog, Double Strand Break Repair Nuclease
2788.	CTSL	Cathepsin L
2789.	GPX4	Glutathione Peroxidase 4
2790.	RHO	Rhodopsin
2791.	RPA1	Replication Protein A1
2792.	CFB	Complement Factor B
2793.	HK2	Hexokinase 2
2794.	HUWE1	HECT, UBA And WWE Domain Containing E3 Ubiquitin Protein Ligase 1
2795.	NEU1	Neuraminidase 1
2796.	THRA	Thyroid Hormone Receptor Alpha
2797.	YWHAQ	Tyrosine 3-Monooxygenase/Tryptophan 5-Monooxygenase Activation Protein Theta
2798.	AMACR	Alpha-Methylacyl-CoA Racemase
2799.	ARRB2	Arrestin Beta 2
2800.	C2	Complement C2
2801.	CDK5R1	Cyclin Dependent Kinase 5 Regulatory Subunit 1
2802.	FADD	Fas Associated Via Death Domain
2803.	MSX2	Msh Homeobox 2
2804.	C5	Complement C5
2805.	CADM1	Cell Adhesion Molecule 1
2806.	CASQ2	Calsequestrin 2
2807.	ILK	Integrin Linked Kinase
2808.	PRMT5	Protein Arginine Methyltransferase 5
2809.	PSMD4	Proteasome 26S Subunit Ubiquitin Receptor, Non-ATPase 4
2810.	PTPRN	Protein Tyrosine Phosphatase Receptor Type N
2811.	PBRM1	Polybromo 1
2812.	PSMA2	Proteasome 20S Subunit Alpha 2
2813.	SLC39A14	Solute Carrier Family 39 Member 14
2814.	TOLLIP	Toll Interacting Protein
2815.	UBQLN1	Ubiquilin 1
2816.	FBL	Fibrillarin
2817.	ITIH4	Inter-Alpha-Trypsin Inhibitor Heavy Chain 4
2818.	NEO1	Neogenin 1
2819.	PRKAR2B	Protein Kinase CAMP-Dependent Type II Regulatory Subunit Beta
2820.	PROCR	Protein C Receptor
2821.	PSMC4	Proteasome 26S Subunit, ATPase 4
2822.	SRD5A2	Steroid 5 Alpha-Reductase 2

2823.	TPSAB1	Tryptase Alpha/Beta 1
2824.	XRCC3	X-Ray Repair Cross Complementing 3
2825.	CBX5	Chromobox 5
2826.	FABP2	Fatty Acid Binding Protein 2
2827.	S100A2	S100 Calcium Binding Protein A2
2828.	WNT9A	Wnt Family Member 9A
2829.	RNF19A	Ring Finger Protein 19A, RBR E3 Ubiquitin Protein Ligase
2830.	RPL7	Ribosomal Protein L7
2831.	BRD3	Bromodomain Containing 3
2832.	MUC6	Mucin 6, Oligomeric Mucus/Gel-Forming
2833.	SLC39A1	Solute Carrier Family 39 Member 1
2834.	KCNK5	Potassium Two Pore Domain Channel Subfamily K Member 5
2835.	MNDA	Myeloid Cell Nuclear Differentiation Antigen
2836.	RAB39B	RAB39B, Member RAS Oncogene Family
2837.	CCNL2	Cyclin L2
2838.	GYPB	Glycophorin B (MNS Blood Group)
2839.	SPA17	Sperm Autoantigenic Protein 17
2840.	TXNL1	Thioredoxin Like 1
2841.	CDIN1	CDAN1 Interacting Nuclease 1
2842.	FBXL5	F-Box And Leucine Rich Repeat Protein 5
2843.	RAB29	RAB29, Member RAS Oncogene Family
2844.	H4C6	H4 Clustered Histone 6
2845.	MYEOV	Myeloma Overexpressed
2846.	H4C7	H4 Clustered Histone 7
2847.	ALDH2	Aldehyde Dehydrogenase 2 Family Member
2848.	PSMB9	Proteasome 20S Subunit Beta 9
2849.	MYF6	Myogenic Factor 6
2850.	ADK	Adenosine Kinase
2851.	SLC29A3	Solute Carrier Family 29 Member 3
2852.	EXOSC2	Exosome Component 2
2853.	SLC29A4	Solute Carrier Family 29 Member 4
2854.	SLC28A1	Solute Carrier Family 28 Member 1
2855.	SLC28A2	Solute Carrier Family 28 Member 2
2856.	NT5C1B	5'-Nucleotidase, Cytosolic IB
2857.	GRIA3	Glutamate Ionotropic Receptor AMPA Type Subunit 3
2858.	LYZ	Lysozyme
2859.	SERPINF1	Serpin Family F Member 1
2860.	ADRA2C	Adrenoceptor Alpha 2C
2861.	KCNJ8	Potassium Inwardly Rectifying Channel Subfamily J Member 8
2862.	POGLUT1	Protein O-Glucosyltransferase 1
2863.	LIMS3	LIM Zinc Finger Domain Containing 3

2864.	HTR1D	5-Hydroxytryptamine Receptor 1D
2865.	TFG	Trafficking From ER To Golgi Regulator
2866.	GLI3	GLI Family Zinc Finger 3
2867.	CTBP1	C-Terminal Binding Protein 1
2868.	LPIN1	Lipin 1
2869.	SLC25A12	Solute Carrier Family 25 Member 12
2870.	GFER	Growth Factor, Augmenter Of Liver Regeneration
2871.	MYL1	Myosin Light Chain 1
2872.	YME1L1	YME1 Like 1 ATPase
2873.	SIL1	SIL1 Nucleotide Exchange Factor
2874.	TNNT1	Troponin T1, Slow Skeletal Type
2875.	UQCRQ	Ubiquinol-Cytochrome C Reductase Complex III Subunit VII
2876.	KLHL40	Kelch Like Family Member 40
2877.	LYRM4	LYR Motif Containing 4
2878.	MYO18B	Myosin XVIIIB
2879.	SMPX	Small Muscle Protein X-Linked
2880.	PAX2	Paired Box 2
2881.	PITX3	Paired Like Homeodomain 3
2882.	PNLIP	Pancreatic Lipase
2883.	CTNS	Cystinosin, Lysosomal Cystine Transporter
2884.	POLR1C	RNA Polymerase I And III Subunit C
2885.	DTNBP1	Dystrobrevin Binding Protein 1
2886.	HPS1	HPS1 Biogenesis Of Lysosomal Organelles Complex 3 Subunit 1
2887.	HPS3	HPS3 Biogenesis Of Lysosomal Organelles Complex 2 Subunit 1
2888.	HPS4	HPS4 Biogenesis Of Lysosomal Organelles Complex 3 Subunit 2
2889.	ENTPD1	Ectonucleoside Triphosphate Diphosphohydrolase 1
2890.	CAST	Calpastatin
2891.	DDX3X	DEAD-Box Helicase 3 X-Linked
2892.	MAF	MAF BZIP Transcription Factor
2893.	SLC19A3	Solute Carrier Family 19 Member 3
2894.	SOD3	Superoxide Dismutase 3
2895.	FXYD2	FXYD Domain Containing Ion Transport Regulator 2
2896.	MAPRE2	Microtubule Associated Protein RP/EB Family Member 2
2897.	BST2	Bone Marrow Stromal Cell Antigen 2
2898.	CD48	CD48 Molecule
2899.	CYGB	Cytoglobin
2900.	RIT2	Ras Like Without CAAX 2
2901.	TMEM230	Transmembrane Protein 230
2902.	SLC25A20	Solute Carrier Family 25 Member 20
2903.	PPP2CA	Protein Phosphatase 2 Catalytic Subunit Alpha
2904.	ACAN	Aggrecan

2905.	NCF1	Neutrophil Cytosolic Factor 1
2906.	RASA1	RAS P21 Protein Activator 1
2907.	CACNA1F	Calcium Voltage-Gated Channel Subunit Alpha1 F
2908.	ROR1	Receptor Tyrosine Kinase Like Orphan Receptor 1
2909.	TLE1	TLE Family Member 1, Transcriptional Corepressor
2910.	ELOC	Elongin C
2911.	P4HA1	Prolyl 4-Hydroxylase Subunit Alpha 1
2912.	CD37	CD37 Molecule
2913.	COL10A1	Collagen Type X Alpha 1 Chain
2914.	GAB2	GRB2 Associated Binding Protein 2
2915.	CCRL2	C-C Motif Chemokine Receptor Like 2
2916.	DHX58	DExH-Box Helicase 58
2917.	HTATIP2	HIV-1 Tat Interactive Protein 2
2918.	SIPA1	Signal-Induced Proliferation-Associated 1
2919.	PLA2R1	Phospholipase A2 Receptor 1
2920.	S100A12	S100 Calcium Binding Protein A12
2921.	SOCS6	Suppressor Of Cytokine Signaling 6
2922.	SLC2A12	Solute Carrier Family 2 Member 12
2923.	EBAG9	Estrogen Receptor Binding Site Associated Antigen 9
2924.	SAA2	Serum Amyloid A2
2925.	SFXN4	Sideroflexin 4
2926.	TCL1B	TCL1 Family AKT Coactivator B
2927.	IGLL5	Immunoglobulin Lambda Like Polypeptide 5
2928.	RIPK1	Receptor Interacting Serine/Threonine Kinase 1
2929.	CAMK2G	Calcium/Calmodulin Dependent Protein Kinase II Gamma
2930.	ITGB4	Integrin Subunit Beta 4
2931.	KCNK3	Potassium Two Pore Domain Channel Subfamily K Member 3
2932.	CPS1	Carbamoyl-Phosphate Synthase 1
2933.	PKD2	Polycystin 2, Transient Receptor Potential Cation Channel
2934.	ADCY5	Adenylate Cyclase 5
2935.	CPE	Carboxypeptidase E
2936.	HCK	HCK Proto-Oncogene, Src Family Tyrosine Kinase
2937.	IL2RG	Interleukin 2 Receptor Subunit Gamma
2938.	IRF7	Interferon Regulatory Factor 7
2939.	TAP1	Transporter 1, ATP Binding Cassette Subfamily B Member
2940.	ZEB2	Zinc Finger E-Box Binding Homeobox 2
2941.	CACNB2	Calcium Voltage-Gated Channel Auxiliary Subunit Beta 2
2942.	FEN1	Flap Structure-Specific Endonuclease 1
2943.	GJB1	Gap Junction Protein Beta 1
2944.	LHCGR	Luteinizing Hormone/Choriogonadotropin Receptor
2945.	NFATC2	Nuclear Factor Of Activated T Cells 2

2946.	PRKAR1B	Protein Kinase CAMP-Dependent Type I Regulatory Subunit Beta
2947.	RPSA	Ribosomal Protein SA
2948.	RXRB	Retinoid X Receptor Beta
2949.	BID	BH3 Interacting Domain Death Agonist
2950.	LPAR1	Lysophosphatidic Acid Receptor 1
2951.	PTGER4	Prostaglandin E Receptor 4
2952.	ADAM12	ADAM Metallopeptidase Domain 12
2953.	ADH4	Alcohol Dehydrogenase 4 (Class II), Pi Polypeptide
2954.	ASL	Argininosuccinate Lyase
2955.	ATP6V1A	ATPase H+ Transporting V1 Subunit A
2956.	CD82	CD82 Molecule
2957.	CTNND1	Catenin Delta 1
2958.	DDB1	Damage Specific DNA Binding Protein 1
2959.	ICOS	Inducible T Cell Costimulator
2960.	ITPR2	Inositol 1,4,5-Trisphosphate Receptor Type 2
2961.	MTRR	5-Methyltetrahydrofolate-Homocysteine Methyltransferase Reductase
2962.	PNPO	Pyridoxamine 5'-Phosphate Oxidase
2963.	RPA2	Replication Protein A2
2964.	TAP2	Transporter 2, ATP Binding Cassette Subfamily B Member
2965.	TBXT	T-Box Transcription Factor T
2966.	AREG	Amphiregulin
2967.	AVPR1B	Arginine Vasopressin Receptor 1B
2968.	LTK	Leukocyte Receptor Tyrosine Kinase
2969.	NARS1	Asparaginyl-TRNA Synthetase 1
2970.	PCSK2	Proprotein Convertase Subtilisin/Kexin Type 2
2971.	RAD17	RAD17 Checkpoint Clamp Loader Component
2972.	SERPINB5	Serpin Family B Member 5
2973.	SLC39A8	Solute Carrier Family 39 Member 8
2974.	SOST	Sclerostin
2975.	ZFHX3	Zinc Finger Homeobox 3
2976.	ACKR1	Atypical Chemokine Receptor 1 (Duffy Blood Group)
2977.	ADH1A	Alcohol Dehydrogenase 1A (Class I), Alpha Polypeptide
2978.	ALAS1	5'-Aminolevulinate Synthase 1
2979.	ASIC2	Acid Sensing Ion Channel Subunit 2
2980.	CRABP2	Cellular Retinoic Acid Binding Protein 2
2981.	DPP10	Dipeptidyl Peptidase Like 10
2982.	EREG	Epiregulin
2983.	ID2	Inhibitor Of DNA Binding 2
2984.	LMNB2	Lamin B2
2985.	MDC1	Mediator Of DNA Damage Checkpoint 1
2986.	PYGB	Glycogen Phosphorylase B

2987.	SLC52A3	Solute Carrier Family 52 Member 3
2988.	SOX17	SRY-Box Transcription Factor 17
2989.	TNFRSF10D	TNF Receptor Superfamily Member 10d
2990.	TPP2	Tripeptidyl Peptidase 2
2991.	TRADD	TNFRSF1A Associated Via Death Domain
2992.	ADD2	Adducin 2
2993.	CLDN14	Claudin 14
2994.	CLSPN	Claspin
2995.	CTSE	Cathepsin E
2996.	ELAVL1	ELAV Like RNA Binding Protein 1
2997.	FABP6	Fatty Acid Binding Protein 6
2998.	HLTF	Helicase Like Transcription Factor
2999.	NEDD9	Neural Precursor Cell Expressed, Developmentally Down-Regulated 9
3000.	RAG2	Recombination Activating 2
3001.	RASGRP3	RAS Guanyl Releasing Protein 3
3002.	SLC30A2	Solute Carrier Family 30 Member 2
3003.	SLC30A9	Solute Carrier Family 30 Member 9
3004.	SPINT2	Serine Peptidase Inhibitor, Kunitz Type 2
3005.	TOPBP1	DNA Topoisomerase II Binding Protein 1
3006.	ABCB10	ATP Binding Cassette Subfamily B Member 10
3007.	ARID2	AT-Rich Interaction Domain 2
3008.	ASGR1	Asialoglycoprotein Receptor 1
3009.	CFHR3	Complement Factor H Related 3
3010.	HHIP	Hedgehog Interacting Protein
3011.	MELTF	Melanotransferrin
3012.	MR1	Major Histocompatibility Complex, Class I-Related
3013.	PANK1	Pantothenate Kinase 1
3014.	TEX14	Testis Expressed 14, Intercellular Bridge Forming Factor
3015.	ASGR2	Asialoglycoprotein Receptor 2
3016.	DNMT3L	DNA Methyltransferase 3 Like
3017.	DPYSL5	Dihydropyrimidinase Like 5
3018.	HLA-DRB5	Major Histocompatibility Complex, Class II, DR Beta 5
3019.	IFIT3	Interferon Induced Protein With Tetratricopeptide Repeats 3
3020.	PCBP2	Poly(RC) Binding Protein 2
3021.	RPLP2	Ribosomal Protein Lateral Stalk Subunit P2
3022.	THBS3	Thrombospondin 3
3023.	TSC22D3	TSC22 Domain Family Member 3
3024.	WDR48	WD Repeat Domain 48
3025.	ALYREF	Aly/REF Export Factor
3026.	EML4	EMAP Like 4
3027.	HSD17B13	Hydroxysteroid 17-Beta Dehydrogenase 13

3028.	ISCA1	Iron-Sulfur Cluster Assembly 1
3029.	RNF4	Ring Finger Protein 4
3030.	SNRPD1	Small Nuclear Ribonucleoprotein D1 Polypeptide
3031.	SSRP1	Structure Specific Recognition Protein 1
3032.	CENPS	Centromere Protein S
3033.	FAM167A	Family With Sequence Similarity 167 Member A
3034.	HSCB	HscB Mitochondrial Iron-Sulfur Cluster Cochaperone
3035.	NPRL3	NPR3 Like, GATOR1 Complex Subunit
3036.	TNNI3K	TNNI3 Interacting Kinase
3037.	PNMA2	PNMA Family Member 2
3038.	SH2D3A	SH2 Domain Containing 3A
3039.	MYOZ1	Myozenin 1
3040.	PSG2	Pregnancy Specific Beta-1-Glycoprotein 2
3041.	SPTBN5	Spectrin Beta, Non-Erythrocytic 5
3042.	RANGRF	RAN Guanine Nucleotide Release Factor
3043.	CLEC4C	C-Type Lectin Domain Family 4 Member C
3044.	NAA80	N-Alpha-Acetyltransferase 80, NatH Catalytic Subunit
3045.	KLHDC10	Kelch Domain Containing 10
3046.	MTNAP1	Mitochondrial Nucleoid Associated Protein 1
3047.	TIGD4	Tigger Transposable Element Derived 4
3048.	PRH2	Proline Rich Protein HaeIII Subfamily 2
3049.	HLA-DRB4	Major Histocompatibility Complex, Class II, DR Beta 4
3050.	TRIM75	Tripartite Motif Containing 75
3051.	C4B_2	Complement Component 4B (Chido/Rodgers Blood Group), Copy 2
3052.	HRH1	Histamine Receptor H1
3053.	CYP3A5	Cytochrome P450 Family 3 Subfamily A Member 5
3054.	TRPV3	Transient Receptor Potential Cation Channel Subfamily V Member 3
3055.	TRPV2	Transient Receptor Potential Cation Channel Subfamily V Member 2
3056.	ASIC1	Acid Sensing Ion Channel Subunit 1
3057.	GYS2	Glycogen Synthase 2
3058.	RAP1A	RAP1A, Member Of RAS Oncogene Family
3059.	RHOB	Ras Homolog Family Member B
3060.	SLC22A2	Solute Carrier Family 22 Member 2
3061.	DUX4	Double Homeobox 4
3062.	TUBB	Tubulin Beta Class I
3063.	SCN3A	Sodium Voltage-Gated Channel Alpha Subunit 3
3064.	LINGO1	Leucine Rich Repeat And Ig Domain Containing 1
3065.	WNT2	Wnt Family Member 2
3066.	LPXN	Leupaxin
3067.	REEP2	Receptor Accessory Protein 2
3068.	CES1	Carboxylesterase 1

3069.	LRP1	LDL Receptor Related Protein 1
3070.	MC1R	Melanocortin 1 Receptor
3071.	TGIF1	TGFB Induced Factor Homeobox 1
3072.	ARF1	ADP Ribosylation Factor 1
3073.	HSPB8	Heat Shock Protein Family B (Small) Member 8
3074.	TEC	Tec Protein Tyrosine Kinase
3075.	BTC	Betacellulin
3076.	CELF2	CUGBP Elav-Like Family Member 2
3077.	EFEMP2	EGF Containing Fibulin Extracellular Matrix Protein 2
3078.	FGF9	Fibroblast Growth Factor 9
3079.	PURA	Purine Rich Element Binding Protein A
3080.	STXBP2	Syntaxin Binding Protein 2
3081.	TBX2	T-Box Transcription Factor 2
3082.	ABCC5	ATP Binding Cassette Subfamily C Member 5
3083.	NEFM	Neurofilament Medium Chain
3084.	NOP56	NOP56 Ribonucleoprotein
3085.	PRSS1	Serine Protease 1
3086.	SCN7A	Sodium Voltage-Gated Channel Alpha Subunit 7
3087.	SFRP4	Secreted Frizzled Related Protein 4
3088.	CALD1	Caldesmon 1
3089.	CCS	Copper Chaperone For Superoxide Dismutase
3090.	CES2	Carboxylesterase 2
3091.	IVD	Isovaleryl-CoA Dehydrogenase
3092.	LOXL1	Lysyl Oxidase Like 1
3093.	MARS2	Methionyl-TRNA Synthetase 2, Mitochondrial
3094.	PLAA	Phospholipase A2 Activating Protein
3095.	SNAP23	Synaptosome Associated Protein 23
3096.	UNC13D	Unc-13 Homolog D
3097.	BICD2	BICD Cargo Adaptor 2
3098.	NTSR1	Neurotensin Receptor 1
3099.	PTN	Pleiotrophin
3100.	VAMP7	Vesicle Associated Membrane Protein 7
3101.	CCNG1	Cyclin G1
3102.	FTCD	Formimidoyltransferase Cyclodeaminase
3103.	G3BP1	G3BP Stress Granule Assembly Factor 1
3104.	RSAD2	Radical S-Adenosyl Methionine Domain Containing 2
3105.	SIGLEC8	Sialic Acid Binding Ig Like Lectin 8
3106.	ACKR2	Atypical Chemokine Receptor 2
3107.	CLDN5	Claudin 5
3108.	KLB	Klotho Beta
3109.	MBNL1	Muscleblind Like Splicing Regulator 1

3110.	NBAS	NBAS Subunit Of NRZ Tethering Complex
3111.	STX11	Syntaxin 11
3112.	ZFPM2	Zinc Finger Protein, FOG Family Member 2
3113.	ATP6V1G3	ATPase H+ Transporting V1 Subunit G3
3114.	DNAJC12	DnaJ Heat Shock Protein Family (Hsp40) Member C12
3115.	INA	Internexin Neuronal Intermediate Filament Protein Alpha
3116.	KRT86	Keratin 86
3117.	NAIP	NLR Family Apoptosis Inhibitory Protein
3118.	OAS3	2'-5'-Oligoadenylate Synthetase 3
3119.	MORC3	MORC Family CW-Type Zinc Finger 3
3120.	NAV2	Neuron Navigator 2
3121.	SH3TC2	SH3 Domain And Tetratricopeptide Repeats 2
3122.	TIMM9	Translocase Of Inner Mitochondrial Membrane 9
3123.	FNBP1	Formin Binding Protein 1
3124.	PIGW	Phosphatidylinositol Glycan Anchor Biosynthesis Class W
3125.	TIMM10	Translocase Of Inner Mitochondrial Membrane 10
3126.	CGAS	Cyclic GMP-AMP Synthase
3127.	ATXN3L	Ataxin 3 Like
3128.	CNKSR3	CNKSR Family Member 3
3129.	CORT	Cortistatin
3130.	SSX2	SSX Family Member 2
3131.	OR14C36	Olfactory Receptor Family 14 Subfamily C Member 36
3132.	NBPF11	NBPF Member 11
3133.	OR5AS1	Olfactory Receptor Family 5 Subfamily AS Member 1
3134.	AURKB	Aurora Kinase B
3135.	SRCAP	Snf2 Related CREBBP Activator Protein
3136.	GTPBP3	GTP Binding Protein 3, Mitochondrial
3137.	GRIN2D	Glutamate Ionotropic Receptor NMDA Type Subunit 2D
3138.	KDM6A	Lysine Demethylase 6A
3139.	CFD	Complement Factor D
3140.	AMBP	Alpha-1-Microglobulin/Bikunin Precursor
3141.	CDKL5	Cyclin Dependent Kinase Like 5
3142.	STK24	Serine/Threonine Kinase 24
3143.	RPGRIP1L	RPGRIP1 Like
3144.	AAK1	AP2 Associated Kinase 1
3145.	HPX	Hemopexin
3146.	CC2D2A	Coiled-Coil And C2 Domain Containing 2A
3147.	OXSR1	Oxidative Stress Responsive Kinase 1
3148.	KCNJ16	Potassium Inwardly Rectifying Channel Subfamily J Member 16
3149.	MAPRE3	Microtubule Associated Protein RP/EB Family Member 3
3150.	MLN	Motilin

3151.	BMAL1	Basic Helix-Loop-Helix ARNT Like 1
3152.	PGAM2	Phosphoglycerate Mutase 2
3153.	ALDH1A1	Aldehyde Dehydrogenase 1 Family Member A1
3154.	DUSP19	Dual Specificity Phosphatase 19
3155.	CPN1	Carboxypeptidase N Subunit 1
3156.	BLOC1S5	Biogenesis Of Lysosomal Organelles Complex 1 Subunit 5
3157.	HCRTR2	Hypocretin Receptor 2
3158.	APOBEC3G	Apolipoprotein B MRNA Editing Enzyme Catalytic Subunit 3G
3159.	ADAM10	ADAM Metallopeptidase Domain 10
3160.	EPHA2	EPH Receptor A2
3161.	PHGDH	Phosphoglycerate Dehydrogenase
3162.	ITGA6	Integrin Subunit Alpha 6
3163.	PRDX1	Peroxiredoxin 1
3164.	DLG4	Discs Large MAGUK Scaffold Protein 4
3165.	MAPK9	Mitogen-Activated Protein Kinase 9
3166.	PRKCQ	Protein Kinase C Theta
3167.	EEF2	Eukaryotic Translation Elongation Factor 2
3168.	IGF2R	Insulin Like Growth Factor 2 Receptor
3169.	PAH	Phenylalanine Hydroxylase
3170.	PIN1	Peptidylprolyl Cis/Trans Isomerase, NIMA-Interacting 1
3171.	PPT1	Palmitoyl-Protein Thioesterase 1
3172.	CDH3	Cadherin 3
3173.	COL18A1	Collagen Type XVIII Alpha 1 Chain
3174.	GP9	Glycoprotein IX Platelet
3175.	KDM3B	Lysine Demethylase 3B
3176.	LAT	Linker For Activation Of T Cells
3177.	NOTCH4	Notch Receptor 4
3178.	SEMA3A	Semaphorin 3A
3179.	ADH1B	Alcohol Dehydrogenase 1B (Class I), Beta Polypeptide
3180.	EIF2S1	Eukaryotic Translation Initiation Factor 2 Subunit Alpha
3181.	FOSL1	FOS Like 1, AP-1 Transcription Factor Subunit
3182.	LMAN1	Lectin, Mannose Binding 1
3183.	PHB1	Prohibitin 1
3184.	PI4KB	Phosphatidylinositol 4-Kinase Beta
3185.	SOX9	SRY-Box Transcription Factor 9
3186.	TAT	Tyrosine Aminotransferase
3187.	CAMK4	Calcium/Calmodulin Dependent Protein Kinase IV
3188.	CLCN5	Chloride Voltage-Gated Channel 5
3189.	CLCN7	Chloride Voltage-Gated Channel 7
3190.	CYP7B1	Cytochrome P450 Family 7 Subfamily B Member 1
3191.	EPHX1	Epoxide Hydrolase 1

3192.	FSHB	Follicle Stimulating Hormone Subunit Beta
3193.	NAT1	N-Acetyltransferase 1
3194.	NT5C3A	5'-Nucleotidase, Cytosolic IIIA
3195.	REV3L	REV3 Like, DNA Directed Polymerase Zeta Catalytic Subunit
3196.	SCO1	Synthesis Of Cytochrome C Oxidase 1
3197.	SERPINB2	Serpin Family B Member 2
3198.	SLC31A1	Solute Carrier Family 31 Member 1
3199.	USP1	Ubiquitin Specific Peptidase 1
3200.	AP2M1	Adaptor Related Protein Complex 2 Subunit Mu 1
3201.	B3GAT3	Beta-1,3-Glucuronyltransferase 3
3202.	CD63	CD63 Molecule
3203.	CRK	CRK Proto-Oncogene, Adaptor Protein
3204.	FCN3	Ficolin 3
3205.	GPD1L	Glycerol-3-Phosphate Dehydrogenase 1 Like
3206.	HSD3B1	Hydroxy-Delta-5-Steroid Dehydrogenase, 3 Beta- And Steroid Delta-Isomerase 1
3207.	MMP11	Matrix Metallopeptidase 11
3208.	NDUFA9	NADH:Ubiquinone Oxidoreductase Subunit A9
3209.	NFASC	Neurofascin
3210.	POLL	DNA Polymerase Lambda
3211.	REST	RE1 Silencing Transcription Factor
3212.	SLC22A1	Solute Carrier Family 22 Member 1
3213.	TFF1	Trefoil Factor 1
3214.	TRIM25	Tripartite Motif Containing 25
3215.	ABL2	ABL Proto-Oncogene 2, Non-Receptor Tyrosine Kinase
3216.	ADD3	Adducin 3
3217.	CCR8	C-C Motif Chemokine Receptor 8
3218.	CDH17	Cadherin 17
3219.	CHIA	Chitinase Acidic
3220.	COX6A1	Cytochrome C Oxidase Subunit 6A1
3221.	CPA1	Carboxypeptidase A1
3222.	CTNNA3	Catenin Alpha 3
3223.	CYSLTR1	Cysteinyl Leukotriene Receptor 1
3224.	EPB41L1	Erythrocyte Membrane Protein Band 4.1 Like 1
3225.	FADS1	Fatty Acid Desaturase 1
3226.	GM2A	Ganglioside GM2 Activator
3227.	HSPA2	Heat Shock Protein Family A (Hsp70) Member 2
3228.	KCNQ4	Potassium Voltage-Gated Channel Subfamily Q Member 4
3229.	LITAF	Lipopolysaccharide Induced TNF Factor
3230.	NFIA	Nuclear Factor I A
3231.	RNF8	Ring Finger Protein 8
3232.	SRSF1	Serine And Arginine Rich Splicing Factor 1

3233.	TRIM33	Tripartite Motif Containing 33
3234.	UCP1	Uncoupling Protein 1
3235.	XRCC4	X-Ray Repair Cross Complementing 4
3236.	ACKR3	Atypical Chemokine Receptor 3
3237.	ASAH2	N-Acylsphingosine Amidohydrolase 2
3238.	CHST14	Carbohydrate Sulfotransferase 14
3239.	CLDN4	Claudin 4
3240.	COX20	Cytochrome C Oxidase Assembly Factor COX20
3241.	DOK1	Docking Protein 1
3242.	IL24	Interleukin 24
3243.	JPH3	Junctophilin 3
3244.	KCNK4	Potassium Two Pore Domain Channel Subfamily K Member 4
3245.	LGALS3BP	Galectin 3 Binding Protein
3246.	NHEJ1	Non-Homologous End Joining Factor 1
3247.	NTNG2	Netrin G2
3248.	OSGEP	O-Sialoglycoprotein Endopeptidase
3249.	PRSS12	Serine Protease 12
3250.	RAD18	RAD18 E3 Ubiquitin Protein Ligase
3251.	RAD52	RAD52 Homolog, DNA Repair Protein
3252.	RECQL	RecQ Like Helicase
3253.	REV1	REV1 DNA Directed Polymerase
3254.	ST8SIA2	ST8 Alpha-N-Acetyl-Neuraminide Alpha-2,8-Sialyltransferase 2
3255.	STING1	Stimulator Of Interferon Response CGAMP Interactor 1
3256.	SULF1	Sulfatase 1
3257.	TBX4	T-Box Transcription Factor 4
3258.	UBE2K	Ubiquitin Conjugating Enzyme E2 K
3259.	VPS13B	Vacuolar Protein Sorting 13 Homolog B
3260.	C4BPA	Complement Component 4 Binding Protein Alpha
3261.	CCR9	C-C Motif Chemokine Receptor 9
3262.	CELF1	CUGBP Elav-Like Family Member 1
3263.	CHGB	Chromogranin B
3264.	CRABP1	Cellular Retinoic Acid Binding Protein 1
3265.	EIF6	Eukaryotic Translation Initiation Factor 6
3266.	JUNB	JunB Proto-Oncogene, AP-1 Transcription Factor Subunit
3267.	MADCAM1	Mucosal Vascular Addressin Cell Adhesion Molecule 1
3268.	NHLRC1	NHL Repeat Containing E3 Ubiquitin Protein Ligase 1
3269.	NSMF	NMDA Receptor Synaptonuclear Signaling And Neuronal Migration Factor
3270.	PRPH2	Peripherin 2
3271.	RAD9A	RAD9 Checkpoint Clamp Component A
3272.	RFX1	Regulatory Factor X1
3273.	RPL4	Ribosomal Protein L4

3274.	ATP1A4	ATPase Na+/K+ Transporting Subunit Alpha 4
3275.	CD180	CD180 Molecule
3276.	CLC	Charcot-Leyden Crystal Galectin
3277.	CLDN7	Claudin 7
3278.	CREM	CAMP Responsive Element Modulator
3279.	CRIPTO	Cripto, EGF-CFC Family Member
3280.	EFNA2	Ephrin A2
3281.	ELF1	E74 Like ETS Transcription Factor 1
3282.	NEUROG3	Neurogenin 3
3283.	NFYA	Nuclear Transcription Factor Y Subunit Alpha
3284.	POLM	DNA Polymerase Mu
3285.	PSCA	Prostate Stem Cell Antigen
3286.	PSME3	Proteasome Activator Subunit 3
3287.	RMI1	RecQ Mediated Genome Instability 1
3288.	SEC24B	SEC24 Homolog B, COPII Coat Complex Component
3289.	TFF2	Trefoil Factor 2
3290.	TRIM38	Tripartite Motif Containing 38
3291.	UBQLN4	Ubiquilin 4
3292.	USP18	Ubiquitin Specific Peptidase 18
3293.	ZMPSTE24	Zinc Metallopeptidase STE24
3294.	ABCF1	ATP Binding Cassette Subfamily F Member 1
3295.	ACBD3	Acyl-CoA Binding Domain Containing 3
3296.	BAG6	BAG Cochaperone 6
3297.	CD207	CD207 Molecule
3298.	DECR1	2,4-Dienoyl-CoA Reductase 1
3299.	GLDN	Gliomedin
3300.	GNA15	G Protein Subunit Alpha 15
3301.	GOLPH3	Golgi Phosphoprotein 3
3302.	IBSP	Integrin Binding Sialoprotein
3303.	IL36G	Interleukin 36 Gamma
3304.	MT1E	Metallothionein 1E
3305.	NEGR1	Neuronal Growth Regulator 1
3306.	NR6A1	Nuclear Receptor Subfamily 6 Group A Member 1
3307.	SH2D2A	SH2 Domain Containing 2A
3308.	TMOD1	Tropomodulin 1
3309.	BTN2A1	Butyrophilin Subfamily 2 Member A1
3310.	H1-5	H1.5 Linker Histone, Cluster Member
3311.	HSPA1B	Heat Shock Protein Family A (Hsp70) Member 1B
3312.	IER3	Immediate Early Response 3
3313.	IL36A	Interleukin 36 Alpha
3314.	INTS11	Integrator Complex Subunit 11

3315.	LGALS8	Galectin 8
3316.	NT5C1A	5'-Nucleotidase, Cytosolic IA
3317.	PATZ1	POZ/BTB And AT Hook Containing Zinc Finger 1
3318.	REG3A	Regenerating Family Member 3 Alpha
3319.	SDF4	Stromal Cell Derived Factor 4
3320.	SELENOI	Selenoprotein I
3321.	SLC52A2	Solute Carrier Family 52 Member 2
3322.	STK19	Serine/Threonine Kinase 19
3323.	TMOD3	Tropomodulin 3
3324.	ACAP3	ArfGAP With Coiled-Coil, Ankyrin Repeat And PH Domains 3
3325.	CACYBP	Calcyclin Binding Protein
3326.	CENPC	Centromere Protein C
3327.	DCHS1	Dachsous Cadherin-Related 1
3328.	HIC2	HIC ZBTB Transcriptional Repressor 2
3329.	HLA-DQB2	Major Histocompatibility Complex, Class II, DQ Beta 2
3330.	IL4I1	Interleukin 4 Induced 1
3331.	MTX1	Metaxin 1
3332.	MYL7	Myosin Light Chain 7
3333.	SH2D3C	SH2 Domain Containing 3C
3334.	SLC15A4	Solute Carrier Family 15 Member 4
3335.	SOX8	SRY-Box Transcription Factor 8
3336.	BTN3A2	Butyrophilin Subfamily 3 Member A2
3337.	DCLRE1A	DNA Cross-Link Repair 1A
3338.	FAAP24	FA Core Complex Associated Protein 24
3339.	TMEM11	Transmembrane Protein 11
3340.	TRIM17	Tripartite Motif Containing 17
3341.	TUT1	Terminal Uridylyl Transferase 1, U6 SnRNA-Specific
3342.	ZNF436	Zinc Finger Protein 436
3343.	ATP6V1G2	ATPase H+ Transporting V1 Subunit G2
3344.	ELMOD2	ELMO Domain Containing 2
3345.	FAAP100	FA Core Complex Associated Protein 100
3346.	GCN1	GCN1 Activator Of EIF2AK4
3347.	IL17C	Interleukin 17C
3348.	IL36B	Interleukin 36 Beta
3349.	TCF19	Transcription Factor 19
3350.	UTP4	UTP4 Small Subunit Processome Component
3351.	DXO	Decapping Exoribonuclease
3352.	GBA3	Glucosylceramidase Beta 3 (Gene/Pseudogene)
3353.	KCP	Kielin Cysteine Rich BMP Regulator
3354.	PHRF1	PHD And Ring Finger Domains 1
3355.	TRAT1	T Cell Receptor Associated Transmembrane Adaptor 1

3356.	BLTP3A	Bridge-Like Lipid Transfer Protein Family Member 3A
3357.	FCGR2C	Fc Gamma Receptor IIc (Gene/Pseudogene)
3358.	GPSM3	G Protein Signaling Modulator 3
3359.	LEPROT	Leptin Receptor Overlapping Transcript
3360.	ZNF44	Zinc Finger Protein 44
3361.	H2BC15	H2B Clustered Histone 15
3362.	H3C3	H3 Clustered Histone 3
3363.	H3C6	H3 Clustered Histone 6
3364.	C1orf159	Chromosome 1 Open Reading Frame 159
3365.	PUSL1	Pseudouridine Synthase Like 1
3366.	TTLL10	Tubulin Tyrosine Ligase Like 10
3367.	ZNF569	Zinc Finger Protein 569
3368.	ZNF629	Zinc Finger Protein 629
3369.	PSORS1C1	Psoriasis Susceptibility 1 Candidate 1
3370.	TSBP1	Testis Expressed Basic Protein 1
3371.	C1QTNF12	C1q And TNF Related 12
3372.	LCE3C	Late Cornified Envelope 3C
3373.	NKG7	Natural Killer Cell Granule Protein 7
3374.	SLX1A	SLX1 Homolog A, Structure-Specific Endonuclease Subunit
3375.	ZNF501	Zinc Finger Protein 501
3376.	ZNF763	Zinc Finger Protein 763
3377.	LCE3B	Late Cornified Envelope 3B
3378.	RNF223	Ring Finger Protein 223
3379.	SPMIP7	Sperm Microtubule Inner Protein 7
3380.	MGAM2	Maltase-Glucoamylase 2 (Putative)
3381.	H3Y2	H3.Y Histone 2
3382.	NCOA2	Nuclear Receptor Coactivator 2
3383.	TUBA1B	Tubulin Alpha 1b
3384.	MYOZ2	Myozenin 2
3385.	TIGIT	T Cell Immunoreceptor With Ig And ITIM Domains
3386.	DAOA	D-Amino Acid Oxidase Activator
3387.	HDAC8	Histone Deacetylase 8
3388.	DNAJB2	DnaJ Heat Shock Protein Family (Hsp40) Member B2
3389.	IGHMBP2	Immunoglobulin Mu DNA Binding Protein 2
3390.	FOXF1	Forkhead Box F1
3391.	BLOC1S6	Biogenesis Of Lysosomal Organelles Complex 1 Subunit 6
3392.	SLC25A42	Solute Carrier Family 25 Member 42
3393.	AK9	Adenylate Kinase 9
3394.	AMH	Anti-Mullerian Hormone
3395.	PDK4	Pyruvate Dehydrogenase Kinase 4
3396.	ABCB6	ATP Binding Cassette Subfamily B Member 6 (LAN Blood Group)

3397.	SIX3	SIX Homeobox 3
3398.	RRAS	RAS Related
3399.	SIX6	SIX Homeobox 6
3400.	VAX1	Ventral Anterior Homeobox 1
3401.	SERPING1	Serpin Family G Member 1
3402.	ARNT	Aryl Hydrocarbon Receptor Nuclear Translocator
3403.	EGLN3	Egl-9 Family Hypoxia Inducible Factor 3
3404.	PTS	6-Pyruvoyltetrahydropterin Synthase
3405.	EGLN2	Egl-9 Family Hypoxia Inducible Factor 2
3406.	ADAMTS4	ADAM Metallopeptidase With Thrombospondin Type 1 Motif 4
3407.	COL9A3	Collagen Type IX Alpha 3 Chain
3408.	SLC26A2	Solute Carrier Family 26 Member 2
3409.	COL9A1	Collagen Type IX Alpha 1 Chain
3410.	HGFAC	HGF Activator
3411.	TNFRSF12A	TNF Receptor Superfamily Member 12A
3412.	SHOC2	SHOC2 Leucine Rich Repeat Scaffold Protein
3413.	PRG2	Proteoglycan 2, Pro Eosinophil Major Basic Protein
3414.	CACNG1	Calcium Voltage-Gated Channel Auxiliary Subunit Gamma 1
3415.	CAPZB	Capping Actin Protein Of Muscle Z-Line Subunit Beta
3416.	DDHD2	DDHD Domain Containing 2
3417.	EIF5B	Eukaryotic Translation Initiation Factor 5B
3418.	NLRP5	NLR Family Pyrin Domain Containing 5
3419.	HOXA2	Homeobox A2
3420.	LALBA	Lactalbumin Alpha
3421.	TRIM8	Tripartite Motif Containing 8
3422.	CD5L	CD5 Molecule Like
3423.	NELFA	Negative Elongation Factor Complex Member A
3424.	TBPL1	TATA-Box Binding Protein Like 1
3425.	IL26	Interleukin 26
3426.	LECT2	Leukocyte Cell Derived Chemotaxin 2
3427.	CHCHD4	Coiled-Coil-Helix-Coiled-Coil-Helix Domain Containing 4
3428.	IFI44	Interferon Induced Protein 44
3429.	CSH2	Chorionic Somatomammotropin Hormone 2
3430.	ANKAR	Ankyrin And Armadillo Repeat Containing
3431.	NEMP2	Nuclear Envelope Integral Membrane Protein 2
3432.	KRT5	Keratin 5
3433.	HTRA2	HtrA Serine Peptidase 2
3434.	PDYN	Prodynorphin
3435.	FOXM1	Forkhead Box M1
3436.	AHSP	Alpha Hemoglobin Stabilizing Protein
3437.	NFATC1	Nuclear Factor Of Activated T Cells 1

3438.	ADAM17	ADAM Metallopeptidase Domain 17
3439.	NAMPT	Nicotinamide Phosphoribosyltransferase
3440.	ACOX1	Acyl-CoA Oxidase 1
3441.	HTRA1	HtrA Serine Peptidase 1
3442.	TK1	Thymidine Kinase 1
3443.	GBE1	1,4-Alpha-Glucan Branching Enzyme 1
3444.	TSG101	Tumor Susceptibility 101
3445.	BCKDHB	Branched Chain Keto Acid Dehydrogenase E1 Subunit Beta
3446.	BST1	Bone Marrow Stromal Cell Antigen 1
3447.	PDE3B	Phosphodiesterase 3B
3448.	BAG1	BAG Cochaperone 1
3449.	NPHS2	NPHS2 Stomatin Family Member, Podocin
3450.	PTGES	Prostaglandin E Synthase
3451.	SLC23A2	Solute Carrier Family 23 Member 2
3452.	CCNT1	Cyclin T1
3453.	BIRC7	Baculoviral IAP Repeat Containing 7
3454.	CBY1	Chibby 1, Beta Catenin Antagonist
3455.	DIO2	Iodothyronine Deiodinase 2
3456.	ETF1	Eukaryotic Translation Termination Factor 1
3457.	PLEKHG4	Pleckstrin Homology And RhoGEF Domain Containing G4
3458.	CEACAM7	CEA Cell Adhesion Molecule 7
3459.	PER2	Period Circadian Regulator 2
3460.	MYLK2	Myosin Light Chain Kinase 2
3461.	ABAT	4-Aminobutyrate Aminotransferase
3462.	KCTD17	Potassium Channel Tetramerization Domain Containing 17
3463.	PRG4	Proteoglycan 4
3464.	TMEM67	Transmembrane Protein 67
3465.	APEH	Acylaminoacyl-Peptide Hydrolase
3466.	TDP2	Tyrosyl-DNA Phosphodiesterase 2
3467.	DAPK1	Death Associated Protein Kinase 1
3468.	RORA	RAR Related Orphan Receptor A
3469.	TIMELESS	Timeless Circadian Regulator
3470.	BMAL2	Basic Helix-Loop-Helix ARNT Like 2
3471.	DBP	D-Box Binding PAR BZIP Transcription Factor
3472.	CTCF	CCCTC-Binding Factor
3473.	SMC1A	Structural Maintenance Of Chromosomes 1A
3474.	FTO	FTO Alpha-Ketoglutarate Dependent Dioxygenase
3475.	MVP	Major Vault Protein
3476.	SLC17A6	Solute Carrier Family 17 Member 6
3477.	ALMS1	ALMS1 Centrosome And Basal Body Associated Protein
3478.	FLT3LG	Fms Related Receptor Tyrosine Kinase 3 Ligand

3479.	MTNR1A	Melatonin Receptor 1A
3480.	WNT5A	Wnt Family Member 5A
3481.	RAB11A	RAB11A, Member RAS Oncogene Family
3482.	FGF3	Fibroblast Growth Factor 3
3483.	NTHL1	Nth Like DNA Glycosylase 1
3484.	ICMT	Isoprenylcysteine Carboxyl Methyltransferase
3485.	AFAP1	Actin Filament Associated Protein 1
3486.	RABIF	RAB Interacting Factor
3487.	NUTM1	NUT Midline Carcinoma Family Member 1
3488.	GGH	Gamma-Glutamyl Hydrolase
3489.	ACAD10	Acyl-CoA Dehydrogenase Family Member 10
3490.	GRIA2	Glutamate Ionotropic Receptor AMPA Type Subunit 2
3491.	GLS	Glutaminase
3492.	ITPR1	Inositol 1,4,5-Trisphosphate Receptor Type 1
3493.	MAP3K1	Mitogen-Activated Protein Kinase Kinase Kinase 1
3494.	BMPR1B	Bone Morphogenetic Protein Receptor Type 1B
3495.	FST	Follistatin
3496.	IDE	Insulin Degrading Enzyme
3497.	ABCC6	ATP Binding Cassette Subfamily C Member 6
3498.	F13A1	Coagulation Factor XIII A Chain
3499.	FOLH1	Folate Hydrolase 1
3500.	GOT2	Glutamic-Oxaloacetic Transaminase 2
3501.	HSF1	Heat Shock Transcription Factor 1
3502.	MAP2K3	Mitogen-Activated Protein Kinase Kinase 3
3503.	ARHGEF2	Rho/Rac Guanine Nucleotide Exchange Factor 2
3504.	ATF4	Activating Transcription Factor 4
3505.	COL4A2	Collagen Type IV Alpha 2 Chain
3506.	GAMT	Guanidinoacetate N-Methyltransferase
3507.	KCND3	Potassium Voltage-Gated Channel Subfamily D Member 3
3508.	LIFR	LIF Receptor Subunit Alpha
3509.	MAPKAPK2	MAPK Activated Protein Kinase 2
3510.	NEDD4	NEDD4 E3 Ubiquitin Protein Ligase
3511.	PNKP	Polynucleotide Kinase 3'-Phosphatase
3512.	PTK6	Protein Tyrosine Kinase 6
3513.	RAB27A	RAB27A, Member RAS Oncogene Family
3514.	RAD54L	RAD54 Like
3515.	RALA	RAS Like Proto-Oncogene A
3516.	SLC5A1	Solute Carrier Family 5 Member 1
3517.	UNG	Uracil DNA Glycosylase
3518.	WNT10B	Wnt Family Member 10B
3519.	WNT3A	Wnt Family Member 3A

3520.	ANTXR2	ANTXR Cell Adhesion Molecule 2
3521.	ARG2	Arginase 2
3522.	CASP4	Caspase 4
3523.	CNR2	Cannabinoid Receptor 2
3524.	FLRT3	Fibronectin Leucine Rich Transmembrane Protein 3
3525.	GSS	Glutathione Synthetase
3526.	KCNH1	Potassium Voltage-Gated Channel Subfamily H Member 1
3527.	KISS1R	KISS1 Receptor
3528.	MCM4	Minichromosome Maintenance Complex Component 4
3529.	MTMR2	Myotubularin Related Protein 2
3530.	PSPH	Phosphoserine Phosphatase
3531.	SKI	SKI Proto-Oncogene
3532.	SORT1	Sortilin 1
3533.	SPHK1	Sphingosine Kinase 1
3534.	TGFBR3	Transforming Growth Factor Beta Receptor 3
3535.	TGM1	Transglutaminase 1
3536.	ACSL4	Acyl-CoA Synthetase Long Chain Family Member 4
3537.	ANTXR1	ANTXR Cell Adhesion Molecule 1
3538.	BGN	Biglycan
3539.	CPT1B	Carnitine Palmitoyltransferase 1B
3540.	DDX1	DEAD-Box Helicase 1
3541.	ETV1	ETS Variant Transcription Factor 1
3542.	FAM20C	FAM20C Golgi Associated Secretory Pathway Kinase
3543.	FGD4	FYVE, RhoGEF And PH Domain Containing 4
3544.	FZD7	Frizzled Class Receptor 7
3545.	GDF2	Growth Differentiation Factor 2
3546.	KLF5	KLF Transcription Factor 5
3547.	PPIF	Peptidylprolyl Isomerase F
3548.	PPP2R2B	Protein Phosphatase 2 Regulatory Subunit Bbeta
3549.	PTPRN2	Protein Tyrosine Phosphatase Receptor Type N2
3550.	RAD23B	RAD23 Homolog B, Nucleotide Excision Repair Protein
3551.	RBBP4	RB Binding Protein 4, Chromatin Remodeling Factor
3552.	RTN4	Reticulon 4
3553.	ULK1	Unc-51 Like Autophagy Activating Kinase 1
3554.	ADAMTS1	ADAM Metallopeptidase With Thrombospondin Type 1 Motif 1
3555.	ANP32A	Acidic Nuclear Phosphoprotein 32 Family Member A
3556.	CASP5	Caspase 5
3557.	CDK13	Cyclin Dependent Kinase 13
3558.	COL11A1	Collagen Type XI Alpha 1 Chain
3559.	DLG1	Discs Large MAGUK Scaffold Protein 1
3560.	EFNA1	Ephrin A1

3561.	FCN2	Ficolin 2
3562.	IL17RD	Interleukin 17 Receptor D
3563.	LARS1	Leucyl-TRNA Synthetase 1
3564.	LEFTY2	Left-Right Determination Factor 2
3565.	LTB4R	Leukotriene B4 Receptor
3566.	MBD4	Methyl-CpG Binding Domain 4, DNA Glycosylase
3567.	OCLN	Occludin
3568.	PHOX2A	Paired Like Homeobox 2A
3569.	PIK3R3	Phosphoinositide-3-Kinase Regulatory Subunit 3
3570.	PLXNA1	Plexin A1
3571.	POLK	DNA Polymerase Kappa
3572.	SLCO1B3	Solute Carrier Organic Anion Transporter Family Member 1B3
3573.	TAC3	Tachykinin Precursor 3
3574.	ADCY7	Adenylate Cyclase 7
3575.	AKAP9	A-Kinase Anchoring Protein 9
3576.	CD2AP	CD2 Associated Protein
3577.	CDT1	Chromatin Licensing And DNA Replication Factor 1
3578.	COPS5	COP9 Signalosome Subunit 5
3579.	DSG1	Desmoglein 1
3580.	E2F3	E2F Transcription Factor 3
3581.	FBLN2	Fibulin 2
3582.	GLP2R	Glucagon Like Peptide 2 Receptor
3583.	GPC1	Glypican 1
3584.	HDAC10	Histone Deacetylase 10
3585.	ITGA9	Integrin Subunit Alpha 9
3586.	LCT	Lactase
3587.	LYVE1	Lymphatic Vessel Endothelial Hyaluronan Receptor 1
3588.	MTA1	Metastasis Associated 1
3589.	NODAL	Nodal Growth Differentiation Factor
3590.	NPY5R	Neuropeptide Y Receptor Y5
3591.	PABPC1	Poly(A) Binding Protein Cytoplasmic 1
3592.	PAPPA	Pappalysin 1
3593.	PTGDR2	Prostaglandin D2 Receptor 2
3594.	RALB	RAS Like Proto-Oncogene B
3595.	RERE	Arginine-Glutamic Acid Dipeptide Repeats
3596.	SAE1	SUMO1 Activating Enzyme Subunit 1
3597.	SLC25A15	Solute Carrier Family 25 Member 15
3598.	STIP1	Stress Induced Phosphoprotein 1
3599.	TRPM4	Transient Receptor Potential Cation Channel Subfamily M Member 4
3600.	AOC1	Amine Oxidase Copper Containing 1
3601.	AP3B2	Adaptor Related Protein Complex 3 Subunit Beta 2

3602.	APOA4	Apolipoprotein A4
3603.	BLVRB	Biliverdin Reductase B
3604.	CCT7	Chaperonin Containing TCP1 Subunit 7
3605.	CLDN2	Claudin 2
3606.	GJC1	Gap Junction Protein Gamma 1
3607.	HS6ST1	Heparan Sulfate 6-O-Sulfotransferase 1
3608.	INF2	Inverted Formin 2
3609.	MNX1	Motor Neuron And Pancreas Homeobox 1
3610.	MOCOS	Molybdenum Cofactor Sulfurase
3611.	OPLAH	5-Oxoprolinase, ATP-Hydrolysing
3612.	PIGK	Phosphatidylinositol Glycan Anchor Biosynthesis Class K
3613.	PROK2	Prokineticin 2
3614.	RHBDF2	Rhomboid 5 Homolog 2
3615.	SECISBP2	SECIS Binding Protein 2
3616.	SEL1L	SEL1L Adaptor Subunit Of SYVN1 Ubiquitin Ligase
3617.	SH3KBP1	SH3 Domain Containing Kinase Binding Protein 1
3618.	SLC35A3	Solute Carrier Family 35 Member A3
3619.	SYN3	Synapsin III
3620.	ADCY4	Adenylate Cyclase 4
3621.	ARTN	Artemin
3622.	CLDN3	Claudin 3
3623.	CPB1	Carboxypeptidase B1
3624.	CTRL	Chymotrypsin Like
3625.	DSG3	Desmoglein 3
3626.	E2F2	E2F Transcription Factor 2
3627.	GATB	Glutamyl-TRNA Amidotransferase Subunit B
3628.	ICA1	Islet Cell Autoantigen 1
3629.	MAFA	MAF BZIP Transcription Factor A
3630.	MC3R	Melanocortin 3 Receptor
3631.	POLQ	DNA Polymerase Theta
3632.	PRX	Periaxin
3633.	SMPD3	Sphingomyelin Phosphodiesterase 3
3634.	SOX18	SRY-Box Transcription Factor 18
3635.	VTCN1	V-Set Domain Containing T Cell Activation Inhibitor 1
3636.	WDR45	WD Repeat Domain 45
3637.	A2ML1	Alpha-2-Macroglobulin Like 1
3638.	CAMTA1	Calmodulin Binding Transcription Activator 1
3639.	CCBE1	Collagen And Calcium Binding EGF Domains 1
3640.	CDK11B	Cyclin Dependent Kinase 11B
3641.	CHD5	Chromodomain Helicase DNA Binding Protein 5
3642.	CLMP	CXADR Like Membrane Protein

3643.	CPSF3	Cleavage And Polyadenylation Specific Factor 3
3644.	CYTH2	Cytohesin 2
3645.	DHRS3	Dehydrogenase/Reductase 3
3646.	DYNLT1	Dynein Light Chain Tctex-Type 1
3647.	ESPL1	Extra Spindle Pole Bodies Like 1, Separase
3648.	FFAR1	Free Fatty Acid Receptor 1
3649.	FIS1	Fission, Mitochondrial 1
3650.	GOLM1	Golgi Membrane Protein 1
3651.	HPR	Haptoglobin-Related Protein
3652.	HSD3B7	Hydroxy-Delta-5-Steroid Dehydrogenase, 3 Beta- And Steroid Delta-Isomerase 7
3653.	HUS1	HUS1 Checkpoint Clamp Component
3654.	KCNIP2	Potassium Voltage-Gated Channel Interacting Protein 2
3655.	KLF10	KLF Transcription Factor 10
3656.	MAPK8IP3	Mitogen-Activated Protein Kinase 8 Interacting Protein 3
3657.	MGAT4B	Alpha-1,3-Mannosyl-Glycoprotein 4-Beta-N-Acetylglucosaminyltransferase B
3658.	MSH4	MutS Homolog 4
3659.	PAPPA2	Pappalysin 2
3660.	PSIP1	PC4 And SRSF1 Interacting Protein 1
3661.	RFK	Riboflavin Kinase
3662.	RND3	Rho Family GTPase 3
3663.	RPA3	Replication Protein A3
3664.	RPS9	Ribosomal Protein S9
3665.	SEMA3B	Semaphorin 3B
3666.	SMUG1	Single-Strand-Selective Monofunctional Uracil-DNA Glycosylase 1
3667.	SPART	Spartin
3668.	STEAP1	STEAP Family Member 1
3669.	STMN2	Stathmin 2
3670.	SYNGR1	Synaptogyrin 1
3671.	UBE4B	Ubiquitination Factor E4B
3672.	ARHGAP32	Rho GTPase Activating Protein 32
3673.	CCL24	C-C Motif Chemokine Ligand 24
3674.	CDK11A	Cyclin Dependent Kinase 11A
3675.	CFC1	Cryptic, EGF-CFC Family Member 1
3676.	CNN1	Calponin 1
3677.	CPSF4	Cleavage And Polyadenylation Specific Factor 4
3678.	CXCR6	C-X-C Motif Chemokine Receptor 6
3679.	DEFB1	Defensin Beta 1
3680.	DNAJA2	DnaJ Heat Shock Protein Family (Hsp40) Member A2
3681.	E2F5	E2F Transcription Factor 5
3682.	EIF3M	Eukaryotic Translation Initiation Factor 3 Subunit M
3683.	FUCA2	Alpha-L-Fucosidase 2

3684.	HEY2	Hes Related Family BHLH Transcription Factor With YRPW Motif 2
3685.	HLX	H2.0 Like Homeobox
3686.	HRK	Harakiri, BCL2 Interacting Protein
3687.	KPNA4	Karyopherin Subunit Alpha 4
3688.	LGI2	Leucine Rich Repeat LGI Family Member 2
3689.	LYST	Lysosomal Trafficking Regulator
3690.	MCM9	Minichromosome Maintenance 9 Homologous Recombination Repair Factor
3691.	MGAT4A	Alpha-1,3-Mannosyl-Glycoprotein 4-Beta-N-Acetylglucosaminyltransferase A
3692.	NAP1L4	Nucleosome Assembly Protein 1 Like 4
3693.	NHLH2	Nescient Helix-Loop-Helix 2
3694.	PIP	Prolactin Induced Protein
3695.	PPL	Periplakin
3696.	RPL27A	Ribosomal Protein L27a
3697.	RPS5	Ribosomal Protein S5
3698.	SBF2	SET Binding Factor 2
3699.	SENP8	SUMO Peptidase Family Member, NEDD8 Specific
3700.	STOX1	Storkhead Box 1
3701.	THSD4	Thrombospondin Type 1 Domain Containing 4
3702.	TMPRSS11D	Transmembrane Serine Protease 11D
3703.	UBE2S	Ubiquitin Conjugating Enzyme E2 S
3704.	XPO5	Exportin 5
3705.	ZC3H12A	Zinc Finger CCCH-Type Containing 12A
3706.	ABRAXAS1	Abraxas 1, BRCA1 A Complex Subunit
3707.	ANP32B	Acidic Nuclear Phosphoprotein 32 Family Member B
3708.	AP2A1	Adaptor Related Protein Complex 2 Subunit Alpha 1
3709.	APOL2	Apolipoprotein L2
3710.	ARID3B	AT-Rich Interaction Domain 3B
3711.	ARL6IP1	ADP Ribosylation Factor Like GTPase 6 Interacting Protein 1
3712.	ATP11C	ATPase Phospholipid Transporting 11C
3713.	ENC1	Ectodermal-Neural Cortex 1
3714.	EPB41L2	Erythrocyte Membrane Protein Band 4.1 Like 2
3715.	EVI5	Ecotropic Viral Integration Site 5
3716.	FBN3	Fibrillin 3
3717.	GEN1	GEN1 Holliday Junction 5' Flap Endonuclease
3718.	GH2	Growth Hormone 2
3719.	HOXB2	Homeobox B2
3720.	HYCC1	Hyccin PI4KA Lipid Kinase Complex Subunit 1
3721.	KPNA6	Karyopherin Subunit Alpha 6
3722.	LYAR	Ly1 Antibody Reactive
3723.	MAEA	Macrophage Erythroblast Attacher, E3 Ubiquitin Ligase
3724.	NBL1	NBL1, DAN Family BMP Antagonist

3725.	NEIL1	Nei Like DNA Glycosylase 1
3726.	NUDT6	Nudix Hydrolase 6
3727.	OLFM1	Olfactomedin 1
3728.	PENK	Proenkephalin
3729.	RBFOX3	RNA Binding Fox-1 Homolog 3
3730.	SENP6	SUMO Specific Peptidase 6
3731.	SLCO1A2	Solute Carrier Organic Anion Transporter Family Member 1A2
3732.	SPARCL1	SPARC Like 1
3733.	TBC1D5	TBC1 Domain Family Member 5
3734.	TMEM98	Transmembrane Protein 98
3735.	ZNF143	Zinc Finger Protein 143
3736.	AATK	Apoptosis Associated Tyrosine Kinase
3737.	AHNAK	AHNAK Nucleoprotein
3738.	BRK1	BRICK1 Subunit Of SCAR/WAVE Actin Nucleating Complex
3739.	CCL25	C-C Motif Chemokine Ligand 25
3740.	COX17	Cytochrome C Oxidase Copper Chaperone COX17
3741.	CSH1	Chorionic Somatomammotropin Hormone 1
3742.	FAM3C	FAM3 Metabolism Regulating Signaling Molecule C
3743.	FCHO2	FCH And Mu Domain Containing Endocytic Adaptor 2
3744.	KLHL12	Kelch Like Family Member 12
3745.	LACC1	Laccase Domain Containing 1
3746.	LGALS13	Galectin 13
3747.	MAFK	MAF BZIP Transcription Factor K
3748.	NAP1L1	Nucleosome Assembly Protein 1 Like 1
3749.	NMT2	N-Myristoyltransferase 2
3750.	OGFR	Opioid Growth Factor Receptor
3751.	PRUNE2	Prune Homolog 2 With BCH Domain
3752.	QSOX2	Quiescin Sulfhydryl Oxidase 2
3753.	RAB33A	RAB33A, Member RAS Oncogene Family
3754.	RASA3	RAS P21 Protein Activator 3
3755.	SLC31A2	Solute Carrier Family 31 Member 2
3756.	TENT4A	Terminal Nucleotidyltransferase 4A
3757.	ZDHHC19	Zinc Finger DHHC-Type Palmitoyltransferase 19
3758.	ADGRB1	Adhesion G Protein-Coupled Receptor B1
3759.	ANKMY1	Ankyrin Repeat And MYND Domain Containing 1
3760.	ANKRD49	Ankyrin Repeat Domain 49
3761.	AP2A2	Adaptor Related Protein Complex 2 Subunit Alpha 2
3762.	APOL4	Apolipoprotein L4
3763.	ASIC5	Acid Sensing Ion Channel Subunit Family Member 5
3764.	ATAD5	ATPase Family AAA Domain Containing 5
3765.	DENND1B	DENN Domain Containing 1B

3766.	FAM107A	Family With Sequence Similarity 107 Member A
3767.	G3BP2	G3BP Stress Granule Assembly Factor 2
3768.	HES7	Hes Family BHLH Transcription Factor 7
3769.	KCTD12	Potassium Channel Tetramerization Domain Containing 12
3770.	LAX1	Lymphocyte Transmembrane Adaptor 1
3771.	MESP2	Mesoderm Posterior BHLH Transcription Factor 2
3772.	PCDH18	Protocadherin 18
3773.	PROK1	Prokineticin 1
3774.	RAB24	RAB24, Member RAS Oncogene Family
3775.	RAB26	RAB26, Member RAS Oncogene Family
3776.	STX17	Syntaxin 17
3777.	TMOD4	Tropomodulin 4
3778.	TMSB10	Thymosin Beta 10
3779.	ZDHHC2	Zinc Finger DHHC-Type Palmitoyltransferase 2
3780.	ZG16B	Zymogen Granule Protein 16B
3781.	BAHD1	Bromo Adjacent Homology Domain Containing 1
3782.	COX18	Cytochrome C Oxidase Assembly Factor COX18
3783.	EMP1	Epithelial Membrane Protein 1
3784.	FAM107B	Family With Sequence Similarity 107 Member B
3785.	GCC1	GRIP And Coiled-Coil Domain Containing 1
3786.	KCNE5	Potassium Voltage-Gated Channel Subfamily E Regulatory Subunit 5
3787.	NPAT	Nuclear Protein, Coactivator Of Histone Transcription
3788.	PI15	Peptidase Inhibitor 15
3789.	PRIMPOL	Primase And DNA Directed Polymerase
3790.	RTF1	RTF1 Homolog, Paf1/RNA Polymerase II Complex Component
3791.	SNAPC3	Small Nuclear RNA Activating Complex Polypeptide 3
3792.	SPRTN	SprT-Like N-Terminal Domain
3793.	SYNPO2L	Synaptopodin 2 Like
3794.	TECPR2	Tectonin Beta-Propeller Repeat Containing 2
3795.	VPS72	Vacuolar Protein Sorting 72 Homolog
3796.	ZBTB48	Zinc Finger And BTB Domain Containing 48
3797.	ALX3	ALX Homeobox 3
3798.	DUSP26	Dual Specificity Phosphatase 26
3799.	GBP4	Guanylate Binding Protein 4
3800.	INSM2	INSM Transcriptional Repressor 2
3801.	JAGN1	Jagunal Homolog 1
3802.	MXRA8	Matrix Remodeling Associated 8
3803.	APLF	Aprataxin And PNKP Like Factor
3804.	ARMC12	Armadillo Repeat Containing 12
3805.	DAW1	Dynein Assembly Factor With WD Repeats 1
3806.	FAM163A	Family With Sequence Similarity 163 Member A

3807.	LRRC17	Leucine Rich Repeat Containing 17
3808.	NBPF3	NBPF Member 3
3809.	SCAI	Suppressor Of Cancer Cell Invasion
3810.	ZNF2	Zinc Finger Protein 2
3811.	C5orf15	Chromosome 5 Open Reading Frame 15
3812.	CAMTA2	Calmodulin Binding Transcription Activator 2
3813.	DALRD3	DALR Anticodon Binding Domain Containing 3
3814.	NHLH1	Nescient Helix-Loop-Helix 1
3815.	RBP5	Retinol Binding Protein 5
3816.	SPIN4	Spindlin Family Member 4
3817.	CBARP	CACN Subunit Beta Associated Regulatory Protein
3818.	PRAF2	PRA1 Domain Family Member 2
3819.	RBMY1A1	RNA Binding Motif Protein Y-Linked Family 1 Member A1
3820.	SLC45A1	Solute Carrier Family 45 Member 1
3821.	ZNF546	Zinc Finger Protein 546
3822.	DENND11	DENN Domain Containing 11
3823.	TOPAZ1	Testis And Ovary Specific TOPAZ 1
3824.	CDRT4	CMT1A Duplicated Region Transcript 4
3825.	CNBD2	Cyclic Nucleotide Binding Domain Containing 2
3826.	LRRC63	Leucine Rich Repeat Containing 63
3827.	NBPF1	NBPF Member 1
3828.	NBPF15	NBPF Member 15
3829.	NBPF6	NBPF Member 6
3830.	OR51I1	Olfactory Receptor Family 51 Subfamily I Member 1
3831.	RAD21L1	RAD21 Cohesin Complex Component Like 1
3832.	SPIN3	Spindlin Family Member 3
3833.	CFAP73	Cilia And Flagella Associated Protein 73
3834.	TEX38	Testis Expressed 38
3835.	TMSB15A	Thymosin Beta 15A
3836.	NBPF12	NBPF Member 12
3837.	GOLGA6L2	Golgin A6 Family Like 2
3838.	NBPF10	NBPF Member 10
3839.	NBPF4	NBPF Member 4
3840.	FREY1	Frey Regulator Of Sperm-Oocyte Fusion 1
3841.	NBPF14	NBPF Member 14
3842.	MEIKIN	Meiotic Kinetochore Factor
3843.	NBPF9	NBPF Member 9
3844.	NBPF8	NBPF Member 8
3845.	NBPF20	NBPF Member 20
3846.	TDRD15	Tudor Domain Containing 15
3847.	RIMBP3B	RIMS Binding Protein 3B

3848.	FPGT-TNNI3K	FPGT-TNNI3K Readthrough
3849.	MICOS10-NBL1	MICOS10-NBL1 Readthrough
3850.	NBPF26	NBPF Member 26
3851.	NBPF19	NBPF Member 19
3852.	PMIS2	PMIS2 Transmembrane Protein
3853.	LMCD1	LIM And Cysteine Rich Domains 1
3854.	AQP1	Aquaporin 1 (Colton Blood Group)
3855.	PI4KA	Phosphatidylinositol 4-Kinase Alpha
3856.	CDH11	Cadherin 11
3857.	DHODH	Dihydroorotate Dehydrogenase (Quinone)
3858.	MMP19	Matrix Metallopeptidase 19
3859.	PDE6B	Phosphodiesterase 6B
3860.	PPP1CA	Protein Phosphatase 1 Catalytic Subunit Alpha
3861.	TAF1	TATA-Box Binding Protein Associated Factor 1
3862.	TRPC3	Transient Receptor Potential Cation Channel Subfamily C Member 3
3863.	CD81	CD81 Molecule
3864.	COL4A3	Collagen Type IV Alpha 3 Chain
3865.	IKBKE	Inhibitor Of Nuclear Factor Kappa B Kinase Subunit Epsilon
3866.	C1QC	Complement C1q C Chain
3867.	SELPLG	Selectin P Ligand
3868.	TPK1	Thiamin Pyrophosphokinase 1
3869.	DARS2	Aspartyl-TRNA Synthetase 2, Mitochondrial
3870.	MAFB	MAF BZIP Transcription Factor B
3871.	PFKL	Phosphofructokinase, Liver Type
3872.	BCKDHA	Branched Chain Keto Acid Dehydrogenase E1 Subunit Alpha
3873.	IRF2	Interferon Regulatory Factor 2
3874.	COCH	Cochlin
3875.	CXCL11	C-X-C Motif Chemokine Ligand 11
3876.	FKBP8	FKBP Prolyl Isomerase 8
3877.	OSTF1	Osteoclast Stimulating Factor 1
3878.	STEAP4	STEAP4 Metalloreductase
3879.	ASIC3	Acid Sensing Ion Channel Subunit 3
3880.	PRSS2	Serine Protease 2
3881.	BTLA	B And T Lymphocyte Associated
3882.	CCL19	C-C Motif Chemokine Ligand 19
3883.	MPP1	MAGUK P55 Scaffold Protein 1
3884.	PDE7A	Phosphodiesterase 7A
3885.	PEG10	Paternally Expressed 10
3886.	PRDX4	Peroxiredoxin 4
3887.	SPAG1	Sperm Associated Antigen 1
3888.	CARD8	Caspase Recruitment Domain Family Member 8

3889.	ERO1B	Endoplasmic Reticulum Oxidoreductase 1 Beta
3890.	FUT7	Fucosyltransferase 7
3891.	TRAPPC2	Trafficking Protein Particle Complex Subunit 2
3892.	CLDN6	Claudin 6
3893.	HM13	Histocompatibility Minor 13
3894.	LIPN	Lipase Family Member N
3895.	RGN	Regucalcin
3896.	SLC23A1	Solute Carrier Family 23 Member 1
3897.	SLC2A8	Solute Carrier Family 2 Member 8
3898.	TAGAP	T Cell Activation RhoGTPase Activating Protein
3899.	CLDN9	Claudin 9
3900.	EEFSEC	Eukaryotic Elongation Factor, Selenocysteine-TRNA Specific
3901.	LAPTM4A	Lysosomal Protein Transmembrane 4 Alpha
3902.	PLB1	Phospholipase B1
3903.	RHOF	Ras Homolog Family Member F, Filopodia Associated
3904.	MIOX	Myo-Inositol Oxygenase
3905.	MPP2	MAGUK P55 Scaffold Protein 2
3906.	MTFR1	Mitochondrial Fission Regulator 1
3907.	TRIM14	Tripartite Motif Containing 14
3908.	MRGPRX2	MAS Related GPR Family Member X2
3909.	SLC23A3	Solute Carrier Family 23 Member 3
3910.	TECTB	Tectorin Beta
3911.	GDPGP1	GDP-D-Glucose Phosphorylase 1
3912.	IFNL4	Interferon Lambda 4 (Gene/Pseudogene)
3913.	LIPE	Lipase E, Hormone Sensitive Type
3914.	ACTG2	Actin Gamma 2, Smooth Muscle
3915.	UTS2R	Urotensin 2 Receptor
3916.	HLA-DRB3	Major Histocompatibility Complex, Class II, DR Beta 3
3917.	NSF	N-Ethylmaleimide Sensitive Factor, Vesicle Fusing ATPase
3918.	VAMP2	Vesicle Associated Membrane Protein 2
3919.	GTF2I	General Transcription Factor IIi
3920.	CASP6	Caspase 6
3921.	GRM4	Glutamate Metabotropic Receptor 4
3922.	NNMT	Nicotinamide N-Methyltransferase
3923.	RAD21	RAD21 Cohesin Complex Component
3924.	BRD2	Bromodomain Containing 2
3925.	HRH3	Histamine Receptor H3
3926.	PDCD1LG2	Programmed Cell Death 1 Ligand 2
3927.	LMO2	LIM Domain Only 2
3928.	STX1B	Syntaxin 1B
3929.	CBFA2T3	CBFA2/RUNX1 Partner Transcriptional Co-Repressor 3

3930.	GFI1B	Growth Factor Independent 1B Transcriptional Repressor
3931.	ASXL2	ASXL Transcriptional Regulator 2
3932.	KCNS2	Potassium Voltage-Gated Channel Modifier Subfamily S Member 2
3933.	ATP2A2	ATPase Sarcoplasmic/Endoplasmic Reticulum Ca2+ Transporting 2
3934.	CACNA1B	Calcium Voltage-Gated Channel Subunit Alpha1 B
3935.	SLC6A1	Solute Carrier Family 6 Member 1
3936.	CCP110	Centriolar Coiled-Coil Protein 110
3937.	MYH1	Myosin Heavy Chain 1
3938.	SLN	Sarcolipin
3939.	FUCA1	Alpha-L-Fucosidase 1
3940.	ARSB	Arylsulfatase B
3941.	DOCK8	Dedicator Of Cytokinesis 8
3942.	M6PR	Mannose-6-Phosphate Receptor, Cation Dependent
3943.	SLC25A6	Solute Carrier Family 25 Member 6
3944.	LIMS2	LIM Zinc Finger Domain Containing 2
3945.	HSPB2	Heat Shock Protein Family B (Small) Member 2
3946.	OGA	O-GlcNAcase
3947.	KCNE4	Potassium Voltage-Gated Channel Subfamily E Regulatory Subunit 4
3948.	SFXN2	Sideroflexin 2
3949.	SYNM	Synemin
3950.	ABCA7	ATP Binding Cassette Subfamily A Member 7
3951.	AFG3L2	AFG3 Like Matrix AAA Peptidase Subunit 2
3952.	SARS2	Seryl-TRNA Synthetase 2, Mitochondrial
3953.	SUCLG1	Succinate-CoA Ligase GDP/ADP-Forming Subunit Alpha
3954.	GGPS1	Geranylgeranyl Diphosphate Synthase 1
3955.	PNPT1	Polyribonucleotide Nucleotidyltransferase 1
3956.	CAVIN1	Caveolae Associated Protein 1
3957.	GOSR2	Golgi SNAP Receptor Complex Member 2
3958.	SLC5A6	Solute Carrier Family 5 Member 6
3959.	CARS2	Cysteinyl-TRNA Synthetase 2, Mitochondrial
3960.	DHX16	DEAH-Box Helicase 16
3961.	NDUFA4	NDUFA4 Mitochondrial Complex Associated
3962.	TRIP4	Thyroid Hormone Receptor Interactor 4
3963.	AGTPBP1	ATP/GTP Binding Carboxypeptidase 1
3964.	GOLGA2	Golgin A2
3965.	MSTO1	Misato Mitochondrial Distribution And Morphology Regulator 1
3966.	POPDC3	Popeye Domain Containing 3
3967.	MIEF2	Mitochondrial Elongation Factor 2
3968.	RILPL1	Rab Interacting Lysosomal Protein Like 1
3969.	TNNC2	Troponin C2, Fast Skeletal Type
3970.	KY	Kyphoscoliosis Peptidase

3971.	PLIN4	Perilipin 4
3972.	VMA21	Vacuolar ATPase Assembly Factor VMA21
3973.	CCDC174	Coiled-Coil Domain Containing 174
3974.	CYP2C8	Cytochrome P450 Family 2 Subfamily C Member 8
3975.	PPARD	Peroxisome Proliferator Activated Receptor Delta
3976.	TFAP2A	Transcription Factor AP-2 Alpha
3977.	TOM1	Target Of Myb1 Membrane Trafficking Protein
3978.	ELP1	Elongator Acetyltransferase Complex Subunit 1
3979.	TKFC	Triokinase And FMN Cyclase
3980.	TUBG1	Tubulin Gamma 1
3981.	CHRM2	Cholinergic Receptor Muscarinic 2
3982.	HSPA9	Heat Shock Protein Family A (Hsp70) Member 9
3983.	EPHA1	EPH Receptor A1
3984.	EPHA3	EPH Receptor A3
3985.	SMC3	Structural Maintenance Of Chromosomes 3
3986.	TUBB2A	Tubulin Beta 2A Class IIa
3987.	APOC3	Apolipoprotein C3
3988.	NQO2	N-Ribosyldihydronicotinamide:Quinone Dehydrogenase 2
3989.	SLC19A2	Solute Carrier Family 19 Member 2
3990.	TRPM8	Transient Receptor Potential Cation Channel Subfamily M Member 8
3991.	BRDT	Bromodomain Testis Associated
3992.	CUL1	Cullin 1
3993.	FABP1	Fatty Acid Binding Protein 1
3994.	PSMD2	Proteasome 26S Subunit Ubiquitin Receptor, Non-ATPase 2
3995.	IGBP1	Immunoglobulin Binding Protein 1
3996.	PSMA1	Proteasome 20S Subunit Alpha 1
3997.	CCNE2	Cyclin E2
3998.	CD226	CD226 Molecule
3999.	METTL3	Methyltransferase 3, N6-Adenosine-Methyltransferase Complex Catalytic Subunit
4000.	GLIS2	GLIS Family Zinc Finger 2
4001.	MANF	Mesencephalic Astrocyte Derived Neurotrophic Factor
4002.	NXF1	Nuclear RNA Export Factor 1
4003.	ELL	Elongation Factor For RNA Polymerase II
4004.	METTL14	Methyltransferase 14, N6-Adenosine-Methyltransferase Subunit
4005.	YTHDC1	YTH N6-Methyladenosine RNA Binding Protein C1
4006.	YTHDF2	YTH N6-Methyladenosine RNA Binding Protein F2
4007.	CD24	CD24 Molecule
4008.	CNTLN	Centlein
4009.	TCHP	Trichoplein Keratin Filament Binding
4010.	YTHDF1	YTH N6-Methyladenosine RNA Binding Protein F1
4011.	USP46	Ubiquitin Specific Peptidase 46

4012.	HAPLN4	Hyaluronan And Proteoglycan Link Protein 4
4013.	MT-ND4L	Mitochondrially Encoded NADH:Ubiquinone Oxidoreductase Core Subunit 4L
4014.	RAB7A	RAB7A, Member RAS Oncogene Family
4015.	DPYSL2	Dihydropyrimidinase Like 2
4016.	PEPD	Peptidase D
4017.	FGD1	FYVE, RhoGEF And PH Domain Containing 1
4018.	ADSS2	Adenylosuccinate Synthase 2
4019.	MCM3AP	Minichromosome Maintenance Complex Component 3 Associated Protein
4020.	ATP1B3	ATPase Na+/K+ Transporting Subunit Beta 3
4021.	TRBV11-2	T Cell Receptor Beta Variable 11-2
4022.	EPHB4	EPH Receptor B4
4023.	GSN	Gelsolin
4024.	P4HB	Prolyl 4-Hydroxylase Subunit Beta
4025.	SYT1	Synaptotagmin 1
4026.	STX1A	Syntaxin 1A
4027.	HADHB	Hydroxyacyl-CoA Dehydrogenase Trifunctional Multienzyme Complex Subunit Beta
4028.	AOX1	Aldehyde Oxidase 1
4029.	COL17A1	Collagen Type XVII Alpha 1 Chain
4030.	DCT	Dopachrome Tautomerase
4031.	GRHPR	Glyoxylate And Hydroxypyruvate Reductase
4032.	PGAM1	Phosphoglycerate Mutase 1
4033.	RB1CC1	RB1 Inducible Coiled-Coil 1
4034.	GAB1	GRB2 Associated Binding Protein 1
4035.	MOCS2	Molybdenum Cofactor Synthesis 2
4036.	PEX19	Peroxisomal Biogenesis Factor 19
4037.	TXK	TXK Tyrosine Kinase
4038.	ARHGEF10	Rho Guanine Nucleotide Exchange Factor 10
4039.	DNM3	Dynamin 3
4040.	DST	Dystonin
4041.	FGF18	Fibroblast Growth Factor 18
4042.	MXI1	MAX Interactor 1, Dimerization Protein
4043.	SERPINE2	Serpin Family E Member 2
4044.	SLC7A9	Solute Carrier Family 7 Member 9
4045.	VASP	Vasodilator Stimulated Phosphoprotein
4046.	ADAMTS2	ADAM Metallopeptidase With Thrombospondin Type 1 Motif 2
4047.	HNRNPH1	Heterogeneous Nuclear Ribonucleoprotein H1
4048.	OLIG2	Oligodendrocyte Transcription Factor 2
4049.	PTPRU	Protein Tyrosine Phosphatase Receptor Type U
4050.	UNC13B	Unc-13 Homolog B
4051.	WASL	WASP Like Actin Nucleation Promoting Factor
4052.	SRSF6	Serine And Arginine Rich Splicing Factor 6

4053.	STX5	Syntaxin 5
4054.	NLRP7	NLR Family Pyrin Domain Containing 7
4055.	OTULIN	OTU Deubiquitinase With Linear Linkage Specificity
4056.	P3H2	Prolyl 3-Hydroxylase 2
4057.	REEP5	Receptor Accessory Protein 5
4058.	VPS45	Vacuolar Protein Sorting 45 Homolog
4059.	RAB1B	RAB1B, Member RAS Oncogene Family
4060.	RNF43	Ring Finger Protein 43
4061.	SSTR4	Somatostatin Receptor 4
4062.	COQ3	Coenzyme Q3, Methyltransferase
4063.	MBNL2	Muscleblind Like Splicing Regulator 2
4064.	NEIL2	Nei Like DNA Glycosylase 2
4065.	TGOLN2	Trans-Golgi Network Protein 2
4066.	TSPAN31	Tetraspanin 31
4067.	ARSD	Arylsulfatase D
4068.	RHOD	Ras Homolog Family Member D
4069.	RMDN3	Regulator Of Microtubule Dynamics 3
4070.	TAX1BP3	Tax1 Binding Protein 3
4071.	RRAGB	Ras Related GTP Binding B
4072.	SEC22B	SEC22 Homolog B, Vesicle Trafficking Protein
4073.	ZNF197	Zinc Finger Protein 197
4074.	AKR1B1	Aldo-Keto Reductase Family 1 Member B
4075.	CYP4F2	Cytochrome P450 Family 4 Subfamily F Member 2
4076.	GPX1	Glutathione Peroxidase 1
4077.	FKBP1B	FKBP Prolyl Isomerase 1B
4078.	FNDC5	Fibronectin Type III Domain Containing 5
4079.	MTERF1	Mitochondrial Transcription Termination Factor 1
4080.	LOC112694756	Uncharaterized LOC112694756
4081.	RAC2	Rac Family Small GTPase 2
4082.	PTPRJ	Protein Tyrosine Phosphatase Receptor Type J
4083.	CTSS	Cathepsin S
4084.	IL17RA	Interleukin 17 Receptor A
4085.	ATIC	5-Aminoimidazole-4-Carboxamide Ribonucleotide Formyltransferase/IMP Cyclohydrolase
4086.	AVPR1A	Arginine Vasopressin Receptor 1A
4087.	ENO3	Enolase 3
4088.	FPR2	Formyl Peptide Receptor 2
4089.	HNMT	Histamine N-Methyltransferase
4090.	KCNC3	Potassium Voltage-Gated Channel Subfamily C Member 3
4091.	POLA1	DNA Polymerase Alpha 1, Catalytic Subunit
4092.	PPP1CB	Protein Phosphatase 1 Catalytic Subunit Beta
4093.	PPP1CC	Protein Phosphatase 1 Catalytic Subunit Gamma

4094.	RELB	RELB Proto-Oncogene, NF-KB Subunit
4095.	TIE1	Tyrosine Kinase With Immunoglobulin Like And EGF Like Domains 1
4096.	FOXP2	Forkhead Box P2
4097.	LTA4H	Leukotriene A4 Hydrolase
4098.	PFKP	Phosphofructokinase, Platelet
4099.	CBLB	Cbl Proto-Oncogene B
4100.	CCL7	C-C Motif Chemokine Ligand 7
4101.	FOLR2	Folate Receptor Beta
4102.	JAM3	Junctional Adhesion Molecule 3
4103.	MYBPC1	Myosin Binding Protein C1
4104.	PLP1	Proteolipid Protein 1
4105.	SPRED1	Sprouty Related EVH1 Domain Containing 1
4106.	HIP1	Huntingtin Interacting Protein 1
4107.	HRH4	Histamine Receptor H4
4108.	HSPE1	Heat Shock Protein Family E (Hsp10) Member 1
4109.	MATN3	Matrilin 3
4110.	PMPCA	Peptidase, Mitochondrial Processing Subunit Alpha
4111.	TPR	Translocated Promoter Region, Nuclear Basket Protein
4112.	CILP	Cartilage Intermediate Layer Protein
4113.	FSTL1	Follistatin Like 1
4114.	HAPLN1	Hyaluronan And Proteoglycan Link Protein 1
4115.	HIBCH	3-Hydroxyisobutyryl-CoA Hydrolase
4116.	PRKRA	Protein Activator Of Interferon Induced Protein Kinase EIF2AK2
4117.	SYVN1	Synoviolin 1
4118.	TRPC1	Transient Receptor Potential Cation Channel Subfamily C Member 1
4119.	IL17B	Interleukin 17B
4120.	LTBR	Lymphotoxin Beta Receptor
4121.	TNFAIP6	TNF Alpha Induced Protein 6
4122.	IL22RA1	Interleukin 22 Receptor Subunit Alpha 1
4123.	NPSR1	Neuropeptide S Receptor 1
4124.	PSMC6	Proteasome 26S Subunit, ATPase 6
4125.	TNFSF14	TNF Superfamily Member 14
4126.	FEZF1	FEZ Family Zinc Finger 1
4127.	FHL3	Four And A Half LIM Domains 3
4128.	GYG2	Glycogenin 2
4129.	PHF19	PHD Finger Protein 19
4130.	PPP1R15A	Protein Phosphatase 1 Regulatory Subunit 15A
4131.	RAB5C	RAB5C, Member RAS Oncogene Family
4132.	SIAE	Sialic Acid Acetylesterase
4133.	THAP1	THAP Domain Containing 1
4134.	EMSY	EMSY Transcriptional Repressor, BRCA2 Interacting

4135.	NSUN5	NOP2/Sun RNA Methyltransferase 5
4136.	ORAI2	ORAI Calcium Release-Activated Calcium Modulator 2
4137.	ZFP36	ZFP36 Ring Finger Protein
4138.	CES3	Carboxylesterase 3
4139.	ASZ1	Ankyrin Repeat, SAM And Basic Leucine Zipper Domain Containing 1
4140.	ESYT1	Extended Synaptotagmin 1
4141.	GOLGB1	Golgin B1
4142.	IRGM	Immunity Related GTPase M
4143.	MTCH1	Mitochondrial Carrier 1
4144.	NAT9	N-Acetyltransferase 9 (Putative)
4145.	OLAH	Oleoyl-ACP Hydrolase
4146.	PDZD11	PDZ Domain Containing 11
4147.	PGP	Phosphoglycolate Phosphatase
4148.	SLFN14	Schlafen Family Member 14
4149.	ZNF334	Zinc Finger Protein 334
4150.	FEZF2	FEZ Family Zinc Finger 2
4151.	ORAI3	ORAI Calcium Release-Activated Calcium Modulator 3
4152.	SARAF	Store-Operated Calcium Entry Associated Regulatory Factor
4153.	CRACR2A	Calcium Release Activated Channel Regulator 2A
4154.	CENPV	Centromere Protein V
4155.	MACIR	Macrophage Immunometabolism Regulator
4156.	PDF	Peptide Deformylase, Mitochondrial
4157.	TMEM134	Transmembrane Protein 134
4158.	CST11	Cystatin 11
4159.	STIMATE	STIM Activating Enhancer
4160.	GPR33	G Protein-Coupled Receptor 33
4161.	GGTLC3	Gamma-Glutamyltransferase Light Chain Family Member 3
4162.	EDNRB	Endothelin Receptor Type B
4163.	STXBP1	Syntaxin Binding Protein 1
4164.	SLC12A5	Solute Carrier Family 12 Member 5
4165.	ALDH5A1	Aldehyde Dehydrogenase 5 Family Member A1
4166.	KAT2A	Lysine Acetyltransferase 2A
4167.	KAT2B	Lysine Acetyltransferase 2B
4168.	PPM1D	Protein Phosphatase, Mg2+/Mn2+ Dependent 1D
4169.	BRD4	Bromodomain Containing 4
4170.	CACNA1E	Calcium Voltage-Gated Channel Subunit Alpha1 E
4171.	DGAT1	Diacylglycerol O-Acyltransferase 1
4172.	MAPK12	Mitogen-Activated Protein Kinase 12
4173.	MAPK13	Mitogen-Activated Protein Kinase 13
4174.	MTHFD1	Methylenetetrahydrofolate Dehydrogenase, Cyclohydrolase And Formyltetrahydrofolate Synthetase 1
4175.	DSC2	Desmocollin 2

4176.	GRIK1	Glutamate Ionotropic Receptor Kainate Type Subunit 1
4177.	GRM2	Glutamate Metabotropic Receptor 2
4178.	MAPK11	Mitogen-Activated Protein Kinase 11
4179.	PCK2	Phosphoenolpyruvate Carboxykinase 2, Mitochondrial
4180.	PSMA3	Proteasome 20S Subunit Alpha 3
4181.	PSMA6	Proteasome 20S Subunit Alpha 6
4182.	PSMB1	Proteasome 20S Subunit Beta 1
4183.	SIAH1	Siah E3 Ubiquitin Protein Ligase 1
4184.	EPRS1	Glutamyl-Prolyl-TRNA Synthetase 1
4185.	FER	FER Tyrosine Kinase
4186.	GSTO1	Glutathione S-Transferase Omega 1
4187.	LMX1B	LIM Homeobox Transcription Factor 1 Beta
4188.	MTTP	Microsomal Triglyceride Transfer Protein
4189.	RELN	Reelin
4190.	SNCB	Synuclein Beta
4191.	TOP2B	DNA Topoisomerase II Beta
4192.	UBB	Ubiquitin B
4193.	UQCRC2	Ubiquinol-Cytochrome C Reductase Core Protein 2
4194.	AGO2	Argonaute RISC Catalytic Component 2
4195.	AMPH	Amphiphysin
4196.	CD151	CD151 Molecule (Raph Blood Group)
4197.	GAK	Cyclin G Associated Kinase
4198.	PSMA4	Proteasome 20S Subunit Alpha 4
4199.	PSMA5	Proteasome 20S Subunit Alpha 5
4200.	PSMB5	Proteasome 20S Subunit Beta 5
4201.	PTPA	Protein Phosphatase 2 Phosphatase Activator
4202.	SNCAIP	Synuclein Alpha Interacting Protein
4203.	CAV2	Caveolin 2
4204.	GPR37	G Protein-Coupled Receptor 37
4205.	KDM5A	Lysine Demethylase 5A
4206.	NUDT1	Nudix Hydrolase 1
4207.	PSMB2	Proteasome 20S Subunit Beta 2
4208.	PSMC1	Proteasome 26S Subunit, ATPase 1
4209.	PSMC5	Proteasome 26S Subunit, ATPase 5
4210.	RPGR	Retinitis Pigmentosa GTPase Regulator
4211.	SNCG	Synuclein Gamma
4212.	SUZ12	SUZ12 Polycomb Repressive Complex 2 Subunit
4213.	ADGRV1	Adhesion G Protein-Coupled Receptor V1
4214.	AFDN	Afadin, Adherens Junction Formation Factor
4215.	CD96	CD96 Molecule
4216.	CHM	CHM Rab Escort Protein

4217.	DSC3	Desmocollin 3
4218.	GIGYF2	GRB10 Interacting GYF Protein 2
4219.	LMX1A	LIM Homeobox Transcription Factor 1 Alpha
4220.	LPA	Lipoprotein(A)
4221.	PSMB6	Proteasome 20S Subunit Beta 6
4222.	PSMD1	Proteasome 26S Subunit, Non-ATPase 1
4223.	RAB10	RAB10, Member RAS Oncogene Family
4224.	UBE2G2	Ubiquitin Conjugating Enzyme E2 G2
4225.	UBE2J1	Ubiquitin Conjugating Enzyme E2 J1
4226.	DGKQ	Diacylglycerol Kinase Theta
4227.	GSTA4	Glutathione S-Transferase Alpha 4
4228.	HOXA5	Homeobox A5
4229.	MN1	MN1 Proto-Oncogene, Transcriptional Regulator
4230.	PACRG	Parkin Coregulated
4231.	PARL	Presenilin Associated Rhomboid Like
4232.	PSMB3	Proteasome 20S Subunit Beta 3
4233.	PSMD6	Proteasome 26S Subunit, Non-ATPase 6
4234.	UBE2E3	Ubiquitin Conjugating Enzyme E2 E3
4235.	VPS29	VPS29 Retromer Complex Component
4236.	VPS41	VPS41 Subunit Of HOPS Complex
4237.	BBS1	Bardet-Biedl Syndrome 1
4238.	COLEC10	Collectin Subfamily Member 10
4239.	GSTO2	Glutathione S-Transferase Omega 2
4240.	LRRK1	Leucine Rich Repeat Kinase 1
4241.	NEDD8	NEDD8 Ubiquitin Like Modifier
4242.	SEPTIN4	Septin 4
4243.	SYT11	Synaptotagmin 11
4244.	TUBGCP2	Tubulin Gamma Complex Component 2
4245.	UBE2E2	Ubiquitin Conjugating Enzyme E2 E2
4246.	VPS26A	VPS26 Retromer Complex Component A
4247.	IFT57	Intraflagellar Transport 57
4248.	KCNS1	Potassium Voltage-Gated Channel Modifier Subfamily S Member 1
4249.	LGALS9	Galectin 9
4250.	MSI2	Musashi RNA Binding Protein 2
4251.	NCR1	Natural Cytotoxicity Triggering Receptor 1
4252.	PBX3	PBX Homeobox 3
4253.	UBE2L6	Ubiquitin Conjugating Enzyme E2 L6
4254.	ARL2BP	ADP Ribosylation Factor Like GTPase 2 Binding Protein
4255.	CDNF	Cerebral Dopamine Neurotrophic Factor
4256.	HOXA7	Homeobox A7
4257.	NANOG	Nanog Homeobox

4258.	NCR2	Natural Cytotoxicity Triggering Receptor 2
4259.	SEPTIN5	Septin 5
4260.	SRRM2	Serine/Arginine Repetitive Matrix 2
4261.	TRAPPC10	Trafficking Protein Particle Complex Subunit 10
4262.	ULBP1	UL16 Binding Protein 1
4263.	WTAP	WT1 Associated Protein
4264.	ZCCHC8	Zinc Finger CCHC-Type Containing 8
4265.	LYL1	LYL1 Basic Helix-Loop-Helix Family Member
4266.	MYBPC2	Myosin Binding Protein C2
4267.	TNFSF9	TNF Superfamily Member 9
4268.	YTHDC2	YTH N6-Methyladenosine RNA Binding Protein C2
4269.	ZNF746	Zinc Finger Protein 746
4270.	ALKBH5	AlkB Homolog 5, RNA Demethylase
4271.	ALPK3	Alpha Kinase 3
4272.	ASPG	Asparaginase
4273.	PHACTR2	Phosphatase And Actin Regulator 2
4274.	YTHDF3	YTH N6-Methyladenosine RNA Binding Protein F3
4275.	ALKBH1	AlkB Homolog 1, Histone H2A Dioxygenase
4276.	ANXA8	Annexin A8
4277.	HS1BP3	HCLS1 Binding Protein 3
4278.	MTIF3	Mitochondrial Translational Initiation Factor 3
4279.	BAALC	BAALC Binder Of MAP3K1 And KLF4
4280.	VIRMA	Vir Like M6A Methyltransferase Associated
4281.	SELENOT	Selenoprotein T
4282.	FREM3	FRAS1 Related Extracellular Matrix 3
4283.	LY6G6D	Lymphocyte Antigen 6 Family Member G6D
4284.	COMMD3-BMI1	COMMD3-BMI1 Readthrough
4285.	FKBP1A	FKBP Prolyl Isomerase 1A
4286.	ITGA3	Integrin Subunit Alpha 3
4287.	SRD5A1	Steroid 5 Alpha-Reductase 1
4288.	NDUFA10	NADH:Ubiquinone Oxidoreductase Subunit A10
4289.	NFATC4	Nuclear Factor Of Activated T Cells 4
4290.	SEZ6L2	Seizure Related 6 Homolog Like 2
4291.	LYRM7	LYR Motif Containing 7
4292.	PPARGC1B	PPARG Coactivator 1 Beta
4293.	CDK8	Cyclin Dependent Kinase 8
4294.	POLH	DNA Polymerase Eta
4295.	PLOD2	Procollagen-Lysine,2-Oxoglutarate 5-Dioxygenase 2
4296.	ARID1B	AT-Rich Interaction Domain 1B
4297.	KIF1A	Kinesin Family Member 1A
4298.	SP7	Sp7 Transcription Factor

4299.	AP3D1	Adaptor Related Protein Complex 3 Subunit Delta 1
4300.	CREB3L1	CAMP Responsive Element Binding Protein 3 Like 1
4301.	CRTAP	Cartilage Associated Protein
4302.	FKBP10	FKBP Prolyl Isomerase 10
4303.	MBTPS2	Membrane Bound Transcription Factor Peptidase, Site 2
4304.	P3H1	Prolyl 3-Hydroxylase 1
4305.	PPM1B	Protein Phosphatase, Mg2+/Mn2+ Dependent 1B
4306.	TMEM38B	Transmembrane Protein 38B
4307.	ACTL6A	Actin Like 6A
4308.	ANKH	ANKH Inorganic Pyrophosphate Transport Regulator
4309.	MED25	Mediator Complex Subunit 25
4310.	KDELR2	KDEL Endoplasmic Reticulum Protein Retention Receptor 2
4311.	MESD	Mesoderm Development LRP Chaperone
4312.	TSEN54	TRNA Splicing Endonuclease Subunit 54
4313.	CCDC134	Coiled-Coil Domain Containing 134
4314.	IFITM5	Interferon Induced Transmembrane Protein 5
4315.	KLHDC8B	Kelch Domain Containing 8B
4316.	PGGHG	Protein-Glucosylgalactosylhydroxylysine Glucosidase
4317.	PHLDB1	Pleckstrin Homology Like Domain Family B Member 1
4318.	RALGAPB	Ral GTPase Activating Protein Non-Catalytic Subunit Beta
4319.	CAMKMT	Calmodulin-Lysine N-Methyltransferase
4320.	TENT5A	Terminal Nucleotidyltransferase 5A
4321.	TOR1AIP2	Torsin 1A Interacting Protein 2
4322.	SNX22	Sorting Nexin 22
4323.	IRAK1BP1	Interleukin 1 Receptor Associated Kinase 1 Binding Protein 1
4324.	GALNS	Galactosamine (N-Acetyl)-6-Sulfatase
4325.	RRAS2	RAS Related 2
4326.	CTSA	Cathepsin A
4327.	FBLN5	Fibulin 5
4328.	HSD17B4	Hydroxysteroid 17-Beta Dehydrogenase 4
4329.	CA8	Carbonic Anhydrase 8
4330.	CLPP	Caseinolytic Mitochondrial Matrix Peptidase Proteolytic Subunit
4331.	SUCLA2	Succinate-CoA Ligase ADP-Forming Subunit Beta
4332.	AGA	Aspartylglucosaminidase
4333.	NAGA	Alpha-N-Acetylgalactosaminidase
4334.	SUOX	Sulfite Oxidase
4335.	A4GALT	Alpha 1,4-Galactosyltransferase (P1PK Blood Group)
4336.	CUL2	Cullin 2
4337.	LZTR1	Leucine Zipper Like Post Translational Regulator 1
4338.	CA14	Carbonic Anhydrase 14
4339.	PDGFRL	Platelet Derived Growth Factor Receptor Like

4340.	PI4K2A	Phosphatidylinositol 4-Kinase Type 2 Alpha
4341.	PYCARD	PYD And CARD Domain Containing
4342.	TIMM8A	Translocase Of Inner Mitochondrial Membrane 8A
4343.	CEBPD	CCAAT Enhancer Binding Protein Delta
4344.	RNASE1	Ribonuclease A Family Member 1, Pancreatic
4345.	ALDH1L1	Aldehyde Dehydrogenase 1 Family Member L1
4346.	ELOB	Elongin B
4347.	GAN	Gigaxonin
4348.	CA5B	Carbonic Anhydrase 5B
4349.	DDHD1	DDHD Domain Containing 1
4350.	HIF3A	Hypoxia Inducible Factor 3 Subunit Alpha
4351.	NEK7	NIMA Related Kinase 7
4352.	RAB4A	RAB4A, Member RAS Oncogene Family
4353.	RETREG1	Reticulophagy Regulator 1
4354.	TIMM17A	Translocase Of Inner Mitochondrial Membrane 17A
4355.	ERAL1	Era Like 12S Mitochondrial RRNA Chaperone 1
4356.	GSDMD	Gasdermin D
4357.	NLRP6	NLR Family Pyrin Domain Containing 6
4358.	SS18	SS18 Subunit Of BAF Chromatin Remodeling Complex
4359.	TIMM13	Translocase Of Inner Mitochondrial Membrane 13
4360.	TIMM44	Translocase Of Inner Mitochondrial Membrane 44
4361.	CA13	Carbonic Anhydrase 13
4362.	ERGIC3	ERGIC And Golgi 3
4363.	GNPTG	N-Acetylglucosamine-1-Phosphate Transferase Subunit Gamma
4364.	PAM16	Presequence Translocase Associated Motor 16
4365.	PITPNM1	Phosphatidylinositol Transfer Protein Membrane Associated 1
4366.	PLEK2	Pleckstrin 2
4367.	SFXN3	Sideroflexin 3
4368.	TIMM22	Translocase Of Inner Mitochondrial Membrane 22
4369.	TIMM10B	Translocase Of Inner Mitochondrial Membrane 10B
4370.	TIMM17B	Translocase Of Inner Mitochondrial Membrane 17B
4371.	TIMM8B	Translocase Of Inner Mitochondrial Membrane 8 Homolog B
4372.	TOMM70	Translocase Of Outer Mitochondrial Membrane 70
4373.	NAGPA	N-Acetylglucosamine-1-Phosphodiester Alpha-N-Acetylglucosaminidase
4374.	TFAP2E	Transcription Factor AP-2 Epsilon
4375.	STOML3	Stomatin Like 3
4376.	TIMM21	Translocase Of Inner Mitochondrial Membrane 21
4377.	TOMM5	Translocase Of Outer Mitochondrial Membrane 5
4378.	TMEM266	Transmembrane Protein 266
4379.	TIMM29	Translocase Of Inner Mitochondrial Membrane 29
4380.	ZNF492	Zinc Finger Protein 492

4381.	TRIM48	Tripartite Motif Containing 48
4382.	VHLL	VHL Like
4383.	PNMA6A	PNMA Family Member 6A
4384.	ZNF814	Zinc Finger Protein 814
4385.	PER1	Period Circadian Regulator 1
4386.	SRI	Sorcin
4387.	SCD	Stearoyl-CoA Desaturase
4388.	ACACA	Acetyl-CoA Carboxylase Alpha
4389.	GABRB3	Gamma-Aminobutyric Acid Type A Receptor Subunit Beta3
4390.	PIM1	Pim-1 Proto-Oncogene, Serine/Threonine Kinase
4391.	PRKAA2	Protein Kinase AMP-Activated Catalytic Subunit Alpha 2
4392.	SREBF1	Sterol Regulatory Element Binding Transcription Factor 1
4393.	FOLR1	Folate Receptor Alpha
4394.	CSTB	Cystatin B
4395.	GLRB	Glycine Receptor Beta
4396.	GNB3	G Protein Subunit Beta 3
4397.	HPGD	15-Hydroxyprostaglandin Dehydrogenase
4398.	SERPINI1	Serpin Family I Member 1
4399.	ADH5	Alcohol Dehydrogenase 5 (Class III), Chi Polypeptide
4400.	AOC3	Amine Oxidase Copper Containing 3
4401.	DUSP1	Dual Specificity Phosphatase 1
4402.	GLO1	Glyoxalase I
4403.	MMUT	Methylmalonyl-CoA Mutase
4404.	NDUFS7	NADH:Ubiquinone Oxidoreductase Core Subunit S7
4405.	PRDX2	Peroxiredoxin 2
4406.	RANBP2	RAN Binding Protein 2
4407.	ANGPTL4	Angiopoietin Like 4
4408.	APOA2	Apolipoprotein A2
4409.	ARRB1	Arrestin Beta 1
4410.	GPD1	Glycerol-3-Phosphate Dehydrogenase 1
4411.	SLC6A5	Solute Carrier Family 6 Member 5
4412.	ACACB	Acetyl-CoA Carboxylase Beta
4413.	APOC2	Apolipoprotein C2
4414.	FABP3	Fatty Acid Binding Protein 3
4415.	MLXIPL	MLX Interacting Protein Like
4416.	NDUFS8	NADH:Ubiquinone Oxidoreductase Core Subunit S8
4417.	NIPBL	NIPBL Cohesin Loading Factor
4418.	OFD1	OFD1 Centriole And Centriolar Satellite Protein
4419.	PCDH19	Protocadherin 19
4420.	PDIA3	Protein Disulfide Isomerase Family A Member 3
4421.	SALL4	Spalt Like Transcription Factor 4

4422.	SETDB1	SET Domain Bifurcated Histone Lysine Methyltransferase 1
4423.	TIMP3	TIMP Metallopeptidase Inhibitor 3
4424.	ATP5PO	ATP Synthase Peripheral Stalk Subunit OSCP
4425.	CDIPT	CDP-Diacylglycerol--Inositol 3-Phosphatidyltransferase
4426.	DDX11	DEAD/H-Box Helicase 11
4427.	MAN2A1	Mannosidase Alpha Class 2A Member 1
4428.	NDUFB8	NADH:Ubiquinone Oxidoreductase Subunit B8
4429.	UQCRB	Ubiquinol-Cytochrome C Reductase Binding Protein
4430.	ACD	ACD Shelterin Complex Subunit And Telomerase Recruitment Factor
4431.	ADIPOR2	Adiponectin Receptor 2
4432.	BBS10	Bardet-Biedl Syndrome 10
4433.	BBS2	Bardet-Biedl Syndrome 2
4434.	BBS4	Bardet-Biedl Syndrome 4
4435.	CYB5R1	Cytochrome B5 Reductase 1
4436.	GPAM	Glycerol-3-Phosphate Acyltransferase, Mitochondrial
4437.	IFT122	Intraflagellar Transport 122
4438.	IFT88	Intraflagellar Transport 88
4439.	KCNJ15	Potassium Inwardly Rectifying Channel Subfamily J Member 15
4440.	KIF3A	Kinesin Family Member 3A
4441.	MKS1	MKS Transition Zone Complex Subunit 1
4442.	MTO1	Mitochondrial TRNA Translation Optimization 1
4443.	PLSCR1	Phospholipid Scramblase 1
4444.	RBCK1	RANBP2-Type And C3HC4-Type Zinc Finger Containing 1
4445.	SLC22A7	Solute Carrier Family 22 Member 7
4446.	SLC30A10	Solute Carrier Family 30 Member 10
4447.	SMARCA5	SWI/SNF Related, Matrix Associated, Actin Dependent Regulator Of Chromatin, Subfamily A, Member 5
4448.	TMLHE	Trimethyllysine Hydroxylase, Epsilon
4449.	XPNPEP3	X-Prolyl Aminopeptidase 3
4450.	BBS9	Bardet-Biedl Syndrome 9
4451.	CNNM2	Cyclin And CBS Domain Divalent Metal Cation Transport Mediator 2
4452.	LMOD1	Leiomodin 1
4453.	PCDH10	Protocadherin 10
4454.	SRP72	Signal Recognition Particle 72
4455.	ARL13B	ADP Ribosylation Factor Like GTPase 13B
4456.	BBS5	Bardet-Biedl Syndrome 5
4457.	BBS7	Bardet-Biedl Syndrome 7
4458.	CDH16	Cadherin 16
4459.	CPD	Carboxypeptidase D
4460.	ELF4	E74 Like ETS Transcription Factor 4
4461.	FGL2	Fibrinogen Like 2
4462.	GJD2	Gap Junction Protein Delta 2

4463.	HAP1	Huntingtin Associated Protein 1
4464.	IFT43	Intraflagellar Transport 43
4465.	RPGRIP1	RPGR Interacting Protein 1
4466.	RPL22	Ribosomal Protein L22
4467.	RPL28	Ribosomal Protein L28
4468.	SON	SON DNA And RNA Binding Protein
4469.	TCTN2	Tectonic Family Member 2
4470.	TCTN3	Tectonic Family Member 3
4471.	TNNI1	Troponin I1, Slow Skeletal Type
4472.	B9D2	B9 Domain Containing 2
4473.	CYB5R2	Cytochrome B5 Reductase 2
4474.	CYB5R4	Cytochrome B5 Reductase 4
4475.	H1-0	H1.0 Linker Histone
4476.	IFT74	Intraflagellar Transport 74
4477.	MMAA	Metabolism Of Cobalamin Associated A
4478.	PKHD1	PKHD1 Ciliary IPT Domain Containing Fibrocystin/Polyductin
4479.	RPL6	Ribosomal Protein L6
4480.	TCTN1	Tectonic Family Member 1
4481.	CLIC2	Chloride Intracellular Channel 2
4482.	IFT80	Intraflagellar Transport 80
4483.	PDS5A	PDS5 Cohesin Associated Factor A
4484.	SPATA7	Spermatogenesis Associated 7
4485.	TMEM138	Transmembrane Protein 138
4486.	TMEM231	Transmembrane Protein 231
4487.	UCN	Urocortin
4488.	B9D1	B9 Domain Containing 1
4489.	CEP83	Centrosomal Protein 83
4490.	CEP97	Centrosomal Protein 97
4491.	CLUAP1	Clusterin Associated Protein 1
4492.	CTRB1	Chymotrypsinogen B1
4493.	IFT52	Intraflagellar Transport 52
4494.	KCNMB3	Potassium Calcium-Activated Channel Subfamily M Regulatory Beta Subunit 3
4495.	RSF1	Remodeling And Spacing Factor 1
4496.	TKTL2	Transketolase Like 2
4497.	BBS12	Bardet-Biedl Syndrome 12
4498.	IFT20	Intraflagellar Transport 20
4499.	IFT70B	Intraflagellar Transport 70B
4500.	TMEM216	Transmembrane Protein 216
4501.	ACAD11	Acyl-CoA Dehydrogenase Family Member 11
4502.	IER3IP1	Immediate Early Response 3 Interacting Protein 1
4503.	LAMTOR5	Late Endosomal/Lysosomal Adaptor, MAPK And MTOR Activator 5

4504.	UNC80	Unc-80 Homolog, NALCN Channel Complex Subunit
4505.	CTRB2	Chymotrypsinogen B2
4506.	RETNLB	Resistin Like Beta
4507.	EPHX3	Epoxide Hydrolase 3
4508.	CYB5RL	Cytochrome B5 Reductase Like
4509.	DCC	DCC Netrin 1 Receptor
4510.	NTN1	Netrin 1
4511.	GJA8	Gap Junction Protein Alpha 8
4512.	KIDINS220	Kinase D Interacting Substrate 220
4513.	MECR	Mitochondrial Trans-2-Enoyl-CoA Reductase
4514.	HOXB1	Homeobox B1
4515.	AFF4	ALF Transcription Elongation Factor 4
4516.	DNAL4	Dynein Axonemal Light Chain 4
4517.	CWF19L1	CWF19 Like Cell Cycle Control Factor 1
4518.	CFAP45	Cilia And Flagella Associated Protein 45
4519.	NECTIN4	Nectin Cell Adhesion Molecule 4
4520.	NRF1	Nuclear Respiratory Factor 1
4521.	PAX7	Paired Box 7
4522.	SIK2	Salt Inducible Kinase 2
4523.	ABCG1	ATP Binding Cassette Subfamily G Member 1
4524.	ADRB3	Adrenoceptor Beta 3
4525.	UGT2B7	UDP Glucuronosyltransferase Family 2 Member B7
4526.	AGRP	Agouti Related Neuropeptide
4527.	COG2	Component Of Oligomeric Golgi Complex 2
4528.	CSRP1	Cysteine And Glycine Rich Protein 1
4529.	ORM1	Orosomucoid 1
4530.	ORM2	Orosomucoid 2
4531.	WAPL	WAPL Cohesin Release Factor
4532.	IDO2	Indoleamine 2,3-Dioxygenase 2
4533.	AMY1B	Amylase Alpha 1B
4534.	CSNK1E	Casein Kinase 1 Epsilon
4535.	NR1D1	Nuclear Receptor Subfamily 1 Group D Member 1
4536.	CRY1	Cryptochrome Circadian Regulator 1
4537.	BHLHE40	Basic Helix-Loop-Helix Family Member E40
4538.	BHLHE41	Basic Helix-Loop-Helix Family Member E41
4539.	CRY2	Cryptochrome Circadian Regulator 2
4540.	NFIL3	Nuclear Factor, Interleukin 3 Regulated
4541.	TIPIN	TIMELESS Interacting Protein
4542.	CAMK2A	Calcium/Calmodulin Dependent Protein Kinase II Alpha
4543.	PPP3CA	Protein Phosphatase 3 Catalytic Subunit Alpha
4544.	GRIA4	Glutamate Ionotropic Receptor AMPA Type Subunit 4

4545.	GSK3A	Glycogen Synthase Kinase 3 Alpha
4546.	SLC1A1	Solute Carrier Family 1 Member 1
4547.	ATF6	Activating Transcription Factor 6
4548.	MAP3K5	Mitogen-Activated Protein Kinase Kinase Kinase 5
4549.	PIKFYVE	Phosphoinositide Kinase, FYVE-Type Zinc Finger Containing
4550.	PTGER2	Prostaglandin E Receptor 2
4551.	TUBA1A	Tubulin Alpha 1a
4552.	ERN1	Endoplasmic Reticulum To Nucleus Signaling 1
4553.	LRP6	LDL Receptor Related Protein 6
4554.	MSN	Moesin
4555.	PRKCH	Protein Kinase C Eta
4556.	TPT1	Tumor Protein, Translationally-Controlled 1
4557.	TUBA4A	Tubulin Alpha 4a
4558.	APRT	Adenine Phosphoribosyltransferase
4559.	CAMKK2	Calcium/Calmodulin Dependent Protein Kinase Kinase 2
4560.	CDON	Cell Adhesion Associated, Oncogene Regulated
4561.	CLDN1	Claudin 1
4562.	MARK3	Microtubule Affinity Regulating Kinase 3
4563.	NTF4	Neurotrophin 4
4564.	PTPN12	Protein Tyrosine Phosphatase Non-Receptor Type 12
4565.	SGPL1	Sphingosine-1-Phosphate Lyase 1
4566.	SLC2A9	Solute Carrier Family 2 Member 9
4567.	TYRP1	Tyrosinase Related Protein 1
4568.	UBE2N	Ubiquitin Conjugating Enzyme E2 N
4569.	ALDH18A1	Aldehyde Dehydrogenase 18 Family Member A1
4570.	ALDH3A2	Aldehyde Dehydrogenase 3 Family Member A2
4571.	ALPI	Alkaline Phosphatase, Intestinal
4572.	BMX	BMX Non-Receptor Tyrosine Kinase
4573.	CANX	Calnexin
4574.	CCNH	Cyclin H
4575.	CDK7	Cyclin Dependent Kinase 7
4576.	CTSC	Cathepsin C
4577.	DDR1	Discoidin Domain Receptor Tyrosine Kinase 1
4578.	FDPS	Farnesyl Diphosphate Synthase
4579.	FGF17	Fibroblast Growth Factor 17
4580.	FZD6	Frizzled Class Receptor 6
4581.	GNAO1	G Protein Subunit Alpha O1
4582.	PIK3R4	Phosphoinositide-3-Kinase Regulatory Subunit 4
4583.	PRODH	Proline Dehydrogenase 1
4584.	RTN4R	Reticulon 4 Receptor
4585.	SAG	S-Antigen Visual Arrestin

4586.	SLC22A12	Solute Carrier Family 22 Member 12
4587.	TRIM24	Tripartite Motif Containing 24
4588.	BUB3	BUB3 Mitotic Checkpoint Protein
4589.	COASY	Coenzyme A Synthase
4590.	DLX5	Distal-Less Homeobox 5
4591.	E2F4	E2F Transcription Factor 4
4592.	EMX2	Empty Spiracles Homeobox 2
4593.	FA2H	Fatty Acid 2-Hydroxylase
4594.	FDXR	Ferredoxin Reductase
4595.	FPR1	Formyl Peptide Receptor 1
4596.	HDAC11	Histone Deacetylase 11
4597.	MADD	MAP Kinase Activating Death Domain
4598.	MAP1B	Microtubule Associated Protein 1B
4599.	MYO5A	Myosin VA
4600.	NPR3	Natriuretic Peptide Receptor 3
4601.	NRCAM	Neuronal Cell Adhesion Molecule
4602.	PITX1	Paired Like Homeodomain 1
4603.	PLOD3	Procollagen-Lysine,2-Oxoglutarate 5-Dioxygenase 3
4604.	SLC1A5	Solute Carrier Family 1 Member 5
4605.	SLC1A6	Solute Carrier Family 1 Member 6
4606.	SLC7A11	Solute Carrier Family 7 Member 11
4607.	SLC7A7	Solute Carrier Family 7 Member 7
4608.	SMAD1	SMAD Family Member 1
4609.	TDP1	Tyrosyl-DNA Phosphodiesterase 1
4610.	AKR1D1	Aldo-Keto Reductase Family 1 Member D1
4611.	ALDH6A1	Aldehyde Dehydrogenase 6 Family Member A1
4612.	ANXA6	Annexin A6
4613.	CCNK	Cyclin K
4614.	CNTFR	Ciliary Neurotrophic Factor Receptor
4615.	COQ7	Coenzyme Q7, Hydroxylase
4616.	CTTN	Cortactin
4617.	EFNB2	Ephrin B2
4618.	EPS15	Epidermal Growth Factor Receptor Pathway Substrate 15
4619.	GART	Phosphoribosylglycinamide Formyltransferase, Phosphoribosylglycinamide Synthetase, Phosphoribosylaminoimidazole Synthetase
4620.	GDA	Guanine Deaminase
4621.	IARS1	Isoleucyl-TRNA Synthetase 1
4622.	IGFBP4	Insulin Like Growth Factor Binding Protein 4
4623.	IGFBP6	Insulin Like Growth Factor Binding Protein 6
4624.	MANBA	Mannosidase Beta
4625.	MYOF	Myoferlin
4626.	PHYH	Phytanoyl-CoA 2-Hydroxylase

4627.	PTPRZ1	Protein Tyrosine Phosphatase Receptor Type Z1
4628.	RBP3	Retinol Binding Protein 3
4629.	RBX1	Ring-Box 1
4630.	RIPK3	Receptor Interacting Serine/Threonine Kinase 3
4631.	RORC	RAR Related Orphan Receptor C
4632.	SLC20A1	Solute Carrier Family 20 Member 1
4633.	TBX3	T-Box Transcription Factor 3
4634.	TYROBP	Transmembrane Immune Signaling Adaptor TYROBP
4635.	VNN1	Vanin 1
4636.	WASF1	WASP Family Member 1
4637.	YARS1	Tyrosyl-TRNA Synthetase 1
4638.	ZIC2	Zic Family Member 2
4639.	BAIAP2	BAR/IMD Domain Containing Adaptor Protein 2
4640.	CCNC	Cyclin C
4641.	COPA	COPI Coat Complex Subunit Alpha
4642.	CUL4A	Cullin 4A
4643.	ENTPD2	Ectonucleoside Triphosphate Diphosphohydrolase 2
4644.	GABARAP	GABA Type A Receptor-Associated Protein
4645.	GABARAPL2	GABA Type A Receptor Associated Protein Like 2
4646.	GPR143	G Protein-Coupled Receptor 143
4647.	HARS2	Histidyl-TRNA Synthetase 2, Mitochondrial
4648.	HOXA11	Homeobox A11
4649.	IRF6	Interferon Regulatory Factor 6
4650.	KDM4C	Lysine Demethylase 4C
4651.	KDSR	3-Ketodihydrosphingosine Reductase
4652.	KHDRBS1	KH RNA Binding Domain Containing, Signal Transduction Associated 1
4653.	KIF5C	Kinesin Family Member 5C
4654.	KRT13	Keratin 13
4655.	LAP3	Leucine Aminopeptidase 3
4656.	LIAS	Lipoic Acid Synthetase
4657.	MAN2B1	Mannosidase Alpha Class 2B Member 1
4658.	MYO5B	Myosin VB
4659.	NCAPH	Non-SMC Condensin I Complex Subunit H
4660.	PAICS	Phosphoribosylaminoimidazole Carboxylase And Phosphoribosylaminoimidazolesuccinocarboxamide Synthase
4661.	PLS3	Plastin 3
4662.	PMEL	Premelanosome Protein
4663.	PRDX3	Peroxiredoxin 3
4664.	PSMB10	Proteasome 20S Subunit Beta 10
4665.	RAD23A	RAD23 Homolog A, Nucleotide Excision Repair Protein
4666.	RBM8A	RNA Binding Motif Protein 8A
4667.	S1PR2	Sphingosine-1-Phosphate Receptor 2

4668.	SBF1	SET Binding Factor 1
4669.	SDC2	Syndecan 2
4670.	SEC24D	SEC24 Homolog D, COPII Coat Complex Component
4671.	SLC17A7	Solute Carrier Family 17 Member 7
4672.	SLC1A7	Solute Carrier Family 1 Member 7
4673.	SLC26A5	Solute Carrier Family 26 Member 5
4674.	SLC2A5	Solute Carrier Family 2 Member 5
4675.	SLC6A6	Solute Carrier Family 6 Member 6
4676.	UFD1	Ubiquitin Recognition Factor In ER Associated Degradation 1
4677.	VAC14	VAC14 Component Of PIKFYVE Complex
4678.	VAPA	VAMP Associated Protein A
4679.	VPS11	VPS11 Core Subunit Of CORVET And HOPS Complexes
4680.	ABCC11	ATP Binding Cassette Subfamily C Member 11
4681.	ACTR2	Actin Related Protein 2
4682.	ARPC2	Actin Related Protein 2/3 Complex Subunit 2
4683.	ARPC3	Actin Related Protein 2/3 Complex Subunit 3
4684.	CA5A	Carbonic Anhydrase 5A
4685.	CACNA2D4	Calcium Voltage-Gated Channel Auxiliary Subunit Alpha2delta 4
4686.	CETN2	Centrin 2
4687.	CPT1C	Carnitine Palmitoyltransferase 1C
4688.	CRB2	Crumbs Cell Polarity Complex Component 2
4689.	CYTH1	Cytohesin 1
4690.	DIAPH3	Diaphanous Related Formin 3
4691.	EIF2B3	Eukaryotic Translation Initiation Factor 2B Subunit Gamma
4692.	ETS2	ETS Proto-Oncogene 2, Transcription Factor
4693.	EXOSC9	Exosome Component 9
4694.	FBXO11	F-Box Protein 11
4695.	FGF13	Fibroblast Growth Factor 13
4696.	FMO5	Flavin Containing Dimethylaniline Monoxygenase 5
4697.	FMOD	Fibromodulin
4698.	GLUD2	Glutamate Dehydrogenase 2
4699.	GNA14	G Protein Subunit Alpha 14
4700.	GOPC	Golgi Associated PDZ And Coiled-Coil Motif Containing
4701.	GTF2H1	General Transcription Factor IIH Subunit 1
4702.	HMGB2	High Mobility Group Box 2
4703.	HMGN1	High Mobility Group Nucleosome Binding Domain 1
4704.	KCNJ4	Potassium Inwardly Rectifying Channel Subfamily J Member 4
4705.	LAMTOR2	Late Endosomal/Lysosomal Adaptor, MAPK And MTOR Activator 2
4706.	LMAN2L	Lectin, Mannose Binding 2 Like
4707.	MACF1	Microtubule Actin Crosslinking Factor 1
4708.	MAP1LC3B	Microtubule Associated Protein 1 Light Chain 3 Beta

4709.	NCK1	NCK Adaptor Protein 1
4710.	PDGFC	Platelet Derived Growth Factor C
4711.	PTTG1	PTTG1 Regulator Of Sister Chromatid Separation, Securin
4712.	RAB3GAP1	RAB3 GTPase Activating Protein Catalytic Subunit 1
4713.	RAB6A	RAB6A, Member RAS Oncogene Family
4714.	RANGAP1	Ran GTPase Activating Protein 1
4715.	SARM1	Sterile Alpha And TIR Motif Containing 1
4716.	SEPTIN9	Septin 9
4717.	SLC13A3	Solute Carrier Family 13 Member 3
4718.	SLC45A2	Solute Carrier Family 45 Member 2
4719.	STX4	Syntaxin 4
4720.	TNNT3	Troponin T3, Fast Skeletal Type
4721.	TNPO1	Transportin 1
4722.	UBASH3B	Ubiquitin Associated And SH3 Domain Containing B
4723.	VPS33B	VPS33B Late Endosome And Lysosome Associated
4724.	ADARB2	Adenosine Deaminase RNA Specific B2 (Inactive)
4725.	B3GALNT2	Beta-1,3-N-Acetylgalactosaminyltransferase 2
4726.	CPM	Carboxypeptidase M
4727.	ERAP2	Endoplasmic Reticulum Aminopeptidase 2
4728.	FDX1	Ferredoxin 1
4729.	FRS2	Fibroblast Growth Factor Receptor Substrate 2
4730.	GABARAPL1	GABA Type A Receptor Associated Protein Like 1
4731.	HOGA1	4-Hydroxy-2-Oxoglutarate Aldolase 1
4732.	INSRR	Insulin Receptor Related Receptor
4733.	ITSN1	Intersectin 1
4734.	LEFTY1	Left-Right Determination Factor 1
4735.	MBD2	Methyl-CpG Binding Domain Protein 2
4736.	MC5R	Melanocortin 5 Receptor
4737.	MLPH	Melanophilin
4738.	MMRN1	Multimerin 1
4739.	POLR3K	RNA Polymerase III Subunit K
4740.	RAB1A	RAB1A, Member RAS Oncogene Family
4741.	RAB33B	RAB33B, Member RAS Oncogene Family
4742.	RPS6KA6	Ribosomal Protein S6 Kinase A6
4743.	SMCHD1	Structural Maintenance Of Chromosomes Flexible Hinge Domain Containing 1
4744.	TRIM2	Tripartite Motif Containing 2
4745.	ACTR3	Actin Related Protein 3
4746.	AGFG1	ArfGAP With FG Repeats 1
4747.	ATG12	Autophagy Related 12
4748.	ATP8A1	ATPase Phospholipid Transporting 8A1
4749.	B4GALT3	Beta-1,4-Galactosyltransferase 3

4750.	B4GALT7	Beta-1,4-Galactosyltransferase 7
4751.	COLGALT1	Collagen Beta(1-O)Galactosyltransferase 1
4752.	DHTKD1	Dehydrogenase E1 And Transketolase Domain Containing 1
4753.	DLX3	Distal-Less Homeobox 3
4754.	DNAJC7	DnaJ Heat Shock Protein Family (Hsp40) Member C7
4755.	FGF16	Fibroblast Growth Factor 16
4756.	FGF6	Fibroblast Growth Factor 6
4757.	FXR2	FMR1 Autosomal Homolog 2
4758.	GALNT12	Polypeptide N-Acetylgalactosaminyltransferase 12
4759.	GLIPR1	GLI Pathogenesis Related 1
4760.	HFM1	Helicase For Meiosis 1
4761.	HIF1AN	Hypoxia Inducible Factor 1 Subunit Alpha Inhibitor
4762.	LRSAM1	Leucine Rich Repeat And Sterile Alpha Motif Containing 1
4763.	MOCS1	Molybdenum Cofactor Synthesis 1
4764.	MPG	N-Methylpurine DNA Glycosylase
4765.	MT3	Metallothionein 3
4766.	MTDH	Metadherin
4767.	NBR1	NBR1 Autophagy Cargo Receptor
4768.	NEUROG1	Neurogenin 1
4769.	PDIA2	Protein Disulfide Isomerase Family A Member 2
4770.	PHC1	Polyhomeotic Homolog 1
4771.	POLD2	DNA Polymerase Delta 2, Accessory Subunit
4772.	QRSL1	Glutaminyl-TRNA Amidotransferase Subunit QRSL1
4773.	RAB35	RAB35, Member RAS Oncogene Family
4774.	RABGGTA	Rab Geranylgeranyltransferase Subunit Alpha
4775.	RENBP	Renin Binding Protein
4776.	RPN2	Ribophorin II
4777.	RTN2	Reticulon 2
4778.	SCFD1	Sec1 Family Domain Containing 1
4779.	SCRIB	Scribble Planar Cell Polarity Protein
4780.	SIX5	SIX Homeobox 5
4781.	SLC24A5	Solute Carrier Family 24 Member 5
4782.	SLC38A1	Solute Carrier Family 38 Member 1
4783.	SP4	Sp4 Transcription Factor
4784.	SRSF7	Serine And Arginine Rich Splicing Factor 7
4785.	STX7	Syntaxin 7
4786.	SYN2	Synapsin II
4787.	TCF7L1	Transcription Factor 7 Like 1
4788.	TDG	Thymine DNA Glycosylase
4789.	TIAL1	TIA1 Cytotoxic Granule Associated RNA Binding Protein Like 1
4790.	TMEM106B	Transmembrane Protein 106B

4791.	TPPP	Tubulin Polymerization Promoting Protein
4792.	TRAK1	Trafficking Kinesin Protein 1
4793.	UBE2V2	Ubiquitin Conjugating Enzyme E2 V2
4794.	VAMP8	Vesicle Associated Membrane Protein 8
4795.	VNN2	Vanin 2
4796.	VPS33A	VPS33A Core Subunit Of CORVET And HOPS Complexes
4797.	VTI1B	Vesicle Transport Through Interaction With T-SNAREs 1B
4798.	WASF2	WASP Family Member 2
4799.	ABCC12	ATP Binding Cassette Subfamily C Member 12
4800.	ACP6	Acid Phosphatase 6, Lysophosphatidic
4801.	AP3S1	Adaptor Related Protein Complex 3 Subunit Sigma 1
4802.	APBB3	Amyloid Beta Precursor Protein Binding Family B Member 3
4803.	ATL3	Atlastin GTPase 3
4804.	ATOH7	Atonal BHLH Transcription Factor 7
4805.	CCN4	Cellular Communication Network Factor 4
4806.	CHRDL1	Chordin Like 1
4807.	CLEC4G	C-Type Lectin Domain Family 4 Member G
4808.	CPLX2	Complexin 2
4809.	CREB3L2	CAMP Responsive Element Binding Protein 3 Like 2
4810.	DCAF17	DDB1 And CUL4 Associated Factor 17
4811.	DTNB	Dystrobrevin Beta
4812.	EEF1B2	Eukaryotic Translation Elongation Factor 1 Beta 2
4813.	ENAM	Enamelin
4814.	ETNK1	Ethanolamine Kinase 1
4815.	GBP1	Guanylate Binding Protein 1
4816.	GLG1	Golgi Glycoprotein 1
4817.	GPA33	Glycoprotein A33
4818.	GTF2H5	General Transcription Factor IIH Subunit 5
4819.	HNRNPA3	Heterogeneous Nuclear Ribonucleoprotein A3
4820.	IL13RA2	Interleukin 13 Receptor Subunit Alpha 2
4821.	ILF2	Interleukin Enhancer Binding Factor 2
4822.	JDP2	Jun Dimerization Protein 2
4823.	KIF13A	Kinesin Family Member 13A
4824.	KRT12	Keratin 12
4825.	KRT15	Keratin 15
4826.	KRT3	Keratin 3
4827.	LGALS4	Galectin 4
4828.	MAP1A	Microtubule Associated Protein 1A
4829.	MCTP2	Multiple C2 And Transmembrane Domain Containing 2
4830.	MSI1	Musashi RNA Binding Protein 1
4831.	MTMR1	Myotubularin Related Protein 1

4832.	MTSS1	MTSS I-BAR Domain Containing 1
4833.	MYO18A	Myosin XVIIIA
4834.	NEUROG2	Neurogenin 2
4835.	NOS1AP	Nitric Oxide Synthase 1 Adaptor Protein
4836.	NPLOC4	NPL4 Homolog, Ubiquitin Recognition Factor
4837.	PIGV	Phosphatidylinositol Glycan Anchor Biosynthesis Class V
4838.	PLPBP	Pyridoxal Phosphate Binding Protein
4839.	RAB27B	RAB27B, Member RAS Oncogene Family
4840.	RAB38	RAB38, Member RAS Oncogene Family
4841.	RHOG	Ras Homolog Family Member G
4842.	RNGTT	RNA Guanylyltransferase And 5'-Phosphatase
4843.	RYBP	RING1 And YY1 Binding Protein
4844.	SCGN	Secretagogin, EF-Hand Calcium Binding Protein
4845.	SEMA6A	Semaphorin 6A
4846.	SLC26A1	Solute Carrier Family 26 Member 1
4847.	SLC2A6	Solute Carrier Family 2 Member 6
4848.	SMNDC1	Survival Motor Neuron Domain Containing 1
4849.	SNX9	Sorting Nexin 9
4850.	SYTL2	Synaptotagmin Like 2
4851.	TPCN2	Two Pore Segment Channel 2
4852.	TRIP10	Thyroid Hormone Receptor Interactor 10
4853.	TSN	Translin
4854.	TTC7A	Tetratricopeptide Repeat Domain 7A
4855.	USP53	Ubiquitin Specific Peptidase 53
4856.	VIL1	Villin 1
4857.	WASF3	WASP Family Member 3
4858.	WDR1	WD Repeat Domain 1
4859.	WNT16	Wnt Family Member 16
4860.	YKT6	YKT6 V-SNARE Homolog
4861.	ZFYVE26	Zinc Finger FYVE-Type Containing 26
4862.	ADAMDEC1	ADAM Like Decysin 1
4863.	AFF3	ALF Transcription Elongation Factor 3
4864.	AMER1	APC Membrane Recruitment Protein 1
4865.	AP3M1	Adaptor Related Protein Complex 3 Subunit Mu 1
4866.	ARL5B	ADP Ribosylation Factor Like GTPase 5B
4867.	ARSK	Arylsulfatase Family Member K
4868.	ASIP	Agouti Signaling Protein
4869.	ATP13A1	ATPase 13A1
4870.	ATP8A2	ATPase Phospholipid Transporting 8A2
4871.	ATP8B2	ATPase Phospholipid Transporting 8B2
4872.	ATP8B3	ATPase Phospholipid Transporting 8B3

4873.	B3GNT5	UDP-GlcNAc:BetaGal Beta-1,3-N-Acetylglucosaminyltransferase 5
4874.	CCL1	C-C Motif Chemokine Ligand 1
4875.	CEBPZ	CCAAT Enhancer Binding Protein Zeta
4876.	COPZ1	COPI Coat Complex Subunit Zeta 1
4877.	DTL	Denticleless E3 Ubiquitin Protein Ligase Homolog
4878.	DUSP13B	Dual Specificity Phosphatase 13B
4879.	DUSP5	Dual Specificity Phosphatase 5
4880.	EEF1G	Eukaryotic Translation Elongation Factor 1 Gamma
4881.	FGD3	FYVE, RhoGEF And PH Domain Containing 3
4882.	FMN1	Formin 1
4883.	GNLY	Granulysin
4884.	GTF2H4	General Transcription Factor IIH Subunit 4
4885.	GTPBP2	GTP Binding Protein 2
4886.	HSPA14	Heat Shock Protein Family A (Hsp70) Member 14
4887.	IL1F10	Interleukin 1 Family Member 10
4888.	IL31	Interleukin 31
4889.	ISYNA1	Inositol-3-Phosphate Synthase 1
4890.	MAFF	MAF BZIP Transcription Factor F
4891.	MED27	Mediator Complex Subunit 27
4892.	MGAT5B	Alpha-1,6-Mannosylglycoprotein 6-Beta-N-Acetylglucosaminyltransferase B
4893.	MIA3	MIA SH3 Domain ER Export Factor 3
4894.	MMS19	MMS19 Homolog, Cytosolic Iron-Sulfur Assembly Component
4895.	MRPS7	Mitochondrial Ribosomal Protein S7
4896.	NAPB	NSF Attachment Protein Beta
4897.	NAT10	N-Acetyltransferase 10
4898.	NDRG4	NDRG Family Member 4
4899.	NEMF	Nuclear Export Mediator Factor
4900.	OAS2	2'-5'-Oligoadenylate Synthetase 2
4901.	PLEKHG2	Pleckstrin Homology And RhoGEF Domain Containing G2
4902.	PSMG1	Proteasome Assembly Chaperone 1
4903.	RAB3IL1	RAB3A Interacting Protein Like 1
4904.	RNF111	Ring Finger Protein 111
4905.	SCG2	Secretogranin II
4906.	SCGB3A2	Secretoglobin Family 3A Member 2
4907.	SCIN	Scinderin
4908.	SHOX2	SHOX Homeobox 2
4909.	SLC2A13	Solute Carrier Family 2 Member 13
4910.	SNAPIN	SNAP Associated Protein
4911.	SPG21	SPG21 Abhydrolase Domain Containing, Maspardin
4912.	ST13	ST13 Hsp70 Interacting Protein
4913.	STX2	Syntaxin 2

4914.	SV2B	Synaptic Vesicle Glycoprotein 2B
4915.	SV2C	Synaptic Vesicle Glycoprotein 2C
4916.	TCEA1	Transcription Elongation Factor A1
4917.	TMED1	Transmembrane P24 Trafficking Protein 1
4918.	TMEM30A	Transmembrane Protein 30A
4919.	TRIM39	Tripartite Motif Containing 39
4920.	UBA6	Ubiquitin Like Modifier Activating Enzyme 6
4921.	UBE2Z	Ubiquitin Conjugating Enzyme E2 Z
4922.	UBXN6	UBX Domain Protein 6
4923.	UNC45A	Unc-45 Myosin Chaperone A
4924.	VAMP4	Vesicle Associated Membrane Protein 4
4925.	VPS54	VPS54 Subunit Of GARP Complex
4926.	VSX1	Visual System Homeobox 1
4927.	WDR4	WD Repeat Domain 4
4928.	XAB2	XPA Binding Protein 2
4929.	ABHD2	Abhydrolase Domain Containing 2, Acylglycerol Lipase
4930.	ACBD4	Acyl-CoA Binding Domain Containing 4
4931.	ADGRB3	Adhesion G Protein-Coupled Receptor B3
4932.	ARSF	Arylsulfatase F
4933.	ATP10D	ATPase Phospholipid Transporting 10D (Putative)
4934.	ATP6V1E2	ATPase H+ Transporting V1 Subunit E2
4935.	ATP9A	ATPase Phospholipid Transporting 9A (Putative)
4936.	CLEC4E	C-Type Lectin Domain Family 4 Member E
4937.	COQ10B	Coenzyme Q10B
4938.	CSNK1A1L	Casein Kinase 1 Alpha 1 Like
4939.	DRAP1	DR1 Associated Protein 1
4940.	EPN1	Epsin 1
4941.	FGF22	Fibroblast Growth Factor 22
4942.	GORAB	Golgin, RAB6 Interacting
4943.	GPN1	GPN-Loop GTPase 1
4944.	GTF2H2	General Transcription Factor IIH Subunit 2
4945.	GTF2H3	General Transcription Factor IIH Subunit 3
4946.	HLF	HLF Transcription Factor, PAR BZIP Family Member
4947.	INSM1	INSM Transcriptional Repressor 1
4948.	IRX4	Iroquois Homeobox 4
4949.	KLHL9	Kelch Like Family Member 9
4950.	LHX2	LIM Homeobox 2
4951.	LRRFIP2	LRR Binding FLII Interacting Protein 2
4952.	NCKIPSD	NCK Interacting Protein With SH3 Domain
4953.	NDOR1	NADPH Dependent Diflavin Oxidoreductase 1
4954.	P3H3	Prolyl 3-Hydroxylase 3

4955.	P3H4	Prolyl 3-Hydroxylase Family Member 4 (Inactive)
4956.	PHF3	PHD Finger Protein 3
4957.	PRDM5	PR/SET Domain 5
4958.	RAD1	RAD1 Checkpoint DNA Exonuclease
4959.	RPH3AL	Rabphilin 3A Like (Without C2 Domains)
4960.	SH3BP5	SH3 Domain Binding Protein 5
4961.	SHPK	Sedoheptulokinase
4962.	SHPRH	SNF2 Histone Linker PHD RING Helicase
4963.	SIGIRR	Single Ig And TIR Domain Containing
4964.	SSR2	Signal Sequence Receptor Subunit 2
4965.	STX12	Syntaxin 12
4966.	THADA	THADA Armadillo Repeat Containing
4967.	TMEM38A	Transmembrane Protein 38A
4968.	TMPRSS5	Transmembrane Serine Protease 5
4969.	UVSSA	UV Stimulated Scaffold Protein A
4970.	VAMP5	Vesicle Associated Membrane Protein 5
4971.	VAX2	Ventral Anterior Homeobox 2
4972.	VCPIP1	Valosin Containing Protein Interacting Protein 1
4973.	VIPAS39	VPS33B Interacting Protein, Apical-Basolateral Polarity Regulator, Spe-39 Homolog
4974.	VPS16	VPS16 Core Subunit Of CORVET And HOPS Complexes
4975.	VPS18	VPS18 Core Subunit Of CORVET And HOPS Complexes
4976.	ZDHHC15	Zinc Finger DHHC-Type Palmitoyltransferase 15
4977.	ZFYVE27	Zinc Finger FYVE-Type Containing 27
4978.	ABHD10	Abhydrolase Domain Containing 10, Depalmitoylase
4979.	ANKRD27	Ankyrin Repeat Domain 27
4980.	AP3S2	Adaptor Related Protein Complex 3 Subunit Sigma 2
4981.	AREL1	Apoptosis Resistant E3 Ubiquitin Protein Ligase 1
4982.	ARFRP1	ADP Ribosylation Factor Related Protein 1
4983.	BAIAP3	BAI1 Associated Protein 3
4984.	BLOC1S1	Biogenesis Of Lysosomal Organelles Complex 1 Subunit 1
4985.	BLOC1S3	Biogenesis Of Lysosomal Organelles Complex 1 Subunit 3
4986.	BRWD3	Bromodomain And WD Repeat Domain Containing 3
4987.	CARD16	Caspase Recruitment Domain Family Member 16
4988.	CBX8	Chromobox 8
4989.	CENPT	Centromere Protein T
4990.	CENPU	Centromere Protein U
4991.	CLYBL	Citramalyl-CoA Lyase
4992.	CNNM3	Cyclin And CBS Domain Divalent Metal Cation Transport Mediator 3
4993.	CTTNBP2	Cortactin Binding Protein 2
4994.	DENND2B	DENN Domain Containing 2B
4995.	DMWD	DM1 Locus, WD Repeat Containing

4996.	EPM2AIP1	EPM2A Interacting Protein 1
4997.	FMNL1	Formin Like 1
4998.	FOXB1	Forkhead Box B1
4999.	GIMAP2	GTPase, IMAP Family Member 2
5000.	GIMAP4	GTPase, IMAP Family Member 4
5001.	GLOD4	Glyoxalase Domain Containing 4
5002.	GOLGA4	Golgin A4
5003.	GXYLT1	Glucoside Xylosyltransferase 1
5004.	IGSF10	Immunoglobulin Superfamily Member 10
5005.	ITFG1	Integrin Alpha FG-GAP Repeat Containing 1
5006.	LAS1L	LAS1 Like Ribosome Biogenesis Factor
5007.	LILRB3	Leukocyte Immunoglobulin Like Receptor B3
5008.	LPCAT1	Lysophosphatidylcholine Acyltransferase 1
5009.	LRMDA	Leucine Rich Melanocyte Differentiation Associated
5010.	MBNL3	Muscleblind Like Splicing Regulator 3
5011.	MON1A	MON1 Homolog A, Secretory Trafficking Associated
5012.	MPLKIP	M-Phase Specific PLK1 Interacting Protein
5013.	NADK2	NAD Kinase 2, Mitochondrial
5014.	OMP	Olfactory Marker Protein
5015.	POM121	POM121 Transmembrane Nucleoporin
5016.	RAB21	RAB21, Member RAS Oncogene Family
5017.	RAB32	RAB32, Member RAS Oncogene Family
5018.	RHEBL1	RHEB Like 1
5019.	RNASE4	Ribonuclease A Family Member 4
5020.	SGSM1	Small G Protein Signaling Modulator 1
5021.	SGSM2	Small G Protein Signaling Modulator 2
5022.	SH3GLB2	SH3 Domain Containing GRB2 Like, Endophilin B2
5023.	SLC2A11	Solute Carrier Family 2 Member 11
5024.	SLC38A8	Solute Carrier Family 38 Member 8
5025.	SNRPC	Small Nuclear Ribonucleoprotein Polypeptide C
5026.	TAF1B	TATA-Box Binding Protein Associated Factor, RNA Polymerase I Subunit B
5027.	TAPT1	Transmembrane Anterior Posterior Transformation 1
5028.	TCEA2	Transcription Elongation Factor A2
5029.	TCEA3	Transcription Elongation Factor A3
5030.	TECRL	Trans-2,3-Enoyl-CoA Reductase Like
5031.	TMEM119	Transmembrane Protein 119
5032.	TNFSF8	TNF Superfamily Member 8
5033.	TNS2	Tensin 2
5034.	ZNF526	Zinc Finger Protein 526
5035.	ANKZF1	Ankyrin Repeat And Zinc Finger Peptidyl TRNA Hydrolase 1
5036.	ARHGAP22	Rho GTPase Activating Protein 22

5037.	ATF5	Activating Transcription Factor 5
5038.	ATP8B4	ATPase Phospholipid Transporting 8B4 (Putative)
5039.	BHLHE22	Basic Helix-Loop-Helix Family Member E22
5040.	BLOC1S2	Biogenesis Of Lysosomal Organelles Complex 1 Subunit 2
5041.	C19orf12	Chromosome 19 Open Reading Frame 12
5042.	CHAF1A	Chromatin Assembly Factor 1 Subunit A
5043.	COQ10A	Coenzyme Q10A
5044.	CREB3L4	CAMP Responsive Element Binding Protein 3 Like 4
5045.	DPH7	Diphthamide Biosynthesis 7
5046.	DSN1	DSN1 Component Of MIS12 Kinetochore Complex
5047.	FKBP11	FKBP Prolyl Isomerase 11
5048.	GCC2	GRIP And Coiled-Coil Domain Containing 2
5049.	GNG12	G Protein Subunit Gamma 12
5050.	GPR89A	G Protein-Coupled Receptor 89A
5051.	GPR89B	G Protein-Coupled Receptor 89B
5052.	LANCL1	LanC Like Glutathione S-Transferase 1
5053.	LARP4	La Ribonucleoprotein 4
5054.	LTN1	Listerin E3 Ubiquitin Protein Ligase 1
5055.	MORC1	MORC Family CW-Type Zinc Finger 1
5056.	MUC20	Mucin 20, Cell Surface Associated
5057.	NAV3	Neuron Navigator 3
5058.	OXSM	3-Oxoacyl-ACP Synthase, Mitochondrial
5059.	PSTPIP2	Proline-Serine-Threonine Phosphatase Interacting Protein 2
5060.	RABAC1	Rab Acceptor 1
5061.	RBBP9	RB Binding Protein 9, Serine Hydrolase
5062.	RBSN	Rabenosyn, RAB Effector
5063.	RILP	Rab Interacting Lysosomal Protein
5064.	SCAND1	SCAN Domain Containing 1
5065.	SGTB	Small Glutamine Rich Tetratricopeptide Repeat Co-Chaperone Beta
5066.	SLC2A14	Solute Carrier Family 2 Member 14
5067.	SNPH	Syntaphilin
5068.	SPCS1	Signal Peptidase Complex Subunit 1
5069.	TEFM	Transcription Elongation Factor, Mitochondrial
5070.	TMEM147	Transmembrane Protein 147
5071.	TMEM30B	Transmembrane Protein 30B
5072.	UBFD1	Ubiquitin Family Domain Containing 1
5073.	UBQLN3	Ubiquilin 3
5074.	WDR41	WD Repeat Domain 41
5075.	ZNF41	Zinc Finger Protein 41
5076.	ABT1	Activator Of Basal Transcription 1
5077.	ALLC	Allantoicase

5078.	CAGE1	Cancer Antigen 1
5079.	CLUH	Clustered Mitochondria Homolog
5080.	GDAP1L1	Ganglioside Induced Differentiation Associated Protein 1 Like 1
5081.	GPS2	G Protein Pathway Suppressor 2
5082.	MEF2B	Myocyte Enhancer Factor 2B
5083.	MFSD12	Major Facilitator Superfamily Domain Containing 12
5084.	MRAP2	Melanocortin 2 Receptor Accessory Protein 2
5085.	MTG1	Mitochondrial Ribosome Associated GTPase 1
5086.	POLN	DNA Polymerase Nu
5087.	RLN2	Relaxin 2
5088.	RND1	Rho Family GTPase 1
5089.	SLC66A1	Solute Carrier Family 66 Member 1
5090.	USB1	U6 SnRNA Biogenesis Phosphodiesterase 1
5091.	ZC3H12D	Zinc Finger CCCH-Type Containing 12D
5092.	AMN1	Antagonist Of Mitotic Exit Network 1 Homolog
5093.	ANKLE1	Ankyrin Repeat And LEM Domain Containing 1
5094.	CABP1	Calcium Binding Protein 1
5095.	CD302	CD302 Molecule
5096.	CENPQ	Centromere Protein Q
5097.	CLEC4F	C-Type Lectin Domain Family 4 Member F
5098.	IFI44L	Interferon Induced Protein 44 Like
5099.	KXD1	KxDL Motif Containing 1
5100.	MEGF9	Multiple EGF Like Domains 9
5101.	MRPS5	Mitochondrial Ribosomal Protein S5
5102.	PDCD2L	Programmed Cell Death 2 Like
5103.	PGAM4	Phosphoglycerate Mutase Family Member 4
5104.	PRSS58	Serine Protease 58
5105.	PSMB11	Proteasome Subunit Beta 11
5106.	SMCR8	SMCR8-C9orf72 Complex Subunit
5107.	SSX1	SSX Family Member 1
5108.	SUCO	SUN Domain Containing Ossification Factor
5109.	SURF2	Surfeit 2
5110.	TRIM58	Tripartite Motif Containing 58
5111.	TRNAU1AP	TRNA Selenocysteine 1 Associated Protein 1
5112.	UAP1L1	UDP-N-Acetylglucosamine Pyrophosphorylase 1 Like 1
5113.	UBXN7	UBX Domain Protein 7
5114.	YIPF2	Yip1 Domain Family Member 2
5115.	ZNF446	Zinc Finger Protein 446
5116.	ZNF490	Zinc Finger Protein 490
5117.	ANKRD24	Ankyrin Repeat Domain 24
5118.	BLOC1S4	Biogenesis Of Lysosomal Organelles Complex 1 Subunit 4

5119.	CASP8AP2	Caspase 8 Associated Protein 2
5120.	CCDC14	Coiled-Coil Domain Containing 14
5121.	CCDC180	Coiled-Coil Domain Containing 180
5122.	CCZ1	CCZ1 Homolog, Vacuolar Protein Trafficking And Biogenesis Associated
5123.	COMMD3	COMM Domain Containing 3
5124.	CREG2	Cellular Repressor Of E1A Stimulated Genes 2
5125.	CWH43	Cell Wall Biogenesis 43 C-Terminal Homolog
5126.	DBNDD1	Dysbindin Domain Containing 1
5127.	DIRAS3	DIRAS Family GTPase 3
5128.	DNAJC14	DnaJ Heat Shock Protein Family (Hsp40) Member C14
5129.	ENDOV	Endonuclease V
5130.	FAXDC2	Fatty Acid Hydroxylase Domain Containing 2
5131.	GAS2L3	Growth Arrest Specific 2 Like 3
5132.	H3C4	H3 Clustered Histone 4
5133.	LILRA6	Leukocyte Immunoglobulin Like Receptor A6
5134.	NLRP13	NLR Family Pyrin Domain Containing 13
5135.	NUP43	Nucleoporin 43
5136.	PRR11	Proline Rich 11
5137.	RBM33	RNA Binding Motif Protein 33
5138.	SLC35D3	Solute Carrier Family 35 Member D3
5139.	SPAG8	Sperm Associated Antigen 8
5140.	SYCN	Syncollin
5141.	TAF1D	TATA-Box Binding Protein Associated Factor, RNA Polymerase I Subunit D
5142.	UBXN2A	UBX Domain Protein 2A
5143.	WDR89	WD Repeat Domain 89
5144.	ZNF136	Zinc Finger Protein 136
5145.	ASTE1	Asteroid Homolog 1
5146.	CFAP47	Cilia And Flagella Associated Protein 47
5147.	FAM120C	Family With Sequence Similarity 120 Member C
5148.	HAGHL	Hydroxyacylglutathione Hydrolase Like
5149.	LRRC66	Leucine Rich Repeat Containing 66
5150.	OR10J1	Olfactory Receptor Family 10 Subfamily J Member 1
5151.	PCP2	Purkinje Cell Protein 2
5152.	SLC7A13	Solute Carrier Family 7 Member 13
5153.	STX19	Syntaxin 19
5154.	SVIP	Small VCP Interacting Protein
5155.	TLCD1	TLC Domain Containing 1
5156.	TM2D2	TM2 Domain Containing 2
5157.	TOMM40L	Translocase Of Outer Mitochondrial Membrane 40 Like
5158.	UTF1	Undifferentiated Embryonic Cell Transcription Factor 1
5159.	ZCCHC13	Zinc Finger CCHC-Type Containing 13

5160.	ZNF674	Zinc Finger Protein 674
5161.	ZNF706	Zinc Finger Protein 706
5162.	ZNF71	Zinc Finger Protein 71
5163.	AADACL2	Arylacetamide Deacetylase Like 2
5164.	CYTL1	Cytokine Like 1
5165.	MORC4	MORC Family CW-Type Zinc Finger 4
5166.	PHF23	PHD Finger Protein 23
5167.	RHOXF1	Rhox Homeobox Family Member 1
5168.	RTP1	Receptor Transporter Protein 1
5169.	SERP1	Stress Associated Endoplasmic Reticulum Protein 1
5170.	TEX30	Testis Expressed 30
5171.	TP53TG5	TP53 Target 5
5172.	ZNF232	Zinc Finger Protein 232
5173.	ZNF81	Zinc Finger Protein 81
5174.	C2orf49	Chromosome 2 Open Reading Frame 49
5175.	CCZ1B	CCZ1 Homolog B, Vacuolar Protein Trafficking And Biogenesis Associated
5176.	DRGX	Dorsal Root Ganglia Homeobox
5177.	ETFRF1	Electron Transfer Flavoprotein Regulatory Factor 1
5178.	ODR4	Odr-4 GPCR Localization Factor Homolog
5179.	STPG2	Sperm Tail PG-Rich Repeat Containing 2
5180.	TRIM52	Tripartite Motif Containing 52
5181.	ZNF697	Zinc Finger Protein 697
5182.	HIGD1C	HIG1 Hypoxia Inducible Domain Family Member 1C
5183.	IFITM10	Interferon Induced Transmembrane Protein 10
5184.	KLHL33	Kelch Like Family Member 33
5185.	MROH6	Maestro Heat Like Repeat Family Member 6
5186.	OR8J1	Olfactory Receptor Family 8 Subfamily J Member 1
5187.	RTP2	Receptor Transporter Protein 2
5188.	UBXN10	UBX Domain Protein 10
5189.	IGHD	Immunoglobulin Heavy Constant Delta
5190.	OR2T35	Olfactory Receptor Family 2 Subfamily T Member 35
5191.	AADACL4	Arylacetamide Deacetylase Like 4
5192.	FAM170B	Family With Sequence Similarity 170 Member B
5193.	HMSD	Histocompatibility Minor Serpin Domain Containing
5194.	NUDT17	Nudix Hydrolase 17
5195.	OR2T34	Olfactory Receptor Family 2 Subfamily T Member 34
5196.	POM121L12	POM121 Transmembrane Nucleoporin Like 12
5197.	TMEM150C	Transmembrane Protein 150C
5198.	YIPF7	Yip1 Domain Family Member 7
5199.	OR5H14	Olfactory Receptor Family 5 Subfamily H Member 14
5200.	TMEM247	Transmembrane Protein 247

5201.	ZSCAN23	Zinc Finger And SCAN Domain Containing 23
5202.	SPATA31A1	SPATA31 Subfamily A Member 1
5203.	VCF2	VCP Nuclear Cofactor Family Member 2
5204.	CCDC177	Coiled-Coil Domain Containing 177
5205.	SSX2B	SSX Family Member 2B
5206.	TRIM39-RPP21	TRIM39-RPP21 Readthrough
5207.	TSPY3	Testis Specific Protein Y-Linked 3
5208.	SAA2-SAA4	SAA2-SAA4 Readthrough
5209.	NOTCH2NLB	Notch 2 N-Terminal Like B
5210.	IGKV1-33	Immunoglobulin Kappa Variable 1-33
5211.	LUZP6	Leucine Zipper Protein 6
5212.	PDXK	Pyridoxal Kinase
5213.	CRISP3	Cysteine Rich Secretory Protein 3
5214.	CD300A	CD300a Molecule
5215.	MAP4	Microtubule Associated Protein 4
5216.	GTF3A	General Transcription Factor IIIA
5217.	GRIN1	Glutamate Ionotropic Receptor NMDA Type Subunit 1
5218.	PRKCG	Protein Kinase C Gamma
5219.	TUBB3	Tubulin Beta 3 Class III
5220.	BUB1B	BUB1 Mitotic Checkpoint Serine/Threonine Kinase B
5221.	CSNK1D	Casein Kinase 1 Delta
5222.	AQP5	Aquaporin 5
5223.	CALCR	Calcitonin Receptor
5224.	CDC25C	Cell Division Cycle 25C
5225.	CYP1B1	Cytochrome P450 Family 1 Subfamily B Member 1
5226.	GABRA2	Gamma-Aminobutyric Acid Type A Receptor Subunit Alpha2
5227.	GABRA5	Gamma-Aminobutyric Acid Type A Receptor Subunit Alpha5
5228.	GABRB2	Gamma-Aminobutyric Acid Type A Receptor Subunit Beta2
5229.	GPHN	Gephyrin
5230.	IMPDH1	Inosine Monophosphate Dehydrogenase 1
5231.	MASP1	MBL Associated Serine Protease 1
5232.	AMHR2	Anti-Mullerian Hormone Receptor Type 2
5233.	CDH5	Cadherin 5
5234.	FGB	Fibrinogen Beta Chain
5235.	FLNB	Filamin B
5236.	GABRB1	Gamma-Aminobutyric Acid Type A Receptor Subunit Beta1
5237.	MAN1B1	Mannosidase Alpha Class 1B Member 1
5238.	PEBP1	Phosphatidylethanolamine Binding Protein 1
5239.	PLCB3	Phospholipase C Beta 3
5240.	POLB	DNA Polymerase Beta
5241.	PTPN2	Protein Tyrosine Phosphatase Non-Receptor Type 2

5242.	SLC4A4	Solute Carrier Family 4 Member 4
5243.	THBS2	Thrombospondin 2
5244.	ADCYAP1R1	ADCYAP Receptor Type I
5245.	AKR1C2	Aldo-Keto Reductase Family 1 Member C2
5246.	CACNA1I	Calcium Voltage-Gated Channel Subunit Alpha1 I
5247.	CALCRL	Calcitonin Receptor Like Receptor
5248.	CHRNA5	Cholinergic Receptor Nicotinic Alpha 5 Subunit
5249.	CLCN3	Chloride Voltage-Gated Channel 3
5250.	DLK1	Delta Like Non-Canonical Notch Ligand 1
5251.	ESRRA	Estrogen Related Receptor Alpha
5252.	GUCY2D	Guanylate Cyclase 2D, Retinal
5253.	HMOX2	Heme Oxygenase 2
5254.	KDM4B	Lysine Demethylase 4B
5255.	NT5C2	5'-Nucleotidase, Cytosolic II
5256.	PANX1	Pannexin 1
5257.	RASGRP1	RAS Guanyl Releasing Protein 1
5258.	SLC26A3	Solute Carrier Family 26 Member 3
5259.	TGFBI	Transforming Growth Factor Beta Induced
5260.	ABCA4	ATP Binding Cassette Subfamily A Member 4
5261.	CEACAM1	CEA Cell Adhesion Molecule 1
5262.	CHD2	Chromodomain Helicase DNA Binding Protein 2
5263.	CHRNB4	Cholinergic Receptor Nicotinic Beta 4 Subunit
5264.	COX4I1	Cytochrome C Oxidase Subunit 4I1
5265.	DCLRE1C	DNA Cross-Link Repair 1C
5266.	EIF2AK1	Eukaryotic Translation Initiation Factor 2 Alpha Kinase 1
5267.	IRF9	Interferon Regulatory Factor 9
5268.	ME2	Malic Enzyme 2
5269.	MEF2D	Myocyte Enhancer Factor 2D
5270.	MYO9B	Myosin IXB
5271.	PCBD1	Pterin-4 Alpha-Carbinolamine Dehydratase 1
5272.	PDE6A	Phosphodiesterase 6A
5273.	RDH12	Retinol Dehydrogenase 12
5274.	RPE65	Retinoid Isomerohydrolase RPE65
5275.	VIPR2	Vasoactive Intestinal Peptide Receptor 2
5276.	ADAM15	ADAM Metallopeptidase Domain 15
5277.	ADGRE5	Adhesion G Protein-Coupled Receptor E5
5278.	ATP2B4	ATPase Plasma Membrane Ca2+ Transporting 4
5279.	ATP6V1B1	ATPase H+ Transporting V1 Subunit B1
5280.	C1QB	Complement C1q B Chain
5281.	CPA6	Carboxypeptidase A6
5282.	DUOX2	Dual Oxidase 2

5283.	GABRA6	Gamma-Aminobutyric Acid Type A Receptor Subunit Alpha6
5284.	GAS6	Growth Arrest Specific 6
5285.	HGD	Homogentisate 1,2-Dioxygenase
5286.	ING1	Inhibitor Of Growth Family Member 1
5287.	MMP10	Matrix Metallopeptidase 10
5288.	NUP107	Nucleoporin 107
5289.	P2RX2	Purinergic Receptor P2X 2
5290.	PNPLA6	Patatin Like Phospholipase Domain Containing 6
5291.	PPP1R1B	Protein Phosphatase 1 Regulatory Inhibitor Subunit 1B
5292.	PRKAR2A	Protein Kinase CAMP-Dependent Type II Regulatory Subunit Alpha
5293.	RACK1	Receptor For Activated C Kinase 1
5294.	RPL10	Ribosomal Protein L10
5295.	SLC25A19	Solute Carrier Family 25 Member 19
5296.	SMS	Spermine Synthase
5297.	SOX6	SRY-Box Transcription Factor 6
5298.	SREBF2	Sterol Regulatory Element Binding Transcription Factor 2
5299.	UQCRC1	Ubiquinol-Cytochrome C Reductase Core Protein 1
5300.	WARS2	Tryptophanyl TRNA Synthetase 2, Mitochondrial
5301.	ADGRG1	Adhesion G Protein-Coupled Receptor G1
5302.	ATG16L1	Autophagy Related 16 Like 1
5303.	BMP15	Bone Morphogenetic Protein 15
5304.	CAMK1D	Calcium/Calmodulin Dependent Protein Kinase ID
5305.	CIB1	Calcium And Integrin Binding 1
5306.	CLDN18	Claudin 18
5307.	COX6B1	Cytochrome C Oxidase Subunit 6B1
5308.	ELAC2	ElaC Ribonuclease Z 2
5309.	EPM2A	EPM2A Glucan Phosphatase, Laforin
5310.	FABP4	Fatty Acid Binding Protein 4
5311.	HAGH	Hydroxyacylglutathione Hydrolase
5312.	HCRTR1	Hypocretin Receptor 1
5313.	HIRA	Histone Cell Cycle Regulator
5314.	HTR1F	5-Hydroxytryptamine Receptor 1F
5315.	JUND	JunD Proto-Oncogene, AP-1 Transcription Factor Subunit
5316.	MACROH2A1	MacroH2A.1 Histone
5317.	PRSS3	Serine Protease 3
5318.	PTPRG	Protein Tyrosine Phosphatase Receptor Type G
5319.	RAMP1	Receptor Activity Modifying Protein 1
5320.	RAMP2	Receptor Activity Modifying Protein 2
5321.	RFC4	Replication Factor C Subunit 4
5322.	SEC23A	SEC23 Homolog A, COPII Coat Complex Component
5323.	SLC12A7	Solute Carrier Family 12 Member 7

5324.	SPTBN2	Spectrin Beta, Non-Erythrocytic 2
5325.	SUV39H1	SUV39H1 Histone Lysine Methyltransferase
5326.	VARS1	Valyl-TRNA Synthetase 1
5327.	ADGRL1	Adhesion G Protein-Coupled Receptor L1
5328.	AIPL1	Aryl Hydrocarbon Receptor Interacting Protein Like 1
5329.	ATP6V0A4	ATPase H+ Transporting V0 Subunit A4
5330.	CLCN4	Chloride Voltage-Gated Channel 4
5331.	COX15	Cytochrome C Oxidase Assembly Homolog COX15
5332.	COX4I2	Cytochrome C Oxidase Subunit 4I2
5333.	CRX	Cone-Rod Homeobox
5334.	DPF2	Double PHD Fingers 2
5335.	FOXL2	Forkhead Box L2
5336.	GNL3	G Protein Nucleolar 3
5337.	IARS2	Isoleucyl-TRNA Synthetase 2, Mitochondrial
5338.	ID1	Inhibitor Of DNA Binding 1
5339.	KIF1C	Kinesin Family Member 1C
5340.	MPDU1	Mannose-P-Dolichol Utilization Defect 1
5341.	MRPS22	Mitochondrial Ribosomal Protein S22
5342.	NPPC	Natriuretic Peptide C
5343.	PLAGL1	PLAG1 Like Zinc Finger 1
5344.	PLIN2	Perilipin 2
5345.	POLR1D	RNA Polymerase I And III Subunit D
5346.	PRDM2	PR/SET Domain 2
5347.	PRKX	Protein Kinase CAMP-Dependent X-Linked Catalytic Subunit
5348.	RARS2	Arginyl-TRNA Synthetase 2, Mitochondrial
5349.	RPL10A	Ribosomal Protein L10a
5350.	TCOF1	Treacle Ribosome Biogenesis Factor 1
5351.	TULP1	TUB Like Protein 1
5352.	VARS2	Valyl-TRNA Synthetase 2, Mitochondrial
5353.	WRAP53	WD Repeat Containing Antisense To TP53
5354.	AKAP1	A-Kinase Anchoring Protein 1
5355.	CAPN10	Calpain 10
5356.	CCL21	C-C Motif Chemokine Ligand 21
5357.	CCN1	Cellular Communication Network Factor 1
5358.	DKK3	Dickkopf WNT Signaling Pathway Inhibitor 3
5359.	ELP4	Elongator Acetyltransferase Complex Subunit 4
5360.	HHEX	Hematopoietically Expressed Homeobox
5361.	HNF4G	Hepatocyte Nuclear Factor 4 Gamma
5362.	KCNV2	Potassium Voltage-Gated Channel Modifier Subfamily V Member 2
5363.	MT2A	Metallothionein 2A
5364.	ONECUT1	One Cut Homeobox 1

5365.	PDLIM1	PDZ And LIM Domain 1
5366.	RAMP3	Receptor Activity Modifying Protein 3
5367.	RBFOX1	RNA Binding Fox-1 Homolog 1
5368.	RPL3	Ribosomal Protein L3
5369.	SSBP1	Single Stranded DNA Binding Protein 1
5370.	ZBTB17	Zinc Finger And BTB Domain Containing 17
5371.	AMY2B	Amylase Alpha 2B
5372.	ATAD1	ATPase Family AAA Domain Containing 1
5373.	CABIN1	Calcineurin Binding Protein 1
5374.	CLDN10	Claudin 10
5375.	CLDN19	Claudin 19
5376.	COX8A	Cytochrome C Oxidase Subunit 8A
5377.	DLX4	Distal-Less Homeobox 4
5378.	DMAP1	DNA Methyltransferase 1 Associated Protein 1
5379.	GCKR	Glucokinase Regulator
5380.	GLRX3	Glutaredoxin 3
5381.	KCNK10	Potassium Two Pore Domain Channel Subfamily K Member 10
5382.	KCNK2	Potassium Two Pore Domain Channel Subfamily K Member 2
5383.	KIF21A	Kinesin Family Member 21A
5384.	NFE2	Nuclear Factor, Erythroid 2
5385.	NPRL2	NPR2 Like, GATOR1 Complex Subunit
5386.	OPN4	Opsin 4
5387.	OTUD7A	OTU Deubiquitinase 7A
5388.	PAXIP1	PAX Interacting Protein 1
5389.	RAD51B	RAD51 Paralog B
5390.	RCVRN	Recoverin
5391.	REG4	Regenerating Family Member 4
5392.	RHCE	Rh Blood Group CcEe Antigens
5393.	SMARCA1	SWI/SNF Related, Matrix Associated, Actin Dependent Regulator Of Chromatin, Subfamily A, Member 1
5394.	SUGCT	Succinyl-CoA:Glutarate-CoA Transferase
5395.	TBC1D24	TBC1 Domain Family Member 24
5396.	TBL1X	Transducin Beta Like 1 X-Linked
5397.	TFB1M	Transcription Factor B1, Mitochondrial
5398.	TMEM126A	Transmembrane Protein 126A
5399.	TRIM22	Tripartite Motif Containing 22
5400.	UIMC1	Ubiquitin Interaction Motif Containing 1
5401.	ADGRB2	Adhesion G Protein-Coupled Receptor B2
5402.	ADGRF5	Adhesion G Protein-Coupled Receptor F5
5403.	CEP104	Centrosomal Protein 104
5404.	CLN6	CLN6 Transmembrane ER Protein
5405.	CRIPT	CXXC Repeat Containing Interactor Of PDZ3 Domain

5406.	CRYBB1	Crystallin Beta B1
5407.	CTF1	Cardiotrophin 1
5408.	DYNC2H1	Dynein Cytoplasmic 2 Heavy Chain 1
5409.	ECSIT	ECSIT Signaling Integrator
5410.	EMG1	EMG1 N1-Specific Pseudouridine Methyltransferase
5411.	ESCO2	Establishment Of Sister Chromatid Cohesion N-Acetyltransferase 2
5412.	FASTKD2	FAST Kinase Domains 2
5413.	GAR1	GAR1 Ribonucleoprotein
5414.	H2AZ1	H2A.Z Variant Histone 1
5415.	LACTB	Lactamase Beta
5416.	LCA5	Lebercilin LCA5
5417.	LGI4	Leucine Rich Repeat LGI Family Member 4
5418.	LY9	Lymphocyte Antigen 9
5419.	MBOAT7	Membrane Bound O-Acyltransferase Domain Containing 7
5420.	NAPSA	Napsin A Aspartic Peptidase
5421.	PDLIM5	PDZ And LIM Domain 5
5422.	PEMT	Phosphatidylethanolamine N-Methyltransferase
5423.	PES1	Pescadillo Ribosomal Biogenesis Factor 1
5424.	PIR	Pirin
5425.	PPP1R14A	Protein Phosphatase 1 Regulatory Inhibitor Subunit 14A
5426.	RECQL5	RecQ Like Helicase 5
5427.	RPS11	Ribosomal Protein S11
5428.	SLAMF6	SLAM Family Member 6
5429.	SLC24A3	Solute Carrier Family 24 Member 3
5430.	TKTL1	Transketolase Like 1
5431.	TOP3B	DNA Topoisomerase III Beta
5432.	TSR1	TSR1 Ribosome Maturation Factor
5433.	USH2A	Usherin
5434.	VRK2	VRK Serine/Threonine Kinase 2
5435.	ASF1A	Anti-Silencing Function 1A Histone Chaperone
5436.	ATOH1	Atonal BHLH Transcription Factor 1
5437.	CALCB	Calcitonin Related Polypeptide Beta
5438.	DR1	Down-Regulator Of Transcription 1
5439.	FTMT	Ferritin Mitochondrial
5440.	GLIS3	GLIS Family Zinc Finger 3
5441.	LUC7L	LUC7 Like
5442.	MYCL	MYCL Proto-Oncogene, BHLH Transcription Factor
5443.	NEIL3	Nei Like DNA Glycosylase 3
5444.	PCDH1	Protocadherin 1
5445.	PCDH7	Protocadherin 7
5446.	PHACTR1	Phosphatase And Actin Regulator 1

5447.	PUS3	Pseudouridine Synthase 3
5448.	RPL29	Ribosomal Protein L29
5449.	RSL24D1	Ribosomal L24 Domain Containing 1
5450.	TSPAN2	Tetraspanin 2
5451.	UBN1	Ubinuclein 1
5452.	UGT3A1	UDP Glycosyltransferase Family 3 Member A1
5453.	UGT3A2	UDP Glycosyltransferase Family 3 Member A2
5454.	ADGRG3	Adhesion G Protein-Coupled Receptor G3
5455.	APLN	Apelin
5456.	ARHGEF5	Rho Guanine Nucleotide Exchange Factor 5
5457.	B4GAT1	Beta-1,4-Glucuronyltransferase 1
5458.	CALR3	Calreticulin 3
5459.	CCDC90B	Coiled-Coil Domain Containing 90B
5460.	COX6A2	Cytochrome C Oxidase Subunit 6A2
5461.	DAP	Death Associated Protein
5462.	DCLRE1B	DNA Cross-Link Repair 1B
5463.	ESCO1	Establishment Of Sister Chromatid Cohesion N-Acetyltransferase 1
5464.	FBXO9	F-Box Protein 9
5465.	GLCE	Glucuronic Acid Epimerase
5466.	HRC	Histidine Rich Calcium Binding Protein
5467.	MAGEA3	MAGE Family Member A3
5468.	MRM2	Mitochondrial RRNA Methyltransferase 2
5469.	MTMR10	Myotubularin Related Protein 10
5470.	POC1B	POC1 Centriolar Protein B
5471.	QTRT1	Queuine TRNA-Ribosyltransferase Catalytic Subunit 1
5472.	RBM6	RNA Binding Motif Protein 6
5473.	RD3	RD3 Regulator Of GUCY2D
5474.	RPL36	Ribosomal Protein L36
5475.	RPL38	Ribosomal Protein L38
5476.	CDK5RAP1	CDK5 Regulatory Subunit Associated Protein 1
5477.	NMD3	NMD3 Ribosome Export Adaptor
5478.	OSGEPL1	O-Sialoglycoprotein Endopeptidase Like 1
5479.	PIF1	PIF1 5'-To-3' DNA Helicase
5480.	RPS25	Ribosomal Protein S25
5481.	SERPINA11	Serpin Family A Member 11
5482.	SKOR1	SKI Family Transcriptional Corepressor 1
5483.	SOHLH1	Spermatogenesis And Oogenesis Specific Basic Helix-Loop-Helix 1
5484.	TMEM87B	Transmembrane Protein 87B
5485.	TPSD1	Tryptase Delta 1
5486.	TXNRD3	Thioredoxin Reductase 3
5487.	VSIG4	V-Set And Immunoglobulin Domain Containing 4

5488.	ADGRG5	Adhesion G Protein-Coupled Receptor G5
5489.	AGBL3	AGBL Carboxypeptidase 3
5490.	CEP89	Centrosomal Protein 89
5491.	DIRAS1	DIRAS Family GTPase 1
5492.	EFHC2	EF-Hand Domain Containing 2
5493.	EYS	Eyes Shut Homolog
5494.	FAM161A	FAM161 Centrosomal Protein A
5495.	FBH1	F-Box DNA Helicase 1
5496.	KRT76	Keratin 76
5497.	PMCH	Pro-Melanin Concentrating Hormone
5498.	RMI2	RecQ Mediated Genome Instability 2
5499.	TBRG1	Transforming Growth Factor Beta Regulator 1
5500.	ADGRF1	Adhesion G Protein-Coupled Receptor F1
5501.	ADM2	Adrenomedullin 2
5502.	DHFR2	Dihydrofolate Reductase 2
5503.	NMRAL1	NmrA Like Redox Sensor 1
5504.	NOL9	Nucleolar Protein 9
5505.	CAMSAP2	Calmodulin Regulated Spectrin Associated Protein Family Member 2
5506.	CFAP418	Cilia And Flagella Associated Protein 418
5507.	COA3	Cytochrome C Oxidase Assembly Factor 3
5508.	FAM234A	Family With Sequence Similarity 234 Member A
5509.	LIPT2	Lipoyl(Octanoyl) Transferase 2
5510.	SPRY3	Sprouty RTK Signaling Antagonist 3
5511.	ZBED3	Zinc Finger BED-Type Containing 3
5512.	CDC42SE1	CDC42 Small Effector 1
5513.	H3C14	H3 Clustered Histone 14
5514.	POP7	POP7 Homolog, Ribonuclease P/MRP Subunit
5515.	TRMT61B	TRNA Methyltransferase 61B
5516.	DHRS13	Dehydrogenase/Reductase 13
5517.	FIBIN	Fin Bud Initiation Factor Homolog
5518.	TAS2R14	Taste 2 Receptor Member 14
5519.	ANKDD1B	Ankyrin Repeat And Death Domain Containing 1B
5520.	C22orf39	Chromosome 22 Open Reading Frame 39
5521.	FAM216A	Family With Sequence Similarity 216 Member A
5522.	GYPE	Glycophorin E (MNS Blood Group)
5523.	HBM	Hemoglobin Subunit Mu
5524.	TAS2R4	Taste 2 Receptor Member 4
5525.	OR10S1	Olfactory Receptor Family 10 Subfamily S Member 1
5526.	CENPX	Centromere Protein X
5527.	FAAP20	FA Core Complex Associated Protein 20
5528.	TASL	TLR Adaptor Interacting With Endolysosomal SLC15A4

5529.	SERPINA2	Serpin Family A Member 2 (Gene/Pseudogene)
5530.	MYMX	Myomixer, Myoblast Fusion Factor
5531.	H3Y1	H3.Y Histone 1
5532.	PROS1	Protein S
5533.	PRKAG3	Protein Kinase AMP-Activated Non-Catalytic Subunit Gamma 3
5534.	GP6	Glycoprotein VI Platelet
5535.	LONP1	Lon Peptidase 1, Mitochondrial
5536.	ALDH1A3	Aldehyde Dehydrogenase 1 Family Member A3
5537.	GDF6	Growth Differentiation Factor 6
5538.	PORCN	Porcupine O-Acyltransferase
5539.	STRA6	Signaling Receptor And Transporter Of Retinol STRA6
5540.	CRYBA4	Crystallin Beta A4
5541.	VSX2	Visual System Homeobox 2
5542.	GDF3	Growth Differentiation Factor 3
5543.	DACH1	Dachshund Family Transcription Factor 1
5544.	NHS	NHS Actin Remodeling Regulator
5545.	AP4E1	Adaptor Related Protein Complex 4 Subunit Epsilon 1
5546.	C12orf57	Chromosome 12 Open Reading Frame 57
5547.	MRPS25	Mitochondrial Ribosomal Protein S25
5548.	RAX	Retina And Anterior Neural Fold Homeobox
5549.	SNAPC4	Small Nuclear RNA Activating Complex Polypeptide 4
5550.	TENM3	Teneurin Transmembrane Protein 3
5551.	GATD3	Glutamine Amidotransferase Class 1 Domain Containing 3
5552.	MLLT11	MLLT11 Transcription Factor 7 Cofactor
5553.	MEA1	Male-Enhanced Antigen 1
5554.	GIMAP1-GIMAP5	GIMAP1-GIMAP5 Readthrough
5555.	GTF2IRD1	GTF2I Repeat Domain Containing 1
5556.	MRPL44	Mitochondrial Ribosomal Protein L44
5557.	TBXA2R	Thromboxane A2 Receptor
5558.	TUBB4A	Tubulin Beta 4A Class IVa
5559.	ITCH	Itchy E3 Ubiquitin Protein Ligase
5560.	SLC6A8	Solute Carrier Family 6 Member 8
5561.	ECHS1	Enoyl-CoA Hydratase, Short Chain 1
5562.	ARHGDIA	Rho GDP Dissociation Inhibitor Alpha
5563.	PDHB	Pyruvate Dehydrogenase E1 Subunit Beta
5564.	CCT5	Chaperonin Containing TCP1 Subunit 5
5565.	ENTPD3	Ectonucleoside Triphosphate Diphosphohydrolase 3
5566.	FCER1A	Fc Epsilon Receptor Ia
5567.	LTC4S	Leukotriene C4 Synthase
5568.	MRAS	Muscle RAS Oncogene Homolog
5569.	PDHA2	Pyruvate Dehydrogenase E1 Subunit Alpha 2

5570.	SOS2	SOS Ras/Rho Guanine Nucleotide Exchange Factor 2
5571.	AHCYL1	Adenosylhomocysteinase Like 1
5572.	ASPA	Aspartoacylase
5573.	CHD3	Chromodomain Helicase DNA Binding Protein 3
5574.	CYFIP2	Cytoplasmic FMR1 Interacting Protein 2
5575.	ENPP3	Ectonucleotide Pyrophosphatase/Phosphodiesterase 3
5576.	HOMER1	Homer Scaffold Protein 1
5577.	NDST1	N-Deacetylase And N-Sulfotransferase 1
5578.	NFU1	NFU1 Iron-Sulfur Cluster Scaffold
5579.	PAPOLA	Poly(A) Polymerase Alpha
5580.	PMPCB	Peptidase, Mitochondrial Processing Subunit Beta
5581.	RCC1	Regulator Of Chromosome Condensation 1
5582.	ATP2C2	ATPase Secretory Pathway Ca2+ Transporting 2
5583.	CERT1	Ceramide Transporter 1
5584.	FOXN1	Forkhead Box N1
5585.	GJC2	Gap Junction Protein Gamma 2
5586.	HS3ST1	Heparan Sulfate-Glucosamine 3-Sulfotransferase 1
5587.	PCSK5	Proprotein Convertase Subtilisin/Kexin Type 5
5588.	PDCD10	Programmed Cell Death 10
5589.	ANO6	Anoctamin 6
5590.	HEPACAM	Hepatic And Glial Cell Adhesion Molecule
5591.	LIPT1	Lipoyltransferase 1
5592.	TECR	Trans-2,3-Enoyl-CoA Reductase
5593.	USP16	Ubiquitin Specific Peptidase 16
5594.	ALG3	ALG3 Alpha-1,3- Mannosyltransferase
5595.	AP4B1	Adaptor Related Protein Complex 4 Subunit Beta 1
5596.	FCER1G	Fc Epsilon Receptor Ig
5597.	KIAA0319	KIAA0319
5598.	LGR6	Leucine Rich Repeat Containing G Protein-Coupled Receptor 6
5599.	PI4K2B	Phosphatidylinositol 4-Kinase Type 2 Beta
5600.	PIP4K2C	Phosphatidylinositol-5-Phosphate 4-Kinase Type 2 Gamma
5601.	TMSB4X	Thymosin Beta 4 X-Linked
5602.	AP4M1	Adaptor Related Protein Complex 4 Subunit Mu 1
5603.	ARFGEF1	ADP Ribosylation Factor Guanine Nucleotide Exchange Factor 1
5604.	CCDC88C	Coiled-Coil Domain Containing 88C
5605.	CHAMP1	Chromosome Alignment Maintaining Phosphoprotein 1
5606.	CYP3A7	Cytochrome P450 Family 3 Subfamily A Member 7
5607.	HS3ST3B1	Heparan Sulfate-Glucosamine 3-Sulfotransferase 3B1
5608.	IMMP2L	Inner Mitochondrial Membrane Peptidase Subunit 2
5609.	KANSL1	KAT8 Regulatory NSL Complex Subunit 1
5610.	PAPOLG	Poly(A) Polymerase Gamma

5611.	RASA2	RAS P21 Protein Activator 2
5612.	RBFOX2	RNA Binding Fox-1 Homolog 2
5613.	RHCG	Rh Family C Glycoprotein
5614.	U2AF2	U2 Small Nuclear RNA Auxiliary Factor 2
5615.	CLEC1B	C-Type Lectin Domain Family 1 Member B
5616.	HS3ST2	Heparan Sulfate-Glucosamine 3-Sulfotransferase 2
5617.	MTMR7	Myotubularin Related Protein 7
5618.	RABEP1	Rabaptin, RAB GTPase Binding Effector Protein 1
5619.	SERPINB10	Serpin Family B Member 10
5620.	TCHH	Trichohyalin
5621.	THSD7A	Thrombospondin Type 1 Domain Containing 7A
5622.	ADPRH	ADP-Ribosylarginine Hydrolase
5623.	CCL13	C-C Motif Chemokine Ligand 13
5624.	CELF5	CUGBP Elav-Like Family Member 5
5625.	CFAP53	Cilia And Flagella Associated Protein 53
5626.	CPNE4	Copine 4
5627.	FLG2	Filaggrin 2
5628.	HS3ST6	Heparan Sulfate-Glucosamine 3-Sulfotransferase 6
5629.	MYO10	Myosin X
5630.	NELFCD	Negative Elongation Factor Complex Member C/D
5631.	NHERF2	NHERF Family PDZ Scaffold Protein 2
5632.	PLEKHB2	Pleckstrin Homology Domain Containing B2
5633.	RHBG	Rh Family B Glycoprotein
5634.	CCN5	Cellular Communication Network Factor 5
5635.	DDX47	DEAD-Box Helicase 47
5636.	EFHD2	EF-Hand Domain Family Member D2
5637.	LHX6	LIM Homeobox 6
5638.	SLBP	Stem-Loop Histone MRNA Binding Protein
5639.	SLC9A5	Solute Carrier Family 9 Member A5
5640.	TMED2	Transmembrane P24 Trafficking Protein 2
5641.	AGO4	Argonaute RISC Component 4
5642.	CMIP	C-Maf Inducing Protein
5643.	DUS4L	Dihydrouridine Synthase 4 Like
5644.	HS3ST3A1	Heparan Sulfate-Glucosamine 3-Sulfotransferase 3A1
5645.	HTR3C	5-Hydroxytryptamine Receptor 3C
5646.	MPP3	MAGUK P55 Scaffold Protein 3
5647.	SAMD4B	Sterile Alpha Motif Domain Containing 4B
5648.	SYPL1	Synaptophysin Like 1
5649.	ZMYM4	Zinc Finger MYM-Type Containing 4
5650.	CYBC1	Cytochrome B-245 Chaperone 1
5651.	MED20	Mediator Complex Subunit 20

5652.	PAPOLB	Poly(A) Polymerase Beta
5653.	PCMTD2	Protein-L-Isoaspartate (D-Aspartate) O-Methyltransferase Domain Containing 2
5654.	STOML1	Stomatin Like 1
5655.	ARMC3	Armadillo Repeat Containing 3
5656.	PTRHD1	Peptidyl-TRNA Hydrolase Domain Containing 1
5657.	VSTM1	V-Set And Transmembrane Domain Containing 1
5658.	BATF2	Basic Leucine Zipper ATF-Like Transcription Factor 2
5659.	CDR2L	Cerebellar Degeneration Related Protein 2 Like
5660.	DNAJC22	DnaJ Heat Shock Protein Family (Hsp40) Member C22
5661.	LRCH2	Leucine Rich Repeats And Calponin Homology Domain Containing 2
5662.	MTCL2	Microtubule Crosslinking Factor 2
5663.	TARM1	T Cell-Interacting, Activating Receptor On Myeloid Cells 1
5664.	TTC32	Tetratricopeptide Repeat Domain 32
5665.	VPS37D	VPS37D Subunit Of ESCRT-I
5666.	CHP2	Calcineurin Like EF-Hand Protein 2
5667.	ERICH6	Glutamate Rich 6
5668.	NNAT	Neuronatin
5669.	PET100	PET100 Cytochrome C Oxidase Chaperone
5670.	FDCSP	Follicular Dendritic Cell Secreted Protein
5671.	SCIMP	SLP Adaptor And CSK Interacting Membrane Protein
5672.	TYW1B	TRNA-YW Synthesizing Protein 1 Homolog B
5673.	PAK1	P21 (RAC1) Activated Kinase 1
5674.	DDR2	Discoidin Domain Receptor Tyrosine Kinase 2
5675.	HK1	Hexokinase 1
5676.	NEK2	NIMA Related Kinase 2
5677.	ABCA1	ATP Binding Cassette Subfamily A Member 1
5678.	CSNK1A1	Casein Kinase 1 Alpha 1
5679.	ECE1	Endothelin Converting Enzyme 1
5680.	PTPRF	Protein Tyrosine Phosphatase Receptor Type F
5681.	TRPC6	Transient Receptor Potential Cation Channel Subfamily C Member 6
5682.	NRXN1	Neurexin 1
5683.	UBE3A	Ubiquitin Protein Ligase E3A
5684.	USP7	Ubiquitin Specific Peptidase 7
5685.	ACAT1	Acetyl-CoA Acetyltransferase 1
5686.	ACTN1	Actinin Alpha 1
5687.	ADAM9	ADAM Metallopeptidase Domain 9
5688.	ADCY1	Adenylate Cyclase 1
5689.	ANGPTL3	Angiopoietin Like 3
5690.	GRK2	G Protein-Coupled Receptor Kinase 2
5691.	INPPL1	Inositol Polyphosphate Phosphatase Like 1
5692.	KIF11	Kinesin Family Member 11

5693.	MAPKAPK3	MAPK Activated Protein Kinase 3
5694.	OAT	Ornithine Aminotransferase
5695.	PCK1	Phosphoenolpyruvate Carboxykinase 1
5696.	PRKAB1	Protein Kinase AMP-Activated Non-Catalytic Subunit Beta 1
5697.	PRKCE	Protein Kinase C Epsilon
5698.	ST14	ST14 Transmembrane Serine Protease Matriptase
5699.	VRK1	VRK Serine/Threonine Kinase 1
5700.	A2M	Alpha-2-Macroglobulin
5701.	ACTN4	Actinin Alpha 4
5702.	ASNS	Asparagine Synthetase (Glutamine-Hydrolyzing)
5703.	CAMK2D	Calcium/Calmodulin Dependent Protein Kinase II Delta
5704.	CDC45	Cell Division Cycle 45
5705.	CYBA	Cytochrome B-245 Alpha Chain
5706.	DVL1	Dishevelled Segment Polarity Protein 1
5707.	ENPEP	Glutamyl Aminopeptidase
5708.	FLI1	Fli-1 Proto-Oncogene, ETS Transcription Factor
5709.	GLDC	Glycine Decarboxylase
5710.	HCFC1	Host Cell Factor C1
5711.	ITPA	Inosine Triphosphatase
5712.	NPR1	Natriuretic Peptide Receptor 1
5713.	NR1H3	Nuclear Receptor Subfamily 1 Group H Member 3
5714.	NR4A1	Nuclear Receptor Subfamily 4 Group A Member 1
5715.	OGT	O-Linked N-Acetylglucosamine (GlcNAc) Transferase
5716.	PAFAH1B1	Platelet Activating Factor Acetylhydrolase 1b Regulatory Subunit 1
5717.	PDK1	Pyruvate Dehydrogenase Kinase 1
5718.	STS	Steroid Sulfatase
5719.	TRPV6	Transient Receptor Potential Cation Channel Subfamily V Member 6
5720.	WNT3	Wnt Family Member 3
5721.	WNT4	Wnt Family Member 4
5722.	AK1	Adenylate Kinase 1
5723.	AMPD2	Adenosine Monophosphate Deaminase 2
5724.	ATP5F1A	ATP Synthase F1 Subunit Alpha
5725.	CDC25B	Cell Division Cycle 25B
5726.	CDC6	Cell Division Cycle 6
5727.	DDX5	DEAD-Box Helicase 5
5728.	DYRK1B	Dual Specificity Tyrosine Phosphorylation Regulated Kinase 1B
5729.	EDN3	Endothelin 3
5730.	EEF2K	Eukaryotic Elongation Factor 2 Kinase
5731.	EHMT1	Euchromatic Histone Lysine Methyltransferase 1
5732.	FAP	Fibroblast Activation Protein Alpha
5733.	FZD5	Frizzled Class Receptor 5

5734.	GLRA1	Glycine Receptor Alpha 1
5735.	GRK6	G Protein-Coupled Receptor Kinase 6
5736.	GSTM3	Glutathione S-Transferase Mu 3
5737.	HERC2	HECT And RLD Domain Containing E3 Ubiquitin Protein Ligase 2
5738.	HSD17B3	Hydroxysteroid 17-Beta Dehydrogenase 3
5739.	KCNC1	Potassium Voltage-Gated Channel Subfamily C Member 1
5740.	LAMA1	Laminin Subunit Alpha 1
5741.	LAMB3	Laminin Subunit Beta 3
5742.	LNPEP	Leucyl And Cystinyl Aminopeptidase
5743.	MCM3	Minichromosome Maintenance Complex Component 3
5744.	MSX1	Msh Homeobox 1
5745.	NLGN1	Neuroligin 1
5746.	PARP2	Poly(ADP-Ribose) Polymerase 2
5747.	PRKCI	Protein Kinase C Iota
5748.	PTGDS	Prostaglandin D2 Synthase
5749.	PTGIR	Prostaglandin I2 Receptor
5750.	RPS6KB2	Ribosomal Protein S6 Kinase B2
5751.	SAT1	Spermidine/Spermine N1-Acetyltransferase 1
5752.	SATB2	SATB Homeobox 2
5753.	SLC9A6	Solute Carrier Family 9 Member A6
5754.	ST3GAL3	ST3 Beta-Galactoside Alpha-2,3-Sialyltransferase 3
5755.	TFAP2B	Transcription Factor AP-2 Beta
5756.	YWHAZ	Tyrosine 3-Monooxygenase/Tryptophan 5-Monooxygenase Activation Protein Zeta
5757.	ACSL1	Acyl-CoA Synthetase Long Chain Family Member 1
5758.	ALOX5AP	Arachidonate 5-Lipoxygenase Activating Protein
5759.	AQP3	Aquaporin 3 (Gill Blood Group)
5760.	ATF3	Activating Transcription Factor 3
5761.	ATP2B2	ATPase Plasma Membrane Ca2+ Transporting 2
5762.	ATP6AP1	ATPase H+ Transporting Accessory Protein 1
5763.	CDH13	Cadherin 13
5764.	CENPE	Centromere Protein E
5765.	CNP	2',3'-Cyclic Nucleotide 3' Phosphodiesterase
5766.	CTPS1	CTP Synthase 1
5767.	EIF4A3	Eukaryotic Translation Initiation Factor 4A3
5768.	FOXC2	Forkhead Box C2
5769.	FOXG1	Forkhead Box G1
5770.	GCLC	Glutamate-Cysteine Ligase Catalytic Subunit
5771.	GOT1	Glutamic-Oxaloacetic Transaminase 1
5772.	HAAO	3-Hydroxyanthranilate 3,4-Dioxygenase
5773.	HABP2	Hyaluronan Binding Protein 2
5774.	INHBA	Inhibin Subunit Beta A

5775.	KRT17	Keratin 17
5776.	LRPAP1	LDL Receptor Related Protein Associated Protein 1
5777.	MAD2L1	Mitotic Arrest Deficient 2 Like 1
5778.	MAT1A	Methionine Adenosyltransferase 1A
5779.	MED12	Mediator Complex Subunit 12
5780.	MYOCD	Myocardin
5781.	NCOA1	Nuclear Receptor Coactivator 1
5782.	NFS1	NFS1 Cysteine Desulfurase
5783.	NME2	NME/NM23 Nucleoside Diphosphate Kinase 2
5784.	PCNT	Pericentrin
5785.	PDE10A	Phosphodiesterase 10A
5786.	PDE2A	Phosphodiesterase 2A
5787.	PEX1	Peroxisomal Biogenesis Factor 1
5788.	PLTP	Phospholipid Transfer Protein
5789.	PRKD2	Protein Kinase D2
5790.	PTGIS	Prostaglandin I2 Synthase
5791.	RBL2	RB Transcriptional Corepressor Like 2
5792.	ROBO1	Roundabout Guidance Receptor 1
5793.	SALL1	Spalt Like Transcription Factor 1
5794.	SLC8A1	Solute Carrier Family 8 Member A1
5795.	SOAT1	Sterol O-Acyltransferase 1
5796.	SRPK1	SRSF Protein Kinase 1
5797.	STMN1	Stathmin 1
5798.	TCF12	Transcription Factor 12
5799.	TIAM1	TIAM Rac1 Associated GEF 1
5800.	USF1	Upstream Transcription Factor 1
5801.	WNT2B	Wnt Family Member 2B
5802.	ACY1	Aminoacylase 1
5803.	ADIPOR1	Adiponectin Receptor 1
5804.	AIMP2	Aminoacyl TRNA Synthetase Complex Interacting Multifunctional Protein 2
5805.	AKR1B10	Aldo-Keto Reductase Family 1 Member B10
5806.	AKR1C1	Aldo-Keto Reductase Family 1 Member C1
5807.	APOA5	Apolipoprotein A5
5808.	ARL3	ADP Ribosylation Factor Like GTPase 3
5809.	BAMBI	BMP And Activin Membrane Bound Inhibitor
5810.	BCL11B	BCL11 Transcription Factor B
5811.	BLVRA	Biliverdin Reductase A
5812.	C3AR1	Complement C3a Receptor 1
5813.	CBR1	Carbonyl Reductase 1
5814.	CCNB2	Cyclin B2
5815.	CLPB	ClpB Family Mitochondrial Disaggregase

5816.	CYC1	Cytochrome C1
5817.	CYP2R1	Cytochrome P450 Family 2 Subfamily R Member 1
5818.	DHPS	Deoxyhypusine Synthase
5819.	ESD	Esterase D
5820.	EYA1	EYA Transcriptional Coactivator And Phosphatase 1
5821.	F2RL3	F2R Like Thrombin Or Trypsin Receptor 3
5822.	FOXA1	Forkhead Box A1
5823.	FUT8	Fucosyltransferase 8
5824.	GALE	UDP-Galactose-4-Epimerase
5825.	GALR3	Galanin Receptor 3
5826.	GRB7	Growth Factor Receptor Bound Protein 7
5827.	HLCS	Holocarboxylase Synthetase
5828.	INHA	Inhibin Subunit Alpha
5829.	IQGAP1	IQ Motif Containing GTPase Activating Protein 1
5830.	KCND2	Potassium Voltage-Gated Channel Subfamily D Member 2
5831.	LBP	Lipopolysaccharide Binding Protein
5832.	LTBP3	Latent Transforming Growth Factor Beta Binding Protein 3
5833.	MAP3K2	Mitogen-Activated Protein Kinase Kinase Kinase 2
5834.	MCM6	Minichromosome Maintenance Complex Component 6
5835.	MGP	Matrix Gla Protein
5836.	MMAB	Metabolism Of Cobalamin Associated B
5837.	NDUFA12	NADH:Ubiquinone Oxidoreductase Subunit A12
5838.	NLGN4X	Neuroligin 4 X-Linked
5839.	NUP62	Nucleoporin 62
5840.	PAX9	Paired Box 9
5841.	PDCD4	Programmed Cell Death 4
5842.	PFKFB3	6-Phosphofructo-2-Kinase/Fructose-2,6-Biphosphatase 3
5843.	POLR3B	RNA Polymerase III Subunit B
5844.	PPP2R2A	Protein Phosphatase 2 Regulatory Subunit Balpha
5845.	PRKCSH	PRKCSH Beta Subunit Of Glucosidase II
5846.	PTPN13	Protein Tyrosine Phosphatase Non-Receptor Type 13
5847.	PTRH2	Peptidyl-TRNA Hydrolase 2
5848.	RCAN1	Regulator Of Calcineurin 1
5849.	SEC63	SEC63 Homolog, Protein Translocation Regulator
5850.	SIX1	SIX Homeobox 1
5851.	SKIC2	SKI2 Subunit Of Superkiller Complex
5852.	SKP1	S-Phase Kinase Associated Protein 1
5853.	SLC25A5	Solute Carrier Family 25 Member 5
5854.	SLC5A5	Solute Carrier Family 5 Member 5
5855.	SMURF1	SMAD Specific E3 Ubiquitin Protein Ligase 1
5856.	SOX4	SRY-Box Transcription Factor 4

5857.	STAG3	STAG3 Cohesin Complex Component
5858.	SUFU	SUFU Negative Regulator Of Hedgehog Signaling
5859.	TOP3A	DNA Topoisomerase III Alpha
5860.	TTBK2	Tau Tubulin Kinase 2
5861.	VANGL2	VANGL Planar Cell Polarity Protein 2
5862.	WIF1	WNT Inhibitory Factor 1
5863.	ADAMTS10	ADAM Metallopeptidase With Thrombospondin Type 1 Motif 10
5864.	AFF2	ALF Transcription Elongation Factor 2
5865.	ALCAM	Activated Leukocyte Cell Adhesion Molecule
5866.	APOD	Apolipoprotein D
5867.	AQP7	Aquaporin 7
5868.	ATN1	Atrophin 1
5869.	BACE2	Beta-Secretase 2
5870.	CACNA2D2	Calcium Voltage-Gated Channel Auxiliary Subunit Alpha2delta 2
5871.	CAPN5	Calpain 5
5872.	CDK10	Cyclin Dependent Kinase 10
5873.	CRYZ	Crystallin Zeta
5874.	CUL5	Cullin 5
5875.	DFFB	DNA Fragmentation Factor Subunit Beta
5876.	DGKA	Diacylglycerol Kinase Alpha
5877.	DYNLL1	Dynein Light Chain LC8-Type 1
5878.	EIF2B5	Eukaryotic Translation Initiation Factor 2B Subunit Epsilon
5879.	ERLIN1	ER Lipid Raft Associated 1
5880.	ERLIN2	ER Lipid Raft Associated 2
5881.	ETV4	ETS Variant Transcription Factor 4
5882.	FERMT3	FERM Domain Containing Kindlin 3
5883.	GNAL	G Protein Subunit Alpha L
5884.	GPLD1	Glycosylphosphatidylinositol Specific Phospholipase D1
5885.	GRB14	Growth Factor Receptor Bound Protein 14
5886.	IFT81	Intraflagellar Transport 81
5887.	KDM2B	Lysine Demethylase 2B
5888.	LAMC1	Laminin Subunit Gamma 1
5889.	LGMN	Legumain
5890.	LIPF	Lipase F, Gastric Type
5891.	LIPG	Lipase G, Endothelial Type
5892.	MAPKAPK5	MAPK Activated Protein Kinase 5
5893.	MDK	Midkine
5894.	NDUFA6	NADH:Ubiquinone Oxidoreductase Subunit A6
5895.	NDUFA8	NADH:Ubiquinone Oxidoreductase Subunit A8
5896.	NID1	Nidogen 1
5897.	NPTX2	Neuronal Pentraxin 2

5898.	P2RX1	Purinergic Receptor P2X 1
5899.	P2RY11	Purinergic Receptor P2Y11
5900.	PADI2	Peptidyl Arginine Deiminase 2
5901.	PCDH15	Protocadherin Related 15
5902.	PDE6D	Phosphodiesterase 6D
5903.	PGC	Progastricsin
5904.	PIP5K1B	Phosphatidylinositol-4-Phosphate 5-Kinase Type 1 Beta
5905.	PMP2	Peripheral Myelin Protein 2
5906.	POLR1A	RNA Polymerase I Subunit A
5907.	PTPRK	Protein Tyrosine Phosphatase Receptor Type K
5908.	RALBP1	RalA Binding Protein 1
5909.	RICTOR	RPTOR Independent Companion Of MTOR Complex 2
5910.	RNF216	Ring Finger Protein 216
5911.	RPL21	Ribosomal Protein L21
5912.	RPS3	Ribosomal Protein S3
5913.	RXFP2	Relaxin Family Peptide Receptor 2
5914.	SEMA3F	Semaphorin 3F
5915.	SETD1A	SET Domain Containing 1A, Histone Lysine Methyltransferase
5916.	SLC18A1	Solute Carrier Family 18 Member A1
5917.	SLC44A1	Solute Carrier Family 44 Member 1
5918.	SNRPB	Small Nuclear Ribonucleoprotein Polypeptides B And B1
5919.	SPRY4	Sprouty RTK Signaling Antagonist 4
5920.	ST3GAL4	ST3 Beta-Galactoside Alpha-2,3-Sialyltransferase 4
5921.	SYCP3	Synaptonemal Complex Protein 3
5922.	TGM3	Transglutaminase 3
5923.	TP53RK	TP53 Regulating Kinase
5924.	TREM1	Triggering Receptor Expressed On Myeloid Cells 1
5925.	TULP3	TUB Like Protein 3
5926.	TWIST2	Twist Family BHLH Transcription Factor 2
5927.	UBTF	Upstream Binding Transcription Factor
5928.	UGT2B15	UDP Glucuronosyltransferase Family 2 Member B15
5929.	UNC119	Unc-119 Lipid Binding Chaperone
5930.	UPP1	Uridine Phosphorylase 1
5931.	AATF	Apoptosis Antagonizing Transcription Factor
5932.	ABI1	Abl Interactor 1
5933.	AP2B1	Adaptor Related Protein Complex 2 Subunit Beta 1
5934.	AQP9	Aquaporin 9
5935.	ARHGDIB	Rho GDP Dissociation Inhibitor Beta
5936.	ATP5F1E	ATP Synthase F1 Subunit Epsilon
5937.	BCL9	BCL9 Transcription Coactivator
5938.	C8B	Complement C8 Beta Chain

5939.	CEP57	Centrosomal Protein 57
5940.	CHMP1A	Charged Multivesicular Body Protein 1A
5941.	CNDP2	Carnosine Dipeptidase 2
5942.	COLEC11	Collectin Subfamily Member 11
5943.	DUOX1	Dual Oxidase 1
5944.	ECT2	Epithelial Cell Transforming 2
5945.	EPB41L3	Erythrocyte Membrane Protein Band 4.1 Like 3
5946.	ERC1	ELKS/RAB6-Interacting/CAST Family Member 1
5947.	EXOSC5	Exosome Component 5
5948.	GCLM	Glutamate-Cysteine Ligase Modifier Subunit
5949.	GDF9	Growth Differentiation Factor 9
5950.	GMPS	Guanine Monophosphate Synthase
5951.	GPAA1	Glycosylphosphatidylinositol Anchor Attachment 1
5952.	HNRNPD	Heterogeneous Nuclear Ribonucleoprotein D
5953.	HSD17B1	Hydroxysteroid 17-Beta Dehydrogenase 1
5954.	HYAL2	Hyaluronidase 2
5955.	ICAM3	Intercellular Adhesion Molecule 3
5956.	IL1RL1	Interleukin 1 Receptor Like 1
5957.	INPP5E	Inositol Polyphosphate-5-Phosphatase E
5958.	IRS4	Insulin Receptor Substrate 4
5959.	IYD	Iodotyrosine Deiodinase
5960.	KCNN2	Potassium Calcium-Activated Channel Subfamily N Member 2
5961.	MATN1	Matrilin 1
5962.	NEUROD2	Neuronal Differentiation 2
5963.	NFATC3	Nuclear Factor Of Activated T Cells 3
5964.	NIN	Ninein
5965.	NUP133	Nucleoporin 133
5966.	PEA15	Proliferation And Apoptosis Adaptor Protein 15
5967.	PGAP1	Post-GPI Attachment To Proteins Inositol Deacylase 1
5968.	PLEKHG5	Pleckstrin Homology And RhoGEF Domain Containing G5
5969.	POLR2B	RNA Polymerase II Subunit B
5970.	POLR3F	RNA Polymerase III Subunit F
5971.	PROX1	Prospero Homeobox 1
5972.	PSMD9	Proteasome 26S Subunit, Non-ATPase 9
5973.	RAD54B	RAD54 Homolog B
5974.	RANBP9	RAN Binding Protein 9
5975.	RAPGEF1	Rap Guanine Nucleotide Exchange Factor 1
5976.	RPS13	Ribosomal Protein S13
5977.	RPS16	Ribosomal Protein S16
5978.	RPS23	Ribosomal Protein S23
5979.	RXRG	Retinoid X Receptor Gamma

5980.	SCAP	SREBF Chaperone
5981.	SERPINA5	Serpin Family A Member 5
5982.	SH3GL3	SH3 Domain Containing GRB2 Like 3, Endophilin A3
5983.	SHANK3	SH3 And Multiple Ankyrin Repeat Domains 3
5984.	SLC15A1	Solute Carrier Family 15 Member 1
5985.	SNRPE	Small Nuclear Ribonucleoprotein Polypeptide E
5986.	SPINT1	Serine Peptidase Inhibitor, Kunitz Type 1
5987.	SPRY1	Sprouty RTK Signaling Antagonist 1
5988.	SRGAP1	SLIT-ROBO Rho GTPase Activating Protein 1
5989.	TCERG1	Transcription Elongation Regulator 1
5990.	THBS4	Thrombospondin 4
5991.	TPBG	Trophoblast Glycoprotein
5992.	TRAF4	TNF Receptor Associated Factor 4
5993.	TRIB3	Tribbles Pseudokinase 3
5994.	TRIP13	Thyroid Hormone Receptor Interactor 13
5995.	TTPA	Alpha Tocopherol Transfer Protein
5996.	UBE2C	Ubiquitin Conjugating Enzyme E2 C
5997.	UBIAD1	UbiA Prenyltransferase Domain Containing 1
5998.	UFM1	Ubiquitin Fold Modifier 1
5999.	UGT2B17	UDP Glucuronosyltransferase Family 2 Member B17
6000.	WIPI2	WD Repeat Domain, Phosphoinositide Interacting 2
6001.	WNT9B	Wnt Family Member 9B
6002.	ANXA7	Annexin A7
6003.	APBA1	Amyloid Beta Precursor Protein Binding Family A Member 1
6004.	APOC1	Apolipoprotein C1
6005.	ARHGAP5	Rho GTPase Activating Protein 5
6006.	BRS3	Bombesin Receptor Subtype 3
6007.	CEP41	Centrosomal Protein 41
6008.	CHRNA6	Cholinergic Receptor Nicotinic Alpha 6 Subunit
6009.	COX7B	Cytochrome C Oxidase Subunit 7B
6010.	CYP2C18	Cytochrome P450 Family 2 Subfamily C Member 18
6011.	DDX3Y	DEAD-Box Helicase 3 Y-Linked
6012.	DEAF1	DEAF1 Transcription Factor
6013.	EFNB3	Ephrin B3
6014.	EXOC5	Exocyst Complex Component 5
6015.	FCAR	Fc Alpha Receptor
6016.	GABRR1	Gamma-Aminobutyric Acid Type A Receptor Subunit Rho1
6017.	KIF3B	Kinesin Family Member 3B
6018.	KLC2	Kinesin Light Chain 2
6019.	LEMD3	LEM Domain Containing 3
6020.	LHX1	LIM Homeobox 1

6021.	MAGT1	Magnesium Transporter 1
6022.	MCM8	Minichromosome Maintenance 8 Homologous Recombination Repair Factor
6023.	MCPH1	Microcephalin 1
6024.	MLNR	Motilin Receptor
6025.	MSRA	Methionine Sulfoxide Reductase A
6026.	NDUFA2	NADH:Ubiquinone Oxidoreductase Subunit A2
6027.	NDUFAB1	NADH:Ubiquinone Oxidoreductase Subunit AB1
6028.	NKX2-2	NK2 Homeobox 2
6029.	NMBR	Neuromedin B Receptor
6030.	NUP160	Nucleoporin 160
6031.	PASK	PAS Domain Containing Serine/Threonine Kinase
6032.	POLR2C	RNA Polymerase II Subunit C
6033.	POU2F2	POU Class 2 Homeobox 2
6034.	PPIC	Peptidylprolyl Isomerase C
6035.	PPP1R9B	Protein Phosphatase 1 Regulatory Subunit 9B
6036.	RPL7A	Ribosomal Protein L7a
6037.	SNIP1	Smad Nuclear Interacting Protein 1
6038.	TERF2	Telomeric Repeat Binding Factor 2
6039.	TRIM63	Tripartite Motif Containing 63
6040.	TTC8	Tetratricopeptide Repeat Domain 8
6041.	USP24	Ubiquitin Specific Peptidase 24
6042.	WDR35	WD Repeat Domain 35
6043.	WNT6	Wnt Family Member 6
6044.	ACSBG1	Acyl-CoA Synthetase Bubblegum Family Member 1
6045.	ADAM28	ADAM Metallopeptidase Domain 28
6046.	ALPK1	Alpha Kinase 1
6047.	ANKS6	Ankyrin Repeat And Sterile Alpha Motif Domain Containing 6
6048.	ANO3	Anoctamin 3
6049.	CACNB3	Calcium Voltage-Gated Channel Auxiliary Subunit Beta 3
6050.	CACNG3	Calcium Voltage-Gated Channel Auxiliary Subunit Gamma 3
6051.	CAP1	Cyclase Associated Actin Cytoskeleton Regulatory Protein 1
6052.	CAPN9	Calpain 9
6053.	CAPZA1	Capping Actin Protein Of Muscle Z-Line Subunit Alpha 1
6054.	CDO1	Cysteine Dioxygenase Type 1
6055.	CEP120	Centrosomal Protein 120
6056.	CHRNB3	Cholinergic Receptor Nicotinic Beta 3 Subunit
6057.	CXCL2	C-X-C Motif Chemokine Ligand 2
6058.	DAAM2	Dishevelled Associated Activator Of Morphogenesis 2
6059.	DCDC2	Doublecortin Domain Containing 2
6060.	DEF6	DEF6 Guanine Nucleotide Exchange Factor
6061.	DGKB	Diacylglycerol Kinase Beta

6062.	DIO1	Iodothyronine Deiodinase 1
6063.	DMRT1	Doublesex And Mab-3 Related Transcription Factor 1
6064.	DNAJB11	DnaJ Heat Shock Protein Family (Hsp40) Member B11
6065.	ELF3	E74 Like ETS Transcription Factor 3
6066.	ELMO1	Engulfment And Cell Motility 1
6067.	EXOSC8	Exosome Component 8
6068.	FBXO32	F-Box Protein 32
6069.	FFAR2	Free Fatty Acid Receptor 2
6070.	FLVCR2	FLVCR Choline And Putative Heme Transporter 2
6071.	FOXH1	Forkhead Box H1
6072.	FSCN2	Fascin Actin-Bundling Protein 2, Retinal
6073.	FUT3	Fucosyltransferase 3 (Lewis Blood Group)
6074.	FYCO1	FYVE And Coiled-Coil Domain Autophagy Adaptor 1
6075.	GABRR2	Gamma-Aminobutyric Acid Type A Receptor Subunit Rho2
6076.	GPR161	G Protein-Coupled Receptor 161
6077.	HDLBP	High Density Lipoprotein Binding Protein
6078.	HOXC13	Homeobox C13
6079.	IFT27	Intraflagellar Transport 27
6080.	INSL3	Insulin Like 3
6081.	IQSEC2	IQ Motif And Sec7 Domain ArfGEF 2
6082.	IST1	IST1 Factor Associated With ESCRT-III
6083.	KIF7	Kinesin Family Member 7
6084.	KLK10	Kallikrein Related Peptidase 10
6085.	LSM1	LSM1 Homolog, MRNA Degradation Associated
6086.	MAP1LC3A	Microtubule Associated Protein 1 Light Chain 3 Alpha
6087.	MCEE	Methylmalonyl-CoA Epimerase
6088.	MIP	Major Intrinsic Protein Of Lens Fiber
6089.	MKRN3	Makorin Ring Finger Protein 3
6090.	MTFMT	Mitochondrial Methionyl-TRNA Formyltransferase
6091.	MYF5	Myogenic Factor 5
6092.	MYLK3	Myosin Light Chain Kinase 3
6093.	MYSM1	Myb Like, SWIRM And MPN Domains 1
6094.	NDUFA5	NADH:Ubiquinone Oxidoreductase Subunit A5
6095.	NDUFB7	NADH:Ubiquinone Oxidoreductase Subunit B7
6096.	NDUFC2	NADH:Ubiquinone Oxidoreductase Subunit C2
6097.	NKX6-1	NK6 Homeobox 1
6098.	NMB	Neuromedin B
6099.	NR2C1	Nuclear Receptor Subfamily 2 Group C Member 1
6100.	NRXN2	Neurexin 2
6101.	NUP153	Nucleoporin 153
6102.	NUP205	Nucleoporin 205

6103.	NUP93	Nucleoporin 93
6104.	OSBP	Oxysterol Binding Protein
6105.	OTUD5	OTU Deubiquitinase 5
6106.	PACSIN2	Protein Kinase C And Casein Kinase Substrate In Neurons 2
6107.	PDIA4	Protein Disulfide Isomerase Family A Member 4
6108.	PLXNC1	Plexin C1
6109.	POLR2D	RNA Polymerase II Subunit D
6110.	PPIE	Peptidylprolyl Isomerase E
6111.	PPIL1	Peptidylprolyl Isomerase Like 1
6112.	PSMC3IP	PSMC3 Interacting Protein
6113.	RAB3GAP2	RAB3 GTPase Activating Non-Catalytic Protein Subunit 2
6114.	RARRES2	Retinoic Acid Receptor Responder 2
6115.	RBBP5	RB Binding Protein 5, Histone Lysine Methyltransferase Complex Subunit
6116.	RNF220	Ring Finger Protein 220
6117.	RPL13A	Ribosomal Protein L13a
6118.	RPL19	Ribosomal Protein L19
6119.	RPLP1	Ribosomal Protein Lateral Stalk Subunit P1
6120.	RPS2	Ribosomal Protein S2
6121.	SENP1	SUMO Specific Peptidase 1
6122.	SERPINA12	Serpin Family A Member 12
6123.	SLC27A1	Solute Carrier Family 27 Member 1
6124.	SLC30A1	Solute Carrier Family 30 Member 1
6125.	SLC36A2	Solute Carrier Family 36 Member 2
6126.	SLC44A2	Solute Carrier Family 44 Member 2 (CTL2 Blood Group)
6127.	SLC6A20	Solute Carrier Family 6 Member 20
6128.	SLC9A2	Solute Carrier Family 9 Member A2
6129.	SMC5	Structural Maintenance Of Chromosomes 5
6130.	SNTB1	Syntrophin Beta 1
6131.	SNX5	Sorting Nexin 5
6132.	SORBS1	Sorbin And SH3 Domain Containing 1
6133.	SORBS2	Sorbin And SH3 Domain Containing 2
6134.	ST6GALNAC1	ST6 N-Acetylgalactosaminide Alpha-2,6-Sialyltransferase 1
6135.	STIL	STIL Centriolar Assembly Protein
6136.	SUN1	Sad1 And UNC84 Domain Containing 1
6137.	TACC1	Transforming Acidic Coiled-Coil Containing Protein 1
6138.	TMC1	Transmembrane Channel Like 1
6139.	TNS1	Tensin 1
6140.	TOB1	Transducer Of ERBB2, 1
6141.	TRAIP	TRAF Interacting Protein
6142.	TRDMT1	TRNA Aspartic Acid Methyltransferase 1
6143.	UBA5	Ubiquitin Like Modifier Activating Enzyme 5

6144.	USF2	Upstream Transcription Factor 2, C-Fos Interacting
6145.	USP9Y	Ubiquitin Specific Peptidase 9 Y-Linked
6146.	UTS2	Urotensin 2
6147.	AADAT	Aminoadipate Aminotransferase
6148.	ACTR3B	Actin Related Protein 3B
6149.	ADAMTS7	ADAM Metallopeptidase With Thrombospondin Type 1 Motif 7
6150.	AGBL5	AGBL Carboxypeptidase 5
6151.	AKAP6	A-Kinase Anchoring Protein 6
6152.	AMY2A	Amylase Alpha 2A
6153.	APIP	APAF1 Interacting Protein
6154.	APOM	Apolipoprotein M
6155.	ARAP1	ArfGAP With RhoGAP Domain, Ankyrin Repeat And PH Domain 1
6156.	ATPAF2	ATP Synthase Mitochondrial F1 Complex Assembly Factor 2
6157.	BAIAP2L1	BAR/IMD Domain Containing Adaptor Protein 2 Like 1
6158.	BMS1	BMS1 Ribosome Biogenesis Factor
6159.	BRAT1	BRCA1 Associated ATM Activator 1
6160.	C2CD3	C2 Domain Containing 3 Centriole Elongation Regulator
6161.	C5AR2	Complement C5a Receptor 2
6162.	C8A	Complement C8 Alpha Chain
6163.	CAPZA2	Capping Actin Protein Of Muscle Z-Line Subunit Alpha 2
6164.	CCDC6	Coiled-Coil Domain Containing 6
6165.	CD8B	CD8 Subunit Beta
6166.	CELA3B	Chymotrypsin Like Elastase 3B
6167.	CFHR2	Complement Factor H Related 2
6168.	CIDEA	Cell Death Inducing DFFA Like Effector A
6169.	CLK4	CDC Like Kinase 4
6170.	CLPS	Colipase
6171.	CSPP1	Centrosome And Spindle Pole Associated Protein 1
6172.	CYP4F3	Cytochrome P450 Family 4 Subfamily F Member 3
6173.	DIO3	Iodothyronine Deiodinase 3
6174.	DPY19L2	Dpy-19 Like 2
6175.	DTX1	Deltex E3 Ubiquitin Ligase 1
6176.	DUSP2	Dual Specificity Phosphatase 2
6177.	DZIP1	DAZ Interacting Zinc Finger Protein 1
6178.	EIF3G	Eukaryotic Translation Initiation Factor 3 Subunit G
6179.	EVL	Enah/Vasp-Like
6180.	FBXO3	F-Box Protein 3
6181.	FCRL5	Fc Receptor Like 5
6182.	FGL1	Fibrinogen Like 1
6183.	FOXD3	Forkhead Box D3
6184.	FOXJ1	Forkhead Box J1

6185.	GJB5	Gap Junction Protein Beta 5
6186.	GPRC5A	G Protein-Coupled Receptor Class C Group 5 Member A
6187.	HDGF	Heparin Binding Growth Factor
6188.	HHAT	Hedgehog Acyltransferase
6189.	HYLS1	HYLS1 Centriolar And Ciliogenesis Associated
6190.	ID3	Inhibitor Of DNA Binding 3
6191.	IL25	Interleukin 25
6192.	ITLN1	Intelectin 1
6193.	KCNT2	Potassium Sodium-Activated Channel Subfamily T Member 2
6194.	KCNU1	Potassium Calcium-Activated Channel Subfamily U Member 1
6195.	KLRB1	Killer Cell Lectin Like Receptor B1
6196.	LHB	Luteinizing Hormone Subunit Beta
6197.	LMF1	Lipase Maturation Factor 1
6198.	LRP1B	LDL Receptor Related Protein 1B
6199.	MAML3	Mastermind Like Transcriptional Coactivator 3
6200.	MAPKBP1	Mitogen-Activated Protein Kinase Binding Protein 1
6201.	MARCKS	Myristoylated Alanine Rich Protein Kinase C Substrate
6202.	MBD5	Methyl-CpG Binding Domain Protein 5
6203.	MFF	Mitochondrial Fission Factor
6204.	MGAT3	Beta-1,4-Mannosyl-Glycoprotein 4-Beta-N-Acetylglucosaminyltransferase
6205.	MOXD1	Monooxygenase DBH Like 1
6206.	MRPL3	Mitochondrial Ribosomal Protein L3
6207.	MYBL1	MYB Proto-Oncogene Like 1
6208.	MYOM1	Myomesin 1
6209.	NCOA4	Nuclear Receptor Coactivator 4
6210.	NDUFS5	NADH:Ubiquinone Oxidoreductase Subunit S5
6211.	NDUFV3	NADH:Ubiquinone Oxidoreductase Subunit V3
6212.	NFYC	Nuclear Transcription Factor Y Subunit Gamma
6213.	NKX6-2	NK6 Homeobox 2
6214.	NPTN	Neuroplastin
6215.	NRGN	Neurogranin
6216.	PARP9	Poly(ADP-Ribose) Polymerase Family Member 9
6217.	PITPNB	Phosphatidylinositol Transfer Protein Beta
6218.	PRDM14	PR/SET Domain 14
6219.	PTPN4	Protein Tyrosine Phosphatase Non-Receptor Type 4
6220.	RCN2	Reticulocalbin 2
6221.	RIOK2	RIO Kinase 2
6222.	RPL12	Ribosomal Protein L12
6223.	RPL23A	Ribosomal Protein L23a
6224.	RPL30	Ribosomal Protein L30
6225.	RPL34	Ribosomal Protein L34

6226.	RPS3A	Ribosomal Protein S3A
6227.	RRP7A	Ribosomal RNA Processing 7 Homolog A
6228.	SCUBE3	Signal Peptide, CUB Domain And EGF Like Domain Containing 3
6229.	SHOX	SHOX Homeobox
6230.	SLC41A1	Solute Carrier Family 41 Member 1
6231.	SLC9A7	Solute Carrier Family 9 Member A7
6232.	SMC2	Structural Maintenance Of Chromosomes 2
6233.	SORCS1	Sortilin Related VPS10 Domain Containing Receptor 1
6234.	SPAG9	Sperm Associated Antigen 9
6235.	SPO11	SPO11 Initiator Of Meiotic Double Strand Breaks
6236.	STEAP2	STEAP2 Metalloreductase
6237.	STRN	Striatin
6238.	SYNPO	Synaptopodin
6239.	TAF10	TATA-Box Binding Protein Associated Factor 10
6240.	TEKT3	Tektin 3
6241.	TEP1	Telomerase Associated Protein 1
6242.	TLN2	Talin 2
6243.	TLX1	T Cell Leukemia Homeobox 1
6244.	TP53BP2	Tumor Protein P53 Binding Protein 2
6245.	TSPAN8	Tetraspanin 8
6246.	TTC19	Tetratricopeptide Repeat Domain 19
6247.	TXNIP	Thioredoxin Interacting Protein
6248.	UBASH3A	Ubiquitin Associated And SH3 Domain Containing A
6249.	UFC1	Ubiquitin-Fold Modifier Conjugating Enzyme 1
6250.	UGT2B10	UDP Glucuronosyltransferase Family 2 Member B10
6251.	UGT2B4	UDP Glucuronosyltransferase Family 2 Member B4
6252.	UXS1	UDP-Glucuronate Decarboxylase 1
6253.	VSIR	V-Set Immunoregulatory Receptor
6254.	ZDHHC13	Zinc Finger DHHC-Type Palmitoyltransferase 13
6255.	ZFP57	ZFP57 Zinc Finger Protein
6256.	ZNF423	Zinc Finger Protein 423
6257.	ACSL6	Acyl-CoA Synthetase Long Chain Family Member 6
6258.	ACSM1	Acyl-CoA Synthetase Medium Chain Family Member 1
6259.	AP5Z1	Adaptor Related Protein Complex 5 Subunit Zeta 1
6260.	BOK	BCL2 Family Apoptosis Regulator BOK
6261.	BYSL	Bystin Like
6262.	CABLES1	Cdk5 And Abl Enzyme Substrate 1
6263.	CCDC115	Coiled-Coil Domain Containing 115
6264.	CCT6B	Chaperonin Containing TCP1 Subunit 6B
6265.	CDCP1	CUB Domain Containing Protein 1
6266.	CEP43	Centrosomal Protein 43

6267.	CHD6	Chromodomain Helicase DNA Binding Protein 6
6268.	CLPTM1L	CLPTM1 Like
6269.	CNMD	Chondromodulin
6270.	CPSF6	Cleavage And Polyadenylation Specific Factor 6
6271.	CRELD2	Cysteine Rich With EGF Like Domains 2
6272.	CSDE1	Cold Shock Domain Containing E1
6273.	CSMD1	CUB And Sushi Multiple Domains 1
6274.	CYP2A13	Cytochrome P450 Family 2 Subfamily A Member 13
6275.	DDX10	DEAD-Box Helicase 10
6276.	DEFA5	Defensin Alpha 5
6277.	DNAAF4	Dynein Axonemal Assembly Factor 4
6278.	DUSP9	Dual Specificity Phosphatase 9
6279.	FAM3D	FAM3 Metabolism Regulating Signaling Molecule D
6280.	FBXO5	F-Box Protein 5
6281.	FCHO1	FCH And Mu Domain Containing Endocytic Adaptor 1
6282.	FHOD3	Formin Homology 2 Domain Containing 3
6283.	FOXA3	Forkhead Box A3
6284.	GABRE	Gamma-Aminobutyric Acid Type A Receptor Subunit Epsilon
6285.	GABRQ	Gamma-Aminobutyric Acid Type A Receptor Subunit Theta
6286.	GADD45G	Growth Arrest And DNA Damage Inducible Gamma
6287.	GLRX2	Glutaredoxin 2
6288.	GPM6B	Glycoprotein M6B
6289.	HEATR1	HEAT Repeat Containing 1
6290.	HELQ	Helicase, POLQ Like
6291.	HJURP	Holliday Junction Recognition Protein
6292.	IFT56	Intraflagellar Transport 56
6293.	IL18RAP	Interleukin 18 Receptor Accessory Protein
6294.	IQGAP3	IQ Motif Containing GTPase Activating Protein 3
6295.	KIF17	Kinesin Family Member 17
6296.	LARGE2	LARGE Xylosyl- And Glucuronyltransferase 2
6297.	LDB1	LIM Domain Binding 1
6298.	LILRA4	Leukocyte Immunoglobulin Like Receptor A4
6299.	LIMD1	LIM Domain Containing 1
6300.	MAGEA4	MAGE Family Member A4
6301.	MCM10	Minichromosome Maintenance 10 Replication Initiation Factor
6302.	MX2	MX Dynamin Like GTPase 2
6303.	MYBPH	Myosin Binding Protein H
6304.	NDUFB6	NADH:Ubiquinone Oxidoreductase Subunit B6
6305.	NOP2	NOP2 Nucleolar Protein
6306.	NOVA1	NOVA Alternative Splicing Regulator 1
6307.	NPNT	Nephronectin

6308.	NUP188	Nucleoporin 188
6309.	NUP54	Nucleoporin 54
6310.	PDCD5	Programmed Cell Death 5
6311.	PGRMC2	Progesterone Receptor Membrane Component 2
6312.	PI3	Peptidase Inhibitor 3
6313.	PIDD1	P53-Induced Death Domain Protein 1
6314.	PSMG2	Proteasome Assembly Chaperone 2
6315.	PUS10	Pseudouridine Synthase 10
6316.	RAB3IP	RAB3A Interacting Protein
6317.	RASD1	Ras Related Dexamethasone Induced 1
6318.	RASD2	RASD Family Member 2
6319.	RASGRP4	RAS Guanyl Releasing Protein 4
6320.	RASSF5	Ras Association Domain Family Member 5
6321.	RBM5	RNA Binding Motif Protein 5
6322.	RBMS1	RNA Binding Motif Single Stranded Interacting Protein 1
6323.	RFX6	Regulatory Factor X6
6324.	RNF7	Ring Finger Protein 7
6325.	RNLS	Renalase, FAD Dependent Amine Oxidase
6326.	RPL14	Ribosomal Protein L14
6327.	RPS4X	Ribosomal Protein S4 X-Linked
6328.	RPS8	Ribosomal Protein S8
6329.	RRAD	RRAD, Ras Related Glycolysis Inhibitor And Calcium Channel Regulator
6330.	RRN3	RRN3 Homolog, RNA Polymerase I Transcription Factor
6331.	SCARA5	Scavenger Receptor Class A Member 5
6332.	SCLY	Selenocysteine Lyase
6333.	SEMA3D	Semaphorin 3D
6334.	SEZ6L	Seizure Related 6 Homolog Like
6335.	SF1	Splicing Factor 1
6336.	SLC26A7	Solute Carrier Family 26 Member 7
6337.	SLC5A11	Solute Carrier Family 5 Member 11
6338.	SOX1	SRY-Box Transcription Factor 1
6339.	SPON2	Spondin 2
6340.	SRY	Sex Determining Region Y
6341.	SWAP70	Switching B Cell Complex Subunit SWAP70
6342.	SYCE1	Synaptonemal Complex Central Element Protein 1
6343.	TES	Testin LIM Domain Protein
6344.	THEMIS	Thymocyte Selection Associated
6345.	UQCC2	Ubiquinol-Cytochrome C Reductase Complex Assembly Factor 2
6346.	WDPCP	WD Repeat Containing Planar Cell Polarity Effector
6347.	WDR12	WD Repeat Domain 12
6348.	WEE2	WEE2 Oocyte Meiosis Inhibiting Kinase

6349.	ZDHHC17	Zinc Finger DHHC-Type Palmitoyltransferase 17
6350.	ANAPC5	Anaphase Promoting Complex Subunit 5
6351.	AQP6	Aquaporin 6
6352.	ATG14	Autophagy Related 14
6353.	BPIFA1	BPI Fold Containing Family A Member 1
6354.	BTBD9	BTB Domain Containing 9
6355.	CAVIN3	Caveolae Associated Protein 3
6356.	CDC123	Cell Division Cycle 123
6357.	CDX1	Caudal Type Homeobox 1
6358.	CIR1	Corepressor Interacting With RBPJ, CIR1
6359.	CNTN5	Contactin 5
6360.	CREB5	CAMP Responsive Element Binding Protein 5
6361.	CTU2	Cytosolic Thiouridylase Subunit 2
6362.	DDRGK1	DDRGK Domain Containing 1
6363.	DDX52	DExD-Box Helicase 52
6364.	DYNC2I2	Dynein 2 Intermediate Chain 2
6365.	EDC4	Enhancer Of MRNA Decapping 4
6366.	ELAVL3	ELAV Like RNA Binding Protein 3
6367.	EMB	Embigin
6368.	EPSTI1	Epithelial Stromal Interaction 1
6369.	FAM111A	FAM111 Trypsin Like Peptidase A
6370.	FIGLA	Folliculogenesis Specific BHLH Transcription Factor
6371.	FNIP1	Folliculin Interacting Protein 1
6372.	FRMD6	FERM Domain Containing 6
6373.	GABRR3	Gamma-Aminobutyric Acid Type A Receptor Subunit Rho3
6374.	GDF1	Growth Differentiation Factor 1
6375.	GIPC3	GIPC PDZ Domain Containing Family Member 3
6376.	GKN1	Gastrokine 1
6377.	GOLGA3	Golgin A3
6378.	GPIHBP1	Glycosylphosphatidylinositol Anchored High Density Lipoprotein Binding Protein 1
6379.	GPR119	G Protein-Coupled Receptor 119
6380.	GSE1	Gse1 Coiled-Coil Protein
6381.	HOXB9	Homeobox B9
6382.	HSPB6	Heat Shock Protein Family B (Small) Member 6
6383.	HSPB7	Heat Shock Protein Family B (Small) Member 7
6384.	HTRA3	HtrA Serine Peptidase 3
6385.	IFNL1	Interferon Lambda 1
6386.	IFNLR1	Interferon Lambda Receptor 1
6387.	KCMF1	Potassium Channel Modulatory Factor 1
6388.	KIF6	Kinesin Family Member 6
6389.	LDB2	LIM Domain Binding 2

6390.	MBD6	Methyl-CpG Binding Domain Protein 6
6391.	NEK10	NIMA Related Kinase 10
6392.	NFYB	Nuclear Transcription Factor Y Subunit Beta
6393.	NOB1	NIN1 (RPN12) Binding Protein 1 Homolog
6394.	NOP58	NOP58 Ribonucleoprotein
6395.	OMA1	OMA1 Zinc Metallopeptidase
6396.	OSBP2	Oxysterol Binding Protein 2
6397.	PNO1	Partner Of NOB1 Homolog
6398.	POLR3H	RNA Polymerase III Subunit H
6399.	PRDM9	PR/SET Domain 9
6400.	PRPF40A	Pre-MRNA Processing Factor 40 Homolog A
6401.	PRSS16	Serine Protease 16
6402.	PWP2	PWP2 Small Subunit Processome Component
6403.	RGS1	Regulator Of G Protein Signaling 1
6404.	RNH1	Ribonuclease/Angiogenin Inhibitor 1
6405.	RPL10L	Ribosomal Protein L10 Like
6406.	RPL37	Ribosomal Protein L37
6407.	RPL37A	Ribosomal Protein L37a
6408.	RPS18	Ribosomal Protein S18
6409.	RPS21	Ribosomal Protein S21
6410.	SCGB2A2	Secretoglobin Family 2A Member 2
6411.	SEC11A	SEC11 Homolog A, Signal Peptidase Complex Subunit
6412.	SEPTIN12	Septin 12
6413.	SF3A3	Splicing Factor 3a Subunit 3
6414.	SFRP5	Secreted Frizzled Related Protein 5
6415.	SGSM3	Small G Protein Signaling Modulator 3
6416.	SLC38A3	Solute Carrier Family 38 Member 3
6417.	SLC38A4	Solute Carrier Family 38 Member 4
6418.	SMG7	SMG7 Nonsense Mediated MRNA Decay Factor
6419.	SNRPD3	Small Nuclear Ribonucleoprotein D3 Polypeptide
6420.	SOX21	SRY-Box Transcription Factor 21
6421.	SOX30	SRY-Box Transcription Factor 30
6422.	TEX11	Testis Expressed 11
6423.	TMEM107	Transmembrane Protein 107
6424.	TMEM237	Transmembrane Protein 237
6425.	TPPP3	Tubulin Polymerization Promoting Protein Family Member 3
6426.	TRIB2	Tribbles Pseudokinase 2
6427.	TSNAX	Translin Associated Factor X
6428.	UBR7	Ubiquitin Protein Ligase E3 Component N-Recognin 7
6429.	UGT2A3	UDP Glucuronosyltransferase Family 2 Member A3
6430.	UGT2B11	UDP Glucuronosyltransferase Family 2 Member B11

6431.	UGT2B28	UDP Glucuronosyltransferase Family 2 Member B28
6432.	UQCRH	Ubiquinol-Cytochrome C Reductase Hinge Protein
6433.	USP42	Ubiquitin Specific Peptidase 42
6434.	VGF	VGF Nerve Growth Factor Inducible
6435.	XPO7	Exportin 7
6436.	ZC3HC1	Zinc Finger C3HC-Type Containing 1
6437.	ZFAND6	Zinc Finger AN1-Type Containing 6
6438.	ZFX	Zinc Finger Protein X-Linked
6439.	ZNF804A	Zinc Finger Protein 804A
6440.	ADAD1	Adenosine Deaminase Domain Containing 1
6441.	AGBL2	AGBL Carboxypeptidase 2
6442.	ANKFY1	Ankyrin Repeat And FYVE Domain Containing 1
6443.	ANXA10	Annexin A10
6444.	API5	Apoptosis Inhibitor 5
6445.	B3GAT2	Beta-1,3-Glucuronyltransferase 2
6446.	CACNG7	Calcium Voltage-Gated Channel Auxiliary Subunit Gamma 7
6447.	CACNG8	Calcium Voltage-Gated Channel Auxiliary Subunit Gamma 8
6448.	CADM4	Cell Adhesion Molecule 4
6449.	CBLN2	Cerebellin 2 Precursor
6450.	CCAR1	Cell Division Cycle And Apoptosis Regulator 1
6451.	CDK5RAP3	CDK5 Regulatory Subunit Associated Protein 3
6452.	CEP131	Centrosomal Protein 131
6453.	CHERP	Calcium Homeostasis Endoplasmic Reticulum Protein
6454.	CIP2A	Cellular Inhibitor Of PP2A
6455.	CLEC4D	C-Type Lectin Domain Family 4 Member D
6456.	CLTRN	Collectrin, Amino Acid Transport Regulator
6457.	CPPED1	Calcineurin Like Phosphoesterase Domain Containing 1
6458.	DNAH1	Dynein Axonemal Heavy Chain 1
6459.	DUS2	Dihydrouridine Synthase 2
6460.	DZIP1L	DAZ Interacting Zinc Finger Protein 1 Like
6461.	ENOSF1	Enolase Superfamily Member 1
6462.	EXOSC4	Exosome Component 4
6463.	FOXI1	Forkhead Box I1
6464.	FOXK2	Forkhead Box K2
6465.	GPR158	G Protein-Coupled Receptor 158
6466.	GPR176	G Protein-Coupled Receptor 176
6467.	GTDC1	Glycosyltransferase Like Domain Containing 1
6468.	ICAM4	Intercellular Adhesion Molecule 4 (Landsteiner-Wiener Blood Group)
6469.	ITGB1BP2	Integrin Subunit Beta 1 Binding Protein 2
6470.	KIAA0586	KIAA0586
6471.	KIF12	Kinesin Family Member 12

6472.	LY6K	Lymphocyte Antigen 6 Family Member K
6473.	METTL13	Methyltransferase 13, EEF1A N-Terminus And K55
6474.	MKX	Mohawk Homeobox
6475.	MRM1	Mitochondrial RRNA Methyltransferase 1
6476.	NIP7	Nucleolar Pre-RRNA Processing Protein NIP7
6477.	NMRK2	Nicotinamide Riboside Kinase 2
6478.	NOXA1	NADPH Oxidase Activator 1
6479.	NUDT15	Nudix Hydrolase 15
6480.	NUP42	Nucleoporin 42
6481.	NUTF2	Nuclear Transport Factor 2
6482.	OLIG1	Oligodendrocyte Transcription Factor 1
6483.	PGA5	Pepsinogen A5
6484.	PIK3AP1	Phosphoinositide-3-Kinase Adaptor Protein 1
6485.	PPHLN1	Periphilin 1
6486.	PSPN	Persephin
6487.	RAD51AP1	RAD51 Associated Protein 1
6488.	RIMBP2	RIMS Binding Protein 2
6489.	RPF2	Ribosome Production Factor 2 Homolog
6490.	RPL22L1	Ribosomal Protein L22 Like 1
6491.	RPS27L	Ribosomal Protein S27 Like
6492.	S100A13	S100 Calcium Binding Protein A13
6493.	SEM1	SEM1 26S Proteasome Subunit
6494.	SLC2A4RG	SLC2A4 Regulator
6495.	SPATA16	Spermatogenesis Associated 16
6496.	SPIRE1	Spire Type Actin Nucleation Factor 1
6497.	SPOCK3	SPARC (Osteonectin), Cwcv And Kazal Like Domains Proteoglycan 3
6498.	TMCC2	Transmembrane And Coiled-Coil Domain Family 2
6499.	TMPRSS11A	Transmembrane Serine Protease 11A
6500.	TRMT10A	TRNA Methyltransferase 10A
6501.	TSPYL2	TSPY Like 2
6502.	TTLL3	Tubulin Tyrosine Ligase Like 3
6503.	WRNIP1	WRN Helicase Interacting Protein 1
6504.	ZBTB32	Zinc Finger And BTB Domain Containing 32
6505.	ZC3H7B	Zinc Finger CCCH-Type Containing 7B
6506.	ZFR	Zinc Finger RNA Binding Protein
6507.	ZNRF3	Zinc And Ring Finger 3
6508.	AHRR	Aryl Hydrocarbon Receptor Repressor
6509.	ANKS3	Ankyrin Repeat And Sterile Alpha Motif Domain Containing 3
6510.	ATP5MK	ATP Synthase Membrane Subunit K
6511.	BOP1	BOP1 Ribosomal Biogenesis Factor
6512.	CAPN8	Calpain 8

6513.	CCDC141	Coiled-Coil Domain Containing 141
6514.	CCNB3	Cyclin B3
6515.	CHSY3	Chondroitin Sulfate Synthase 3
6516.	CLDN23	Claudin 23
6517.	DEPDC1B	DEP Domain Containing 1B
6518.	DUOXA1	Dual Oxidase Maturation Factor 1
6519.	DUOXA2	Dual Oxidase Maturation Factor 2
6520.	FBXO6	F-Box Protein 6
6521.	FNIP2	Folliculin Interacting Protein 2
6522.	FUNDC1	FUN14 Domain Containing 1
6523.	FXYD5	FXYD Domain Containing Ion Transport Regulator 5
6524.	GADD45B	Growth Arrest And DNA Damage Inducible Beta
6525.	GRWD1	Glutamate Rich WD Repeat Containing 1
6526.	H1-6	H1.6 Linker Histone, Cluster Member
6527.	IFT46	Intraflagellar Transport 46
6528.	INAVA	Innate Immunity Activator
6529.	JADE1	Jade Family PHD Finger 1
6530.	KCNG2	Potassium Voltage-Gated Channel Modifier Subfamily G Member 2
6531.	KCNK16	Potassium Two Pore Domain Channel Subfamily K Member 16
6532.	KIF24	Kinesin Family Member 24
6533.	KLHL26	Kelch Like Family Member 26
6534.	LETM2	Leucine Zipper And EF-Hand Containing Transmembrane Protein 2
6535.	LSG1	Large 60S Subunit Nuclear Export GTPase 1
6536.	MAGEC2	MAGE Family Member C2
6537.	MAK16	MAK16 Homolog
6538.	MFHAS1	Multifunctional ROCO Family Signaling Regulator 1
6539.	MRTFB	Myocardin Related Transcription Factor B
6540.	MRTO4	MRT4 Homolog, Ribosome Maturation Factor
6541.	MUC17	Mucin 17, Cell Surface Associated
6542.	MUC3A	Mucin 3A, Cell Surface Associated
6543.	NDUFAF7	NADH:Ubiquinone Oxidoreductase Complex Assembly Factor 7
6544.	NHLRC2	NHL Repeat Containing 2
6545.	NOL6	Nucleolar Protein 6
6546.	NOP53	NOP53 Ribosome Biogenesis Factor
6547.	NRAP	Nebulin Related Anchoring Protein
6548.	NUP35	Nucleoporin 35
6549.	NUP58	Nucleoporin 58
6550.	NXPH4	Neurexophilin 4
6551.	PAK1IP1	PAK1 Interacting Protein 1
6552.	PLAAT4	Phospholipase A And Acyltransferase 4
6553.	PLEKHA3	Pleckstrin Homology Domain Containing A3

6554.	POLR1H	RNA Polymerase I Subunit H
6555.	PUDP	Pseudouridine 5'-Phosphatase
6556.	PURB	Purine Rich Element Binding Protein B
6557.	RBM24	RNA Binding Motif Protein 24
6558.	RBMS2	RNA Binding Motif Single Stranded Interacting Protein 2
6559.	RLF	RLF Zinc Finger
6560.	RNPEPL1	Arginyl Aminopeptidase Like 1
6561.	RPL36A	Ribosomal Protein L36a
6562.	RPL36AL	Ribosomal Protein L36a Like
6563.	SART3	Spliceosome Associated Factor 3, U4/U6 Recycling Protein
6564.	SCLT1	Sodium Channel And Clathrin Linker 1
6565.	SGO2	Shugoshin 2
6566.	SIGLEC15	Sialic Acid Binding Ig Like Lectin 15
6567.	SLC35F6	Solute Carrier Family 35 Member F6
6568.	SLC38A5	Solute Carrier Family 38 Member 5
6569.	SLC41A3	Solute Carrier Family 41 Member 3
6570.	SPIRE2	Spire Type Actin Nucleation Factor 2
6571.	STYX	Serine/Threonine/Tyrosine Interacting Protein
6572.	TCFL5	Transcription Factor Like 5
6573.	TMC2	Transmembrane Channel Like 2
6574.	TRAPPC14	Trafficking Protein Particle Complex Subunit 14
6575.	TRIM68	Tripartite Motif Containing 68
6576.	UCN3	Urocortin 3
6577.	UFL1	UFM1 Specific Ligase 1
6578.	URGCP	Upregulator Of Cell Proliferation
6579.	XRN1	5'-3' Exoribonuclease 1
6580.	ZDHHC24	Zinc Finger DHHC-Type Containing 24
6581.	ZNHIT3	Zinc Finger HIT-Type Containing 3
6582.	ZRANB3	Zinc Finger RANBP2-Type Containing 3
6583.	ZUP1	Zinc Finger Containing Ubiquitin Peptidase 1
6584.	ARSJ	Arylsulfatase Family Member J
6585.	BOD1L1	Biorientation Of Chromosomes In Cell Division 1 Like 1
6586.	CSN2	Casein Beta
6587.	DEF8	Differentially Expressed In FDCP 8 Homolog
6588.	FANK1	Fibronectin Type III And Ankyrin Repeat Domains 1
6589.	GIMAP6	GTPase, IMAP Family Member 6
6590.	GLIS1	GLIS Family Zinc Finger 1
6591.	HELB	DNA Helicase B
6592.	HOPX	HOP Homeobox
6593.	KIAA0930	KIAA0930
6594.	KLHL24	Kelch Like Family Member 24

6595.	LIPI	Lipase I
6596.	LRRN2	Leucine Rich Repeat Neuronal 2
6597.	LTV1	LTV1 Ribosome Biogenesis Factor
6598.	MOSPD2	Motile Sperm Domain Containing 2
6599.	NDC1	NDC1 Transmembrane Nucleoporin
6600.	NIPAL3	NIPA Like Domain Containing 3
6601.	NOL11	Nucleolar Protein 11
6602.	PCLAF	PCNA Clamp Associated Factor
6603.	PPAN	Peter Pan Homolog
6604.	RRS1	Ribosome Biogenesis Regulator 1 Homolog
6605.	RTBDN	Retbindin
6606.	SASH3	SAM And SH3 Domain Containing 3
6607.	SELENOS	Selenoprotein S
6608.	SLC35F5	Solute Carrier Family 35 Member F5
6609.	SLC48A1	Solute Carrier Family 48 Member 1
6610.	TEX15	Testis Expressed 15, Meiosis And Synapsis Associated
6611.	TSPY1	Testis Specific Protein Y-Linked 1
6612.	ZFP90	ZFP90 Zinc Finger Protein
6613.	ANKRD44	Ankyrin Repeat Domain 44
6614.	ANKS4B	Ankyrin Repeat And Sterile Alpha Motif Domain Containing 4B
6615.	ARSI	Arylsulfatase Family Member I
6616.	BLTP1	Bridge-Like Lipid Transfer Protein Family Member 1
6617.	CCDC102A	Coiled-Coil Domain Containing 102A
6618.	CCL23	C-C Motif Chemokine Ligand 23
6619.	CHRFAM7A	CHRNA7 (Exons 5-10) And FAM7A (Exons A-E) Fusion
6620.	CMTR1	Cap Methyltransferase 1
6621.	CNTNAP3	Contactin Associated Protein Family Member 3
6622.	CYRIB	CYFIP Related Rac1 Interactor B
6623.	DAZ1	Deleted In Azoospermia 1
6624.	EME2	Essential Meiotic Structure-Specific Endonuclease Subunit 2
6625.	FABP12	Fatty Acid Binding Protein 12
6626.	FAM168A	Family With Sequence Similarity 168 Member A
6627.	FBF1	Fas Binding Factor 1
6628.	GCSAM	Germinal Center Associated Signaling And Motility
6629.	GFRA4	GDNF Family Receptor Alpha 4
6630.	GKN2	Gastrokine 2
6631.	GSDMA	Gasdermin A
6632.	GTPBP10	GTP Binding Protein 10
6633.	HACD3	3-Hydroxyacyl-CoA Dehydratase 3
6634.	HAUS1	HAUS Augmin Like Complex Subunit 1
6635.	IFT22	Intraflagellar Transport 22

6636.	LRRC10	Leucine Rich Repeat Containing 10
6637.	LRRC14	Leucine Rich Repeat Containing 14
6638.	MFSD10	Major Facilitator Superfamily Domain Containing 10
6639.	MOSPD3	Motile Sperm Domain Containing 3
6640.	MTCP1	Mature T Cell Proliferation 1
6641.	MUC12	Mucin 12, Cell Surface Associated
6642.	MYOM3	Myomesin 3
6643.	NIPAL2	NIPA Like Domain Containing 2
6644.	PALMD	Palmdelphin
6645.	PGA3	Pepsinogen A3
6646.	PRSS57	Serine Protease 57
6647.	REM2	RRAD And GEM Like GTPase 2
6648.	REPIN1	Replication Initiator 1
6649.	RPL39	Ribosomal Protein L39
6650.	RPRM	Reprimo, TP53 Dependent G2 Arrest Mediator Homolog
6651.	RPS19BP1	Ribosomal Protein S19 Binding Protein 1
6652.	SCHIP1	Schwannomin Interacting Protein 1
6653.	SLC25A2	Solute Carrier Family 25 Member 2
6654.	SLC25A35	Solute Carrier Family 25 Member 35
6655.	SLFN11	Schlafen Family Member 11
6656.	SP6	Sp6 Transcription Factor
6657.	SPIDR	Scaffold Protein Involved In DNA Repair
6658.	STK32B	Serine/Threonine Kinase 32B
6659.	SYNRG	Synergin Gamma
6660.	TAAR6	Trace Amine Associated Receptor 6
6661.	TMEM17	Transmembrane Protein 17
6662.	ZFAND3	Zinc Finger AN1-Type Containing 3
6663.	ZFY	Zinc Finger Protein Y-Linked
6664.	ZKSCAN5	Zinc Finger With KRAB And SCAN Domains 5
6665.	AGBL4	AGBL Carboxypeptidase 4
6666.	ANGPTL8	Angiopoietin Like 8
6667.	ANKRD55	Ankyrin Repeat Domain 55
6668.	BBIP1	BBSome Interacting Protein 1
6669.	CLRN2	Clarin 2
6670.	GGACT	Gamma-Glutamylamine Cyclotransferase
6671.	GGNBP2	Gametogenetin Binding Protein 2
6672.	H2AC13	H2A Clustered Histone 13
6673.	KLF14	KLF Transcription Factor 14
6674.	LRTOMT	Leucine Rich Transmembrane And O-Methyltransferase Domain Containing
6675.	MED19	Mediator Complex Subunit 19
6676.	MROH7	Maestro Heat Like Repeat Family Member 7

6677.	MRPL36	Mitochondrial Ribosomal Protein L36
6678.	MS4A4A	Membrane Spanning 4-Domains A4A
6679.	OTOL1	Otolin 1
6680.	PRPF40B	Pre-MRNA Processing Factor 40 Homolog B
6681.	PRRX2	Paired Related Homeobox 2
6682.	SPINK4	Serine Peptidase Inhibitor Kazal Type 4
6683.	TCIM	Transcriptional And Immune Response Regulator
6684.	TMEM218	Transmembrane Protein 218
6685.	ZBTB9	Zinc Finger And BTB Domain Containing 9
6686.	ZC3H4	Zinc Finger CCCH-Type Containing 4
6687.	ZNF395	Zinc Finger Protein 395
6688.	ARL13A	ADP Ribosylation Factor Like GTPase 13A
6689.	ARMS2	Age-Related Maculopathy Susceptibility 2
6690.	BRINP3	BMP/Retinoic Acid Inducible Neural Specific 3
6691.	C17orf78	Chromosome 17 Open Reading Frame 78
6692.	CDY1	Chromodomain Y-Linked 1
6693.	CHCHD5	Coiled-Coil-Helix-Coiled-Coil-Helix Domain Containing 5
6694.	DDIAS	DNA Damage Induced Apoptosis Suppressor
6695.	EPPK1	Epiplakin 1
6696.	FAM220A	Family With Sequence Similarity 220 Member A
6697.	H2AC8	H2A Clustered Histone 8
6698.	HEPACAM2	HEPACAM Family Member 2
6699.	HES4	Hes Family BHLH Transcription Factor 4
6700.	INTS14	Integrator Complex Subunit 14
6701.	LDAH	Lipid Droplet Associated Hydrolase
6702.	MALL	Mal, T Cell Differentiation Protein Like
6703.	MIX23	Mitochondrial Matrix Import Factor 23
6704.	MVB12A	Multivesicular Body Subunit 12A
6705.	NDUFAF8	NADH:Ubiquinone Oxidoreductase Complex Assembly Factor 8
6706.	OAZ3	Ornithine Decarboxylase Antizyme 3
6707.	OC90	Otoconin 90
6708.	OR52A1	Olfactory Receptor Family 52 Subfamily A Member 1
6709.	PASD1	PAS Domain Containing Repressor 1
6710.	PAXX	PAXX Non-Homologous End Joining Factor
6711.	PNRC2	Proline Rich Nuclear Receptor Coactivator 2
6712.	RPL39L	Ribosomal Protein L39 Like
6713.	SPAG7	Sperm Associated Antigen 7
6714.	SPATA33	Spermatogenesis Associated 33
6715.	SPINK13	Serine Peptidase Inhibitor Kazal Type 13
6716.	SYT10	Synaptotagmin 10
6717.	TCEAL7	Transcription Elongation Factor A Like 7

6718.	TLCD3A	TLC Domain Containing 3A
6719.	TUSC2	Tumor Suppressor 2, Mitochondrial Calcium Regulator
6720.	UQCC3	Ubiquinol-Cytochrome C Reductase Complex Assembly Factor 3
6721.	VPS9D1	VPS9 Domain Containing 1
6722.	YDJC	YdjC Chitooligosaccharide Deacetylase Homolog
6723.	ZNF14	Zinc Finger Protein 14
6724.	ASPDH	Aspartate Dehydrogenase Domain Containing
6725.	BEX1	Brain Expressed X-Linked 1
6726.	BEX2	Brain Expressed X-Linked 2
6727.	BICRAL	BICRA Like Chromatin Remodeling Complex Associated Protein
6728.	CENATAC	Centrosomal AT-AC Splicing Factor
6729.	CMC4	C-X9-C Motif Containing 4
6730.	CSNK2A3	Casein Kinase 2 Alpha 3
6731.	LINGO4	Leucine Rich Repeat And Ig Domain Containing 4
6732.	OCM	Oncomodulin
6733.	R3HDML	R3H Domain Containing Like
6734.	TAAR8	Trace Amine Associated Receptor 8
6735.	TTC9B	Tetratricopeptide Repeat Domain 9B
6736.	VGLL2	Vestigial Like Family Member 2
6737.	ZNF780A	Zinc Finger Protein 780A
6738.	ANKRD31	Ankyrin Repeat Domain 31
6739.	C2CD4A	C2 Calcium Dependent Domain Containing 4A
6740.	C2CD4B	C2 Calcium Dependent Domain Containing 4B
6741.	CSTPP1	Centriolar Satellite-Associated Tubulin Polyglutamylase Complex Regulator 1
6742.	NXPH2	Neurexophilin 2
6743.	OR4C16	Olfactory Receptor Family 4 Subfamily C Member 16
6744.	RGPD2	RANBP2 Like And GRIP Domain Containing 2
6745.	RIOX1	Ribosomal Oxygenase 1
6746.	SAMD13	Sterile Alpha Motif Domain Containing 13
6747.	TMEM45A	Transmembrane Protein 45A
6748.	TUSC1	Tumor Suppressor Candidate 1
6749.	ZNF383	Zinc Finger Protein 383
6750.	ZNF543	Zinc Finger Protein 543
6751.	ZNF845	Zinc Finger Protein 845
6752.	C12orf75	Chromosome 12 Open Reading Frame 75
6753.	CDRT15	CMT1A Duplicated Region Transcript 15
6754.	CIMAP1B	Ciliary Microtubule Associated Protein 1B
6755.	CIMIP1	Ciliary Microtubule Inner Protein 1
6756.	CT83	Cancer/Testis Antigen 83
6757.	DAZ2	Deleted In Azoospermia 2
6758.	DAZ3	Deleted In Azoospermia 3

6759.	DNAAF8	Dynein Axonemal Assembly Factor 8
6760.	HGH1	HGH1 Homolog
6761.	HSFY1	Heat Shock Transcription Factor Y-Linked 1
6762.	KRTAP4-2	Keratin Associated Protein 4-2
6763.	LNP1	Leukemia NUP98 Fusion Partner 1
6764.	PGA4	Pepsinogen A4
6765.	RBIS	Ribosomal Biogenesis Factor
6766.	DAZ4	Deleted In Azoospermia 4
6767.	EZHIP	EZH Inhibitory Protein
6768.	GARIN1B	Golgi Associated RAB2 Interactor 1B
6769.	INS-IGF2	INS-IGF2 Readthrough
6770.	OCM2	Oncomodulin 2
6771.	SLC15A5	Solute Carrier Family 15 Member 5
6772.	CDY2A	Chromodomain Y-Linked 2A
6773.	FBXW10B	F-Box And WD Repeat Domain Containing 10B
6774.	RPL41	Ribosomal Protein L41
6775.	SMIM14	Small Integral Membrane Protein 14
6776.	TRABD2B	TraB Domain Containing 2B
6777.	UGT2A2	UDP Glucuronosyltransferase Family 2 Member A2
6778.	ASCL5	Achaete-Scute Family BHLH Transcription Factor 5
6779.	AGAP6	ArfGAP With GTPase Domain, Ankyrin Repeat And PH Domain 6
6780.	BPY2	Basic Charge Y-Linked 2
6781.	JMJD7-PLA2G4B	JMJD7-PLA2G4B Readthrough
6782.	OR8U3	Olfactory Receptor Family 8 Subfamily U Member 3
6783.	PPAN-P2RY11	PPAN-P2RY11 Readthrough
6784.	XKR5	XK Related 5
6785.	MTLN	Mitoregulin
6786.	TPBGL	Trophoblast Glycoprotein Like
6787.	TRA	T Cell Receptor Alpha Locus
6788.	BPY2B	Basic Charge Y-Linked 2B
6789.	BPY2C	Basic Charge Y-Linked 2C
6790.	STRIT1	Small Transmembrane Regulator Of Ion Transport 1
6791.	USP17L13	Ubiquitin Specific Peptidase 17 Like Family Member 13
6792.	ETDA	Embryonic Testis Differentiation Homolog A
6793.	ETDC	Embryonic Testis Differentiation Homolog C
6794.	MSBP1	Minisatellite Binding Protein 1
6795.	CAPN1	Calpain 1
6796.	PSAT1	Phosphoserine Aminotransferase 1
6797.	HMBS	Hydroxymethylbilane Synthase
6798.	PPP3R1	Protein Phosphatase 3 Regulatory Subunit B, Alpha
6799.	ACP3	Acid Phosphatase 3

6800.	PTH2R	Parathyroid Hormone 2 Receptor
6801.	DHRS9	Dehydrogenase/Reductase 9
6802.	SNW1	SNW Domain Containing 1
6803.	BBOX1	Gamma-Butyrobetaine Hydroxylase 1
6804.	TLR10	Toll Like Receptor 10
6805.	DBNL	Drebrin Like
6806.	GNG2	G Protein Subunit Gamma 2
6807.	TRMT5	TRNA Methyltransferase 5
6808.	TMEM108	Transmembrane Protein 108
6809.	FGG	Fibrinogen Gamma Chain
6810.	SORD	Sorbitol Dehydrogenase
6811.	CA1	Carbonic Anhydrase 1
6812.	CA4	Carbonic Anhydrase 4
6813.	PAK4	P21 (RAC1) Activated Kinase 4
6814.	KLKB1	Kallikrein B1
6815.	NFIB	Nuclear Factor I B
6816.	ADRA1B	Adrenoceptor Alpha 1B
6817.	PTK7	Protein Tyrosine Kinase 7 (Inactive)
6818.	ADRA1D	Adrenoceptor Alpha 1D
6819.	NR2E3	Nuclear Receptor Subfamily 2 Group E Member 3
6820.	SH2B1	SH2B Adaptor Protein 1
6821.	AEBP1	AE Binding Protein 1
6822.	NISCH	Nischarin
6823.	PTPRR	Protein Tyrosine Phosphatase Receptor Type R
6824.	SLC16A7	Solute Carrier Family 16 Member 7
6825.	TAAR1	Trace Amine Associated Receptor 1
6826.	ANAPC2	Anaphase Promoting Complex Subunit 2
6827.	KLF15	KLF Transcription Factor 15
6828.	ZC3HAV1	Zinc Finger CCCH-Type Containing, Antiviral 1
6829.	BCAS3	BCAS3 Microtubule Associated Cell Migration Factor
6830.	CA7	Carbonic Anhydrase 7
6831.	CDCA5	Cell Division Cycle Associated 5
6832.	GABRG1	Gamma-Aminobutyric Acid Type A Receptor Subunit Gamma1
6833.	KIR2DL3	Killer Cell Immunoglobulin Like Receptor, Two Ig Domains And Long Cytoplasmic Tail 3
6834.	MYH13	Myosin Heavy Chain 13
6835.	OSBPL1A	Oxysterol Binding Protein Like 1A
6836.	SRL	Sarcalumenin
6837.	APOBEC3F	Apolipoprotein B MRNA Editing Enzyme Catalytic Subunit 3F
6838.	TNP2	Transition Protein 2
6839.	TMEM14C	Transmembrane Protein 14C
6840.	AMY1A	Amylase Alpha 1A

6841.	STATH	Statherin
6842.	AMY1C	Amylase Alpha 1C

**Supplementary Table S5.** Genes associated with photophobia that are extracted from GeneCards.		
**Index**	**Gene Symbol**	**Gene Full Name**
1.	MBTPS2	Membrane Bound Transcription Factor Peptidase, Site 2
2.	SREBF1	Sterol Regulatory Element Binding Transcription Factor 1
3.	GJB2	Gap Junction Protein Beta 2
4.	CNGA3	Cyclic Nucleotide Gated Channel Subunit Alpha 3
5.	CNGB3	Cyclic Nucleotide Gated Channel Subunit Beta 3
6.	RPGR	Retinitis Pigmentosa GTPase Regulator
7.	TYR	Tyrosinase
8.	GUCY2D	Guanylate Cyclase 2D, Retinal
9.	RPGRIP1	RPGR Interacting Protein 1
10.	ABCA4	ATP Binding Cassette Subfamily A Member 4
11.	CRX	Cone-Rod Homeobox
12.	PDE6C	Phosphodiesterase 6C
13.	GNAT2	G Protein Subunit Alpha Transducin 2
14.	OPN1MW	Opsin 1, Medium Wave Sensitive
15.	CABP4	Calcium Binding Protein 4
16.	CEP290	Centrosomal Protein 290
17.	RPE65	Retinoid Isomerohydrolase RPE65
18.	TULP1	TUB Like Protein 1
19.	LCA5	Lebercilin LCA5
20.	LRAT	Lecithin Retinol Acyltransferase
21.	NR2E3	Nuclear Receptor Subfamily 2 Group E Member 3
22.	OPN1LW	Opsin 1, Long Wave Sensitive
23.	AIPL1	Aryl Hydrocarbon Receptor Interacting Protein Like 1
24.	ATF6	Activating Transcription Factor 6
25.	CTNS	Cystinosin, Lysosomal Cystine Transporter
26.	CACNA1F	Calcium Voltage-Gated Channel Subunit Alpha1 F
27.	SPATA7	Spermatogenesis Associated 7
28.	RDH12	Retinol Dehydrogenase 12
29.	GUCA1A	Guanylate Cyclase Activator 1A
30.	KRT3	Keratin 3
31.	IQCB1	IQ Motif Containing B1
32.	ALMS1	ALMS1 Centrosome And Basal Body Associated Protein

33.	PRPH2	Peripherin 2
34.	RGS9	Regulator Of G Protein Signaling 9
35.	RGS9BP	Regulator Of G Protein Signaling 9 Binding Protein
36.	PAX6	Paired Box 6
37.	KCNV2	Potassium Voltage-Gated Channel Modifier Subfamily V Member 2
38.	NMNAT1	Nicotinamide Nucleotide Adenylyltransferase 1
39.	KRT12	Keratin 12
40.	PDE6B	Phosphodiesterase 6B
41.	IMPDH1	Inosine Monophosphate Dehydrogenase 1
42.	SLC24A5	Solute Carrier Family 24 Member 5
43.	RHO	Rhodopsin
44.	GPR143	G Protein-Coupled Receptor 143
45.	AP1B1	Adaptor Related Protein Complex 1 Subunit Beta 1
46.	CDHR1	Cadherin Related Family Member 1
47.	USH2A	Usherin
48.	CRB1	Crumbs Cell Polarity Complex Component 1
49.	GJB6	Gap Junction Protein Beta 6
50.	PDE6H	Phosphodiesterase 6H
51.	OCA2	OCA2 Melanosomal Transmembrane Protein
52.	TNF	Tumor Necrosis Factor
53.	EYS	Eyes Shut Homolog
54.	CACNA2D4	Calcium Voltage-Gated Channel Auxiliary Subunit Alpha2delta 4
55.	PDE6A	Phosphodiesterase 6A
56.	PITPNM3	PITPNM Family Member 3
57.	IMPG1	Interphotoreceptor Matrix Proteoglycan 1
58.	RD3	RD3 Regulator Of GUCY2D
59.	KCNJ13	Potassium Inwardly Rectifying Channel Subfamily J Member 13
60.	CNGB1	Cyclic Nucleotide Gated Channel Subunit Beta 1
61.	MKS1	MKS Transition Zone Complex Subunit 1
62.	BEST1	Bestrophin 1
63.	TGFBI	Transforming Growth Factor Beta Induced
64.	CNGA1	Cyclic Nucleotide Gated Channel Subunit Alpha 1
65.	CERKL	Ceramide Kinase Like
66.	RAB28	RAB28, Member RAS Oncogene Family
67.	LRMDA	Leucine Rich Melanocyte Differentiation Associated
68.	BBS1	Bardet-Biedl Syndrome 1
69.	PROM1	Prominin 1
70.	RDH5	Retinol Dehydrogenase 5
71.	RIMS2	Regulating Synaptic Membrane Exocytosis 2
72.	AHI1	Abelson Helper Integration Site 1
73.	SAG	S-Antigen Visual Arrestin

74.	POC1B	POC1 Centriolar Protein B
75.	IFT140	Intraflagellar Transport 140
76.	FAM161A	FAM161 Centrosomal Protein A
77.	RP1L1	RP1 Like 1
78.	PRPF31	Pre-MRNA Processing Factor 31
79.	CFAP410	Cilia And Flagella Associated Protein 410
80.	GUCA1B	Guanylate Cyclase Activator 1B
81.	CHST6	Carbohydrate Sulfotransferase 6
82.	SEMA4A	Semaphorin 4A
83.	NPHP4	Nephrocystin 4
84.	CFAP418	Cilia And Flagella Associated Protein 418
85.	RBP3	Retinol Binding Protein 3
86.	PIKFYVE	Phosphoinositide Kinase, FYVE-Type Zinc Finger Containing
87.	RLBP1	Retinaldehyde Binding Protein 1
88.	PCARE	Photoreceptor Cilium Actin Regulator
89.	CNNM4	Cyclin And CBS Domain Divalent Metal Cation Transport Mediator 4
90.	ARL3	ADP Ribosylation Factor Like GTPase 3
91.	MERTK	MER Proto-Oncogene, Tyrosine Kinase
92.	CACNA1A	Calcium Voltage-Gated Channel Subunit Alpha1 A
93.	NRL	Neural Retina Leucine Zipper
94.	WDR19	WD Repeat Domain 19
95.	CLN3	CLN3 Lysosomal/Endosomal Transmembrane Protein, Battenin
96.	TACSTD2	Tumor Associated Calcium Signal Transducer 2
97.	IMPG2	Interphotoreceptor Matrix Proteoglycan 2
98.	BBS10	Bardet-Biedl Syndrome 10
99.	BBS2	Bardet-Biedl Syndrome 2
100.	IFT172	Intraflagellar Transport 172
101.	BBS7	Bardet-Biedl Syndrome 7
102.	FOXC2	Forkhead Box C2
103.	RGR	Retinal G Protein Coupled Receptor
104.	UNC119	Unc-119 Lipid Binding Chaperone
105.	GNB3	G Protein Subunit Beta 3
106.	TYRP1	Tyrosinase Related Protein 1
107.	PRPF8	Pre-MRNA Processing Factor 8
108.	CEP78	Centrosomal Protein 78
109.	RIMS1	Regulating Synaptic Membrane Exocytosis 1
110.	BBS4	Bardet-Biedl Syndrome 4
111.	BBS9	Bardet-Biedl Syndrome 9
112.	BBS12	Bardet-Biedl Syndrome 12
113.	NLRP3	NLR Family Pyrin Domain Containing 3
114.	PRCD	Photoreceptor Disc Component

115.	CLRN1	Clarin 1
116.	PCDH15	Protocadherin Related 15
117.	MYO7A	Myosin VIIA
118.	RAX2	Retina And Anterior Neural Fold Homeobox 2
119.	RP2	RP2 Activator Of ARL3 GTPase
120.	GPHN	Gephyrin
121.	LYST	Lysosomal Trafficking Regulator
122.	PRPF3	Pre-MRNA Processing Factor 3
123.	TTLL5	Tubulin Tyrosine Ligase Like 5
124.	HLA-B	Major Histocompatibility Complex, Class I, B
125.	GRM6	Glutamate Metabotropic Receptor 6
126.	USP45	Ubiquitin Specific Peptidase 45
127.	CHM	CHM Rab Escort Protein
128.	SNRNP200	Small Nuclear Ribonucleoprotein U5 Subunit 200
129.	PDE6G	Phosphodiesterase 6G
130.	SLC24A1	Solute Carrier Family 24 Member 1
131.	CA4	Carbonic Anhydrase 4
132.	DRAM2	DNA Damage Regulated Autophagy Modulator 2
133.	AGBL5	AGBL Carboxypeptidase 5
134.	CST6	Cystatin E/M
135.	GUCA1ANB-GUCA1A	GUCA1ANB-GUCA1A Readthrough
136.	VSX2	Visual System Homeobox 2
137.	AP3D1	Adaptor Related Protein Complex 3 Subunit Delta 1
138.	MAK	Male Germ Cell Associated Kinase
139.	GNAT1	G Protein Subunit Alpha Transducin 1
140.	ZFYVE26	Zinc Finger FYVE-Type Containing 26
141.	DCN	Decorin
142.	ESR1	Estrogen Receptor 1
143.	CYP4V2	Cytochrome P450 Family 4 Subfamily V Member 2
144.	MFSD8	Major Facilitator Superfamily Domain Containing 8
145.	DHX38	DEAH-Box Helicase 38
146.	CC2D2A	Coiled-Coil And C2 Domain Containing 2A
147.	CDH23	Cadherin Related 23
148.	USH1C	USH1 Protein Network Component Harmonin
149.	SDCCAG8	SHH Signaling And Ciliogenesis Regulator SDCCAG8
150.	SLC38A8	Solute Carrier Family 38 Member 8
151.	ERCC2	ERCC Excision Repair 2, TFIIH Core Complex Helicase Subunit
152.	IL10	Interleukin 10
153.	TOPORS	TOP1 Binding Arginine/Serine Rich Protein, E3 Ubiquitin Ligase
154.	PRPF6	Pre-MRNA Processing Factor 6
155.	KCNK18	Potassium Two Pore Domain Channel Subfamily K Member 18

156.	MYEF2	Myelin Expression Factor 2
157.	SCN1A	Sodium Voltage-Gated Channel Alpha Subunit 1
158.	NPHP1	Nephrocystin 1
159.	HPS6	HPS6 Biogenesis Of Lysosomal Organelles Complex 2 Subunit 3
160.	ZDHHC24	Zinc Finger DHHC-Type Containing 24
161.	ADGRV1	Adhesion G Protein-Coupled Receptor V1
162.	INPP5E	Inositol Polyphosphate-5-Phosphatase E
163.	HPS3	HPS3 Biogenesis Of Lysosomal Organelles Complex 2 Subunit 1
164.	ROM1	Retinal Outer Segment Membrane Protein 1
165.	ARL6	ADP Ribosylation Factor Like GTPase 6
166.	COL18A1	Collagen Type XVIII Alpha 1 Chain
167.	AP3B1	Adaptor Related Protein Complex 3 Subunit Beta 1
168.	RPGRIP1L	RPGRIP1 Like
169.	HPS4	HPS4 Biogenesis Of Lysosomal Organelles Complex 3 Subunit 2
170.	CLUAP1	Clusterin Associated Protein 1
171.	TLR4	Toll Like Receptor 4
172.	ARL2BP	ADP Ribosylation Factor Like GTPase 2 Binding Protein
173.	RLIG1	RNA 5'-Phosphate And 3'-OH Ligase 1
174.	HPS1	HPS1 Biogenesis Of Lysosomal Organelles Complex 3 Subunit 1
175.	TLCD3B	TLC Domain Containing 3B
176.	PCNA	Proliferating Cell Nuclear Antigen
177.	ITM2B	Integral Membrane Protein 2B
178.	VPS13B	Vacuolar Protein Sorting 13 Homolog B
179.	ATP1A2	ATPase Na+/K+ Transporting Subunit Alpha 2
180.	PANK2	Pantothenate Kinase 2
181.	BBS5	Bardet-Biedl Syndrome 5
182.	HGSNAT	Heparan-Alpha-Glucosaminide N-Acetyltransferase
183.	RP1	RP1 Axonemal Microtubule Associated
184.	KIZ	Kizuna Centrosomal Protein
185.	ALDH3A2	Aldehyde Dehydrogenase 3 Family Member A2
186.	HK1	Hexokinase 1
187.	ATP5ME	ATP Synthase Membrane Subunit E
188.	CRYGS	Crystallin Gamma S
189.	BLOC1S5	Biogenesis Of Lysosomal Organelles Complex 1 Subunit 5
190.	COL17A1	Collagen Type XVII Alpha 1 Chain
191.	ERCC4	ERCC Excision Repair 4, Endonuclease Catalytic Subunit
192.	HPS5	HPS5 Biogenesis Of Lysosomal Organelles Complex 2 Subunit 2
193.	NOD2	Nucleotide Binding Oligomerization Domain Containing 2
194.	SCAPER	S-Phase Cyclin A Associated Protein In The ER
195.	BLOC1S6	Biogenesis Of Lysosomal Organelles Complex 1 Subunit 6
196.	XPC	XPC Complex Subunit, DNA Damage Recognition And Repair Factor

197.	CEP250	Centrosomal Protein 250
198.	ITGA4	Integrin Subunit Alpha 4
199.	OPA3	Outer Mitochondrial Membrane Lipid Metabolism Regulator OPA3
200.	MC1R	Melanocortin 1 Receptor
201.	OTX2	Orthodenticle Homeobox 2
202.	PPT1	Palmitoyl-Protein Thioesterase 1
203.	SAT1	Spermidine/Spermine N1-Acetyltransferase 1
204.	SLC19A1	Solute Carrier Family 19 Member 1
205.	PHF3	PHD Finger Protein 3
206.	ADAM9	ADAM Metallopeptidase Domain 9
207.	IDH3A	Isocitrate Dehydrogenase (NAD(+)) 3 Catalytic Subunit Alpha
208.	DHDDS	Dehydrodolichyl Diphosphate Synthase Subunit
209.	KLHL7	Kelch Like Family Member 7
210.	KIAA1549	KIAA1549
211.	KRTCAP3	Keratinocyte Associated Protein 3
212.	XPA	XPA, DNA Damage Recognition And Repair Factor
213.	LRP1	LDL Receptor Related Protein 1
214.	ERCC3	ERCC Excision Repair 3, TFIIH Core Complex Helicase Subunit
215.	GIGYF2	GRB10 Interacting GYF Protein 2
216.	FLVCR1	FLVCR Choline And Heme Transporter 1
217.	ADGRA3	Adhesion G Protein-Coupled Receptor A3
218.	MT-CO1	Mitochondrially Encoded Cytochrome C Oxidase I
219.	GDF6	Growth Differentiation Factor 6
220.	CEP164	Centrosomal Protein 164
221.	TTC21B	Tetratricopeptide Repeat Domain 21B
222.	NPHP3	Nephrocystin 3
223.	PCYT1A	Phosphate Cytidylyltransferase 1A, Choline
224.	DDB2	Damage Specific DNA Binding Protein 2
225.	KRT14	Keratin 14
226.	DTNBP1	Dystrobrevin Binding Protein 1
227.	MT-CYB	Mitochondrially Encoded Cytochrome B
228.	MEFV	MEFV Innate Immunity Regulator, Pyrin
229.	MT-ND1	Mitochondrially Encoded NADH:Ubiquinone Oxidoreductase Core Subunit 1
230.	ERCC1	ERCC Excision Repair 1, Endonuclease Non-Catalytic Subunit
231.	TRAF3IP1	TRAF3 Interacting Protein 1
232.	SLC45A2	Solute Carrier Family 45 Member 2
233.	NOTCH3	Notch Receptor 3
234.	TMEM67	Transmembrane Protein 67
235.	TGFB1	Transforming Growth Factor Beta 1
236.	BDNF	Brain Derived Neurotrophic Factor
237.	KIF11	Kinesin Family Member 11

238.	CDH3	Cadherin 3
239.	PHYH	Phytanoyl-CoA 2-Hydroxylase
240.	LARGE1	LARGE Xylosyl- And Glucuronyltransferase 1
241.	CYGB	Cytoglobin
242.	P3H2	Prolyl 3-Hydroxylase 2
243.	RCBTB1	RCC1 And BTB Domain Containing Protein 1
244.	MAPT	Microtubule Associated Protein Tau
245.	MT-ND6	Mitochondrially Encoded NADH:Ubiquinone Oxidoreductase Core Subunit 6
246.	TAT	Tyrosine Aminotransferase
247.	FAS	Fas Cell Surface Death Receptor
248.	NYX	Nyctalopin
249.	ERAP1	Endoplasmic Reticulum Aminopeptidase 1
250.	INVS	Inversin
251.	CDCA3	Cell Division Cycle Associated 3
252.	ST14	ST14 Transmembrane Serine Protease Matriptase
253.	SSBP1	Single Stranded DNA Binding Protein 1
254.	ABHD12	Abhydrolase Domain Containing 12, Lysophospholipase
255.	MT-ND4	Mitochondrially Encoded NADH:Ubiquinone Oxidoreductase Core Subunit 4
256.	GSN	Gelsolin
257.	EDNRA	Endothelin Receptor Type A
258.	ERCC5	ERCC Excision Repair 5, Endonuclease
259.	COL8A2	Collagen Type VIII Alpha 2 Chain
260.	TUBB4B	Tubulin Beta 4B Class IVb
261.	MKKS	MKKS Centrosomal Shuttling Protein
262.	OPN1SW	Opsin 1, Short Wave Sensitive
263.	MAOA	Monoamine Oxidase A
264.	ELOVL4	ELOVL Fatty Acid Elongase 4
265.	ZNF454	Zinc Finger Protein 454
266.	CYP1B1	Cytochrome P450 Family 1 Subfamily B Member 1
267.	SRD5A3	Steroid 5 Alpha-Reductase 3
268.	CBS	Cystathionine Beta-Synthase
269.	IFNGR1	Interferon Gamma Receptor 1
270.	NDUFS2	NADH:Ubiquinone Oxidoreductase Core Subunit S2
271.	FOXC1	Forkhead Box C1
272.	BRCA2	BRCA2 DNA Repair Associated
273.	OFD1	OFD1 Centriole And Centriolar Satellite Protein
274.	CP	Ceruloplasmin
275.	TTC8	Tetratricopeptide Repeat Domain 8
276.	MT-CO2	Mitochondrially Encoded Cytochrome C Oxidase II
277.	MT-ND2	Mitochondrially Encoded NADH:Ubiquinone Oxidoreductase Core Subunit 2
278.	MT-ATP6	Mitochondrially Encoded ATP Synthase Membrane Subunit 6

279.	MT-ND3	Mitochondrially Encoded NADH:Ubiquinone Oxidoreductase Core Subunit 3
280.	MT-ND5	Mitochondrially Encoded NADH:Ubiquinone Oxidoreductase Core Subunit 5
281.	MT-CO3	Mitochondrially Encoded Cytochrome C Oxidase III
282.	MT-ATP8	Mitochondrially Encoded ATP Synthase Membrane Subunit 8
283.	MT-ND4L	Mitochondrially Encoded NADH:Ubiquinone Oxidoreductase Core Subunit 4L
284.	ELP4	Elongator Acetyltransferase Complex Subunit 4
285.	AFG3L2	AFG3 Like Matrix AAA Peptidase Subunit 2
286.	C4A	Complement C4A (Chido/Rodgers Blood Group)
287.	RTN4IP1	Reticulon 4 Interacting Protein 1
288.	IL23R	Interleukin 23 Receptor
289.	PSTPIP1	Proline-Serine-Threonine Phosphatase Interacting Protein 1
290.	POLR1C	RNA Polymerase I And III Subunit C
291.	IL6	Interleukin 6
292.	OPN4	Opsin 4
293.	DCT	Dopachrome Tautomerase
294.	MYOC	Myocilin
295.	NOS3	Nitric Oxide Synthase 3
296.	SLC4A11	Solute Carrier Family 4 Member 11
297.	BIVM-ERCC5	BIVM-ERCC5 Readthrough
298.	TIMM8A	Translocase Of Inner Mitochondrial Membrane 8A
299.	TNFRSF1A	TNF Receptor Superfamily Member 1A
300.	MFRP	Membrane Frizzled-Related Protein
301.	LTBP2	Latent Transforming Growth Factor Beta Binding Protein 2
302.	RP9	RP9 Pre-MRNA Splicing Factor
303.	NDUFAF5	NADH:Ubiquinone Oxidoreductase Complex Assembly Factor 5
304.	PRICKLE3	Prickle Planar Cell Polarity Protein 3
305.	CFH	Complement Factor H
306.	IFNG	Interferon Gamma
307.	POLH	DNA Polymerase Eta
308.	TRIM44	Tripartite Motif Containing 44
309.	ALB	Albumin
310.	VSX1	Visual System Homeobox 1
311.	FSCN2	Fascin Actin-Bundling Protein 2, Retinal
312.	ZNF513	Zinc Finger Protein 513
313.	IKZF1	IKAROS Family Zinc Finger 1
314.	STAT4	Signal Transducer And Activator Of Transcription 4
315.	CCR1	C-C Motif Chemokine Receptor 1
316.	IL12A	Interleukin 12A
317.	UBAC2	UBA Domain Containing 2
318.	KLRC4	Killer Cell Lectin Like Receptor C4
319.	PPOX	Protoporphyrinogen Oxidase

320.	WT1	WT1 Transcription Factor
321.	GRK1	G Protein-Coupled Receptor Kinase 1
322.	CD63	CD63 Molecule
323.	EFEMP1	EGF Containing Fibulin Extracellular Matrix Protein 1
324.	ERCC6	ERCC Excision Repair 6, Chromatin Remodeling Factor
325.	ERCC8	ERCC Excision Repair 8, CSA Ubiquitin Ligase Complex Subunit
326.	REEP6	Receptor Accessory Protein 6
327.	WDR45	WD Repeat Domain 45
328.	SLC1A3	Solute Carrier Family 1 Member 3
329.	IFT27	Intraflagellar Transport 27
330.	AGT	Angiotensinogen
331.	HPD	4-Hydroxyphenylpyruvate Dioxygenase
332.	CHRDL1	Chordin Like 1
333.	IFT88	Intraflagellar Transport 88
334.	MMACHC	Metabolism Of Cobalamin Associated C
335.	PRPF4	Pre-MRNA Splicing Tri-SnRNP Complex Factor PRPF4
336.	IFT74	Intraflagellar Transport 74
337.	CRP	C-Reactive Protein
338.	UBIAD1	UbiA Prenyltransferase Domain Containing 1
339.	TP63	Tumor Protein P63
340.	HLA-DRB1	Major Histocompatibility Complex, Class II, DR Beta 1
341.	TRPM1	Transient Receptor Potential Cation Channel Subfamily M Member 1
342.	HCN1	Hyperpolarization Activated Cyclic Nucleotide Gated Potassium Channel 1
343.	OVOL2	Ovo Like Zinc Finger 2
344.	IL4	Interleukin 4
345.	ATXN7	Ataxin 7
346.	WDPCP	WD Repeat Containing Planar Cell Polarity Effector
347.	AIRE	Autoimmune Regulator
348.	LZTFL1	Leucine Zipper Transcription Factor Like 1
349.	TFAP2A	Transcription Factor AP-2 Alpha
350.	CEP19	Centrosomal Protein 19
351.	DKK1	Dickkopf WNT Signaling Pathway Inhibitor 1
352.	GRK7	G Protein-Coupled Receptor Kinase 7
353.	SCLT1	Sodium Channel And Clathrin Linker 1
354.	CD4	CD4 Molecule
355.	ARR3	Arrestin 3
356.	TAB2	TGF-Beta Activated Kinase 1 (MAP3K7) Binding Protein 2
357.	CAPN3	Calpain 3
358.	GATM	Glycine Amidinotransferase
359.	CLCN1	Chloride Voltage-Gated Channel 1
360.	ADA2	Adenosine Deaminase 2

361.	ITPR1	Inositol 1,4,5-Trisphosphate Receptor Type 1
362.	CPAMD8	C3 And PZP Like Alpha-2-Macroglobulin Domain Containing 8
363.	IL1B	Interleukin 1 Beta
364.	MCOLN1	Mucolipin TRP Cation Channel 1
365.	FRMD7	FERM Domain Containing 7
366.	CHD7	Chromodomain Helicase DNA Binding Protein 7
367.	ALS2	Alsin Rho Guanine Nucleotide Exchange Factor ALS2
368.	TUG1	Taurine Up-Regulated 1
369.	IL17A	Interleukin 17A
370.	IDH3B	Isocitrate Dehydrogenase (NAD(+)) 3 Non-Catalytic Subunit Beta
371.	RCVRN	Recoverin
372.	MTHFR	Methylenetetrahydrofolate Reductase
373.	FLT1	Fms Related Receptor Tyrosine Kinase 1
374.	ACO2	Aconitase 2
375.	PXDN	Peroxidasin
376.	YME1L1	YME1 Like 1 ATPase
377.	MCAT	Malonyl-CoA-Acyl Carrier Protein Transacylase
378.	GTF2H5	General Transcription Factor IIH Subunit 5
379.	HLA-A	Major Histocompatibility Complex, Class I, A
380.	BLOC1S3	Biogenesis Of Lysosomal Organelles Complex 1 Subunit 3
381.	MPLKIP	M-Phase Specific PLK1 Interacting Protein
382.	IFT122	Intraflagellar Transport 122
383.	PNPLA6	Patatin Like Phospholipase Domain Containing 6
384.	CDS1	CDP-Diacylglycerol Synthase 1
385.	CNGA2	Cyclic Nucleotide Gated Channel Subunit Alpha 2
386.	GUCY2F	Guanylate Cyclase 2F, Retinal
387.	CD8A	CD8 Subunit Alpha
388.	CCL2	C-C Motif Chemokine Ligand 2
389.	FOXL1	Forkhead Box L1
390.	NLRP1	NLR Family Pyrin Domain Containing 1
391.	NXNL1	Nucleoredoxin Like 1
392.	COMT	Catechol-O-Methyltransferase
393.	GUSB	Glucuronidase Beta
394.	IL2	Interleukin 2
395.	GNGT1	G Protein Subunit Gamma Transducin 1
396.	ZNF408	Zinc Finger Protein 408
397.	KCNB1	Potassium Voltage-Gated Channel Subfamily B Member 1
398.	GUCA2A	Guanylate Cyclase Activator 2A
399.	KIF3B	Kinesin Family Member 3B
400.	KIAA0586	KIAA0586
401.	GFAP	Glial Fibrillary Acidic Protein

402.	RS1	Retinoschisin 1
403.	AGBL1	AGBL Carboxypeptidase 1
404.	C1QTNF5	C1q And TNF Related 5
405.	CNGA4	Cyclic Nucleotide Gated Channel Subunit Alpha 4
406.	GPR179	G Protein-Coupled Receptor 179
407.	TRPV3	Transient Receptor Potential Cation Channel Subfamily V Member 3
408.	CNTF	Ciliary Neurotrophic Factor
409.	POMGNT1	Protein O-Linked Mannose N-Acetylglucosaminyltransferase 1 (Beta 1,2-)
410.	CXCL8	C-X-C Motif Chemokine Ligand 8
411.	OCRL	OCRL Inositol Polyphosphate-5-Phosphatase
412.	PITPNM1	Phosphatidylinositol Transfer Protein Membrane Associated 1
413.	RCC1L	RCC1 Like
414.	TLR2	Toll Like Receptor 2
415.	RAB8A	RAB8A, Member RAS Oncogene Family
416.	LAMP1	Lysosomal Associated Membrane Protein 1
417.	WHRN	Whirlin
418.	CUBN	Cubilin
419.	DMD	Dystrophin
420.	CALCA	Calcitonin Related Polypeptide Alpha
421.	PRL	Prolactin
422.	RDH11	Retinol Dehydrogenase 11
423.	RDH8	Retinol Dehydrogenase 8
424.	B9D1	B9 Domain Containing 1
425.	CSNK1D	Casein Kinase 1 Delta
426.	RAB11A	RAB11A, Member RAS Oncogene Family
427.	PDE6D	Phosphodiesterase 6D
428.	USH1G	USH1 Protein Network Component Sans
429.	SCN2A	Sodium Voltage-Gated Channel Alpha Subunit 2
430.	IL1A	Interleukin 1 Alpha
431.	TMEM231	Transmembrane Protein 231
432.	IFT52	Intraflagellar Transport 52
433.	EPHA2	EPH Receptor A2
434.	TEK	TEK Receptor Tyrosine Kinase
435.	ACTG1	Actin Gamma 1
436.	FZD5	Frizzled Class Receptor 5
437.	ABCB6	ATP Binding Cassette Subfamily B Member 6 (LAN Blood Group)
438.	CORIN	Corin, Serine Peptidase
439.	NSD1	Nuclear Receptor Binding SET Domain Protein 1
440.	SALL2	Spalt Like Transcription Factor 2
441.	STOX1	Storkhead Box 1
442.	ZNF354A	Zinc Finger Protein 354A

443.	LIN54	Lin-54 DREAM MuvB Core Complex Component
444.	PRR12	Proline Rich 12
445.	USP50	Ubiquitin Specific Peptidase 50
446.	FRG2C	FSHD Region Gene 2 Family Member C
447.	TSTD3	Thiosulfate Sulfurtransferase Like Domain Containing 3
448.	SLC6A4	Solute Carrier Family 6 Member 4
449.	MYO5A	Myosin VA
450.	APOE	Apolipoprotein E
451.	TRPV1	Transient Receptor Potential Cation Channel Subfamily V Member 1
452.	ASTN2	Astrotactin 2
453.	ERVW-1	Endogenous Retrovirus Group W Member 1, Envelope
454.	TTLL10	Tubulin Tyrosine Ligase Like 10
455.	GUCA1C	Guanylate Cyclase Activator 1C
456.	IL13	Interleukin 13
457.	LTF	Lactotransferrin
458.	CNTLN	Centlein
459.	SLC7A14	Solute Carrier Family 7 Member 14
460.	MPO	Myeloperoxidase
461.	SLC4A4	Solute Carrier Family 4 Member 4
462.	IL18	Interleukin 18
463.	COL4A1	Collagen Type IV Alpha 1 Chain
464.	SERPINF1	Serpin Family F Member 1
465.	MMP9	Matrix Metallopeptidase 9
466.	KIF3A	Kinesin Family Member 3A
467.	CCP110	Centriolar Coiled-Coil Protein 110
468.	ARL13B	ADP Ribosylation Factor Like GTPase 13B
469.	B9D2	B9 Domain Containing 2
470.	IFT57	Intraflagellar Transport 57
471.	IFT80	Intraflagellar Transport 80
472.	CEP83	Centrosomal Protein 83
473.	IFT20	Intraflagellar Transport 20
474.	TMEM216	Transmembrane Protein 216
475.	HTR2A	5-Hydroxytryptamine Receptor 2A
476.	PRF1	Perforin 1
477.	MLPH	Melanophilin
478.	SDR9C7	Short Chain Dehydrogenase/Reductase Family 9C Member 7
479.	TGM1	Transglutaminase 1
480.	GPT	Glutamic--Pyruvic Transaminase
481.	BHLHE22	Basic Helix-Loop-Helix Family Member E22
482.	IFT81	Intraflagellar Transport 81
483.	DNAH8	Dynein Axonemal Heavy Chain 8

484.	ACE	Angiotensin I Converting Enzyme
485.	RAB27A	RAB27A, Member RAS Oncogene Family
486.	LRIT3	Leucine Rich Repeat, Ig-Like And Transmembrane Domains 3
487.	ARMS2	Age-Related Maculopathy Susceptibility 2
488.	HTR2C	5-Hydroxytryptamine Receptor 2C
489.	GDNF	Glial Cell Derived Neurotrophic Factor
490.	DRD3	Dopamine Receptor D3
491.	IL5	Interleukin 5
492.	LRP2	LDL Receptor Related Protein 2
493.	CCL5	C-C Motif Chemokine Ligand 5
494.	KERA	Keratocan
495.	PRSS56	Serine Protease 56
496.	HMGCL	3-Hydroxy-3-Methylglutaryl-CoA Lyase
497.	PTCH1	Patched 1
498.	POMC	Proopiomelanocortin
499.	IFNA1	Interferon Alpha 1
500.	MITF	Melanocyte Inducing Transcription Factor
501.	CALB1	Calbindin 1
502.	HSPD1	Heat Shock Protein Family D (Hsp60) Member 1
503.	CFI	Complement Factor I
504.	TP53	Tumor Protein P53
505.	TCTN1	Tectonic Family Member 1
506.	CEP97	Centrosomal Protein 97
507.	ANK2	Ankyrin 2
508.	ICAM1	Intercellular Adhesion Molecule 1
509.	C3	Complement C3
510.	GNB5	G Protein Subunit Beta 5
511.	CFP	Complement Factor Properdin
512.	GNB1	G Protein Subunit Beta 1
513.	SOD1	Superoxide Dismutase 1
514.	KCNA3	Potassium Voltage-Gated Channel Subfamily A Member 3
515.	GUF1	GTP Binding Elongation Factor GUF1
516.	STXBP2	Syntaxin Binding Protein 2
517.	ACTB	Actin Beta
518.	C2CD3	C2 Domain Containing 3 Centriole Elongation Regulator
519.	DYNC2H1	Dynein Cytoplasmic 2 Heavy Chain 1
520.	KIF17	Kinesin Family Member 17
521.	RAB3IP	RAB3A Interacting Protein
522.	RDH10	Retinol Dehydrogenase 10
523.	CCDC28B	Coiled-Coil Domain Containing 28B
524.	EGFLAM	EGF Like, Fibronectin Type III And Laminin G Domains

525.	CROCC	Ciliary Rootlet Coiled-Coil, Rootletin
526.	HTR3A	5-Hydroxytryptamine Receptor 3A
527.	TUB	TUB Bipartite Transcription Factor
528.	SLC6A3	Solute Carrier Family 6 Member 3
529.	WFS1	Wolframin ER Transmembrane Glycoprotein
530.	MBL2	Mannose Binding Lectin 2
531.	TLR9	Toll Like Receptor 9
532.	ASRGL1	Asparaginase And Isoaspartyl Peptidase 1
533.	SLC24A4	Solute Carrier Family 24 Member 4
534.	ENO1	Enolase 1
535.	PRTN3	Proteinase 3
536.	FAM20A	FAM20A Golgi Associated Secretory Pathway Pseudokinase
537.	RABIF	RAB Interacting Factor
538.	SNCA	Synuclein Alpha
539.	DRD2	Dopamine Receptor D2
540.	ADA	Adenosine Deaminase
541.	POU5F1	POU Class 5 Homeobox 1
542.	SMAD9	SMAD Family Member 9
543.	PMEL	Premelanosome Protein
544.	SIX3	SIX Homeobox 3
545.	CRB2	Crumbs Cell Polarity Complex Component 2
546.	SIX6	SIX Homeobox 6
547.	ATOH7	Atonal BHLH Transcription Factor 7
548.	CD40LG	CD40 Ligand
549.	CASP1	Caspase 1
550.	CACNA1D	Calcium Voltage-Gated Channel Subunit Alpha1 D
551.	CSF3	Colony Stimulating Factor 3
552.	STRA6	Signaling Receptor And Transporter Of Retinol STRA6
553.	OTX1	Orthodenticle Homeobox 1
554.	PDZD7	PDZ Domain Containing 7
555.	ZEB1	Zinc Finger E-Box Binding Homeobox 1
556.	HARS1	Histidyl-TRNA Synthetase 1
557.	DDB1	Damage Specific DNA Binding Protein 1
558.	NGF	Nerve Growth Factor
559.	STS	Steroid Sulfatase
560.	FGF2	Fibroblast Growth Factor 2
561.	TIMP3	TIMP Metallopeptidase Inhibitor 3
562.	UNC13D	Unc-13 Homolog D
563.	STX11	Syntaxin 11
564.	LOXHD1	Lipoxygenase Homology PLAT Domains 1
565.	STX19	Syntaxin 19

566.	CCR5	C-C Motif Chemokine Receptor 5
567.	ARRB2	Arrestin Beta 2
568.	CNNM2	Cyclin And CBS Domain Divalent Metal Cation Transport Mediator 2
569.	PRKN	Parkin RBR E3 Ubiquitin Protein Ligase
570.	GJA1	Gap Junction Protein Alpha 1
571.	AARS1	Alanyl-TRNA Synthetase 1
572.	TRIM32	Tripartite Motif Containing 32
573.	CLCC1	Chloride Channel CLIC Like 1
574.	CETN2	Centrin 2
575.	VAX2	Ventral Anterior Homeobox 2
576.	TEX28	Testis Expressed 28
577.	ZNF449	Zinc Finger Protein 449
578.	PRKCA	Protein Kinase C Alpha
579.	ALDH7A1	Aldehyde Dehydrogenase 7 Family Member A1
580.	SORD	Sorbitol Dehydrogenase
581.	RORB	RAR Related Orphan Receptor B
582.	MPDZ	Multiple PDZ Domain Crumbs Cell Polarity Complex Component
583.	CTBP2	C-Terminal Binding Protein 2
584.	CIB2	Calcium And Integrin Binding Family Member 2
585.	RBP1	Retinol Binding Protein 1
586.	POU4F2	POU Class 4 Homeobox 2
587.	PDC	Phosducin
588.	LRIT1	Leucine Rich Repeat, Ig-Like And Transmembrane Domains 1
589.	AKT1	AKT Serine/Threonine Kinase 1
590.	GRIN2B	Glutamate Ionotropic Receptor NMDA Type Subunit 2B
591.	SHROOM2	Shroom Family Member 2
592.	F2	Coagulation Factor II, Thrombin
593.	F3	Coagulation Factor III, Tissue Factor
594.	KCNA1	Potassium Voltage-Gated Channel Subfamily A Member 1
595.	ZNF469	Zinc Finger Protein 469
596.	CACNA1S	Calcium Voltage-Gated Channel Subunit Alpha1 S
597.	FASLG	Fas Ligand
598.	VWF	Von Willebrand Factor
599.	CLCN5	Chloride Voltage-Gated Channel 5
600.	KLK4	Kallikrein Related Peptidase 4
601.	CDH17	Cadherin 17
602.	MMP20	Matrix Metallopeptidase 20
603.	CCL3	C-C Motif Chemokine Ligand 3
604.	PRRT2	Proline Rich Transmembrane Protein 2
605.	LRBA	LPS Responsive Beige-Like Anchor Protein
606.	DLX3	Distal-Less Homeobox 3

607.	ENAM	Enamelin
608.	WDR72	WD Repeat Domain 72
609.	AMELX	Amelogenin X-Linked
610.	CNNM3	Cyclin And CBS Domain Divalent Metal Cation Transport Mediator 3
611.	FAM83H	Family With Sequence Similarity 83 Member H
612.	ODAPH	Odontogenesis Associated Phosphoprotein
613.	POLA1	DNA Polymerase Alpha 1, Catalytic Subunit
614.	INS	Insulin
615.	PAX3	Paired Box 3
616.	TSC2	TSC Complex Subunit 2
617.	NF1	Neurofibromin 1
618.	PDE5A	Phosphodiesterase 5A
619.	OGG1	8-Oxoguanine DNA Glycosylase
620.	XRCC1	X-Ray Repair Cross Complementing 1
621.	CUL4A	Cullin 4A
622.	GRIA1	Glutamate Ionotropic Receptor AMPA Type Subunit 1
623.	CD55	CD55 Molecule (Cromer Blood Group)
624.	CSF2	Colony Stimulating Factor 2
625.	GALC	Galactosylceramidase
626.	SST	Somatostatin
627.	CCL11	C-C Motif Chemokine Ligand 11
628.	PITPNB	Phosphatidylinositol Transfer Protein Beta
629.	WRAP73	WD Repeat Containing, Antisense To TP73
630.	ELFN1	Extracellular Leucine Rich Repeat And Fibronectin Type III Domain Containing 1
631.	SCN9A	Sodium Voltage-Gated Channel Alpha Subunit 9
632.	TFEB	Transcription Factor EB
633.	CLN8	CLN8 Transmembrane ER And ERGIC Protein
634.	PVALB	Parvalbumin
635.	RAB3GAP1	RAB3 GTPase Activating Protein Catalytic Subunit 1
636.	OTOF	Otoferlin
637.	GPBAR1	G Protein-Coupled Bile Acid Receptor 1
638.	RTBDN	Retbindin
639.	DZANK1	Double Zinc Ribbon And Ankyrin Repeat Domains 1
640.	HADHA	Hydroxyacyl-CoA Dehydrogenase Trifunctional Multienzyme Complex Subunit Alpha
641.	WNT10A	Wnt Family Member 10A
642.	ARHGEF18	Rho/Rac Guanine Nucleotide Exchange Factor 18
643.	HTR1B	5-Hydroxytryptamine Receptor 1B
644.	PKD2	Polycystin 2, Transient Receptor Potential Cation Channel
645.	HTR1A	5-Hydroxytryptamine Receptor 1A
646.	LEP	Leptin
647.	DRD4	Dopamine Receptor D4

648.	MAOB	Monoamine Oxidase B
649.	HLA-DQB1	Major Histocompatibility Complex, Class II, DQ Beta 1
650.	OXT	Oxytocin/Neurophysin I Prepropeptide
651.	FURIN	Furin, Paired Basic Amino Acid Cleaving Enzyme
652.	LCAT	Lecithin-Cholesterol Acyltransferase
653.	LAMP2	Lysosomal Associated Membrane Protein 2
654.	NEK9	NIMA Related Kinase 9
655.	CST3	Cystatin C
656.	SLC34A1	Solute Carrier Family 34 Member 1
657.	NEK8	NIMA Related Kinase 8
658.	IFT43	Intraflagellar Transport 43
659.	TCTN2	Tectonic Family Member 2
660.	TCTN3	Tectonic Family Member 3
661.	CRYBA1	Crystallin Beta A1
662.	PRIMPOL	Primase And DNA Directed Polymerase
663.	SLC66A1	Solute Carrier Family 66 Member 1
664.	STAT1	Signal Transducer And Activator Of Transcription 1
665.	LIG1	DNA Ligase 1
666.	APEX1	Apurinic/Apyrimidinic Endodeoxyribonuclease 1
667.	RAD23B	RAD23 Homolog B, Nucleotide Excision Repair Protein
668.	CRYGC	Crystallin Gamma C
669.	SHPK	Sedoheptulokinase
670.	XIAP	X-Linked Inhibitor Of Apoptosis
671.	CA2	Carbonic Anhydrase 2
672.	IL2RA	Interleukin 2 Receptor Subunit Alpha
673.	GLA	Galactosidase Alpha
674.	PLAT	Plasminogen Activator, Tissue Type
675.	SH2D1A	SH2 Domain Containing 1A
676.	FHL2	Four And A Half LIM Domains 2
677.	TTBK2	Tau Tubulin Kinase 2
678.	ARF4	ADP Ribosylation Factor 4
679.	EDEM1	ER Degradation Enhancing Alpha-Mannosidase Like Protein 1
680.	NBEAL2	Neurobeachin Like 2
681.	KIFAP3	Kinesin Associated Protein 3
682.	WDR35	WD Repeat Domain 35
683.	GJD2	Gap Junction Protein Delta 2
684.	IFT22	Intraflagellar Transport 22
685.	NTRK2	Neurotrophic Receptor Tyrosine Kinase 2
686.	ACE2	Angiotensin Converting Enzyme 2
687.	FOXP2	Forkhead Box P2
688.	IFNB1	Interferon Beta 1

689.	GJC2	Gap Junction Protein Gamma 2
690.	CNR1	Cannabinoid Receptor 1
691.	HYLS1	HYLS1 Centriolar And Ciliogenesis Associated
692.	NINL	Ninein Like
693.	PRDM13	PR/SET Domain 13
694.	DTHD1	Death Domain Containing 1
695.	HTR1D	5-Hydroxytryptamine Receptor 1D
696.	GTF2H1	General Transcription Factor IIH Subunit 1
697.	UVSSA	UV Stimulated Scaffold Protein A
698.	CEP104	Centrosomal Protein 104
699.	ATP1A3	ATPase Na+/K+ Transporting Subunit Alpha 3
700.	CKB	Creatine Kinase B
701.	MAF	MAF BZIP Transcription Factor
702.	SLC17A5	Solute Carrier Family 17 Member 5
703.	HLA-DQA1	Major Histocompatibility Complex, Class II, DQ Alpha 1
704.	CLTB	Clathrin Light Chain B
705.	BLOC1S2	Biogenesis Of Lysosomal Organelles Complex 1 Subunit 2
706.	C9	Complement C9
707.	CNTNAP2	Contactin Associated Protein 2
708.	DBH	Dopamine Beta-Hydroxylase
709.	MTOR	Mechanistic Target Of Rapamycin Kinase
710.	NFKB1	Nuclear Factor Kappa B Subunit 1
711.	APP	Amyloid Beta Precursor Protein
712.	SLC1A2	Solute Carrier Family 1 Member 2
713.	SCN8A	Sodium Voltage-Gated Channel Alpha Subunit 8
714.	CYP2D6	Cytochrome P450 Family 2 Subfamily D Member 6
715.	SYNGAP1	Synaptic Ras GTPase Activating Protein 1
716.	STX3	Syntaxin 3
717.	CERS3	Ceramide Synthase 3
718.	HCRT	Hypocretin Neuropeptide Precursor
719.	GTF2H2	General Transcription Factor IIH Subunit 2
720.	SQSTM1	Sequestosome 1
721.	ADRB2	Adrenoceptor Beta 2
722.	GH1	Growth Hormone 1
723.	MBTPS1	Membrane Bound Transcription Factor Peptidase, Site 1
724.	RCC1	Regulator Of Chromosome Condensation 1
725.	ARHGEF4	Rho Guanine Nucleotide Exchange Factor 4
726.	ARL2	ADP Ribosylation Factor Like GTPase 2
727.	GNRH1	Gonadotropin Releasing Hormone 1
728.	TAC1	Tachykinin Precursor 1
729.	LUM	Lumican

730.	NIPAL4	NIPA Like Domain Containing 4
731.	PEX26	Peroxisomal Biogenesis Factor 26
732.	YBX3	Y-Box Binding Protein 3
733.	CPQ	Carboxypeptidase Q
734.	EMC1	ER Membrane Protein Complex Subunit 1
735.	DENND2B	DENN Domain Containing 2B
736.	PNPLA1	Patatin Like Phospholipase Domain Containing 1
737.	ATXN7L2	Ataxin 7 Like 2
738.	TRANK1	Tetratricopeptide Repeat And Ankyrin Repeat Containing 1
739.	BRCA1	BRCA1 DNA Repair Associated
740.	TBP	TATA-Box Binding Protein
741.	FEN1	Flap Structure-Specific Endonuclease 1
742.	HTRA1	HtrA Serine Peptidase 1
743.	MOG	Myelin Oligodendrocyte Glycoprotein
744.	XRCC6	X-Ray Repair Cross Complementing 6
745.	FMR1	Fragile X Messenger Ribonucleoprotein 1
746.	LIG3	DNA Ligase 3
747.	XRCC4	X-Ray Repair Cross Complementing 4
748.	SLC19A2	Solute Carrier Family 19 Member 2
749.	SOX2	SRY-Box Transcription Factor 2
750.	CGA	Glycoprotein Hormones, Alpha Polypeptide
751.	S100B	S100 Calcium Binding Protein B
752.	TMEM138	Transmembrane Protein 138
753.	C6orf15	Chromosome 6 Open Reading Frame 15
754.	GAN	Gigaxonin
755.	PPBP	Pro-Platelet Basic Protein
756.	ERF	ETS2 Repressor Factor
757.	RNASE3	Ribonuclease A Family Member 3
758.	FOS	Fos Proto-Oncogene, AP-1 Transcription Factor Subunit
759.	SMARCA2	SWI/SNF Related, Matrix Associated, Actin Dependent Regulator Of Chromatin, Subfamily A, Member 2
760.	IGF1	Insulin Like Growth Factor 1
761.	XRCC5	X-Ray Repair Cross Complementing 5
762.	TCEA1	Transcription Elongation Factor A1
763.	STAT3	Signal Transducer And Activator Of Transcription 3
764.	SHH	Sonic Hedgehog Signaling Molecule
765.	TH	Tyrosine Hydroxylase
766.	TRPA1	Transient Receptor Potential Cation Channel Subfamily A Member 1
767.	MVK	Mevalonate Kinase
768.	VEGFC	Vascular Endothelial Growth Factor C
769.	CYP2C19	Cytochrome P450 Family 2 Subfamily C Member 19
770.	FIG4	FIG4 Phosphoinositide 5-Phosphatase

771.	HLA-C	Major Histocompatibility Complex, Class I, C
772.	PCNT	Pericentrin
773.	CRH	Corticotropin Releasing Hormone
774.	ANK3	Ankyrin 3
775.	CD69	CD69 Molecule
776.	CFHR1	Complement Factor H Related 1
777.	GJA4	Gap Junction Protein Alpha 4
778.	VAC14	VAC14 Component Of PIKFYVE Complex
779.	TMEM126A	Transmembrane Protein 126A
780.	RBFOX3	RNA Binding Fox-1 Homolog 3
781.	TIMM22	Translocase Of Inner Mitochondrial Membrane 22
782.	TIMM8B	Translocase Of Inner Mitochondrial Membrane 8 Homolog B
783.	TIMM21	Translocase Of Inner Mitochondrial Membrane 21
784.	TBL1Y	Transducin Beta Like 1 Y-Linked
785.	BBIP1	BBSome Interacting Protein 1
786.	THRB	Thyroid Hormone Receptor Beta
787.	CTLA4	Cytotoxic T-Lymphocyte Associated Protein 4
788.	TTR	Transthyretin
789.	FES	FES Proto-Oncogene, Tyrosine Kinase
790.	KCNJ10	Potassium Inwardly Rectifying Channel Subfamily J Member 10
791.	RASA1	RAS P21 Protein Activator 1
792.	CARD9	Caspase Recruitment Domain Family Member 9
793.	APOH	Apolipoprotein H
794.	NEUROD1	Neuronal Differentiation 1
795.	NLRP12	NLR Family Pyrin Domain Containing 12
796.	ARSG	Arylsulfatase G
797.	RAB23	RAB23, Member RAS Oncogene Family
798.	CCL4	C-C Motif Chemokine Ligand 4
799.	NIN	Ninein
800.	ODF2	Outer Dense Fiber Of Sperm Tails 2
801.	PEX6	Peroxisomal Biogenesis Factor 6
802.	PLIN2	Perilipin 2
803.	RXRG	Retinoid X Receptor Gamma
804.	ADAMTS18	ADAM Metallopeptidase With Thrombospondin Type 1 Motif 18
805.	CERK	Ceramide Kinase
806.	SLC39A5	Solute Carrier Family 39 Member 5
807.	ZPR1	ZPR1 Zinc Finger
808.	CETN3	Centrin 3
809.	CWC27	CWC27 Spliceosome Associated Cyclophilin
810.	TAF9	TATA-Box Binding Protein Associated Factor 9
811.	TRIM8	Tripartite Motif Containing 8

812.	POU4F3	POU Class 4 Homeobox 3
813.	ZNF644	Zinc Finger Protein 644
814.	CYP20A1	Cytochrome P450 Family 20 Subfamily A Member 1
815.	INPP5F	Inositol Polyphosphate-5-Phosphatase F
816.	NMNAT3	Nicotinamide Nucleotide Adenylyltransferase 3
817.	ATXN7L1	Ataxin 7 Like 1
818.	CEP89	Centrosomal Protein 89
819.	RABAC1	Rab Acceptor 1
820.	TULP2	TUB Like Protein 2
821.	FICD	FIC Domain Protein Adenylyltransferase
822.	CCDC120	Coiled-Coil Domain Containing 120
823.	FAM161B	FAM161 Centrosomal Protein B
824.	ATXN7L3	Ataxin 7 Like 3
825.	MAP9	Microtubule Associated Protein 9
826.	FOXN4	Forkhead Box N4
827.	RGS7BP	Regulator Of G Protein Signaling 7 Binding Protein
828.	ATM	ATM Serine/Threonine Kinase
829.	PARP1	Poly(ADP-Ribose) Polymerase 1
830.	BRIP1	BRCA1 Interacting Helicase 1
831.	IL12RB1	Interleukin 12 Receptor Subunit Beta 1
832.	HSPA4	Heat Shock Protein Family A (Hsp70) Member 4
833.	HMGN1	High Mobility Group Nucleosome Binding Domain 1
834.	XAB2	XPA Binding Protein 2
835.	ERV3-1	Endogenous Retrovirus Group 3 Member 1, Envelope
836.	ERVFRD-1	Endogenous Retrovirus Group FRD Member 1, Envelope
837.	SHANK3	SH3 And Multiple Ankyrin Repeat Domains 3
838.	ADNP	Activity Dependent Neuroprotector Homeobox
839.	EGF	Epidermal Growth Factor
840.	GAPDH	Glyceraldehyde-3-Phosphate Dehydrogenase
841.	IL1RN	Interleukin 1 Receptor Antagonist
842.	SCN5A	Sodium Voltage-Gated Channel Alpha Subunit 5
843.	GATA2	GATA Binding Protein 2
844.	IFNAR1	Interferon Alpha And Beta Receptor Subunit 1
845.	TNFRSF1B	TNF Receptor Superfamily Member 1B
846.	CYP3A4	Cytochrome P450 Family 3 Subfamily A Member 4
847.	FOXP3	Forkhead Box P3
848.	SCN3A	Sodium Voltage-Gated Channel Alpha Subunit 3
849.	SCN4A	Sodium Voltage-Gated Channel Alpha Subunit 4
850.	SPTLC1	Serine Palmitoyltransferase Long Chain Base Subunit 1
851.	SCN11A	Sodium Voltage-Gated Channel Alpha Subunit 11
852.	TULP3	TUB Like Protein 3

853.	CLDN7	Claudin 7
854.	KIF7	Kinesin Family Member 7
855.	IFNA5	Interferon Alpha 5
856.	IFNA6	Interferon Alpha 6
857.	TMEM237	Transmembrane Protein 237
858.	IFNA14	Interferon Alpha 14
859.	IFNA21	Interferon Alpha 21
860.	IFNA8	Interferon Alpha 8
861.	CPLANE1	Ciliogenesis And Planar Polarity Effector Complex Subunit 1
862.	IFNA10	Interferon Alpha 10
863.	IFNA4	Interferon Alpha 4
864.	IFNA13	Interferon Alpha 13
865.	IFNA17	Interferon Alpha 17
866.	IFNA7	Interferon Alpha 7
867.	IFNA16	Interferon Alpha 16
868.	ARL13A	ADP Ribosylation Factor Like GTPase 13A
869.	NOS1	Nitric Oxide Synthase 1
870.	PTEN	Phosphatase And Tensin Homolog
871.	MECP2	Methyl-CpG Binding Protein 2
872.	KNG1	Kininogen 1
873.	FAH	Fumarylacetoacetate Hydrolase
874.	EDNRB	Endothelin Receptor Type B
875.	FN1	Fibronectin 1
876.	F9	Coagulation Factor IX
877.	PITX2	Paired Like Homeodomain 2
878.	HAL	Histidine Ammonia-Lyase
879.	LACTB	Lactamase Beta
880.	RAB38	RAB38, Member RAS Oncogene Family
881.	PI16	Peptidase Inhibitor 16
882.	SECTM1	Secreted And Transmembrane 1
883.	RAX	Retina And Anterior Neural Fold Homeobox
884.	KRT82	Keratin 82
885.	TF	Transferrin
886.	OTC	Ornithine Transcarbamylase
887.	GSS	Glutathione Synthetase
888.	HBB	Hemoglobin Subunit Beta
889.	GC	GC Vitamin D Binding Protein
890.	VNN1	Vanin 1
891.	OPLAH	5-Oxoprolinase, ATP-Hydrolysing
892.	PIAS3	Protein Inhibitor Of Activated STAT 3
893.	SARM1	Sterile Alpha And TIR Motif Containing 1

894.	ITGAE	Integrin Subunit Alpha E
895.	GAST	Gastrin
896.	LMBR1	Limb Development Membrane Protein 1
897.	VNN2	Vanin 2
898.	CLPS	Colipase
899.	NMNAT2	Nicotinamide Nucleotide Adenylyltransferase 2
900.	P2RX5	Purinergic Receptor P2X 5
901.	PALS1	Protein Associated With LIN7 1, MAGUK P55 Family Member
902.	SCIN	Scinderin
903.	TAX1BP3	Tax1 Binding Protein 3
904.	RDH14	Retinol Dehydrogenase 14
905.	TENT5A	Terminal Nucleotidyltransferase 5A
906.	TMEM147	Transmembrane Protein 147
907.	MFSD12	Major Facilitator Superfamily Domain Containing 12
908.	FRYL	FRY Like Transcription Coactivator
909.	GRIN2A	Glutamate Ionotropic Receptor NMDA Type Subunit 2A
910.	CREB1	CAMP Responsive Element Binding Protein 1
911.	DDC	Dopa Decarboxylase
912.	PRKDC	Protein Kinase, DNA-Activated, Catalytic Subunit
913.	RAD51	RAD51 Recombinase
914.	CACNA1C	Calcium Voltage-Gated Channel Subunit Alpha1 C
915.	SNAP25	Synaptosome Associated Protein 25
916.	HPRT1	Hypoxanthine Phosphoribosyltransferase 1
917.	TRPV4	Transient Receptor Potential Cation Channel Subfamily V Member 4
918.	GRM5	Glutamate Metabotropic Receptor 5
919.	CCNH	Cyclin H
920.	CDK7	Cyclin Dependent Kinase 7
921.	CYP1A2	Cytochrome P450 Family 1 Subfamily A Member 2
922.	DRD1	Dopamine Receptor D1
923.	HELLS	Helicase, Lymphoid Specific
924.	TP53BP1	Tumor Protein P53 Binding Protein 1
925.	RAD23A	RAD23 Homolog A, Nucleotide Excision Repair Protein
926.	ABCA12	ATP Binding Cassette Subfamily A Member 12
927.	NHEJ1	Non-Homologous End Joining Factor 1
928.	RAD52	RAD52 Homolog, DNA Repair Protein
929.	DTL	Denticleless E3 Ubiquitin Protein Ligase Homolog
930.	GTF2H4	General Transcription Factor IIH Subunit 4
931.	POU3F4	POU Class 3 Homeobox 4
932.	GTF2H3	General Transcription Factor IIH Subunit 3
933.	TRPM6	Transient Receptor Potential Cation Channel Subfamily M Member 6
934.	GRHL2	Grainyhead Like Transcription Factor 2

935.	BTK	Bruton Tyrosine Kinase
936.	EPHB4	EPH Receptor B4
937.	FLT4	Fms Related Receptor Tyrosine Kinase 4
938.	EDN1	Endothelin 1
939.	NR2F2	Nuclear Receptor Subfamily 2 Group F Member 2
940.	ACHE	Acetylcholinesterase (Yt Blood Group)
941.	NFATC1	Nuclear Factor Of Activated T Cells 1
942.	CLDN1	Claudin 1
943.	PAX2	Paired Box 2
944.	THBD	Thrombomodulin
945.	ANK1	Ankyrin 1
946.	CD34	CD34 Molecule
947.	DRD5	Dopamine Receptor D5
948.	IDUA	Alpha-L-Iduronidase
949.	PTH	Parathyroid Hormone
950.	SELP	Selectin P
951.	TJP1	Tight Junction Protein 1
952.	TNFRSF10A	TNF Receptor Superfamily Member 10a
953.	AGO2	Argonaute RISC Catalytic Component 2
954.	CRHR1	Corticotropin Releasing Hormone Receptor 1
955.	EFNB2	Ephrin B2
956.	IL15	Interleukin 15
957.	GZMA	Granzyme A
958.	IL17F	Interleukin 17F
959.	IL22	Interleukin 22
960.	ITGA9	Integrin Subunit Alpha 9
961.	LYVE1	Lymphatic Vessel Endothelial Hyaluronan Receptor 1
962.	SPI1	Spi-1 Proto-Oncogene
963.	PPIG	Peptidylprolyl Isomerase G
964.	PROX1	Prospero Homeobox 1
965.	CELSR1	Cadherin EGF LAG Seven-Pass G-Type Receptor 1
966.	ERAP2	Endoplasmic Reticulum Aminopeptidase 2
967.	FOXF1	Forkhead Box F1
968.	GNAZ	G Protein Subunit Alpha Z
969.	SOX18	SRY-Box Transcription Factor 18
970.	CCBE1	Collagen And Calcium Binding EGF Domains 1
971.	CXCL9	C-X-C Motif Chemokine Ligand 9
972.	HLA-DMA	Major Histocompatibility Complex, Class II, DM Alpha
973.	PIEZO1	Piezo Type Mechanosensitive Ion Channel Component 1 (Er Blood Group)
974.	SUGCT	Succinyl-CoA:Glutarate-CoA Transferase
975.	SHOX	SHOX Homeobox

976.	VIPAS39	VPS33B Interacting Protein, Apical-Basolateral Polarity Regulator, Spe-39 Homolog
977.	C16orf95	Chromosome 16 Open Reading Frame 95
978.	DISC1	DISC1 Scaffold Protein
979.	CTSL	Cathepsin L
980.	CSF1	Colony Stimulating Factor 1
981.	LGMN	Legumain
982.	RUNX2	RUNX Family Transcription Factor 2
983.	SLC2A1	Solute Carrier Family 2 Member 1
984.	CD28	CD28 Molecule
985.	DCTN1	Dynactin Subunit 1
986.	RPA1	Replication Protein A1
987.	TMPRSS2	Transmembrane Serine Protease 2
988.	WRN	WRN RecQ Like Helicase
989.	KDM5C	Lysine Demethylase 5C
990.	HP	Haptoglobin
991.	TPH1	Tryptophan Hydroxylase 1
992.	GJA8	Gap Junction Protein Alpha 8
993.	HCRTR2	Hypocretin Receptor 2
994.	RPA2	Replication Protein A2
995.	POLI	DNA Polymerase Iota
996.	GJA3	Gap Junction Protein Alpha 3
997.	DYNLT1	Dynein Light Chain Tctex-Type 1
998.	AMBN	Ameloblastin
999.	TPCN2	Two Pore Segment Channel 2
1000.	NRXN1	Neurexin 1
1001.	TACR3	Tachykinin Receptor 3
1002.	UBE3A	Ubiquitin Protein Ligase E3A
1003.	ESRRB	Estrogen Related Receptor Beta
1004.	C1R	Complement C1r
1005.	C1S	Complement C1s
1006.	ATP2B3	ATPase Plasma Membrane Ca2+ Transporting 3
1007.	CANX	Calnexin
1008.	TRPM8	Transient Receptor Potential Cation Channel Subfamily M Member 8
1009.	LRPAP1	LDL Receptor Related Protein Associated Protein 1
1010.	PRDM1	PR/SET Domain 1
1011.	SNRPN	Small Nuclear Ribonucleoprotein Polypeptide N
1012.	TNFRSF4	TNF Receptor Superfamily Member 4
1013.	BAMBI	BMP And Activin Membrane Bound Inhibitor
1014.	LBP	Lipopolysaccharide Binding Protein
1015.	SH3GL1	SH3 Domain Containing GRB2 Like 1, Endophilin A2
1016.	ACKR1	Atypical Chemokine Receptor 1 (Duffy Blood Group)

1017.	CDH15	Cadherin 15
1018.	NDUFS4	NADH:Ubiquinone Oxidoreductase Subunit S4
1019.	RAB3A	RAB3A, Member RAS Oncogene Family
1020.	TNFRSF14	TNF Receptor Superfamily Member 14
1021.	C7	Complement C7
1022.	LHX4	LIM Homeobox 4
1023.	MRC2	Mannose Receptor C Type 2
1024.	ACBD5	Acyl-CoA Binding Domain Containing 5
1025.	CFHR3	Complement Factor H Related 3
1026.	CXCL13	C-X-C Motif Chemokine Ligand 13
1027.	BTLA	B And T Lymphocyte Associated
1028.	C6	Complement C6
1029.	CD160	CD160 Molecule
1030.	CEP63	Centrosomal Protein 63
1031.	CCL24	C-C Motif Chemokine Ligand 24
1032.	CEP135	Centrosomal Protein 135
1033.	CEP152	Centrosomal Protein 152
1034.	LY75	Lymphocyte Antigen 75
1035.	MMP25	Matrix Metallopeptidase 25
1036.	DR1	Down-Regulator Of Transcription 1
1037.	ERC2	ELKS/RAB6-Interacting/CAST Family Member 2
1038.	PHACTR1	Phosphatase And Actin Regulator 1
1039.	SLC24A2	Solute Carrier Family 24 Member 2
1040.	SLC4A3	Solute Carrier Family 4 Member 3
1041.	CREB3	CAMP Responsive Element Binding Protein 3
1042.	GIPC3	GIPC PDZ Domain Containing Family Member 3
1043.	NPRL3	NPR3 Like, GATOR1 Complex Subunit
1044.	POC1A	POC1 Centriolar Protein A
1045.	SEC16B	SEC16 Homolog B, Endoplasmic Reticulum Export Factor
1046.	TNFSF9	TNF Superfamily Member 9
1047.	CACNG8	Calcium Voltage-Gated Channel Auxiliary Subunit Gamma 8
1048.	ITGA10	Integrin Subunit Alpha 10
1049.	ARL6IP5	ADP Ribosylation Factor Like GTPase 6 Interacting Protein 5
1050.	CEP128	Centrosomal Protein 128
1051.	CEP350	Centrosomal Protein 350
1052.	NDUFAF7	NADH:Ubiquinone Oxidoreductase Complex Assembly Factor 7
1053.	NIPAL1	NIPA Like Domain Containing 1
1054.	TTLL6	Tubulin Tyrosine Ligase Like 6
1055.	GPKOW	G-Patch Domain And KOW Motifs
1056.	KCP	Kielin Cysteine Rich BMP Regulator
1057.	KIAA0753	KIAA0753

1058.	NFATC2IP	Nuclear Factor Of Activated T Cells 2 Interacting Protein
1059.	NIPAL3	NIPA Like Domain Containing 3
1060.	ONECUT2	One Cut Homeobox 2
1061.	PHOSPHO2	Phosphatase, Orphan 2
1062.	SHISA6	Shisa Family Member 6
1063.	C18orf32	Chromosome 18 Open Reading Frame 32
1064.	FAM83G	Family With Sequence Similarity 83 Member G
1065.	LRIT2	Leucine Rich Repeat, Ig-Like And Transmembrane Domains 2
1066.	MAPK1IP1L	Mitogen-Activated Protein Kinase 1 Interacting Protein 1 Like
1067.	NIPAL2	NIPA Like Domain Containing 2
1068.	TTLL7	Tubulin Tyrosine Ligase Like 7
1069.	ZNF385A	Zinc Finger Protein 385A
1070.	BARHL2	BarH Like Homeobox 2
1071.	CCDC126	Coiled-Coil Domain Containing 126
1072.	CCDC66	Coiled-Coil Domain Containing 66
1073.	CDR1	Cerebellar Degeneration Related 1
1074.	KLHL23	Kelch Like Family Member 23
1075.	BHLHE23	Basic Helix-Loop-Helix Family Member E23
1076.	TMEM215	Transmembrane Protein 215
1077.	UBAP1L	Ubiquitin Associated Protein 1 Like
1078.	CCDC175	Coiled-Coil Domain Containing 175
1079.	TTLL8	Tubulin Tyrosine Ligase Like 8
1080.	LY75-CD302	LY75-CD302 Readthrough
1081.	SHANK2	SH3 And Multiple Ankyrin Repeat Domains 2
1082.	FOXE3	Forkhead Box E3
1083.	DLD	Dihydrolipoamide Dehydrogenase
1084.	CFTR	CF Transmembrane Conductance Regulator
1085.	LPL	Lipoprotein Lipase
1086.	CACNA1B	Calcium Voltage-Gated Channel Subunit Alpha1 B
1087.	IMPDH2	Inosine Monophosphate Dehydrogenase 2
1088.	KLF4	KLF Transcription Factor 4
1089.	ADM	Adrenomedullin
1090.	GK	Glycerol Kinase
1091.	ADCY7	Adenylate Cyclase 7
1092.	DEPDC5	DEP Domain Containing 5, GATOR1 Subcomplex Subunit
1093.	LNX1	Ligand Of Numb-Protein X 1
1094.	RECK	Reversion Inducing Cysteine Rich Protein With Kazal Motifs
1095.	CCT2	Chaperonin Containing TCP1 Subunit 2
1096.	CEP41	Centrosomal Protein 41
1097.	ANOS1	Anosmin 1
1098.	LIN7A	Lin-7 Homolog A, Crumbs Cell Polarity Complex Component

1099.	MTDH	Metadherin
1100.	POU4F1	POU Class 4 Homeobox 1
1101.	SLC6A20	Solute Carrier Family 6 Member 20
1102.	SMN1	Survival Of Motor Neuron 1, Telomeric
1103.	SMN2	Survival Of Motor Neuron 2, Centromeric
1104.	SQOR	Sulfide Quinone Oxidoreductase
1105.	TMC1	Transmembrane Channel Like 1
1106.	CSPP1	Centrosome And Spindle Pole Associated Protein 1
1107.	PNOC	Prepronociceptin
1108.	RBBP6	RB Binding Protein 6, Ubiquitin Ligase
1109.	RDH13	Retinol Dehydrogenase 13
1110.	FARP1	FERM, ARH/RhoGEF And Pleckstrin Domain Protein 1
1111.	LRRTM1	Leucine Rich Repeat Transmembrane Neuronal 1
1112.	UGGT2	UDP-Glucose Glycoprotein Glucosyltransferase 2
1113.	LHX2	LIM Homeobox 2
1114.	PATJ	PATJ Crumbs Cell Polarity Complex Component
1115.	TMEM107	Transmembrane Protein 107
1116.	CHML	CHM Like Rab Escort Protein
1117.	PNPLA4	Patatin Like Phospholipase Domain Containing 4
1118.	HPCAL4	Hippocalcin Like 4
1119.	KCNG3	Potassium Voltage-Gated Channel Modifier Subfamily G Member 3
1120.	PUDP	Pseudouridine 5'-Phosphatase
1121.	CRB3	Crumbs Cell Polarity Complex Component 3
1122.	PPP1R12C	Protein Phosphatase 1 Regulatory Subunit 12C
1123.	FAM91A1	Family With Sequence Similarity 91 Member A1
1124.	TMEM161A	Transmembrane Protein 161A
1125.	TMEM184C	Transmembrane Protein 184C
1126.	ARMC6	Armadillo Repeat Containing 6
1127.	TMEM86B	Transmembrane Protein 86B
1128.	PYDC1	Pyrin Domain Containing 1
1129.	PRRT4	Proline Rich Transmembrane Protein 4
1130.	UTP14C	UTP14C Small Subunit Processome Component
1131.	C17orf75	Chromosome 17 Open Reading Frame 75
1132.	TMEM86A	Transmembrane Protein 86A
1133.	ERBB4	Erb-B2 Receptor Tyrosine Kinase 4
1134.	JAK2	Janus Kinase 2
1135.	CD19	CD19 Molecule
1136.	CHEK1	Checkpoint Kinase 1
1137.	ALDH2	Aldehyde Dehydrogenase 2 Family Member
1138.	RAD50	RAD50 Double Strand Break Repair Protein
1139.	VCP	Valosin Containing Protein

1140.	TOP1	DNA Topoisomerase I
1141.	GABRA5	Gamma-Aminobutyric Acid Type A Receptor Subunit Alpha5
1142.	POLG	DNA Polymerase Gamma, Catalytic Subunit
1143.	CACNB4	Calcium Voltage-Gated Channel Auxiliary Subunit Beta 4
1144.	DOCK8	Dedicator Of Cytokinesis 8
1145.	FUS	FUS RNA Binding Protein
1146.	PDYN	Prodynorphin
1147.	POLD1	DNA Polymerase Delta 1, Catalytic Subunit
1148.	PRSS8	Serine Protease 8
1149.	APTX	Aprataxin
1150.	NPY	Neuropeptide Y
1151.	EXO1	Exonuclease 1
1152.	HTR2B	5-Hydroxytryptamine Receptor 2B
1153.	LTA	Lymphotoxin Alpha
1154.	NTHL1	Nth Like DNA Glycosylase 1
1155.	TRIM21	Tripartite Motif Containing 21
1156.	AFF2	ALF Transcription Elongation Factor 2
1157.	FBL	Fibrillarin
1158.	GSTM1	Glutathione S-Transferase Mu 1
1159.	MDC1	Mediator Of DNA Damage Checkpoint 1
1160.	RTEL1	Regulator Of Telomere Elongation Helicase 1
1161.	SPTBN2	Spectrin Beta, Non-Erythrocytic 2
1162.	DNA2	DNA Replication Helicase/Nuclease 2
1163.	RECQL4	RecQ Like Helicase 4
1164.	CLMP	CXADR Like Membrane Protein
1165.	AP3S1	Adaptor Related Protein Complex 3 Subunit Sigma 1
1166.	SLC24A3	Solute Carrier Family 24 Member 3
1167.	SLX4	SLX4 Structure-Specific Endonuclease Subunit
1168.	SUPT5H	SPT5 Homolog, DSIF Elongation Factor Subunit
1169.	GEN1	GEN1 Holliday Junction 5' Flap Endonuclease
1170.	MMS19	MMS19 Homolog, Cytosolic Iron-Sulfur Assembly Component
1171.	NEIL1	Nei Like DNA Glycosylase 1
1172.	SNAPIN	SNAP Associated Protein
1173.	GPRASP2	G Protein-Coupled Receptor Associated Sorting Protein 2
1174.	H2AC20	H2A Clustered Histone 20
1175.	TCEA2	Transcription Elongation Factor A2
1176.	TCEA3	Transcription Elongation Factor A3
1177.	BLOC1S4	Biogenesis Of Lysosomal Organelles Complex 1 Subunit 4
1178.	ENDOV	Endonuclease V
1179.	LRRC70	Leucine Rich Repeat Containing 70
1180.	VEGFA	Vascular Endothelial Growth Factor A

1181.	HTR1F	5-Hydroxytryptamine Receptor 1F
1182.	NEK2	NIMA Related Kinase 2
1183.	EPCAM	Epithelial Cell Adhesion Molecule
1184.	ITGB6	Integrin Subunit Beta 6
1185.	PSAP	Prosaposin
1186.	AHR	Aryl Hydrocarbon Receptor
1187.	KRT5	Keratin 5
1188.	LSS	Lanosterol Synthase
1189.	PLCD1	Phospholipase C Delta 1
1190.	AHSG	Alpha 2-HS Glycoprotein
1191.	ANTXR1	ANTXR Cell Adhesion Molecule 1
1192.	SLC6A19	Solute Carrier Family 6 Member 19
1193.	TARS1	Threonyl-TRNA Synthetase 1
1194.	CARS1	Cysteinyl-TRNA Synthetase 1
1195.	GTF2E2	General Transcription Factor IIE Subunit 2
1196.	SLC39A4	Solute Carrier Family 39 Member 4
1197.	ELOVL1	ELOVL Fatty Acid Elongase 1
1198.	RNF113A	Ring Finger Protein 113A
1199.	CLTRN	Collectrin, Amino Acid Transport Regulator
1200.	PERCC1	Proline And Glutamate Rich With Coiled Coil 1
1201.	JUN	Jun Proto-Oncogene, AP-1 Transcription Factor Subunit
1202.	TCF4	Transcription Factor 4
1203.	GRID2	Glutamate Ionotropic Receptor Delta Type Subunit 2
1204.	DPP10	Dipeptidyl Peptidase Like 10
1205.	GDPD1	Glycerophosphodiester Phosphodiesterase Domain Containing 1
1206.	YPEL2	Yippee Like 2
1207.	KDR	Kinase Insert Domain Receptor
1208.	TLR3	Toll Like Receptor 3
1209.	GSK3B	Glycogen Synthase Kinase 3 Beta
1210.	HDAC9	Histone Deacetylase 9
1211.	MSH6	MutS Homolog 6
1212.	OPRM1	Opioid Receptor Mu 1
1213.	FBN1	Fibrillin 1
1214.	GZMB	Granzyme B
1215.	ITGAM	Integrin Subunit Alpha M
1216.	NR1H4	Nuclear Receptor Subfamily 1 Group H Member 4
1217.	SLC9A3	Solute Carrier Family 9 Member A3
1218.	ABCB11	ATP Binding Cassette Subfamily B Member 11
1219.	ABCB4	ATP Binding Cassette Subfamily B Member 4
1220.	HTR7	5-Hydroxytryptamine Receptor 7
1221.	ALDH3A1	Aldehyde Dehydrogenase 3 Family Member A1

1222.	CA8	Carbonic Anhydrase 8
1223.	CD274	CD274 Molecule
1224.	GRM2	Glutamate Metabotropic Receptor 2
1225.	NEFL	Neurofilament Light Chain
1226.	TIMP1	TIMP Metallopeptidase Inhibitor 1
1227.	ALDH4A1	Aldehyde Dehydrogenase 4 Family Member A1
1228.	ARRB1	Arrestin Beta 1
1229.	ATXN2	Ataxin 2
1230.	CD86	CD86 Molecule
1231.	CXCL10	C-X-C Motif Chemokine Ligand 10
1232.	FA2H	Fatty Acid 2-Hydroxylase
1233.	HTT	Huntingtin
1234.	CD80	CD80 Molecule
1235.	FGF19	Fibroblast Growth Factor 19
1236.	GABRG3	Gamma-Aminobutyric Acid Type A Receptor Subunit Gamma3
1237.	IL12RB2	Interleukin 12 Receptor Subunit Beta 2
1238.	ADCYAP1	Adenylate Cyclase Activating Polypeptide 1
1239.	CALM3	Calmodulin 3
1240.	CAV2	Caveolin 2
1241.	CYP7A1	Cytochrome P450 Family 7 Subfamily A Member 1
1242.	HAVCR1	Hepatitis A Virus Cellular Receptor 1
1243.	SLC10A2	Solute Carrier Family 10 Member 2
1244.	XRCC3	X-Ray Repair Cross Complementing 3
1245.	AGK	Acylglycerol Kinase
1246.	ALDH9A1	Aldehyde Dehydrogenase 9 Family Member A1
1247.	DNAJC19	DnaJ Heat Shock Protein Family (Hsp40) Member C19
1248.	DNMBP	Dynamin Binding Protein
1249.	HTR5A	5-Hydroxytryptamine Receptor 5A
1250.	C9orf72	C9orf72-SMCR8 Complex Subunit
1251.	TOMM40	Translocase Of Outer Mitochondrial Membrane 40
1252.	AIF1	Allograft Inflammatory Factor 1
1253.	ALDH1L1	Aldehyde Dehydrogenase 1 Family Member L1
1254.	CALML3	Calmodulin Like 3
1255.	DDHD2	DDHD Domain Containing 2
1256.	KIR3DL1	Killer Cell Immunoglobulin Like Receptor, Three Ig Domains And Long Cytoplasmic Tail 1
1257.	NUP210	Nucleoporin 210
1258.	OGA	O-GlcNAcase
1259.	PLEKHG4	Pleckstrin Homology And RhoGEF Domain Containing G4
1260.	RGS4	Regulator Of G Protein Signaling 4
1261.	RHCE	Rh Blood Group CcEe Antigens
1262.	ALDH1L2	Aldehyde Dehydrogenase 1 Family Member L2

1263.	ALDH3B1	Aldehyde Dehydrogenase 3 Family Member B1
1264.	DCAF17	DDB1 And CUL4 Associated Factor 17
1265.	HACL1	2-Hydroxyacyl-CoA Lyase 1
1266.	SLC35B2	Solute Carrier Family 35 Member B2
1267.	TIMM17A	Translocase Of Inner Mitochondrial Membrane 17A
1268.	TIMM50	Translocase Of Inner Mitochondrial Membrane 50
1269.	TTC7A	Tetratricopeptide Repeat Domain 7A
1270.	CALML5	Calmodulin Like 5
1271.	NAXD	NAD(P)HX Dehydratase
1272.	TIMM13	Translocase Of Inner Mitochondrial Membrane 13
1273.	TIMM9	Translocase Of Inner Mitochondrial Membrane 9
1274.	CALML4	Calmodulin Like 4
1275.	DRP2	Dystrophin Related Protein 2
1276.	GPN1	GPN-Loop GTPase 1
1277.	SERAC1	Serine Active Site Containing 1
1278.	TIMM10	Translocase Of Inner Mitochondrial Membrane 10
1279.	CHCHD4	Coiled-Coil-Helix-Coiled-Coil-Helix Domain Containing 4
1280.	DERA	Deoxyribose-Phosphate Aldolase
1281.	ROGDI	Rogdi Atypical Leucine Zipper
1282.	SMPX	Small Muscle Protein X-Linked
1283.	TIMM10B	Translocase Of Inner Mitochondrial Membrane 10B
1284.	TYW3	TRNA-YW Synthesizing Protein 3 Homolog
1285.	ALDH16A1	Aldehyde Dehydrogenase 16 Family Member A1
1286.	CFAP43	Cilia And Flagella Associated Protein 43
1287.	CNNM1	Cyclin And CBS Domain Divalent Metal Cation Transport Mediator 1
1288.	DNAJC9	DnaJ Heat Shock Protein Family (Hsp40) Member C9
1289.	FITM2	Fat Storage Inducing Transmembrane Protein 2
1290.	IGLON5	IgLON Family Member 5
1291.	KRI1	KRI1 Homolog
1292.	PPRC1	PPARG Related Coactivator 1
1293.	SLC50A1	Solute Carrier Family 50 Member 1
1294.	TIMM23	Translocase Of Inner Mitochondrial Membrane 23
1295.	CALML6	Calmodulin Like 6
1296.	SCT	Secretin
1297.	NXPH3	Neurexophilin 3
1298.	TAF7L	TATA-Box Binding Protein Associated Factor 7 Like
1299.	DNAJC22	DnaJ Heat Shock Protein Family (Hsp40) Member C22
1300.	MBLAC2	Metallo-Beta-Lactamase Domain Containing 2
1301.	TIMM29	Translocase Of Inner Mitochondrial Membrane 29
1302.	AKT3	AKT Serine/Threonine Kinase 3
1303.	CALR	Calreticulin

1304.	SERPINC1	Serpin Family C Member 1
1305.	CACNA1G	Calcium Voltage-Gated Channel Subunit Alpha1 G
1306.	IGF2	Insulin Like Growth Factor 2
1307.	PTK2B	Protein Tyrosine Kinase 2 Beta
1308.	REN	Renin
1309.	RHEB	Ras Homolog, MTORC1 Binding
1310.	F5	Coagulation Factor V
1311.	IL1R1	Interleukin 1 Receptor Type 1
1312.	PON1	Paraoxonase 1
1313.	XDH	Xanthine Dehydrogenase
1314.	KCNJ3	Potassium Inwardly Rectifying Channel Subfamily J Member 3
1315.	PLA2G6	Phospholipase A2 Group VI
1316.	VCAM1	Vascular Cell Adhesion Molecule 1
1317.	PANX1	Pannexin 1
1318.	EPHX1	Epoxide Hydrolase 1
1319.	MEF2D	Myocyte Enhancer Factor 2D
1320.	PRDM16	PR/SET Domain 16
1321.	SELE	Selectin E
1322.	HGD	Homogentisate 1,2-Dioxygenase
1323.	NLRC4	NLR Family CARD Domain Containing 4
1324.	P2RX3	Purinergic Receptor P2X 3
1325.	SELL	Selectin L
1326.	SGCE	Sarcoglycan Epsilon
1327.	CDA	Cytidine Deaminase
1328.	GNB2	G Protein Subunit Beta 2
1329.	NAT2	N-Acetyltransferase 2
1330.	SDC4	Syndecan 4
1331.	ABCG8	ATP Binding Cassette Subfamily G Member 8
1332.	AP2B1	Adaptor Related Protein Complex 2 Subunit Beta 1
1333.	GSTZ1	Glutathione S-Transferase Zeta 1
1334.	IL33	Interleukin 33
1335.	NPRL2	NPR2 Like, GATOR1 Complex Subunit
1336.	PIP4K2C	Phosphatidylinositol-5-Phosphate 4-Kinase Type 2 Gamma
1337.	KCNK5	Potassium Two Pore Domain Channel Subfamily K Member 5
1338.	SBF2	SET Binding Factor 2
1339.	ZFP36L2	ZFP36 Ring Finger Protein Like 2
1340.	GNG3	G Protein Subunit Gamma 3
1341.	TSPAN2	Tetraspanin 2
1342.	BCO1	Beta-Carotene Oxygenase 1
1343.	ZFYVE27	Zinc Finger FYVE-Type Containing 27
1344.	IFT70B	Intraflagellar Transport 70B

1345.	NUB1	Negative Regulator Of Ubiquitin Like Proteins 1
1346.	RIC1	RIC1 Homolog, RAB6A GEF Complex Partner 1
1347.	TSSK4	Testis Specific Serine Kinase 4
1348.	APOL3	Apolipoprotein L3
1349.	CMKLR2	Chemerin Chemokine-Like Receptor 2
1350.	LUZP2	Leucine Zipper Protein 2
1351.	MXRA8	Matrix Remodeling Associated 8
1352.	CLASRP	CLK4 Associating Serine/Arginine Rich Protein
1353.	PITPNM2	Phosphatidylinositol Transfer Protein Membrane Associated 2
1354.	GNG10	G Protein Subunit Gamma 10
1355.	ZNF337	Zinc Finger Protein 337
1356.	ZDBF2	Zinc Finger DBF-Type Containing 2
1357.	GNG14	G Protein Subunit Gamma 14
1358.	PCSK9	Proprotein Convertase Subtilisin/Kexin Type 9
1359.	CD46	CD46 Molecule
1360.	GCH1	GTP Cyclohydrolase 1
1361.	BCL11A	BCL11 Transcription Factor A
1362.	SMS	Spermine Synthase
1363.	SEC24D	SEC24 Homolog D, COPII Coat Complex Component
1364.	ATP10A	ATPase Phospholipid Transporting 10A (Putative)
1365.	CREB3L1	CAMP Responsive Element Binding Protein 3 Like 1
1366.	CRTAP	Cartilage Associated Protein
1367.	FKBP10	FKBP Prolyl Isomerase 10
1368.	P3H1	Prolyl 3-Hydroxylase 1
1369.	TMEM38B	Transmembrane Protein 38B
1370.	ABCA5	ATP Binding Cassette Subfamily A Member 5
1371.	CREB3L2	CAMP Responsive Element Binding Protein 3 Like 2
1372.	APOBEC1	Apolipoprotein B MRNA Editing Enzyme Catalytic Subunit 1
1373.	CCDC134	Coiled-Coil Domain Containing 134
1374.	MIA3	MIA SH3 Domain ER Export Factor 3
1375.	PAOX	Polyamine Oxidase
1376.	PHYKPL	5-Phosphohydroxy-L-Lysine Phospho-Lyase
1377.	SMOX	Spermine Oxidase
1378.	IFITM5	Interferon Induced Transmembrane Protein 5
1379.	IMMP1L	Inner Mitochondrial Membrane Peptidase Subunit 1
1380.	SH2D3C	SH2 Domain Containing 3C
1381.	TMEM38A	Transmembrane Protein 38A
1382.	SH2D3A	SH2 Domain Containing 3A
1383.	DOHH	Deoxyhypusine Hydroxylase
1384.	DNAJC24	DnaJ Heat Shock Protein Family (Hsp40) Member C24
1385.	PRSS57	Serine Protease 57

1386.	GAD2	Glutamate Decarboxylase 2
1387.	STXBP1	Syntaxin Binding Protein 1
1388.	CHI3L1	Chitinase 3 Like 1
1389.	RTN4R	Reticulon 4 Receptor
1390.	RELN	Reelin
1391.	FHIT	Fragile Histidine Triad Diadenosine Triphosphatase
1392.	DLGAP2	DLG Associated Protein 2
1393.	ZNF804A	Zinc Finger Protein 804A
1394.	CHEK2	Checkpoint Kinase 2
1395.	ATR	ATR Serine/Threonine Kinase
1396.	AR	Androgen Receptor
1397.	FANCA	FA Complementation Group A
1398.	G6PD	Glucose-6-Phosphate Dehydrogenase
1399.	CACNA1H	Calcium Voltage-Gated Channel Subunit Alpha1 H
1400.	CASP3	Caspase 3
1401.	MLH1	MutL Homolog 1
1402.	PTGS2	Prostaglandin-Endoperoxide Synthase 2
1403.	RRM1	Ribonucleotide Reductase Catalytic Subunit M1
1404.	MGMT	O-6-Methylguanine-DNA Methyltransferase
1405.	MSH2	MutS Homolog 2
1406.	CHRM2	Cholinergic Receptor Muscarinic 2
1407.	FAAH	Fatty Acid Amide Hydrolase
1408.	FANCC	FA Complementation Group C
1409.	GRN	Granulin Precursor
1410.	KCNQ2	Potassium Voltage-Gated Channel Subfamily Q Member 2
1411.	LIG4	DNA Ligase 4
1412.	SCN10A	Sodium Voltage-Gated Channel Alpha Subunit 10
1413.	ADIPOQ	Adiponectin, C1Q And Collagen Domain Containing
1414.	CTSS	Cathepsin S
1415.	FANCD2	FA Complementation Group D2
1416.	FANCL	FA Complementation Group L
1417.	IGFBP3	Insulin Like Growth Factor Binding Protein 3
1418.	PNKP	Polynucleotide Kinase 3'-Phosphatase
1419.	RAD54L	RAD54 Like
1420.	RPSA	Ribosomal Protein SA
1421.	UBE2N	Ubiquitin Conjugating Enzyme E2 N
1422.	UNG	Uracil DNA Glycosylase
1423.	ADCYAP1R1	ADCYAP Receptor Type I
1424.	GRIK1	Glutamate Ionotropic Receptor Kainate Type Subunit 1
1425.	HERC2	HECT And RLD Domain Containing E3 Ubiquitin Protein Ligase 2
1426.	IL1RAP	Interleukin 1 Receptor Accessory Protein

1427.	SCN1B	Sodium Voltage-Gated Channel Beta Subunit 1
1428.	TACR1	Tachykinin Receptor 1
1429.	CRYAA	Crystallin Alpha A
1430.	DDX1	DEAD-Box Helicase 1
1431.	H2AX	H2A.X Variant Histone
1432.	KPNB1	Karyopherin Subunit Beta 1
1433.	LGALS3	Galectin 3
1434.	MUTYH	MutY DNA Glycosylase
1435.	PALB2	Partner And Localizer Of BRCA2
1436.	RBBP8	RB Binding Protein 8, Endonuclease
1437.	REV3L	REV3 Like, DNA Directed Polymerase Zeta Catalytic Subunit
1438.	SFTPA1	Surfactant Protein A1
1439.	TDP1	Tyrosyl-DNA Phosphodiesterase 1
1440.	USP1	Ubiquitin Specific Peptidase 1
1441.	VIP	Vasoactive Intestinal Peptide
1442.	VIPR2	Vasoactive Intestinal Peptide Receptor 2
1443.	AICDA	Activation Induced Cytidine Deaminase
1444.	FANCG	FA Complementation Group G
1445.	FANCI	FA Complementation Group I
1446.	GADD45A	Growth Arrest And DNA Damage Inducible Alpha
1447.	MSH3	MutS Homolog 3
1448.	POLK	DNA Polymerase Kappa
1449.	POLL	DNA Polymerase Lambda
1450.	RAD17	RAD17 Checkpoint Clamp Loader Component
1451.	ATRIP	ATR Interacting Protein
1452.	CDT1	Chromatin Licensing And DNA Replication Factor 1
1453.	CHST3	Carbohydrate Sulfotransferase 3
1454.	FANCM	FA Complementation Group M
1455.	HRH3	Histamine Receptor H3
1456.	KPNA1	Karyopherin Subunit Alpha 1
1457.	MAD2L2	Mitotic Arrest Deficient 2 Like 2
1458.	RNF168	Ring Finger Protein 168
1459.	RNF8	Ring Finger Protein 8
1460.	SATB1	SATB Homeobox 1
1461.	SETX	Senataxin
1462.	TGM3	Transglutaminase 3
1463.	CLSPN	Claspin
1464.	FMOD	Fibromodulin
1465.	HLTF	Helicase Like Transcription Factor
1466.	KLRK1	Killer Cell Lectin Like Receptor K1
1467.	RAD18	RAD18 E3 Ubiquitin Protein Ligase

1468.	RECQL	RecQ Like Helicase
1469.	REV1	REV1 DNA Directed Polymerase
1470.	SH2B1	SH2B Adaptor Protein 1
1471.	TOPBP1	DNA Topoisomerase II Binding Protein 1
1472.	XRCC2	X-Ray Repair Cross Complementing 2
1473.	GOLGA2	Golgin A2
1474.	MICB	MHC Class I Polypeptide-Related Sequence B
1475.	MUS81	MUS81 Structure-Specific Endonuclease Subunit
1476.	POLQ	DNA Polymerase Theta
1477.	RAD9A	RAD9 Checkpoint Clamp Component A
1478.	CRYBB2	Crystallin Beta B2
1479.	HUS1	HUS1 Checkpoint Clamp Component
1480.	IL1RAPL2	Interleukin 1 Receptor Accessory Protein Like 2
1481.	IL37	Interleukin 37
1482.	MPG	N-Methylpurine DNA Glycosylase
1483.	OTUD7A	OTU Deubiquitinase 7A
1484.	POLD2	DNA Polymerase Delta 2, Accessory Subunit
1485.	POLD3	DNA Polymerase Delta 3, Accessory Subunit
1486.	POLM	DNA Polymerase Mu
1487.	RMI1	RecQ Mediated Genome Instability 1
1488.	RPA3	Replication Protein A3
1489.	SMUG1	Single-Strand-Selective Monofunctional Uracil-DNA Glycosylase 1
1490.	SP4	Sp4 Transcription Factor
1491.	STN1	STN1 Subunit Of CST Complex
1492.	TDG	Thymine DNA Glycosylase
1493.	UBE2V2	Ubiquitin Conjugating Enzyme E2 V2
1494.	VPS33A	VPS33A Core Subunit Of CORVET And HOPS Complexes
1495.	WDR48	WD Repeat Domain 48
1496.	CHST5	Carbohydrate Sulfotransferase 5
1497.	SFTPA2	Surfactant Protein A2
1498.	SIK3	SIK Family Kinase 3
1499.	SULT1B1	Sulfotransferase Family 1B Member 1
1500.	UFSP2	UFM1 Specific Peptidase 2
1501.	EME1	Essential Meiotic Structure-Specific Endonuclease 1
1502.	EMSY	EMSY Transcriptional Repressor, BRCA2 Interacting
1503.	HOXC4	Homeobox C4
1504.	IL1F10	Interleukin 1 Family Member 10
1505.	NEIL2	Nei Like DNA Glycosylase 2
1506.	RNF111	Ring Finger Protein 111
1507.	CHST1	Carbohydrate Sulfotransferase 1
1508.	NAV1	Neuron Navigator 1

1509.	SHPRH	SNF2 Histone Linker PHD RING Helicase
1510.	TENT4A	Terminal Nucleotidyltransferase 4A
1511.	ATAD5	ATPase Family AAA Domain Containing 5
1512.	DCLRE1A	DNA Cross-Link Repair 1A
1513.	FAAP24	FA Core Complex Associated Protein 24
1514.	NDUFAF6	NADH:Ubiquinone Oxidoreductase Complex Assembly Factor 6
1515.	TUBGCP5	Tubulin Gamma Complex Component 5
1516.	B3GNT7	UDP-GlcNAc:BetaGal Beta-1,3-N-Acetylglucosaminyltransferase 7
1517.	FAAP100	FA Core Complex Associated Protein 100
1518.	SPRTN	SprT-Like N-Terminal Domain
1519.	SULT1C3	Sulfotransferase Family 1C Member 3
1520.	SULT1C4	Sulfotransferase Family 1C Member 4
1521.	POLN	DNA Polymerase Nu
1522.	ACTMAP	Actin Maturation Protease
1523.	H2AC18	H2A Clustered Histone 18
1524.	SLX1A	SLX1 Homolog A, Structure-Specific Endonuclease Subunit
1525.	TMPRSS11F	Transmembrane Serine Protease 11F
1526.	MKRN2OS	MKRN2 Opposite Strand
1527.	GRIA2	Glutamate Ionotropic Receptor AMPA Type Subunit 2
1528.	CDH1	Cadherin 1
1529.	MCHR1	Melanin Concentrating Hormone Receptor 1
1530.	IGHE	Immunoglobulin Heavy Constant Epsilon
1531.	C16orf92	Chromosome 16 Open Reading Frame 92
1532.	TREX1	Three Prime Repair Exonuclease 1
1533.	NOTCH2NLC	Notch 2 N-Terminal Like C
1534.	TGFB2	Transforming Growth Factor Beta 2
1535.	SLC1A1	Solute Carrier Family 1 Member 1
1536.	LGI1	Leucine Rich Glioma Inactivated 1
1537.	RBX1	Ring-Box 1
1538.	GJB4	Gap Junction Protein Beta 4
1539.	LINGO1	Leucine Rich Repeat And Ig Domain Containing 1
1540.	FKBP4	FKBP Prolyl Isomerase 4
1541.	ADI1	Acireductone Dioxygenase 1
1542.	KCTD17	Potassium Channel Tetramerization Domain Containing 17
1543.	PITX3	Paired Like Homeodomain 3
1544.	SLC17A8	Solute Carrier Family 17 Member 8
1545.	THAP1	THAP Domain Containing 1
1546.	KIAA0319L	KIAA0319 Like
1547.	RAI14	Retinoic Acid Induced 14
1548.	GLB1L3	Galactosidase Beta 1 Like 3
1549.	LDLRAD3	Low Density Lipoprotein Receptor Class A Domain Containing 3

1550.	ZNF558	Zinc Finger Protein 558
1551.	SEL1L3	SEL1L Family Member 3
1552.	XCL2	X-C Motif Chemokine Ligand 2
1553.	GDPD4	Glycerophosphodiester Phosphodiesterase Domain Containing 4
1554.	DYTN	Dystrotelin
1555.	FJX1	Four-Jointed Box Kinase 1
1556.	TRIM61	Tripartite Motif Containing 61
1557.	DNMT3A	DNA Methyltransferase 3 Alpha
1558.	CSNK2A1	Casein Kinase 2 Alpha 1
1559.	CUL3	Cullin 3
1560.	MEF2C	Myocyte Enhancer Factor 2C
1561.	SETD2	SET Domain Containing 2, Histone Lysine Methyltransferase
1562.	SLC3A1	Solute Carrier Family 3 Member 1
1563.	SMC3	Structural Maintenance Of Chromosomes 3
1564.	DPP6	Dipeptidyl Peptidase Like 6
1565.	TOP2B	DNA Topoisomerase II Beta
1566.	CHD8	Chromodomain Helicase DNA Binding Protein 8
1567.	AUTS2	Activator Of Transcription And Developmental Regulator AUTS2
1568.	CDC42BPB	CDC42 Binding Protein Kinase Beta
1569.	CTNND2	Catenin Delta 2
1570.	DLG2	Discs Large MAGUK Scaffold Protein 2
1571.	TMLHE	Trimethyllysine Hydroxylase, Epsilon
1572.	DEAF1	DEAF1 Transcription Factor
1573.	RBFOX1	RNA Binding Fox-1 Homolog 1
1574.	SYN2	Synapsin II
1575.	DAOA	D-Amino Acid Oxidase Activator
1576.	MPZ	Myelin Protein Zero
1577.	NDUFAF2	NADH:Ubiquinone Oxidoreductase Complex Assembly Factor 2
1578.	JAK1	Janus Kinase 1
1579.	HMOX1	Heme Oxygenase 1
1580.	PRKACA	Protein Kinase CAMP-Activated Catalytic Subunit Alpha
1581.	PSEN1	Presenilin 1
1582.	TYK2	Tyrosine Kinase 2
1583.	LDLR	Low Density Lipoprotein Receptor
1584.	B2M	Beta-2-Microglobulin
1585.	CD40	CD40 Molecule
1586.	CXCR2	C-X-C Motif Chemokine Receptor 2
1587.	LEPR	Leptin Receptor
1588.	PCSK1	Proprotein Convertase Subtilisin/Kexin Type 1
1589.	BMP4	Bone Morphogenetic Protein 4
1590.	ENPP1	Ectonucleotide Pyrophosphatase/Phosphodiesterase 1

1591.	GBA1	Glucosylceramidase Beta 1
1592.	LRRK2	Leucine Rich Repeat Kinase 2
1593.	NOS2	Nitric Oxide Synthase 2
1594.	SERPINA1	Serpin Family A Member 1
1595.	SLC2A2	Solute Carrier Family 2 Member 2
1596.	VKORC1	Vitamin K Epoxide Reductase Complex Subunit 1
1597.	HIF1A	Hypoxia Inducible Factor 1 Subunit Alpha
1598.	PRKACB	Protein Kinase CAMP-Activated Catalytic Subunit Beta
1599.	TNFAIP3	TNF Alpha Induced Protein 3
1600.	ABCA3	ATP Binding Cassette Subfamily A Member 3
1601.	ANXA5	Annexin A5
1602.	CALCR	Calcitonin Receptor
1603.	HSPG2	Heparan Sulfate Proteoglycan 2
1604.	KCNQ3	Potassium Voltage-Gated Channel Subfamily Q Member 3
1605.	MYH9	Myosin Heavy Chain 9
1606.	PAH	Phenylalanine Hydroxylase
1607.	PARK7	Parkinsonism Associated Deglycase
1608.	PDCD1	Programmed Cell Death 1
1609.	SLC18A2	Solute Carrier Family 18 Member A2
1610.	SLC5A2	Solute Carrier Family 5 Member 2
1611.	TAP1	Transporter 1, ATP Binding Cassette Subfamily B Member
1612.	TARDBP	TAR DNA Binding Protein
1613.	ATP7A	ATPase Copper Transporting Alpha
1614.	CACNA1E	Calcium Voltage-Gated Channel Subunit Alpha1 E
1615.	CYP2C9	Cytochrome P450 Family 2 Subfamily C Member 9
1616.	ENPEP	Glutamyl Aminopeptidase
1617.	FCGR3A	Fc Gamma Receptor IIIa
1618.	FGF23	Fibroblast Growth Factor 23
1619.	HNF1A	HNF1 Homeobox A
1620.	IDO1	Indoleamine 2,3-Dioxygenase 1
1621.	IL17RA	Interleukin 17 Receptor A
1622.	KCNK9	Potassium Two Pore Domain Channel Subfamily K Member 9
1623.	MC4R	Melanocortin 4 Receptor
1624.	NTF4	Neurotrophin 4
1625.	PINK1	PTEN Induced Kinase 1
1626.	PTPN22	Protein Tyrosine Phosphatase Non-Receptor Type 22
1627.	TGIF1	TGFB Induced Factor Homeobox 1
1628.	VIPR1	Vasoactive Intestinal Peptide Receptor 1
1629.	ACADVL	Acyl-CoA Dehydrogenase Very Long Chain
1630.	ARF1	ADP Ribosylation Factor 1
1631.	BCS1L	BCS1 Homolog, Ubiquinol-Cytochrome C Reductase Complex Chaperone

1632.	CALCRL	Calcitonin Receptor Like Receptor
1633.	CCR3	C-C Motif Chemokine Receptor 3
1634.	CS	Citrate Synthase
1635.	CYP2B6	Cytochrome P450 Family 2 Subfamily B Member 6
1636.	DAG1	Dystroglycan 1
1637.	FUT2	Fucosyltransferase 2 (H Blood Group)
1638.	IRF8	Interferon Regulatory Factor 8
1639.	LNPEP	Leucyl And Cystinyl Aminopeptidase
1640.	PDE4A	Phosphodiesterase 4A
1641.	PRKACG	Protein Kinase CAMP-Activated Catalytic Subunit Gamma
1642.	RIPK2	Receptor Interacting Serine/Threonine Kinase 2
1643.	SLC22A12	Solute Carrier Family 22 Member 12
1644.	SOX9	SRY-Box Transcription Factor 9
1645.	TBX21	T-Box Transcription Factor 21
1646.	TNFSF13B	TNF Superfamily Member 13b
1647.	TXNRD2	Thioredoxin Reductase 2
1648.	CXCR3	C-X-C Motif Chemokine Receptor 3
1649.	P2RX4	Purinergic Receptor P2X 4
1650.	PGF	Placental Growth Factor
1651.	RAB5A	RAB5A, Member RAS Oncogene Family
1652.	SLC22A6	Solute Carrier Family 22 Member 6
1653.	SV2A	Synaptic Vesicle Glycoprotein 2A
1654.	TAP2	Transporter 2, ATP Binding Cassette Subfamily B Member
1655.	AMBP	Alpha-1-Microglobulin/Bikunin Precursor
1656.	ATP6V1B1	ATPase H+ Transporting V1 Subunit B1
1657.	BCKDHB	Branched Chain Keto Acid Dehydrogenase E1 Subunit Beta
1658.	CCR6	C-C Motif Chemokine Receptor 6
1659.	DPEP1	Dipeptidase 1
1660.	FCGR3B	Fc Gamma Receptor IIIb
1661.	IFNA2	Interferon Alpha 2
1662.	IL1RL2	Interleukin 1 Receptor Like 2
1663.	P2RX2	Purinergic Receptor P2X 2
1664.	RORC	RAR Related Orphan Receptor C
1665.	SFTPB	Surfactant Protein B
1666.	SLC22A2	Solute Carrier Family 22 Member 2
1667.	TXK	TXK Tyrosine Kinase
1668.	ZP3	Zona Pellucida Glycoprotein 3
1669.	ACAD8	Acyl-CoA Dehydrogenase Family Member 8
1670.	ASIC2	Acid Sensing Ion Channel Subunit 2
1671.	ATP12A	ATPase H+/K+ Transporting Non-Gastric Alpha2 Subunit
1672.	BCKDHA	Branched Chain Keto Acid Dehydrogenase E1 Subunit Alpha

1673.	CARD14	Caspase Recruitment Domain Family Member 14
1674.	DST	Dystonin
1675.	EEA1	Early Endosome Antigen 1
1676.	EHHADH	Enoyl-CoA Hydratase And 3-Hydroxyacyl CoA Dehydrogenase
1677.	GCG	Glucagon
1678.	IL36RN	Interleukin 36 Receptor Antagonist
1679.	MUC5B	Mucin 5B, Oligomeric Mucus/Gel-Forming
1680.	NOD1	Nucleotide Binding Oligomerization Domain Containing 1
1681.	PHEX	Phosphate Regulating Endopeptidase X-Linked
1682.	PI4K2A	Phosphatidylinositol 4-Kinase Type 2 Alpha
1683.	RAMP1	Receptor Activity Modifying Protein 1
1684.	RAMP2	Receptor Activity Modifying Protein 2
1685.	SNAP23	Synaptosome Associated Protein 23
1686.	UMOD	Uromodulin
1687.	VPS11	VPS11 Core Subunit Of CORVET And HOPS Complexes
1688.	AP3B2	Adaptor Related Protein Complex 3 Subunit Beta 2
1689.	ATP6V0A4	ATPase H+ Transporting V0 Subunit A4
1690.	GPX3	Glutathione Peroxidase 3
1691.	IL11	Interleukin 11
1692.	KCNK4	Potassium Two Pore Domain Channel Subfamily K Member 4
1693.	LAMTOR2	Late Endosomal/Lysosomal Adaptor, MAPK And MTOR Activator 2
1694.	NAGS	N-Acetylglutamate Synthase
1695.	SFTPC	Surfactant Protein C
1696.	SLC22A8	Solute Carrier Family 22 Member 8
1697.	SLC34A3	Solute Carrier Family 34 Member 3
1698.	STX4	Syntaxin 4
1699.	SUMF1	Sulfatase Modifying Factor 1
1700.	UBASH3B	Ubiquitin Associated And SH3 Domain Containing B
1701.	UNC13B	Unc-13 Homolog B
1702.	VAMP7	Vesicle Associated Membrane Protein 7
1703.	VPS33B	VPS33B Late Endosome And Lysosome Associated
1704.	ANKRD26	Ankyrin Repeat Domain Containing 26
1705.	ASIC3	Acid Sensing Ion Channel Subunit 3
1706.	ATP4A	ATPase H+/K+ Transporting Subunit Alpha
1707.	IAPP	Islet Amyloid Polypeptide
1708.	RAMP3	Receptor Activity Modifying Protein 3
1709.	SIM1	SIM BHLH Transcription Factor 1
1710.	SRSF6	Serine And Arginine Rich Splicing Factor 6
1711.	TRPV2	Transient Receptor Potential Cation Channel Subfamily V Member 2
1712.	AGFG1	ArfGAP With FG Repeats 1
1713.	AMN	Amnion Associated Transmembrane Protein

1714.	AP1S1	Adaptor Related Protein Complex 1 Subunit Sigma 1
1715.	CCL17	C-C Motif Chemokine Ligand 17
1716.	CCRL2	C-C Motif Chemokine Receptor Like 2
1717.	IL23A	Interleukin 23 Subunit Alpha
1718.	INPP5B	Inositol Polyphosphate-5-Phosphatase B
1719.	KCNK10	Potassium Two Pore Domain Channel Subfamily K Member 10
1720.	KCNK2	Potassium Two Pore Domain Channel Subfamily K Member 2
1721.	MAVS	Mitochondrial Antiviral Signaling Protein
1722.	OTULIN	OTU Deubiquitinase With Linear Linkage Specificity
1723.	PPCS	Phosphopantothenoylcysteine Synthetase
1724.	PSIP1	PC4 And SRSF1 Interacting Protein 1
1725.	RABGGTA	Rab Geranylgeranyltransferase Subunit Alpha
1726.	RAI1	Retinoic Acid Induced 1
1727.	SLC17A1	Solute Carrier Family 17 Member 1
1728.	SLC36A2	Solute Carrier Family 36 Member 2
1729.	STX7	Syntaxin 7
1730.	VAMP8	Vesicle Associated Membrane Protein 8
1731.	VPS45	Vacuolar Protein Sorting 45 Homolog
1732.	VTI1B	Vesicle Transport Through Interaction With T-SNAREs 1B
1733.	DTNB	Dystrobrevin Beta
1734.	GOLPH3	Golgi Phosphoprotein 3
1735.	HESX1	HESX Homeobox 1
1736.	KIF13A	Kinesin Family Member 13A
1737.	PIEZO2	Piezo Type Mechanosensitive Ion Channel Component 2
1738.	PPP1R14A	Protein Phosphatase 1 Regulatory Inhibitor Subunit 14A
1739.	RAB27B	RAB27B, Member RAS Oncogene Family
1740.	RGS6	Regulator Of G Protein Signaling 6
1741.	SYTL2	Synaptotagmin Like 2
1742.	AP3M1	Adaptor Related Protein Complex 3 Subunit Mu 1
1743.	ARL5B	ADP Ribosylation Factor Like GTPase 5B
1744.	ASIP	Agouti Signaling Protein
1745.	CALCB	Calcitonin Related Polypeptide Beta
1746.	CCL22	C-C Motif Chemokine Ligand 22
1747.	GNLY	Granulysin
1748.	HLA-DQA2	Major Histocompatibility Complex, Class II, DQ Alpha 2
1749.	HSPB3	Heat Shock Protein Family B (Small) Member 3
1750.	HTR1E	5-Hydroxytryptamine Receptor 1E
1751.	LIPN	Lipase Family Member N
1752.	NANOG	Nanog Homeobox
1753.	RGS19	Regulator Of G Protein Signaling 19
1754.	RGS7	Regulator Of G Protein Signaling 7

1755.	SUMO4	Small Ubiquitin Like Modifier 4
1756.	TGOLN2	Trans-Golgi Network Protein 2
1757.	TRIM39	Tripartite Motif Containing 39
1758.	IL26	Interleukin 26
1759.	LACC1	Laccase Domain Containing 1
1760.	LAPTM4A	Lysosomal Protein Transmembrane 4 Alpha
1761.	PAM16	Presequence Translocase Associated Motor 16
1762.	RGS11	Regulator Of G Protein Signaling 11
1763.	RGS12	Regulator Of G Protein Signaling 12
1764.	SFXN2	Sideroflexin 2
1765.	SFXN3	Sideroflexin 3
1766.	SFXN4	Sideroflexin 4
1767.	STX12	Syntaxin 12
1768.	VPS16	VPS16 Core Subunit Of CORVET And HOPS Complexes
1769.	VPS18	VPS18 Core Subunit Of CORVET And HOPS Complexes
1770.	ANKRD27	Ankyrin Repeat Domain 27
1771.	AP3S2	Adaptor Related Protein Complex 3 Subunit Sigma 2
1772.	ASB10	Ankyrin Repeat And SOCS Box Containing 10
1773.	BLOC1S1	Biogenesis Of Lysosomal Organelles Complex 1 Subunit 1
1774.	GIMAP2	GTPase, IMAP Family Member 2
1775.	GIMAP4	GTPase, IMAP Family Member 4
1776.	KIR2DL1	Killer Cell Immunoglobulin Like Receptor, Two Ig Domains And Long Cytoplasmic Tail 1
1777.	MON1A	MON1 Homolog A, Secretory Trafficking Associated
1778.	MTUS1	Microtubule Associated Scaffold Protein 1
1779.	RAB32	RAB32, Member RAS Oncogene Family
1780.	SEMA6C	Semaphorin 6C
1781.	SGSM2	Small G Protein Signaling Modulator 2
1782.	ADAT3	Adenosine Deaminase TRNA Specific 3
1783.	EMP1	Epithelial Membrane Protein 1
1784.	KRT76	Keratin 76
1785.	MRAP	Melanocortin 2 Receptor Accessory Protein
1786.	MTUS2	Microtubule Associated Scaffold Protein 2
1787.	RBSN	Rabenosyn, RAB Effector
1788.	XXYLT1	Xyloside Xylosyltransferase 1
1789.	ADM2	Adrenomedullin 2
1790.	GNG11	G Protein Subunit Gamma 11
1791.	KXD1	KxDL Motif Containing 1
1792.	ZNF821	Zinc Finger Protein 821
1793.	ATAD3C	ATPase Family AAA Domain Containing 3C
1794.	CCDC180	Coiled-Coil Domain Containing 180
1795.	CCZ1	CCZ1 Homolog, Vacuolar Protein Trafficking And Biogenesis Associated

1796.	DBNDD1	Dysbindin Domain Containing 1
1797.	EEF1AKMT1	EEF1A Lysine Methyltransferase 1
1798.	SLC35D3	Solute Carrier Family 35 Member D3
1799.	CCDC148	Coiled-Coil Domain Containing 148
1800.	WDR17	WD Repeat Domain 17
1801.	ANKDD1B	Ankyrin Repeat And Death Domain Containing 1B
1802.	MRPL57	Mitochondrial Ribosomal Protein L57
1803.	C1orf141	Chromosome 1 Open Reading Frame 141
1804.	CCZ1B	CCZ1 Homolog B, Vacuolar Protein Trafficking And Biogenesis Associated
1805.	CABCOCO1	Ciliary Associated Calcium Binding Coiled-Coil 1
1806.	IGHD	Immunoglobulin Heavy Constant Delta
1807.	IQCF3	IQ Motif Containing F3
1808.	TRIM39-RPP21	TRIM39-RPP21 Readthrough
1809.	SMO	Smoothened, Frizzled Class Receptor
1810.	OXTR	Oxytocin Receptor
1811.	POLE	DNA Polymerase Epsilon, Catalytic Subunit
1812.	PPP2R1A	Protein Phosphatase 2 Scaffold Subunit Aalpha
1813.	GPR161	G Protein-Coupled Receptor 161
1814.	TBCD	Tubulin Folding Cofactor D
1815.	DSG1	Desmoglein 1
1816.	SAA4	Serum Amyloid A4, Constitutive
1817.	JUP	Junction Plakoglobin
1818.	OPA1	OPA1 Mitochondrial Dynamin Like GTPase
1819.	NPC1	NPC Intracellular Cholesterol Transporter 1
1820.	SIRT1	Sirtuin 1
1821.	DLG4	Discs Large MAGUK Scaffold Protein 4
1822.	SMPD1	Sphingomyelin Phosphodiesterase 1
1823.	COL4A3	Collagen Type IV Alpha 3 Chain
1824.	GRIN2C	Glutamate Ionotropic Receptor NMDA Type Subunit 2C
1825.	CD209	CD209 Molecule
1826.	CHIT1	Chitinase 1
1827.	GJA5	Gap Junction Protein Alpha 5
1828.	POLR2A	RNA Polymerase II Subunit A
1829.	POLR3A	RNA Polymerase III Subunit A
1830.	SLC1A4	Solute Carrier Family 1 Member 4
1831.	NEK1	NIMA Related Kinase 1
1832.	NPC2	NPC Intracellular Cholesterol Transporter 2
1833.	COPS5	COP9 Signalosome Subunit 5
1834.	CIMAP3	Ciliary Microtubule Associated Protein 3
1835.	ERBB3	Erb-B2 Receptor Tyrosine Kinase 3
1836.	DYRK1A	Dual Specificity Tyrosine Phosphorylation Regulated Kinase 1A

1837.	CHN1	Chimerin 1
1838.	NR4A2	Nuclear Receptor Subfamily 4 Group A Member 2
1839.	SATB2	SATB Homeobox 2
1840.	DAO	D-Amino Acid Oxidase
1841.	KMT2D	Lysine Methyltransferase 2D
1842.	MAP1B	Microtubule Associated Protein 1B
1843.	ARX	Aristaless Related Homeobox
1844.	BCL11B	BCL11 Transcription Factor B
1845.	RPL10	Ribosomal Protein L10
1846.	BIRC6	Baculoviral IAP Repeat Containing 6
1847.	PQBP1	Polyglutamine Binding Protein 1
1848.	CSMD1	CUB And Sushi Multiple Domains 1
1849.	KATNAL2	Katanin Catalytic Subunit A1 Like 2
1850.	PTCHD1	Patched Domain Containing 1
1851.	MACROD2	Mono-ADP Ribosylhydrolase 2
1852.	KIAA1586	KIAA1586
1853.	CDK5	Cyclin Dependent Kinase 5
1854.	ATP2A2	ATPase Sarcoplasmic/Endoplasmic Reticulum Ca2+ Transporting 2
1855.	BCHE	Butyrylcholinesterase
1856.	CHAT	Choline O-Acetyltransferase
1857.	PRNP	Prion Protein (Kanno Blood Group)
1858.	BACE1	Beta-Secretase 1
1859.	DDX3X	DEAD-Box Helicase 3 X-Linked
1860.	XPO1	Exportin 1
1861.	AGER	Advanced Glycosylation End-Product Specific Receptor
1862.	PPP1CA	Protein Phosphatase 1 Catalytic Subunit Alpha
1863.	HRH1	Histamine Receptor H1
1864.	IL3	Interleukin 3
1865.	QDPR	Quinoid Dihydropteridine Reductase
1866.	CHMP2B	Charged Multivesicular Body Protein 2B
1867.	KPNA2	Karyopherin Subunit Alpha 2
1868.	TSPO	Translocator Protein
1869.	WNT2	Wnt Family Member 2
1870.	AHCYL1	Adenosylhomocysteinase Like 1
1871.	SELENBP1	Selenium Binding Protein 1
1872.	NTNG1	Netrin G1
1873.	NTNG2	Netrin G2
1874.	PAX4	Paired Box 4
1875.	KISS1	KiSS-1 Metastasis Suppressor
1876.	ALPK1	Alpha Kinase 1
1877.	BFSP2	Beaded Filament Structural Protein 2

1878.	CRYBA4	Crystallin Beta A4
1879.	CRYBB3	Crystallin Beta B3
1880.	CRYGD	Crystallin Gamma D
1881.	BFSP1	Beaded Filament Structural Protein 1
1882.	CRYBB1	Crystallin Beta B1
1883.	NRGN	Neurogranin
1884.	ADPRH	ADP-Ribosylarginine Hydrolase
1885.	B4GAT1	Beta-1,4-Glucuronyltransferase 1
1886.	MOBP	Myelin Associated Oligodendrocyte Basic Protein
1887.	GCM1	Glial Cells Missing Transcription Factor 1
1888.	RBM19	RNA Binding Motif Protein 19
1889.	GLIS1	GLIS Family Zinc Finger 1
1890.	TCF23	Transcription Factor 23
1891.	BCR	BCR Activator Of RhoGEF And GTPase
1892.	GRIN1	Glutamate Ionotropic Receptor NMDA Type Subunit 1
1893.	GLB1	Galactosidase Beta 1
1894.	TUBG1	Tubulin Gamma 1
1895.	USP7	Ubiquitin Specific Peptidase 7
1896.	CTCF	CCCTC-Binding Factor
1897.	SOD2	Superoxide Dismutase 2
1898.	GJB1	Gap Junction Protein Beta 1
1899.	MAG	Myelin Associated Glycoprotein
1900.	SIGMAR1	Sigma Non-Opioid Intracellular Receptor 1
1901.	RBP4	Retinol Binding Protein 4
1902.	MARS1	Methionyl-TRNA Synthetase 1
1903.	MBP	Myelin Basic Protein
1904.	AMPH	Amphiphysin
1905.	NLGN4X	Neuroligin 4 X-Linked
1906.	GJB3	Gap Junction Protein Beta 3
1907.	MYO5B	Myosin VB
1908.	SLC17A7	Solute Carrier Family 17 Member 7
1909.	GJC1	Gap Junction Protein Gamma 1
1910.	CHGB	Chromogranin B
1911.	RFX1	Regulatory Factor X1
1912.	NR2E1	Nuclear Receptor Subfamily 2 Group E Member 1
1913.	DOCK9	Dedicator Of Cytokinesis 9
1914.	GJB5	Gap Junction Protein Beta 5
1915.	EDARADD	EDAR Associated Via Death Domain
1916.	DYNLT2B	Dynein Light Chain Tctex-Type 2B
1917.	IFT25	Intraflagellar Transport 25
1918.	PNMA2	PNMA Family Member 2

1919.	TMEM163	Transmembrane Protein 163
1920.	TUT1	Terminal Uridylyl Transferase 1, U6 SnRNA-Specific
1921.	IRAK4	Interleukin 1 Receptor Associated Kinase 4
1922.	ENO2	Enolase 2
1923.	FCGR2A	Fc Gamma Receptor IIa
1924.	LYZ	Lysozyme
1925.	EDN3	Endothelin 3
1926.	EPX	Eosinophil Peroxidase
1927.	CXCL1	C-X-C Motif Chemokine Ligand 1
1928.	C8B	Complement C8 Beta Chain
1929.	LMOD1	Leiomodin 1
1930.	B3GLCT	Beta 3-Glucosyltransferase
1931.	FOXD3	Forkhead Box D3
1932.	SNURF	SNRPN Upstream Open Reading Frame
1933.	NPAP1	Nuclear Pore Associated Protein 1
1934.	STH	Saitohin
1935.	DCDC1	Doublecortin Domain Containing 1
1936.	VIM	Vimentin
1937.	PKP1	Plakophilin 1
1938.	SERPINB2	Serpin Family B Member 2
1939.	CTNNB1	Catenin Beta 1
1940.	ABL1	ABL Proto-Oncogene 1, Non-Receptor Tyrosine Kinase
1941.	CAMK2A	Calcium/Calmodulin Dependent Protein Kinase II Alpha
1942.	EP300	E1A Binding Protein P300
1943.	PTPRC	Protein Tyrosine Phosphatase Receptor Type C
1944.	SMARCA4	SWI/SNF Related, Matrix Associated, Actin Dependent Regulator Of Chromatin, Subfamily A, Member 4
1945.	EIF2AK2	Eukaryotic Translation Initiation Factor 2 Alpha Kinase 2
1946.	GAD1	Glutamate Decarboxylase 1
1947.	MMP1	Matrix Metallopeptidase 1
1948.	RARA	Retinoic Acid Receptor Alpha
1949.	SLC12A2	Solute Carrier Family 12 Member 2
1950.	CDKN1A	Cyclin Dependent Kinase Inhibitor 1A
1951.	CYBB	Cytochrome B-245 Beta Chain
1952.	FKBP5	FKBP Prolyl Isomerase 5
1953.	GABRB3	Gamma-Aminobutyric Acid Type A Receptor Subunit Beta3
1954.	MAPK3	Mitogen-Activated Protein Kinase 3
1955.	PRKD1	Protein Kinase D1
1956.	TPH2	Tryptophan Hydroxylase 2
1957.	DNM1L	Dynamin 1 Like
1958.	GLI1	GLI Family Zinc Finger 1
1959.	RIGI	RNA Sensor RIG-I

1960.	SLC6A9	Solute Carrier Family 6 Member 9
1961.	TSC1	TSC Complex Subunit 1
1962.	ACTN4	Actinin Alpha 4
1963.	ADCY3	Adenylate Cyclase 3
1964.	CA1	Carbonic Anhydrase 1
1965.	CFB	Complement Factor B
1966.	HEXA	Hexosaminidase Subunit Alpha
1967.	PIK3C2A	Phosphatidylinositol-4-Phosphate 3-Kinase Catalytic Subunit Type 2 Alpha
1968.	SLC6A1	Solute Carrier Family 6 Member 1
1969.	TRPC3	Transient Receptor Potential Cation Channel Subfamily C Member 3
1970.	TRPV6	Transient Receptor Potential Cation Channel Subfamily V Member 6
1971.	ARID1A	AT-Rich Interaction Domain 1A
1972.	CLEC7A	C-Type Lectin Domain Containing 7A
1973.	GAL	Galanin And GMAP Prepropeptide
1974.	NECTIN1	Nectin Cell Adhesion Molecule 1
1975.	NLGN1	Neuroligin 1
1976.	PKD1	Polycystin 1, Transient Receptor Potential Channel Interacting
1977.	TBL1XR1	TBL1X/Y Related 1
1978.	CLCN7	Chloride Voltage-Gated Channel 7
1979.	FOXG1	Forkhead Box G1
1980.	MTTP	Microsomal Triglyceride Transfer Protein
1981.	NTF3	Neurotrophin 3
1982.	SF3B1	Splicing Factor 3b Subunit 1
1983.	SLC1A6	Solute Carrier Family 1 Member 6
1984.	SSTR3	Somatostatin Receptor 3
1985.	VTN	Vitronectin
1986.	COL4A4	Collagen Type IV Alpha 4 Chain
1987.	DGCR8	DGCR8 Microprocessor Complex Subunit
1988.	DHX9	DExH-Box Helicase 9
1989.	EEF1A1	Eukaryotic Translation Elongation Factor 1 Alpha 1
1990.	ENTPD3	Ectonucleoside Triphosphate Diphosphohydrolase 3
1991.	NRXN3	Neurexin 3
1992.	SKIC2	SKI2 Subunit Of Superkiller Complex
1993.	TRPV5	Transient Receptor Potential Cation Channel Subfamily V Member 5
1994.	CA14	Carbonic Anhydrase 14
1995.	CAPN5	Calpain 5
1996.	CCK	Cholecystokinin
1997.	CUL4B	Cullin 4B
1998.	DDX11	DEAD/H-Box Helicase 11
1999.	DNAH11	Dynein Axonemal Heavy Chain 11
2000.	HNRNPU	Heterogeneous Nuclear Ribonucleoprotein U

2001.	NDE1	NudE Neurodevelopment Protein 1
2002.	RBM8A	RNA Binding Motif Protein 8A
2003.	RSPO1	R-Spondin 1
2004.	SRSF1	Serine And Arginine Rich Splicing Factor 1
2005.	AFDN	Afadin, Adherens Junction Formation Factor
2006.	COQ2	Coenzyme Q2, Polyprenyltransferase
2007.	DLGAP1	DLG Associated Protein 1
2008.	SLC17A6	Solute Carrier Family 17 Member 6
2009.	TRH	Thyrotropin Releasing Hormone
2010.	TRPC1	Transient Receptor Potential Cation Channel Subfamily C Member 1
2011.	UBE2D2	Ubiquitin Conjugating Enzyme E2 D2
2012.	CD68	CD68 Molecule
2013.	DNAI1	Dynein Axonemal Intermediate Chain 1
2014.	NDEL1	NudE Neurodevelopment Protein 1 Like 1
2015.	CEP120	Centrosomal Protein 120
2016.	DDX23	DEAD-Box Helicase 23
2017.	DNAH5	Dynein Axonemal Heavy Chain 5
2018.	DYNC2LI1	Dynein Cytoplasmic 2 Light Intermediate Chain 1
2019.	NRXN2	Neurexin 2
2020.	POLR2L	RNA Polymerase II, I And III Subunit L
2021.	CYP3A7	Cytochrome P450 Family 3 Subfamily A Member 7
2022.	HMCN1	Hemicentin 1
2023.	MCOLN3	Mucolipin TRP Cation Channel 3
2024.	OAS3	2'-5'-Oligoadenylate Synthetase 3
2025.	PKHD1	PKHD1 Ciliary IPT Domain Containing Fibrocystin/Polyductin
2026.	UBR5	Ubiquitin Protein Ligase E3 Component N-Recognin 5
2027.	CKMT1B	Creatine Kinase, Mitochondrial 1B
2028.	COPS8	COP9 Signalosome Subunit 8
2029.	CYP2F1	Cytochrome P450 Family 2 Subfamily F Member 1
2030.	DYNC2I1	Dynein 2 Intermediate Chain 1
2031.	DYNC2I2	Dynein 2 Intermediate Chain 2
2032.	PRPS1L1	Phosphoribosyl Pyrophosphate Synthetase 1 Like 1
2033.	COPS6	COP9 Signalosome Subunit 6
2034.	ENTPD8	Ectonucleoside Triphosphate Diphosphohydrolase 8
2035.	MCOLN2	Mucolipin TRP Cation Channel 2
2036.	TPCN1	Two Pore Segment Channel 1
2037.	CDH26	Cadherin 26
2038.	COPS4	COP9 Signalosome Subunit 4
2039.	DPH6	Diphthamine Biosynthesis 6
2040.	KLHDC3	Kelch Domain Containing 3
2041.	QARS1	Glutaminyl-TRNA Synthetase 1

2042.	TCF15	Transcription Factor 15
2043.	ZG16	Zymogen Granule Protein 16
2044.	PWWP2B	PWWP Domain Containing 2B
2045.	CIPC	CLOCK Interacting Pacemaker
2046.	ELOA2	Elongin A2
2047.	ARL17A	ADP Ribosylation Factor Like GTPase 17A
2048.	CFAP46	Cilia And Flagella Associated Protein 46
2049.	LCE4A	Late Cornified Envelope 4A
2050.	RRM2B	Ribonucleotide Reductase Regulatory TP53 Inducible Subunit M2B
2051.	EHMT1	Euchromatic Histone Lysine Methyltransferase 1
2052.	HADHB	Hydroxyacyl-CoA Dehydrogenase Trifunctional Multienzyme Complex Subunit Beta
2053.	PLEC	Plectin
2054.	SETD1A	SET Domain Containing 1A, Histone Lysine Methyltransferase
2055.	CNTN6	Contactin 6
2056.	HKDC1	Hexokinase Domain Containing 1
2057.	BTNL2	Butyrophilin Like 2
2058.	TRAPPC9	Trafficking Protein Particle Complex Subunit 9
2059.	MBD5	Methyl-CpG Binding Domain Protein 5
2060.	PLA2G5	Phospholipase A2 Group V
2061.	TRAPPC3	Trafficking Protein Particle Complex Subunit 3
2062.	GNPTG	N-Acetylglucosamine-1-Phosphate Transferase Subunit Gamma
2063.	OSBPL8	Oxysterol Binding Protein Like 8
2064.	SMG8	SMG8 Nonsense Mediated MRNA Decay Factor
2065.	TSPOAP1	TSPO Associated Protein 1
2066.	ARL6IP6	ADP Ribosylation Factor Like GTPase 6 Interacting Protein 6
2067.	CFAP20	Cilia And Flagella Associated Protein 20
2068.	GAREM2	GRB2 Associated Regulator Of MAPK1 Subtype 2
2069.	ATXN7L3B	Ataxin 7 Like 3B
2070.	PGBD3	PiggyBac Transposable Element Derived 3
2071.	ELANE	Elastase, Neutrophil Expressed
2072.	PSEN2	Presenilin 2
2073.	AFP	Alpha Fetoprotein
2074.	NPHS1	NPHS1 Adhesion Molecule, Nephrin
2075.	FSHB	Follicle Stimulating Hormone Subunit Beta
2076.	LMX1B	LIM Homeobox Transcription Factor 1 Beta
2077.	CCL7	C-C Motif Chemokine Ligand 7
2078.	FCER2	Fc Epsilon Receptor II
2079.	LTBP3	Latent Transforming Growth Factor Beta Binding Protein 3
2080.	S100A8	S100 Calcium Binding Protein A8
2081.	IL16	Interleukin 16
2082.	XPNPEP3	X-Prolyl Aminopeptidase 3

2083.	MUC5AC	Mucin 5AC, Oligomeric Mucus/Gel-Forming
2084.	MAB21L1	Mab-21 Like 1
2085.	S100A12	S100 Calcium Binding Protein A12
2086.	MAB21L2	Mab-21 Like 2
2087.	MPPED2	Metallophosphoesterase Domain Containing 2
2088.	LACRT	Lacritin
2089.	PRRG4	Proline Rich And Gla Domain 4
2090.	ARL14EP	ADP Ribosylation Factor Like GTPase 14 Effector Protein
2091.	GRIA4	Glutamate Ionotropic Receptor AMPA Type Subunit 4
2092.	C2	Complement C2
2093.	OXA1L	OXA1L Mitochondrial Inner Membrane Protein
2094.	IGF1R	Insulin Like Growth Factor 1 Receptor
2095.	DNMT1	DNA Methyltransferase 1
2096.	PIK3CA	Phosphatidylinositol-4,5-Bisphosphate 3-Kinase Catalytic Subunit Alpha
2097.	PPARG	Peroxisome Proliferator Activated Receptor Gamma
2098.	HGF	Hepatocyte Growth Factor
2099.	CDC42	Cell Division Cycle 42
2100.	TUBB3	Tubulin Beta 3 Class III
2101.	GRIK2	Glutamate Ionotropic Receptor Kainate Type Subunit 2
2102.	HSPA8	Heat Shock Protein Family A (Hsp70) Member 8
2103.	NRG1	Neuregulin 1
2104.	NTRK1	Neurotrophic Receptor Tyrosine Kinase 1
2105.	PIK3C3	Phosphatidylinositol 3-Kinase Catalytic Subunit Type 3
2106.	YWHAE	Tyrosine 3-Monooxygenase/Tryptophan 5-Monooxygenase Activation Protein Epsilon
2107.	GABRG2	Gamma-Aminobutyric Acid Type A Receptor Subunit Gamma2
2108.	GLUL	Glutamate-Ammonia Ligase
2109.	USP9X	Ubiquitin Specific Peptidase 9 X-Linked
2110.	ABCC6	ATP Binding Cassette Subfamily C Member 6
2111.	ARSA	Arylsulfatase A
2112.	CASP9	Caspase 9
2113.	IDS	Iduronate 2-Sulfatase
2114.	PIK3CB	Phosphatidylinositol-4,5-Bisphosphate 3-Kinase Catalytic Subunit Beta
2115.	ARSB	Arylsulfatase B
2116.	IHH	Indian Hedgehog Signaling Molecule
2117.	SYP	Synaptophysin
2118.	WNT10B	Wnt Family Member 10B
2119.	XBP1	X-Box Binding Protein 1
2120.	AVPR1A	Arginine Vasopressin Receptor 1A
2121.	GNAO1	G Protein Subunit Alpha O1
2122.	KARS1	Lysyl-TRNA Synthetase 1
2123.	PEPD	Peptidase D

2124.	PRODH	Proline Dehydrogenase 1
2125.	SGSH	N-Sulfoglucosamine Sulfohydrolase
2126.	THBS1	Thrombospondin 1
2127.	TPP1	Tripeptidyl Peptidase 1
2128.	WARS1	Tryptophanyl-TRNA Synthetase 1
2129.	WWOX	WW Domain Containing Oxidoreductase
2130.	ATXN1	Ataxin 1
2131.	CHRNA7	Cholinergic Receptor Nicotinic Alpha 7 Subunit
2132.	CKM	Creatine Kinase, M-Type
2133.	DLX5	Distal-Less Homeobox 5
2134.	E2F4	E2F Transcription Factor 4
2135.	EGR1	Early Growth Response 1
2136.	GRM3	Glutamate Metabotropic Receptor 3
2137.	NFIB	Nuclear Factor I B
2138.	NLGN3	Neuroligin 3
2139.	SLC6A5	Solute Carrier Family 6 Member 5
2140.	SYN1	Synapsin I
2141.	TSPAN7	Tetraspanin 7
2142.	ADCY10	Adenylate Cyclase 10
2143.	CDKL5	Cyclin Dependent Kinase Like 5
2144.	COL4A6	Collagen Type IV Alpha 6 Chain
2145.	DAB1	DAB Adaptor Protein 1
2146.	IL18R1	Interleukin 18 Receptor 1
2147.	NAGLU	N-Acetyl-Alpha-Glucosaminidase
2148.	PCDH19	Protocadherin 19
2149.	PHOX2A	Paired Like Homeobox 2A
2150.	PLP1	Proteolipid Protein 1
2151.	SETDB1	SET Domain Bifurcated Histone Lysine Methyltransferase 1
2152.	SLC32A1	Solute Carrier Family 32 Member 1
2153.	SLC39A8	Solute Carrier Family 39 Member 8
2154.	SLC7A5	Solute Carrier Family 7 Member 5
2155.	YARS1	Tyrosyl-TRNA Synthetase 1
2156.	ZIC2	Zic Family Member 2
2157.	ASCL1	Achaete-Scute Family BHLH Transcription Factor 1
2158.	BMAL1	Basic Helix-Loop-Helix ARNT Like 1
2159.	DNM3	Dynamin 3
2160.	EDA	Ectodysplasin A
2161.	FGF14	Fibroblast Growth Factor 14
2162.	FLCN	Folliculin
2163.	HARS2	Histidyl-TRNA Synthetase 2, Mitochondrial
2164.	RPL21	Ribosomal Protein L21

2165.	SLC1A7	Solute Carrier Family 1 Member 7
2166.	ALOX12B	Arachidonate 12-Lipoxygenase, 12R Type
2167.	ALOXE3	Arachidonate Lipoxygenase 3
2168.	ARHGEF9	Cdc42 Guanine Nucleotide Exchange Factor 9
2169.	ATL1	Atlastin GTPase 1
2170.	BCL9	BCL9 Transcription Coactivator
2171.	CHST14	Carbohydrate Sulfotransferase 14
2172.	CLDN11	Claudin 11
2173.	CNTN4	Contactin 4
2174.	EDAR	Ectodysplasin A Receptor
2175.	GHRL	Ghrelin And Obestatin Prepropeptide
2176.	HTR6	5-Hydroxytryptamine Receptor 6
2177.	NES	Nestin
2178.	OLIG2	Oligodendrocyte Transcription Factor 2
2179.	OSTF1	Osteoclast Stimulating Factor 1
2180.	PEX10	Peroxisomal Biogenesis Factor 10
2181.	POLR3F	RNA Polymerase III Subunit F
2182.	RARS2	Arginyl-TRNA Synthetase 2, Mitochondrial
2183.	SEC23B	SEC23 Homolog B, COPII Coat Complex Component
2184.	SYN3	Synapsin III
2185.	CYFIP1	Cytoplasmic FMR1 Interacting Protein 1
2186.	STX5	Syntaxin 5
2187.	CALB2	Calbindin 2
2188.	DSE	Dermatan Sulfate Epimerase
2189.	FAN1	FANCD2 And FANCI Associated Nuclease 1
2190.	KIF21A	Kinesin Family Member 21A
2191.	MYO1A	Myosin IA
2192.	PCM1	Pericentriolar Material 1
2193.	PEX11B	Peroxisomal Biogenesis Factor 11 Beta
2194.	PEX12	Peroxisomal Biogenesis Factor 12
2195.	RFX2	Regulatory Factor X2
2196.	RFX3	Regulatory Factor X3
2197.	SLC4A5	Solute Carrier Family 4 Member 5
2198.	AP4M1	Adaptor Related Protein Complex 4 Subunit Mu 1
2199.	CIAO1	Cytosolic Iron-Sulfur Assembly Component 1
2200.	COL8A1	Collagen Type VIII Alpha 1 Chain
2201.	DLGAP3	DLG Associated Protein 3
2202.	IPO5	Importin 5
2203.	PEX13	Peroxisomal Biogenesis Factor 13
2204.	PEX16	Peroxisomal Biogenesis Factor 16
2205.	TFDP2	Transcription Factor Dp-2

2206.	TUT4	Terminal Uridylyl Transferase 4
2207.	ANKK1	Ankyrin Repeat And Kinase Domain Containing 1
2208.	CDH7	Cadherin 7
2209.	DLX6	Distal-Less Homeobox 6
2210.	DYNC1I1	Dynein Cytoplasmic 1 Intermediate Chain 1
2211.	ESPN	Espin
2212.	H3C1	H3 Clustered Histone 1
2213.	KCTD13	Potassium Channel Tetramerization Domain Containing 13
2214.	LRP10	LDL Receptor Related Protein 10
2215.	NSFL1C	NSFL1 Cofactor
2216.	SRGAP2	SLIT-ROBO Rho GTPase Activating Protein 2
2217.	TOR2A	Torsin Family 2 Member A
2218.	YTHDC1	YTH N6-Methyladenosine RNA Binding Protein C1
2219.	AP4E1	Adaptor Related Protein Complex 4 Subunit Epsilon 1
2220.	CAPN6	Calpain 6
2221.	CYP4F22	Cytochrome P450 Family 4 Subfamily F Member 22
2222.	EXOC1	Exocyst Complex Component 1
2223.	GNGT2	G Protein Subunit Gamma Transducin 2
2224.	GRHL1	Grainyhead Like Transcription Factor 1
2225.	KCNMB3	Potassium Calcium-Activated Channel Subfamily M Regulatory Beta Subunit 3
2226.	MYO15A	Myosin XVA
2227.	RILPL1	Rab Interacting Lysosomal Protein Like 1
2228.	VCPIP1	Valosin Containing Protein Interacting Protein 1
2229.	ALKBH5	AlkB Homolog 5, RNA Demethylase
2230.	ANKRD49	Ankyrin Repeat Domain 49
2231.	CEP192	Centrosomal Protein 192
2232.	OSBPL1A	Oxysterol Binding Protein Like 1A
2233.	PLLP	Plasmolipin
2234.	STAP2	Signal Transducing Adaptor Family Member 2
2235.	TENT4B	Terminal Nucleotidyltransferase 4B
2236.	TUBA3C	Tubulin Alpha 3c
2237.	CCM2L	CCM2 Like Scaffold Protein
2238.	CORO2A	Coronin 2A
2239.	DCP1B	Decapping MRNA 1B
2240.	EFHC2	EF-Hand Domain Containing 2
2241.	FBXO45	F-Box Protein 45
2242.	FBXW4	F-Box And WD Repeat Domain Containing 4
2243.	FNDC3A	Fibronectin Type III Domain Containing 3A
2244.	RBBP9	RB Binding Protein 9, Serine Hydrolase
2245.	ITGBL1	Integrin Subunit Beta Like 1
2246.	LRRN1	Leucine Rich Repeat Neuronal 1

2247.	LRCH4	Leucine Rich Repeats And Calponin Homology Domain Containing 4
2248.	RABL2A	RAB, Member Of RAS Oncogene Family Like 2A
2249.	SPP2	Secreted Phosphoprotein 2
2250.	TENT2	Terminal Nucleotidyltransferase 2
2251.	ZNF133	Zinc Finger Protein 133
2252.	CCDC85A	Coiled-Coil Domain Containing 85A
2253.	CFAP263	Cilia And Flagella Associated Protein 263
2254.	H3C11	H3 Clustered Histone 11
2255.	H3C4	H3 Clustered Histone 4
2256.	H4C5	H4 Clustered Histone 5
2257.	LRTOMT	Leucine Rich Transmembrane And O-Methyltransferase Domain Containing
2258.	NSG2	Neuronal Vesicle Trafficking Associated 2
2259.	OTOG	Otogelin
2260.	SAMD11	Sterile Alpha Motif Domain Containing 11
2261.	FAM171A2	Family With Sequence Similarity 171 Member A2
2262.	H3C10	H3 Clustered Histone 10
2263.	PET117	PET117 Cytochrome C Oxidase Chaperone
2264.	C1orf56	Chromosome 1 Open Reading Frame 56
2265.	GRID2IP	Grid2 Interacting Protein
2266.	H3C8	H3 Clustered Histone 8
2267.	YAE1	YAE1 Maturation Factor Of ABCE1
2268.	H3C7	H3 Clustered Histone 7
2269.	ZNF639	Zinc Finger Protein 639
2270.	ERICH6B	Glutamate Rich 6B
2271.	BHLHA9	Basic Helix-Loop-Helix Family Member A9
2272.	DUXA	Double Homeobox A
2273.	NUTM2A	NUT Family Member 2A
2274.	SCP2D1	SCP2 Sterol Binding Domain Containing 1
2275.	SPEM2	SPEM Family Member 2
2276.	NUTM2F	NUT Family Member 2F
2277.	SPRR2B	Small Proline Rich Protein 2B
2278.	OPN1MW2	Opsin 1, Medium Wave Sensitive 2
2279.	FGFR2	Fibroblast Growth Factor Receptor 2
2280.	EGFR	Epidermal Growth Factor Receptor
2281.	ERBB2	Erb-B2 Receptor Tyrosine Kinase 2
2282.	FGFR1	Fibroblast Growth Factor Receptor 1
2283.	RET	Ret Proto-Oncogene
2284.	BRAF	B-Raf Proto-Oncogene, Serine/Threonine Kinase
2285.	CCND1	Cyclin D1
2286.	CDK4	Cyclin Dependent Kinase 4
2287.	HDAC6	Histone Deacetylase 6

2288.	KIT	KIT Proto-Oncogene, Receptor Tyrosine Kinase
2289.	MDM2	MDM2 Proto-Oncogene
2290.	PDGFRA	Platelet Derived Growth Factor Receptor Alpha
2291.	PDGFRB	Platelet Derived Growth Factor Receptor Beta
2292.	CTSD	Cathepsin D
2293.	EZH2	Enhancer Of Zeste 2 Polycomb Repressive Complex 2 Subunit
2294.	IDH1	Isocitrate Dehydrogenase (NADP(+)) 1
2295.	KRAS	KRAS Proto-Oncogene, GTPase
2296.	MYC	MYC Proto-Oncogene, BHLH Transcription Factor
2297.	NOTCH1	Notch Receptor 1
2298.	PIK3CD	Phosphatidylinositol-4,5-Bisphosphate 3-Kinase Catalytic Subunit Delta
2299.	AXL	AXL Receptor Tyrosine Kinase
2300.	BAX	BCL2 Associated X, Apoptosis Regulator
2301.	CASR	Calcium Sensing Receptor
2302.	CDH2	Cadherin 2
2303.	CDK6	Cyclin Dependent Kinase 6
2304.	CDKN2A	Cyclin Dependent Kinase Inhibitor 2A
2305.	FOXO1	Forkhead Box O1
2306.	HSP90AA1	Heat Shock Protein 90 Alpha Family Class A Member 1
2307.	IDH2	Isocitrate Dehydrogenase (NADP(+)) 2
2308.	NFE2L2	NFE2 Like BZIP Transcription Factor 2
2309.	NT5E	5'-Nucleotidase Ecto
2310.	SOS1	SOS Ras/Rac Guanine Nucleotide Exchange Factor 1
2311.	SRC	SRC Proto-Oncogene, Non-Receptor Tyrosine Kinase
2312.	TERT	Telomerase Reverse Transcriptase
2313.	ALK	ALK Receptor Tyrosine Kinase
2314.	BCL2	BCL2 Apoptosis Regulator
2315.	CD36	CD36 Molecule (CD36 Blood Group)
2316.	DSP	Desmoplakin
2317.	EPAS1	Endothelial PAS Domain Protein 1
2318.	GRM1	Glutamate Metabotropic Receptor 1
2319.	HDAC1	Histone Deacetylase 1
2320.	HSD11B1	Hydroxysteroid 11-Beta Dehydrogenase 1
2321.	IL2RB	Interleukin 2 Receptor Subunit Beta
2322.	PHGDH	Phosphoglycerate Dehydrogenase
2323.	PRKCG	Protein Kinase C Gamma
2324.	PRKG1	Protein Kinase CGMP-Dependent 1
2325.	PTH1R	Parathyroid Hormone 1 Receptor
2326.	RHOA	Ras Homolog Family Member A
2327.	RUNX1	RUNX Family Transcription Factor 1
2328.	TFRC	Transferrin Receptor

2329.	TOP2A	DNA Topoisomerase II Alpha
2330.	ABCA1	ATP Binding Cassette Subfamily A Member 1
2331.	ANGPT2	Angiopoietin 2
2332.	COL2A1	Collagen Type II Alpha 1 Chain
2333.	EIF2AK3	Eukaryotic Translation Initiation Factor 2 Alpha Kinase 3
2334.	ESR2	Estrogen Receptor 2
2335.	FZD4	Frizzled Class Receptor 4
2336.	GLI2	GLI Family Zinc Finger 2
2337.	GLI3	GLI Family Zinc Finger 3
2338.	GRIN2D	Glutamate Ionotropic Receptor NMDA Type Subunit 2D
2339.	HSP90AB1	Heat Shock Protein 90 Alpha Family Class B Member 1
2340.	HSPA5	Heat Shock Protein Family A (Hsp70) Member 5
2341.	KCNQ1	Potassium Voltage-Gated Channel Subfamily Q Member 1
2342.	KDM1A	Lysine Demethylase 1A
2343.	KRT18	Keratin 18
2344.	LRP5	LDL Receptor Related Protein 5
2345.	MAPK8	Mitogen-Activated Protein Kinase 8
2346.	MUSK	Muscle Associated Receptor Tyrosine Kinase
2347.	NR3C1	Nuclear Receptor Subfamily 3 Group C Member 1
2348.	PIK3CG	Phosphatidylinositol-4,5-Bisphosphate 3-Kinase Catalytic Subunit Gamma
2349.	PIM1	Pim-1 Proto-Oncogene, Serine/Threonine Kinase
2350.	PKM	Pyruvate Kinase M1/2
2351.	TLR8	Toll Like Receptor 8
2352.	ASAH1	N-Acylsphingosine Amidohydrolase 1
2353.	BCL2L1	BCL2 Like 1
2354.	CD44	CD44 Molecule (IN Blood Group)
2355.	CYCS	Cytochrome C, Somatic
2356.	DICER1	Dicer 1, Ribonuclease III
2357.	FGF8	Fibroblast Growth Factor 8
2358.	HMGB1	High Mobility Group Box 1
2359.	IFIH1	Interferon Induced With Helicase C Domain 1
2360.	MFN2	Mitofusin 2
2361.	PGK1	Phosphoglycerate Kinase 1
2362.	PROS1	Protein S
2363.	SDHB	Succinate Dehydrogenase Complex Iron Sulfur Subunit B
2364.	SLC12A5	Solute Carrier Family 12 Member 5
2365.	TLR1	Toll Like Receptor 1
2366.	TLR7	Toll Like Receptor 7
2367.	TUBA1A	Tubulin Alpha 1a
2368.	VDR	Vitamin D Receptor
2369.	AQP1	Aquaporin 1 (Colton Blood Group)

2370.	CAPN2	Calpain 2
2371.	CDK1	Cyclin Dependent Kinase 1
2372.	CLU	Clusterin
2373.	ERN1	Endoplasmic Reticulum To Nucleus Signaling 1
2374.	FGF10	Fibroblast Growth Factor 10
2375.	GABBR1	Gamma-Aminobutyric Acid Type B Receptor Subunit 1
2376.	GALK1	Galactokinase 1
2377.	GGCX	Gamma-Glutamyl Carboxylase
2378.	GNRHR	Gonadotropin Releasing Hormone Receptor
2379.	KAT2A	Lysine Acetyltransferase 2A
2380.	KAT2B	Lysine Acetyltransferase 2B
2381.	KEAP1	Kelch Like ECH Associated Protein 1
2382.	LOX	Lysyl Oxidase
2383.	MAPKAPK3	MAPK Activated Protein Kinase 3
2384.	MIF	Macrophage Migration Inhibitory Factor
2385.	MMP8	Matrix Metallopeptidase 8
2386.	MYB	MYB Proto-Oncogene, Transcription Factor
2387.	NGFR	Nerve Growth Factor Receptor
2388.	OAT	Ornithine Aminotransferase
2389.	PRKAB1	Protein Kinase AMP-Activated Non-Catalytic Subunit Beta 1
2390.	PRPS1	Phosphoribosyl Pyrophosphate Synthetase 1
2391.	PTPRJ	Protein Tyrosine Phosphatase Receptor Type J
2392.	RDX	Radixin
2393.	SMC1A	Structural Maintenance Of Chromosomes 1A
2394.	STX1A	Syntaxin 1A
2395.	TUBA4A	Tubulin Alpha 4a
2396.	TUBB4A	Tubulin Beta 4A Class IVa
2397.	TYRO3	TYRO3 Protein Tyrosine Kinase
2398.	YAP1	Yes1 Associated Transcriptional Regulator
2399.	ADRA2A	Adrenoceptor Alpha 2A
2400.	APOB	Apolipoprotein B
2401.	ATF4	Activating Transcription Factor 4
2402.	ATRX	ATRX Chromatin Remodeler
2403.	ATXN3	Ataxin 3
2404.	BECN1	Beclin 1
2405.	CD27	CD27 Molecule
2406.	COMP	Cartilage Oligomeric Matrix Protein
2407.	CTNNA1	Catenin Alpha 1
2408.	CYP27B1	Cytochrome P450 Family 27 Subfamily B Member 1
2409.	DCX	Doublecortin
2410.	DDIT3	DNA Damage Inducible Transcript 3

2411.	DVL1	Dishevelled Segment Polarity Protein 1
2412.	FBLN5	Fibulin 5
2413.	FTO	FTO Alpha-Ketoglutarate Dependent Dioxygenase
2414.	FXN	Frataxin
2415.	GNA11	G Protein Subunit Alpha 11
2416.	HADH	Hydroxyacyl-CoA Dehydrogenase
2417.	HSD17B4	Hydroxysteroid 17-Beta Dehydrogenase 4
2418.	HSP90B1	Heat Shock Protein 90 Beta Family Member 1
2419.	HUWE1	HECT, UBA And WWE Domain Containing E3 Ubiquitin Protein Ligase 1
2420.	MYCN	MYCN Proto-Oncogene, BHLH Transcription Factor
2421.	NEDD4	NEDD4 E3 Ubiquitin Protein Ligase
2422.	NOG	Noggin
2423.	NOTCH4	Notch Receptor 4
2424.	P2RX7	Purinergic Receptor P2X 7
2425.	PMM2	Phosphomannomutase 2
2426.	PPARGC1A	PPARG Coactivator 1 Alpha
2427.	PTGDR	Prostaglandin D2 Receptor
2428.	SEMA3A	Semaphorin 3A
2429.	SLC11A1	Solute Carrier Family 11 Member 1
2430.	SLC5A1	Solute Carrier Family 5 Member 1
2431.	SLC6A2	Solute Carrier Family 6 Member 2
2432.	SPTLC2	Serine Palmitoyltransferase Long Chain Base Subunit 2
2433.	TNFRSF13B	TNF Receptor Superfamily Member 13B
2434.	TXN	Thioredoxin
2435.	VCAN	Versican
2436.	ALDOB	Aldolase, Fructose-Bisphosphate B
2437.	AMACR	Alpha-Methylacyl-CoA Racemase
2438.	ANTXR2	ANTXR Cell Adhesion Molecule 2
2439.	ATG5	Autophagy Related 5
2440.	ATP5F1A	ATP Synthase F1 Subunit Alpha
2441.	AVP	Arginine Vasopressin
2442.	AVPR2	Arginine Vasopressin Receptor 2
2443.	CDKN2B	Cyclin Dependent Kinase Inhibitor 2B
2444.	DDX5	DEAD-Box Helicase 5
2445.	DGKE	Diacylglycerol Kinase Epsilon
2446.	EPB41	Erythrocyte Membrane Protein Band 4.1
2447.	ETV6	ETS Variant Transcription Factor 6
2448.	EWSR1	EWS RNA Binding Protein 1
2449.	EZR	Ezrin
2450.	FGF17	Fibroblast Growth Factor 17
2451.	FHL1	Four And A Half LIM Domains 1

2452.	FOSL1	FOS Like 1, AP-1 Transcription Factor Subunit
2453.	GABRA4	Gamma-Aminobutyric Acid Type A Receptor Subunit Alpha4
2454.	GLO1	Glyoxalase I
2455.	GPT2	Glutamic--Pyruvic Transaminase 2
2456.	GRM7	Glutamate Metabotropic Receptor 7
2457.	GUCY2C	Guanylate Cyclase 2C
2458.	HCN2	Hyperpolarization Activated Cyclic Nucleotide Gated Potassium And Sodium Channel 2
2459.	HNMT	Histamine N-Methyltransferase
2460.	HNRNPA2B1	Heterogeneous Nuclear Ribonucleoprotein A2/B1
2461.	IRS2	Insulin Receptor Substrate 2
2462.	KCNC3	Potassium Voltage-Gated Channel Subfamily C Member 3
2463.	KIF5B	Kinesin Family Member 5B
2464.	MMUT	Methylmalonyl-CoA Mutase
2465.	MYH14	Myosin Heavy Chain 14
2466.	PARP2	Poly(ADP-Ribose) Polymerase 2
2467.	PHB1	Prohibitin 1
2468.	SI	Sucrase-Isomaltase
2469.	SIAH1	Siah E3 Ubiquitin Protein Ligase 1
2470.	SLC26A3	Solute Carrier Family 26 Member 3
2471.	SPP1	Secreted Phosphoprotein 1
2472.	TFAM	Transcription Factor A, Mitochondrial
2473.	TLR6	Toll Like Receptor 6
2474.	TPM3	Tropomyosin 3
2475.	ATF3	Activating Transcription Factor 3
2476.	BSG	Basigin (Ok Blood Group)
2477.	CA12	Carbonic Anhydrase 12
2478.	CCR7	C-C Motif Chemokine Receptor 7
2479.	CHD4	Chromodomain Helicase DNA Binding Protein 4
2480.	CLOCK	Clock Circadian Regulator
2481.	CNTN1	Contactin 1
2482.	COL6A1	Collagen Type VI Alpha 1 Chain
2483.	CSF2RB	Colony Stimulating Factor 2 Receptor Subunit Beta
2484.	CX3CL1	C-X3-C Motif Chemokine Ligand 1
2485.	CX3CR1	C-X3-C Motif Chemokine Receptor 1
2486.	DNAJB1	DnaJ Heat Shock Protein Family (Hsp40) Member B1
2487.	DROSHA	Drosha Ribonuclease III
2488.	E2F1	E2F Transcription Factor 1
2489.	ECM1	Extracellular Matrix Protein 1
2490.	EFEMP2	EGF Containing Fibulin Extracellular Matrix Protein 2
2491.	EGLN2	Egl-9 Family Hypoxia Inducible Factor 2
2492.	FBLN1	Fibulin 1

2493.	FGF3	Fibroblast Growth Factor 3
2494.	FGF4	Fibroblast Growth Factor 4
2495.	FGF5	Fibroblast Growth Factor 5
2496.	FGF9	Fibroblast Growth Factor 9
2497.	FOXP1	Forkhead Box P1
2498.	FXR1	FMR1 Autosomal Homolog 1
2499.	H6PD	Hexose-6-Phosphate Dehydrogenase/Glucose 1-Dehydrogenase
2500.	IFNGR2	Interferon Gamma Receptor 2
2501.	IGF2BP2	Insulin Like Growth Factor 2 MRNA Binding Protein 2
2502.	IGFBP2	Insulin Like Growth Factor Binding Protein 2
2503.	ISL1	ISL LIM Homeobox 1
2504.	LIF	LIF Interleukin 6 Family Cytokine
2505.	NCL	Nucleolin
2506.	NRP2	Neuropilin 2
2507.	OAS1	2'-5'-Oligoadenylate Synthetase 1
2508.	PDE10A	Phosphodiesterase 10A
2509.	PDE2A	Phosphodiesterase 2A
2510.	PEX1	Peroxisomal Biogenesis Factor 1
2511.	PTGIS	Prostaglandin I2 Synthase
2512.	RAG1	Recombination Activating 1
2513.	RAP1A	RAP1A, Member Of RAS Oncogene Family
2514.	RBBP4	RB Binding Protein 4, Chromatin Remodeling Factor
2515.	S100A9	S100 Calcium Binding Protein A9
2516.	SCO2	Synthesis Of Cytochrome C Oxidase 2
2517.	SFTPD	Surfactant Protein D
2518.	SLC1A5	Solute Carrier Family 1 Member 5
2519.	SLC7A7	Solute Carrier Family 7 Member 7
2520.	TUBB2B	Tubulin Beta 2B Class IIb
2521.	UBC	Ubiquitin C
2522.	UGCG	UDP-Glucose Ceramide Glucosyltransferase
2523.	ULK1	Unc-51 Like Autophagy Activating Kinase 1
2524.	VAPB	VAMP Associated Protein B And C
2525.	ADGRE5	Adhesion G Protein-Coupled Receptor E5
2526.	ADIPOR1	Adiponectin Receptor 1
2527.	ANXA11	Annexin A11
2528.	ATG7	Autophagy Related 7
2529.	CNTFR	Ciliary Neurotrophic Factor Receptor
2530.	COL11A1	Collagen Type XI Alpha 1 Chain
2531.	COL7A1	Collagen Type VII Alpha 1 Chain
2532.	COQ8A	Coenzyme Q8A
2533.	CYP21A2	Cytochrome P450 Family 21 Subfamily A Member 2

2534.	DLG1	Discs Large MAGUK Scaffold Protein 1
2535.	EIF3A	Eukaryotic Translation Initiation Factor 3 Subunit A
2536.	ELN	Elastin
2537.	EPO	Erythropoietin
2538.	EXOSC3	Exosome Component 3
2539.	FBXW11	F-Box And WD Repeat Domain Containing 11
2540.	FGF7	Fibroblast Growth Factor 7
2541.	GAS6	Growth Arrest Specific 6
2542.	HMGA2	High Mobility Group AT-Hook 2
2543.	HTR4	5-Hydroxytryptamine Receptor 4
2544.	IL9	Interleukin 9
2545.	KREMEN1	Kringle Containing Transmembrane Protein 1
2546.	LEFTY2	Left-Right Determination Factor 2
2547.	LGR5	Leucine Rich Repeat Containing G Protein-Coupled Receptor 5
2548.	MGAM	Maltase-Glucoamylase
2549.	MYT1L	Myelin Transcription Factor 1 Like
2550.	NFASC	Neurofascin
2551.	OPHN1	Oligophrenin 1
2552.	PEX19	Peroxisomal Biogenesis Factor 19
2553.	PEX7	Peroxisomal Biogenesis Factor 7
2554.	PLXNA2	Plexin A2
2555.	POLR3B	RNA Polymerase III Subunit B
2556.	POU3F2	POU Class 3 Homeobox 2
2557.	PPP1R1B	Protein Phosphatase 1 Regulatory Inhibitor Subunit 1B
2558.	RNF2	Ring Finger Protein 2
2559.	SEC63	SEC63 Homolog, Protein Translocation Regulator
2560.	SFPQ	Splicing Factor Proline And Glutamine Rich
2561.	SLC26A4	Solute Carrier Family 26 Member 4
2562.	SLC39A14	Solute Carrier Family 39 Member 14
2563.	SOX4	SRY-Box Transcription Factor 4
2564.	SRF	Serum Response Factor
2565.	SSTR1	Somatostatin Receptor 1
2566.	SUFU	SUFU Negative Regulator Of Hedgehog Signaling
2567.	THY1	Thy-1 Cell Surface Antigen
2568.	U2AF1	U2 Small Nuclear RNA Auxiliary Factor 1
2569.	UGT8	UDP Glycosyltransferase 8
2570.	VANGL2	VANGL Planar Cell Polarity Protein 2
2571.	VEGFB	Vascular Endothelial Growth Factor B
2572.	AARS2	Alanyl-TRNA Synthetase 2, Mitochondrial
2573.	AIP	Aryl Hydrocarbon Receptor Interacting Protein
2574.	ASPA	Aspartoacylase

2575.	BANF1	BAF Nuclear Assembly Factor 1
2576.	BHMT	Betaine--Homocysteine S-Methyltransferase
2577.	CD2AP	CD2 Associated Protein
2578.	CKMT2	Creatine Kinase, Mitochondrial 2
2579.	CLPX	Caseinolytic Mitochondrial Matrix Peptidase Chaperone Subunit X
2580.	CNTNAP1	Contactin Associated Protein 1
2581.	COQ6	Coenzyme Q6, Monooxygenase
2582.	CUL5	Cullin 5
2583.	CYP26A1	Cytochrome P450 Family 26 Subfamily A Member 1
2584.	CYP51A1	Cytochrome P450 Family 51 Subfamily A Member 1
2585.	DNAJC5	DnaJ Heat Shock Protein Family (Hsp40) Member C5
2586.	DNTT	DNA Nucleotidylexotransferase
2587.	DYNLL1	Dynein Light Chain LC8-Type 1
2588.	ENTPD2	Ectonucleoside Triphosphate Diphosphohydrolase 2
2589.	FADS1	Fatty Acid Desaturase 1
2590.	FGF18	Fibroblast Growth Factor 18
2591.	FLOT1	Flotillin 1
2592.	GM2A	Ganglioside GM2 Activator
2593.	GNPTAB	N-Acetylglucosamine-1-Phosphate Transferase Subunits Alpha And Beta
2594.	GOSR2	Golgi SNAP Receptor Complex Member 2
2595.	GRIK3	Glutamate Ionotropic Receptor Kainate Type Subunit 3
2596.	HCRTR1	Hypocretin Receptor 1
2597.	HGFAC	HGF Activator
2598.	HNRNPDL	Heterogeneous Nuclear Ribonucleoprotein D Like
2599.	HOMER1	Homer Scaffold Protein 1
2600.	HTR3B	5-Hydroxytryptamine Receptor 3B
2601.	IVNS1ABP	Influenza Virus NS1A Binding Protein
2602.	KCNQ4	Potassium Voltage-Gated Channel Subfamily Q Member 4
2603.	KLC1	Kinesin Light Chain 1
2604.	KRT16	Keratin 16
2605.	MDH1	Malate Dehydrogenase 1
2606.	MYO6	Myosin VI
2607.	NDP	Norrin Cystine Knot Growth Factor NDP
2608.	NPY2R	Neuropeptide Y Receptor Y2
2609.	P2RY11	Purinergic Receptor P2Y11
2610.	PABPC1	Poly(A) Binding Protein Cytoplasmic 1
2611.	POLR1A	RNA Polymerase I Subunit A
2612.	PSMC5	Proteasome 26S Subunit, ATPase 5
2613.	PSMD3	Proteasome 26S Subunit, Non-ATPase 3
2614.	RICTOR	RPTOR Independent Companion Of MTOR Complex 2
2615.	RNF216	Ring Finger Protein 216

2616.	SDHC	Succinate Dehydrogenase Complex Subunit C
2617.	SERPINE2	Serpin Family E Member 2
2618.	SF3B4	Splicing Factor 3b Subunit 4
2619.	SLC3A2	Solute Carrier Family 3 Member 2
2620.	SLC7A9	Solute Carrier Family 7 Member 9
2621.	TBR1	T-Box Brain Transcription Factor 1
2622.	TFE3	Transcription Factor Binding To IGHM Enhancer 3
2623.	TMC6	Transmembrane Channel Like 6
2624.	TPX2	TPX2 Microtubule Nucleation Factor
2625.	UBTF	Upstream Binding Transcription Factor
2626.	ADAMTSL1	ADAMTS Like 1
2627.	BPI	Bactericidal Permeability Increasing Protein
2628.	CA3	Carbonic Anhydrase 3
2629.	CA5A	Carbonic Anhydrase 5A
2630.	CA6	Carbonic Anhydrase 6
2631.	CCT7	Chaperonin Containing TCP1 Subunit 7
2632.	CDK20	Cyclin Dependent Kinase 20
2633.	CILK1	Ciliogenesis Associated Kinase 1
2634.	CLN5	CLN5 Intracellular Trafficking Protein
2635.	COCH	Cochlin
2636.	CRYM	Crystallin Mu
2637.	CYTH1	Cytohesin 1
2638.	DDX17	DEAD-Box Helicase 17
2639.	DGCR2	DiGeorge Syndrome Critical Region Gene 2
2640.	EFHC1	EF-Hand Domain Containing 1
2641.	EFTUD2	Elongation Factor Tu GTP Binding Domain Containing 2
2642.	ELAVL1	ELAV Like RNA Binding Protein 1
2643.	ELOVL5	ELOVL Fatty Acid Elongase 5
2644.	EXOSC5	Exosome Component 5
2645.	EXOSC9	Exosome Component 9
2646.	FGF13	Fibroblast Growth Factor 13
2647.	GATAD2B	GATA Zinc Finger Domain Containing 2B
2648.	GPX2	Glutathione Peroxidase 2
2649.	HNRNPC	Heterogeneous Nuclear Ribonucleoprotein C
2650.	IFITM3	Interferon Induced Transmembrane Protein 3
2651.	IRS4	Insulin Receptor Substrate 4
2652.	KLK7	Kallikrein Related Peptidase 7
2653.	MAPKAP1	MAPK Associated Protein 1
2654.	METTL3	Methyltransferase 3, N6-Adenosine-Methyltransferase Complex Catalytic Subunit
2655.	MNAT1	MNAT1 Component Of CDK Activating Kinase
2656.	MX1	MX Dynamin Like GTPase 1

2657.	NLGN2	Neuroligin 2
2658.	NTS	Neurotensin
2659.	PDPN	Podoplanin
2660.	PDSS1	Decaprenyl Diphosphate Synthase Subunit 1
2661.	PDSS2	Decaprenyl Diphosphate Synthase Subunit 2
2662.	PIWIL1	Piwi Like RNA-Mediated Gene Silencing 1
2663.	PLEKHM1	Pleckstrin Homology And RUN Domain Containing M1
2664.	POLG2	DNA Polymerase Gamma 2, Accessory Subunit
2665.	POLR2B	RNA Polymerase II Subunit B
2666.	PSMD1	Proteasome 26S Subunit, Non-ATPase 1
2667.	PTBP1	Polypyrimidine Tract Binding Protein 1
2668.	RAB6A	RAB6A, Member RAS Oncogene Family
2669.	RAG2	Recombination Activating 2
2670.	RBMX	RNA Binding Motif Protein X-Linked
2671.	RHOBTB2	Rho Related BTB Domain Containing 2
2672.	SCGB1A1	Secretoglobin Family 1A Member 1
2673.	SECISBP2	SECIS Binding Protein 2
2674.	SF3B2	Splicing Factor 3b Subunit 2
2675.	SH3KBP1	SH3 Domain Containing Kinase Binding Protein 1
2676.	SLC15A1	Solute Carrier Family 15 Member 1
2677.	SLC30A10	Solute Carrier Family 30 Member 10
2678.	SLC30A2	Solute Carrier Family 30 Member 2
2679.	SLC39A7	Solute Carrier Family 39 Member 7
2680.	SLITRK1	SLIT And NTRK Like Family Member 1
2681.	SMARCA5	SWI/SNF Related, Matrix Associated, Actin Dependent Regulator Of Chromatin, Subfamily A, Member 5
2682.	SPINT1	Serine Peptidase Inhibitor, Kunitz Type 1
2683.	SPINT2	Serine Peptidase Inhibitor, Kunitz Type 2
2684.	SPRED2	Sprouty Related EVH1 Domain Containing 2
2685.	STING1	Stimulator Of Interferon Response CGAMP Interactor 1
2686.	SURF1	SURF1 Cytochrome C Oxidase Assembly Factor
2687.	TCP1	T-Complex 1
2688.	TMPRSS3	Transmembrane Serine Protease 3
2689.	TNPO1	Transportin 1
2690.	TRNT1	TRNA Nucleotidyl Transferase 1
2691.	TTPA	Alpha Tocopherol Transfer Protein
2692.	TWNK	Twinkle MtDNA Helicase
2693.	TXNL4A	Thioredoxin Like 4A
2694.	CHRD	Chordin
2695.	CLEC4M	C-Type Lectin Domain Family 4 Member M
2696.	CNOT3	CCR4-NOT Transcription Complex Subunit 3
2697.	DSG4	Desmoglein 4

2698.	EML1	EMAP Like 1
2699.	EXOC5	Exocyst Complex Component 5
2700.	EXOC7	Exocyst Complex Component 7
2701.	EXOSC2	Exosome Component 2
2702.	G3BP1	G3BP Stress Granule Assembly Factor 1
2703.	GABARAPL1	GABA Type A Receptor Associated Protein Like 1
2704.	GBA2	Glucosylceramidase Beta 2
2705.	H3-3B	H3.3 Histone B
2706.	IFITM1	Interferon Induced Transmembrane Protein 1
2707.	ITIH3	Inter-Alpha-Trypsin Inhibitor Heavy Chain 3
2708.	MCPH1	Microcephalin 1
2709.	MYO3A	Myosin IIIA
2710.	PANK1	Pantothenate Kinase 1
2711.	RPL4	Ribosomal Protein L4
2712.	RSAD2	Radical S-Adenosyl Methionine Domain Containing 2
2713.	SFRP2	Secreted Frizzled Related Protein 2
2714.	SLC39A13	Solute Carrier Family 39 Member 13
2715.	SLC39A6	Solute Carrier Family 39 Member 6
2716.	SLC6A17	Solute Carrier Family 6 Member 17
2717.	SP100	SP100 Nuclear Antigen
2718.	TBCE	Tubulin Folding Cofactor E
2719.	TGM5	Transglutaminase 5
2720.	TSPAN12	Tetraspanin 12
2721.	USP22	Ubiquitin Specific Peptidase 22
2722.	VEGFD	Vascular Endothelial Growth Factor D
2723.	YBX1	Y-Box Binding Protein 1
2724.	ADGRL3	Adhesion G Protein-Coupled Receptor L3
2725.	AGRP	Agouti Related Neuropeptide
2726.	ANKS6	Ankyrin Repeat And Sterile Alpha Motif Domain Containing 6
2727.	ASAP1	ArfGAP With SH3 Domain, Ankyrin Repeat And PH Domain 1
2728.	CELF4	CUGBP Elav-Like Family Member 4
2729.	CLDN5	Claudin 5
2730.	COPS3	COP9 Signalosome Subunit 3
2731.	CXCL5	C-X-C Motif Chemokine Ligand 5
2732.	DACT1	Dishevelled Binding Antagonist Of Beta Catenin 1
2733.	DCDC2	Doublecortin Domain Containing 2
2734.	DCTN2	Dynactin Subunit 2
2735.	DHX36	DEAH-Box Helicase 36
2736.	DPP3	Dipeptidyl Peptidase 3
2737.	EDN2	Endothelin 2
2738.	EHD1	EH Domain Containing 1

2739.	EIF1AX	Eukaryotic Translation Initiation Factor 1A X-Linked
2740.	EVC2	EvC Ciliary Complex Subunit 2
2741.	EXOSC8	Exosome Component 8
2742.	FGF16	Fibroblast Growth Factor 16
2743.	FGF20	Fibroblast Growth Factor 20
2744.	FGF6	Fibroblast Growth Factor 6
2745.	FLVCR2	FLVCR Choline And Putative Heme Transporter 2
2746.	GABRR2	Gamma-Aminobutyric Acid Type A Receptor Subunit Rho2
2747.	GLIS2	GLIS Family Zinc Finger 2
2748.	GPR65	G Protein-Coupled Receptor 65
2749.	GTF2B	General Transcription Factor IIB
2750.	GTPBP1	GTP Binding Protein 1
2751.	GUK1	Guanylate Kinase 1
2752.	HR	HR Lysine Demethylase And Nuclear Receptor Corepressor
2753.	IGF2BP1	Insulin Like Growth Factor 2 MRNA Binding Protein 1
2754.	JPH1	Junctophilin 1
2755.	KLB	Klotho Beta
2756.	KRT6B	Keratin 6B
2757.	LIN28A	Lin-28 Homolog A
2758.	LMOD3	Leiomodin 3
2759.	LSM2	LSM2 Homolog, U6 Small Nuclear RNA And MRNA Degradation Associated
2760.	NBAS	NBAS Subunit Of NRZ Tethering Complex
2761.	NEUROG3	Neurogenin 3
2762.	NLRP5	NLR Family Pyrin Domain Containing 5
2763.	NME8	NME/NM23 Family Member 8
2764.	NUCB1	Nucleobindin 1
2765.	PIGN	Phosphatidylinositol Glycan Anchor Biosynthesis Class N
2766.	POU3F3	POU Class 3 Homeobox 3
2767.	RBPMS	RNA Binding Protein, MRNA Processing Factor
2768.	SCRIB	Scribble Planar Cell Polarity Protein
2769.	SHANK1	SH3 And Multiple Ankyrin Repeat Domains 1
2770.	SLC30A1	Solute Carrier Family 30 Member 1
2771.	SLC38A2	Solute Carrier Family 38 Member 2
2772.	SLC39A1	Solute Carrier Family 39 Member 1
2773.	SLC6A14	Solute Carrier Family 6 Member 14
2774.	SMARCA1	SWI/SNF Related, Matrix Associated, Actin Dependent Regulator Of Chromatin, Subfamily A, Member 1
2775.	SRSF2	Serine And Arginine Rich Splicing Factor 2
2776.	TBL1X	Transducin Beta Like 1 X-Linked
2777.	TGM4	Transglutaminase 4
2778.	TMEM70	Transmembrane Protein 70
2779.	UVRAG	UV Radiation Resistance Associated

2780.	AADAT	Aminoadipate Aminotransferase
2781.	ANO2	Anoctamin 2
2782.	ATPAF2	ATP Synthase Mitochondrial F1 Complex Assembly Factor 2
2783.	BAG6	BAG Cochaperone 6
2784.	BBOX1	Gamma-Butyrobetaine Hydroxylase 1
2785.	BRAT1	BRCA1 Associated ATM Activator 1
2786.	C5AR2	Complement C5a Receptor 2
2787.	CA5B	Carbonic Anhydrase 5B
2788.	CEACAM8	CEA Cell Adhesion Molecule 8
2789.	CENPB	Centromere Protein B
2790.	CLIC5	Chloride Intracellular Channel 5
2791.	CLN6	CLN6 Transmembrane ER Protein
2792.	COQ4	Coenzyme Q4
2793.	COQ8B	Coenzyme Q8B
2794.	CPSF4	Cleavage And Polyadenylation Specific Factor 4
2795.	CXCR6	C-X-C Motif Chemokine Receptor 6
2796.	DNAI2	Dynein Axonemal Intermediate Chain 2
2797.	EXOSC1	Exosome Component 1
2798.	EXOSC10	Exosome Component 10
2799.	FBXO3	F-Box Protein 3
2800.	FOXJ1	Forkhead Box J1
2801.	FUZ	Fuzzy Planar Cell Polarity Protein
2802.	GHRH	Growth Hormone Releasing Hormone
2803.	GP2	Glycoprotein 2
2804.	GPX8	Glutathione Peroxidase 8 (Putative)
2805.	H3-4	H3.4 Histone, Cluster Member
2806.	HYDIN	HYDIN Axonemal Central Pair Apparatus Protein
2807.	IL17RC	Interleukin 17 Receptor C
2808.	IMMP2L	Inner Mitochondrial Membrane Peptidase Subunit 2
2809.	IVL	Involucrin
2810.	KANSL1	KAT8 Regulatory NSL Complex Subunit 1
2811.	KCNJ16	Potassium Inwardly Rectifying Channel Subfamily J Member 16
2812.	KPNA4	Karyopherin Subunit Alpha 4
2813.	LGALS4	Galectin 4
2814.	MAGEL2	MAGE Family Member L2
2815.	NAP1L4	Nucleosome Assembly Protein 1 Like 4
2816.	NDUFAF3	NADH:Ubiquinone Oxidoreductase Complex Assembly Factor 3
2817.	NOS1AP	Nitric Oxide Synthase 1 Adaptor Protein
2818.	NUS1	NUS1 Dehydrodolichyl Diphosphate Synthase Subunit
2819.	PBX2	PBX Homeobox 2
2820.	PLVAP	Plasmalemma Vesicle Associated Protein

2821.	RCN2	Reticulocalbin 2
2822.	RPL27A	Ribosomal Protein L27a
2823.	SKIC3	SKI3 Subunit Of Superkiller Complex
2824.	SLC30A5	Solute Carrier Family 30 Member 5
2825.	SLC39A10	Solute Carrier Family 39 Member 10
2826.	SLC7A8	Solute Carrier Family 7 Member 8
2827.	SPTLC3	Serine Palmitoyltransferase Long Chain Base Subunit 3
2828.	TAAR1	Trace Amine Associated Receptor 1
2829.	TAF10	TATA-Box Binding Protein Associated Factor 10
2830.	TBX19	T-Box Transcription Factor 19
2831.	TEAD3	TEA Domain Transcription Factor 3
2832.	TOR1B	Torsin Family 1 Member B
2833.	TSPAN1	Tetraspanin 1
2834.	U2AF2	U2 Small Nuclear RNA Auxiliary Factor 2
2835.	UBR4	Ubiquitin Protein Ligase E3 Component N-Recognin 4
2836.	ZDHHC8	Zinc Finger DHHC-Type Palmitoyltransferase 8
2837.	ZP2	Zona Pellucida Glycoprotein 2
2838.	AGPAT3	1-Acylglycerol-3-Phosphate O-Acyltransferase 3
2839.	AOC2	Amine Oxidase Copper Containing 2
2840.	ARHGEF11	Rho Guanine Nucleotide Exchange Factor 11
2841.	ARMC9	Armadillo Repeat Containing 9
2842.	ARVCF	ARVCF Delta Catenin Family Member
2843.	CAND1	Cullin Associated And Neddylation Dissociated 1
2844.	CDC42EP3	CDC42 Effector Protein 3
2845.	CENPA	Centromere Protein A
2846.	CLEC6A	C-Type Lectin Domain Containing 6A
2847.	CTR9	CTR9 Homolog, Paf1/RNA Polymerase II Complex Component
2848.	DCAF1	DDB1 And CUL4 Associated Factor 1
2849.	DNAAF4	Dynein Axonemal Assembly Factor 4
2850.	ELOA	Elongin A
2851.	ENSA	Endosulfine Alpha
2852.	EVC	EvC Ciliary Complex Subunit 1
2853.	GLT8D1	Glycosyltransferase 8 Domain Containing 1
2854.	GTF2E1	General Transcription Factor IIE Subunit 1
2855.	IFT56	Intraflagellar Transport 56
2856.	IQCE	IQ Motif Containing E
2857.	LARGE2	LARGE Xylosyl- And Glucuronyltransferase 2
2858.	LRRC8A	Leucine Rich Repeat Containing 8 VRAC Subunit A
2859.	MEPE	Matrix Extracellular Phosphoglycoprotein
2860.	METTL14	Methyltransferase 14, N6-Adenosine-Methyltransferase Subunit
2861.	MYCL	MYCL Proto-Oncogene, BHLH Transcription Factor

2862.	MYO3B	Myosin IIIB
2863.	MYO9A	Myosin IXA
2864.	NHS	NHS Actin Remodeling Regulator
2865.	OAS2	2'-5'-Oligoadenylate Synthetase 2
2866.	OTOA	Otoancorin
2867.	PIWIL4	Piwi Like RNA-Mediated Gene Silencing 4
2868.	PPIH	Peptidylprolyl Isomerase H
2869.	RIOK3	RIO Kinase 3
2870.	RNPC3	RNA Binding Region (RNP1, RRM) Containing 3
2871.	SCUBE1	Signal Peptide, CUB Domain And EGF Like Domain Containing 1
2872.	SLC16A8	Solute Carrier Family 16 Member 8
2873.	SLC2A13	Solute Carrier Family 2 Member 13
2874.	SLC30A4	Solute Carrier Family 30 Member 4
2875.	SLC30A6	Solute Carrier Family 30 Member 6
2876.	SLC45A3	Solute Carrier Family 45 Member 3
2877.	SLC6A15	Solute Carrier Family 6 Member 15
2878.	SLC6A7	Solute Carrier Family 6 Member 7
2879.	SNU13	Small Nuclear Ribonucleoprotein 13
2880.	SRGAP3	SLIT-ROBO Rho GTPase Activating Protein 3
2881.	SRY	Sex Determining Region Y
2882.	SSBP3	Single Stranded DNA Binding Protein 3
2883.	SUPT4H1	SPT4 Homolog, DSIF Elongation Factor Subunit
2884.	TBL2	Transducin Beta Like 2
2885.	UNC45A	Unc-45 Myosin Chaperone A
2886.	WTAP	WT1 Associated Protein
2887.	YTHDF2	YTH N6-Methyladenosine RNA Binding Protein F2
2888.	AAR2	AAR2 Splicing Factor
2889.	ABHD16A	Abhydrolase Domain Containing 16A, Phospholipase
2890.	CA13	Carbonic Anhydrase 13
2891.	CA7	Carbonic Anhydrase 7
2892.	CCL18	C-C Motif Chemokine Ligand 18
2893.	CLEC4E	C-Type Lectin Domain Family 4 Member E
2894.	COPS7A	COP9 Signalosome Subunit 7A
2895.	CPEB4	Cytoplasmic Polyadenylation Element Binding Protein 4
2896.	CSMD2	CUB And Sushi Multiple Domains 2
2897.	CSTF1	Cleavage Stimulation Factor Subunit 1
2898.	FGF22	Fibroblast Growth Factor 22
2899.	FHOD1	Formin Homology 2 Domain Containing 1
2900.	FRMD6	FERM Domain Containing 6
2901.	GJC3	Gap Junction Protein Gamma 3
2902.	GNB1L	G Protein Subunit Beta 1 Like

2903.	GPR182	G Protein-Coupled Receptor 182
2904.	GPS1	G Protein Pathway Suppressor 1
2905.	HMX1	H6 Family Homeobox 1
2906.	HSCB	HscB Mitochondrial Iron-Sulfur Cluster Cochaperone
2907.	IFNW1	Interferon Omega 1
2908.	KCNAB3	Potassium Voltage-Gated Channel Subfamily A Regulatory Beta Subunit 3
2909.	KRT74	Keratin 74
2910.	NAP1L1	Nucleosome Assembly Protein 1 Like 1
2911.	PIGW	Phosphatidylinositol Glycan Anchor Biosynthesis Class W
2912.	RBM15	RNA Binding Motif Protein 15
2913.	RHOF	Ras Homolog Family Member F, Filopodia Associated
2914.	RSPH4A	Radial Spoke Head Component 4A
2915.	RSPH9	Radial Spoke Head Component 9
2916.	SH3PXD2B	SH3 And PX Domains 2B
2917.	SLC30A7	Solute Carrier Family 30 Member 7
2918.	SLC36A1	Solute Carrier Family 36 Member 1
2919.	SNX14	Sorting Nexin 14
2920.	SRPX	Sushi Repeat Containing Protein X-Linked
2921.	TAF12	TATA-Box Binding Protein Associated Factor 12
2922.	TEX11	Testis Expressed 11
2923.	TRAPPC11	Trafficking Protein Particle Complex Subunit 11
2924.	YTHDC2	YTH N6-Methyladenosine RNA Binding Protein C2
2925.	ZDHHC19	Zinc Finger DHHC-Type Palmitoyltransferase 19
2926.	ZFPM1	Zinc Finger Protein, FOG Family Member 1
2927.	ABHD6	Abhydrolase Domain Containing 6, Acylglycerol Lipase
2928.	APEX2	Apurinic/Apyrimidinic Endodeoxyribonuclease 2
2929.	ATP6V1G1	ATPase H+ Transporting V1 Subunit G1
2930.	BOLL	Boule Homolog, RNA Binding Protein
2931.	CDC42BPG	CDC42 Binding Protein Kinase Gamma
2932.	CEP131	Centrosomal Protein 131
2933.	CGAS	Cyclic GMP-AMP Synthase
2934.	CHERP	Calcium Homeostasis Endoplasmic Reticulum Protein
2935.	DBR1	Debranching RNA Lariats 1
2936.	DEFB4A	Defensin Beta 4A
2937.	DGCR6	DiGeorge Syndrome Critical Region Gene 6
2938.	DNAAF3	Dynein Axonemal Assembly Factor 3
2939.	DYNLT3	Dynein Light Chain Tctex-Type 3
2940.	ELOF1	Elongation Factor 1
2941.	EXPH5	Exophilin 5
2942.	FBXW5	F-Box And WD Repeat Domain Containing 5
2943.	G3BP2	G3BP Stress Granule Assembly Factor 2

2944.	GATAD2A	GATA Zinc Finger Domain Containing 2A
2945.	GATC	Glutamyl-TRNA Amidotransferase Subunit C
2946.	IFITM2	Interferon Induced Transmembrane Protein 2
2947.	LPCAT1	Lysophosphatidylcholine Acyltransferase 1
2948.	MDFIC	MyoD Family Inhibitor Domain Containing
2949.	NDUFA3	NADH:Ubiquinone Oxidoreductase Subunit A3
2950.	OMP	Olfactory Marker Protein
2951.	PKD1L1	Polycystin 1 Like 1, Transient Receptor Potential Channel Interacting
2952.	POC5	POC5 Centriolar Protein
2953.	RBM7	RNA Binding Motif Protein 7
2954.	RRH	Retinal Pigment Epithelium-Derived Rhodopsin Homolog
2955.	SIT1	Signaling Threshold Regulating Transmembrane Adaptor 1
2956.	SLC16A10	Solute Carrier Family 16 Member 10
2957.	SLC39A9	Solute Carrier Family 39 Member 9
2958.	SLC6A18	Solute Carrier Family 6 Member 18
2959.	SPATA22	Spermatogenesis Associated 22
2960.	SRPK3	SRSF Protein Kinase 3
2961.	STRC	Stereocilin
2962.	UXT	Ubiquitously Expressed Prefoldin Like Chaperone
2963.	YTHDF1	YTH N6-Methyladenosine RNA Binding Protein F1
2964.	YTHDF3	YTH N6-Methyladenosine RNA Binding Protein F3
2965.	ZDHHC2	Zinc Finger DHHC-Type Palmitoyltransferase 2
2966.	ZNF526	Zinc Finger Protein 526
2967.	ZNRF3	Zinc And Ring Finger 3
2968.	ACP4	Acid Phosphatase 4
2969.	ATXN3L	Ataxin 3 Like
2970.	BPIFB1	BPI Fold Containing Family B Member 1
2971.	COPS2	COP9 Signalosome Subunit 2
2972.	DACT2	Dishevelled Binding Antagonist Of Beta Catenin 2
2973.	ESX1	ESX Homeobox 1
2974.	FAM135A	Family With Sequence Similarity 135 Member A
2975.	H3C12	H3 Clustered Histone 12
2976.	IFT46	Intraflagellar Transport 46
2977.	KIF24	Kinesin Family Member 24
2978.	MPP4	MAGUK P55 Scaffold Protein 4
2979.	OLAH	Oleoyl-ACP Hydrolase
2980.	OTUD4	OTU Deubiquitinase 4
2981.	PMCH	Pro-Melanin Concentrating Hormone
2982.	POLR2I	RNA Polymerase II Subunit I
2983.	PROM2	Prominin 2
2984.	PXDNL	Peroxidasin Like

2985.	RHBDD2	Rhomboid Domain Containing 2
2986.	RNF112	Ring Finger Protein 112
2987.	SLC39A12	Solute Carrier Family 39 Member 12
2988.	SLC6A16	Solute Carrier Family 6 Member 16
2989.	SLCO6A1	Solute Carrier Organic Anion Transporter Family Member 6A1
2990.	SMAP1	Small ArfGAP 1
2991.	TBCCD1	TBCC Domain Containing 1
2992.	TECPR2	Tectonin Beta-Propeller Repeat Containing 2
2993.	TFPT	TCF3 Fusion Partner
2994.	TTLL9	Tubulin Tyrosine Ligase Like 9
2995.	VEZT	Vezatin, Adherens Junctions Transmembrane Protein
2996.	ZNF142	Zinc Finger Protein 142
2997.	ZUP1	Zinc Finger Containing Ubiquitin Peptidase 1
2998.	C1QTNF2	C1q And TNF Related 2
2999.	DHX40	DEAH-Box Helicase 40
3000.	FEZF2	FEZ Family Zinc Finger 2
3001.	FRMPD1	FERM And PDZ Domain Containing 1
3002.	H3C13	H3 Clustered Histone 13
3003.	KLHL24	Kelch Like Family Member 24
3004.	TADA3	Transcriptional Adaptor 3
3005.	TBCC	Tubulin Folding Cofactor C
3006.	VIRMA	Vir Like M6A Methyltransferase Associated
3007.	ZNF407	Zinc Finger Protein 407
3008.	AMN1	Antagonist Of Mitotic Exit Network 1 Homolog
3009.	ANKRD46	Ankyrin Repeat Domain 46
3010.	ARHGEF37	Rho Guanine Nucleotide Exchange Factor 37
3011.	ARL4D	ADP Ribosylation Factor Like GTPase 4D
3012.	ASB11	Ankyrin Repeat And SOCS Box Containing 11
3013.	CCDC85C	Coiled-Coil Domain Containing 85C
3014.	CHRFAM7A	CHRNA7 (Exons 5-10) And FAM7A (Exons A-E) Fusion
3015.	CLIC6	Chloride Intracellular Channel 6
3016.	COL6A6	Collagen Type VI Alpha 6 Chain
3017.	CRYZL1	Crystallin Zeta Like 1
3018.	DTWD2	DTW Domain Containing 2
3019.	ENY2	ENY2 Transcription And Export Complex 2 Subunit
3020.	FBF1	Fas Binding Factor 1
3021.	FRY	FRY Microtubule Binding Protein
3022.	KLHL31	Kelch Like Family Member 31
3023.	LGSN	Lengsin, Lens Protein With Glutamine Synthetase Domain
3024.	LYNX1	Ly6/Neurotoxin 1
3025.	METTL16	Methyltransferase 16, RNA N6-Adenosine

3026.	NEXMIF	Neurite Extension And Migration Factor
3027.	NUDT10	Nudix Hydrolase 10
3028.	PCDHGA12	Protocadherin Gamma Subfamily A, 12
3029.	RNASEK	Ribonuclease K
3030.	SP6	Sp6 Transcription Factor
3031.	TADA2B	Transcriptional Adaptor 2B
3032.	TBC1D32	TBC1 Domain Family Member 32
3033.	TMEM240	Transmembrane Protein 240
3034.	VWA8	Von Willebrand Factor A Domain Containing 8
3035.	AFTPH	Aftiphilin
3036.	ATXN1L	Ataxin 1 Like
3037.	CLRN2	Clarin 2
3038.	CYB561D2	Cytochrome B561 Family Member D2
3039.	EFCAB7	EF-Hand Calcium Binding Domain 7
3040.	H3-5	H3.5 Histone
3041.	KCNRG	Potassium Channel Regulator
3042.	LHFPL5	LHFPL Tetraspan Subfamily Member 5
3043.	LSM8	LSM8 Homolog, U6 Small Nuclear RNA Associated
3044.	OGFRL1	Opioid Growth Factor Receptor Like 1
3045.	OR2W3	Olfactory Receptor Family 2 Subfamily W Member 3
3046.	RBM12B	RNA Binding Motif Protein 12B
3047.	SLC44A5	Solute Carrier Family 44 Member 5
3048.	TECTB	Tectorin Beta
3049.	TMEM218	Transmembrane Protein 218
3050.	XKR6	XK Related 6
3051.	ZP4	Zona Pellucida Glycoprotein 4
3052.	C1QTNF9	C1q And TNF Related 9
3053.	CFAP36	Cilia And Flagella Associated Protein 36
3054.	FAM120AOS	Family With Sequence Similarity 120 Member A Opposite Strand
3055.	FAM151A	Family With Sequence Similarity 151 Member A
3056.	NXNL2	Nucleoredoxin Like 2
3057.	OR10J1	Olfactory Receptor Family 10 Subfamily J Member 1
3058.	PDILT	Protein Disulfide Isomerase Like, Testis Expressed
3059.	SAMD7	Sterile Alpha Motif Domain Containing 7
3060.	SLC7A13	Solute Carrier Family 7 Member 13
3061.	TARM1	T Cell-Interacting, Activating Receptor On Myeloid Cells 1
3062.	PGBD2	PiggyBac Transposable Element Derived 2
3063.	PHF24	PHD Finger Protein 24
3064.	SPATA3	Spermatogenesis Associated 3
3065.	TDRP	Testis Development Related Protein
3066.	GARIN4	Golgi Associated RAB2 Interactor Family Member 4

3067.	HARBI1	Harbinger Transposase Derived 1
3068.	RNF113B	Ring Finger Protein 113B
3069.	ZNF568	Zinc Finger Protein 568
3070.	CRYBG3	Crystallin Beta-Gamma Domain Containing 3
3071.	DUX4	Double Homeobox 4
3072.	FAM151B	Family With Sequence Similarity 151 Member B
3073.	PLGLB2	Plasminogen Like B2
3074.	POTEF	POTE Ankyrin Domain Family Member F
3075.	RGPD1	RANBP2 Like And GRIP Domain Containing 1
3076.	ZNF682	Zinc Finger Protein 682
3077.	ZNF841	Zinc Finger Protein 841
3078.	OR2T35	Olfactory Receptor Family 2 Subfamily T Member 35
3079.	DEFB123	Defensin Beta 123
3080.	OR2T34	Olfactory Receptor Family 2 Subfamily T Member 34
3081.	THAP9	THAP Domain Containing 9
3082.	ZFTRAF1	Zinc Finger TRAF-Type Containing 1
3083.	PGBD4	PiggyBac Transposable Element Derived 4
3084.	ZNF736	Zinc Finger Protein 736
3085.	MBD3L2	Methyl-CpG Binding Domain Protein 3 Like 2
3086.	PPAN-P2RY11	PPAN-P2RY11 Readthrough
3087.	NUTM2D	NUT Family Member 2D
3088.	H3-7	H3.7 Histone (Putative)
3089.	PRAMEF15	PRAME Family Member 15
3090.	PRAMEF9	PRAME Family Member 9
3091.	ZC3H11C	Zinc Finger CCCH-Type Containing 11C
3092.	LOC102723407	Immunoglobulin Heavy Variable 4-38-2-Like
3093.	MAP2K1	Mitogen-Activated Protein Kinase Kinase 1
3094.	PTPN11	Protein Tyrosine Phosphatase Non-Receptor Type 11
3095.	CSNK2B	Casein Kinase 2 Beta
3096.	KCNMA1	Potassium Calcium-Activated Channel Subfamily M Alpha 1
3097.	PBX1	PBX Homeobox 1
3098.	DCC	DCC Netrin 1 Receptor
3099.	GABRB2	Gamma-Aminobutyric Acid Type A Receptor Subunit Beta2
3100.	KDM6A	Lysine Demethylase 6A
3101.	KMT2A	Lysine Methyltransferase 2A
3102.	KDM3B	Lysine Demethylase 3B
3103.	NR3C2	Nuclear Receptor Subfamily 3 Group C Member 2
3104.	SMARCB1	SWI/SNF Related, Matrix Associated, Actin Dependent Regulator Of Chromatin, Subfamily B, Member 1
3105.	FBXW7	F-Box And WD Repeat Domain Containing 7
3106.	KAT6A	Lysine Acetyltransferase 6A
3107.	KDM5B	Lysine Demethylase 5B

3108.	SPTBN1	Spectrin Beta, Non-Erythrocytic 1
3109.	ASXL1	ASXL Transcriptional Regulator 1
3110.	CHD2	Chromodomain Helicase DNA Binding Protein 2
3111.	DYNC1H1	Dynein Cytoplasmic 1 Heavy Chain 1
3112.	KAT6B	Lysine Acetyltransferase 6B
3113.	LPP	LIM Domain Containing Preferred Translocation Partner In Lipoma
3114.	PDE11A	Phosphodiesterase 11A
3115.	ARID1B	AT-Rich Interaction Domain 1B
3116.	CDK13	Cyclin Dependent Kinase 13
3117.	CIC	Capicua Transcriptional Repressor
3118.	EED	Embryonic Ectoderm Development
3119.	KMT2C	Lysine Methyltransferase 2C
3120.	MCCC2	Methylcrotonyl-CoA Carboxylase Subunit 2
3121.	NR2F1	Nuclear Receptor Subfamily 2 Group F Member 1
3122.	MYL9	Myosin Light Chain 9
3123.	RERE	Arginine-Glutamic Acid Dipeptide Repeats
3124.	SBF1	SET Binding Factor 1
3125.	SRD5A2	Steroid 5 Alpha-Reductase 2
3126.	ACTL6B	Actin Like 6B
3127.	CRLF1	Cytokine Receptor Like Factor 1
3128.	KMT5B	Lysine Methyltransferase 5B
3129.	MOCOS	Molybdenum Cofactor Sulfurase
3130.	NBEA	Neurobeachin
3131.	POGZ	Pogo Transposable Element Derived With ZNF Domain
3132.	PTPRM	Protein Tyrosine Phosphatase Receptor Type M
3133.	PTPRT	Protein Tyrosine Phosphatase Receptor Type T
3134.	WDFY3	WD Repeat And FYVE Domain Containing 3
3135.	AEBP1	AE Binding Protein 1
3136.	CLASP1	Cytoplasmic Linker Associated Protein 1
3137.	EBF3	EBF Transcription Factor 3
3138.	HEPACAM	Hepatic And Glial Cell Adhesion Molecule
3139.	MSRA	Methionine Sulfoxide Reductase A
3140.	RNF135	Ring Finger Protein 135
3141.	SPEN	Spen Family Transcriptional Repressor
3142.	UBE4A	Ubiquitination Factor E4A
3143.	IQSEC2	IQ Motif And Sec7 Domain ArfGEF 2
3144.	MED13L	Mediator Complex Subunit 13L
3145.	NPAS3	Neuronal PAS Domain Protein 3
3146.	PHIP	Pleckstrin Homology Domain Interacting Protein
3147.	PUS7	Pseudouridine Synthase 7
3148.	TNRC6B	Trinucleotide Repeat Containing Adaptor 6B

3149.	ASXL2	ASXL Transcriptional Regulator 2
3150.	DIP2A	Disco Interacting Protein 2 Homolog A
3151.	IRF2BPL	Interferon Regulatory Factor 2 Binding Protein Like
3152.	TCF20	Transcription Factor 20
3153.	TSEN54	TRNA Splicing Endonuclease Subunit 54
3154.	APOL2	Apolipoprotein L2
3155.	RNASEH2B	Ribonuclease H2 Subunit B
3156.	SETD5	SET Domain Containing 5
3157.	ULK4	Unc-51 Like Kinase 4
3158.	ABCA13	ATP Binding Cassette Subfamily A Member 13
3159.	RCL1	RNA Terminal Phosphate Cyclase Like 1
3160.	TANC2	Tetratricopeptide Repeat, Ankyrin Repeat And Coiled-Coil Containing 2
3161.	APOL4	Apolipoprotein L4
3162.	GIGYF1	GRB10 Interacting GYF Protein 1
3163.	ASXL3	ASXL Transcriptional Regulator 3
3164.	PARD3B	Par-3 Family Cell Polarity Regulator Beta
3165.	ZGRF1	Zinc Finger GRF-Type Containing 1
3166.	HECTD4	HECT Domain E3 Ubiquitin Protein Ligase 4
3167.	UBN2	Ubinuclein 2
3168.	FAM98C	Family With Sequence Similarity 98 Member C
3169.	IRAK1BP1	Interleukin 1 Receptor Associated Kinase 1 Binding Protein 1
3170.	CSTA	Cystatin A
3171.	INSR	Insulin Receptor
3172.	MMP2	Matrix Metallopeptidase 2
3173.	AGTR1	Angiotensin II Receptor Type 1
3174.	PLAU	Plasminogen Activator, Urokinase
3175.	APOA1	Apolipoprotein A1
3176.	ITGB2	Integrin Subunit Beta 2
3177.	MMP14	Matrix Metallopeptidase 14
3178.	MMP3	Matrix Metallopeptidase 3
3179.	PSMB8	Proteasome 20S Subunit Beta 8
3180.	SERPINE1	Serpin Family E Member 1
3181.	FGA	Fibrinogen Alpha Chain
3182.	GNAQ	G Protein Subunit Alpha Q
3183.	NR5A1	Nuclear Receptor Subfamily 5 Group A Member 1
3184.	PLG	Plasminogen
3185.	TNFSF11	TNF Superfamily Member 11
3186.	FOLR1	Folate Receptor Alpha
3187.	FST	Follistatin
3188.	ADAMTS13	ADAM Metallopeptidase With Thrombospondin Type 1 Motif 13
3189.	CD79A	CD79a Molecule

3190.	CTSF	Cathepsin F
3191.	ENG	Endoglin
3192.	F8	Coagulation Factor VIII
3193.	IRF7	Interferon Regulatory Factor 7
3194.	PDHA1	Pyruvate Dehydrogenase E1 Subunit Alpha 1
3195.	TGFB3	Transforming Growth Factor Beta 3
3196.	TNFRSF11B	TNF Receptor Superfamily Member 11b
3197.	ALAD	Aminolevulinate Dehydratase
3198.	EPHX2	Epoxide Hydrolase 2
3199.	FGR	FGR Proto-Oncogene, Src Family Tyrosine Kinase
3200.	GLDC	Glycine Decarboxylase
3201.	HEXB	Hexosaminidase Subunit Beta
3202.	ITPR3	Inositol 1,4,5-Trisphosphate Receptor Type 3
3203.	NAMPT	Nicotinamide Phosphoribosyltransferase
3204.	PSMB9	Proteasome 20S Subunit Beta 9
3205.	SH2B3	SH2B Adaptor Protein 3
3206.	YWHAQ	Tyrosine 3-Monooxygenase/Tryptophan 5-Monooxygenase Activation Protein Theta
3207.	ACVR2A	Activin A Receptor Type 2A
3208.	ANGPT1	Angiopoietin 1
3209.	BTD	Biotinidase
3210.	CCR4	C-C Motif Chemokine Receptor 4
3211.	GDF5	Growth Differentiation Factor 5
3212.	HSD11B2	Hydroxysteroid 11-Beta Dehydrogenase 2
3213.	IRF3	Interferon Regulatory Factor 3
3214.	ITGAX	Integrin Subunit Alpha X
3215.	NEFH	Neurofilament Heavy Chain
3216.	PDX1	Pancreatic And Duodenal Homeobox 1
3217.	POMT1	Protein O-Mannosyltransferase 1
3218.	PSMA3	Proteasome 20S Subunit Alpha 3
3219.	PSMB4	Proteasome 20S Subunit Beta 4
3220.	PTS	6-Pyruvoyltetrahydropterin Synthase
3221.	SERPINA6	Serpin Family A Member 6
3222.	ADAM12	ADAM Metallopeptidase Domain 12
3223.	ATP13A2	ATPase Cation Transporting 13A2
3224.	BDKRB2	Bradykinin Receptor B2
3225.	CXADR	CXADR Ig-Like Cell Adhesion Molecule
3226.	EXT2	Exostosin Glycosyltransferase 2
3227.	HBEGF	Heparin Binding EGF Like Growth Factor
3228.	HLA-G	Major Histocompatibility Complex, Class I, G
3229.	ITGA1	Integrin Subunit Alpha 1
3230.	ITPR2	Inositol 1,4,5-Trisphosphate Receptor Type 2

3231.	NPPA	Natriuretic Peptide A
3232.	OPTN	Optineurin
3233.	PKP2	Plakophilin 2
3234.	PRDX5	Peroxiredoxin 5
3235.	SELPLG	Selectin P Ligand
3236.	SLC25A13	Solute Carrier Family 25 Member 13
3237.	TREM2	Triggering Receptor Expressed On Myeloid Cells 2
3238.	AGTR2	Angiotensin II Receptor Type 2
3239.	DGUOK	Deoxyguanosine Kinase
3240.	DPM1	Dolichyl-Phosphate Mannosyltransferase Subunit 1, Catalytic
3241.	EYA1	EYA Transcriptional Coactivator And Phosphatase 1
3242.	GLRX	Glutaredoxin
3243.	IGFBP1	Insulin Like Growth Factor Binding Protein 1
3244.	IL7	Interleukin 7
3245.	INHA	Inhibin Subunit Alpha
3246.	P2RY2	Purinergic Receptor P2Y2
3247.	PHF21A	PHD Finger Protein 21A
3248.	POMT2	Protein O-Mannosyltransferase 2
3249.	RPS27A	Ribosomal Protein S27a
3250.	SOCS3	Suppressor Of Cytokine Signaling 3
3251.	TAC3	Tachykinin Precursor 3
3252.	VPS35	VPS35 Retromer Complex Component
3253.	ADAMTS10	ADAM Metallopeptidase With Thrombospondin Type 1 Motif 10
3254.	ADRB3	Adrenoceptor Beta 3
3255.	APCS	Amyloid P Component, Serum
3256.	DIS3L2	DIS3 Like 3'-5' Exoribonuclease 2
3257.	EPHA5	EPH Receptor A5
3258.	ERLIN1	ER Lipid Raft Associated 1
3259.	MINK1	Misshapen Like Kinase 1
3260.	NPHS2	NPHS2 Stomatin Family Member, Podocin
3261.	OLR1	Oxidized Low Density Lipoprotein Receptor 1
3262.	PAPPA	Pappalysin 1
3263.	PAX1	Paired Box 1
3264.	PLD3	Phospholipase D Family Member 3
3265.	PTGDR2	Prostaglandin D2 Receptor 2
3266.	PTGFR	Prostaglandin F Receptor
3267.	PTX3	Pentraxin 3
3268.	SHBG	Sex Hormone Binding Globulin
3269.	TAPBP	TAP Binding Protein
3270.	UBQLN2	Ubiquilin 2
3271.	UROS	Uroporphyrinogen III Synthase

3272.	VPS4A	Vacuolar Protein Sorting 4 Homolog A
3273.	FKTN	Fukutin
3274.	IL1RL1	Interleukin 1 Receptor Like 1
3275.	INHBB	Inhibin Subunit Beta B
3276.	LMO2	LIM Domain Only 2
3277.	MPDU1	Mannose-P-Dolichol Utilization Defect 1
3278.	PECAM1	Platelet And Endothelial Cell Adhesion Molecule 1
3279.	BGLAP	Bone Gamma-Carboxyglutamate Protein
3280.	CCN1	Cellular Communication Network Factor 1
3281.	DDAH2	DDAH Family Member 2, ADMA-Independent
3282.	DPM3	Dolichyl-Phosphate Mannosyltransferase Subunit 3, Regulatory
3283.	FCAR	Fc Alpha Receptor
3284.	GATB	Glutamyl-TRNA Amidotransferase Subunit B
3285.	MEIS2	Meis Homeobox 2
3286.	PRG2	Proteoglycan 2, Pro Eosinophil Major Basic Protein
3287.	SPN	Sialophorin
3288.	ALX4	ALX Homeobox 4
3289.	ANGPT4	Angiopoietin 4
3290.	ANKH	ANKH Inorganic Pyrophosphate Transport Regulator
3291.	ATP1A4	ATPase Na+/K+ Transporting Subunit Alpha 4
3292.	DMRT1	Doublesex And Mab-3 Related Transcription Factor 1
3293.	FKRP	Fukutin Related Protein
3294.	H1-2	H1.2 Linker Histone, Cluster Member
3295.	LGR4	Leucine Rich Repeat Containing G Protein-Coupled Receptor 4
3296.	PAEP	Progestagen Associated Endometrial Protein
3297.	PAPPA2	Pappalysin 2
3298.	PHF1	PHD Finger Protein 1
3299.	RCN1	Reticulocalbin 1
3300.	TMCO1	Transmembrane And Coiled-Coil Domains 1
3301.	TMEM106B	Transmembrane Protein 106B
3302.	UTS2	Urotensin 2
3303.	ACBD3	Acyl-CoA Binding Domain Containing 3
3304.	ADAMTS17	ADAM Metallopeptidase With Thrombospondin Type 1 Motif 17
3305.	CD200	CD200 Molecule
3306.	DOLK	Dolichol Kinase
3307.	EVL	Enah/Vasp-Like
3308.	GDF3	Growth Differentiation Factor 3
3309.	GLS2	Glutaminase 2
3310.	ICAM5	Intercellular Adhesion Molecule 5
3311.	KCTD1	Potassium Channel Tetramerization Domain Containing 1
3312.	KRT15	Keratin 15

3313.	LIN7C	Lin-7 Homolog C, Crumbs Cell Polarity Complex Component
3314.	SENP8	SUMO Peptidase Family Member, NEDD8 Specific
3315.	SOCS5	Suppressor Of Cytokine Signaling 5
3316.	STX6	Syntaxin 6
3317.	WDR36	WD Repeat Domain 36
3318.	AMER1	APC Membrane Recruitment Protein 1
3319.	APBB1IP	Amyloid Beta Precursor Protein Binding Family B Member 1 Interacting Protein
3320.	MUC16	Mucin 16, Cell Surface Associated
3321.	PSG1	Pregnancy Specific Beta-1-Glycoprotein 1
3322.	PSMG2	Proteasome Assembly Chaperone 2
3323.	RNF170	Ring Finger Protein 170
3324.	SDF4	Stromal Cell Derived Factor 4
3325.	TRAM1	Translocation Associated Membrane Protein 1
3326.	WDR81	WD Repeat Domain 81
3327.	KIR2DL4	Killer Cell Immunoglobulin Like Receptor, Two Ig Domains And Long Cytoplasmic Tail 4
3328.	KLHL41	Kelch Like Family Member 41
3329.	LECT2	Leukocyte Cell Derived Chemotaxin 2
3330.	LGALS13	Galectin 13
3331.	NEK4	NIMA Related Kinase 4
3332.	NOSTRIN	Nitric Oxide Synthase Trafficking
3333.	PIGF	Phosphatidylinositol Glycan Anchor Biosynthesis Class F
3334.	RXYLT1	Ribitol Xylosyltransferase 1
3335.	SLC25A18	Solute Carrier Family 25 Member 18
3336.	STARD7	StAR Related Lipid Transfer Domain Containing 7
3337.	VAX1	Ventral Anterior Homeobox 1
3338.	BZW1	Basic Leucine Zipper And W2 Domains 1
3339.	CBX8	Chromobox 8
3340.	DPM2	Dolichyl-Phosphate Mannosyltransferase Subunit 2, Regulatory
3341.	EMILIN2	Elastin Microfibril Interfacer 2
3342.	EPDR1	Ependymin Related 1
3343.	SPOCK3	SPARC (Osteonectin), Cwcv And Kazal Like Domains Proteoglycan 3
3344.	CHMP6	Charged Multivesicular Body Protein 6
3345.	COL20A1	Collagen Type XX Alpha 1 Chain
3346.	DMRT2	Doublesex And Mab-3 Related Transcription Factor 2
3347.	EBNA1BP2	EBNA1 Binding Protein 2
3348.	FHL5	Four And A Half LIM Domains 5
3349.	GET4	Guided Entry Of Tail-Anchored Proteins Factor 4
3350.	NDFIP2	Nedd4 Family Interacting Protein 2
3351.	NOP53	NOP53 Ribosome Biogenesis Factor
3352.	NXT1	Nuclear Transport Factor 2 Like Export Factor 1
3353.	PGAP2	Post-GPI Attachment To Proteins 2

3354.	PRDM8	PR/SET Domain 8
3355.	RGS22	Regulator Of G Protein Signaling 22
3356.	SLC16A12	Solute Carrier Family 16 Member 12
3357.	VPS50	VPS50 Subunit Of EARP/GARPII Complex
3358.	CGB3	Chorionic Gonadotropin Subunit Beta 3
3359.	KAZN	Kazrin, Periplakin Interacting Protein
3360.	MAGEE1	MAGE Family Member E1
3361.	MCF2L2	MCF.2 Cell Line Derived Transforming Sequence-Like 2
3362.	MRAP2	Melanocortin 2 Receptor Accessory Protein 2
3363.	NTPCR	Nucleoside-Triphosphatase, Cancer-Related
3364.	PLAC1	Placenta Enriched 1
3365.	SH3D19	SH3 Domain Containing 19
3366.	TMEM184B	Transmembrane Protein 184B
3367.	UACA	Uveal Autoantigen With Coiled-Coil Domains And Ankyrin Repeats
3368.	ZC3H12D	Zinc Finger CCCH-Type Containing 12D
3369.	ASTN1	Astrotactin 1
3370.	CCDC34	Coiled-Coil Domain Containing 34
3371.	JPT1	Jupiter Microtubule Associated Homolog 1
3372.	MPPED1	Metallophosphoesterase Domain Containing 1
3373.	TRAM2	Translocation Associated Membrane Protein 2
3374.	ZNF274	Zinc Finger Protein 274
3375.	KIR2DS4	Killer Cell Immunoglobulin Like Receptor, Two Ig Domains And Short Cytoplasmic Tail 4
3376.	OARD1	O-Acyl-ADP-Ribose Deacylase 1
3377.	BROX	BRO1 Domain And CAAX Motif Containing
3378.	CGB7	Chorionic Gonadotropin Subunit Beta 7
3379.	LCA5L	Lebercilin LCA5 Like
3380.	LRRC37A	Leucine Rich Repeat Containing 37A
3381.	LRRC37A3	Leucine Rich Repeat Containing 37 Member A3
3382.	OAZ3	Ornithine Decarboxylase Antizyme 3
3383.	OR2A25	Olfactory Receptor Family 2 Subfamily A Member 25
3384.	PRRG1	Proline Rich And Gla Domain 1
3385.	PRRG2	Proline Rich And Gla Domain 2
3386.	ACSM6	Acyl-CoA Synthetase Medium Chain Family Member 6
3387.	TMEM187	Transmembrane Protein 187
3388.	INTS15	Integrator Complex Subunit 15
3389.	TLCD5	TLC Domain Containing 5
3390.	MAB21L3	Mab-21 Like 3
3391.	RBMXL1	RBMX Like 1
3392.	SAMD1	Sterile Alpha Motif Domain Containing 1
3393.	PNMA6A	PNMA Family Member 6A
3394.	STPG4	Sperm-Tail PG-Rich Repeat Containing 4

3395.	PMP22	Peripheral Myelin Protein 22
3396.	MET	MET Proto-Oncogene, Receptor Tyrosine Kinase
3397.	NTRK3	Neurotrophic Receptor Tyrosine Kinase 3
3398.	SMAD4	SMAD Family Member 4
3399.	ABCB1	ATP Binding Cassette Subfamily B Member 1
3400.	DNMT3B	DNA Methyltransferase 3 Beta
3401.	PPP3CA	Protein Phosphatase 3 Catalytic Subunit Alpha
3402.	EIF4E	Eukaryotic Translation Initiation Factor 4E
3403.	GLUD1	Glutamate Dehydrogenase 1
3404.	GRIA3	Glutamate Ionotropic Receptor AMPA Type Subunit 3
3405.	GSTP1	Glutathione S-Transferase Pi 1
3406.	IL6R	Interleukin 6 Receptor
3407.	KCNH2	Potassium Voltage-Gated Channel Subfamily H Member 2
3408.	PLA2G7	Phospholipase A2 Group VII
3409.	UGT1A1	UDP Glucuronosyltransferase Family 1 Member A1
3410.	ACACA	Acetyl-CoA Carboxylase Alpha
3411.	ALDOA	Aldolase, Fructose-Bisphosphate A
3412.	APC	APC Regulator Of WNT Signaling Pathway
3413.	CAMK2B	Calcium/Calmodulin Dependent Protein Kinase II Beta
3414.	CASK	Calcium/Calmodulin Dependent Serine Protein Kinase
3415.	CHRNA4	Cholinergic Receptor Nicotinic Alpha 4 Subunit
3416.	FASN	Fatty Acid Synthase
3417.	GLS	Glutaminase
3418.	GSK3A	Glycogen Synthase Kinase 3 Alpha
3419.	IRAK1	Interleukin 1 Receptor Associated Kinase 1
3420.	LAMB1	Laminin Subunit Beta 1
3421.	RORA	RAR Related Orphan Receptor A
3422.	ATP2A1	ATPase Sarcoplasmic/Endoplasmic Reticulum Ca2+ Transporting 1
3423.	NCAM1	Neural Cell Adhesion Molecule 1
3424.	SDHA	Succinate Dehydrogenase Complex Flavoprotein Subunit A
3425.	SPTAN1	Spectrin Alpha, Non-Erythrocytic 1
3426.	VLDLR	Very Low Density Lipoprotein Receptor
3427.	ABCC1	ATP Binding Cassette Subfamily C Member 1 (ABCC1 Blood Group)
3428.	ALDH5A1	Aldehyde Dehydrogenase 5 Family Member A1
3429.	DHCR7	7-Dehydrocholesterol Reductase
3430.	FOLH1	Folate Hydrolase 1
3431.	FUCA1	Alpha-L-Fucosidase 1
3432.	FYN	FYN Proto-Oncogene, Src Family Tyrosine Kinase
3433.	GABRA1	Gamma-Aminobutyric Acid Type A Receptor Subunit Alpha1
3434.	GABRA2	Gamma-Aminobutyric Acid Type A Receptor Subunit Alpha2
3435.	GALNS	Galactosamine (N-Acetyl)-6-Sulfatase

3436.	HMGCR	3-Hydroxy-3-Methylglutaryl-CoA Reductase
3437.	IRS1	Insulin Receptor Substrate 1
3438.	MAPK7	Mitogen-Activated Protein Kinase 7
3439.	NQO1	NAD(P)H Quinone Dehydrogenase 1
3440.	NRP1	Neuropilin 1
3441.	PAK2	P21 (RAC1) Activated Kinase 2
3442.	PI4KA	Phosphatidylinositol 4-Kinase Alpha
3443.	PLA2G4A	Phospholipase A2 Group IVA
3444.	PLCB1	Phospholipase C Beta 1
3445.	TTN	Titin
3446.	VCL	Vinculin
3447.	ABAT	4-Aminobutyrate Aminotransferase
3448.	ADORA2A	Adenosine A2a Receptor
3449.	ADRA1A	Adrenoceptor Alpha 1A
3450.	CACNA2D1	Calcium Voltage-Gated Channel Auxiliary Subunit Alpha2delta 1
3451.	CACNB2	Calcium Voltage-Gated Channel Auxiliary Subunit Beta 2
3452.	CDC45	Cell Division Cycle 45
3453.	CIT	Citron Rho-Interacting Serine/Threonine Kinase
3454.	COL4A2	Collagen Type IV Alpha 2 Chain
3455.	CRKL	CRK Like Proto-Oncogene, Adaptor Protein
3456.	CYP27A1	Cytochrome P450 Family 27 Subfamily A Member 1
3457.	FECH	Ferrochelatase
3458.	GABRB1	Gamma-Aminobutyric Acid Type A Receptor Subunit Beta1
3459.	GPD2	Glycerol-3-Phosphate Dehydrogenase 2
3460.	HSPA1A	Heat Shock Protein Family A (Hsp70) Member 1A
3461.	HYAL1	Hyaluronidase 1
3462.	KCNA2	Potassium Voltage-Gated Channel Subfamily A Member 2
3463.	MAD1L1	Mitotic Arrest Deficient 1 Like 1
3464.	MAP4K4	Mitogen-Activated Protein Kinase Kinase Kinase Kinase 4
3465.	MMP19	Matrix Metallopeptidase 19
3466.	NR4A1	Nuclear Receptor Subfamily 4 Group A Member 1
3467.	PAFAH1B1	Platelet Activating Factor Acetylhydrolase 1b Regulatory Subunit 1
3468.	PLA2G2A	Phospholipase A2 Group IIA
3469.	PRKAR1B	Protein Kinase CAMP-Dependent Type I Regulatory Subunit Beta
3470.	PSAT1	Phosphoserine Aminotransferase 1
3471.	RALA	RAS Like Proto-Oncogene A
3472.	RXRB	Retinoid X Receptor Beta
3473.	RYR2	Ryanodine Receptor 2
3474.	SLC25A12	Solute Carrier Family 25 Member 12
3475.	SLC6A8	Solute Carrier Family 6 Member 8
3476.	SP1	Sp1 Transcription Factor

3477.	TOR1A	Torsin Family 1 Member A
3478.	AGL	Amylo-Alpha-1, 6-Glucosidase, 4-Alpha-Glucanotransferase
3479.	AMPD2	Adenosine Monophosphate Deaminase 2
3480.	BCL6	BCL6 Transcription Repressor
3481.	CACNA1I	Calcium Voltage-Gated Channel Subunit Alpha1 I
3482.	CCKAR	Cholecystokinin A Receptor
3483.	CHRM1	Cholinergic Receptor Muscarinic 1
3484.	CHRNA3	Cholinergic Receptor Nicotinic Alpha 3 Subunit
3485.	CHRNA5	Cholinergic Receptor Nicotinic Alpha 5 Subunit
3486.	CHRNB2	Cholinergic Receptor Nicotinic Beta 2 Subunit
3487.	CNTN2	Contactin 2
3488.	CUX1	Cut Like Homeobox 1
3489.	DPYSL2	Dihydropyrimidinase Like 2
3490.	EEF2K	Eukaryotic Elongation Factor 2 Kinase
3491.	FLNC	Filamin C
3492.	GABRD	Gamma-Aminobutyric Acid Type A Receptor Subunit Delta
3493.	GNAI1	G Protein Subunit Alpha I1
3494.	GNAI3	G Protein Subunit Alpha I3
3495.	GRM4	Glutamate Metabotropic Receptor 4
3496.	GSTM3	Glutathione S-Transferase Mu 3
3497.	KCNC1	Potassium Voltage-Gated Channel Subfamily C Member 1
3498.	KDM6B	Lysine Demethylase 6B
3499.	MYH10	Myosin Heavy Chain 10
3500.	NQO2	N-Ribosyldihydronicotinamide:Quinone Dehydrogenase 2
3501.	NT5C2	5'-Nucleotidase, Cytosolic II
3502.	PPP2R5D	Protein Phosphatase 2 Regulatory Subunit B'Delta
3503.	PRKAB2	Protein Kinase AMP-Activated Non-Catalytic Subunit Beta 2
3504.	PTGDS	Prostaglandin D2 Synthase
3505.	PTPRD	Protein Tyrosine Phosphatase Receptor Type D
3506.	SLC25A1	Solute Carrier Family 25 Member 1
3507.	SLC9A6	Solute Carrier Family 9 Member A6
3508.	SOX10	SRY-Box Transcription Factor 10
3509.	UROD	Uroporphyrinogen Decarboxylase
3510.	YWHAH	Tyrosine 3-Monooxygenase/Tryptophan 5-Monooxygenase Activation Protein Eta
3511.	APOL1	Apolipoprotein L1
3512.	CACNG2	Calcium Voltage-Gated Channel Auxiliary Subunit Gamma 2
3513.	CADM1	Cell Adhesion Molecule 1
3514.	CCKBR	Cholecystokinin B Receptor
3515.	CNP	2',3'-Cyclic Nucleotide 3' Phosphodiesterase
3516.	CORO1A	Coronin 1A
3517.	DBI	Diazepam Binding Inhibitor, Acyl-CoA Binding Protein

3518.	DLL1	Delta Like Canonical Notch Ligand 1
3519.	EGR2	Early Growth Response 2
3520.	FZD3	Frizzled Class Receptor 3
3521.	GBE1	1,4-Alpha-Glucan Branching Enzyme 1
3522.	GLRA2	Glycine Receptor Alpha 2
3523.	GRIK5	Glutamate Ionotropic Receptor Kainate Type Subunit 5
3524.	GRM8	Glutamate Metabotropic Receptor 8
3525.	GSTO1	Glutathione S-Transferase Omega 1
3526.	HAAO	3-Hydroxyanthranilate 3,4-Dioxygenase
3527.	HES1	Hes Family BHLH Transcription Factor 1
3528.	HINT1	Histidine Triad Nucleotide Binding Protein 1
3529.	HMBS	Hydroxymethylbilane Synthase
3530.	KIF22	Kinesin Family Member 22
3531.	KMO	Kynurenine 3-Monooxygenase
3532.	MADD	MAP Kinase Activating Death Domain
3533.	MAP2	Microtubule Associated Protein 2
3534.	MED12	Mediator Complex Subunit 12
3535.	NRCAM	Neuronal Cell Adhesion Molecule
3536.	NSF	N-Ethylmaleimide Sensitive Factor, Vesicle Fusing ATPase
3537.	PABPN1	Poly(A) Binding Protein Nuclear 1
3538.	PDE4B	Phosphodiesterase 4B
3539.	PLOD3	Procollagen-Lysine,2-Oxoglutarate 5-Dioxygenase 3
3540.	PPARA	Peroxisome Proliferator Activated Receptor Alpha
3541.	RARS1	Arginyl-TRNA Synthetase 1
3542.	RTN4	Reticulon 4
3543.	TDO2	Tryptophan 2,3-Dioxygenase
3544.	TRIO	Trio Rho Guanine Nucleotide Exchange Factor
3545.	TRRAP	Transformation/Transcription Domain Associated Protein
3546.	VAMP2	Vesicle Associated Membrane Protein 2
3547.	ACY1	Aminoacylase 1
3548.	AVPR1B	Arginine Vasopressin Receptor 1B
3549.	B3GAT1	Beta-1,3-Glucuronyltransferase 1
3550.	BDH1	3-Hydroxybutyrate Dehydrogenase 1
3551.	BSCL2	BSCL2 Lipid Droplet Biogenesis Associated, Seipin
3552.	BST1	Bone Marrow Stromal Cell Antigen 1
3553.	COX10	Cytochrome C Oxidase Assembly Factor Heme A:Farnesyltransferase COX10
3554.	CYP2R1	Cytochrome P450 Family 2 Subfamily R Member 1
3555.	GAP43	Growth Associated Protein 43
3556.	GNS	Glucosamine (N-Acetyl)-6-Sulfatase
3557.	GP1BB	Glycoprotein Ib Platelet Subunit Beta
3558.	GRK3	G Protein-Coupled Receptor Kinase 3

3559.	HNF1B	HNF1 Homeobox B
3560.	HRH2	Histamine Receptor H2
3561.	IL1RAPL1	Interleukin 1 Receptor Accessory Protein Like 1
3562.	KCNC4	Potassium Voltage-Gated Channel Subfamily C Member 4
3563.	KCND2	Potassium Voltage-Gated Channel Subfamily D Member 2
3564.	KIF1A	Kinesin Family Member 1A
3565.	LRP8	LDL Receptor Related Protein 8
3566.	LZTR1	Leucine Zipper Like Post Translational Regulator 1
3567.	MAFB	MAF BZIP Transcription Factor B
3568.	MANBA	Mannosidase Beta
3569.	NDUFA12	NADH:Ubiquinone Oxidoreductase Subunit A12
3570.	NDUFV2	NADH:Ubiquinone Oxidoreductase Core Subunit V2
3571.	PER2	Period Circadian Regulator 2
3572.	PIP4K2A	Phosphatidylinositol-5-Phosphate 4-Kinase Type 2 Alpha
3573.	PPP3CB	Protein Phosphatase 3 Catalytic Subunit Beta
3574.	PTGES3	Prostaglandin E Synthase 3
3575.	PTK7	Protein Tyrosine Kinase 7 (Inactive)
3576.	RPS24	Ribosomal Protein S24
3577.	SERPINA3	Serpin Family A Member 3
3578.	SOD3	Superoxide Dismutase 3
3579.	SOX3	SRY-Box Transcription Factor 3
3580.	TBX1	T-Box Transcription Factor 1
3581.	TCN2	Transcobalamin 2
3582.	TNIK	TRAF2 And NCK Interacting Kinase
3583.	VAMP1	Vesicle Associated Membrane Protein 1
3584.	ADRA2B	Adrenoceptor Alpha 2B
3585.	AKAP9	A-Kinase Anchoring Protein 9
3586.	APBA2	Amyloid Beta Precursor Protein Binding Family A Member 2
3587.	APOD	Apolipoprotein D
3588.	ATN1	Atrophin 1
3589.	BACE2	Beta-Secretase 2
3590.	C4B	Complement C4B (Chido/Rodgers Blood Group)
3591.	CACNB1	Calcium Voltage-Gated Channel Auxiliary Subunit Beta 1
3592.	CD5	CD5 Molecule
3593.	CDIPT	CDP-Diacylglycerol--Inositol 3-Phosphatidyltransferase
3594.	CHIA	Chitinase Acidic
3595.	CHL1	Cell Adhesion Molecule L1 Like
3596.	CHRM4	Cholinergic Receptor Muscarinic 4
3597.	CRHR2	Corticotropin Releasing Hormone Receptor 2
3598.	DAGLA	Diacylglycerol Lipase Alpha
3599.	DOCK3	Dedicator Of Cytokinesis 3

3600.	FERMT1	FERM Domain Containing Kindlin 1
3601.	FGF12	Fibroblast Growth Factor 12
3602.	FLG	Filaggrin
3603.	FLII	FLII Actin Remodeling Protein
3604.	GNAL	G Protein Subunit Alpha L
3605.	GNE	Glucosamine (UDP-N-Acetyl)-2-Epimerase/N-Acetylmannosamine Kinase
3606.	GRIP1	Glutamate Receptor Interacting Protein 1
3607.	H1-4	H1.4 Linker Histone, Cluster Member
3608.	HIRA	Histone Cell Cycle Regulator
3609.	JARID2	Jumonji And AT-Rich Interaction Domain Containing 2
3610.	KCNN3	Potassium Calcium-Activated Channel Subfamily N Member 3
3611.	KDM4C	Lysine Demethylase 4C
3612.	KDM5A	Lysine Demethylase 5A
3613.	KIF5C	Kinesin Family Member 5C
3614.	M6PR	Mannose-6-Phosphate Receptor, Cation Dependent
3615.	MLC1	Modulator Of VRAC Current 1
3616.	NRG3	Neuregulin 3
3617.	ORC4	Origin Recognition Complex Subunit 4
3618.	PPP3CC	Protein Phosphatase 3 Catalytic Subunit Gamma
3619.	PREP	Prolyl Endopeptidase
3620.	SETBP1	SET Binding Protein 1
3621.	SLC18A1	Solute Carrier Family 18 Member A1
3622.	SLC22A3	Solute Carrier Family 22 Member 3
3623.	TRIP12	Thyroid Hormone Receptor Interactor 12
3624.	XRN2	5'-3' Exoribonuclease 2
3625.	AATF	Apoptosis Antagonizing Transcription Factor
3626.	ADGRL1	Adhesion G Protein-Coupled Receptor L1
3627.	AGO1	Argonaute RISC Component 1
3628.	AIM2	Absent In Melanoma 2
3629.	AP1S2	Adaptor Related Protein Complex 1 Subunit Sigma 2
3630.	ATOX1	Antioxidant 1 Copper Chaperone
3631.	BRD1	Bromodomain Containing 1
3632.	CC2D1A	Coiled-Coil And C2 Domain Containing 1A
3633.	CELSR2	Cadherin EGF LAG Seven-Pass G-Type Receptor 2
3634.	CERT1	Ceramide Transporter 1
3635.	CHMP1A	Charged Multivesicular Body Protein 1A
3636.	CHN2	Chimerin 2
3637.	CHRM5	Cholinergic Receptor Muscarinic 5
3638.	CPLX1	Complexin 1
3639.	CRHBP	Corticotropin Releasing Hormone Binding Protein
3640.	CUX2	Cut Like Homeobox 2

3641.	DLG3	Discs Large MAGUK Scaffold Protein 3
3642.	DOCK4	Dedicator Of Cytokinesis 4
3643.	DSCAM	DS Cell Adhesion Molecule
3644.	EIF4B	Eukaryotic Translation Initiation Factor 4B
3645.	EN2	Engrailed Homeobox 2
3646.	ERC1	ELKS/RAB6-Interacting/CAST Family Member 1
3647.	FABP7	Fatty Acid Binding Protein 7
3648.	FMO5	Flavin Containing Dimethylaniline Monoxygenase 5
3649.	FOXL2	Forkhead Box L2
3650.	GCLM	Glutamate-Cysteine Ligase Modifier Subunit
3651.	GPSM2	G Protein Signaling Modulator 2
3652.	GRID1	Glutamate Ionotropic Receptor Delta Type Subunit 1
3653.	GRIK4	Glutamate Ionotropic Receptor Kainate Type Subunit 4
3654.	GRIN3B	Glutamate Ionotropic Receptor NMDA Type Subunit 3B
3655.	GSTM2	Glutathione S-Transferase Mu 2
3656.	HECW2	HECT, C2 And WW Domain Containing E3 Ubiquitin Protein Ligase 2
3657.	HOXA1	Homeobox A1
3658.	HSF4	Heat Shock Transcription Factor 4
3659.	IMPA2	Inositol Monophosphatase 2
3660.	JMJD1C	Jumonji Domain Containing 1C
3661.	KMT2E	Lysine Methyltransferase 2E (Inactive)
3662.	LMAN2L	Lectin, Mannose Binding 2 Like
3663.	MEGF10	Multiple EGF Like Domains 10
3664.	MVP	Major Vault Protein
3665.	NAA15	N-Alpha-Acetyltransferase 15, NatA Auxiliary Subunit
3666.	NCS1	Neuronal Calcium Sensor 1
3667.	NTSR1	Neurotensin Receptor 1
3668.	PICK1	Protein Interacting With PRKCA 1
3669.	QKI	QKI, KH Domain Containing RNA Binding
3670.	RPS23	Ribosomal Protein S23
3671.	SEMA5A	Semaphorin 5A
3672.	SLC35A3	Solute Carrier Family 35 Member A3
3673.	SRR	Serine Racemase
3674.	ST8SIA2	ST8 Alpha-N-Acetyl-Neuraminide Alpha-2,8-Sialyltransferase 2
3675.	TAOK2	TAO Kinase 2
3676.	WDR26	WD Repeat Domain 26
3677.	C4BPA	Complement Component 4 Binding Protein Alpha
3678.	CEBPD	CCAAT Enhancer Binding Protein Delta
3679.	CHD1L	Chromodomain Helicase DNA Binding Protein 1 Like
3680.	DRG2	Developmentally Regulated GTP Binding Protein 2
3681.	GSTM5	Glutathione S-Transferase Mu 5

3682.	HOXA13	Homeobox A13
3683.	ITSN1	Intersectin 1
3684.	KALRN	Kalirin RhoGEF Kinase
3685.	LHX1	LIM Homeobox 1
3686.	LMO1	LIM Domain Only 1
3687.	MOV10	Mov10 RNA Helicase
3688.	NMBR	Neuromedin B Receptor
3689.	PCDH10	Protocadherin 10
3690.	PPP1R9B	Protein Phosphatase 1 Regulatory Subunit 9B
3691.	QPRT	Quinolinate Phosphoribosyltransferase
3692.	SLC25A22	Solute Carrier Family 25 Member 22
3693.	SRCAP	Snf2 Related CREBBP Activator Protein
3694.	VSNL1	Visinin Like 1
3695.	AGAP1	ArfGAP With GTPase Domain, Ankyrin Repeat And PH Domain 1
3696.	AMY2B	Amylase Alpha 2B
3697.	ARF3	ADP Ribosylation Factor 3
3698.	ASH1L	ASH1 Like Histone Lysine Methyltransferase
3699.	ATXN2L	Ataxin 2 Like
3700.	BRWD1	Bromodomain And WD Repeat Domain Containing 1
3701.	CDH10	Cadherin 10
3702.	CDH8	Cadherin 8
3703.	CLTCL1	Clathrin Heavy Chain Like 1
3704.	CNKSR2	Connector Enhancer Of Kinase Suppressor Of Ras 2
3705.	DGKB	Diacylglycerol Kinase Beta
3706.	EGR3	Early Growth Response 3
3707.	ETF1	Eukaryotic Translation Termination Factor 1
3708.	FXR2	FMR1 Autosomal Homolog 2
3709.	KCNC2	Potassium Voltage-Gated Channel Subfamily C Member 2
3710.	LIPH	Lipase H
3711.	LLGL1	LLGL Scribble Cell Polarity Complex Component 1
3712.	MED13	Mediator Complex Subunit 13
3713.	NCAN	Neurocan
3714.	NCKAP1	NCK Associated Protein 1
3715.	NIPA1	NIPA Magnesium Transporter 1
3716.	PDE4DIP	Phosphodiesterase 4D Interacting Protein
3717.	PPIP5K2	Diphosphoinositol Pentakisphosphate Kinase 2
3718.	PPY	Pancreatic Polypeptide
3719.	RBBP5	RB Binding Protein 5, Histone Lysine Methyltransferase Complex Subunit
3720.	RGS10	Regulator Of G Protein Signaling 10
3721.	RGS2	Regulator Of G Protein Signaling 2
3722.	RIT2	Ras Like Without CAAX 2

3723.	RPH3A	Rabphilin 3A
3724.	RPL31	Ribosomal Protein L31
3725.	SLC9A9	Solute Carrier Family 9 Member A9
3726.	SLITRK2	SLIT And NTRK Like Family Member 2
3727.	SORBS2	Sorbin And SH3 Domain Containing 2
3728.	ST8SIA4	ST8 Alpha-N-Acetyl-Neuraminide Alpha-2,8-Sialyltransferase 4
3729.	SULT4A1	Sulfotransferase Family 4A Member 1
3730.	SYNE2	Spectrin Repeat Containing Nuclear Envelope Protein 2
3731.	SYNGR1	Synaptogyrin 1
3732.	SYT11	Synaptotagmin 11
3733.	ZNF462	Zinc Finger Protein 462
3734.	ACP6	Acid Phosphatase 6, Lysophosphatidic
3735.	ACTR3B	Actin Related Protein 3B
3736.	AIFM3	Apoptosis Inducing Factor Mitochondria Associated 3
3737.	AMY2A	Amylase Alpha 2A
3738.	ANKRD11	Ankyrin Repeat Domain Containing 11
3739.	AS3MT	Arsenite Methyltransferase
3740.	CACNG5	Calcium Voltage-Gated Channel Auxiliary Subunit Gamma 5
3741.	CLINT1	Clathrin Interactor 1
3742.	COLEC12	Collectin Subfamily Member 12
3743.	CPLX2	Complexin 2
3744.	DISP1	Dispatched RND Transporter Family Member 1
3745.	DOC2A	Double C2 Domain Alpha
3746.	DYRK3	Dual Specificity Tyrosine Phosphorylation Regulated Kinase 3
3747.	E2F6	E2F Transcription Factor 6
3748.	EPN2	Epsin 2
3749.	FEZF1	FEZ Family Zinc Finger 1
3750.	GALNT17	Polypeptide N-Acetylgalactosaminyltransferase 17
3751.	INTU	Inturned Planar Cell Polarity Protein
3752.	KLF13	KLF Transcription Factor 13
3753.	KYAT1	Kynurenine Aminotransferase 1
3754.	MED12L	Mediator Complex Subunit 12L
3755.	MPRIP	Myosin Phosphatase Rho Interacting Protein
3756.	PDLIM5	PDZ And LIM Domain 5
3757.	PHF2	PHD Finger Protein 2
3758.	PIR	Pirin
3759.	PMF1	Polyamine Modulated Factor 1
3760.	PRELP	Proline And Arginine Rich End Leucine Rich Repeat Protein
3761.	RAB39B	RAB39B, Member RAS Oncogene Family
3762.	SCGN	Secretagogin, EF-Hand Calcium Binding Protein
3763.	SETD1B	SET Domain Containing 1B, Histone Lysine Methyltransferase

3764.	SLITRK5	SLIT And NTRK Like Family Member 5
3765.	SUPT16H	SPT16 Homolog, Facilitates Chromatin Remodeling Subunit
3766.	TOP3B	DNA Topoisomerase III Beta
3767.	VRK2	VRK Serine/Threonine Kinase 2
3768.	WAC	WW Domain Containing Adaptor With Coiled-Coil
3769.	AKAP10	A-Kinase Anchoring Protein 10
3770.	ARSK	Arylsulfatase Family Member K
3771.	ASMT	Acetylserotonin O-Methyltransferase
3772.	BAZ2B	Bromodomain Adjacent To Zinc Finger Domain 2B
3773.	CADPS2	Calcium Dependent Secretion Activator 2
3774.	CALY	Calcyon Neuron Specific Vesicular Protein
3775.	CETN1	Centrin 1
3776.	COL25A1	Collagen Type XXV Alpha 1 Chain
3777.	DLGAP4	DLG Associated Protein 4
3778.	DSCAML1	DS Cell Adhesion Molecule Like 1
3779.	EIF3C	Eukaryotic Translation Initiation Factor 3 Subunit C
3780.	ESS2	Ess-2 Splicing Factor Homolog
3781.	FARP2	FERM, ARH/RhoGEF And Pleckstrin Domain Protein 2
3782.	GABRQ	Gamma-Aminobutyric Acid Type A Receptor Subunit Theta
3783.	GPR17	G Protein-Coupled Receptor 17
3784.	GRIN3A	Glutamate Ionotropic Receptor NMDA Type Subunit 3A
3785.	IL31	Interleukin 31
3786.	IPO13	Importin 13
3787.	KCNN1	Potassium Calcium-Activated Channel Subfamily N Member 1
3788.	KLHL22	Kelch Like Family Member 22
3789.	LRFN5	Leucine Rich Repeat And Fibronectin Type III Domain Containing 5
3790.	NIPA2	NIPA Magnesium Transporter 2
3791.	NLGN4Y	Neuroligin 4 Y-Linked
3792.	PATZ1	POZ/BTB And AT Hook Containing Zinc Finger 1
3793.	RSRC1	Arginine And Serine Rich Coiled-Coil 1
3794.	SACS	Sacsin Molecular Chaperone
3795.	SCG3	Secretogranin III
3796.	SEZ6L	Seizure Related 6 Homolog Like
3797.	TSHZ3	Teashirt Zinc Finger Homeobox 3
3798.	YPEL3	Yippee Like 3
3799.	ZC3H14	Zinc Finger CCCH-Type Containing 14
3800.	ARC	Activity Regulated Cytoskeleton Associated Protein
3801.	ARHGAP18	Rho GTPase Activating Protein 18
3802.	CNTN5	Contactin 5
3803.	DDX52	DExD-Box Helicase 52
3804.	DHRS11	Dehydrogenase/Reductase 11

3805.	DRC3	Dynein Regulatory Complex Subunit 3
3806.	EIF3K	Eukaryotic Translation Initiation Factor 3 Subunit K
3807.	FEZ1	Fasciculation And Elongation Protein Zeta 1
3808.	FLG2	Filaggrin 2
3809.	HIRIP3	HIRA Interacting Protein 3
3810.	KANSL3	KAT8 Regulatory NSL Complex Subunit 3
3811.	MBD6	Methyl-CpG Binding Domain Protein 6
3812.	MDGA1	MAM Domain Containing Glycosylphosphatidylinositol Anchor 1
3813.	MTMR10	Myotubularin Related Protein 10
3814.	PLEKHB2	Pleckstrin Homology Domain Containing B2
3815.	SEC31B	SEC31 Homolog B, COPII Coat Complex Component
3816.	SLC12A8	Solute Carrier Family 12 Member 8
3817.	SOBP	Sine Oculis Binding Protein Homolog
3818.	TSNAX	Translin Associated Factor X
3819.	TXNDC9	Thioredoxin Domain Containing 9
3820.	UHMK1	U2AF Homology Motif Kinase 1
3821.	ZNF292	Zinc Finger Protein 292
3822.	ZNF711	Zinc Finger Protein 711
3823.	AMIGO1	Adhesion Molecule With Ig Like Domain 1
3824.	ATP13A4	ATPase 13A4
3825.	CNIH3	Cornichon Family AMPA Receptor Auxiliary Protein 3
3826.	ELMOD1	ELMO Domain Containing 1
3827.	ELMOD3	ELMO Domain Containing 3
3828.	GDPD3	Glycerophosphodiester Phosphodiesterase Domain Containing 3
3829.	HIVEP3	HIVEP Zinc Finger 3
3830.	KPTN	Kaptin, Actin Binding Protein
3831.	LIM2	Lens Intrinsic Membrane Protein 2
3832.	OSBPL7	Oxysterol Binding Protein Like 7
3833.	RBM12	RNA Binding Motif Protein 12
3834.	RPS27L	Ribosomal Protein S27 Like
3835.	TM4SF20	Transmembrane 4 L Six Family Member 20
3836.	ATP13A5	ATPase 13A5
3837.	CAPN12	Calpain 12
3838.	CARMIL2	Capping Protein Regulator And Myosin 1 Linker 2
3839.	CCDC91	Coiled-Coil Domain Containing 91
3840.	CLUL1	Clusterin Like 1
3841.	CMIP	C-Maf Inducing Protein
3842.	CNIH2	Cornichon Family AMPA Receptor Auxiliary Protein 2
3843.	DIP2C	Disco Interacting Protein 2 Homolog C
3844.	DUSP15	Dual Specificity Phosphatase 15
3845.	EIF2D	Eukaryotic Translation Initiation Factor 2D

3846.	FOXD4	Forkhead Box D4
3847.	FUNDC1	FUN14 Domain Containing 1
3848.	GPR89A	G Protein-Coupled Receptor 89A
3849.	GPR89B	G Protein-Coupled Receptor 89B
3850.	LRRC40	Leucine Rich Repeat Containing 40
3851.	PBLD	Phenazine Biosynthesis Like Protein Domain Containing
3852.	PCP4	Purkinje Cell Protein 4
3853.	PPP4R3A	Protein Phosphatase 4 Regulatory Subunit 3A
3854.	RAP2B	RAP2B, Member Of RAS Oncogene Family
3855.	RASGEF1B	RasGEF Domain Family Member 1B
3856.	WDR47	WD Repeat Domain 47
3857.	ZNF451	Zinc Finger Protein 451
3858.	ASPHD1	Aspartate Beta-Hydroxylase Domain Containing 1
3859.	CR1L	Complement C3b/C4b Receptor 1 Like
3860.	DGCR6L	DiGeorge Syndrome Critical Region Gene 6 Like
3861.	DIPK2B	Divergent Protein Kinase Domain 2B
3862.	LRRN2	Leucine Rich Repeat Neuronal 2
3863.	POU3F1	POU Class 3 Homeobox 1
3864.	RFX7	Regulatory Factor X7
3865.	TMEM87A	Transmembrane Protein 87A
3866.	ANKRD13B	Ankyrin Repeat Domain 13B
3867.	CDC42SE2	CDC42 Small Effector 2
3868.	EMX1	Empty Spiracles Homeobox 1
3869.	ENTREP2	Endosomal Transmembrane Epsin Interactor 2
3870.	GOT1L1	Glutamic-Oxaloacetic Transaminase 1 Like 1
3871.	GPR153	G Protein-Coupled Receptor 153
3872.	GPR34	G Protein-Coupled Receptor 34
3873.	GSTT1	Glutathione S-Transferase Theta 1
3874.	INO80E	INO80 Complex Subunit E
3875.	IQCK	IQ Motif Containing K
3876.	MAP6	Microtubule Associated Protein 6
3877.	MYO16	Myosin XVI
3878.	NBPF3	NBPF Member 3
3879.	NTAN1	N-Terminal Asparagine Amidase
3880.	OXNAD1	Oxidoreductase NAD Binding Domain Containing 1
3881.	PCMTD2	Protein-L-Isoaspartate (D-Aspartate) O-Methyltransferase Domain Containing 2
3882.	TAAR6	Trace Amine Associated Receptor 6
3883.	TSC22D2	TSC22 Domain Family Member 2
3884.	C16orf54	Chromosome 16 Open Reading Frame 54
3885.	CEP20	Centrosomal Protein 20
3886.	CNIH1	Cornichon Family AMPA Receptor Auxiliary Protein 1

3887.	CRYGA	Crystallin Gamma A
3888.	DNAJC14	DnaJ Heat Shock Protein Family (Hsp40) Member C14
3889.	GGNBP2	Gametogenetin Binding Protein 2
3890.	GJD4	Gap Junction Protein Delta 4
3891.	GPR78	G Protein-Coupled Receptor 78
3892.	IGSF5	Immunoglobulin Superfamily Member 5
3893.	TSPAN10	Tetraspanin 10
3894.	WBP1L	WW Domain Binding Protein 1 Like
3895.	AMY1A	Amylase Alpha 1A
3896.	ANKAR	Ankyrin And Armadillo Repeat Containing
3897.	BMERB1	BMERB Domain Containing 1
3898.	BRI3	Brain Protein I3
3899.	C17orf78	Chromosome 17 Open Reading Frame 78
3900.	CIBAR2	CBY1 Interacting BAR Domain Containing 2
3901.	ERMARD	ER Membrane Associated RNA Degradation
3902.	GJA9	Gap Junction Protein Alpha 9
3903.	IGLL5	Immunoglobulin Lambda Like Polypeptide 5
3904.	NOTCH2NLA	Notch 2 N-Terminal Like A
3905.	OR6A2	Olfactory Receptor Family 6 Subfamily A Member 2
3906.	TIGD4	Tigger Transposable Element Derived 4
3907.	TSNARE1	T-SNARE Domain Containing 1
3908.	ZNF674	Zinc Finger Protein 674
3909.	BOLA2B	BolA Family Member 2B
3910.	CNPY1	Canopy FGF Signaling Regulator 1
3911.	WASHC1	WASH Complex Subunit 1
3912.	ARHGAP11B	Rho GTPase Activating Protein 11B
3913.	CARMIL3	Capping Protein Regulator And Myosin 1 Linker 3
3914.	FPGT	Fucose-1-Phosphate Guanylyltransferase
3915.	H4C13	H4 Clustered Histone 13
3916.	INO80B	INO80 Complex Subunit B
3917.	ISX	Intestine Specific Homeobox
3918.	NBPF6	NBPF Member 6
3919.	PRDM11	PR/SET Domain 11
3920.	AMY1C	Amylase Alpha 1C
3921.	CYP27C1	Cytochrome P450 Family 27 Subfamily C Member 1
3922.	GJB7	Gap Junction Protein Beta 7
3923.	HGH1	HGH1 Homolog
3924.	RAB40AL	RAB40A Like
3925.	TIGD3	Tigger Transposable Element Derived 3
3926.	AMY1B	Amylase Alpha 1B
3927.	NBPF12	NBPF Member 12

3928.	NBPF10	NBPF Member 10
3929.	NBPF4	NBPF Member 4
3930.	OR9I1	Olfactory Receptor Family 9 Subfamily I Member 1
3931.	POM121L12	POM121 Transmembrane Nucleoporin Like 12
3932.	RTL8B	Retrotransposon Gag Like 8B
3933.	NBPF14	NBPF Member 14
3934.	VSTM5	V-Set And Transmembrane Domain Containing 5
3935.	GOLGA6B	Golgin A6 Family Member B
3936.	SPACA5	Sperm Acrosome Associated 5
3937.	HSFX2	Heat Shock Transcription Factor Family, X-Linked 2
3938.	NBPF9	NBPF Member 9
3939.	TSPY2	Testis Specific Protein Y-Linked 2
3940.	PMF1-BGLAP	PMF1-BGLAP Readthrough
3941.	USP17L3	Ubiquitin Specific Peptidase 17 Like Family Member 3
3942.	RFPL4AL1	Ret Finger Protein Like 4A Like 1
3943.	GOLGA8T	Golgin A8 Family Member T
3944.	PRR20B	Proline Rich 20B
3945.	PRR20C	Proline Rich 20C
3946.	LOC647264	Keratin-Associated Protein 21-1
3947.	FGFR3	Fibroblast Growth Factor Receptor 3
3948.	HRAS	HRas Proto-Oncogene, GTPase
3949.	TUBB	Tubulin Beta Class I
3950.	CSTB	Cystatin B
3951.	CTSV	Cathepsin V
3952.	SUPV3L1	Suv3 Like RNA Helicase
3953.	IRX5	Iroquois Homeobox 5
3954.	ASIC5	Acid Sensing Ion Channel Subunit Family Member 5
3955.	GABPA	GA Binding Protein Transcription Factor Subunit Alpha
3956.	IRX6	Iroquois Homeobox 6

Supplementary Table S6. MPXV-Human Gene Interactions and Clade Specificity based on literature.

**MPXV Gene**	**Human Gene**	**Function of MPXV protein**	**Strain/Clade**	**Reference**
MOPICE	CFH	Inhibits complement system (binds C3b/C4b)	Congo Basin (Clade I)	(Liszewski et al., 2006; Estep et al., 2011; Hudson et al., 2012)
A46R	NFKB1	Inhibits TLR/NF-κB signaling	Clade I & II	(Chen et al., 2005)
C23L	CXCL8	Chemokine mimic; modulates leukocyte chemotaxis	Clade I	(Reading et al., 2003)

References

Chen N, Li G, Liszewski M K, Atkinson J P, Jahrling P B, Feng Z, Schriewer J, Buck C, Wang C, Lefkowitz E J (2005). Virulence differences between monkeypox virus isolates from West Africa and the Congo basin. Virology, 340(1): 46-63

Estep R D, Messaoudi I, O'connor M A, Li H, Sprague J, Barron A, Engelmann F, Yen B, Powers M F, Jones J M, Robinson B A, Orzechowska B U, Manoharan M, Legasse A, Planer S, Wilk J, Axthelm M K, Wong S W (2011). Deletion of the monkeypox virus inhibitor of complement enzymes locus impacts the adaptive immune response to monkeypox virus in a nonhuman primate model of infection. J Virol, 85(18): 9527-9542

Hudson P N, Self J, Weiss S, Braden Z, Xiao Y, Girgis N M, Emerson G, Hughes C, Sammons S A, Isaacs S N, Damon I K, Olson V A (2012). Elucidating the Role of the Complement Control Protein in Monkeypox Pathogenicity. PLoS One, 7(4): e35086

Liszewski M K, Leung M K, Hauhart R, Buller R M, Bertram P, Wang X, Rosengard A M, Kotwal G J, Atkinson J P (2006). Structure and regulatory profile of the monkeypox inhibitor of complement: comparison to homologs in vaccinia and variola and evidence for dimer formation. J Immunol, 176(6): 3725-3734

Reading P C, Symons J A, Smith G L (2003). A soluble chemokine-binding protein from vaccinia virus reduces virus virulence and the inflammatory response to infection. J Immunol, 170(3): 1435-1442
